# Dehydroxylative radical N-glycosylation of heterocycles with 1-hydroxycarbohydrates enabled by copper metallaphotoredox catalysis

**DOI:** 10.1038/s41467-024-47711-9

**Published:** 2024-04-22

**Authors:** Da-Peng Liu, Xiao-Sen Zhang, Shuai Liu, Xiang-Guo Hu

**Affiliations:** National Engineering Research Center for Carbohydrate Synthesis, Jiangxi Normal University, Nanchang, 330022 China

**Keywords:** Carbohydrate chemistry, Synthetic chemistry methodology, Synthetic chemistry methodology

## Abstract

N-Glycosylated heterocycles play important roles in biological systems and drug development. The synthesis of these compounds heavily relies on ionic N-glycosylation, which is usually constrained by factors such as labile glycosyl donors, precious metal catalysts, and stringent conditions. Herein, we report a dehydroxylative radical method for synthesizing *N*-glycosides by leveraging copper metallaphotoredox catalysis, in which stable and readily available 1-hydroxy carbohydrates are activated for direct N-glycosylation. Our method employs inexpensive photo- and copper- catalysts and can tolerate some extent of water. The reaction exhibits a broad substrate scope, encompassing 76 examples, and demonstrates high stereoselectivity, favoring 1,2-*trans* selectivity for furanoses and α-selectivity for pyranoses. It also exhibits high site-selectivity for substrates containing multiple N-atoms. The synthetic utility is showcased through the late-stage functionalization of bioactive compounds and pharmaceuticals like Olaparib, Axitinib, and Metaxalone. Mechanistic studies prove the presence of glycosyl radicals and the importance of copper metallaphotoredox catalysis.

## Abstract (web summary)

N-Glycosylated heterocycles play important roles in biological systems and drug development, but the synthesis heavily relies on ionic N-glycosylation. Herein, the authors report a dehydroxylative radical method for synthesizing N-glycosides by leveraging copper metallaphotoredox catalysis.

## Introduction

**Fig. 1:**
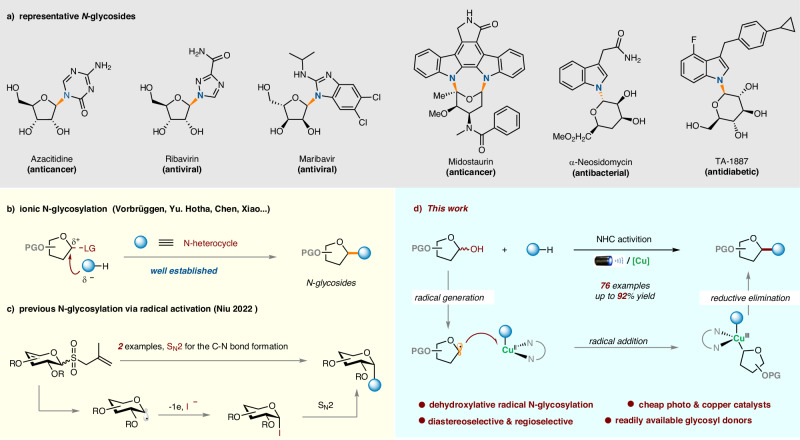
The importance of N-glycosides and the background of this research. **a** Representative examples N-glycosides as marketed drugs and bioactive compounds; **b** Selected examples of ionic glycosylation; **c** previous N-glycosylation via radical activation; **d** this work: dehydroxylative radical N-glycosylation.

N-glycosylated heterocycles are important in biological systems and drug development. For instance, N-glycosylated (ribosylated) purines or pyrimidines constitute the main components of DNA/RNA. Moreover, these heterocycles are common motifs in marketed drugs, natural products, and synthetic bioactive compounds (Fig. 1a)^1–4^. Therefore, their synthesis is widely recognized as a key objective for carbohydrate synthesis in laboratory and industrial settings.

Despite the existence of other methods, including de novo approaches and the coupling reactions of glycosyl amines^5–9^, N-glycosylation offers a straightforward strategy to access N-glycosylated heterocycles due to the ubiquity of starting materials ^10^ N-glycosylation typically occurs through an ionic process, in which the glycosyl donor undergoes activation to generate a highly electrophilic intermediate (e.g., oxocarbenium) and then react with an N-heterocycle (Fig. 1b). Techniques following this activation mode typically necessitate the use of labile glycosyl donors and harsh reaction conditions, as exemplified by elevated temperatures in fusion reactions^11^_,_ labile glycosyl halides and highly basic conditions in metal salt-based methods^12^, and acidic conditions in Vorbrüggen reactions, thus causing functionality compatibility problems (Fig. 1b)^13^. Recent advancements by Yu^14,15^, Hotha^16^, Chen^17^ and Xiao^18^ have facilitated N-glycosylation under significantly milder conditions, offering dependable tools for addressing diverse synthetic challenges (Fig. 1b)^19–22^. However, costly catalysts and/or requisite glycosyl donors are still required in these approaches. Therefore, it remains highly desirable to develop general N-glycosylation methods that use stable glycosyl donors, circumvent the use of expensive catalysts or promoters, and avoid harsh activation conditions.

The development of mechanistically distinct glycosylation methods may provide opportunities to address some of the aforementioned limitations. Considering the mild conditions, great functionality tolerance, and unique stereoselectivity, a glycosyl radical-based approach would be intriguing^23–25^. However, great challenges exist for realizing such an N-glycosylation, including common issues such as stereoselectivity and regioselectivity, as well as the tendency of glycosyl radicals to undergo homocoupling^26,27^, oxidation^28^ or reduction^29^. Consequently, while the synthesis of C-glycosides^23–25^ and S-glycosides^30–32^ has been well-established, research on corresponding N-glycosylation remains virtually untouched. Recently, Niu et al. demonstrated the possibility of radical N-glycosylation by utilizing allyl glycosyl sulfones as glycosyl donors^28^. However, their investigation was limited to only two substrates, and the formation of the C-N bond still followed an ionic S_N_2 process via glycosyl iodides (Fig. 1c)^28^.

Inspired by recent advances in copper-catalyzed C-N coupling reaction^33–39^ and dehydroxylative coupling reactions^40^, specifically employing metallaphotoredox catalysis^41^, we sought to develop a copper-catalyzed, dehydroxylative radical N-glycosylation as an alternative to conventional ionic approaches. In our working hypothesis (Fig. 1d), we envisioned that a glycosyl radical could be generated from a 1-hydroxycarbohydrate after proper activation^40^ under mildly photoredox conditions^23–25,42^. It can then capture the copper (II)-amido complex efficiently^43^. Subsequently, the resulting high-valent Cu(III) species can undergo facile reductive elimination to yield the desired N-glycosides^44,45^. We report herein the successful realization of this hypothesis (Fig. 1c). Merits of this work include i) mechanistically distinct, dehydroxylative radical N-glycosylation; ii) readily available and stable 1-hydroxycarbohydrates as the glycosyl donors and inexpensive copper catalyst; iii) highly diastereoselective and regioselective for a broad substrate scope.

## Results and discussion

Though alkyl halides and carboxylic acids have been extensively studied in C-N coupling reactions^33–39^, the corresponding glycosyl counterparts are either unstable or difficult to prepare^46^. Therefore, our objective was to directly activate 1-hydroxycarbohydrates to form glycosyl radicals. Following a preliminary investigation of hemioxalate^47^, dihydropyridine (DHP) ester^48,49^ and xanthate salt^50^ activation techniques (see section 2.4 in Supplementary Information), we decided to use the *N*-heterocyclic carbene (NHC) activation method developed by MacMillan and co-workers (Supplementary Table 13)^51–53^. This method does not require isolation or workup to prepare the NHC-adduct, making it the preferred choice.

After a comprehensive screening process, we successfully identified the optimal reaction conditions, leading to a yield of 87% for 0.1 mmol and 74% for 1 mmol scale of the target product **2a** (see Supplementary Table 1–14 and section 2.3 in Supplementary Information). The reaction protocol consisted of using [Cu(CH_3_CN)_4_]PF_6_ as the catalyst, 4,4’-di-*tert*-butyl-2,2’-bipyridine (dtbbpy) as the ligand, 2,4,5,6-Tetra(9H-carbazol-9-yl)isophthalonitrile (4CzIPN) as the photocatalyst, CsOAc as the base, and *t*-BuOOH as the oxidant in MeCN (entry 1, Table 1). Control experiments demonstrated that copper catalyst, photocatalyst, base, oxidant, light and ligand were all indispensable for the reaction (entries 2–7). Replacement of [Cu(CH_3_CN)_4_]PF_6_ with Cu(TMHD)_2_ yielded the product in 66% yield as a mixture of N1/N2 regioisomers (entry 8). Regioselectivity was not compromised when 1,10-Phenanthroline (Bphen) was used, but the yield decreased (entry 9). Other bases such as 2-*tert*-butyl-1,1,3,3-tetramethylguanidine (BTMG) displayed unsuitability for the reaction (entry 10). Though cumyl trimethylsilyl peroxide (cumOOTMS) and Ir-based photosensitizer proved to be suitable for the reaction, they are not selected due to their high cost (entries 11-12). The reaction efficiency significantly decreased in DMSO (entry 13). Moreover, the reaction exhibited tolerance to temperature variations (up to 50 °C) and moisture (entries14–16); 78% yield was obtained with the deliberate addition of 10 equivalents of water. The fact that the tolerance of some extent of water, a potential advantage relative to ionic N-glycosylation, is not clear at the moment^54^. Other glycosyl donors derived from **1a** such as **1c**-**1e** cannot afford any products under the optimized conditions (entries 17-19). Finally, common alcohols (**1f**-**1g**) could not react under the optimized conditions, suggesting the unique role of glycosyl radicals in this transformation. It is noteworthy that while 1-hydroxy carbohydrates have been utilized as glycosyl donors for O-^55^ and C-glycosylation^51^, we demonstrate the successful application of 1-hydroxy carbohydrates for N-glycosylation in this work.

**Table 1:** Optimization of the Reaction Conditions^a^


Entry	Variation from theoptimal conditions	Yield(%)^[c]^	Entry	Variation from the optimal conditions	Yield(%)^[c]^
1	none	87^b^ (85^c^_,_ 74%^d^)	11	Ir[dF(CF_3_)ppy]_2_(dtbppy)PF_6_	84
2	No [Cu]	N.D.	12	cumOOTMS as oxidant	81
3	No base	N.D.	13	DMSO as solvent	55
4	No PC	trace	14	50 °C	76
5	No [O]	N.D.	15	under air	86
6	No light	trace	16	10 equiv H_2_O	78
7	No ligand	71 (N1:N2 = 9:1)	17	**1c** instead of **1a** + NHC	N.D.
8	Cu(TMHD)_2_ as catalyst	66 (N1:N2 = 3:2)	18	**1d** instead of **1a** + NHC	N.D.
9	Bphen as ligand	75	19	**1e** instead of **1a** + NHC	trace
10	BTMG as base	57	20	**1** **f, 1** **g** instead of **1a** + NHC	N.D.

^a^Reactions were conducted on 0.1 mmol scale. ^b^Yields were determined by ^1^H NMR, using 3,5-bis(trifluoromethyl)bromobenzene as the internal standard. ^c^Isolated yield. ^d^ Isolated yield on 1 mmol scale.

**Fig. 2:**
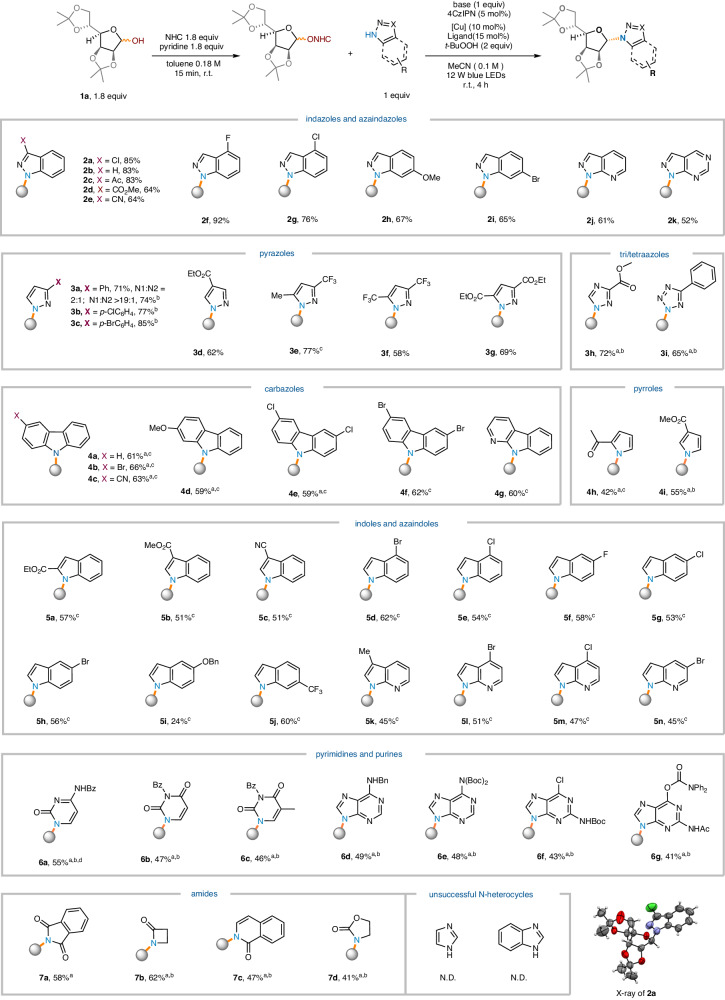
The substrate scope with respect to N-heterocycles. Conditions: ^a^ TMG as base. ^b^ CuTc (10 mol%), Bphen (10 mol%). ^c^ Cu(TMHD)_2_ (10 mol%). no ligand. ^d^ DMSO as solvent. N.D. Not detected, Ac Acetyl, Bz Benzoyl, Bn benzyl, Boc *t*-Butyloxy carbonyl.

After determining the optimized conditions, we proceeded to investigate the range of N-heterocycles using furanose **1a** (Fig. 2). To our delight, the reaction is successful with over 10 different N-heterocycles, including indazole (**2a**-**2i**), azaindazole (**2j-2k**), pyrazole (**3a-3g**), triazole (**3** **h**), tetrazole (**3i**), carbazole (**4a**-**4g**), pyrrole (**4h**-**4i**), indole (**5a**-**5j**), azaindole (**5k**-**5n**), pyrimidine (**6a**-**6c**), and purine (**6d**-**6g**), cyclic imide (**7a**), β-lactam (**7b**), isoquininone (**7c**), and cyclic carbamate (**7d**). α-*N*-glycosides were consistently observed in all cases, showing 1,2-*trans* selectivity similar to other glycosyl radical-based glycosylations for furanose substrates^56–61^, likely due to steric effects. Different from indazoles, pyrazoles proved to be problematic substrates, resulting in the formation of 2:1 regioisomers (**3a**). This issue was found to be solvable through re-optimization using the copper(I) thiophene-2-carboxylate (CuTc) and 4,7-diphenyl-1,10-phenanthroline (Bpen) catalytic system (regioselectivity>20:1). Other heterocycles containing multiple N-atoms exhibited excellent regioselectivity (e.g. **3b-3e, 3** **h, 3i, 6d-6g**). Importantly, purines yielded only N9-glycosides without any N7-side products (**6d-6g**)^7^. The reaction conditions are mild and can accommodate various functionalities, including halo (**2a, 2** **f, 2** **g, 2i, 4b, 4e-4f, 5d-5h, 5l-5n**), nitrile (**2e,** **4c,** **5c**), ester (**2d,** **3d,** **3** **g, 3** **h,** **4i,** **5a,** **5b**), keto (**2c,** **4** **h**), ether (2 h, **4d,** **5i**), trifluoromethyl (**3e, 3** **f, 5j**), and common protecting groups [N-Bz (**6a**-**6c**), N-Boc (**6e, 6** **f**), N-Bn (**6d**), O-carbamate (**6** **g**)]. It is worth noting that the bromo-group remains intact (**2i,** **4b,** **4** **f,** **5d,** **5** **h,** **5i**), providing a reactive site for subsequent derivatization through other metal-catalyzed cross-coupling reactions. Our protocol provides a convenient alternative for accessing certain significant N-glycosides. For instance, glycosyl carbazoles usually require the utilization of 1,2-anhydro sugars as glycosyl donors followed by the construction of the carbazole or a de novo synthesis of the sugar ring^62,63^. Furthermore, to our knowledge, there is no prior report on the direct N-glycosylation of β-lactam, a privileged motif found in antibiotics. Finally, it should be noted that our method has certain limitations; in particular, (benzo)imidazole cannot be involved, and indoles with electron-rich groups are not able to efficiently participate in this reaction, possibly due to the decreased acidity of N-H group.

**Fig. 3:**
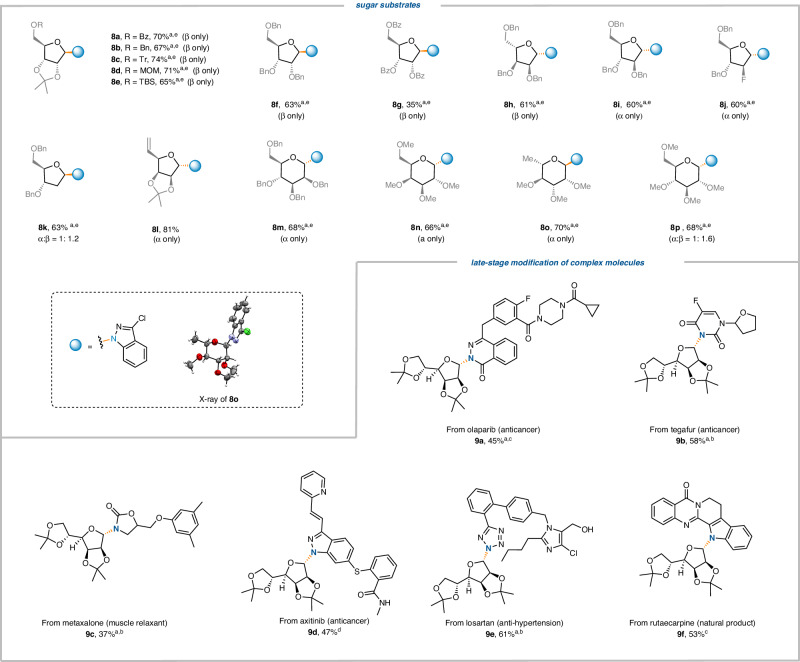
The substrate scope with respect to sugar and the late-stage modification. Conditions: ^a^ TMG as base. ^b^ CuTc (10 mol%), Bphen (10 mol%). ^c^ Cu(TMHD)_2_ (10 mol%). no ligand. ^d^ DMSO as solvent. ^e^ at 0 °C. Bz Benzoyl, Bn benzyl, Tr Trityl, MOM Methoxymethyl.

We moved on to explore the scope of sugar substrates with **1b** unchanged (Fig. 3). Due to the tendency of NHC-adducts derived from sugars other than **1a** to decompose at room temperature, we conducted the reaction at 0 °C to ensure its effectiveness. Furanose derivatives with a range of protecting groups consistently produced 1,2-*trans* products (**8a**-**8l**), except for 2-deoxyribose lacking a substituent at C2 (**8k**). Intriguingly, the presence of a small fluorine atom can restore the *trans*-selectivity, possibly due to the fluorine conformational effect (**8j**)^64^. The electron-rich protecting group is beneficial to the reaction, and perbenzoyl protected **8** **g** was obtained in a much lower yield than **8** **h**. This argument is also supported by the fact that acyl-protected pyranoses do not participate in the desired transformation, while only those with ether-type (e.g. OMe) protecting groups do (**8m**-**8p**). Among the pyranose series, substrates derived from, mannose (**8** **m**), galactose (**8n**), and rhamnose (**8o**) predominantly yielded the α-isomer. This can be explained by stereoelectronic effect^65–67^, which refers to the hyperconjugative interaction between the lone pair of the endocyclic oxygen and the low-lying C−Cu antibonding orbitals. This interaction leads to the preference of the copper species at the axial position, resulting in high α-stereoselectivity. The glucose-derived substrate was obtained as a mixture of anomers (**8p**), perhaps due to the glucosyl radical adopting a *B*_25_ conformation, in which the steric hindrance of C2-OMe reduces α-selectivity^65,66^. Nevertheless, it is important to recall that highly β-stereoselective C-glycosylation with glucosyl substrates can be achieved under Ni-catalyzed conditions^68,69^.

To demonstrate the applicability of this N-glycosylation protocol, we sought to functionalize a wide variety of structurally complex molecules in a late-stage fashion. Marketed anticancer drugs, including Olaparib (**9a**), Tegafur (**9b**), and Axitinib (**9d**), underwent successful conversion to the corresponding *N*-glycosides with yields ranging from 47% to 58% as the exclusive α-isomer. Metaxalone, a muscle relaxant (**9c**) and Losartan (**9e**), an anti-hypertension drug, are also viable substrates. Interestingly, the latter compound demonstrated that the presence of a free alcohol group does not affect the reaction efficiency. Finally, the natural product rutecarpine, derived from the traditional Chinese medicine *fructus evodiae*, underwent efficient N-glycosylation, resulting in the formation of (**9f**) with a 53% yield.

**Fig. 4:**
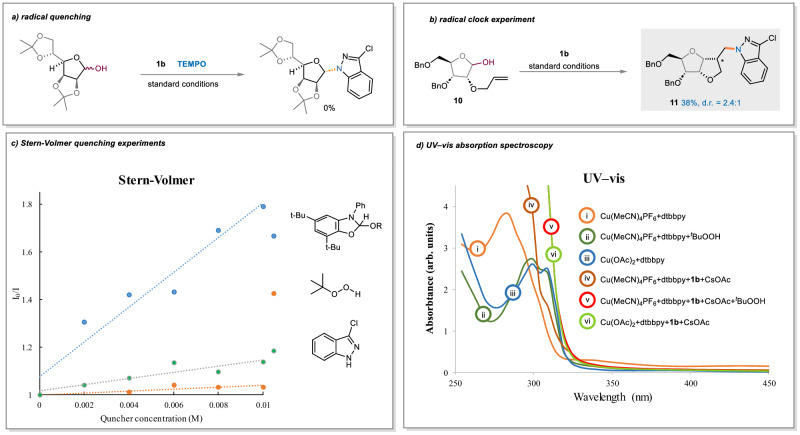
Mechanistic Studies. **a** The radical quenching experiment. **b** The radical clock experiment. **c** Stern-Volmer quenching experiments. **d** UV-vis absorption spectroscopy.

To understand the reaction pathway, we have conducted a series of mechanistic studies (see section 2.5 in Supplementary Information). The reaction was completely shut down by the addition of TEMPO, thus suggesting a possible radical pathway (Fig. 4a). When **10** with an allyl group at O-2 position was used, diastereomers of **11** were obtained in 38% yield, which presumably arises from 5-*exo*-trig cyclization of the radical intermediate (Fig. 4b). This radical clock reaction clearly proved the involvement of glycosyl radical intermediates. The reaction cannot occur without photosentizer or light (Entries 3, 5, Table 1). Furthermore, the Stern–Volmer experiments showed that NHC-alcohol adduct quenched the excited state photocatalyst (PC*) significantly faster than other reagents such as an indazole (Fig. 4c). These experiments, along with the control experiments listed in the reaction optimization section (Table 1), suggested glycosyl radicals are likely generated by photoactivation.

We next conducted UV–vis absorption spectroscopy and cyclic voltammetry experiments (see section 2.5.3-2.5.4 in Supplementary Information) to get some insight on the oxidation of Cu (I) to Cu (II) in the copper catalytic cycle (Fig. 4d). According to the UV/Vis studies, reaction of LnCu(I) with *t*-BuOOH generated a new copper species (i→ii), which has a nearly identical profile as LnCu(II) (iii). The oxidation of LnCu(I)-amido to LnCu(II)-amido (iv→v) is also feasible, probably easier than the oxidation of LnCu(I) to LnCu(II), as shown by the CV experiment (Supplementary Fig. 7).

**Fig. 5:**
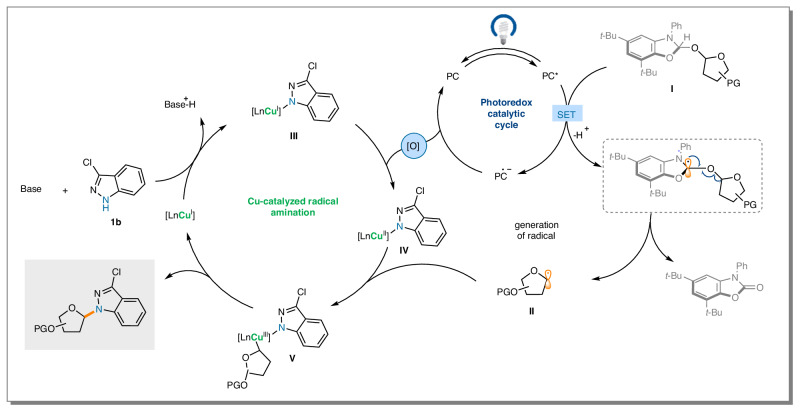
Proposed Mechanism for the dehydroxylative radical N-glycosylation.

On the basis of the conducted experiments, we proposed a plausible mechanism as illustrated in Fig. 5. The reaction is initiated by the Single Electron Transfer (SET) oxidation of the NHC adduct **I** with 4CzIPN^51^, resulting in the formation of glycosyl radical **II**. In the copper catalytic cycle, the ligand change reaction of the copper catalyst with an N-heterocycle (e.g., **1b**) generates copper (I)-amido complex **III**. This species is then oxidized by the oxidant (*t*BuOOH), leading to the formation of copper (II)-amido species **IV**. The glycosyl radical is then captured by **IV**, yielding copper (III) complex **V**^43^, which gives the desired product through reductive elimination^44,45^, thus regenerating the copper catalyst and closing the copper catalytic cycle. The photoredox cycle is closed by the oxidation of the reduced form of the photocacalyst (PC-) with the oxidant (*t*BuOOH), followed by sensitization.

In summary, we have developed a dehydroxylative radical method for synthesizing *N*-glycosides by leveraging copper metallaphotoredox catalysis, in which stable and readily available 1-hydroxy carbohydrates are directly activated for N-glycosylation. Complementing with the well-established ionic approaches, our method employs inexpensive photo- and copper- catalysts and can tolerate some extent of water. The reaction exhibits a broad substrate scope, encompassing 76 examples, and demonstrates high stereoselectivity, favoring 1,2-*trans* selectivity for furanoses and α-selectivity for pyranoses. It also exhibits high site-selectivity for substrates containing multiple N-atoms. The synthetic utility was showcased through the late-stage functionalization of bioactive compounds and pharmaceuticals like Olaparib, Axitinib, and Metaxalone. The presence of glycosyl radicals was confirmed through radical suppressing reactions and a radical clock reaction. Additionally, the importance of copper metallaphotoredox catalysis was demonstrated through control experiments and various spectroscopic studies, such as UV-vis experiments. Though limitations, such as unsuitability for certain electron-rich hetercoycles and electron-deficient sugar substrates, still exist, we believe this work will stimulate more research in the radical N-glycosylation for the preparation of valuable *N*-glycosides that are difficult-to-made in future.

## Methods

For ^1^H, ^13^C, and ^19^F nuclear magnetic resonance (NMR) spectra of compounds in this manuscript and details of the synthetic procedures as well as more reaction condition screening, see Supplementary Information.

### General procedure for 2a-2k, 3d, 3f, 3g, 8l. For more substrate procedures see Supplementary Information

An oven-dried 10 mL Schlenk tube was charged with 1-hydroxylmannose **1a** (93.6 mg, 0.36 mmol, 1.8 equiv), NHC (142.4 mg, 0.36 mmol, 1.8 equiv) and a magnetic stir bar. After the Schlenk tube was vacuumed and refilled with nitrogen gas three times, dry toluene (2.0 mL) was added and the reaction was stirred at r.t. for 5 min. Then, pyridine (29.1 µL, 0.36 mmol, 1.8 equiv) was added dropwise at room temperature. The resulting solution was stirred at r.t. for 10 min. A white solid precipitated out during this time. Another 10 mL Schlenk tube was charged with 1,2,3,5-Tetrakis(carbazol-9-yl)−4,6-dicyanobenzene (4CzIPN, 5 mg, 0.01 mmol, 0.05 equiv), Cu(MeCN)_4_PF_6_ (7.4 mg, 0.02 mmol, 0.1 equiv), dtbbpy (4,4-di-*tert*-butyl bipyridine, 8.0 mg, 0.03 mmol, 0.15 equiv), CsOAc (38.2 mg, 0.2 mmol, 1.0 equiv), *N*-heterocycle (0.2 mmol, 1.0 equiv) and a magnetic stir bar. This Schlenk tube was vacuumed and refilled with nitrogen gas three times. Dry acetonitrile (2.0 mL) was added to this Schlenk tube under an atmosphere of nitrogen and stirred at room temperature. The toluene suspension was transferred to a 5 mL syringe under an atmosphere of nitrogen. Then a syringe filter and new needle were installed on the syringe, and the toluene solution was injected through the syringe filter into the MeCN solution. *t*-BuOOH (80 μL, 5-6 M in decane, 2.0 equiv) was added, before subjecting the reaction mixture to irradiation by 420 nm blue LEDs at room temperature for a duration of 4 hours. The organic layers were evaporated and then purified by flash column chromatography on silica gel.

### Supplementary information

Supplementary Information

Peer Review File

## Data Availability

All data supporting the findings of this study are available within the Article and its Supplementary Information, and are also available from the corresponding author upon request. Crystallographic data for the structure (**2a, 8o**) reported in this Article have been deposited at the Cambridge Crystallographic Data Centre, under deposition number CCDC 2305000, 2305001. Copies of the data can be obtained free of charge via https://www.ccdc.cam.ac.uk/structures/.
