# Supplementary material for: Dehydroxylative radical N-glycosylation of heterocycles with 1-hydroxycarbohydrates enabled by copper metallaphotoredox catalysis
Source: Nat Commun. 2024 Apr 22;15:3401. doi: 10.1038/s41467-024-47711-9 (PMC11035684; doi:10.1038/s41467-024-47711-9)
Supplement: Supplementary file 1 — Supplementary Information [file 41467_2024_47711_MOESM1_ESM.pdf]

Supplementary Information

Dehydroxylative Radical N-Glycosylation of Heterocycles with 1-Hydroxycarbohydrates Enabled by Metallaphotoredox Catalysis

Da-Peng Liu, Xiao-Sen Zhang, Shuai Liu, Xiang-Guo Hu*

National Engineering Research Center for Carbohydrate Synthesis, Jiangxi Normal University, Nanchang, 330022, China. E-mail: Xiangguo.hu@jxnu.edu.cn

Contents

1. General information 2
2. Experimental section 3
    2.1 Optimization of the reaction conditions 3
    2.2 General procedures for N-glycosylation of heterocycles 11
    2.3 N-glycosylation procedure on 1 mmol scale 43
    2.4 Oxalate, DHP ester, xanthate salt as glycosyl donors 44
    2.5 Mechanistic studies 45
        2.5.1 Radical trapping experiments 45
        2.5.2 Radical clock experiments 46
        2.5.2 Cyclic voltammetry data 49
        2.5.3 Stern-Volmer experiment 50
        2.5.4 UV-Vis spectra 51
        2.5.5 Oxidation of copper salts 52
3. X-ray crystal structure data 53
4. Structure Determination 56
    4.1 Determination of the anomeric stereochemistry 56
    4.2 Determination of the regioisomer 64
5. Copies of NMR spectra 70
6. References 176

1. General information

All purchased reagents were used without further purification unless otherwise specified. Solvents were dried over 4Å molecular sieves before use, unless stated otherwise. Reactions were stirred using Teflon-coated magnetic stirrers. Analytical thin-layer chromatography (TLC) was performed using 0.20 mm silica gel 60F plates with a 254 nm fluorescent indicator. TLC plates were visualized by ultraviolet light or by treatment with a spray of Pancaldi reagent {$(NH_4)_6MoO_4$, $Ce(SO_4)_2$, H_2SO_4, H_2O}, or a 10% H_2SO_4 in ethanol solution. Products were purified through flash column chromatography on silica gel (200-300 mesh). Melting points were determined using a WRX-4 visual melting point apparatus. Both melting points and boiling points are uncorrected. Infrared spectra were recorded using an IR Affinity-1 spectrometer. Nuclear magnetic resonance spectra (^{1}H NMR, ^{13}C NMR, and ^{19}F NMR) were acquired using a Bruker AV 400 spectrometer operating at the following frequencies: 400 MHz for ^{1}H, 100 MHz for ^{13}C, and 376 MHz for ^{19}F. Chemical shifts (δ) are reported in ppm, and coupling constants (*J*) are in Hz. The following abbreviations were used for multiplicities: s = singlet, d = doublet, t = triplet, q = quartet, m = multiplet. ESI mass spectra were recorded on an AB sciex Triple-TOF 5600+ mass instrument. The samples for the irradiation reaction were positioned at a distance of 5 cm from a 40 W blue LED lamp (λmax = 420 nm, purchased from Shenzhen Hongye Optoelectronics Technology Corporation). Alternatively, a parallel light low-temperature reactor (purchased from Shanghai Shanshi Technology Corporation) was also utilized. The reaction temperature was maintained at approximately 25ºC by fans or 0ºC by a low-temperature reactor.

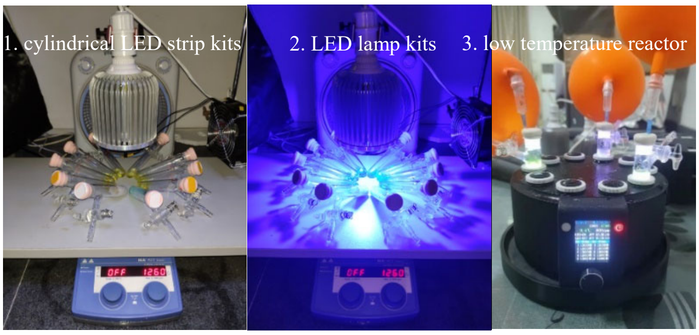

Supplementary Figure 1 Reaction setup. 1. cylindrical LED strip kits. 2. LED lamp kits. 3. low temperature reactor

2. Experimental section

2.1 Optimization of the reaction conditions

Supplementary Table 1 | Evaluation of copper catalyst

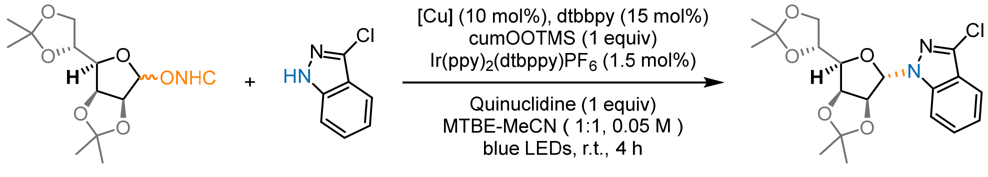

1a' **1b** **2a**

Entry	Copper source	Yield (%)
1	$CuBr_2$	31
2	CuBr	35
3	CuTc	32
4	$Cu(hfac)_2$	38
5	$Cu(MeCN)_4PF_6$	42
6	$Cu(OAc)_2$	39
7	$Cu(acac)_2$	9
8	$Cu(OTf)_2$	30
9	$Cu(TMHD)_2$	42

Supplementary Table 2 | Evaluation of base

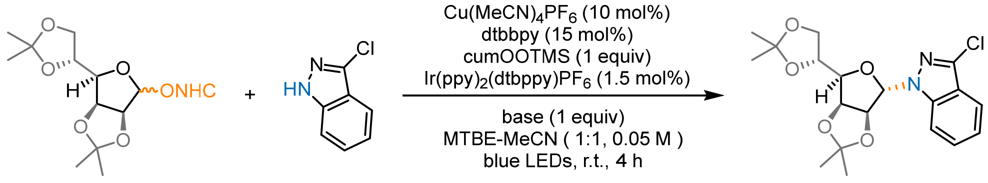

1a' **1b** **2a**

Entry	Base	Yield (%)
1	Quinuclidne	42
2	NaOAc	27
3	CsOAc	43
4	Cs_2CO_3	12
5	K_3PO_4	12
6	*t*-BuOLi	12

7	DABCO	11
8	CsF	13
9	Et_3N	—
10	DBU	9
11	TMG	41
12	BTMG	10

Supplementary Table 3 | Evaluation of solvent B

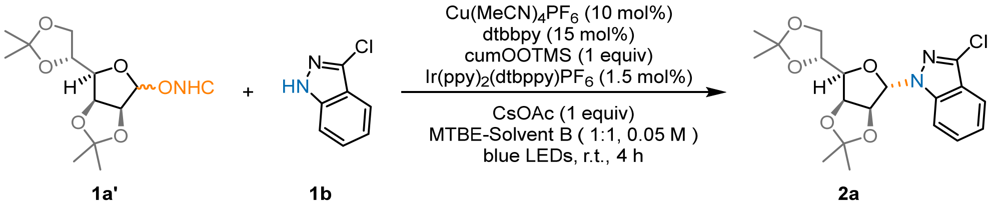

Entry	Solvent B	Yield (%)
1	MeCN	43
2	MTBE	23
3	DCM	<10
4	1,4-Dioxane	20
5	Acetone	<10
6	THF	trace
7	DMF	7
8	DMSO	22
9	EtOAc	40
10	$PhCF_3$	26

Supplementary Table 4 | Evaluation of PC source

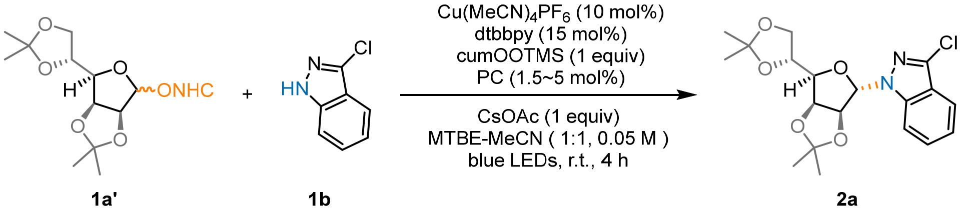

Entry	PC source	Yield (%)
1	4CzIPN (5 mol%)	43
2	PTH1 (5 mol%)	trace

3	PTH2 (5 mol%)	trace
4	PTH4 (5 mol%)	13
5	PTH6 (5 mol%)	13
6	----	16
7	EoSinY (5 mol%)	15
8	Acridine (5 mol%)	14
9	$Ru(bpy)_3(PF_6)_2$ (1.5 mol%)	28
10	*fac*-$Ir(ppy)_3$ (1.5 mol%)	29
11	$Ir[(ppy)_2dtbppy]PF_6$ (1.5 mol%)	42
12	$Ir[dF(CF_3)ppy]_2(dtbppy)PF_6$ (1.5 mol%)	42

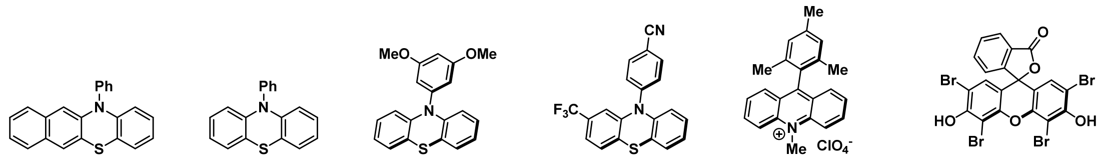

PTH1 PTH2 PTH4 PTH6 Acridine EoSinY

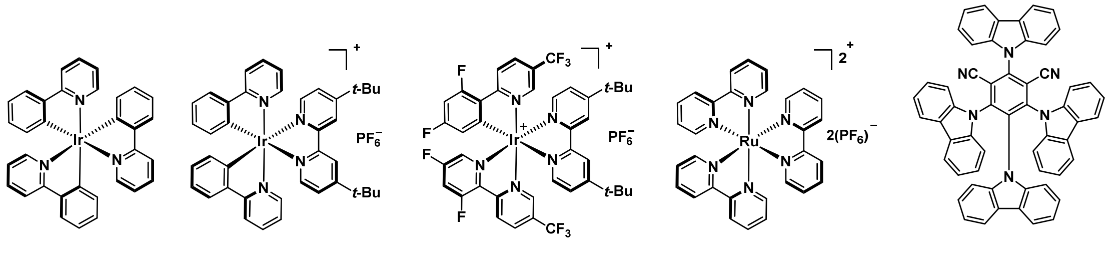

fac-$Ir(ppy)_3$ $Ir(ppy)_2(dtbppy)PF_6$ $Ir(dFCF_3ppy)_2(dtbppy)PF_6$ $Ru(ppy)_3(PF_6)_2$ 4CzIPN

Supplementary Table 5 | Evaluation of oxidants

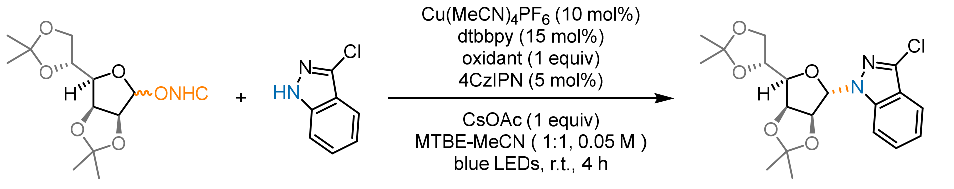

1a' 1b 2a

Entry	Oxidant	Yield (%)
1	*t*-BuOOH (70% wt in H_2O)	45
2	cumOOH	41
3	(*t*-BuO)$_2$	-
4	BzOO*t*Bu	31
5	BPO	-
6	LPO	12

7	$Na_2S_2O_8$	-
8	DDQ	-
9	cumOOTMS	43
10	O_2	-
11	$PhI(OAc)_2$	5
12	$NaNO_2$	-

Supplementary Table 6 | Evaluation of ligands

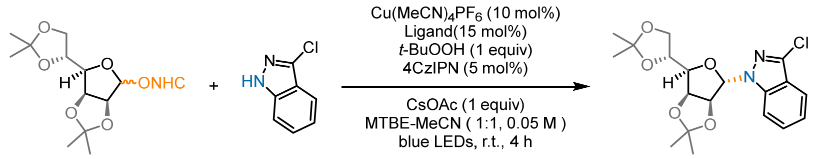

1a' **1b** **2a**

ligands:

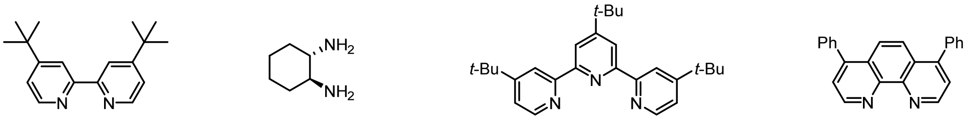

L1, >20:1, 45% **L2,** 6:1, 15% **L3,** >20:1,28% **L4,** >20:1,24%

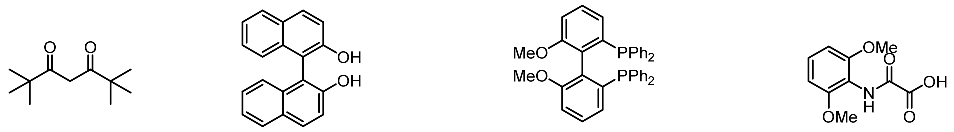

L5, 4:1, 37% **L6,** trace **L7,** trace **L8,** 4:1, 30%

Entry	Ligand source	Yield (%)	Ratio
1	-----	24	14:1
2	L1	45	>20:1
3	L2	15	6:1
4	L3	28	>20:1
5	L4	24	>20:1
6	L5	37	4:1
7	L6	trace	---
8	L7	trace	---
9	L8	30	4:1

Supplementary Table 7 | Evaluation of solvent A

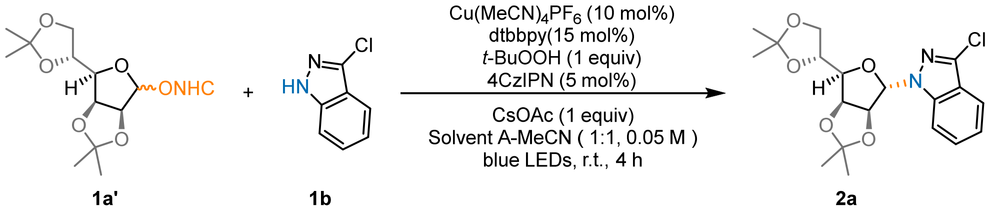

Entry	Solvent A	Yield (%)
1	*t*-BuOMe	45
2	PhCl	54
3	$PhCF_3$	43
4	ethyl acetate	42
5	PhF	42
6	$(i\text{-Pr})_2O$	43
7	toluene	55
8	MeCN	n.r.

Supplementary Table 8 | Evaluation of the oxidant loading

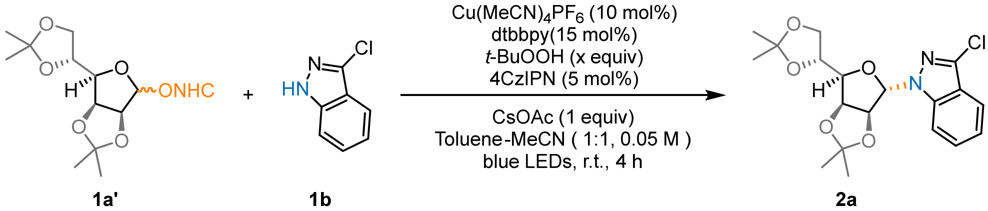

Entry	Oxidant loading (*t*-BuOOH)	Yield (%)
1	------	trace
2	1 equiv (70% wt in H_2O)	55
3	1.5 equiv (70% wt in H_2O)	62
4	2 equiv (70% wt in H_2O)	76
5	2.5 equiv (70% wt in H_2O)	75
6	2 equiv (5 M in decane)	87

Supplementary Table 9 | Evaluation of the loading of NHC-adduct (**1a’**)

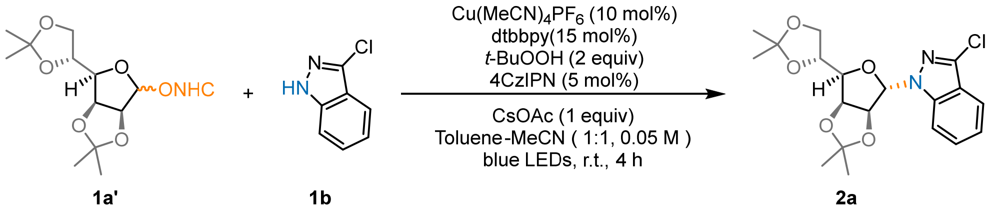

Entry	1a’	Yield (%)
1	1.2 equiv	65
2	1.8 equiv	87
3	2.4 equiv	83
4	3.0 equiv	70

Supplementary Table 10 | Evaluation of the loading of copper catalyst

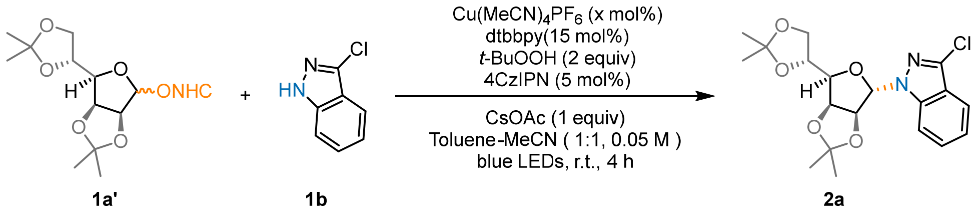

Entry	Copper loading	Yield (%)*
1	20 mol%	85
2	15 mol%	86
3	10 mol%	87
4	5 mol%	70
5	1 mol%	12

Supplementary Table 11 | Evaluation of the temperature

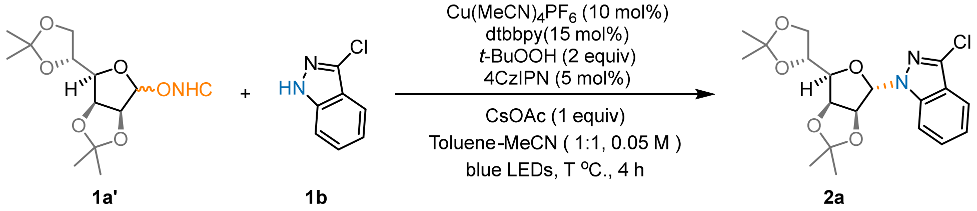

Entry	T °C	Yield (%)*
1	0 °C	89
2	rt	87
3	50 °C	76

Supplementary Table 12 | Evaluation of the light source

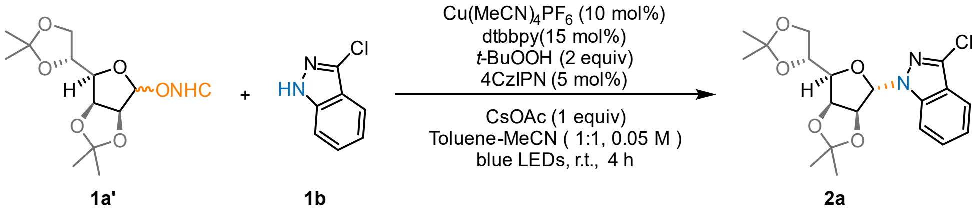

Entry	Deviations from standard	Yield (%)	N1:N2
1	12 W blue light	87	>20:1
2	12 W white light	85	9:1
3	Sun light	77	9:1

The reaction proceeded smoothly under both white light and sunlight, which can be attributed to the wide absorption range of the photocatalyst (250-470 nm). However, blue light showed better results.

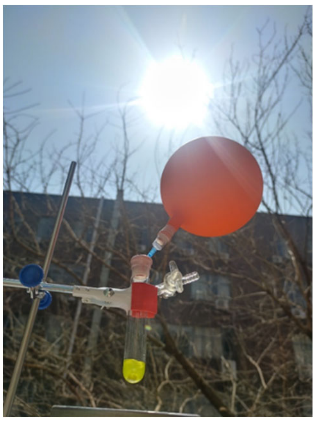

Supplementary Figure 2 | Reaction setup in sunlight conditions

Supplementary Table 13 | Varation from optimal conditions

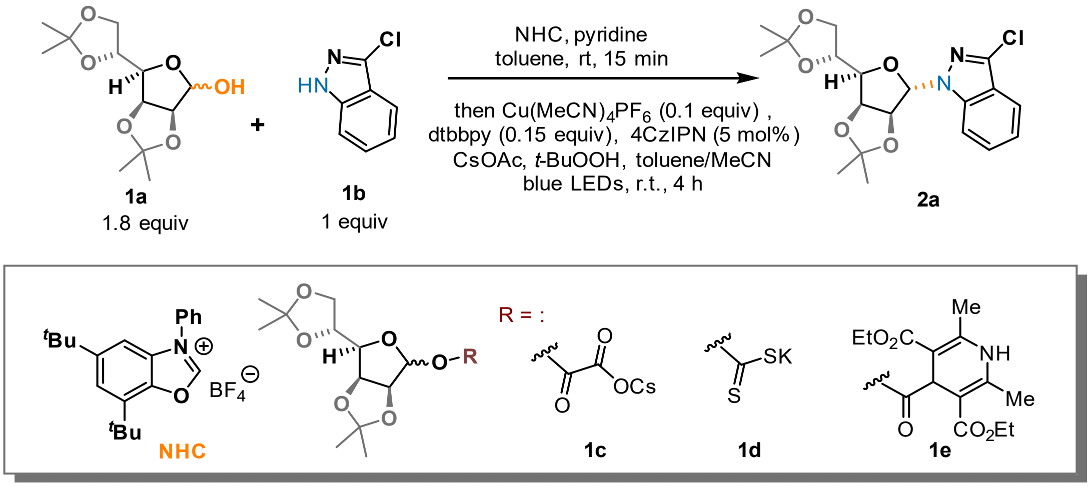

entry	Varation from optimal conditions	Yield(%)[c]
1	------	87%
	No [Cu]	N.D.
2	No base	N.D.
3	No PC	trace
4	No [O]	N.D.
5	No light	trace
6	No ligand	71 (N1:N2=9:1)
7	Cu(TMHD)$_2$ as catalyst	66 (N1:N2 = 3:2)
8	Bphen as ligand	75
9	BTMG as base	57
10	Ir[dF(CF$_3$)ppy]$_2$(dtbppy)PF$_6$ (1.5 mol%)	84
11	cumOOTMS as oxidant	81
12	DMSO as solvent	55
13	under air	86
14	10 equiv H$_2$O	78
15	**1c** instead of **1a**+NHC	N.D.
16	**1d** instead of **1a**+NHC	N.D.
17	**1e** instead of **1a**+NHC	trace

Supplementary Table 14 | The regioselectivity of 3-phenyl pyrozole

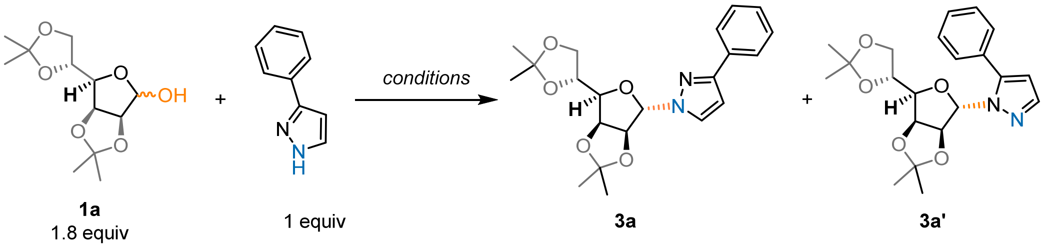

Entry	Conditions	Yield (%)	N1 : N2 (**3a**:**3a'**)
1	standard conditions	74	2.1:1
2	TMG as base	78	2.4:1
3	Cu(TMHD)$_2$ as cat.	54	1.3:1
4	CH$_3$COOCH(CH$_3$)$_2$ as solvent	74	5.1:1
5	Bphen as Ligand	55	>20:1
6	10 mol% CuTc + 10 mol% Bphen as cat.	71	>20:1

The regioselectivity of the regioisomers was determined from the C_3-H signals observed in the spectra shown below.

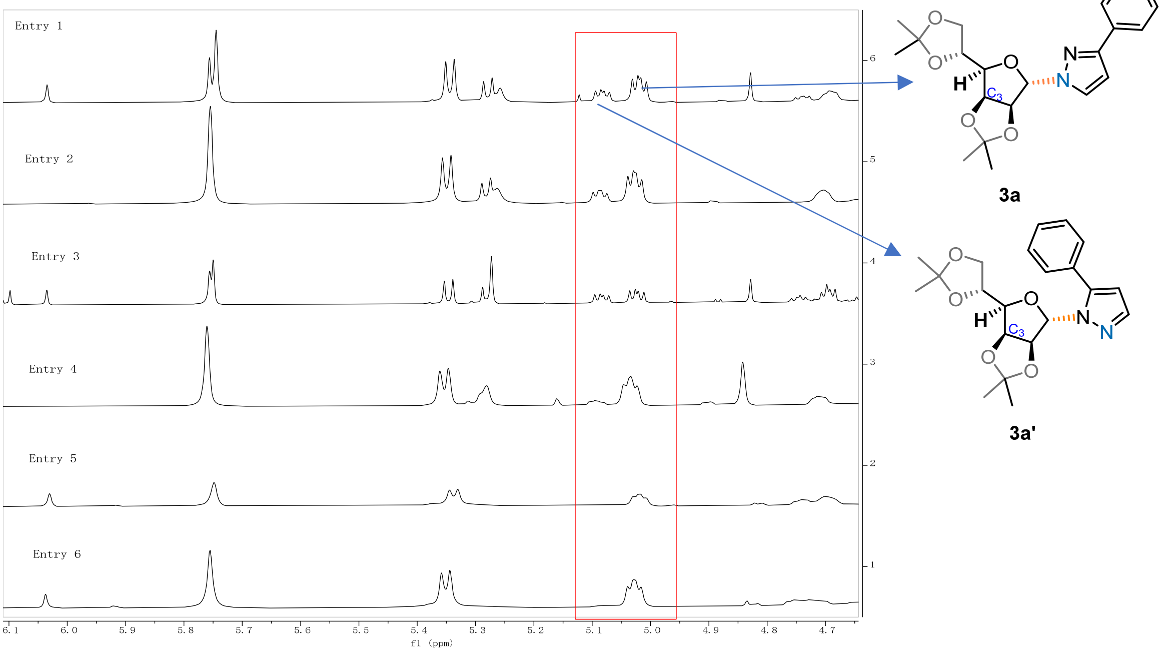

Supplementary Figure 3 | Determination of the regioselectivity of 3a/3a'

2.2 General procedures for N-glycosylation of heterocycles

Procedure A:

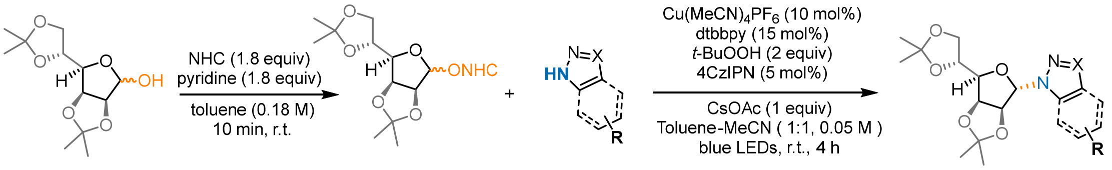

An oven-dried 10 mL Schlenk tube was charged with 1-hydroxylmannose **1a** (93.6 mg, 0.36 mmol, 1.8 equiv), NHC (142.4 mg, 0.36 mmol, 1.8 equiv) and a magnetic stir bar. After the Schlenk tube was vacuumed and refilled with nitrogen gas three times, dry toluene (2.0 mL) was added and the reaction was stirred at r.t. for 5 min. Then, pyridine (29.1 μL, 0.36 mmol, 1.8 equiv) was added dropwise at room temperature. The resulting solution was stirred at r.t. for 10 min. A white solid precipitated out during this time. [1-3]

Another 10 mL Schlenk tube was charged with 1,2,3,5-Tetrakis(carbazol-9-yl)-4,6-dicyanobenzene (4CzIPN, 5 mg, 0.01 mmol, 0.05 equiv), $Cu(MeCN)_4PF_6$ (7.4 mg, 0.02 mmol, 0.1 equiv), dtbbpy (4,4-di-*tert*-butyl bipyridine, 8.0 mg, 0.03 mmol, 0.15 equiv), CsOAc (38.2 mg, 0.2 mmol, 1.0 equiv), *N*-heterocycle (0.2 mmol, 1.0 equiv) and a magnetic stir bar. This Schlenk tube was vacuumed and refilled with nitrogen gas three times. Dry acetonitrile (2.0 mL) was added to this Schlenk tube under an atmosphere of nitrogen and stirred at room

temperature.

The toluene suspension was transferred to a 5 mL syringe under an atmosphere of nitrogen. Then a syringe filter and new needle were installed on the syringe, and the toluene solution was injected through the syringe filter into the MeCN solution. *t*-BuOOH (80 µL, 5-6 M in decane, 2.0 equiv) was added, before subjecting the reaction mixture to irradiation by 420 nm blue LEDs at room temperature for a duration of 4 hours. The organic layers were evaporated and then purified by flash column chromatography on silica gel.

Procedure B:

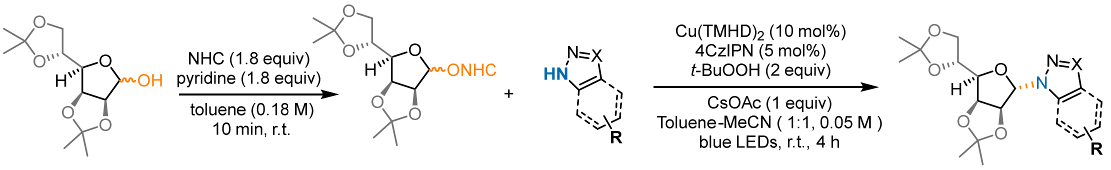

An oven-dried 10 mL Schlenk tube was charged with 1-hydroxylmannose **1a** (93.6 mg, 0.36 mmol, 1.8 equiv), NHC (142.4 mg, 0.36 mmol, 1.8 equiv) and a magnetic stir bar. After the Schlenk tube was vacuumed and refilled with nitrogen gas three times, dry toluene (2.0 mL) was added and the reaction was stirred at r.t. for 5 min. Then, pyridine (29.1 µL, 0.36 mmol, 1.8 equiv) was added dropwise at room temperature. The resulting solution was stirred at r.t. for 10 min. A white solid precipitated out during this time. [1-3]

Another 10 mL Schlenk tube was charged with 4CzIPN (7.5 mg, 0.01 mmol, 0.05 equiv), 2,2,6,6-Tetramethyl-3,5-heptanedione copper (II) ($Cu(TMHD)_2$, 8.6 mg, 0.02 mmol, 0.1 equiv), CsOAc (38.2 mg, 0.2 mmol, 1.0 equiv), *N*-heterocycle (0.2 mmol, 1.0 equiv) and a magnetic stir bar. And this Schlenk tube was vacuumed and refilled with nitrogen gas three times. Dry MeCN (2.0 mL) was added to this Schlenk tube under an atmosphere of nitrogen and stirred at r.t.

The toluene suspension was transferred to a 5 mL syringe under an atmosphere of nitrogen. Then a syringe filter and new needle were installed on the syringe, and the toluene solution was injected through the syringe filter into the MeCN solution. *t*-BuOOH (80 µL, 5-6 M in decane, 2.0 equiv) was added, before subjecting the reaction mixture to irradiation by 420 nm blue LEDs at room temperature for a duration of 4 hours. The organic layers were evaporated and then purified by flash column chromatography on silica gel.

Procedure C:

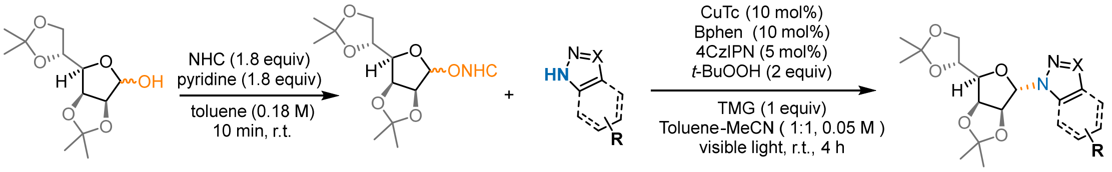

An oven-dried 10 mL Schlenk tube was charged with 1-hydroxylmannose 1a (93.6 mg, 0.36 mmol, 1.8 equiv), NHC (142.4 mg, 0.36 mmol, 1.8 equiv) and a magnetic stir bar. After the Schlenk tube was vacuumed and refilled with nitrogen gas three times, dry toluene (2.0 mL) was added and the reaction was stirred at r.t. for 5 min. Then, pyridine (29.1 μL, 0.36 mmol, 1.8 equiv) was added dropwise at room temperature. The resulting solution was stirred at r.t. for 10 min. A white solid precipitated out during this time. [1-3]

Another 10 mL Schlenk tube was charged with 4CzIPN (7.5 mg, 0.01 mmol, 0.05 equiv), copper(I) thiophene-2-carboxylate (CuTc, 3.8 mg, 0.02 mmol, 0.1 equiv), 4,7-diphenyl-1,10-phenanthroline (Bphen, 10.0 mg, 0.03 mmol, 0.15 equiv), 1,1,3,3-Tetramethylguanidine (TMG, 25.1 μL, 0.2 mmol, 1.0 equiv), *N*-heterocycle (0.2 mmol, 1.0 equiv) and a magnetic stir bar. This Schlenk tube was vacuumed and refilled with nitrogen gas three times. Dry acetonitrile (2.0 mL) was added to this Schlenk tube under an atmosphere of nitrogen and stirred at r.t..

The toluene suspension was transferred to a 5 mL syringe under an atmosphere of nitrogen. Then a syringe filter and new needle were installed on the syringe, and the toluene solution was injected through the syringe filter into the MeCN solution. *t*-BuOOH (80 μL, 5-6 M in decane, 2.0 equiv) was added, before subjecting the reaction mixture to irradiation by 420 nm blue LEDs at room temperature for a duration of 4 hours. The organic layers were evaporated and then purified by flash column chromatography on silica gel.

Supplementary Table 15 | Synthesis method for substrate

Substrate	Synthesis method
**2a-2k, 3d, 3f, 3g, 8l**	according to procedure A
**3e, 4f, 4g, 5a-5n, 9f**	according to procedure B
**3h, 3i, 4i, 6b-6g, 7b-7d, 9b, 9c, 9e**	according to procedure C
**4a-4e, 4h, 9a**	according to procedure B, but base CsOAc replaced with TMG
**3a, 3b, 3c**	according to procedure C, but base replaced TMG with CsOAc
**6a**	according to procedure C, but solvent MeCN replaced with DMSO
**7a**	according to procedure A, but base CsOAc replaced with TMG
**8a-8k, 8m-8p**	according to procedure A, but reaction irradiated at 0 °C, base CsOAc replaced with TMG
**9d**	according to procedure A, but solvent MeCN replaced with DMSO

3-Chloro-1-((3a*S*,4*S*,6*R*,6a*S*)-6-((*R*)-2,2-dimethyl-1,3-dioxolan-4-yl)-2,2-dimethyltetrahydrofuro[3,4-*d*][1,3]dioxol-4-yl)-1*H*-indazole (2a)

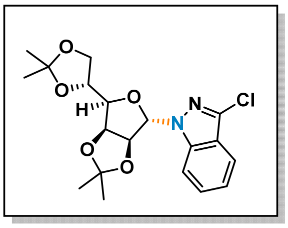

Purified by flash column chromatography (ethyl acetate/ petroleum ether = 1:33, v:v); white solid (67 mg, 85%), mp: 90-95 ºC; $[\alpha]_D^{25}$ = +90.6 (*c* = 0.4, $CHCl_3$); **^{1}H NMR** (400 MHz, $CDCl_3$) δ 7.68 (d, *J* = 8.2 Hz, 1H), 7.54–7.44 (m, 2H), 7.30–7.22 (m, 1H), 6.20 (s, 1H), 5.55 (d, *J* = 5.9 Hz, 1H), 5.16 (dd, *J* = 5.9, 3.8 Hz, 1H), 4.45–4.38 (m, 1H), 4.10 (dd, *J* = 7.3, 3.8 Hz, 1H), 4.04 (dd, *J* = 8.7, 6.3 Hz, 1H), 3.89 (dd, *J* = 8.7, 4.7 Hz, 1H), 1.59 (s, 3H), 1.42 (s, 3H), 1.40 (s, 3H), 1.35 (s, 3H); **^{13}C NMR** (101 MHz, $CDCl_3$) δ 141.3, 135.4, 128.5, 122.4, 121.86, 120.0, 113.0, 109.9, 109.3, 90.0, 84.6, 82.7, 80.6, 73.4, 66.7, 26.9, 26.1, 25.2, 24.5; **HRMS (ESI)** m/z cald for $C_{19}H_{24}ClN_2O_5^+$ $[M+H]^+$ 395.1368, found 395.1346.

1-((3a*S*,4*S*,6*R*,6a*S*)-6-((*R*)-2,2-dimethyl-1,3-dioxolan-4-yl)-2,2-dimethyltetrahydrofuro[3,4-*d*][1,3]dioxol-4-yl)-1*H*-indazole (2b)

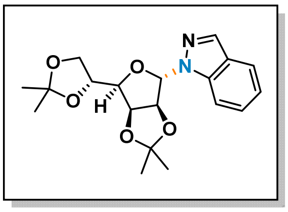

Purified by flash column chromatography (ethyl acetate/petroleum ether = 1:28, v:v); white solid (60 mg, 83%), mp: 96-98 ºC; $[\alpha]_D^{25}$ = +78.3 (*c* = 0.8, $CHCl_3$); **^{1}H NMR** (400 MHz, $CDCl_3$) δ 7.99 (s, 1H), 7.74 (d, *J* = 8.1 Hz, 1H), 7.54 (d, *J* = 8.4 Hz, 1H), 7.46–7.37 (m, 1H), 7.24–7.16 (m, 1H), 6.27 (s, 1H), 5.59 (d, *J* = 5.9 Hz, 1H), 5.17 (dd, *J* = 5.9, 3.9 Hz, 1H), 4.46–4.37 (m, 1H), 4.10–3.99 (m, 2H), 3.87 (dd, *J* = 8.7, 4.4 Hz, 1H), 1.60 (s, 3H), 1.43 (s, 3H), 1.39 (s, 3H), 1.35 (s, 3H); **^{13}C NMR** (101 MHz, $CDCl_3$) δ 140.1, 134.9, 127.2, 124.5, 121.7, 121.2, 112.9, 109.4, 109.3, 89.7, 84.7, 82.6, 80.7, 73.4, 66.8, 27.0, 26.1, 25.3, 24.5; **HRMS (ESI)** m/z cald for $C_{19}H_{25}N_2O_5^+$ $[M+H]^+$ 361.1758, found 361.1747.

1-(1-((3a*S*,4*S*,6*R*,6a*S*)-6-((*R*)-2,2-dimethyl-1,3-dioxolan-4-yl)-2,2-dimethyltetrahydrofuro[3,4-*d*][1,3]dioxol-4-yl)-1*H*-indazol-3-yl)ethan-1-one (2c)

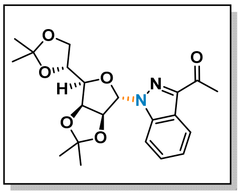

Purified by flash column chromatography (acetone / petroleum ether = 1:24, v:v); Colorless oil (67 mg, 83%); $[\alpha]_D^{25}$ = -76.2 (*c* = 3.0, $CHCl_3$); **^{1}H NMR** (400 MHz, $CDCl_3$) δ 8.37-8.33 (m, 1H), 7.60–7.52 (m, 1H), 7.50–7.42 (m, 1H), 7.37–7.31 (m, 1H), 6.31 (s, 1H), 5.60 (d, *J* = 5.8 Hz, 1H), 5.17 (dd, *J* = 5.9, 3.8 Hz, 1H), 4.45 (ddd, *J* = 7.4, 6.3, 4.5 Hz, 1H), 4.12 (dd, *J* = 7.3, 3.8 Hz, 1H), 4.04 (dd, *J* = 8.7, 6.3 Hz, 1H), 3.90 (dd, *J* = 8.7, 4.5 Hz, 1H), 2.67 (s, 3H), 1.60 (s, 3H), 1.44 (s, 3H), 1.38 (s, 3H), 1.34 (s, 3H); **^{13}C NMR** (101 MHz, $CDCl_3$) δ 194.7, 143.6, 141.2,

127.8, 124.3, 123.1, 122.7, 113.3, 109.6, 109.3, 90.3, 84.6, 83.0, 80.6, 73.2, 66.6, 26.9, 26.8, 26.1, 25.2, 24.6; **HRMS (ESI)** m/z cald for $NaC_{21}H_{26}N_2O_6^+$ $[M+Na]^+$ 425.1683, found 425.1676.

Methyl1-((3a*S*,4*S*,6*R*,6a*S*)-6-((*R*)-2,2-dimethyl-1,3-dioxolan-4-yl)-2,2-dimethyltetrahydrofuro[3,4-*d*][1,3]dioxol-4-yl)-1*H*-indazole-3-carboxylate (2d)

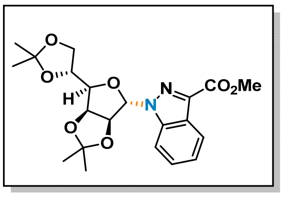

Purified by flash column chromatography (acetone/petroleum ether = 1:20, v:v); Colorless oil (54 mg, 64%); $[\alpha]_D^{25}$ = +59.2 (*c* = 1.2, $CHCl_3$); **^{1}H NMR** (400 MHz, $CDCl_3$) δ 8.23-8.17 (m, 1H), 7.63-7.58 (m, 1H), 7.49-7.45 (m, 1H), 7.38-7.32 (m, 1H), 6.31 (s, 1H), 5.72 (d, *J* = 5.8 Hz, 1H), 5.20 (dd, *J* = 5.9, 3.7 Hz, 1H), 4.43 (ddd, *J* = 7.2, 6.4, 4.7 Hz, 1H), 4.07–4.02 (m, 5H), 3.87 (dd, *J* = 8.7, 4.7 Hz, 1H), 1.59 (s, 3H), 1.42 (s, 3H), 1.37 (s, 3H), 1.34 (s, 3H); **^{13}C NMR** (101 MHz, $CDCl_3$) δ 163.0, 141.1, 136.6, 127.7, 123.9, 123.9, 122.5, 113.1, 110.1, 109.3, 90.6, 84.3, 82.8, 80.5, 73.2, 66.7, 52.2, 26.9, 26.1, 25.2, 24.6; **HRMS (ESI)** m/z cald for $C_{21}H_{27}N_2O_7^+$ $[M+H]^+$ 419.1813, found 419.1805.

1-((3a*S*,4*S*,6*R*,6a*S*)-6-((*R*)-2,2-dimethyl-1,3-dioxolan-4-yl)-2,2-dimethyltetrahydrofuro[3,4-*d*][1,3]dioxol-4-yl)-1*H*-indazole-3-carbonitrile (2e)

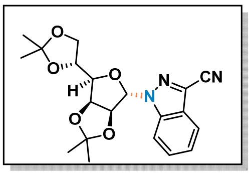

Purified by flash column chromatography (acetone/petroleum ether = 1:17, v:v); white solid (49 mg, 64%), mp: 132-134°C; $[\alpha]_D^{25}$ = +62.1 (*c* = 0.4, $CHCl_3$); **^{1}H NMR** (400 MHz, $CDCl_3$) δ 7.88-7.85 (m, 1H), 7.70–7.62 (m, 1H), 7.60–7.50 (m, 1H), 7.44–7.38 (m, 1H), 6.30 (s, 1H), 5.58 (d, *J* = 5.9 Hz, 1H), 5.16 (dd, *J* = 5.9, 3.8 Hz, 1H), 4.46–4.38 (m, 1H), 4.07–4.02 (m, 2H), 3.89 (dd, *J* = 8.8, 4.5 Hz, 1H), 1.60 (s, 3H), 1.43 (s, 3H), 1.40 (s, 3H), 1.35 (s, 3H); **^{13}C NMR** (101 MHz, $CDCl_3$) δ 140.1, 128.8, 125.5, 124.5, 119.9, 119.8, 113.4, 113.3, 110.6, 109.5, 90.6, 84.5, 83.1, 80.4, 73.2, 66.6, 27.0, 26.1, 25.2, 24.5; **HRMS (ESI)** m/z cald for $C_{20}H_{24}N_3O_5^+$ $[M+H]^+$ 386.1710, found 386.1710.

1-((3a*S*,4*S*,6*R*,6a*S*)-6-((*R*)-2,2-dimethyl-1,3-dioxolan-4-yl)-2,2-dimethyltetrahydrofuro[3,4-*d*][1,3]dioxol-4-yl)-4-fluoro-1*H*-indazole (2f)

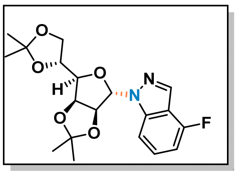

Purified by flash column chromatography (acetone / petroleum ether = 1:27, v:v); Colorless oil (70 mg, 92%); $[\alpha]_D^{25}$ = +103.2 (*c* = 1.4, $CHCl_3$); **^{1}H NMR** (400 MHz, $CDCl_3$) δ 8.05 (s, 1H), 7.38–7.28 (m, 2H), 6.83 (dd,

J = 9.8, 7.2 Hz, 1H), 6.23 (s, 1H), 5.57 (d, J = 5.8 Hz, 1H), 5.16 (dd, J = 5.9, 3.8 Hz, 1H), 4.42 (td, J = 6.9, 4.5 Hz, 1H), 4.13–3.98 (m, 2H), 3.83-3.91 (m, 1H), 1.59 (s, 3H), 1.42 (s, 3H), 1.39 (s, 3H), 1.34 (s, 3H); **^{13}C NMR** (101 MHz, $CDCl_3$) δ 154.8 (d, J = 253.0 Hz), 141.5 (d, J = 8.8 Hz), 130.2, 127.1 (d, J = 7.6 Hz), 113.7 (d, J = 23.2 Hz), 111.9, 108.2, 104.9 (d, J = 18.4 Hz), 104.4 (d, J = 4.2 Hz), 88.9, 83.5, 81.6 , 79.5, 72.2, 65.6, 25.8, 24.9, 24.1, 23.4; **^{19}F NMR** (376 MHz, $CDCl_3$) δ -117.73 (dd, J = 9.8, 4.6 Hz, 1F); **^{19}F{^{1}H} NMR** (376 MHz, $CDCl_3$) δ -117.73 (s, 1F); **HRMS (ESI)** m/z cald for $C_{19}H_{24}FN_2O_5^+$ $[M+H]^+$ 379.1664, found 379.1674.

4-Chloro-1-((3a*S*,4*S*,6*R*,6a*S*)-6-((*R*)-2,2-dimethyl-1,3-dioxolan-4-yl)-2,2-dimethyltetrahydrofuro[3,4-*d*][1,3]dioxol-4-yl)-1*H*-indazole (2g)

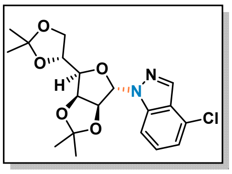

Purified by flash column chromatography (acetone/petroleum ether = 1:35, v:v); Colorless oil (59 mg, 76%); $[\alpha]_D^{25}$ = +84.3 (c = 2.0, $CHCl_3$); **^{1}H NMR** (400 MHz, $CDCl_3$) δ 8.06 (s, 1H), 7.43 (d, J = 8.3 Hz, 1H), 7.32 (dd, J = 8.4, 7.4 Hz, 1H), 7.17 (d, J = 7.4 Hz, 1H), 6.22 (s, 1H), 5.58 (d, J = 5.9 Hz, 1H), 5.16 (dd, J = 5.9, 3.8 Hz, 1H), 4.41 (ddd, J = 7.5, 6.3, 4.5 Hz, 1H), 4.11–3.97 (m, 2H), 3.86 (dd, J = 8.7, 4.5 Hz, 1H), 1.59 (s, 3H), 1.42 (s, 3H), 1.39 (s, 3H), 1.34 (s, 3H); **^{13}C NMR** (101 MHz, $CDCl_3$) δ 141.0, 133.5, 127.8, 126.9, 123.8, 121.3, 113.0, 109.3, 108.1, 90.0, 84.6, 82.8, 80.7, 73.3, 66.8, 26.9, 26.1, 25.3, 24.5; **HRMS (ESI)** m/z cald for $C_{19}H_{24}ClN_2O_5^+$ $[M+H]^+$ 395.1368, found 395.1362.

1-((3a*S*,4*S*,6*R*,6a*S*)-6-((*R*)-2,2-dimethyl-1,3-dioxolan-4-yl)-2,2-dimethyltetrahydrofuro[3,4-*d*][1,3]dioxol-4-yl)-6-methoxy-1H-indazole (2h)

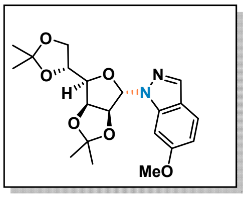

Purified by flash column chromatography (ethyl acetate/petroleum ether = 1:25, v:v); white solid (54 mg, 67%), mp: 140-141 ºC; $[\alpha]_D^{25}$ = +101.4 (c = 2.7, $CHCl_3$); **^{1}H NMR** (400 MHz, $CDCl_3$) δ 7.86 (s, 1H), 7.56 (d, J = 8.8 Hz, 1H), 6.87 (d, J = 2.0 Hz, 1H), 6.84 (dd, J = 8.8, 2.1 Hz, 1H), 6.19 (s, 1H), 5.58 (d, J = 5.9 Hz, 1H), 5.16 (dd, J = 5.9, 3.8 Hz, 1H), 4.42 (ddd, J = 7.6, 6.3, 4.4 Hz, 1H), 4.08 (dd, J = 7.6, 3.9 Hz, 1H), 4.04 (dd, J = 8.7, 6.3 Hz, 1H), 3.89 (dd, J = 8.7, 4.4 Hz, 1H), 3.86 (s, 3H), 1.59 (s, 3H), 1.42 (s, 3H), 1.39 (s, 3H), 1.34 (s, 3H); **^{13}C NMR** (100 MHz, $CDCl_3$) δ 159.9, 141.3, 134.7, 121.8, 118.9, 113.8, 112.7, 109.2, 90.5, 89.5, 84.5, 82.4, 80.6, 73.3, 66.7, 55.5, 26.9, 26.0, 25.1, 24.4; **HRMS (ESI)** m/z cald for $C_{20}H_{27}N_2O_6^+$ $[M+H]^+$ 391.1864, found 391.1854

6-Bromo-1-((3a*S*,4*S*,6*R*,6a*S*)-6-((*R*)-2,2-dimethyl-1,3-dioxolan-4-yl)-2,2-dimethyltetrahydrofuro[3,4-*d*][1,3]dioxol-4-yl)-1*H*-indazole (2i)

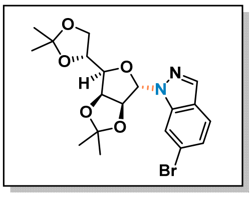

Purified by flash column chromatography (ethyl acetate/petroleum ether = 1:25, v:v); white solid (57 mg, 65%), mp: 140-143 °C; $[\alpha]_D^{25}$ = +139.0 (*c* = 2.5, $CHCl_3$); **^{1}H NMR** (400 MHz, $CDCl_3$) δ 7.94 (s, 1H), 7.73 (s, 1H), 7.57 (d, *J* = 8.5 Hz, 1H), 7.29 (dd, *J* = 8.5, 1.6 Hz, 1H), 6.17 (s, 1H), 5.57 (d, *J* = 5.9 Hz, 1H), 5.13 (dd, *J* = 5.9, 3.8 Hz, 1H), 4.41 (ddd, *J* = 7.6, 6.3, 4.5 Hz, 1H), 4.08–4.01 (m, 2H), 3.88 (dd, *J* = 8.8, 4.5 Hz, 1H), 1.58 (s, 3H), 1.41 (s, 3H), 1.39 (s, 3H), 1.34 (s, 3H); **^{13}C NMR** (101 MHz, $CDCl_3$) δ 140.8, 135.0, 125.3, 123.3, 122.3, 121.7, 113.0, 112.6, 109.3, 89.9, 84.5, 82.7, 80.6, 73.3, 66.7, 26.9, 26.1, 25.2, 24.5; **HRMS (ESI)** m/z cald for $C_{19}H_{24}BrN_2O_5$ $[M+H]^+$ 439.0863, found 439.0872.

1-((3a*S*,4*S*,6*R*,6a*S*)-6-((*R*)-2,2-dimethyl-1,3-dioxolan-4-yl)-2,2-dimethyltetrahydrofuro[3,4-*d*][1,3]dioxol-4-yl)-1*H*-pyrazolo[3,4-*b*]pyridine (2j)

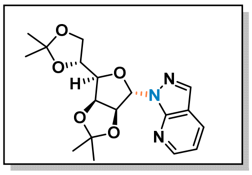

Purified by flash column chromatography (ethyl acetate/petroleum ether = 1:12, v:v); white solid (44 mg, 61%), mp: 107-109 °C; $[\alpha]_D^{25}$ = +77.8 (*c* = 2.1, $CHCl_3$); **^{1}H NMR** (400 MHz, DMSO-*d6*) δ 8.60 (dd, *J* = 4.5, 1.5 Hz, 1H), 8.30 (d, *J* = 8.0 Hz, 2H), 7.30 (dd, *J* = 8.0, 4.5 Hz, 1H), 6.50 (s, 1H), 5.35 (d, *J* = 5.8 Hz, 1H), 5.14 (dd, *J* = 5.9, 3.3 Hz, 1H), 4.36–4.26 (m, 2H), 3.95 (dd, *J* = 8.4, 5.8 Hz, 1H), 3.73 (dd, *J* = 8.5, 5.0 Hz, 1H), 1.49 (s, 3H), 1.33 (s, 3H), 1.26 (s, 4H), 1.23 (s, 3H); **^{13}C NMR** (101 MHz, DMSO-*d6*) δ 150.9, 150.4, 135.4, 132.1, 119.0, 116.4, 113.2, 108.9, 89.1, 85.4, 83.6, 81.3, 73.7, 66.6, 27.5, 26.9, 26.0, 25.3; **HRMS (ESI)** m/z cald for $C_{18}H_{24}N_3O_5^+$ $[M+H]^+$ 362.1710, found 362.1699.

1-((3a*S*,4*S*,6*R*,6a*S*)-6-((*R*)-2,2-dimethyl-1,3-dioxolan-4-yl)-2,2-dimethyltetrahydrofuro[3,4-*d*][1,3]dioxol-4-yl)-1*H*-pyrazolo[3,4-*d*]pyrimidine (2k)

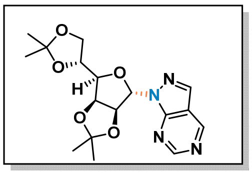

Purified by flash column chromatography (ethyl acetate/ petroleum ether = 2:1, v:v); Colorless oil (38 mg, 52%); $[\alpha]_D^{25}$ = +71.8 (*c* = 1.9, $CHCl_3$); **^{1}H NMR** (400 MHz, $CDCl_3$) δ 9.21 (s, 1H), 9.04 (s, 1H), 8.15 (s, 1H), 6.61 (s, 1H), 5.40 (d, *J* = 5.9 Hz, 1H), 5.20 (dd, *J* = 5.9, 3.8 Hz, 1H), 4.42 (ddd, *J* = 7.7, 6.2, 4.3 Hz, 1H), 4.26 (dd, *J* = 7.7, 3.8 Hz, 1H), 4.03 (dd, *J* = 8.8, 6.2 Hz, 1H), 3.90 (dd, *J* = 8.8, 4.3 Hz, 1H), 1.57 (s, 3H), 1.40 (s, 6H), 1.34 (s, 3H); **^{13}C NMR** (101 MHz, $CDCl_3$) δ 155.7, 152.9, 152.6, 133.9, 114.7, 113.4, 109.3, 89.0, 84.9, 83.4, 80.9, 73.4, 66.8, 27.0, 26.2, 25.3, 24.71; **HRMS (ESI)** m/z cald for $C_{17}H_{23}N_4O_5^+$ $[M+H]^+$ 363.1663, found

363.1655.

1-((3a*S*,4*S*,6*R*,6a*S*)-6-((*R*)-2,2-dimethyl-1,3-dioxolan-4-yl)-2,2-dimethyltetrahydrofuro[3,4-*d*][1,3]dioxol-4-yl)-3-phenyl-1*H*-pyrazole (3a)

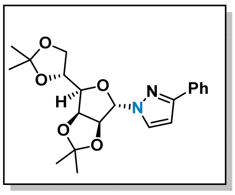

Purified by flash column chromatography (ethyl acetate/petroleum ether = 1:30, v:v); white solid (57 mg, 74%), mp: 77-78 °C; $[\alpha]_D^{25}$ = -50.1 (*c* = 1.4, $CHCl_3$); ^{1}**H NMR** (400 MHz, $CDCl_3$) δ 7.82–7.75 (m, 2H), 7.54 (d, *J* = 2.4 Hz, 1H), 7.44–7.37 (m, 2H), 7.36–7.28 (m, 1H), 6.59 (d, *J* = 2.4 Hz, 1H), 5.85 (s, 1H), 5.46 (d, *J* = 5.9 Hz, 1H), 5.13 (dd, *J* = 5.9, 3.8 Hz, 1H), 4.47–4.40 (m, 1H), 4.35 (dd, *J* = 7.2, 3.9 Hz, 1H), 4.09 (dd, *J* = 8.6, 6.3 Hz, 1H), 4.00 (dd, *J* = 8.6, 4.8 Hz, 1H), 1.56 (s, 3H), 1.43 (s, 3H), 1.40 (s, 3H), 1.37 (s, 3H); ^{13}**C NMR** (101 MHz, $CDCl_3$) δ 152.8, 133.2, 131.1, 128.7, 128.1, 125.9, 113.0, 109.2, 103.7, 92.9, 84.9, 83.1, 80.7, 73.4, 66.7, 26.9, 26.1, 25.3, 24.6; **HRMS (ESI)** m/z cald for $C_{21}H_{27}N_2O_5^+$ $[M+H]^+$ 387.1914, found 387.1907.

3-(4-Chlorophenyl)-1-((3a*S*,4*S*,6*R*,6a*S*)-6-((*R*)-2,2-dimethyl-1,3-dioxolan-4-yl)-2,2-dimethyltetrahydrofuro[3,4-*d*][1,3]dioxol-4-yl)-1*H*-pyrazole (3b)

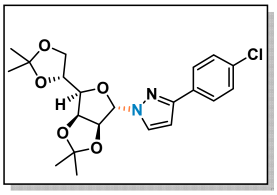

Purified by flash column chromatography (acetone / petroleum ether = 1:40, v:v); white solid (64 mg, 77%), mp: 127-133 °C; $[\alpha]_D^{25}$ = -62.2 (*c* = 0.8, $CHCl_3$); ^{1}**H NMR** (400 MHz, $CDCl_3$) δ 7.74–7.67 (m, 2H), 7.54 (d, *J* = 2.4 Hz, 1H), 7.39–7.33 (m, 2H), 6.56 (d, *J* = 2.4 Hz, 1H), 5.84 (s, 1H), 5.43 (d, *J* = 5.9 Hz, 1H), 5.10 (dd, *J* = 5.9, 3.8 Hz, 1H), 4.47–4.40 (m, 1H), 4.32 (dd, *J* = 7.0, 3.8 Hz, 1H), 4.08 (dd, *J* = 8.6, 6.3 Hz, 1H), 3.99 (dd, *J* = 8.7, 4.8 Hz, 1H), 1.56 (s, 3H), 1.43 (s, 3H), 1.40 (s, 3H), 1.37 (s, 3H); ^{13}**C NMR** (101 MHz, $CDCl_3$) δ 151.7, 133.8, 131.7, 131.3, 128.9, 127.1, 113.1, 109.3, 103.7 (d, *J* = 3.4 Hz), 93.0 (d, *J* = 3.8 Hz), 84.9 (d, *J* = 5.5 Hz), 83.1, 80.7 (d, *J* = 2.1 Hz), 73.4, 66.7, 27.0, 26.1, 25.3, 24.6; **HRMS (ESI)** m/z cald for $C_{21}H_{26}ClN_2O_5^+$ $[M+H]^+$ 421.1525, found 421.1518.

3-(4-bromophenyl)-1-((3a*S*,4*S*,6*R*,6a*S*)-6-((*R*)-2,2-dimethyl-1,3-dioxolan-4-yl)-2,2-dimethyltetrahydrofuro[3,4-*d*][1,3]dioxol-4-yl)-1*H*-pyrazole (3c)

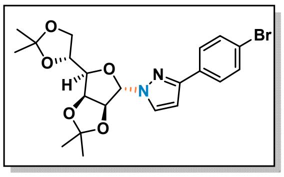

Purified by flash column chromatography (acetone/petroleum ether = 1:40, v:v); colorless oil (79 mg, 85%); $[\alpha]_D^{25}$ = -56.2 (*c* = 1.5, $CHCl_3$); ^{1}**H NMR** (400 MHz, $CDCl_3$) δ 7.57 (d, *J* = 8.6 Hz, 2H), 7.48–7.41 (m, 3H), 6.48 (d, *J* = 2.4 Hz, 1H), 5.77 (s, 1H), 5.35 (d, *J* = 5.9 Hz, 1H), 5.03 (dd, *J* = 5.9, 3.8 Hz, 1H), 4.40–4.31 (m, 1H), 4.24 (dd, *J* = 7.1, 3.8 Hz, 1H), 4.01 (dd,

J = 8.6, 6.3 Hz, 1H), 3.92 (dd, J = 8.7, 4.8 Hz, 1H), 1.48 (s, 3H), 1.35 (s, 3H), 1.32 (s, 3H), 1.29 (s, 3H); **^{13}C NMR** (101 MHz, $CDCl_3$) δ 151.7, 132.2, 131.8, 131.3, 127.4, 122.0, 113.1, 109.2, 103.7 (d, J = 2.6 Hz), 93.0 (d, J = 3.5 Hz), 84.8 (d, J = 3.7 Hz), 83.1, 80.7, 73.4, 66.6, 26.9, 26.1, 25.2, 24.6; **HRMS (ESI)** m/z cald for $C_{21}H_{25}BrN_2O_5Na^+$ $[M+Na]^+$ 487.0839, found 487.0840.

Ethyl 1-((3a*S*,4*S*,6*R*,6a*S*)-6-((*R*)-2,2-dimethyl-1,3-dioxolan-4-yl)-2,2-dimethyltetrahydrofuro[3,4-*d*][1,3]dioxol-4-yl)-1*H*-pyrazole-3-carboxylate (3d)

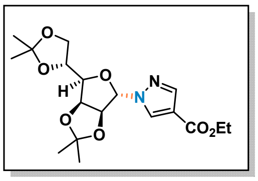

Purified by flash column chromatography (ethyl acetate/ petroleum ether = 1:12, v:v); colorless oil (47 mg, 62%); $[\alpha]_D^{25}$ = -56.2 (c = 1.7, $CHCl_3$); **^{1}H NMR** (400 MHz, $CDCl_3$) δ 8.00 (s, 1H), 7.90 (s, 1H), 5.81 (s, 1H), 5.35 (d, J = 5.9 Hz, 1H), 5.05 (dd, J = 5.9, 3.8 Hz, 1H), 4.43–4.36 (m, 1H), 4.29 (q, J = 7.1 Hz, 2H), 4.21 (dd, J = 7.3, 3.8 Hz, 1H), 4.06 (dd, J = 8.7, 6.3 Hz, 1H), 3.95 (dd, J = 8.7, 4.5 Hz, 1H), 1.53 (s, 3H), 1.42 (s, 3H), 1.37 (s, 3H), 1.36–1.31 (m, 6H); **^{13}C NMR** (101 MHz, $CDCl_3$) δ 162.7, 141.9, 133.0, 116.0, 113.2, 109.3, 93.1, 84.6, 83.2, 80.4, 73.1, 66.6, 60.4, 26.8, 25.9, 25.1, 24.4, 14.4; **HRMS (ESI)** m/z cald for $C_{18}H_{27}N_2O_7^+$ $[M+H]^+$ 383.1813, found 383.1820.

1-((3a*S*,4*S*,6*R*,6a*S*)-6-((*R*)-2,2-dimethyl-1,3-dioxolan-4-yl)-2,2-dimethyltetrahydrofuro[3,4-*d*][1,3]dioxol-4-yl)-5-methyl-3-(trifluoromethyl)-1*H*-pyrazole (3e)

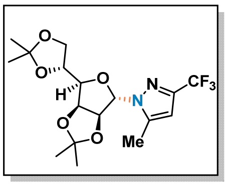

Purified by flash column chromatography (ethyl acetate/ petroleum ether = 1:28, v:v); Colorless oil (61 mg, 77%); $[\alpha]_D^{25}$ = +20.9 (c = 0.8, $CHCl_3$); **^{1}H NMR** (400 MHz, $CDCl_3$) δ 6.30 (s, 1H), 5.86 (s, 1H), 5.46 (d, J = 5.8 Hz, 1H), 5.08 (dd, J = 5.9, 4.0 Hz, 1H), 4.44–4.32 (m, 1H), 4.10–4.02 (m, 2H), 3.94 (dd, J = 8.7, 4.7 Hz, 1H), 2.36 (s, 3H), 1.41 (s, 3H), 1.39 (s, 3H), 1.35 (s, 3H); **^{13}C NMR** (101 MHz, $CDCl_3$) δ 142.4 (q, J = 38.1 Hz), 141.4, 121.3 (q, J = 268.6 Hz), 113.0, 109.3, 104.8, 89.9, 84.3, 82.4, 80.6, 73.3, 66.5, 26.9, 26.0, 25.3, 24.5, 11.0. **^{19}F NMR** (376 MHz, $CDCl_3$) δ -62.46 (s, 3F); **^{19}F {^{1}H} NMR** (376 MHz, $CDCl_3$) δ -62.46 (s, 3F); **HRMS (ESI)** m/z cald for $C_{17}H_{24}F_3N_2O_5^+$ $[M+H]^+$ 393.1632, found 393.1630.

1-((3a*S*,4*S*,6*R*,6a*S*)-6-((*R*)-2,2-dimethyl-1,3-dioxolan-4-yl)-2,2-dimethyltetrahydrofuro[3,4-*d*][1,3]dioxol-4-yl)-3,5-bis(trifluoromethyl)-1*H*-pyrazole (3f)

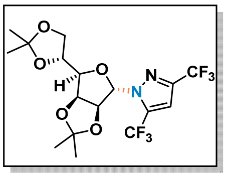

Purified by flash column chromatography (ethyl acetate/ petroleum ether = 1:40, v:v); Colorless oil (52 mg, 58%); $[\alpha]_D^{25}$ = +30.2 (c = 0.7, $CHCl_3$); **^{1}H NMR** (400 MHz, $CDCl_3$) δ 6.94 (s, 1H), 6.06 (s, 1H), 5.42 (d, J = 5.8

Hz, 1H), 5.09 (dd, $J = 5.9, 3.9$ Hz, 1H), 4.45-4.37 (m, 1H), 4.12 (dd, $J = 7.3, 3.9$ Hz, 1H), 4.06 (dd, $J = 8.9, 6.2$ Hz, 1H), 3.95 (dd, $J = 8.8, 4.5$ Hz, 1H), 1.55 (s, 3H), 1.41 (s, 3H), 1.40 (s, 3H), 1.36 (s, 3H); **^{13}C NMR** (101 MHz, $CDCl_3$) δ 142.8 (q, $J = 39.6$ Hz), 134.5 (q, $J = 41.0$ Hz), 120.2 (q, $J = 269.1$ Hz), 119.0 (q, $J = 269.0$ Hz), 113.5, 109.5, 107.1, 92.2, 84.7, 83.5, 80.3, 73.2, 66.5, 26.9, 26.0, 25.4, 24.5; **^{19}F NMR** (376 MHz, $CHCl_3$) δ -59.04 (s, 3F), -62.56 (s, 3F); **^{19}F {^{1}H} NMR** (376 MHz, $CDCl_3$) δ -59.04 (s, 3F), -62.56 (s, 3F); **HRMS (ESI)** m/z cald for $C_{17}H_{21}F_6N_2O_5^+$ $[M+H]^+$ 447.1349, found 447.1332.

Diethyl 1-((3a*S*,4*S*,6*R*,6a*S*)-6-((*R*)-2,2-dimethyl-1,3-dioxolan-4-yl)-2,2-dimethyltetrahydrofuro[3,4-*d*][1,3]dioxol-4-yl)-1*H*-pyrazole-3,5-dicarboxylate (3g)

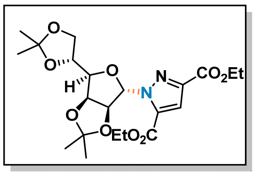

Purified by flash column chromatography (ethyl acetate/ petroleum ether = 1:25, v:v); Colorless oil (62 mg, 69%), $[\alpha]_D^{25} = +26.1$ ($c = 1.2$, $CHCl_3$); **^{1}H NMR** (400 MHz, $CDCl_3$) δ 7.33 (s, 1H), 6.96 (s, 1H), 5.42 (d, $J = 5.9$ Hz, 1H), 5.14 (dd, $J = 5.9, 3.8$ Hz, 1H), 4.47–4.33 (m, 5H), 4.18 (dd, $J = 7.6, 3.8$ Hz, 1H), 4.04 (dd, $J = 8.8, 6.2$ Hz, 1H), 3.91 (dd, $J = 8.7, 4.6$ Hz, 1H), 1.55 (s, 3H), 1.42–1.34 (m, 15H). **^{13}C NMR** (101 MHz, $CDCl_3$) δ 161.4, 158.8, 143.5, 134.6, 114.5, 113.1, 109.3, 91.9, 84.8, 83.1, 80.8, 73.3, 66.8, 61.9, 61.4, 27.0, 26.1, 25.3, 24.7, 14.4, 14.3; **HRMS (ESI)** m/z cald for $NaC_{21}H_{30}N_2O_9^+$ $[M+Na]^+$ 477.1844, found 477.1835.

Methyl 1-((3a*S*,4*S*,6*R*,6a*S*)-6-((*R*)-2,2-dimethyl-1,3-dioxolan-4-yl)-2,2-dimethyltetrahydrofuro[3,4-*d*][1,3]dioxol-4-yl)-1*H*-1,2,4-triazole-3-carboxylate (3h)

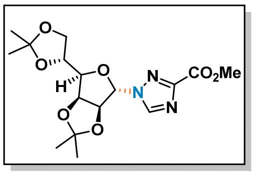

Purified by flash column chromatography (ethyl acetate/petroleum ether = 1:4, v:v); white solid (44 mg, 72%), mp: 124-125 ºC; $[\alpha]_D^{25} = +66.2$ ($c = 1.2$, $CHCl_3$); **^{1}H NMR** (400 MHz, $CDCl_3$) δ 7.96 (s, 1H), 6.92 (s, 1H), 5.22 (d, $J = 5.9$ Hz, 1H), 5.08 (dd, $J = 5.9, 3.8$ Hz, 1H), 4.39 (ddd, $J = 7.8, 6.2, 4.3$ Hz, 1H), 4.24 (dd, $J = 7.7, 3.8$ Hz, 1H), 4.04 (dd, $J = 8.8, 6.2$ Hz, 1H), 4.01 (s, 3H), 3.90 (dd, $J = 8.8, 4.4$ Hz, 1H), 1.54 (s, 3H), 1.41 (s, 3H), 1.37 (s, 3H), 1.34 (s, 3H); **^{13}C NMR** (101 MHz, $CDCl_3$) δ 158.0, 151.2, 144.8, 113.4, 109.4, 91.3, 85.2, 83.6, 80.6, 73.2, 66.8, 53.5, 27.0, 26.1, 25.3, 24.6; **HRMS (ESI)** m/z cald for $C_{16}H_{24}N_3O_7^+$ $[M+H]^+$ 370.1609, found 370.1622.

2-((3a*S*,4*S*,6*R*,6a*S*)-6-((*R*)-2,2-dimethyl-1,3-dioxolan-4-yl)-2,2-dimethyltetrahydrofuro[3,4-*d*][1,3]dioxol-4-yl)-5-phenyl-2*H*-tetrazole (3i)

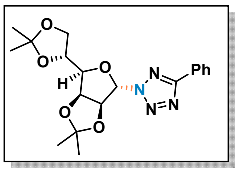

Purified by flash column chromatography (ethyl acetate/petroleum ether = 1:4.5, v:v); Colorless oil (49 mg, 65%); $[\alpha]_D^{25}$ = +16.6 (c = 0.9, $CHCl_3$); **^{1}H NMR** (400 MHz, $CDCl_3$) δ 8.17-8.12 (m, 2H), 7.53-7.47 (m, 3H), 6.49 (s, 1H), 5.35 (d, J = 5.8 Hz, 1H), 5.18 (dd, J = 5.9, 3.7 Hz, 1H), 4.47 (ddd, J = 7.5, 6.2, 4.5 Hz, 1H), 4.35 (dd, J = 7.5, 3.7 Hz, 1H), 4.09 (dd, J = 8.9, 6.2 Hz, 1H), 3.98 (dd, J = 8.9, 4.5 Hz, 1H), 1.59 (s, 3H), 1.44 (s, 3H), 1.42 (s, 3H), 1.37 (s, 3H); **^{13}C NMR** (101 MHz, $CDCl_3$) δ 164.5, 129.7, 127.9, 126.0, 125.8, 112.8, 108.4, 93.2, 83.8, 82.8, 79.1, 71.9, 65.5, 25.8, 25.0, 24.1, 23.5; **HRMS (ESI)** m/z cald for $C_{19}H_{25}N_4O_5^+$ $[M+H]^+$ 389.1819, found 389.1816.

9-((3a*S*,4*S*,6*R*,6a*S*)-6-((*R*)-2,2-dimethyl-1,3-dioxolan-4-yl)-2,2-dimethyltetrahydrofuro[3,4-*d*][1,3]dioxol-4-yl)-9*H*-carbazole (4a)

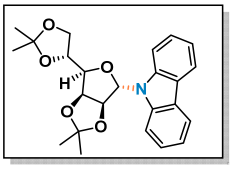

Purified by flash column chromatography (ethyl acetate/petroleum ether = 1:28, v:v); white solid (50 mg, 61%), mp: 84-85 °C; $[\alpha]_D^{25}$ = +15.2 (c = 1.9, $CHCl_3$); **^{1}H NMR** (400 MHz, $CDCl_3$) δ 8.09-8.06 (m, 2H), 7.48–7.39 (m, 4H), 7.29–7.24 (m, 2H), 6.39 (d, J = 1.5 Hz, 1H), 5.52 (dd, J = 5.9, 1.6 Hz, 1H), 5.21 (dd, J = 5.9, 3.7 Hz, 1H), 4.55–4.49 (m, 1H), 4.42 (dd, J = 7.2, 3.7 Hz, 1H), 4.11 (dd, J = 8.8, 6.2 Hz, 1H), 4.04 (dd, J = 8.9, 4.7 Hz, 1H), 1.66 (s, 3H), 1.44 (s, 6H), 1.39 (s, 3H); **^{13}C NMR** (101 MHz, $CDCl_3$) δ 139.6, 126.3, 124.2, 120.5, 120.2, 113.9, 110.1, 109.4, 92.2, 84.5, 83.6, 81.2, 73.7, 66.7, 27.0, 26.6, 25.4, 24.9. **HRMS (ESI)** m/z cald for $C_{24}H_{28}NO_5^+$ $[M+H]^+$ 410.1962, found 410.1953.

3-Bromo-9-((3a*S*,4*S*,6*R*,6a*S*)-6-((*R*)-2,2-dimethyl-1,3-dioxolan-4-yl)-2,2-dimethyltetrahydrofuro[3,4-*d*][1,3]dioxol-4-yl)-9*H*-carbazole (4b)

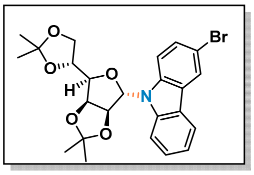

Purified by flash column chromatography (ethyl acetate/petroleum ether = 1:30, v:v); Colorless oil (64 mg, 66%); $[\alpha]_D^{25}$ = +33.3 (c = 2.0, $CHCl_3$). **^{1}H NMR** (400 MHz, $CDCl_3$) δ 8.18 (d, J = 1.9 Hz, 1H), 8.02 (d, J = 7.8 Hz, 1H), 7.53 (dd, J = 8.7, 2.0 Hz, 1H), 7.48 (ddd, J = 8.3, 7.0, 1.3 Hz, 1H), 7.40 (d, J = 8.3 Hz, 1H), 7.33 (d, J = 8.8 Hz, 1H), 7.31-7.26 (m, 1H), 6.33 (d, J = 1.6 Hz, 1H), 5.45 (dd, J = 5.9, 1.6 Hz, 1H), 5.17 (dd, J = 5.8, 3.6 Hz, 1H), 4.56–4.49 (m, 1H), 4.41 (dd, J = 6.9, 3.7 Hz, 1H), 4.12 (dd, J = 8.8, 6.3 Hz, 1H), 4.06 (dd, J = 8.9, 4.8 Hz, 1H), 1.66 (s, 3H), 1.45 (s, 3H), 1.44 (s, 3H), 1.40 (s, 3H); **^{13}C NMR** (101 MHz, $CDCl_3$) δ 139.8, 138.3, 128.9, 127.0, 125.9, 123.2, 123.2, 120.7, 120.6, 114.1, 113.1, 111.7, 110.4, 109.4, 92.5,

84.6, 83.6, 81.0, 73.7, 66.6, 27.0, 26.6, 25.3, 25.0; **HRMS (ESI)** m/z cald for $C_{24}H_{27}BrNO_5^+$ $[M+H]^+$ 488.1067, found 488.1070.

9-((3a*S*,4*S*,6*R*,6a*S*)-6-((*R*)-2,2-dimethyl-1,3-dioxolan-4-yl)-2,2-dimethyltetrahydrofuro[3,4-*d*][1,3]dioxol-4-yl)-9*H*-carbazole-3-carbonitrile (4c)

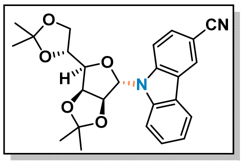

Purified by flash column chromatography (ethyl acetate/ petroleum ether = 1:25, v:v); white solid (55 mg, 63%), mp: 79-80 ºC; $[\alpha]_D^{25}$ = +33.4 (*c* = 1.3, $CHCl_3$); **^{1}H NMR** (400 MHz, $CDCl_3$) δ 8.28 (d, *J* = 1.6 Hz, 1H), 8.01 (d, *J* = 7.8 Hz, 1H), 7.61 (dd, *J* = 8.6, 1.6 Hz, 1H), 7.51–7.42 (m, 2H), 7.38 (d, *J* = 8.3 Hz, 1H), 7.28 (t, *J* = 7.5 Hz, 1H), 6.28 (d, *J* = 1.7 Hz, 1H), 5.32 (dd, *J* = 5.8, 1.7 Hz, 1H), 5.06 (dd, *J* = 5.8, 3.6 Hz, 1H), 4.52–4.37 (m, 2H), 4.04 (qd, *J* = 8.9, 5.5 Hz, 2H), 1.59 (s, 3H), 1.39 (s, 3H), 1.36 (s, 3H), 1.33 (s, 3H); **^{13}C NMR** (101 MHz, $CDCl_3$) δ 141.4, 139.8, 129.5, 127.7, 125.1, 124.3, 123.1, 121.5, 120.9, 120.2, 114.3, 111.2, 110.8, 109.4, 103.1, 93.0, 84.9, 83.7, 80.8, 73.7, 66.4, 26.9, 26.7 , 25.3, 25.0; **HRMS (ESI)** m/z cald for $C_{25}H_{27}N_2O_5^+$ $[M+H]^+$ 435.1914., found 435.1919.

9-((3a*S*,4*S*,6*R*,6a*S*)-6-((*R*)-2,2-dimethyl-1,3-dioxolan-4-yl)-2,2-dimethyltetrahydrofuro[3,4-*d*][1,3]dioxol-4-yl)-3-methoxy-9*H*-carbazole (4d)

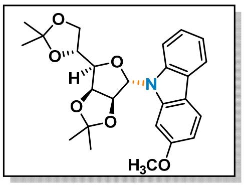

Purified by flash column chromatography (ethyl acetate/petroleum ether = 1:30, v:v); white solid (52 mg, 59%), mp: 124-125 ºC; $[\alpha]_D^{25}$ = +21.1 (*c* = 1.6, $CHCl_3$); **^{1}H NMR** (400 MHz, $CDCl_3$) δ 7.86 (dd, *J* = 16.8, 8.1 Hz, 2H), 7.32–7.24 (m, 2H), 7.18-7.13 (m, 1H), 6.87 (d, *J* = 2.1 Hz, 1H), 6.80 (dd, *J* = 8.6, 2.2 Hz, 1H), 6.24 (d, *J* = 1.4 Hz, 1H), 5.39 (dd, *J* = 5.8, 1.4 Hz, 1H), 5.09 (dd, *J* = 5.9, 3.6 Hz, 1H), 4.48-4.42 (m, 1H), 4.37 (dd, *J* = 7.0, 3.7 Hz, 1H), 4.06–3.97 (m, 2H), 3.84 (s, 3H), 1.58 (s, 3H), 1.36 (d, *J* = 5.7 Hz, 6H), 1.31 (s, 3H); **^{13}C NMR** (101 MHz, $CDCl_3$) δ 159.3, 141.0, 139.4, 124.9, 124.4, 121.0, 120.3, 119.6, 117.9, 113.9, 109.9, 109.3, 108.4, 95.2, 92.4, 84.6, 83.5, 81.1, 73.7, 66.6, 55.9, 27.0, 26.6, 25.3, 24.9; **HRMS (ESI)** m/z cald for $C_{25}H_{30}NO_6^+$ $[M+H]^+$ 440.2068., found 440.2072.

3,6-dichloro-9-((3a*S*,4*S*,6*R*,6a*S*)-6-((*R*)-2,2-dimethyl-1,3-dioxolan-4-yl)-2,2-dimethyltetrahydrofuro[3,4-*d*][1,3]dioxol-4-yl)-9*H*-carbazole (4e)

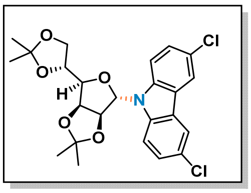

Purified by flash column chromatography (ethyl acetate/ petroleum ether = 1:25, v:v); white solid (56 mg, 59%), mp: 180-181 ºC; $[\alpha]_D^{25}$ = +35.4 (c = 1.2, CHCl$_3$); **^{1}H NMR** (400 MHz, CDCl$_3$) δ 7.96 (d, J = 2.0 Hz, 2H), 7.42 (dd, J = 8.8, 2.1 Hz, 2H), 7.36 (d, J = 8.8 Hz, 2H), 6.28 (d, J = 1.6 Hz, 1H), 5.36 (dd, J = 5.9, 1.6 Hz, 1H), 5.12 (dd, J = 5.8, 3.6 Hz, 1H), 4.56–4.48 (m, 1H), 4.41 (dd, J = 6.6, 3.7 Hz, 1H), 4.15–4.04 (m, 2H), 1.66 (s, 3H), 1.46 (s, 3H), 1.43 (s, 3H), 1.40 (s, 3H); **^{13}C NMR** (101 MHz, CDCl$_3$) δ 138.3, 127.0, 126.1, 124.4, 120.4, 114.3, 111.6, 109.4, 92.9, 84.8, 83.6, 80.8, 73.7, 66.4, 27.0, 26.7, 25.3, 25.0; **HRMS (ESI)** m/z cald for $NaC_{24}H_{25}Cl_2NO_5^+$ $[M+Na]^+$ 500.1002, found 500.1009.

3,6-dibromo-9-((3a*S*,4*S*,6*R*,6a*S*)-6-((*R*)-2,2-dimethyl-1,3-dioxolan-4-yl)-2,2-dimethyltetrahydrofuro[3,4-*d*][1,3]dioxol-4-yl)-9*H*-carbazole (4f)

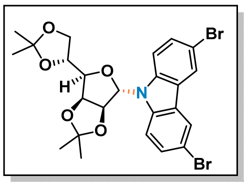

Purified by flash column chromatography (ethyl acetate/petroleum ether = 1:20, v:v); Colorless oil (70 mg, 62%) $[\alpha]_D^{25}$ = +25.0 (c = 2.1, CHCl$_3$); **^{1}H NMR** (400 MHz, CDCl$_3$) δ 8.09 (d, J = 2.0 Hz, 2H), 7.54 (d, J = 2.0 Hz, 1H), 7.52 (d, J = 2.0 Hz, 1H), 7.31 (s, 1H), 7.29 (s, 1H), 6.25 (d, J = 1.5 Hz, 1H), 5.34 (dd, J = 5.8, 1.6 Hz, 1H), 5.11 (dd, J = 5.8, 3.6 Hz, 1H), 4.52 (td, J = 6.4, 4.9 Hz, 1H), 4.39 (dd, J = 6.5, 3.6 Hz, 1H), 4.19 – 4.02 (m, J = 5.5 Hz, 2H), 1.66 (s, 3H), 1.46 (s, 3H), 1.43 (s, 3H), 1.40 (s, 3H); **^{13}C NMR** (101 MHz, CDCl$_3$) δ 138.4, 129.7, 124.8, 123.4, 114.2, 113.4, 112.0, 109.4, 92.9, 84.8, 83.6, 80.8, 73.7, 66.4, 26.9, 26.6, 25.3, 25.0; **HRMS (ESI)** m/z cald for $C_{24}H_{26}Br_2NO_5^+$ $[M+H]^+$ 566.0172, found 566.0186.

9-((3a*S*,4*S*,6*R*,6a*S*)-6-((*R*)-2,2-dimethyl-1,3-dioxolan-4-yl)-2,2-dimethyltetrahydrofuro[3,4-*d*][1,3]dioxol-4-yl)-9*H*-pyrido[2,3-*b*]indole (4g)

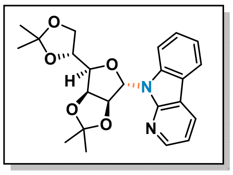

Purified by flash column chromatography (ethyl acetate/petroleum ether = 1:25, v:v); colorless oil (49 mg, 60%); $[\alpha]_D^{25}$ = +15.0 (c = 0.5, CHCl$_3$); **^{1}H NMR** (400 MHz, CDCl$_3$) δ 8.38 (dd, J = 4.9, 1.6 Hz, 1H), 8.29 (dd, J = 7.7, 1.6 Hz, 1H), 8.04 (d, J = 7.8 Hz, 1H), 7.60 (d, J = 8.3 Hz, 1H), 7.52 (ddd, J = 8.3, 7.2, 1.2 Hz, 1H), 7.33–7.29 (m, 1H), 7.18 (dd, J = 7.7, 4.9 Hz, 1H), 6.33 (s, 1H), 5.92 (d, J = 5.9 Hz, 1H), 5.48 (dd, J = 5.9, 2.8 Hz, 1H), 4.53–4.42 (m, 2H), 4.07–4.01 (m, 1H), 3.97–3.91 (m, 1H), 1.63 (s, 3H), 1.46 (s, 3H), 1.38 (s, 3H), 1.35 (s, 3H); **^{13}C NMR** (101 MHz, CDCl$_3$) δ 151.4, 145.7, 139.8, 128.2, 127.2, 121.1, 121.0, 120.9, 116.8, 116.2, 112.8, 109.9, 109.1, 89.0, 85.0, 84.3, 82.3, 73.9, 66.7, 27.0, 26.4, 25.4, 24.7; **HRMS (ESI)** m/z cald

for $C_{23}H_{27}N_2O_5^+$ [M+H]$^+$ 411.1914., found 411.1921.

1-(1-((3a*S*,4*S*,6*R*,6a*S*)-6-((*R*)-2,2-dimethyl-1,3-dioxolan-4-yl)-2,2-dimethyltetrahydrofuro[3,4-*d*][1,3]dioxol-4-yl)-1*H*-pyrrol-2-yl)ethan-1-one (4h)

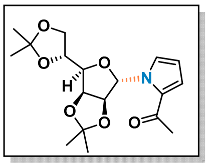

Purified by flash column chromatography (acetone / petroleum ether = 1:25, v:v); White solid; (29 mg, 42%), mp: 102-103 °C; $[\alpha]_D^{25}$ = -16.0 (c =0.6, $CHCl_3$);**^{1}H NMR** (400 MHz, $CDCl_3$) δ 7.05–7.02 (m, 2H), 6.44 (s, 1H), 6.20 (dd, *J* = 3.9, 2.8 Hz, 1H), 4.80 (d, *J* = 2.4 Hz, 2H), 4.53–4.47 (m, 1H), 4.24 (dd, *J* = 7.0, 2.6 Hz, 1H), 4.16–4.13 (m, 2H), 2.44 (s, 3H), 1.58 (s, 3H), 1.46 (s, 3H), 1.39 (s, 3H), 1.36 (s, 3H),**^{13}C NMR** (101 MHz, $CDCl_3$) δ 186.9, 129.4, 124.6, 120.2, 112.1, 108.2, 107.7, 93.3, 87.2, 82.3, 78.8, 72.4, 65.6, 25.9, 25.8, 25.2, 24.1, 23.8; **HRMS (ESI)** m/z cald for $C_{18}H_{26}NO_6^+$ [M+H]$^+$ 352.1755, found 352.1748.

Methyl1-((3a*S*,4*S*,6*R*,6a*S*)-6-((*R*)-2,2-dimethyl-1,3-dioxolan-4-yl)-2,2-dimethyltetrahydrofuro[3,4-*d*][1,3]dioxol-4-yl)- 1*H*-pyrrole-3-carboxylate (4i)

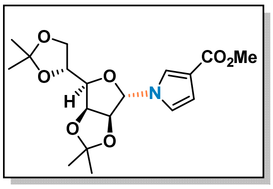

Purified by flash column chromatography (ethyl acetate/petroleum ether = 1:25, v:v); colorless oil (40 mg, 55%), $[\alpha]_D^{25}$ = +96.3 (*c* =0.5, $CHCl_3$); **^{1}H NMR** (400 MHz, $CDCl_3$) δ 7.32-7.28 (m, 1H), 6.67–6.63 (m, 1H), 6.64–6.59 (m, 1H), 5.71 (s, 1H), 5.06 (d, *J* = 5.8 Hz, 1H), 4.93 (dd, *J* = 5.9, 3.7 Hz, 1H), 4.41 (ddd, *J* = 7.4, 6.3, 4.5 Hz, 1H), 4.09 (dd, *J* = 8.8, 6.3 Hz, 1H), 4.03–3.95 (m, 2H), 3.78 (s, 3H), 1.53 (s, 3H), 1.41 (s, 3H), 1.36 (s, 3H), 1.35 (s, 1H); **^{13}C NMR** (101 MHz, $CDCl_3$) δ 165.0, 123.6 (d, *J* = 1.9 Hz), 119.9, 117.4, 113.7, 111.2, 109.5, 92.7 (d, *J* = 4.2 Hz), 85.5, 82.8, 80.3, 73.0, 66.8, 51.2 (d, *J* = 4.1 Hz), 26.9, 26.0, 25.2, 24.6; **HRMS (ESI)** m/z cald for $C_{18}H_{26}NO_7^+$ [M+H]$^+$ 368.1704, found 368.1701.

Ethyl1-((3a*S*,4*S*,6*R*,6a*S*)-6-((*R*)-2,2-dimethyl-1,3-dioxolan-4-yl)-2,2-dimethyltetrahydrofuro[3,4-*d*][1,3]dioxol-4-yl)-1*H*-indole-2-carboxylate (5a)

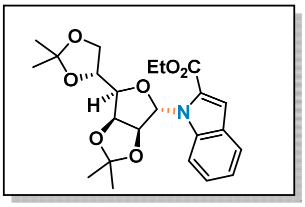

Purified by flash column chromatography (acetone/petroleum ether = 1:30, v:v); colorless oil (49 mg, 57%), $[\alpha]_D^{25}$ = +29.9 (*c* =1.5, $CHCl_3$); **^{1}H NMR** (400 MHz, DMSO-*d*$_6$) δ 7.74 (d, *J* = 7.9 Hz, 1H), 7.54 (d, *J* = 8.5 Hz, 1H), 7.43 (s, 1H), 7.42-7.36 (m, 1H), 7.23-7.17 (m, 1H), 6.93 (d, *J* = 3.1 Hz, 1H), 5.38 (dd, *J* = 5.8, 3.2 Hz, 1H), 5.22 (dd, *J* = 5.8, 4.0 Hz, 1H), 4.55 (dd, *J* = 6.0, 4.0 Hz, 1H), 4.38–4.27 (m, 3H), 4.00 (dd, *J* = 8.5, 6.5 Hz, 1H), 3.81 (dd, *J* = 8.6, 5.6 Hz, 1H), 1.56 (s, 3H), 1.35–1.25 (m, 12H); **^{13}C**

NMR (101 MHz, DMSO-d_6) δ 162.2, 138.7, 128.5, 127.3, 126.9, 123.9, 122.4, 114.4, 113.9, 113.2, 109.0, 92.5, 85.1, 83.6, 81.7, 74.5, 66.5, 61.9, 27.7, 27.5, 26.0, 25.9, 15.1; **HRMS (ESI)** m/z cald for $NaC_{23}H_{29}NO_7^+$ $[M+Na]^+$ 454.1836, found 454.1827.

Methyl1-((3a*S*,4*S*,6*R*,6a*S*)-6-((*R*)-2,2-dimethyl-1,3-dioxolan-4-yl)-2,2-dimethyltetrahydrofuro[3,4-*d*][1,3]dioxol-4-yl)-1*H*-indole-3-carboxylate (5b)

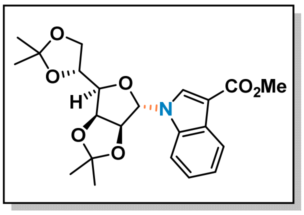

Purified by flash column chromatography (ethyl acetate/petroleum ether = 1:15, v:v); colorless oil (42 mg, 51%), $[\alpha]_D^{25}$ = +55.3 (*c* =0.7, $CHCl_3$); **^{1}H NMR** (400 MHz, $CDCl_3$) δ 8.21-8.16 (m, 1H), 7.77 (s, 1H), 7.53–7.44 (m, 1H), 7.38–7.28 (m, 2H), 6.13 (s, 1H), 5.20 (d, *J* = 5.8 Hz, 1H), 5.01 (dd, *J* = 5.9, 3.6 Hz, 1H), 4.49 (ddd, *J* = 7.3, 6.3, 4.5 Hz, 1H), 4.17–4.06 (m, 2H), 4.03 (dd, *J* = 8.9, 4.5 Hz, 1H), 3.93 (s, 3H), 1.62 (s, 3H), 1.44 (s, 3H), 1.42 (s, 3H), 1.38 (s, 3H); **^{13}C NMR** (101 MHz, $CDCl_3$) δ 165.4, 136.0, 130.1, 127.0, 123.7, 122.8, 122.1, 114.0, 110.8, 109.6, 109.0, 91.2, 85.3, 83.1, 80.4, 73.2, 66.8, 51.3, 27.0, 26.3, 25.2, 24.8. **HRMS (ESI)** m/z cald for $C_{22}H_{28}NO_7^+$ $[M+H]^+$ 418.1860, found 418.1862.

1-((3a*S*,4*S*,6*R*,6a*S*)-6-((*R*)-2,2-dimethyl-1,3-dioxolan-4-yl)-2,2-dimethyltetrahydrofuro[3,4-*d*][1,3]dioxol-4-yl)-1*H*-indole-3-carbonitrile (5c)

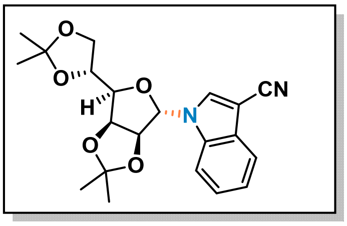

Purified by flash column chromatography (ethyl acetate/ petroleum ether = 1:25, v:v); Colorless oil (39 mg, 51%), $[\alpha]_D^{25}$ = +50.1 (*c* =1.0, $CHCl_3$); **^{1}H NMR** (400 MHz, $CDCl_3$) δ 7.80–7.75 (m, 1H), 7.63 (s, 1H), 7.53–7.48 (m, 1H), 7.31–7.41 (m, 2H), 6.08 (s, 1H), 5.14 (d, *J* = 5.9 Hz, 1H), 4.96 (dd, *J* = 5.8, 3.5 Hz, 1H), 4.51 (td, *J* = 6.6, 4.5 Hz, 1H), 4.18–4.13 (m, 2H), 4.08 (dd, *J* = 9.0, 4.5 Hz, 1H), 1.62 (s, 3H), 1.46 (s, 3H), 1.42 (s, 3H), 1.40 (s, 3H). **^{13}C NMR** (100 MHz, $CDCl_3$) δ 134.3, 130.8, 128.4, 124.6, 123.0, 120.3, 115.5, 114.3, 111.4, 109.7, 92.1, 87.6, 85.4, 83.5, 80.3, 73.1, 66.6, 27.0, 26.3, 25.2, 24.9; **HRMS (ESI)** m/z cald for $C_{21}H_{25}N_2O_5^+$ $[M+H]^+$ 385.1758, found 385.1760.

4-bromo-1-((3a*S*,4*S*,6*R*,6a*S*)-6-((*R*)-2,2-dimethyl-1,3-dioxolan-4-yl)-2,2-dimethyltetrahydrofuro[3,4-*d*][1,3]dioxol-4-yl)-1*H*-indole (5d)

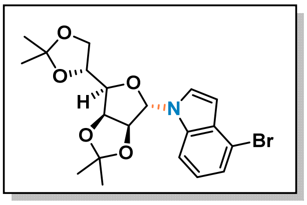

Purified by flash column chromatography (acetone/petroleum ether = 1:35, v:v); colorless oil; (55 mg, 62%), $[\alpha]_D^{25}$ = +57.3 (c =1, $CHCl_3$); **^{1}H NMR** (400 MHz, $CDCl_3$) δ 7.40 (d, J = 8.3 Hz, 1H), 7.34 (d, J = 7.6 Hz, 1H), 7.14–7.08 (m, 2H), 6.61 (d, J = 3.4 Hz, 1H), 6.10 (s, 1H), 5.20 (d, J = 5.8 Hz, 1H), 5.00 (dd, J = 5.8, 3.7 Hz, 1H), 4.48 (td, J = 6.8, 4.4 Hz, 1H), 4.11 (dd, J = 8.9, 6.3 Hz, 1H), 4.06–3.97 (m, 2H), 1.61 (s, 3H), 1.42 (s, 6H), 1.38 (s, 3H). **^{13}C NMR** (100 MHz, $CDCl_3$) δ 135.9 , 129.7 , 124.4 (d, J = 2.4 Hz), 123.5 , 123.3 , 115.0 , 113.7 , 109.4 , 109.4 , 103.5 (d, J = 2.2 Hz), 90.7 , 85.0 , 82.6 , 80.3 , 73.1 , 66.7 , 26.9 , 26.1 , 25.1 , 24.7; **HRMS (ESI)** m/z cald for $C_{20}H_{25}BrNO_5^+$ $[M+H]^+$ 438.0911, found 438.0925.

4-chloro-1-((3a*S*,4*S*,6*R*,6a*S*)-6-((*R*)-2,2-dimethyl-1,3-dioxolan-4-yl)-2,2-dimethyltetrahydrofuro[3,4-*d*][1,3]dioxol-4-yl)-1*H*-indole (5e)

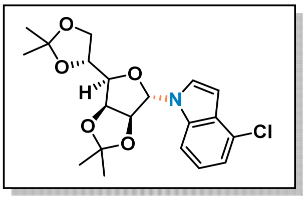

Purified by flash column chromatography (ethyl acetate/petroleum ether = 1:40, v:v); white solid; (42 mg, 54%), mp: 125 °C; $[\alpha]_D^{25}$ = +49.0 (c =0.5, $CHCl_3$); **^{1}H NMR** (400 MHz, $CDCl_3$) δ 7.39–7.33 (m, 1H), 7.18–7.15 (m, 2H), 7.08 (d, J = 3.4 Hz, 1H), 6.67 (dd, J = 3.4, 0.8 Hz, 1H), 6.11 (s, 1H), 5.21 (d, J = 5.8 Hz, 1H), 5.01 (dd, J = 5.8, 3.6 Hz, 1H), 4.47 (ddd, J = 7.6, 6.3, 4.4 Hz, 1H), 4.11 (dd, J = 8.9, 6.3 Hz, 1H), 4.06–3.97 (m, 2H), 1.61 (s, 3H), 1.42 (s, 6H), 1.38 (s, 3H). **^{13}C NMR** (100 MHz, $CDCl_3$) δ 136.5, 128.0, 126.5, 124.6, 123.2, 120.4, 113.8, 109.6, 109.0, 101.9, 90.9, 85.2, 82.8, 80.5, 73.2, 66.9, 27.0, 26.3, 25.3, 24.8; **HRMS (ESI)** m/z cald for $C_{20}H_{25}ClNO_5^+$ $[M+H]^+$ 394.1416, found 394.1409.

1-((3a*S*,4*S*,6*R*,6a*S*)-6-((*R*)-2,2-dimethyl-1,3-dioxolan-4-yl)-2,2-dimethyltetrahydrofuro[3,4-*d*][1,3]dioxol-4-yl)-5-fluoro-1*H*-indole (5f)

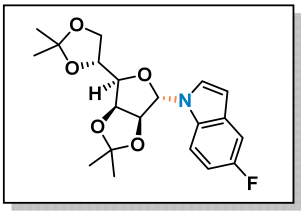

Purified by flash column chromatography (ethyl acetate/ petroleum ether = 1:35, v:v); Colorless oil; (43 mg, 58%), $[\alpha]_D^{25}$ = + 68.5 (c =0.8, $CHCl_3$); **^{1}H NMR** (400 MHz, $CDCl_3$) δ 7.37 (dd, J = 8.9, 4.3 Hz, 1H), 7.31–7.22 (m, 1H), 7.07 (d, J = 3.3 Hz, 1H), 7.00 (td, J = 9.1, 2.6 Hz, 1H), 6.51 (d, J = 3.3 Hz, 1H), 6.09 (s, 1H), 5.20 (d, J = 5.8 Hz, 1H), 5.00 (dd, J = 5.9, 3.6 Hz, 1H), 4.47 (ddd, J = 7.6, 6.3, 4.3 Hz, 1H), 4.11 (dd, J = 8.9, 6.3 Hz, 1H), 4.03–3.97 (m, 2H), 1.61 (s, 3H), 1.42 (s, 6H), 1.38 (s, 3H); **^{13}C NMR** (101 MHz, $CDCl_3$) δ 158.43 (d, J = 235.6 Hz), 132.4 , 129.5 (d, J = 10.1 Hz), 125.6, 113.7, 111.0 (d, J = 2.7 Hz), 110.9 (d, J = 13.8 Hz), 109.6, 106.0 (d, J = 23.5 Hz), 103.3 (d, J = 4.6 Hz), 90.7, 85.1, 82.6, 80.5, 73.2, 66.9, 27.0, 26.2, 25.2, 24.8. **^{19}F NMR** (376 MHz, $CDCl_3$) δ -123.97 (td,

J = 9.4, 2.9 Hz, 1F); ^{19}**F {^{1}H} NMR** (376 MHz, $CDCl_3$) δ -123.97 (s, 1F); **HRMS (ESI)** m/z cald for $C_{20}H_{25}FNO_5^+$ $[M+H]^+$ 378.1711, found 378.1703.

5-chloro-1-((3a*S*,4*S*,6*R*,6a*S*)-6-((*R*)-2,2-dimethyl-1,3-dioxolan-4-yl)-2,2-dimethyltetrahydrofuro[3,4-*d*][1,3]dioxol-4-yl)-1*H*-indole (5g)

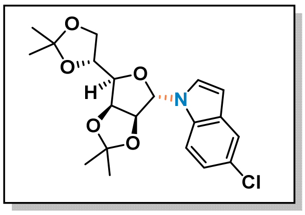

Purified by flash column chromatography (ethyl acetate/ petroleum ether = 1:35, v:v); colorless oil; (41 mg, 53%), $[\alpha]_D^{25}$ = +47.5 (c = 0.7, $CHCl_3$); ^{1}**H NMR** (400 MHz, $CDCl_3$) δ 7.59 (d, J = 1.9 Hz, 1H), 7.37 (d, J = 8.8 Hz, 1H), 7.20 (dd, J = 8.7, 1.9 Hz, 1H), 7.06 (d, J = 3.4 Hz, 1H), 6.49 (d, J = 3.3 Hz, 1H), 6.09 (s, 1H), 5.20 (d, J = 5.8 Hz, 1H), 5.00 (dd, J = 5.9, 3.7 Hz, 1H), 4.47 (td, J = 6.8, 4.4 Hz, 1H), 4.11 (dd, J = 9.0, 6.3 Hz, 1H), 4.03-3.98 (m, 2H), 1.57 (s, 3H), 1.42 (s, 6H), 1.37 (s, 3H). ^{13}**C NMR** (101 MHz, $CDCl_3$) δ 134.2, 130.2, 126.4, 125.3, 122.9, 120.6, 113.8, 111.3, 109.6, 103.0, 90.6, 85.1, 82.7, 80.4, 73.2, 66.9, 27.0, 26.2, 25.2, 24.8; **HRMS (ESI)** m/z cald for $NaC_{20}H_{24}ClNO_5^+$ $[M+Na]^+$ 416.1241, found 416.1236.

5-Bromo-1-((3a*S*,4*S*,6*R*,6a*S*)-6-((*R*)-2,2-dimethyl-1,3-dioxolan-4-yl)-2,2-dimethyltetrahydrofuro[3,4-*d*][1,3]dioxol-4-yl)-1*H*-indole (5h)

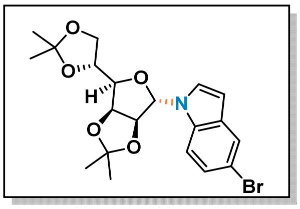

Purified by flash column chromatography (ethyl acetate/ petroleum ether = 1:35, v:v); colorless oil; (49 mg, 56%), $[\alpha]_D^{25}$ = +63.3 (c =0.9, $CHCl_3$); ^{1}**H NMR** (400 MHz, $CDCl_3$) δ 7.80 (s, 1H), 7.30 (s, 2H) 7.04 (d, J = 3.3 Hz, 1H), 6.49 (d, J = 3.3 Hz, 1H), 6.08 (s, 1H), 5.19 (d, J = 5.8 Hz, 1H), 5.00 (dd, J = 5.9, 3.6 Hz, 1H), 4.50–4.44 (m, 1H), 4.11 (dd, J = 8.8, 6.3 Hz, 1H), 4.05 – 3.96 (m, 2H), 1.61 (s, 3H), 1.42 (s, 6H), 1.37 (s, 3H); ^{13}**C NMR** (101 MHz, $CDCl_3$) δ 133.3, 129.7, 124.2, 124.0, 122.6, 112.8, 112.6, 110.6, 108.4, 101.7, 89.5, 84.0, 81.6, 79.3, 72.0, 65.7, 25.9, 25.1, 24.1, 23.6. **HRMS (ESI)** m/z cald for $C_{20}H_{25}BrNO_5^+$ $[M+H]^+$ 438.0911, found 438.0914.

5-(benzyloxy)-1-((3a*S*,4*S*,6*R*,6a*S*)-6-((*R*)-2,2-dimethyl-1,3-dioxolan-4-yl)-2,2-dimethyltetrahydrofuro[3,4-*d*][1,3]dioxol-4-yl)-1*H*-indole (5i)

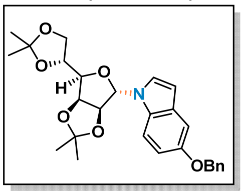

Purified by flash column chromatography (acetone/ petroleum ether = 1:40, v:v); colorless oil; (34 mg, 24%), $[\alpha]_D^{25}$ = +62.1 (c =0.8, $CHCl_3$); ^{1}**H NMR** (400 MHz, $CDCl_3$) δ 7.48 (d, J = 7.0 Hz, 2H), 7.42–7.29 (m, 4H), 7.17 (d, J = 2.4 Hz, 1H), 7.00 (dd, J = 8.5, 2.7 Hz, 2H), 6.47 (d, J = 3.2

Hz, 1H), 6.08 (s, 1H), 5.22 (d, J = 5.8 Hz, 1H), 5.12 (s, 2H), 5.00 (dd, J = 5.9, 3.7 Hz, 1H), 4.47 (ddd, J = 7.6, 6.2, 4.4 Hz, 1H), 4.11 (dd, J = 8.8, 6.3 Hz, 1H), 4.00 (ddd, J = 10.9, 8.2, 4.0 Hz, 2H), 1.61 (s, 3H), 1.42 (s, 6H), 1.38 (s, 3H); **^{13}C NMR** (101 MHz, $CDCl_3$) δ 154.0, 137.7, 131.3, 129.6, 128.7, 127.9, 127.6, 124.7, 113.6, 113.5, 111.0, 109.5, 104.6, 103.1, 90.6, 85.1, 82.5, 80.5, 73.3, 71.0, 66.9, 27.0, 26.2, 25.3, 24.8; **HRMS (ESI)** m/z cald for $C_{27}H_{31}NO_6Na^+$ $[M+Na]^+$ 488.2044, found 488.2047.

1-((3a*S*,4*S*,6*R*,6a*S*)-6-((*R*)-2,2-dimethyl-1,3-dioxolan-4-yl)-2,2-dimethyltetrahydrofuro[3,4-*d*][1,3]dioxol-4-yl)-6-(trifluoromethyl)-1*H*-indole (5j)

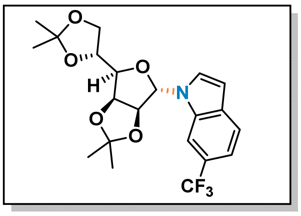

Purified by flash column chromatography (ethyl acetate/ petroleum ether = 1:35, v:v); Colorless oil; (51 mg, 60%), $[\alpha]_D^{25}$ = +44.9 (c =2.2, $CHCl_3$); **^{1}H NMR** (400 MHz, $CDCl_3$) δ 7.77 (s, 1H), 7.71 (d, J = 8.3 Hz, 1H), 7.40 (dd, J = 8.4, 1.5 Hz, 1H), 7.18 (d, J = 3.3 Hz, 1H), 6.62 (dd, J = 3.3, 0.8 Hz, 1H), 6.17 (s, 1H), 5.24 (d, J = 5.9 Hz, 1H), 5.03 (dd, J = 5.8, 3.6 Hz, 1H), 4.48 (ddd, J = 7.4, 6.3, 4.3 Hz, 1H), 4.11 (dd, J = 8.9, 6.3 Hz, 1H), 4.04–3.96 (m, 2H), 1.62 (s, 3H), 1.43 (s, 3H), 1.42 (s, 3H), 1.38 (s, 3H). **^{13}C NMR** (100 MHz, $CDCl_3$) δ 133.8, 130.3, 124.0 (q, J = 272 Hz), 125.5, 123.6 (q, J = 32 Hz), 120.4, 116.2 (q, J = 3.4 Hz), 112.7, 108.4, 106.8, 102.5, 89.4, 83.9, 81.5, 79.3, 72.0, 65.6, 25.8, 25.0, 24.1, 23.6. **^{19}F NMR** (376 MHz, $CDCl_3$) δ -60.6 (s, 3F); **^{19}F {H}NMR** (376 MHz, $CDCl_3$) δ -60.6 (s, 3F); **HRMS (ESI)** m/z cald for $C_{21}H_{25}F_3NO_5^+$ $[M+H]^+$ 428.1679, found 428.1674.

1-((3a*S*,4*S*,6*R*,6a*S*)-6-((*R*)-2,2-dimethyl-1,3-dioxolan-4-yl)-2,2-dimethyltetrahydrofuro[3,4-*d*][1,3]dioxol-4-yl)-3-methyl-1*H*-pyrrolo[2,3-b]pyridine (5k)

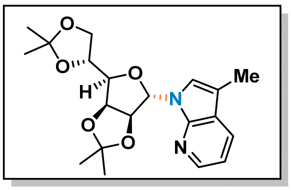

Purified by flash column chromatography (acetone/petroleum ether = 1:50, v:v); Colorless oil; (34 mg, 45%), $[\alpha]_D^{25}$ = -25.4 (c =2.4, $CHCl_3$); **^{1}H NMR** (400 MHz, $CDCl_3$) δ 8.23 (dd, J = 4.7, 1.6 Hz, 1H), 7.81 (dd, J = 7.8, 1.6 Hz, 1H), 7.05 (dd, J = 7.8, 4.7 Hz, 1H), 6.94 (d, J = 1.3 Hz, 1H), 6.01 (s, 1H), 5.62 (d, J = 5.9 Hz, 1H), 5.31 (dd, J = 5.9, 3.7 Hz, 1H), 4.44 (ddd, J = 7.4, 6.1, 4.4 Hz, 1H), 4.38 (dd, J = 7.3, 3.7 Hz, 1H), 4.05 (dd, J = 8.7, 6.1 Hz, 1H), 3.97 (dd, J = 8.7, 4.4 Hz, 1H), 2.27 (d, J = 1.2 Hz, 3H), 1.58 (s, 3H), 1.41 (s, 3H), 1.40 (s, 3H), 1.35 (s, 3H); **^{13}C NMR** (101 MHz, $CDCl_3$) δ 147.5, 142.9, 127.2, 125.4, 122.1, 115.9, 112.9, 110.4, 109.1, 91.5, 85.2, 83.7, 81.7, 73.7, 66.7, 27.0, 26.3, 25.2, 24.7, 9.7; **HRMS (ESI)** m/z cald for $C_{20}H_{27}N_2O_5^+$ $[M+H]^+$ 375.1914, found 375.1910.

4-Bromo-1-((3a*S*,4*S*,6*R*,6a*S*)-6-((*R*)-2,2-dimethyl-1,3-dioxolan-4-yl)-2,2-dimethyltetrahydrofuro[3,4-*d*][1,3]dioxol-4-yl)-1*H*-pyrrolo[2,3-b]pyridine (5l)

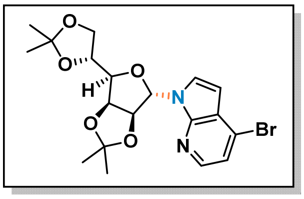

Purified by flash column chromatography (acetone/ petroleum ether = 1:50, v:v); Colorless oil; (45 mg, 51%), $[\alpha]_D^{25}$ = -9.0 (c =2.1, $CHCl_3$); ^{1}**H NMR** (400 MHz, $CDCl_3$) δ 8.06 (d, J = 5.1 Hz, 1H), 7.29–7.24 (m, 2H), 6.51 (d, J = 3.6 Hz, 1H), 6.02 (s, 1H), 5.59 (d, J = 5.9 Hz, 1H), 5.28 (dd, J = 5.9, 3.3 Hz, 1H), 4.49–4.37 (m, 2H), 4.06 (dd, J = 8.8, 5.8 Hz, 1H), 3.98 (dd, J = 8.7, 4.0 Hz, 1H), 1.57 (s, 3H), 1.41 (s, 3H), 1.40 (s, 3H), 1.35 (s, 3H); ^{13}**C NMR** (101 MHz, $CDCl_3$) δ 146.9, 143.2, 128.8, 125.4, 123.1, 119.9, 113.1, 109.1, 101.2, 92.3, 85.2, 84.2, 81.6, 73.6, 66.5, 27.0, 26.2, 25.2, 24.7; **HRMS (ESI)** m/z cald for $C_{19}H_{24}BrN_2O_5^+$ $[M+H]^+$ 439.0863., found 439.0869.

4-chloro-1-((3a*S*,4*S*,6*R*,6a*S*)-6-((*R*)-2,2-dimethyl-1,3-dioxolan-4-yl)-2,2-dimethyltetrahydrofuro[3,4-*d*][1,3]dioxol-4-yl)-1*H*-pyrrolo[2,3-b]pyridine (5m)

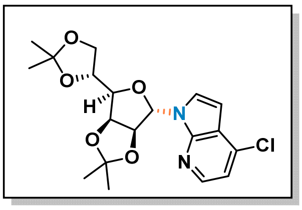

Purified by flash column chromatography (acetone/ petroleum ether = 1:50, v:v); Colorless oil; (37 mg, 47%), $[\alpha]_D^{25}$ = -16.7 (c =3.2, $CHCl_3$); ^{1}**H NMR** (400 MHz, $CDCl_3$) δ 8.14 (d, J = 5.2 Hz, 1H), 7.23 (d, J = 3.6 Hz, 1H), 7.10 (d, J = 5.2 Hz, 1H), 6.56 (d, J = 3.6 Hz, 1H), 6.02 (s, 1H), 5.59 (d, J = 5.9 Hz, 1H), 5.28 (dd, J = 5.9, 3.2 Hz, 1H), 4.48–4.40 (m, 2H), 4.06 (dd, J = 8.7, 5.7 Hz, 1H), 3.98 (dd, J = 8.7, 3.9 Hz, 1H), 1.56 (s, 3H), 1.41 (s, 3H), 1.40 (s, 3H), 1.35 (s, 3H).; ^{13}**C NMR** (101 MHz, $CDCl_3$) δ 147.7, 143.4, 136.2, 128.7, 120.9, 116.8, 113.1, 109.1, 99.6, 92.3, 85.2, 84.1, 81.6, 73.6, 66.5, 26.9, 26.2, 25.2, 24.7; **HRMS (ESI)** m/z cald for $C_{19}H_{24}ClN_2O_5^+$ $[M+H]^+$ 395.1368, found 395.1378.

5-bromo-1-((3a*S*,4*S*,6*R*,6a*S*)-6-((*R*)-2,2-dimethyl-1,3-dioxolan-4-yl)-2,2-dimethyltetrahydrofuro[3,4-*d*][1,3]dioxol-4-yl)-1*H*-pyrrolo[2,3-*b*]pyridine (5n)

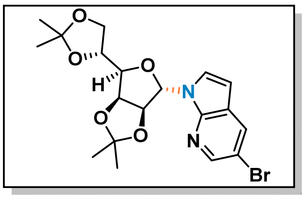

Purified by flash column chromatography (ethyl acetate/ petroleum ether = 1:40, v:v); White solid; (39 mg, 45%), mp: 136 °C; $[\alpha]_D^{25}$ = +15.7(c =0.45, $CHCl_3$); ^{1}**H NMR** (400 MHz, $CDCl_3$) δ 8.29 (d, J = 2.2 Hz, 1H), 8.01 (d, J = 2.2 Hz, 1H), 7.22 (d, J = 3.6 Hz, 1H), 6.42 (d, J = 3.6 Hz, 1H), 6.00 (s, 1H), 5.58 (d, J = 5.9 Hz, 1H), 5.27 (dd, J = 5.9, 3.4 Hz, 1H), 4.48–4.39 (m, 2H), 4.06 (dd, J = 8.8, 5.7 Hz, 1H), 3.98 (dd, J = 8.7, 4.1 Hz, 1H), 1.58 (s, 3H), 1.41 (s, 6H), 1.36 (s, 3H); ^{13}**C NMR** (101 MHz, $CDCl_3$) δ 145.6,

143.7, 131.1, 129.9, 123.3, 113.2, 112.7, 109.2, 100.7, 92.2, 85.3, 84.2 , 81.7, 73.7, 66.6, 27.0, 26.3, 25.3, 24.7; **HRMS (ESI)** m/z cald for $C_{19}H_{24}BrN_2O_5^+$ $[M+H]^+$ 439.0863, found 439.0854.

***N*-(1-((3a*S*,4*S*,6*R*,6a*S*)-6-((*R*)-2,2-dimethyl-1,3-dioxolan-4-yl)-2,2-dimethyltetrahydrofuro[3,4-*d*][1,3]dioxol-4-yl)-2-oxo-1,2-dihydropyrimidin-4-yl)benzamide (6a)**

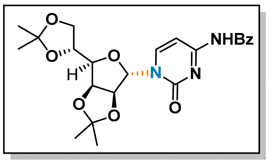

Purified by flash column chromatography (ethyl acetate / petroleum ether = 1:6, v:v); White solid; (50 mg, 55%), m.p.: 190 °C; $[\alpha]_D^{25}$ = +57.3 (*c* = 1.0, $CHCl_3$); **^{1}H NMR** [1] (400 MHz, $CDCl_3$) δ 8.61 (s, 1H), 8.50 (d, *J* = 5.7 Hz, 1H), 8.03 (d, *J* = 5.6 Hz, 1H), 7.93–7.86 (m, 2H), 7.65–7.56 (m, 1H), 7.51 (dd, *J* = 8.3, 6.8 Hz, 2H), 6.46 (s, 1H), 4.95–4.87 (m, 2H), 4.43 (ddd, *J* = 8.2, 6.1, 3.9 Hz, 1H), 4.19 (dd, *J* = 8.3, 3.1 Hz, 1H), 4.08 (dd, *J* = 8.9, 6.1 Hz, 1H), 4.00 (dd, *J* = 8.9, 3.9 Hz, 1H), 1.52 (s, 3H), 1.42 (s, 3H), 1.37 (s, 3H), 1.35 (s, 3H); **^{13}C NMR** (101 MHz, $CDCl_3$) δ 166.0, 163.0, 161.0, 159.4, 133.2, 133.1, 129.2, 127.5, 113.4, 109.6, 105.2, 103.2, 85.2, 82.3, 79.6, 73.0, 67.1, 27.1, 26.2, 25.3, 24.8; **HRMS (ESI)** m/z cald for $C_{23}H_{27}N_3O_7Na^+$ $[M+Na]^+$ 480.1741, found 480.1735.

3-Benzoyl-1-((3a*S*,4*S*,6*R*,6a*S*)-6-((*R*)-2,2-dimethyl-1,3-dioxolan-4-yl)-2,2-dimethyltetrahydrofuro[3,4-*d*][1,3]dioxol-4-yl)pyrimidine-2,4(1*H*,3*H*)-dione (6b)

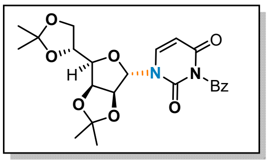

Purified by flash column chromatography (MeOH/toluene = 1:5, v:v); White solid (43 mg, 47%), m.p.: 156-157 °C; $[\alpha]_D^{25}$ = +22.2 (*c* = 0.6, $CHCl_3$); **^{1}H NMR** (400 MHz, DMSO-*d*$_6$) δ 8.79 (d, *J* = 5.4 Hz, 1H), 8.18–8.09 (m, 2H), 7.84–7.77 (m, 1H), 7.68–7.60 (m, 2H), 7.30 (d, *J* = 5.4 Hz, 1H), 6.32 (s, 1H), 4.92 (dd, *J* = 5.9, 3.4 Hz, 1H), 4.88 (d, *J* = 5.9 Hz, 1H), 4.35–4.26 (m, 1H), 4.15 (dd, *J* = 6.4, 3.4 Hz, 1H), 4.00 (dd, *J* = 8.5, 6.5 Hz, 1H), 3.82 (dd, *J* = 8.5, 5.3 Hz, 1H), 1.41 (s, 3H), 1.32 (s, 3H), 1.29 (s, 3H), 1.26 (s, 3H). **^{13}C NMR** (101 MHz, DMSO-*d*$_6$) δ 166.5, 163.2, 163.1, 162.5, 135.0, 130.2, 129.3, 127.8, 112.2, 108.6, 108.2, 103.3, 84.5, 82.0, 78.9, 72.5, 65.7, 26.6, 25.8, 25.2, 24.4; **HRMS (ESI)** m/z cald for $C_{23}H_{26}N_2O_8Na^+$ $[M+Na]^+$ 481.1581, found 481.1584.

3-benzoyl-1-((3a*S*,4*S*,6*R*,6a*S*)-6-((*R*)-2,2-dimethyl-1,3-dioxolan-4-yl)-2,2-dimethyltetrahydrofuro[3,4-*d*][1,3]dioxol-4-yl)-5-methylpyrimidine-2,4(1*H*,3*H*)-dione (6c)

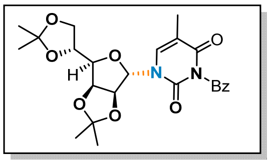

Purified by flash column chromatography (MeOH/toluene = 1:5, v:v); White solid (44 mg, 46%), m.p.: 88-89 °C; $[\alpha]_D^{25}$ = +49.8 (c = 0.3, $CHCl_3$); **^{1}H NMR** (400 MHz, DMSO-d_6) δ 8.67 (d, J = 1.0 Hz, 1H), 8.22–8.12 (m, 2H), 7.86–7.78 (m, 1H), 7.72–7.61 (m, 2H), 6.28 (s, 1H), 4.91 (dd, J = 5.9, 3.4 Hz, 1H), 4.86 (d, J = 5.9 Hz, 1H), 4.30 (td, J = 6.4, 5.3 Hz, 1H), 4.13 (dd, J = 6.4, 3.4 Hz, 1H), 3.99 (dd, J = 8.5, 6.4 Hz, 1H), 3.81 (dd, J = 8.5, 5.3 Hz, 1H), 2.12 (s, 2H), 1.40 (s, 3H), 1.31 (s, 3H), 1.28 (s, 3H), 1.26 (s, 3H); **^{13}C NMR** (101 MHz, DMSO-d_6) δ 164.9, 162.9, 162.5, 161.7, 135.0, 130.2, 129.4, 127.5, 117.1, 112.1, 108.2, 103.2, 84.4, 81.9, 78.9, 72.5, 65.7, 26.6, 25.8, 25.2, 24.4, 11.6; **HRMS (ESI)** m/z cald for $C_{24}H_{28}N_2O_8Na^+$ $[M+Na]^+$ 495.1738, found 495.1730.

_N_-benzyl-9-((3a_S_,4_S_,6_R_,6a_S_)-6-((_R_)-2,2-dimethyl-1,3-dioxolan-4-yl)-2,2-dimethyltetrahydrofuro[3,4-_d_][1,3]dioxol-4-yl)-9_H_-purin-6-amine (6d)

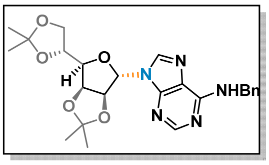

Purified by flash column chromatography (ethyl acetate / petroleum ether = 1:8, v:v); colorless oil; (45 mg, 49%), $[\alpha]_D^{25}$ = -1.1 (c = 0.7, $CHCl_3$); **^{1}H NMR** (400 MHz, $CDCl_3$) δ 8.28 (s, 1H), 7.61 (s, 1H), 7.35–7.19 (m, 5H), 6.33 (s, 1H), 5.88 (s, 1H), 5.47 (d, J = 5.7 Hz, 1H), 5.21 (dd, J = 6.0, 2.9 Hz, 1H), 4.80 (s, 2H), 4.48–4.33 (m, 2H), 3.96-4.00 (m, 1H), 3.88-3.95 (m, 1H), 1.50 (s, 3H), 1.35 (s, 6H), 1.29 (s, 3H); **^{13}C NMR** (101 MHz, $CDCl_3$) δ 154.8, 153.4, 147.6, 140.0, 138.4, 128.8, 127.8, 127.7, 119.2, 113.5, 109.2, 90.6, 84.8, 84.1, 81.4, 73.5, 66.4, 43.6, 26.9, 26.2, 25.2, 24.7; **HRMS (ESI)** m/z cald for $C_{24}H_{30}N_5O_5^+$ $[M+H]^+$ 468.2241, found 468.2239.

***Tert*-butyl (*tert*-butoxycarbonyl)(9-((3a*S*,4*S*,6*R*,6a*S*)-6-((*R*)-2,2-dimethyl-1,3-dioxolan-4-yl)-2,2-dimethyltetrahydrofuro[3,4-*d*][1,3]dioxol-4-yl)-9*H*-purin-6-yl)carbamate (6e)**

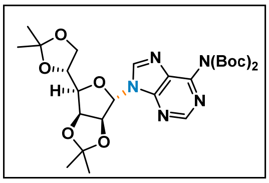

Purified by flash column chromatography (acetatone / petroleum ethe r = 1:20, v:v); colorless oil; (54 mg, 48%), $[\alpha]_D^{25}$ = -7.0 (c = 0.4, $CHCl_3$); **^{1}H NMR** (400 MHz, Acetone-d_6) δ 8.82 (s, 1H), 8.59 (s, 1H), 6.32 (s, 1H), 5.64 (d, J = 5.8 Hz, 1H), 5.32 (dd, J = 5.9, 3.5 Hz, 1H), 4.52 (dd, J = 6.3, 3.6 Hz, 1H), 4.46–4.40 (m, 1H), 4.03 (dd, J = 8.5, 6.4 Hz, 1H), 3.90 (dd, J = 8.5, 5.5 Hz, 1H), 1.53 (s, 3H), 1.45 (s, 18H), 1.39 (s, 3H), 1.29 (s, 3H), 1.27 (s, 3H); **^{13}C NMR** (101 MHz, Acetone-d_6) δ 153.8, 152.6, 151.3, 151.3, 146.4 129.7, 113.7, 109.3, 91.3, 85.6, 85.1, 84.0, 82.0, 74.1, 66.8, 27.9, 26.9, 26.4, 25.5, 24.7; **HRMS (ESI)** m/z cald for $C_{27}H_{40}N_5O_9^+$ $[M+H]^+$ 578.2821, found 578.2829.

***Tert*-butyl (6-chloro-9-((3a*S*,4*S*,6*R*,6a*S*)-6-((*S*)-2,2-dimethyl-1,3-dioxolan-4-yl)-2,2-dimethyltetrahydrofuro[3,4-*d*][1,3]dioxol-4-yl)-9*H*-purin-2-yl)carbamate (6f)**

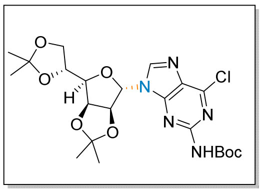

Purified by flash column chromatography (acetatone / DCM = 1:30, v:v); colorless oil; (44 mg, 43%), $[\alpha]_D^{25}$ = -92.8 (c =1.2, $CHCl_3$); **^{1}H NMR** (400 MHz, Acetone-d_6) δ 9.23 (s, 1H), 8.39 (s, 1H), 6.15 (s, 1H), 5.66–5.56 (m, 2H), 4.57 (dd, J = 6.2, 3.5 Hz, 1H), 4.39–4.30 (m, 1H), 3.96 (dd, J = 8.4, 6.4 Hz, 1H), 3.85 (dd, J = 8.4, 5.4 Hz, 1H), 1.49 (s, 9H), 1.46 (s, 3H), 1.34 (s, 3H), 1.26 (s, 3H), 1.22 (s, 3H); **^{13}C NMR** (101 MHz, Acetone-d_6) 153.6, 153.4, 151.3, 151.2, 146.3, 128.7, 113.1, 109.1, 91.1, 85.4, 85.2, 82.1, 81.0, 74.4, 66.7, 28.4, 27.0, 26.3, 25.5, 24.5; **HRMS (ESI)** m/z cald for $C_{22}H_{31}ClN_5O_7^+$ $[M+H]^+$ 512.1907, found 512.1894.

2-acetamido-9-((3a*S*,4*S*,6*R*,6a*S*)-6-((*R*)-2,2-dimethyl-1,3-dioxolan-4-yl)-2,2-dimethyltetrahydrofuro[3,4-*d*][1,3]dioxol-4-yl)-9*H*-purin-6-yl diphenylcarbamate (6g)

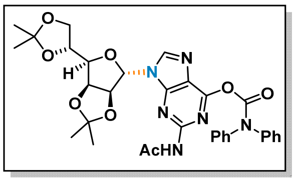

Purified by flash column chromatography (acetatone / DCM = 1:10, v:v); white solid (52 mg, 41%), m.p.: 88-89 °C;, $[\alpha]_D^{25}$ = -92.8 (c =1.2, $CHCl_3$); **^{1}H NMR** (400 MHz, DMSO-d_6) δ 10.84 (s, 1H), 8.55 (s, 1H), 7.53–7.41 (m, 8H), 7.36–7.29 (m, 2H), 6.27 (s, 1H), 5.53 (dd, J = 5.9, 3.8 Hz, 1H), 5.43 (d, J = 5.9 Hz, 1H), 4.56 (dd, J = 5.5, 3.8 Hz, 1H), 4.31 (q, J = 5.9 Hz, 1H), 3.95 (dd, J = 8.4, 6.5 Hz, 1H), 3.83 (dd, J = 8.4, 5.7 Hz, 1H), 2.16 (s, 3H), 1.48 (s, 3H), 1.32 (s, 3H), 1.26 (s, 3H), 1.24 (s, 3H). **^{13}C NMR** (101 MHz, DMSO-d_6) δ 168.2, 155.2, 154.1, 151.9, 150.0, 145.4, 141.5, 129.4, 127.3, 126.9, 120.3, 111.9, 107.8, 88.9, 84.1, 83.6, 80.6, 73.1, 65.1, 26.3, 25.7, 25.0, 24.5, 24.2; **HRMS (ESI)** m/z cald for $C_{32}H_{34}N_6O_8Na^+$ $[M+Na]^+$ 653.2330, found 653.2341.

2-((3a*S*,4*S*,6*R*,6a*S*)-6-((*R*)-2,2-dimethyl-1,3-dioxolan-4-yl)-2,2-dimethyltetrahydrofuro[3,4-*d*][1,3]dioxol-4-yl)isoindoline-1,3-dione (7a)

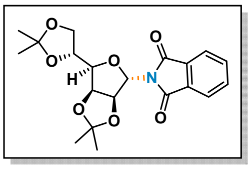

Purified by flash column chromatography (ether / DCM = 1:10, v:v); White solid; (45 mg, 58%), $[\alpha]_D^{25}$ = +23.8 (c = 1.1, $CHCl_3$); mp: 114 °C; **^{1}H NMR** (400 MHz, $CDCl_3$) δ 7.90–7.84 (m, 2H), 7.80–7.73 (m, 2H), 5.91 (s, 1H), 5.26 (d, J = 5.9 Hz, 1H), 5.17 (dd, J = 6.0, 3.8 Hz, 1H), 4.39 (ddd, J = 7.3, 6.1, 4.7 Hz, 1H), 4.29 (dd, J = 7.3, 3.8 Hz, 1H), 4.05 (dd, J = 8.8, 6.1 Hz, 1H), 3.99 (dd, J = 8.8, 4.7 Hz, 1H), 1.54 (s, 3H), 1.44 (s, 3H), 1.38 (s, 3H), 1.36 (s, 3H); **^{13}C**

NMR (101 MHz, $CDCl_3$) δ 168.1, 134.8, 131.9, 124.0, 113.3, 109.3, 84.9, 84.7, 84.2, 81.7, 73.8, 66.8, 27.2, 26.3, 25.5, 24.7; **HRMS (ESI)** m/z cald for $C_{20}H_{24}NO_7^+$ $[M+H]^+$ 390.1547, found 390.1543.

1-((3a*S*,4*S*,6*R*,6a*S*)-6-((*R*)-2,2-dimethyl-1,3-dioxolan-4-yl)-2,2-dimethyltetrahydrofuro[3,4-*d*][1,3]dioxol-4-yl)azetidin-2-one (7b)

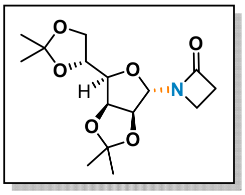

Purified by flash column chromatography (ether / DCM = 1:10, v:v); Colorless oil; (38 mg, 62%), $[\alpha]_D^{25}$ = +4.3 (*c* = 1, $CHCl_3$); **^{1}H NMR** (400 MHz, $CDCl_3$) δ 5.32 (d, *J* = 5.9 Hz, 1H), 4.86 (dd, *J* = 5.9, 3.7 Hz, 1H), 4.70 (s, 1H), 4.33 (td, *J* = 6.5, 4.7 Hz, 1H), 4.04–3.94 (m, 2H), 3.88 (dd, *J* = 6.9, 3.6 Hz, 1H), 3.32 (td, *J* = 5.6, 3.1 Hz, 1H), 3.17 (td, *J* = 5.5, 3.0 Hz, 1H), 2.95-2.80 (m, 2H), 1.40 (s, 3H), 1.39 (s, 3H), 1.30 (s, 3H), 1.27 (s, 3H). **^{13}C NMR** (101 MHz, $CDCl_3$) δ 166.6, 111.9, 108.2, 88.2, 81.9, 80.9, 79.3, 72.4, 65.7, 37.2, 35.6, 26.0, 25.0, 24.2, 23.6; **HRMS (ESI)** m/z cald for $C_{15}H_{23}NO_6Na^+$ $[M+Na]^+$ 336.1418, found 336.1423.

2-((3a*S*,4*S*,6*R*,6a*S*)-6-((*R*)-2,2-dimethyl-1,3-dioxolan-4-yl)-2,2-dimethyltetrahydrofuro[3,4-*d*][1,3]dioxol-4-yl)isoquinolin-1(2*H*)-one (7c)

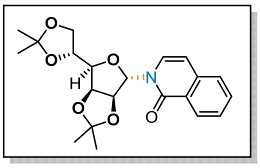

Purified by flash column chromatography (ethyl acetate/ petroleum ether = 1:12, v:v); Colorless oil; (36 mg, 47%), $[\alpha]_D^{25}$ = +28.2 (*c* = 0.5, $CHCl_3$); **^{1}H NMR** (400 MHz, $CDCl_3$) δ 8.17 (d, *J* = 8.3 Hz, 1H), 8.05 (d, *J* = 5.8 Hz, 1H), 7.80 – 7.73 (m, 1H), 7.72–7.63 (m, 1H), 7.60–7.51 (m, 1H), 7.24-7.31 (m, 1H), 6.70 (s, 1H), 5.06–4.97 (m, 2H), 4.43-4.55 (m, 1H), 4.22 (dd, *J* = 8.2, 3.3 Hz, 1H), 4.12 (dd, *J* = 8.9, 6.0 Hz, 1H), 4.04 (dd, *J* = 8.8, 4.1 Hz, 1H), 1.56 (s, 3H), 1.46 (s, 3H), 1.40 (s, 3H), 1.38 (s, 3H); **^{13}C NMR** (101 MHz, $CDCl_3$) δ 157.4, 138.7, 137.1, 129.6, 125.7, 125.3, 122.8, 118.4, 114.8, 112.2, 108.3, 101.6, 84.5, 81.3, 78.6, 72.0, 66.1, 26.0, 25.1, 24.1, 23.7; **HRMS (ESI)** m/z cald for $C_{21}H_{26}NO_6^+$ $[M+H]^+$ 388.1755, found 388.1765.

3-((3a*S*,4*S*,6*R*,6a*S*)-6-((*R*)-2,2-dimethyl-1,3-dioxolan-4-yl)-2,2-dimethyltetrahydrofuro[3,4-*d*][1,3]dioxol-4-yl)oxazolidin-2-one (7d)

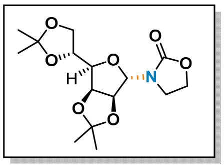

Purified by flash column chromatography (ethyl acetate/petroleum ether = 1:8, v:v); colorless oil; (54 mg, 41%), $[\alpha]_D^{25}$ = +23.9 (*c* = 0.7, $CHCl_3$); **^{1}H NMR** (400 MHz, $CDCl_3$) δ 5.38 (d, *J* = 5.9 Hz, 1H), 5.00 (d, *J* = 5.8 Hz, 2H), 4.43–4.29 (m, 3H), 4.21 (dd, *J* = 6.7, 3.7 Hz, 1H), 4.10–3.98 (m, 2H), 3.82 (q, *J* = 8.7 Hz, 1H), 3.63–3.52 (m, 1H), 1.48 (s, 3H), 1.44 (s, 3H), 1.36 (s, 3H),

1.34 (s, 3H); **^{13}C NMR** (101 MHz, $CDCl_3$) δ 157.9, 112.8, 109.2, 91.0, 83.7, 83.5, 80.9, 73.7, 66.5, 62.5, 45.3, 26.9, 26.1, 25.2, 24.5; **HRMS (ESI)** m/z cald for $C_{15}H_{23}NO_7Na^+$ $[M+Na]^+$ 352.1367, found 352.1364.

((3a*R*,4*R*,6*R*,6a*R*)-6-(3-chloro-1*H*-indazol-1-yl)-2,2-dimethyltetrahydrofuro[3,4-*d*][1,3]dioxol-4-yl)methyl benzoate (8a)

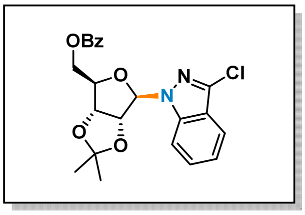

Purified by flash column chromatography (acetone / petroleum ether = 1:25, v:v); colorless oil; (61 mg, 70%), $[\alpha]_D^{25}$ = +68.4 (*c* = 0.9, $CHCl_3$); **^{1}H NMR** (400 MHz, $CDCl_3$) δ 8.01–7.94 (m, 2H), 7.67 (d, *J* = 8.2 Hz, 1H), 7.58–7.50 (m, 2H), 7.49–7.37 (m, 3H), 7.27–7.22 (m, 1H), 6.34 (d, *J* = 1.2 Hz, 1H), 5.62 (dd, *J* = 6.1, 1.2 Hz, 1H), 5.12 (dd, *J* = 6.0, 2.5 Hz, 1H), 4.58 (td, *J* = 6.3, 2.5 Hz, 1H), 4.35 (dd, *J* = 11.6, 6.4 Hz, 1H), 4.23 (dd, *J* = 11.6, 6.2 Hz, 1H), 1.63 (s, 3H), 1.43 (s, 3H); **^{13}C NMR** (101 MHz, $CDCl_3$) *δ* 166.2, 141.6, 135.9, 133.2, 129.9, 129.7, 128.6, 128.5, 122.5, 122.0, 120.1, 113.8, 110.0, 91.5, 85.3, 84.4, 82.4, 64.4, 27.1, 25.4; **HRMS (ESI)** m/z cald for $C_{22}H_{22}ClN_2O_5^+$ $[M+H]^+$ 429.1212, found 429.1210.

1-((3a*R*,4*R*,6*R*,6a*R*)-6-((benzyloxy)methyl)-2,2-dimethyltetrahydrofuro[3,4-*d*][1,3]dioxol-4-yl)-3-chloro-1*H*-indazole (8b)

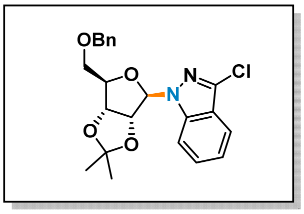

Purified by flash column chromatography (ethyl acetate/ petroleum ether = 1:30, v:v); colorless oil; (83 mg, 67%), $[\alpha]_D^{25}$ = -78.4 (*c* =2.6, $CHCl_3$); **^{1}H NMR** (400 MHz, $CDCl_3$) δ 7.65 (dd, *J* = 8.2, 1.0 Hz, 1H), 7.55 (d, *J* = 8.5 Hz, 1H), 7.39-7.47 (m, 1H), 7.13–7.28 (m, 6H), 6.28 (d, *J* = 1.3 Hz, 1H), 5.53 (dd, *J* = 6.1, 1.3 Hz, 1H), 4.98 (dd, *J* = 6.1, 2.5 Hz, 1H), 4.44 (td, *J* = 6.5, 2.4 Hz, 1H), 4.37 (d, *J* = 1.7 Hz, 2H), 3.47 (dd, *J* = 10.1, 6.3 Hz, 1H), 3.34 (dd, *J* = 10.1, 6.8 Hz, 1H), 1.61 (s, 3H), 1.40 (s, 3H); **^{13}C NMR** (101 MHz, $CDCl_3$) δ 141.5, 137.8, 135.2, 128.4, 128.4, 127.7, 127.7, 122.3, 121.9, 119.9, 113.5, 110.2, 91.6, 86.5, 84.1, 82.5, 73.4, 70.3, 27.1, 25.4; **HRMS (ESI)** m/z cald for $C_{22}H_{24}ClN_2O_4^+$ $[M+H]^+$ 415.1419, found 415.1425.

3-Chloro-1-((3a*R*,4*R*,6*R*,6a*R*)-2,2-dimethyl-6-((trityloxy)methyl)tetrahydrofuro[3,4-*d*][1,3]dioxol-4-yl)-1*H*-indazole (8c)

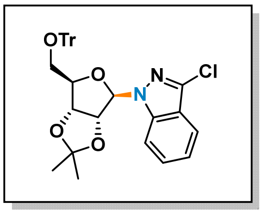

Purified by flash column chromatography (diethyl ether/petroleum ether = 1:30, v:v); colorless oil; (84 mg, 74%), $[\alpha]_D^{25}$ = -24.9 (c = 0.8, $CHCl_3$); **^{1}H NMR** (400 MHz, DMSO-d_6) δ 7.98 (d, J = 8.6 Hz, 1H), 7.66 (d, J = 8.1 Hz, 1H), 7.58–7.52 (m, 1H), 7.33 (t, J = 7.5 Hz, 1H), 7.24–7.13 (m, 15H), 6.59 (d, J = 1.2 Hz, 1H), 5.38 (dd, J = 6.0, 1.2 Hz, 1H), 4.83 (dd, J = 6.0, 2.5 Hz, 1H), 4.31 (td, J = 6.0, 2.4 Hz, 1H), 3.00–2.89 (m, 2H), 1.53 (s, 3H), 1.32 (s, 3H). **^{13}C NMR** (101 MHz, DMSO-d_6) δ 144.4, 142.1, 134.8, 129.5, 129.0, 128.7, 127.9, 123.6, 121.9, 120.2, 113.6, 112.0, 91.4, 87.2, 86.9, 84.4, 82.7, 65.3, 27.8, 26.0; **HRMS (ESI)** m/z cald for $C_{34}H_{31}ClN_2O_4Na^+$ $[M+Na]^+$ 589.1865, found 589.1877.

3-chloro-1-((3a*R*,4*R*,6*R*,6a*R*)-6-((methoxymethoxy)methyl)-2,2-dimethyltetrahydrofuro[3,4-*d*][1,3]dioxol-4-yl)-1*H*-indazole (8d)

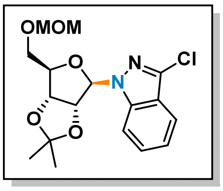

Purified by flash column chromatography (acetone/petroleum ether = 1:35, v:v); colorless oil; (55 mg, 71%), $[\alpha]_D^{25}$ = +68.1(c =3.8, $CHCl_3$); **^{1}H NMR** (400 MHz, $CDCl_3$) δ 7.67–7.62 (m, 1H), 7.55–7.51 (m, 1H), 7.48–7.41 (m, 1H), 7.27–7.19 (m, 1H), 6.30 (d, J = 1.2 Hz, 1H), 5.55 (d, J = 1.2 Hz, 1H), 4.99 (dd, J = 6.1, 2.4 Hz, 1H), 4.49 (d, J = 6.6 Hz, 1H), 4.45–4.38 (m, 2H), 3.51-3.39 (m, 2H), 3.16 (s, 3H), 1.61 (s, 3H), 1.41 (s, 3H); **^{13}C NMR** (101 MHz, $CDCl_3$) δ 141.5, 135.3, 128.3, 122.3, 121.9, 119.9, 113.5, 110.1, 96.6, 91.5, 86.5, 84.2, 82.5, 67.9, 55.1, 27.0, 25.3. **HRMS (ESI)** m/z cald for $C_{17}H_{21}ClN_2NaO_5^+$ $[M+Na]^+$ 391.1031, found 391.1041.

1-((3a*R*,4*R*,6*R*,6a*R*)-6-(((*tert*-butyldimethylsilyl)oxy)methyl)-2,2-dimethyltetrahydrofuro[3,4-*d*][1,3]dioxol-4-yl)-3-chloro-1*H*-indazole (8e)

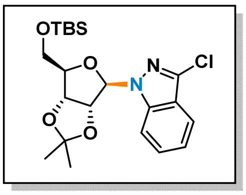

Purified by flash column chromatography (diethyl ether/ petroleum ether = 1:50, v:v); Colorless oil; (57 mg, 65%), $[\alpha]_D^{25}$ = +42.9 (c =0.5, $CHCl_3$); **^{1}H NMR** (400 MHz, $CDCl_3$) δ 7.70–7.64 (m, 1H), 7.55 (d, J = 8.5 Hz, 1H), 7.50–7.44 (m, 1H), 7.28–7.23 (m, 1H), 6.28 (d, J = 1.3 Hz, 1H), 5.56 (dd, J = 6.1, 1.3 Hz, 1H), 4.99 (dd, J = 6.1, 2.1 Hz, 1H), 4.30 (ddd, J = 7.8, 6.2, 2.1 Hz, 1H), 3.55 (dd, J = 10.5, 7.4 Hz, 1H), 3.43 (dd, J = 10.5, 6.2 Hz, 1H), 1.61 (s, 3H), 1.42 (s, 3H), 0.81 (s, 9H), -0.07 (s, 3H), -0.09 (s, 3H); **^{13}C NMR** (101 MHz, $CDCl_3$) δ 141.6, 135.2, 128.4, 122.4, 122.0, 119.9, 113.3, 110.2, 91.8, 88.3, 84.1, 82.4, 63.4, 27.1, 26.0, 25.4, 18.5, -5.4, -5.4; **HRMS (ESI)** m/z cald for $C_{22}H_{32}ClN_2O_4Si^+$ $[M+H]^+$ 439.1814, found 439.1828.

1-((2*R*,3*R*,4*R*,5*R*)-3,4-bis(benzyloxy)-5-((benzyloxy)methyl)tetrahydrofuran-2-yl)-3-chloro-1*H*-indazole (8f)

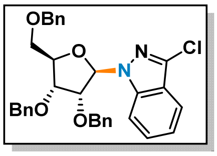

Purified by flash column chromatography (ethyl acetate/ petroleum ether = 1:32, v:v); colorless oil; (70 mg, 63%), $[\alpha]_D^{25}$ = -40.3 (c = 1.9, $CHCl_3$); **^{1}H NMR** (400 MHz, $CDCl_3$) δ 7.56 (d, J = 8.1 Hz, 1H), 7.38 (d, J = 8.5 Hz, 1H), 7.32–7.17 (m, 11H), 7.13 (td, J = 6.3, 2.4 Hz, 6H), 6.12 (d, J = 4.7 Hz, 1H), 4.73 (t, J = 5.0 Hz, 1H), 4.64–4.54 (m, 2H), 4.54–4.34 (m, 4H), 4.32 (q, J = 4.7 Hz, 1H), 4.24 (t, J = 4.9 Hz, 1H), 3.55 (dd, J = 10.6, 4.4 Hz, 1H), 3.48 (dd, J = 10.6, 5.0 Hz, 1H); **^{13}C NMR** (101 MHz, $CDCl_3$) δ 141.8, 138.1, 137.8, 137.6, 135.4, 128.6, 128.5, 128.4, 128.3, 128.1, 128.0, 128.0, 128.0, 127.8, 127.7, 127.7, 122.2, 122.0, 119.8, 110.1, 89.1, 81.9, 79.6, 77.4, 73.5, 73.0, 72.6, 70.4; **HRMS (ESI)** m/z cald for $C_{33}H_{32}ClN_2O_4^+$ $[M+H]^+$ 555.2045, found 555.2048.

(2*R*,3*R*,4*R*,5*R*)-2-((benzoyloxy)methyl)-5-(3-chloro-1*H*-indazol-1-yl) tetrahydrofuran-3,4-diyl dibenzoate (8g)

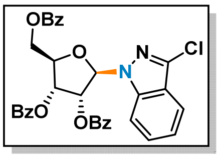

Purified by flash column chromatography (DCM / petroleum ether = 1:1.5, v:v); colorless oil; (41 mg, 35%), $[\alpha]_D^{25}$ = -55.7 (c = 0.9, $CHCl_3$); **^{1}H NMR** (400 MHz, $CDCl_3$) δ 8.01 (d, J = 7.1 Hz, 2H), 7.93 (dd, J = 8.1, 1.4 Hz, 2H), 7.80–7.75 (m, 2H), 7.65 (d, J = 8.1 Hz, 1H), 7.62–7.53 (m, 2H), 7.53–7.39 (m, 5H), 7.32 (t, J = 7.7 Hz, 2H), 7.26–7.18 (m, 3H), 6.48 (t, J = 3.3 Hz, 1H), 6.32 (d, J = 6.6 Hz, 1H), 6.17 (dd, J = 6.6, 3.4 Hz, 1H), 5.70 (ddd, J = 7.1, 5.1, 3.2 Hz, 1H), 4.25–4.18 (m, 2H); **^{13}C NMR** (101 MHz, $CDCl_3$) δ 165.6, 165.3, 165.1, 141.2, 136.0, 133.6, 133.5, 133.4, 130.0, 130.0, 129.9, 129.6, 129.4, 129.1, 128.8, 128.6, 128.6, 128.4, 122.7, 122.5, 120.3, 110.3, 83.9, 68.5, 68.4, 67.2, 63.9; **HRMS (ESI)** m/z cald for $C_{33}H_{25}ClN_2O_7Na^+$ $[M+Na]^+$ 619.1242, found 619.1248.

1-((2*S*,3*S*,4*S*,5*S*)-3,4-bis(benzyloxy)-5-((benzyloxy)methyl)tetrahydrofuran-2-yl)-3-chloro-1*H*-indazole (8h)

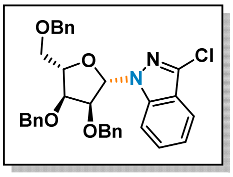

Purified by flash column chromatography (ethyl acetate/ petroleum ether = 1:30, v:v); Colorless oil; (69 mg, 61%), $[\alpha]_D^{25}$= +38.9 (c =2.9, $CHCl_3$); **^{1}H NMR** (400 MHz, $CDCl_3$) ^{1}H NMR (400 MHz, $CDCl_3$) δ 7.56 (d, J = 8.1 Hz, 1H), 7.38 (d, J = 8.5 Hz, 1H), 7.31–7.17 (m, 11H), 7.15–7.10 (m, 6H), 6.13 (d, J = 4.7 Hz, 1H), 4.74 (t, J = 4.9 Hz, 1H), 4.64–4.54 (m, 2H), 4.54–4.35 (m, 4H), 4.32 (q, J = 4.7 Hz, 1H), 4.25 (t, J = 4.9 Hz, 1H), 3.55 (dd, J = 10.7, 4.4 Hz, 1H), 3.48 (dd, J = 10.6, 5.1 Hz, 1H); **^{13}C NMR** (101 MHz, $CDCl_3$) δ 141.8, 138.1, 137.8, 137.6, 135.4, 128.5,

128.4, 128.4, 128.3, 128.1, 128.0, 128.0, 128.0, 127.7, 127.6, 122.2, 122.0, 119.8, 110.1, 89.1, 81.9, 79.6, 77.4, 73.5, 73.0, 72.6, 70.4; **HRMS (ESI)** m/z cald for $NaC_{33}H_{31}ClN_2O_4^+$ $[M+Na]^+$ 577.1865, found 577.1863.

1-((2*S*,3*S*,4*R*,5*R*)-3,4-bis(benzyloxy)-5-((benzyloxy)methyl)tetrahydrofuran-2-yl)-3-chloro-1*H*-indazole (8i)

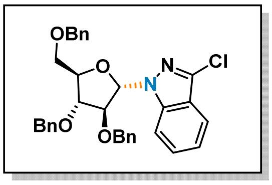

Purified by flash column chromatography (ethyl acetate/ petroleum ether = 1:30, v:v); White solid; (66 mg, 60%), mp: 58 °C; $[\alpha]_D^{25}$ = +44.2(*c* =0.7, $CHCl_3$); **^{1}H NMR** (400 MHz, $CDCl_3$) δ 7.59 (d, *J* = 8.1 Hz, 1H), 7.39–7.30 (m, 3H), 7.28–7.19 (m, 10H), 7.16–7.04 (m, 5H), 6.06 (d, *J* = 4.6 Hz, 1H), 5.14 (t, *J* = 5.0 Hz, 1H), 4.63 (d, *J* = 11.7 Hz, 1H), 4.55–4.49 (m, 2H), 4.47–4.34 (m, 3H), 4.34–4.31 (m, 1H), 4.27 (dd, *J* = 7.6, 5.5 Hz, 1H), 3.61 (dd, *J* = 11.0, 3.0 Hz, 1H), 3.53 (dd, *J* = 11.0, 4.2 Hz, 1H); **^{13}C NMR** (101 MHz, $CDCl_3$) δ 141.4, 138.1, 138.0, 137.4, 135.5, 128.5, 128.4, 128.1, 128.0, 128.0, 127.9, 127.9, 127.8, 122.2, 122.2, 119.9, 110.2, 90.3, 86.5, 82.7, 81.0, 73.6, 73.0, 72.6, 69.3; **HRMS (ESI)** m/z cald for $NaC_{33}H_{31}ClN_2O_4^+$ $[M+Na]^+$ 577.1865, found 577.1879.

1-((2*S*,3*S*,4*R*,5*R*)-4-(benzyloxy)-5-((benzyloxy)methyl)-3-fluorotetrahydrofuran-2-yl)-3-chloro-1*H*-indazole (8j)

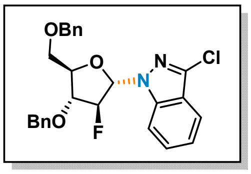

Purified by flash column chromatography (ethyl acetate/ petroleum ether = 1:35, v:v); Colorless oil; (56 mg, 60%), $[\alpha]_D^{25}$= +103.1(*c* =1.6, $CHCl_3$); **^{1}H NMR** (400 MHz, $CDCl_3$) δ 7.60 (d, *J* = 8.1 Hz, 1H), 7.44 (d, *J* = 8.5 Hz, 1H), 7.41–7.33 (m, 1H), 7.14-7.30 (m, 11H), 6.23 (dd, *J* = 16.3, 3.3 Hz, 1H), 6.08 (dt, *J* = 53.2, 3.6 Hz, 1H), 4.73 (d, *J* = 11.8 Hz, 1H), 4.57–4.46 (m, 2H), 4.46–4.29 (m, 3H), 3.65–3.57 (m, 1H), 3.53 (dd, *J* = 11.1, 3.7 Hz, 1H); **^{13}C NMR** (101 MHz, $CDCl_3$) δ 141.7, 137.9, 137.3, 136.0, 128.6, 128.6, 128.4, 128.1, 128.1, 127.9, 127.8, 122.4, 122.3, 120.1, 110.1, 99.5 (d, *J* = 187.0 Hz), 89.3 (d, *J* = 36.0 Hz), 81.9 (d, *J* = 23.2 Hz), 81.6 (d, *J* = 7.1 Hz), 73.7, 72.7, 68.7; **^{19}F NMR** (376 MHz, $CDCl_3$) δ -187.44 – -190.67 (m, 1F); **^{19}F {^{1}H} NMR** (376 MHz, $CDCl_3$) δ -189.51 (s, 1F); **HRMS (ESI)** m/z cald for $C_{26}H_{24}ClFN_2O_3Na^+$ $[M+Na]^+$ 489.1352, found 489.1356.

1-((2*R*,4*S*,5*R*)-4-(benzyloxy)-5-((benzyloxy)methyl)tetrahydrofuran-2-yl)-3-chloro-1*H*-indazole (8ka)

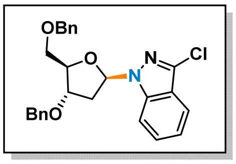

Purified by flash column chromatography (ethyl acetate/ petroleum ether = 1:30, v:v); colorless oil; (30.7 mg, 34%), $[\alpha]_D^{25}$ = -32.2 (c =0.4, $CHCl_3$); **^{1}H NMR** (400 MHz, $CDCl_3$) δ 7.60–7.53 (m, 1H), 7.49 (d, J = 8.6 Hz, 1H), 7.37–7.28 (m, 1H), 7.30–7.25 (m, 3H), 7.27–7.10 (m, 8H), 6.38 (t, J = 6.3 Hz, 1H), 4.58–4.46 (m, 2H), 4.39 (s, 2H), 4.38–4.34 (m, 1H), 4.27 (td, J = 5.7, 3.0 Hz, 1H), 3.48 (dd, J = 10.2, 5.8 Hz, 1H), 3.42 (dd, J = 10.3, 5.7 Hz, 1H), 3.15–3.04 (m, 1H), 2.41 (ddd, J = 13.7, 6.5, 3.5 Hz, 1H); **^{13}C NMR** (101 MHz, $CDCl_3$) δ 141.5, 138.2, 138.0, 134.8, 128.6, 128.5, 128.1, 128.0, 128.0, 127.8, 127.7, 122.2, 122.1, 119.9, 110.2, 87.0, 83.8, 80.1, 73.53, 71.8, 70.9, 36.2; **HRMS (ESI)** m/z cald for $C_{26}H_{26}ClN_2O_3^+$ $[M+H]^+$ 449.1626, found 449.1610.

1-((2*S*,4*S*,5*R*)-4-(benzyloxy)-5-((benzyloxy)methyl)tetrahydrofuran-2-yl)-3-chloro-1*H*-indazole (8kb)

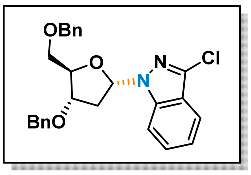

Purified by flash column chromatography (ethyl acetate/ petroleum ether = 1:30, v:v); colorless oil; (26.0 mg, 29%); **^{1}H NMR** (400 MHz, $CDCl_3$) δ 7.66–7.55 (m, 2H), 7.35–7.30 (m, 1H), 7.30–7.20 (m, 10H), 7.16–7.12 (m, 1H), 6.32 (dd, J = 7.2, 5.2 Hz, 1H), 4.60–4.50 (m, 2H), 4.49–4.39 (m, 2H), 4.34–4.29 (m, 1H), 4.28–4.20 (m, 1H), 3.62 (dd, J = 10.7, 3.0 Hz, 1H), 3.53 (dd, J = 10.7, 3.8 Hz, 1H), 3.09–3.00 (m, 1H), 2.72–2.60 (m, 1H); **^{13}C NMR** (101 MHz, $CDCl_3$) δ 139.8, 137.0, 136.8, 133.2, 127.4, 127.4, 126.8, 126.7, 126.7, 126.7, 126.6, 121.2, 120.8, 118.7, 109.7, 86.8, 81.8, 77.5, 72.5, 70.9, 68.9, 35.0.

3-chloro-1-((3a*S*,4*S*,6*R*,6a*S*)-2,2-dimethyl-6-vinyltetrahydrofuro[3,4-*d*][1,3]dioxol-4-yl)-1*H*-indazole (8l)

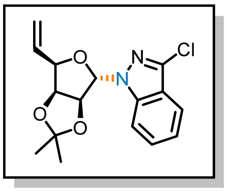

Purified by flash column chromatography (MeOH/toluene/ petroleum ether = 1:20:50, v:v:v); white solid; (52 mg, 81%), mp: 86 °C; $[\alpha]_D^{25}$ = +34.6 (c = 2.5, $CHCl_3$); **^{1}H NMR** (400 MHz, $CDCl_3$) δ 7.68 (d, J = 8.2 Hz, 1H), 7.55 (d, J = 8.5 Hz, 1H), 7.47 (ddd, J = 8.4, 6.8, 1.1 Hz, 1H), 7.30–7.21 (m, 1H), 6.24 (s, 1H), 6.03-5.93 (m, 1H), 5.57 (d, J = 5.8 Hz, 1H), 5.38–5.28 (m, 2H), 5.09 (dd, J = 5.8, 3.9 Hz, 1H), 4.57 (dd, J = 7.5, 3.9 Hz, 1H), 1.60 (s, 3H), 1.41 (s, 3H); **^{13}C NMR** (101 MHz, $CDCl_3$) δ 141.5, 135.3, 132.0, 128.5, 122.4, 121.8, 119.9, 119.8, 113.0, 110.0, 89.8, 84.8, 83.9, 82.4, 26.3, 24.9; **HRMS (ESI)** m/z cald for $C_{16}H_{17}ClN_2O_3Na^+$ $[M+Na]^+$ 343.0820, found 343.0820.

3-chloro-1-((2*S*,3*S*,4*S*,5*R*,6*R*)-3,4,5-tris(benzyloxy)-6-((benzyloxy)methyl)tetrahydro-2*H*-pyran-2-yl)-1*H*-indazole (8m)

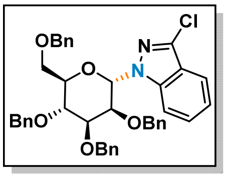

Purified by flash column chromatography (acetone/petroleum ether = 1:50, v:v); colorless oil; (92 mg, 68%), $[\alpha]_D^{25}$= +53.9 (*c* = 2.9, $CHCl_3$); **^{1}H NMR** (400 MHz, $CDCl_3$) δ 7.58 (d, *J* = 8.6 Hz, 2H), 7.43–7.36 (m, 2H), 7.34–7.23 (m, 6H), 7.25–7.11 (m, 12H), 7.13–7.04 (m, 2H), 6.02 (d, *J* = 3.1 Hz, 1H), 4.78–4.65 (m, 5H), 4.64–4.46 (m, 2H), 4.45–4.33 (m, 3H), 4.01 (dd, *J* = 9.1, 7.9 Hz, 1H), 3.68 (dd, *J* = 11.0, 5.1 Hz, 1H), 3.60–3.50 (m, 2H); **^{13}C NMR** (101 MHz, $CDCl_3$) δ 141.5, 138.4, 138.3, 138.2, 138.1, 134.3, 128.5, 128.5, 128.4, 128.3, 128.1, 128.0, 128.0, 127.9, 127.8, 127.8, 127.7, 127.6, 122.3, 121.9, 119.7, 111.3, 84.2, 78.8, 74.9, 74.5, 74.5, 73.9, 73.4, 73.3, 72.6, 69.1; **HRMS (ESI)** m/z cald for $C_{41}H_{39}ClN_2O_5Na^+$ $[M+Na]^+$ 697.2440, found 697.2432.

3-chloro-1-((2*R*,3*R*,4*S*,5*S*,6*R*)-3,4,5-trimethoxy-6-(methoxymethyl)tetrahydro-2*H*-pyran-2-yl)-1*H*-indazole (8n)

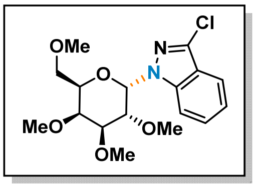

Purified by flash column chromatography (acetone / petroleum ether = 1:50, v:v); colorless oil; (49 mg, 66%), $[\alpha]_D^{25}$= +57.2 (*c* =2.7, $CHCl_3$); **^{1}H NMR** (400 MHz, $CDCl_3$) δ 7.70–7.62 (m, 1H), 7.56 (d, *J* = 8.5 Hz, 1H), 7.49–7.39 (m, 1H), 7.27–7.19 (m, 1H), 6.11 (d, *J* = 4.6 Hz, 1H), 4.88 (t, *J* = 4.9 Hz, 1H), 4.23 (dd, *J* = 7.3, 2.8 Hz, 1H), 4.07 (dd, *J* = 7.2, 5.2 Hz, 1H), 3.59–3.44 (m, 9H), 3.32 (s, 3H), 3.31 (s, 3H); **^{13}C NMR** (101 MHz, $CDCl_3$) δ 141.3, 135.4, 128.1, 122.2, 122.2, 119.9, 110.2, 90.3, 87.9, 84.8, 81.7, 79.6, 72.5, 59.7, 59.6, 59.2, 58.3; **HRMS (ESI)** m/z cald for $C_{17}H_{24}ClN_2O_5^+$ $[M+H]^+$ 371.1368, found 371.1364.

3-Chloro-1-((2*R*,3*R*,4*R*,5*S*,6*S*)-3,4,5-trimethoxy-6-methyltetrahydro-2*H*-pyran-2-yl)-1*H*-indazole (8o)

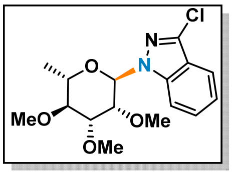

Purified by flash column chromatography (acetone / petroleum ether = 1:50, v:v); White solid; (47 mg, 70%), mp: 69 °C; $[\alpha]_D^{25}$= -66.5 (c =2.8, $CHCl_3$); **^{1}H NMR** (400 MHz, $CDCl_3$) δ 7.70–7.59 (m, 2H), 7.49–7.40 (m, 1H), 7.28–7.20 (m, 1H), 6.01 (d, *J* = 2.7 Hz, 1H), 4.53 (t, *J* = 3.1 Hz, 1H), 4.12 (dd, *J* = 8.0, 3.4 Hz, 1H), 3.63 (s, 3H), 3.55 (s, 3H), 3.54 (s, 3H), 3.35–3.26 (m, 1H), 3.24 (dd, *J* = 9.0, 8.0 Hz, 1H), 1.23 (d, *J* = 6.1 Hz, 3H); **^{13}C NMR** (101 MHz, $CDCl_3$) δ 141.5, 134.3, 128.2, 122.3, 121.9, 119.8, 111.0, 83.1, 82.1, 80.7, 76.6, 70.1, 60.5, 59.5, 58.1, 18.0; **HRMS (ESI)** m/z cald for $C_{16}H_{22}ClN_2O_4^+$ $[M+H]^+$ 341.1263, found 341.1263.

3-chloro-1-((2*S*,3*R*,4*S*,5*R*,6*R*)-3,4,5-trimethoxy-6-(methoxymethyl)tetrahydro-2*H*-pyran-2-yl)-1*H*-indazole (8pa)

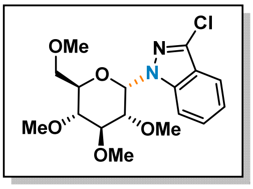

Purified by flash column chromatography (acetone/petroleum ether = 1:25, v:v); white solid; (19 mg, 26%), mp: 159 ºC; $[\alpha]_D^{25}$ = +98.5 (c = 1.2, $CHCl_3$); **^{1}H NMR** (400 MHz, $CDCl_3$) δ 7.68 (d, *J* = 8.1 Hz, 1H), 7.46 (d, *J* = 3.8 Hz, 2H), 7.27–7.21 (m, 1H), 6.30 (d, *J* = 6.0 Hz, 1H), 4.60 (t, *J* = 9.2 Hz, 1H), 3.82–3.75 (m, 2H), 3.73 (s, 3H), 3.60 (s, 3H), 3.51 (dd, *J* = 10.6, 3.1 Hz, 1H), 3.44 (dd, *J* = 10.2, 8.9 Hz, 1H), 3.40–3.35 (m, 4H), 3.34 (s, 3H); **^{13}C NMR** (101 MHz, $CDCl_3$) δ 142.3, 135.1, 128.1, 122.1, 121.6, 120.0, 109.5, 83.6, 81.4, 80.1, 79.5, 72.8, 70.7, 61.0, 60.5, 59.7, 59.2.

3-chloro-1-((2*R*,3*R*,4*S*,5*R*,6*R*)-3,4,5-trimethoxy-6-(methoxymethyl)tetrahydro-2*H*-pyran-2-yl)-1*H*-indazole (8pb)

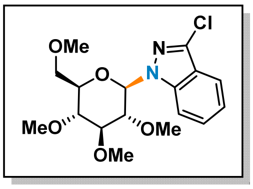

Purified by flash column chromatography (acetone/petroleum ether = 1:40, v:v); white solid; (31 mg, 42%), mp: 137 ºC; $[\alpha]_D^{25}$ = -5.8 (c = 1.3, $CHCl_3$); **^{1}H NMR** (400 MHz, $CDCl_3$) δ 7.70–7.62 (m, 1H), 7.50–7.41 (m, 2H), 7.29–7.20 (m, 1H), 5.38 (d, *J* = 8.9 Hz, 1H), 4.02 (t, *J* = 9.0 Hz, 1H), 3.68 (s, 3H), 3.63–3.53 (m, 6H), 3.39 (t, *J* = 9.1 Hz, 1H), 3.35 – 3.27 (m, 4H), 3.14 (s, 3H); **^{13}C NMR** (101 MHz, $CDCl_3$) δ 140.58, 134.26, 127.07, 121.10, 120.92, 118.82, 108.76, 86.76, 84.94, 80.04, 78.22, 76.50, 70.23, 59.94, 59.62, 59.24, 58.30; **HRMS (ESI)** m/z cald for $C_{17}H_{24}ClN_2O_5^+$ $[M+H]^+$ 371.1368, found 371.1368.

4-(3-(4-(cyclopropanecarbonyl)piperazine-1-carbonyl)-4-fluorobenzyl)-2-((3a*S*,4*S*,6*R*,6a*S*)-6-((*R*)-2,2-dimethyl-1,3-dioxolan-4-yl)-2,2-dimethyltetrahydrofuro[3,4-*d*][1,3]dioxol-4-yl)phthalazin-1(2*H*)-one (9a)

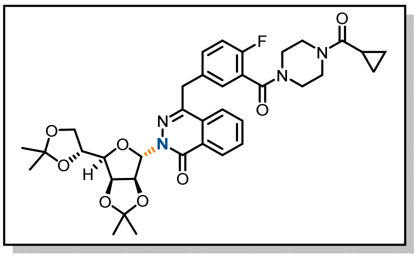

Purified by flash column chromatography (acetone/ petroleum ether = 1 : 4, v:v); colorless oil (61 mg, 45%); $[\alpha]_D^{25}$ = +39.8 (*c* = 0.9, $CHCl_3$); **^{1}H NMR** (400 MHz, DMSO-d_6) δ 8.27–8.19 (m, 2H), 8.04–7.94 (m, 2H), 7.49-7.45 (m, 1H), 7.39 (s, 1H), 7.26-7.20 (m, 1H), 6.64 (s, 1H), 5.00 (d, *J* = 5.9 Hz, 1H), 4.94 (dd, *J* = 6.0, 2.6 Hz, 1H), 4.63 (s, 2H), 4.40–4.31 (m, 2H), 4.08–4.00 (m, 1H), 3.91–3.83 (m, 1H), 3.78–3.47 (m, 5H), 3.40–3.34 (m, 1H), 3.12-3.12 (m, 2H), 2.34-1.85 (m, 1H), 1.46 (s, 3H), 1.34 (s, 3H), 1.31 (s, 3H), 1.28 (s, 3H), 0.77–0.69 (m,

4H); ^{13}**C NMR** (101 MHz, DMSO-d_6) δ 172.3, 165.0, 159.0, 158.5, 156.5, 156.1, 136.3 (d, J = 3.2 Hz), 134.1, 133.5, 132.7 (d, J = 8.2 Hz), 129.8, 128.3, 125.8, 124.6, 124.5, 124.1, 120.1, 116.9 (d, J = 21.8 Hz), 113.0, 109.1, 104.0, 85.6, 83.1, 79.8, 73.5, 66.7, 38.1, 27.5, 26.7, 26.1, 25.3, 11.3, 8.1; ^{19}**F NMR** (376 MHz, DMSO-d_6) δ -119.67 (s, 1F); ^{19}**F {^{1}H} NMR** (376 MHz, DMSO-d_6) δ -119.65 (s, 1F)； **HRMS (ESI)** m/z cald for $C_{36}H_{41}FN_4O_8Na^+$ [M+Na]$^+$ 699.2801, found 699.2791.

3-((3a*S*,4*S*,6*R*,6a*S*)-6-((*R*)-2,2-dimethyl-1,3-dioxolan-4-yl)-2,2-dimethyltetrahydrofuro[3,4-*d*][1,3]dioxol-4-yl)-5-fluoro-1-(tetrahydrofuran-2-yl)pyrimidine-2,4(1*H*,3*H*)-dione (9b)

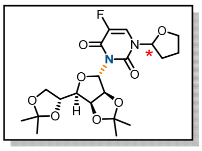

Purified by flash column chromatography (ethyl acetate / petroleum ether = 1:6, v:v); colorless oil (76 mg, 58%); $[\alpha]_D^{25}$ = +17.4 (c =1.3, CHCl$_3$); ^{1}**H NMR** (600 MHz, Acetone-d_6) δ 7.77 (dd, J = 6.4, 1.7 Hz, 1H), 6.39 (d, J = 3.0 Hz, 1H), 6.02–5.93 (m, 1H), 5.23 (dd, J = 11.4, 6.1 Hz, 1H), 5.03 (dd, J = 6.1, 4.1 Hz, 1H), 4.61–4.56 (m, 1H), 4.38–4.33 (m, 1H), 4.31 (q, J = 6.2 Hz, 1H), 4.00 (dd, J = 8.3, 6.5 Hz, 1H), 3.97–3.91 (m, 1H), 3.87 (dd, J = 8.4, 5.9 Hz, 1H), 2.40–2.31 (m, 1H), 2.23–2.15 (m, 1H), 2.07-2.04 (m, 1H), 2.04–2.01 (m, 1H), 1.47 (s, 3H), 1.33 (s, 3H), 1.31 (s, 3H), 1.28 (s, 3H); ^{13}**C NMR** (151 MHz, Acetone-d_6) δ 158.14 (d, J = 4.0 Hz), 157.97 (d, J = 4.2 Hz), 150.2, 150.1, 141.5, 140.0, 124.56 (d, J = 4.6 Hz), 124.34 (d, J = 4.4 Hz), 113.0, 112.9, 108.9, 108.9, 89. 7, 89.0, 89.0, 87.3, 85.8, 85.8, 83.0, 83.0, 74.7, 74.7, 70.9, 70.9, 66.8, 66.8, 33.2, 33.1, 30.6, 27.0, 27.0, 26.5, 26.5, 25.5, 24.5, 24.4, 24.3, 24.3 (two groups of ^{13}C-NMR peaks exist due to isomers as indicated by the red star); ^{19}**F NMR** (565 MHz, Acetone-d_6) δ-167.61 (s, 1F); ^{19}**F {^{1}H} NMR** (565 MHz, Acetone-d_6) δ -167.61(s, 1F); **HRMS (ESI)** m/z cald for $C_{20}H_{27}FN_2O_8Na^+$ [M+Na]$^+$ 465.1644, found 465.1650.

3-((3a*S*,6*R*,6a*S*)-6-((*R*)-2,2-dimethyl-1,3-dioxolan-4-yl)-2,2-dimethyltetrahydrofuro[3,4-*d*][1,3]dioxol-4-yl)-5-((3,5-dimethylphenoxy)methyl)oxazolidin-2-one (9c)

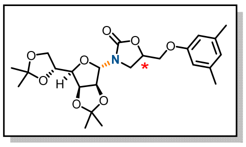

Purified by flash column chromatography (ethyl acetate/ petroleum ether = 1 : 2.7, v:v); colorless oil (34 mg, 37%); $[\alpha]_D^{25}$ = +44.5 (c =0.5, CHCl$_3$); ^{1}**H NMR** (400 MHz, CDCl$_3$) δ 6.64 (s, 1H), 6.54 (s, 1H), 6.53 (s, 1H), 5.40 (dd, J = 9.7, 5.8 Hz, 1H), 5.05–4.97 (m, 2H), 4.90–4.80 (m, 1H), 4.42–4.33 (m, 1H), 4.27–4.18 (m, 1H), 4.18–4.00 (m, 4H), 3.98–3.55 (m, 2H), 2.29 (s, 6H), 1.49 (d, J = 1.6 Hz, 3H), 1.45 (d, J = 4.6 Hz, 3H), 1.37 (s, 3H), 1.36 (s, 3H); ^{13}**C NMR** (101 MHz, CDCl$_3$) δ 158.2, 157.1, 157.0, 139.6, 123.6, 123.6, 112.8, 112.8, 112.5, 112.5, 109.3, 109.3, 90.9, 90.8,

83.6, 83.6, 83.6, 83.4, 80.9, 73.7, 73.6, 71.9, 71.6, 67.9, 67.8, 66.7, 66.5, 47.3, 47.3, 27.0, 26.9, 26.1, 26.1, 25.3, 25.2, 24.5, 24.5, 21.5, 21.5 (two groups of ^{13}C-NMR peaks exist due to isomers as indicated by the red star); **HRMS (ESI)** m/z cald for $C_{24}H_{33}NO_8Na^+$ $[M+Na]^+$ 486.2098, found 486.2097.

2-((1-((3a*S*,4*S*,6*R*,6a*S*)-6-((*R*)-2,2-dimethyl-1,3-dioxolan-4-yl)-2,2-dimethyltetrahydrofuro[3,4-*d*][1,3]dioxol-4-yl)-3-((*E*)-2-(pyridin-2-yl)vinyl)-1*H*-indazol-6-yl)thio)-*N*-methylbenzamide (9d)

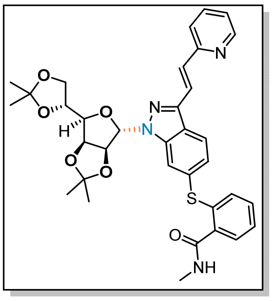

Purified by flash column chromatography (MeOH/DCM = 1:20, v:v); colorless oil; (59 mg, 47%), $[\alpha]_D^{25}$ = +50.3 (*c* =0.6, $CHCl_3$); **^{1}H NMR** (400 MHz, DMSO-d_6) δ 8.65–8.59 (m, 1H), 8.40 (q, *J* = 4.5 Hz, 1H), 8.28–8.22 (m, 1H), 8.14 (s, 1H), 7.93 (d, *J* = 16.4 Hz, 1H), 7.83 (td, *J* = 7.7, 1.8 Hz, 1H), 7.72–7.61 (m, 2H), 7.54–7.48 (m, 1H), 7.35–7.27 (m, 4H), 7.24 (dd, *J* = 8.5, 1.4 Hz, 1H), 7.06–7.01 (m, 1H), 6.63 (s, 1H), 5.49 (d, *J* = 5.9 Hz, 1H), 5.15 (dd, *J* = 5.9, 3.8 Hz, 1H), 4.39–4.29 (m, 1H), 4.18 (dd, *J* = 5.7, 3.8 Hz, 1H), 3.96 (dd, *J* = 8.5, 6.6 Hz, 1H), 3.77 (dd, *J* = 8.4, 5.9 Hz, 1H), 2.78 (d, *J* = 4.6 Hz, 3H), 1.50 (s, 3H), 1.37 (s, 3H), 1.26 (s, 3H), 1.24 (s, 3H); **^{13}C NMR** (101 MHz, DMSO-d_6) δ 168.3, 154.9, 150.1, 143.3, 142.0, 137.4, 137.4, 136.1, 134.5, 131.5, 130.8, 130.5, 128.2, 127.2, 126.6, 123.4, 123.3, 123.0, 122.5, 121.7, 115.4, 112.5, 108.3, 89.5, 84.6, 82.7, 80.5, 73.3, 65.8, 26.9, 26.6, 26.2, 25.5, 24.7; **HRMS (ESI)** m/z cald for $C_{34}H_{36}N_4O_6SNa^+$ $[M+Na]^+$ 651.2248, found 651.2235.

(2-butyl-4-chloro-1-((2'-(2-((3a*S*,4*S*,6*R*,6a*S*)-6-((*R*)-2,2-dimethyl-1,3-dioxolan-4-yl)-2,2-dimethyltetrahydrofuro[3,4-*d*][1,3]dioxol-4-yl)-2*H*-tetrazol-5-yl)-[1,1'-biphenyl]-4-yl)methyl)-1*H*-imidazol-5-yl)methanol (9e)

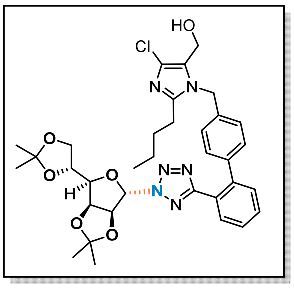

Purified by flash column chromatography (ethyl acetate / petroleum ether = 1:3.5, v:v); colorless oil; (81 mg, 61%), $[\alpha]_D^{25}$ = +19.2 (*c* =1.3, $CHCl_3$); **^{1}H NMR** (400 MHz, $CDCl_3$) δ 7.92 (dd, *J* = 7.6, 1.6 Hz, 1H), 7.58–7.46 (m, 2H), 7.39 (dd, *J* = 7.4, 1.6 Hz, 1H), 7.15 (d, *J* = 7.9 Hz, 2H), 7.00 (d, *J* = 8.2 Hz, 2H), 6.33 (s, 1H), 5.24 (s, 2H), 5.05 (d, *J* = 5.8 Hz, 1H), 4.87 (dd, *J* = 5.9, 3.8 Hz, 1H), 4.50 (s, 2H), 4.39 (td, *J* = 6.8, 4.4 Hz, 1H), 4.04 (dt, *J* = 7.3, 5.2 Hz, 2H), 3.85 (dd, *J* = 8.8, 4.4 Hz, 1H), 2.58 (t, *J* = 7.8 Hz, 2H), 1.63 (ddd, *J* = 15.4, 8.8, 6.6 Hz, 2H), 1.54 (s, 3H), 1.41 (s, 3H), 1.33 (d, *J* = 7.5 Hz, 9H), 0.86 (t, *J* = 7.3 Hz, 3H). **^{13}C NMR** (101 MHz, $CDCl_3$) δ 164.4, 147.5, 140.2,

139.7, 133.9, 129.9, 129.4, 129.3, 128.8, 126.8, 125.8, 124.8, 124.4, 124.1, 112.8, 108.4, 92.7, 83.7, 82.6, 78.9, 71.8, 65.4, 52.0, 46.6, 28.7, 25.8, 25.7, 24.9, 24.1, 23.5, 21.4, 12.7; **HRMS (ESI)** m/z cald for $C_{34}H_{41}ClN_6O_6Na^+$ $[M+Na]^+$ 687.2668, found 687.2675.

13-((3a*S*,4*S*,6*R*,6a*S*)-6-((*R*)-2,2-dimethyl-1,3-dioxolan-4-yl)-2,2-dimethyltetrahydrofuro[3,4-*d*][1,3]dioxol-4-yl)-8,13-dihydroindolo[2',3':3,4]pyrido[2,1-*b*]quinazolin-5(7*H*)-one (9f)

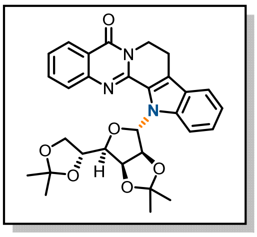

Purified by flash column chromatography (acetone/DCM = 1:20, v:v); white solid (112 mg, 53%), m.p.: 218-220 °C; $[\alpha]_D^{25}$ = -15.2 (*c* =0.6, $CHCl_3$); **^{1}H NMR** (600 MHz, $CDCl_3$) δ 8.31 (dd, *J* = 8.0, 1.5 Hz, 1H), 7.75–7.69 (m, 1H), 7.67 (d, *J* = 8.1 Hz, 1H), 7.63 (d, *J* = 7.9 Hz, 1H), 7.51 (d, *J* = 3.0 Hz, 1H), 7.50–7.35 (m, 3H), 7.22 (t, *J* = 7.3 Hz, 1H), 5.61 (dd, *J* = 5.9, 3.0 Hz, 1H), 5.23 (dd, *J* = 5.9, 4.0 Hz, 1H), 4.69 (dd, *J* = 7.3, 4.1 Hz, 1H), 4.66–4.58 (m, 1H), 4.56–4.51 (m, 1H), 4.51–4.44 (m, 1H), 4.14 (dd, *J* = 8.8, 6.3 Hz, 1H), 4.06 (dd, *J* = 8.8, 4.9 Hz, 1H), 3.13 (t, *J* = 6.7 Hz, 2H), 1.69 (s, 3H), 1.43 (s, 3H), 1.42 (s, 3H), 1.40 (s, 3H); **^{13}C NMR** (151 MHz, $CDCl_3$) δ 161.6, 147.1, 145.2, 139.6, 134.4, 127.3, 127.3, 126.9, 126.6, 126.2, 125.5, 123.1, 121.4, 121.0, 120.5, 113.9, 112.1, 109.3, 93.2, 85.5, 83.3, 81.6, 74.1, 66.8, 40.4, 27.2, 27.0, 25.5, 25.4, 20.0; **HRMS (ESI)** m/z cald for $C_{30}H_{31}N_3O_6Na^+$ $[M+Na]^+$ 552.2105, found 552.2111.

2.3 N-glycosylation procedure on 1 mmol scale

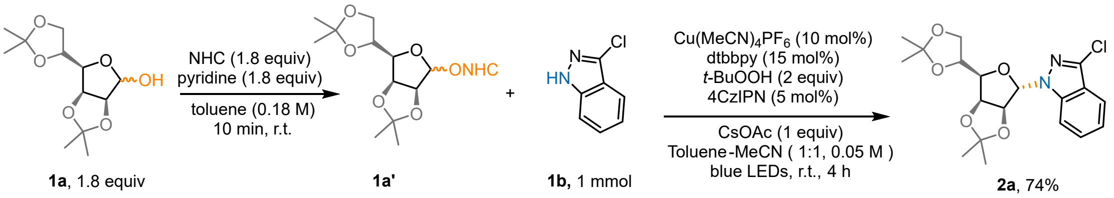

An oven-dried 50 mL Schlenk tube was charged with 1-hydroxylmannose **1a** (470.3 mg, 1.8 mmol, 1.8 equiv), NHC (395.3 mg, 1.8 mmol, 1.8 equiv) and a magnetic stir bar. After the Schlenk tube was vacuumed and refilled with nitrogen gas three times, dry toluene (10 mL) was added and the reaction stirred at r.t. for 5 min. Then, pyridine (145.5 μL, 1.8 mmol, 1.8 equiv) was added dropwise at room temperature. The resulting solution stirred at r.t. for 10 min. A white solid precipitated out during this time.

Another 10 mL Schlenk tube was charged with 4CzIPN (38 mg, 0.1 mmol, 0.05 equiv),

$Cu(MeCN)_4PF_6$ (37.4 mg, 0.1 mmol, 0.1 equiv), dtbbpy (40.0 mg, 0.15 mmol, 0.15 equiv) CsOAc (192 mg, 1 mmol, 1.0 equiv), 3-chlorideindazole (156 mg, 1 mmol, 1.0 equiv) and a magnetic stir bar. And this Schlenk tube was vacuumed and refilled with nitrogen gas three times. Dry MeCN (10 mL) was added to this Schlenk tube under an atmosphere of nitrogen and stirred at r.t.

The toluene suspension was transferred to a 10 mL syringe under an atmosphere of nitrogen (twice). Then a syringe filter and new needle were installed on the syringe, before the toluene solution was injected through the syringe filter into the MeCN solution. *t*-BuOOH (400 μL, 5-6 M in decane, 2.0 equiv) was added before placing under 420 nm blue LEDs. The mixture then irradiates at r.t. for 4 h. The organic layers were evaporated and then purified by flash column chromatography (petroleum ether/acetone = 150:1 ~ 80:1) on silica gel to give the desired product **2a** (288 mg, 74%) as a white solid.

2.4 Oxalate, DHP ester, xanthate salt as glycosyl donors

Glycosyl oxalate (**1c**) [3], glycosyl xanthate salt (**1d)** [6], glycosyl DHP ester [7] (**1e**) were synthesized according to the reported procedures. Due to the instability of **1c** and **1d**, they were used directly without purification. **1e** was purified by column chromatography on silica gel.

An oven-dried 10 mL Schlenk tube was charged with glycosyl donor (**1c**, **1d,** or **1e,** 0.36 mmol, 1.8 equiv), 4CzIPN (7.5 mg, 0.01 mmol, 0.05 equiv), $Cu(MeCN)_4PF_6$ (7.4 mg, 0.02 mmol, 0.1 equiv), dtbbpy (8.0 mg, 0.03 mmol, 0.15 equiv), CsOAc (38.4 mg, 0.2 mmol, 1.0 equiv), 3-chlorideindazole (31.2 mg, 0.2 mmol, 1.0 equiv) and a magnetic stir bar. And this Schlenk tube was vacuumed and refilled with nitrogen gas three times. Dry MeCN (2.0 mL) was added to this Schlenk tube under an atmosphere of nitrogen and stirred for 5 min. *t*-BuOOH (80 μL, 5-6 M in decane, 2 equiv) was added before placing under 420 nm blue LEDs. The mixture then irradiates at r.t. for 4 h. Virtually no products were observed in each reaction.

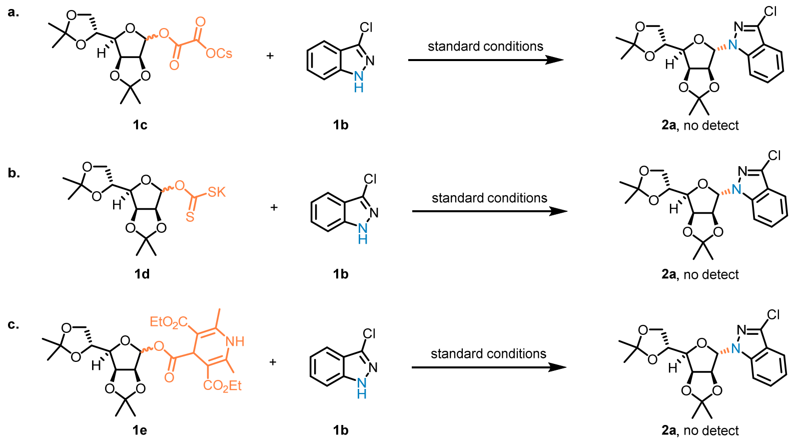

Supplementary Figure 4 | Oxalate, DHP ester, xanthate salt as glycosyl donors

2.5 Mechanistic studies

2.5.1 Radical trapping experiments

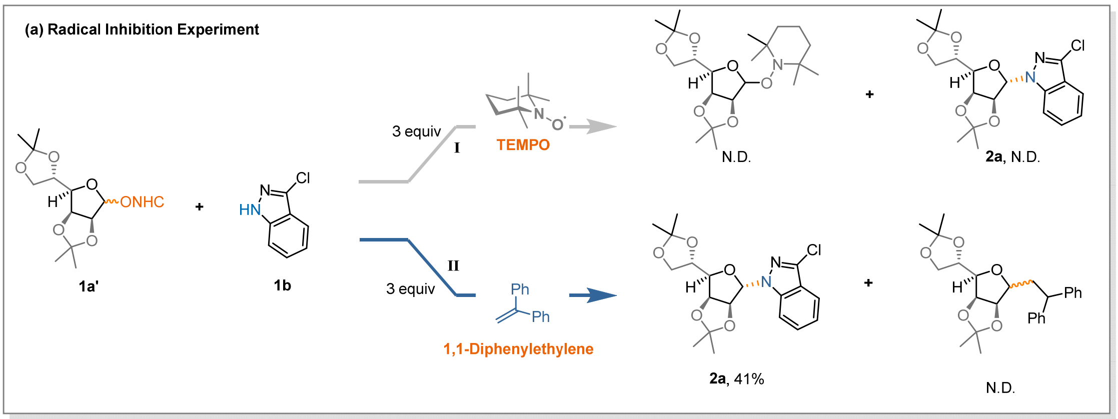

Supplementary Figure 5 | Radical trapping experiments

(I) *With TEMPO:* An oven-dried 10 mL Schlenk tube was charged with 1-hydroxylmannose **1a** (94 mg, 0.36 mmol, 1.8 equiv), NHC (142.4 mg, 0.36 mmol, 1.8 equiv) and a magnetic stir bar. After the Schlenk tube was vacuumed and refilled with nitrogen gas three times, dry toluene (2.0 mL) was added and the reaction stirred at r.t. for 5 min. Then, pyridine (29.1 µL, 0.36 mmol, 1.8 equiv) was added dropwise at room temperature. The resulting solution stirred at r.t. for 10 min. A white solid precipitated out during this time.

Another 10 mL Schlenk tube was charged with TEMPO (93.5 mg, 0.6 mmol, 3.0 equiv), 4CzIPN (7.5 mg, 0.01 mmol, 0.05 equiv), $Cu(MeCN)_4PF_6$ (7.4 mg, 0.02 mmol, 0.1 equiv), dtbbpy (8.0 mg, 0.03 mmol, 0.15 equiv), CsOAc (38.4 mg, 0.2 mmol, 1.0 equiv), 3-chlorideindazole (31.2 mg, 0.2 mmol, 1.0 equiv) and a magnetic stir bar. And this Schlenk tube was vacuumed and refilled with nitrogen gas three times. Dry MeCN (2.0 mL) was added to

this Schlenk tube under an atmosphere of nitrogen and stirred at r.t.

The toluene suspension was transferred to a 5 mL syringe under an atmosphere of nitrogen. Then a syringe filter and new needle were installed on the syringe, before the toluene solution was injected through the syringe filter into the MeCN solution. *t*-BuOOH (80 μL 5-6 M in decane, 2.0 equiv) was added before placing under 420 nm blue LEDs. The mixture then irradiates at r.t. for 4 h.

(II): *With 1,1-Diphenylethylene:* An oven-dried 10 mL Schlenk tube was charged with 1-hydroxylmannose **1a** (94 mg, 0.36 mmol, 1.8 equiv), NHC (142.4 mg, 0.36 mmol, 1.8 equiv) and a magnetic stir bar. After the Schlenk tube was vacuumed and refilled with nitrogen gas three times, dry toluene (2.0 mL) was added and the reaction stirred at r.t. for 5 min. Then, pyridine (29.1 μL, 0.36 mmol, 1.8 equiv) was added dropwise at room temperature. The resulting solution stirred at r.t. for 10 min. A white solid precipitated out during this time.

Another 10 mL Schlenk tube was charged with 1,1-Diphenylethylene (108 mg, 0.6 mmol, 3.0 equiv), 4CzIPN (7.5 mg, 0.01 mmol, 0.05 equiv), $Cu(MeCN)_4PF_6$ (7.4 mg, 0.02 mmol, 0.1 equiv), dtbbpy (8.0 mg, 0.03 mmol, 0.15 equiv), CsOAc (38.4 mg, 0.2 mmol, 1.0 equiv), 3-chlorideindazole (31.2 mg, 0.2 mmol, 1.0 equiv) and a magnetic stir bar. And this Schlenk tube was vacuumed and refilled with nitrogen gas three times. Dry MeCN (2.0 mL) was added to this Schlenk tube under an atmosphere of nitrogen and stirred at r.t.

The toluene suspension was transferred to a 5 mL syringe under an atmosphere of nitrogen. Then a syringe filter and new needle were installed on the syringe, before the toluene solution was injected through the syringe filter into the MeCN solution. *t*-BuOOH (80 μL, 5-6 M in decane, 2.0 equiv) was added before placing under 420 nm blue LEDs. The mixture then irradiates at r.t. for 4 h.

2.5.2 Radical clock experiments

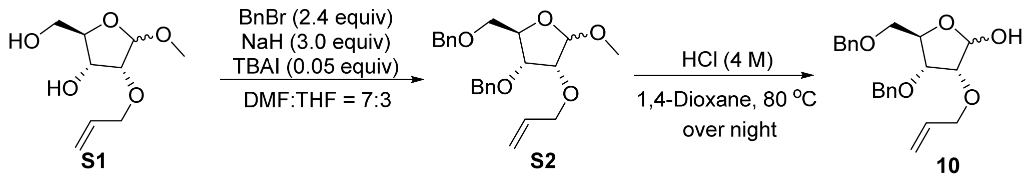

Compound **S1** [4] (1.02 g, 5 mmol, 1.0 equiv) and TBAI (92 mg) were placed in a 100 mL round-bottom flask, along with a magnetic stirring bar. The flask was evacuated and backfilled with N_2 three times, and a N_2 balloon was attached before adding DMF (35 mL) and THF (15 mL, 0.1 M). The mixture was cooled to 0 °C. Subsequently, NaH was added in batches (600

mg, 15 mmol, 3.0 equiv) and stirred at the same temperature for 1 hour. The reaction mixture was then allowed to warm up to room temperature. BnBr (1.43 mL, 12 mmol, 2.4 equiv) was added dropwise to the reaction mixture. After vigorous stirring for 8 hours, the reaction mixture was quenched with water (2 mL), and then concentrated under reduced pressure. Compound **S2** (1.42 g, 74%) was obtained as a mixture of diastereomers by column chromatography (silica gel, EtOAc / petroleum ether, 0:100 to 1:20) and used directly for the next step.

Compound **S2** (1.42 g, 3.7 mmol) was dissolved in 10 mL of 1,4-dioxane and added to a 50 mL round-bottom flask equipped with a magnetic stirring bar. Subsequently, 10 mL of 4 M HCl was introduced. The resulting mixture was heated to 80 °C and stirred for 18 hours. Following this, the mixture was concentrated under reduced pressure. Purification of the mixture was performed through column chromatography on silica gel using an eluent mixture of ethyl acetate and petroleum ether in increasing proportions ranging from 0:100 to 1:5. This technique afforded compound **10** (790 mg, 58%) as yellow syrup [2]. $[\alpha]_D^{25}$ = +22.1 (c = 1.2, $CHCl_3$); **^{1}H NMR** (400 MHz, $CDCl_3$) δ 7.29–7.16 (m, 10H), 5.87 (m, 1H), 5.26–5.18 (m, 2H), 5.13 (d, J = 10.4 Hz, 1H), 4.67–4.35 (m, 4H), 4.31–3.99 (m, 4H), 3.95–3.35 (m, 4H); **^{13}C NMR** (101 MHz, $CDCl_3$) δ 138.0, 137.8, 137.6, 137.5, 134.5, 134.2, 128.6, 128.6, 128.5, 128.5, 128.1, 128.0, 128.0, 127.9, 127.8, 127.7, 117.9, 117.8, 100.5, 96.3, 81.0, 81.0, 80.9, 77.8, 77.7, 77.3, 73.6, 73.6, 72.9, 72.6, 71.7, 71.6, 70.1, 69.6.

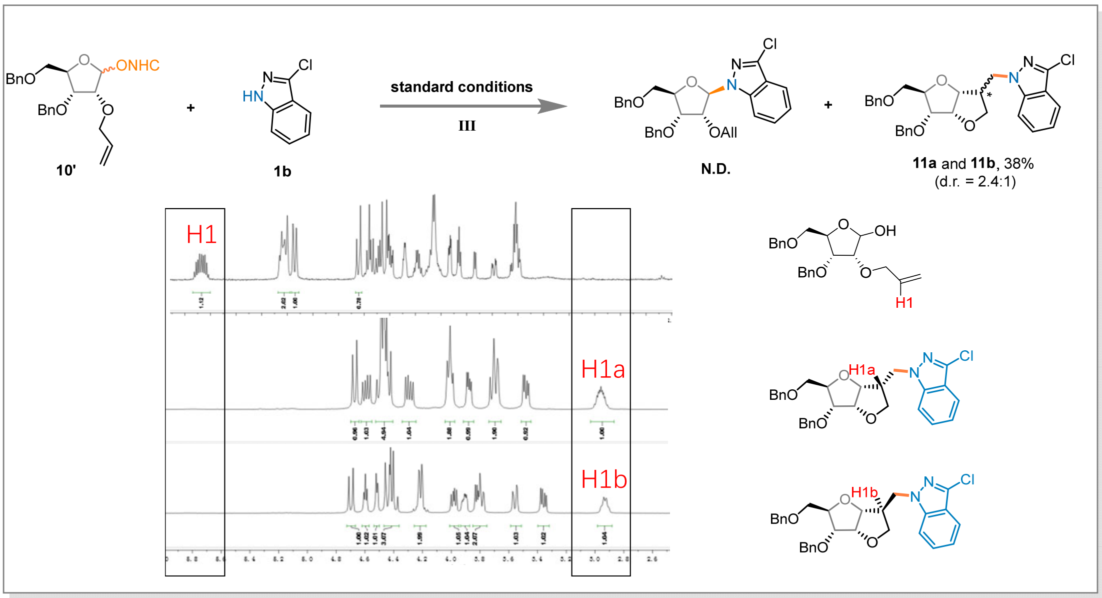

Supplementary Figure 6 | Radical clock experiments

(III): 1d *as radical donor:* An oven-dried 10 mL Schlenk tube was charged with 1-hydroxylmannose **10** (133 mg, 0.36 mmol, 1.8 equiv), NHC (142.4 mg, 0.36 mmol, 1.8 equiv.) and a magnetic stir bar. After the Schlenk tube was vacuumed and refilled with nitrogen gas

three times, dry toluene (2.0 mL) was added and the reaction stirred at r.t. for 5 min. Then, pyridine (29.1 μL, 0.36 mmol, 1.8 equiv) was added dropwise at room temperature. The resulting solution stirred at r.t. for 10 min. A white solid precipitated out during this time.

Another 10 mL Schlenk tube was charged with 4CzIPN (7.5 mg, 0.01 mmol, 0.05 equiv), $Cu(MeCN)_4PF_6$ (7.4 mg, 0.02 mmol, 0.1 equiv), dtbbpy (8 mg, 0.03 mmol, 0.15 equiv) CsOAc (38.4 mg, 0.2 mmol, 1.0 equiv), 3-chlorideindazole (31.2 mg, 0.2 mmol, 1.0 equiv) and a magnetic stir bar. And this Schlenk tube was vacuumed and refilled with nitrogen gas three times. Dry MeCN (2.0 mL) was added to this Schlenk tube under an atmosphere of nitrogen and stirred in a parallel light low-temperature reactor (0 ºC, 420 nm blue LEDs).

The toluene suspension was transferred to a 5 mL syringe under an atmosphere of nitrogen. Then a syringe filter and new needle were installed on the syringe, before the toluene solution was injected through the syringe filter into the MeCN solution. *t*-BuOOH (80 μL, 5-6 M in decane, 2 equiv) was added and irradiated at 0 ºC for 4 h. The organic layers were evaporated and then purified by flash column chromatography (petroleum ether/ ethyl acetate = 100:1~20:1) on silica gel.

1-(((3aR,5R,6R,6aS)-6-(benzyloxy)-5-((benzyloxy)methyl)hexahydrofuro[3,2-b]furan-3-yl)methyl)-3-chloro-1*H*-indazole (11a)

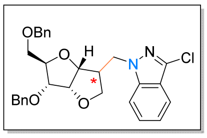

Purified by flash column chromatography (petroleum ether/ ethyl acetate = 100:1~20:1, v:v); Yellow oil; (26.7 mg, 27%); $[\alpha]_D^{25}$ = +22.8 (c = 0.46, $CHCl_3$); **^{1}H NMR** (400 MHz, $CDCl_3$) δ 7.55 (d, *J* = 8.2 Hz, 1H), 7.41 (d, *J* = 8.5 Hz, 1H), 7.31–7.15 (m, 11H), 7.09 (t, *J* = 7.5 Hz, 1H), 4.66 (d, *J* = 11.8 Hz, 1H), 4.58 (dd, *J* = 14.1, 7.4 Hz, 1H), 4.51–4.38 (m, 4H), 4.27 (dd, *J* = 14.1, 7.0 Hz, 1H), 3.98 (td, *J* = 8.1, 7.7, 3.2 Hz, 2H), 3.84 (dd, *J* = 8.4, 4.1 Hz, 1H), 3.71 – 3.62 (m, 2H), 3.44 (dd, *J* = 10.9, 4.6 Hz, 1H), 2.97–2.83 (m, 1H); **^{13}C NMR** (101 MHz, $CDCl_3$) δ 141.2, 138.2, 137.8, 133.3, 128.5, 128.5, 128.1, 128.0, 127.7, 127.7, 127.6, 121.4, 121.0, 119.7, 109.9, 82.5, 81.8, 81.7, 80.2, 73.6, 72.3, 72.3, 70.2, 46.7, 45.5; **HRMS (ESI)** m/z cald for $C_{29}H_{29}ClN_2O_4Na^+$ $[M+Na]^+$ 527.1708, found 527.1697.

1-(((3aR,5R,6R,6aS)-6-(benzyloxy)-5-((benzyloxy)methyl)hexahydrofuro[3,2-b]furan-3-yl)methyl)-3-chloro-1*H*-indazole (11b)

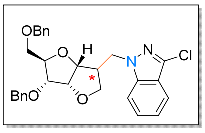

Purified by flash column chromatography (petroleum ether/ ethyl acetate = 100:1~20:1, v:v); Yellow oil; (11.1 mg, 11%); $[\alpha]_D^{25}$ = +19.3 (c = 0.15, $CHCl_3$); **^{1}H NMR** (400 MHz, $CDCl_3$) δ 7.60 (d, *J* = 8.2 Hz, 1H), 7.36 (dd,

J = 8.3, 7.0 Hz, 1H), 7.30–7.17 (m, 11H), 7.13 (t, *J* = 7.5 Hz, 1H), 4.70 (d, *J* = 11.8 Hz, 1H), 4.60 (t, *J* = 4.5 Hz, 1H), 4.52 (d, *J* = 4.4 Hz, 1H), 4.48–4.35 (m, 3H), 4.28–4.14 (m, 2H), 3.98 (dd, *J* = 9.4, 5.6 Hz, 1H), 3.91 (ddd, *J* = 7.4, 4.8, 2.3 Hz, 1H), 3.80 (ddd, *J* = 11.4, 8.9, 3.6 Hz, 2H), 3.56 (dd, *J* = 10.7, 2.3 Hz, 1H), 3.36 (dd, *J* = 10.8, 4.8 Hz, 1H), 2.94 (dd, *J* = 10.5, 4.8 Hz, 1H); **^{13}C NMR** (101 MHz, $CDCl_3$) δ 141.1, 138.1, 137.8, 133.4, 128.6, 128.5, 128.1, 128.1, 128.0, 127.9, 127.8, 121.6, 121.4, 120.1, 109.1, 85.1, 80.7, 80.1, 79.7, 73.6, 72.4, 72.0, 69.9, 49.3, 48.6; **HRMS (ESI)** m/z cald for $C_{29}H_{29}ClN_2O_4Na^+$ $[M+Na]^+$ 527.1708, found 527.1703.

2.5.2 Cyclic voltammetry data

Cyclic voltammetry experiment was performed in a three-electrode cell connected to an undivided three-necked bottle with stir bar under nitrogen at room temperature. The working electrode was a glass carbon electrode, while the counter electrode was a platinum wire. The reference electrode was an Ag/AgCl electrode submerged in a saturated aqueous KCl solution. After adding 0.01 mmol substrates (if solid), the mixture then purged with nitrogen. Under the protection of N_2, anhydrous and degassed 10 mL 0.01M $^{n}Bu_4N^+BF_4^-$ solution (in CH_3CN) were injected into the electrochemical cell. The scan rate was set at 0.1 V/s, covering a voltage range from 0 V to 3 V.

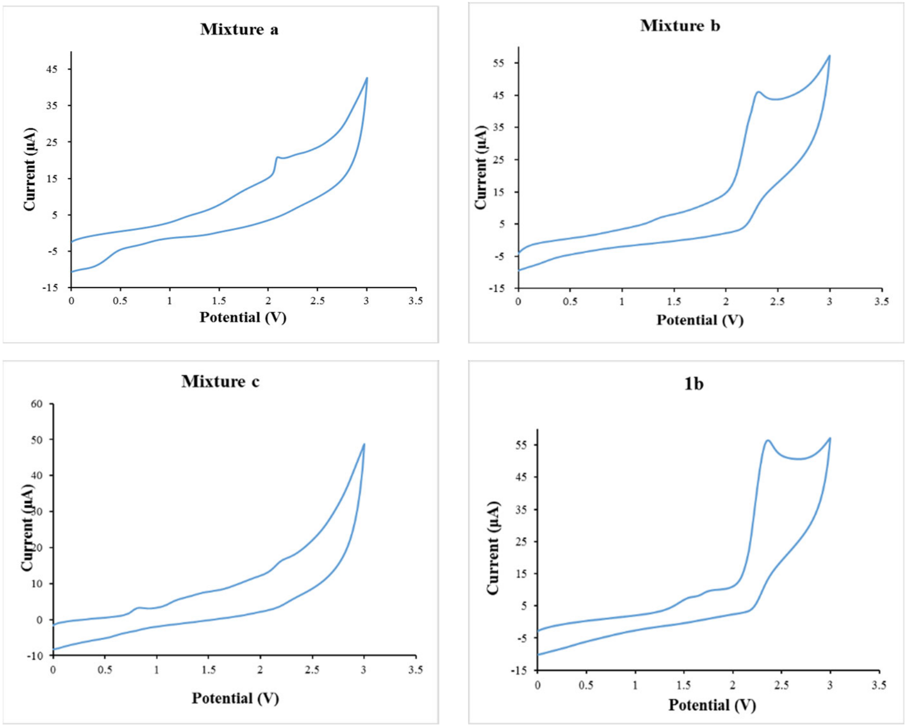

Supplementary Figure 7 | Cyclic voltammetry data of catalyst system.

Mixture a: 0.01 mmol $Cu(MeCN)_4PF_6$ + 0.01 mmol dtbbpy

Mixture b: 0.01 mmol $Cu(MeCN)_4PF_6$ + 0.01 mmol dtbbpy + 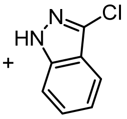 (0.01 mmol)

Mixture c: 0.01 mmol $Cu(MeCN)_4PF_6$ + 0.01 mmol dtbbpy + CsOAc + 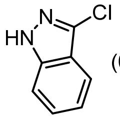 (0.01 mmol)

1b: 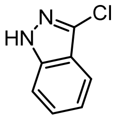 (0.01 mmol)

Supplementary Table 16 | Cyclic voltammetry data of catalyst system

Mixture	***E*** **(V)**
Mixture a	E_{ox} = 2.10 V
Mixture b	E_{ox} = 2.32 V
Mixture c	E_{ox} = 0.83 V
**1b**	E_{ox} = 2.36 V

2.5.3 Stern-Volmer experiment

The fluorescence quenching studies were performed using an Agilent Cary Eclipse Fluorescence Spectrophotometer. In each experiment, the photocatalyst and different concentrations of quencher were mixed in a 1:1 MeCN/toluene mixture in screw top 1.0 cm quartz cuvettes. Both MeCN and toluene were degassed by sparging with argon for 30 minutes. The emission quenching of 4CzIPN involved a photocatalyst concentration of 5.0×10^{-4} M. The solution was then irradiated at 420 nm, and the resulting emission intensity was observed at 550 nm. Plots were constructed according to the Stern–Volmer equation $I_0/I = 1 + k_q\tau_0[Q]$.

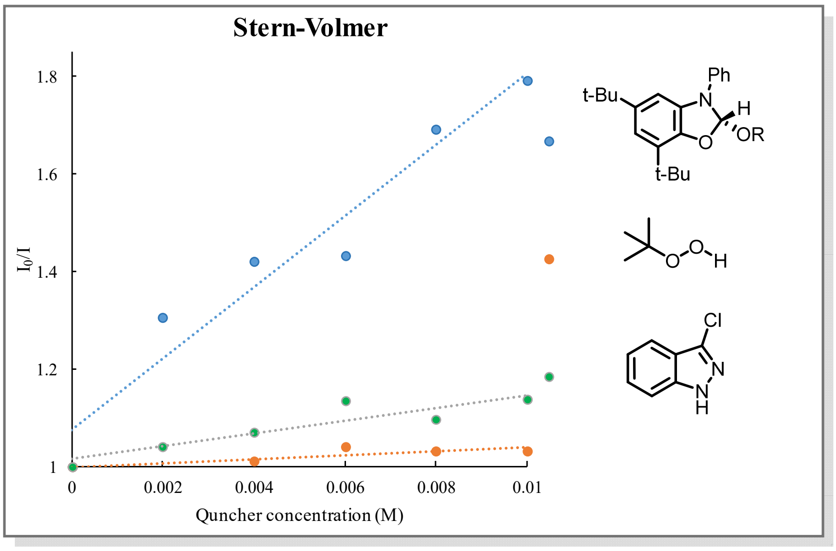

Supplementary Figure 8 | Emission quenching study of three different substrates with 4CzIPN in MeCN/toluene = 1 : 1 mixture.

2.5.4 UV-Vis spectra

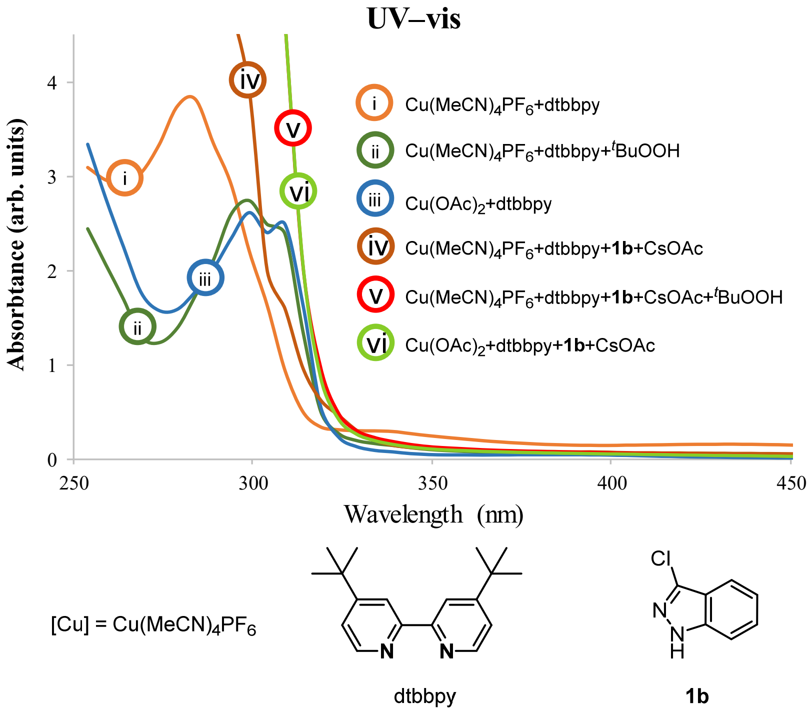

Supplementary Figure 9 | UV-Vis spectra of 0.1 mM solutions of copper salt mixture in MeCN.

2.5.5 Oxidation of copper salts

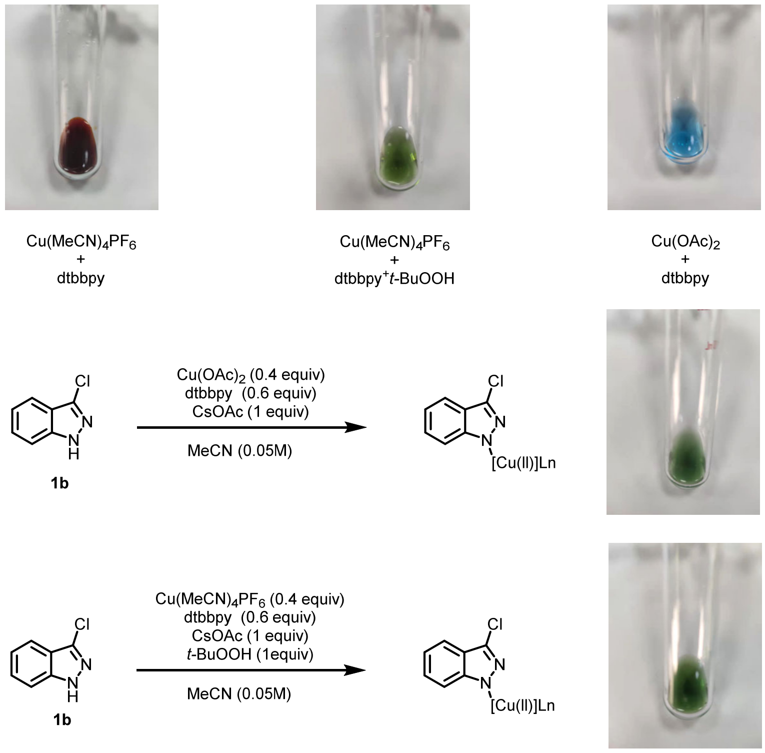

Supplementary Figure 10 | Oxidation of copper salt mixture in MeCN.

3. X-ray crystal structure data

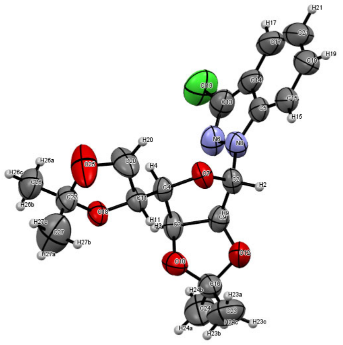

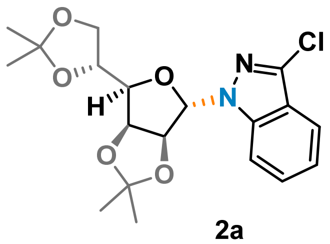

2a

The X-ray crystallographic coordinates for structures reported in this study have been deposited at the Cambridge Crystallographic Data Centre (CCDC), under deposition numbers 2305000. These data can be obtained free of charge from The Cambridge Crystallographic Data Centre via www.ccdc.cam.ac.uk/data_request/cif.

Supplementary Table 17 |. Crystal data and structure refinement for **2a**.

Identification code	**2a**	
Empirical formula	C19 H23 Cl N2 O5	
Formula weight	394.84	
Temperature	296(2) K	
Wavelength	0.71073 Å	
Crystal system	Orthorhombic	
Space group	$P2_12_12_1$	
Unit cell dimensions	a = 9.946(3) Å	α= 90°.
	b = 13.868(4) Å	β= 90°.
	c = 14.628(4) Å	γ = 90°.
Volume	2017.7(9) $Å^3$	
Z	4	
Density (calculated)	1.300 Mg/m^3	
Absorption coefficient	0.221 mm^{-1}	
F(000)	832	
Crystal size	0.240 x 0.220 x 0.210 mm^3	
Theta range for data collection	2.024 to 25.498°.	
Index ranges	-12<=h<=11, -16<=k<=16, -17<=l<=17	
Reflections collected	15818	
Independent reflections	3752 [R(int) = 0.0243]	
Completeness to theta = 25.242°	100.0 %	

Absorption correction	None
Refinement method	Full-matrix least-squares on F^2
Data / restraints / parameters	3752 / 0 / 249
Goodness-of-fit on F^2	1.045
Final R indices [I>2sigma(I)]	R1 = 0.0447, wR2 = 0.1115
R indices (all data)	R1 = 0.0579, wR2 = 0.1252
Absolute structure parameter	0.019(19)
Extinction coefficient	0.0113(16)
Largest diff. peak and hole	0.408 and -0.310 e.Å^{-3}

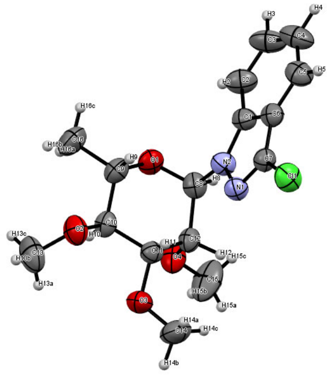

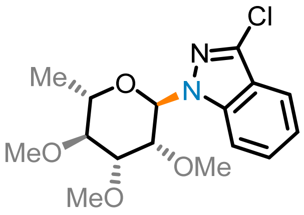

8o

The X-ray crystallographic coordinates for structures reported in this study have been deposited at the Cambridge Crystallographic Data Centre (CCDC), under deposition numbers 2305001. These data can be obtained free of charge from The Cambridge Crystallographic Data Centre via www.ccdc.cam.ac.uk/data_request/cif.

Supplementary Table 18 | Crystal data and structure refinement for **8o**

Identification code	**8o**	
Empirical formula	C16 H21 Cl N2 O4	
Formula weight	340.80	
Temperature	296(2) K	
Wavelength	0.71073 Å	
Crystal system	Monoclinic	
Space group	$P2_1$	
Unit cell dimensions	a = 8.025(2) Å	= 90°.
	b = 8.715(2) Å	= 95.615(3)°.
	c = 12.609(3) Å	= 90°.
Volume	877.6(4) Å^3	
Z	2	

Density (calculated)	1.290 Mg/m^3
Absorption coefficient	0.238 mm^{-1}
F(000)	360
Crystal size	0.220 x 0.180 x 0.170 mm^3
Theta range for data collection	2.550 to 25.498°.
Index ranges	-9<=h<=9, -9<=k<=10, -15<=l<=15
Reflections collected	6825
Independent reflections	2978 [R(int) = 0.0138]
Completeness to theta = 25.242°	99.9 %
Absorption correction	None
Refinement method	Full-matrix least-squares on F^2
Data / restraints / parameters	2978 / 1 / 212
Goodness-of-fit on F^2	1.046
Final R indices [I>2sigma(I)]	R1 = 0.0282, wR2 = 0.0776
R indices (all data)	R1 = 0.0300, wR2 = 0.0792
Absolute structure parameter	0.960(18)
Extinction coefficient	n/a
Largest diff. peak and hole	0.123 and -0.130 e.Å^{-3}

4. Structure Determination

4.1 Determination of the anomeric stereochemistry

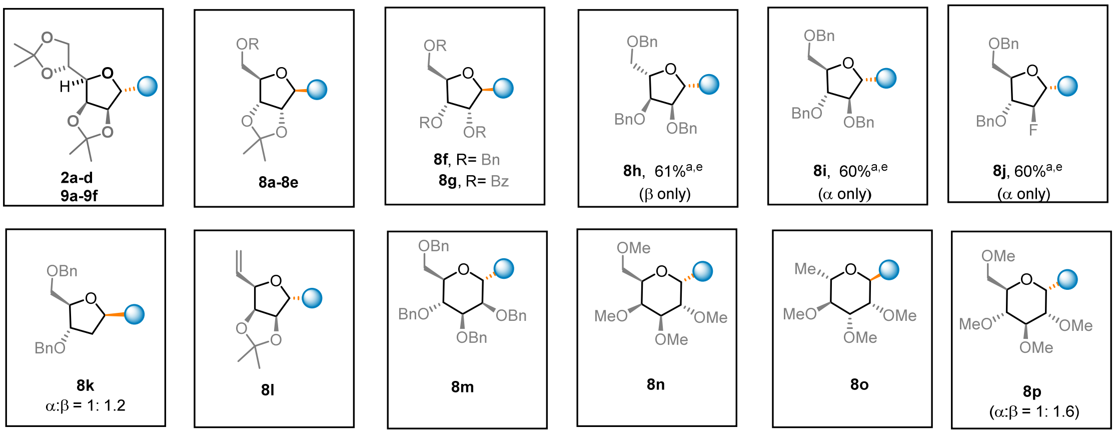

Supplementary Figure 11 | The types of sugar in this work

As shown in the figure above, there are 12 types of sugar used in this work. To determine the stereochemistry of the anomeric carbon, compound **2a**, **8a**, **8f**, **8h**, **8i**, **8j**, **8ka**, **8l**, **8m**, **8n**, **8o**, **8pb** were selected for detailed NMR analyses. Firstly, most meaningful protons were assigned with the aid of COSY. Then, characteristic NOE signals between H1 and H4 on the sugar ring were identified as shown in the following spectra (**8a**, **8f**, **8h**, **8i**, **8j**, **8ka**). For **8m**, **8n**, **8o** and **8pb**, the coupling constant J of anomeric hydrogen can judge their spatial configuration. Under such circumstances, the stereochemistry is determined as α when J < 6Hz. This conclusion is consistent with the X-Ray of **8o**. For **2a** and **8l**, there is no characteristic NOE signals between H1 and H4, indicating 1,4-trans relationship, which is consistent with X-ray crystal structure of **2a**. Therefore, the stereochemistry of **8l** was assigned in analogy to **2a**.

Anomeric stereochemistry of **2a** determined by X-Ray

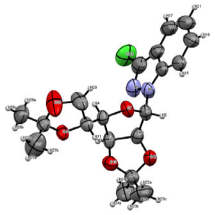

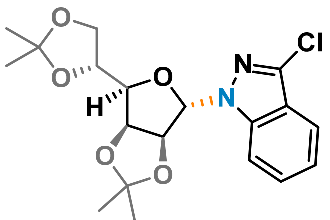

2a

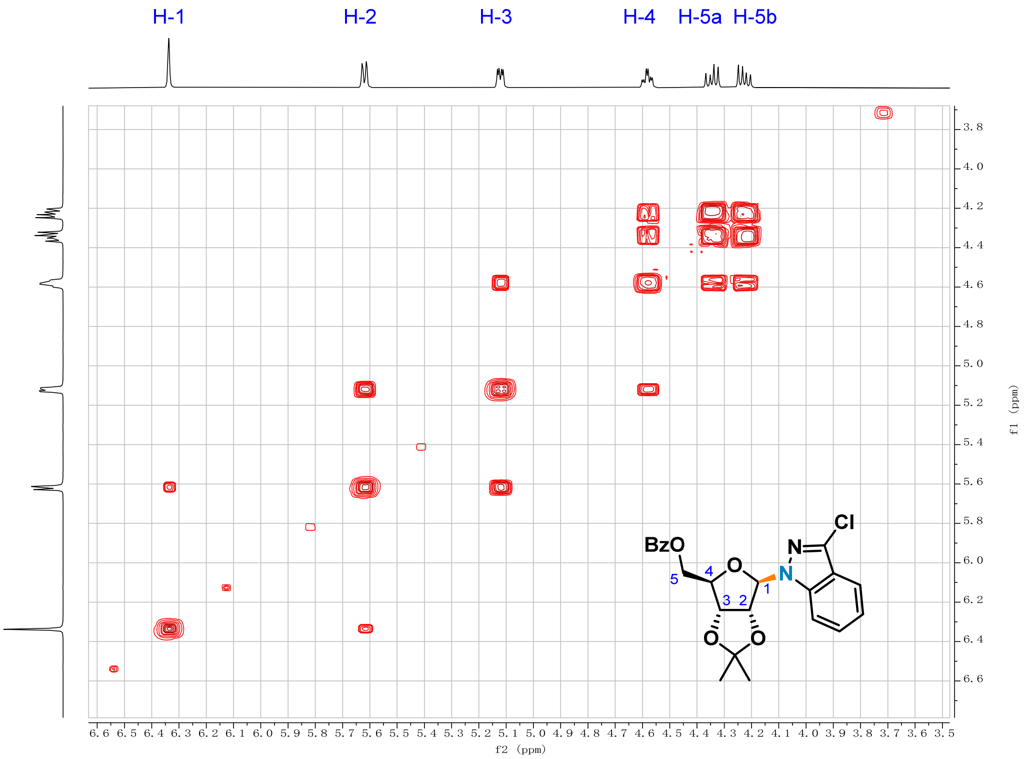

Supplementary Figure 12 | ^{1}H-^{1}H-COSY of **8a**

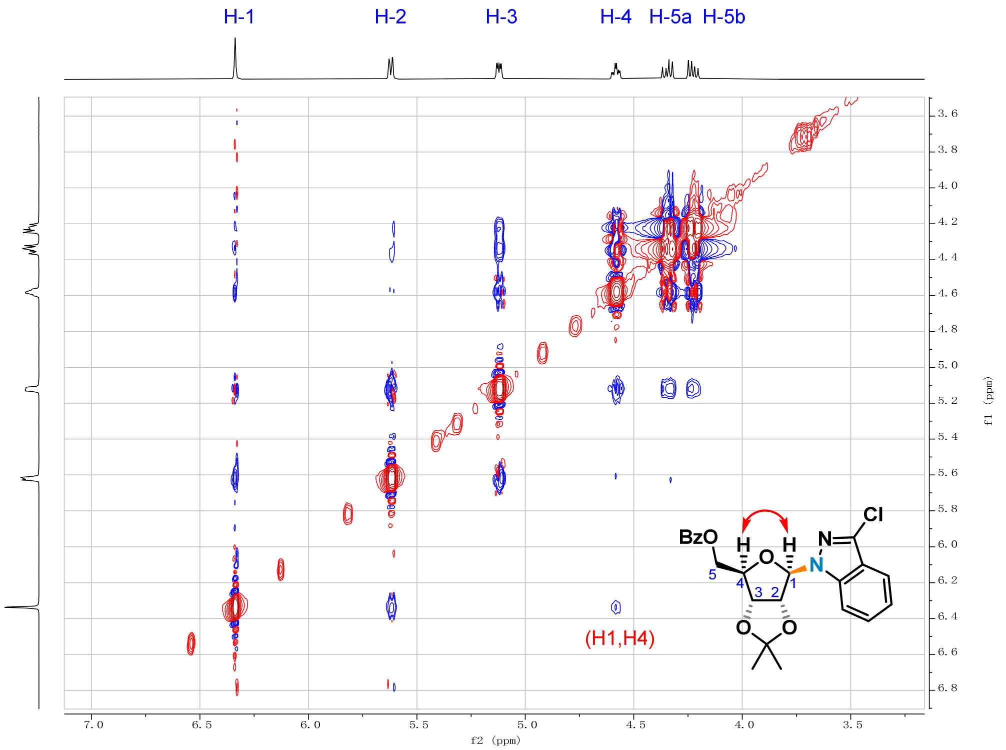

Supplementary Figure 13 | NOESY of **8a**

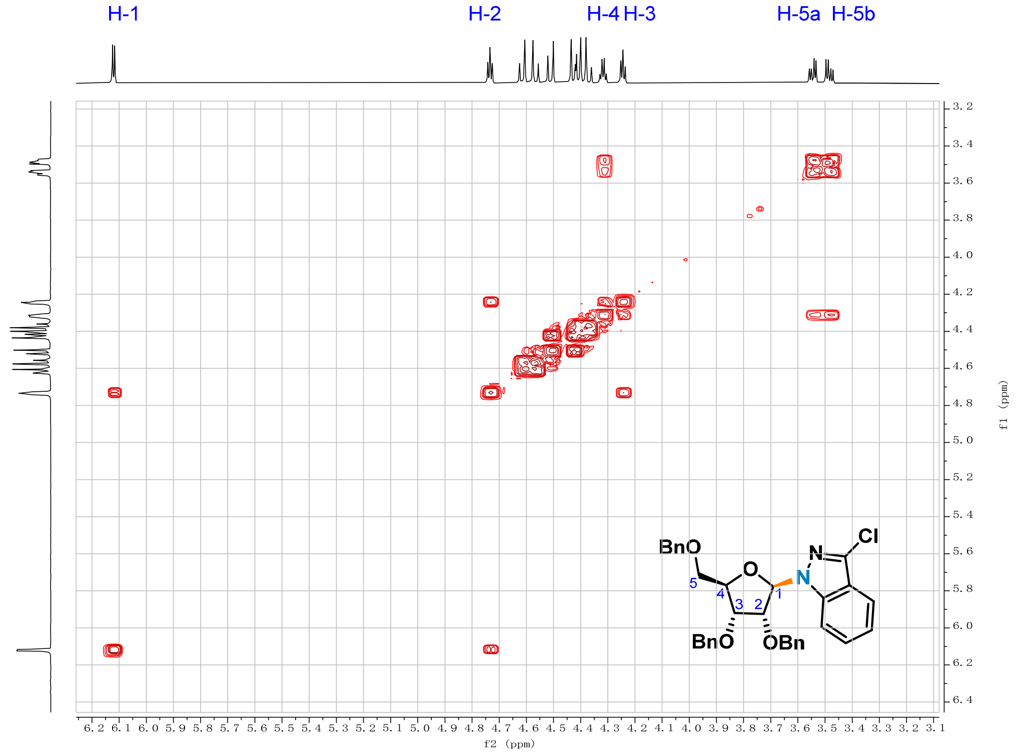

Supplementary Figure 14 | ^{1}H-^{1}H-COSY of **8f**

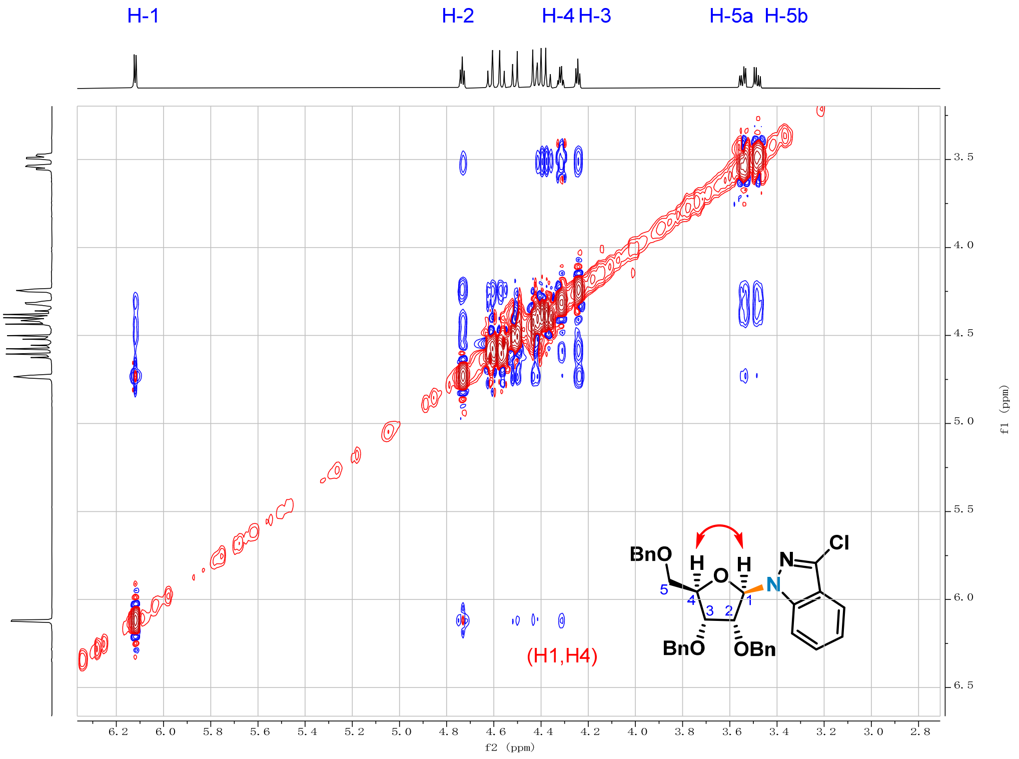

Supplementary Figure 15 | NOESY of **8f**

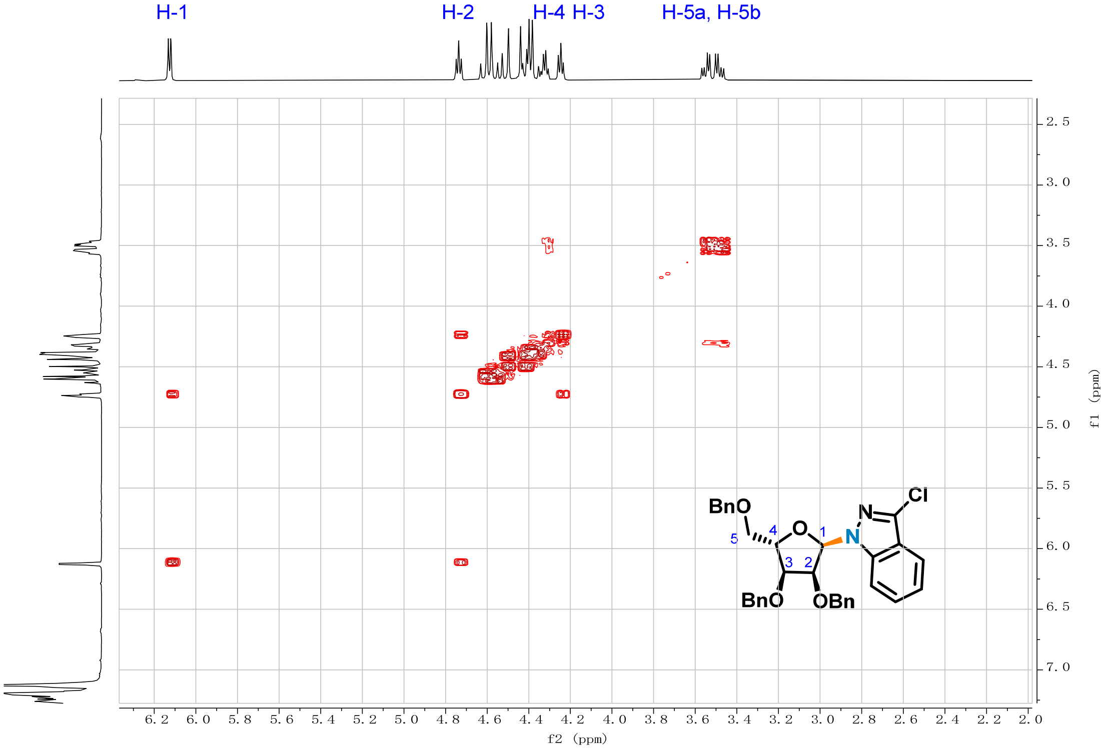

Supplementary Figure 16 | ^{1}H-^{1}H-COSY of **8h**

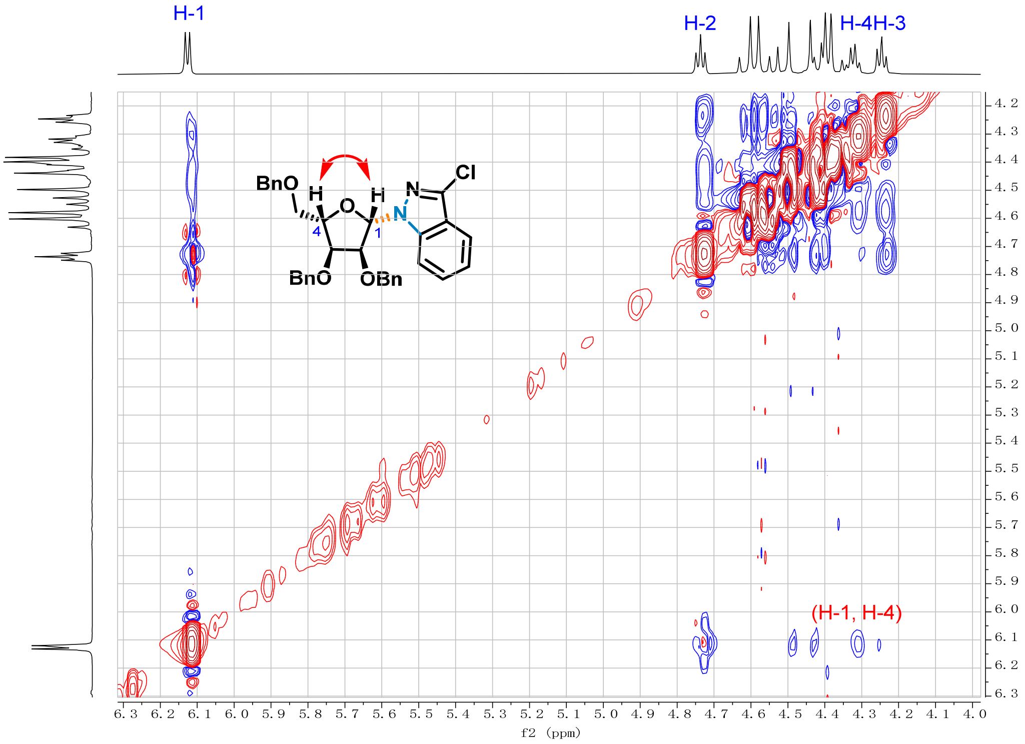

Supplementary Figure 17 | NOESY of **8h**

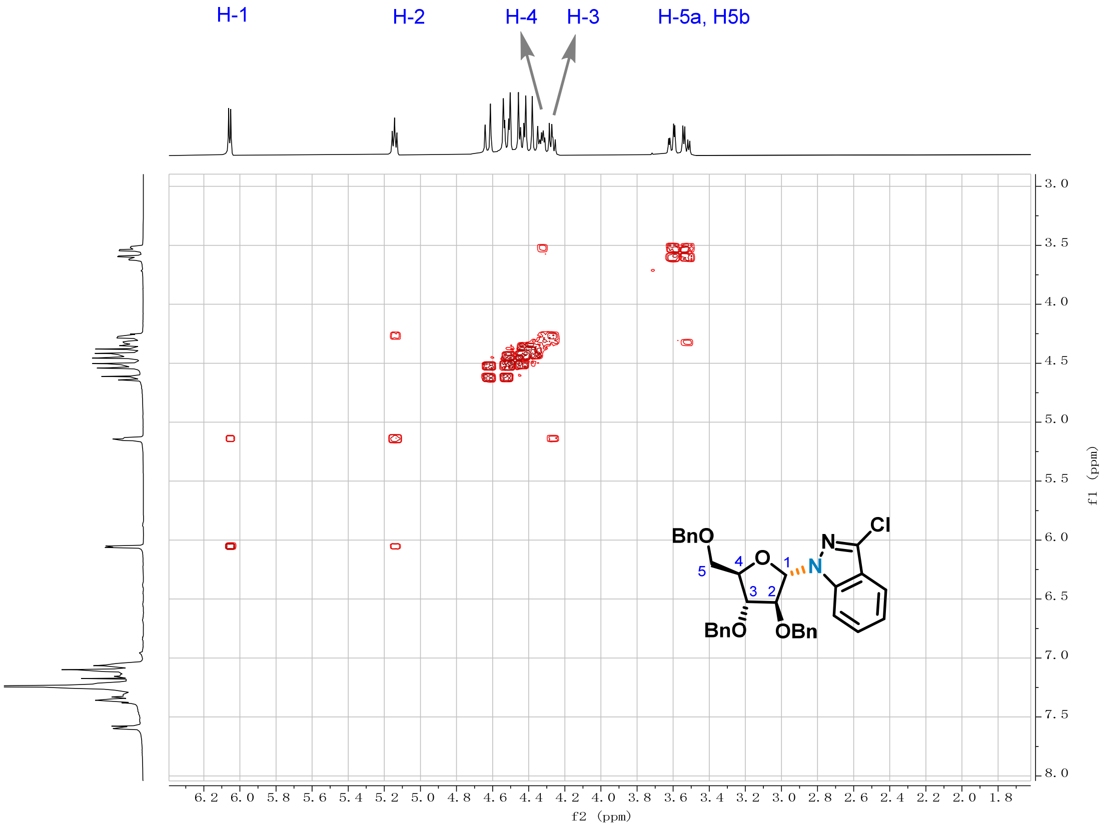

Supplementary Figure 18 | ^{1}H-^{1}H-COSY of **8i**

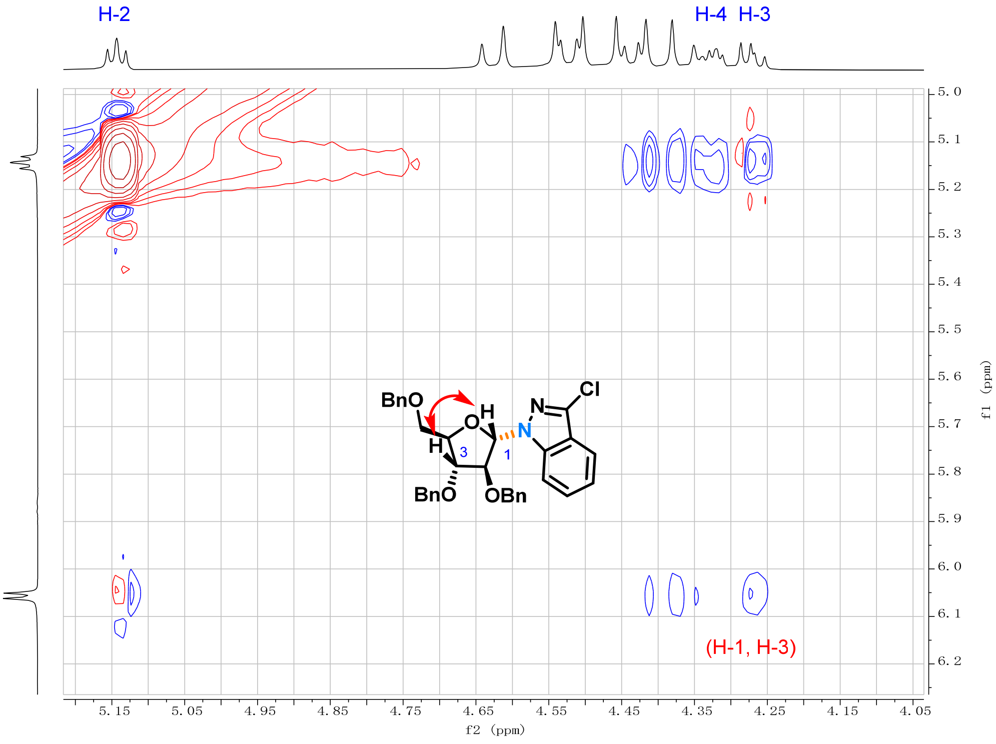

Supplementary Figure 19 | NOESY of **8i**

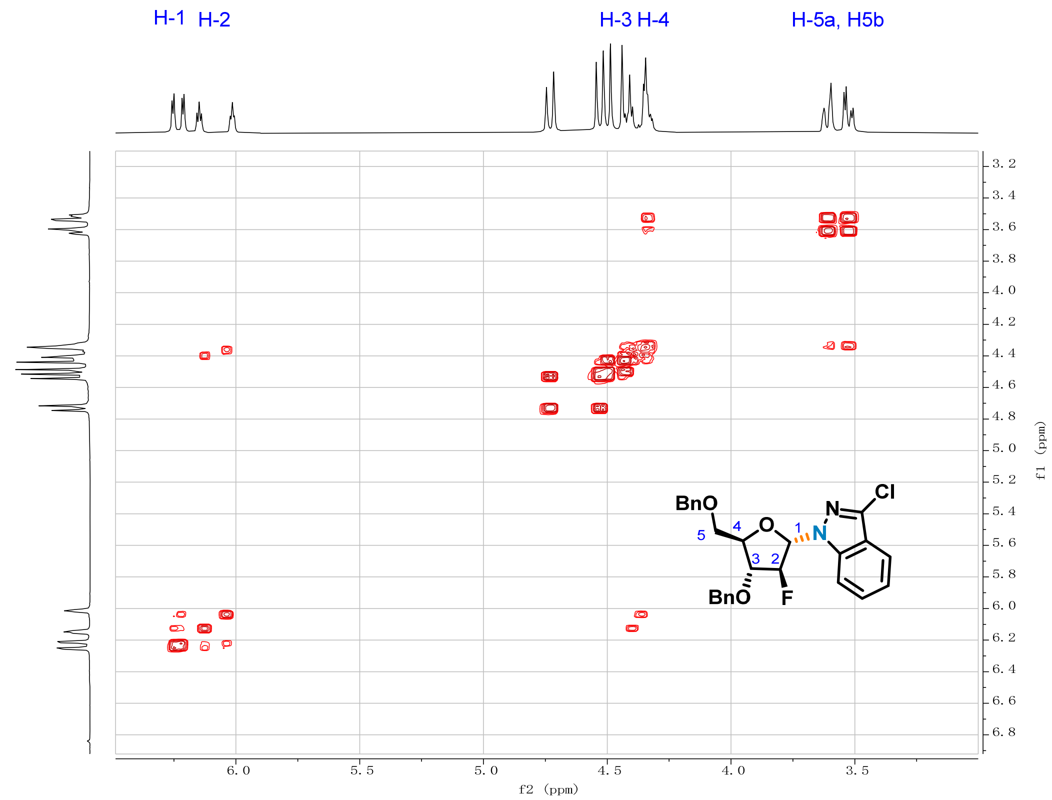

Supplementary Figure 20 | ^{1}H-^{1}H-COSY of **8j**

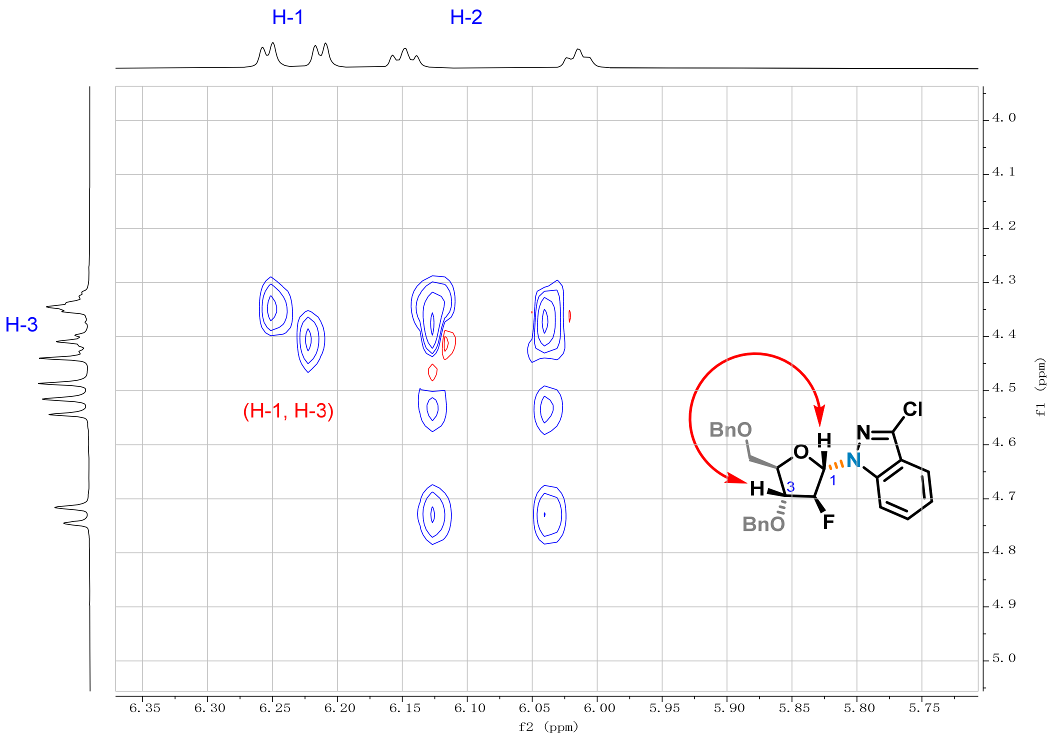

Supplementary Figure 21 | NOESY of **8j**

NOESY of **8ka**

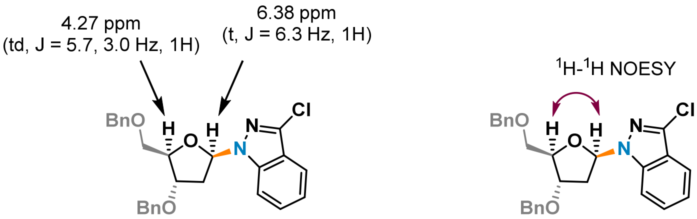

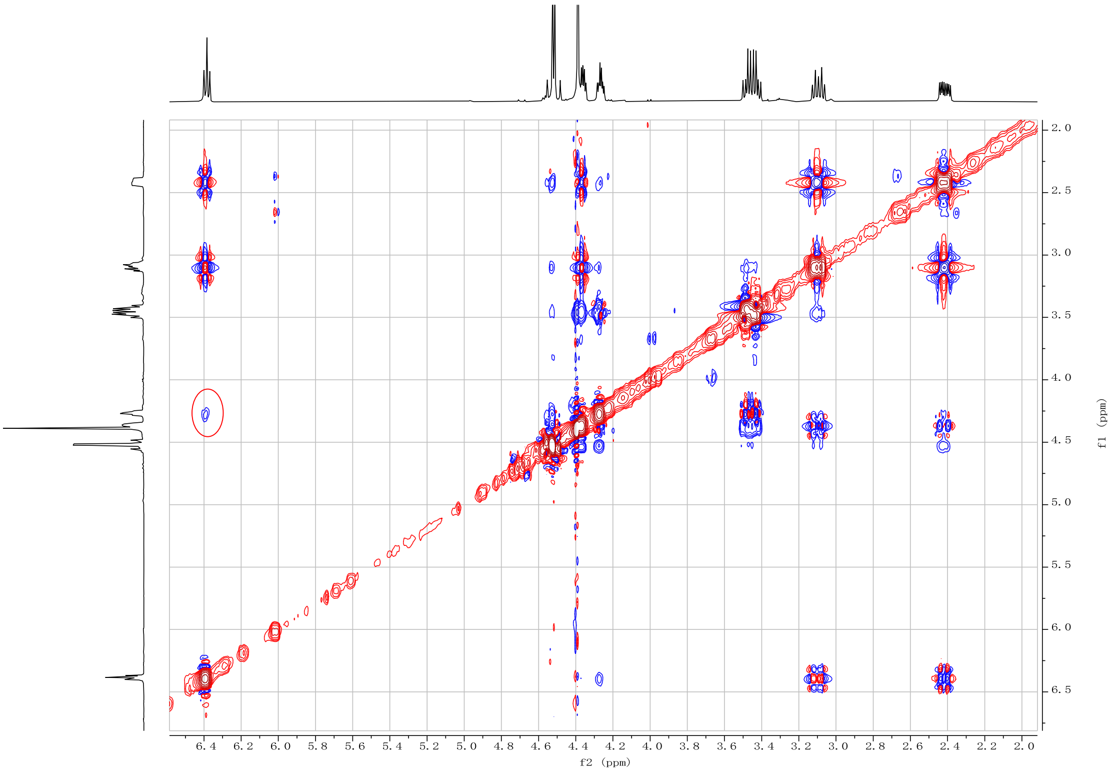

Supplementary Figure 22 | NOESY of **8ka**

Anomeric stereochemistry of **8o** determined by X-Ray

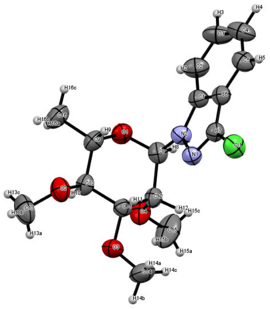

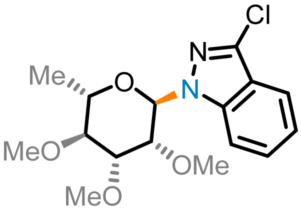

8o

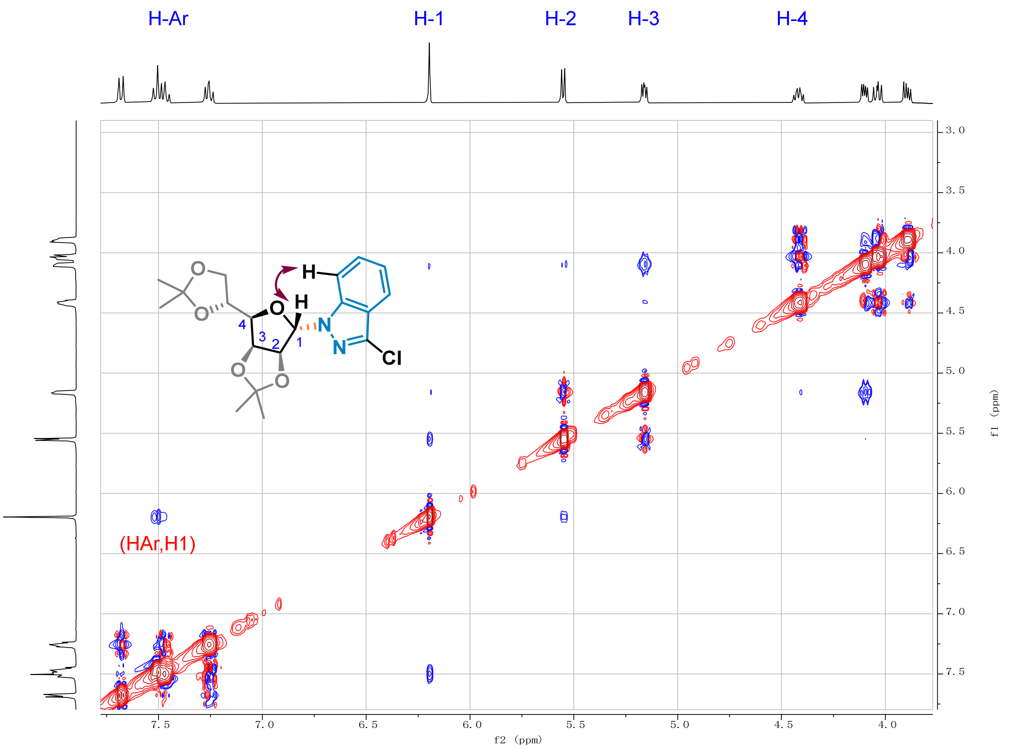

Supplementary Figure 23 | NOESY of **2a**

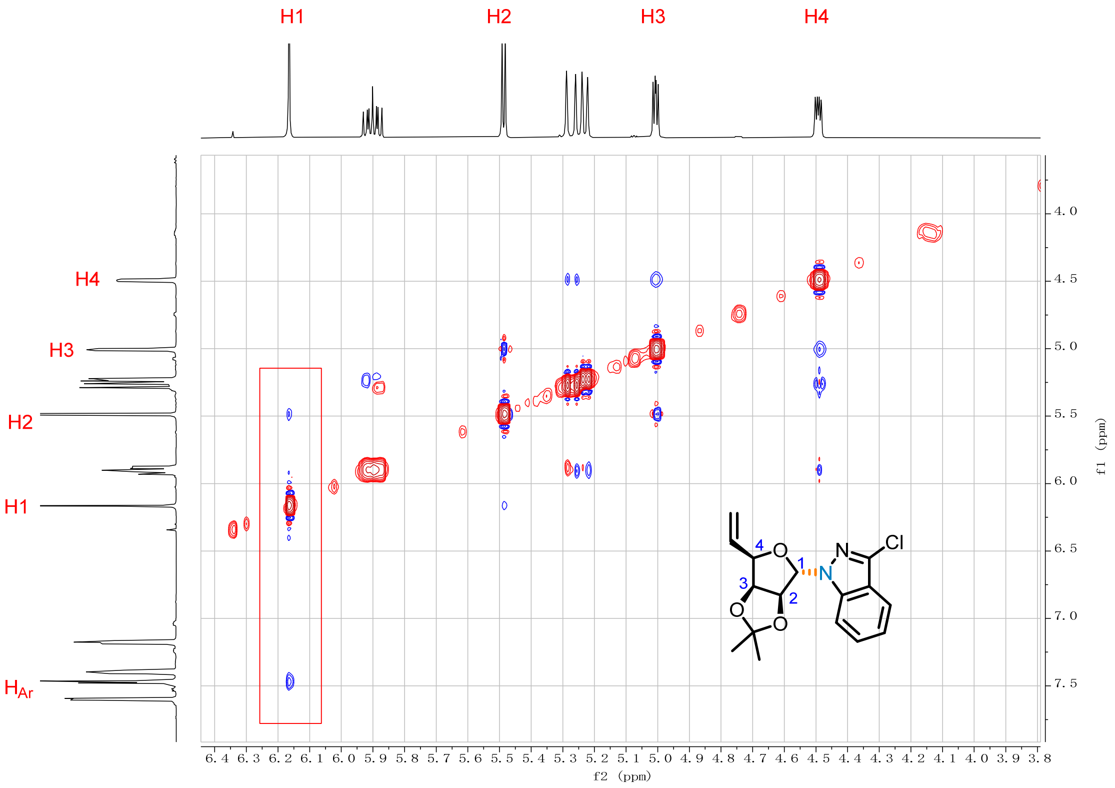

Supplementary Figure 24 | NOESY of **8l**

4.2 Determination of the regioisomer

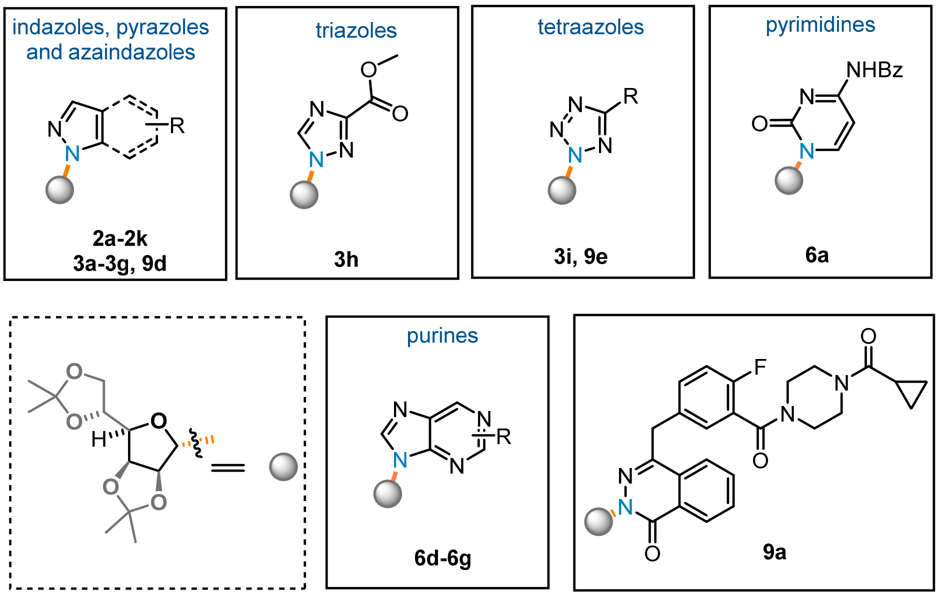

Supplementary Figure 25 | The types of N-htereocycle in this work

As shown above, there are 6 types of N-htereocycle in this paper that may yield regioisomers. To determine the regiochemistry, compound **2a**, **3h**, **3i, 6a**, **6e** and **9a** were selected for detailed NMR analyses. For **2a**, characteristic NOE signals between H1 and H_{Ar} were identified as shown in the following spectra. In addition, X-ray crystal structure data of **2a** was also obtained. For **3h**, C_{Ar1} have characteristic correlation with H_1 in HMBC. For **3i**, H1 have no correlation with H_{Ar} and C_{Ar} in both NOE and HMBC . For **6a**, we identified meaningful carbons and protons through 2D NMR, then we find C_{Ar5} have characteristic HMBC reference with H1. For **6e**, C_{ar8} was identified by its HMBC correlation with H1. Then, H_{ar8} and H_{ar2} could be assigned from C_{ar8}. From H_{ar8} and H_{ar2}, we then identified C_{ar4} and C_{ar5}. Because H1 has strong correlation with C_{ar4} rather than C_{ar5}, **6e** was assigned as N9 isomer. For **9a**, C_{Ar1} has characteristic HMBC correlation with H1.

NOESY of **2a**

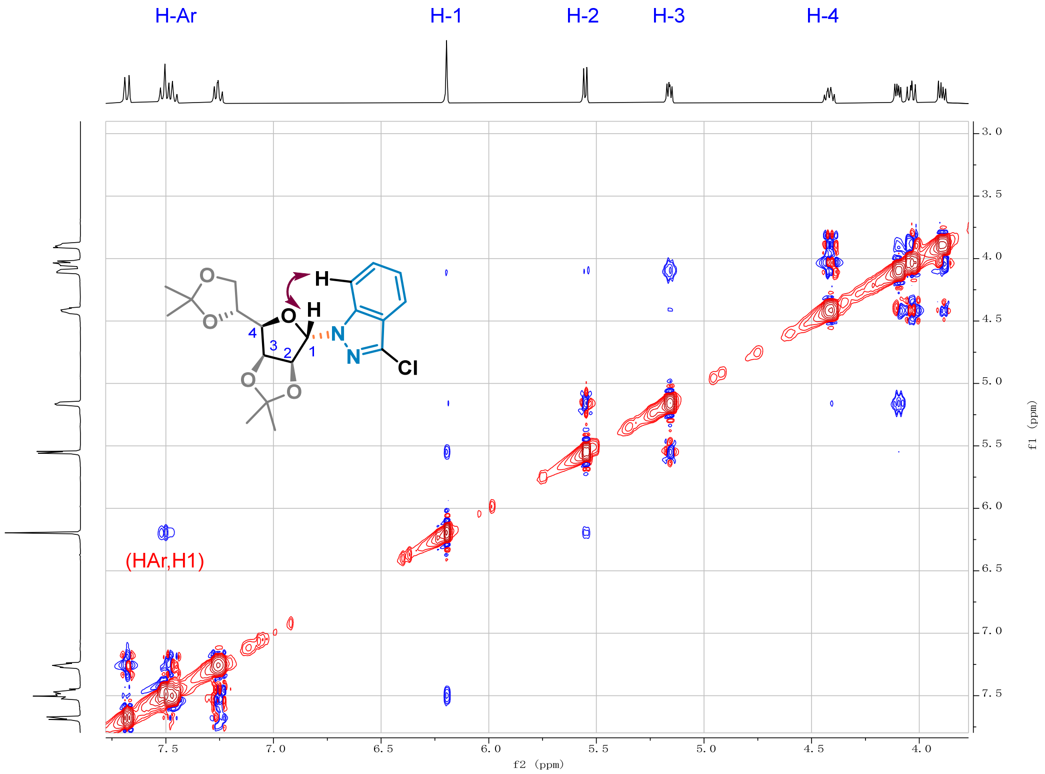

Supplementary Figure 26 | NOESY of **2a**

Regiochemistry of **2a** determined by X-ray

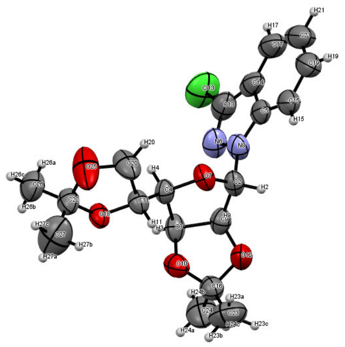

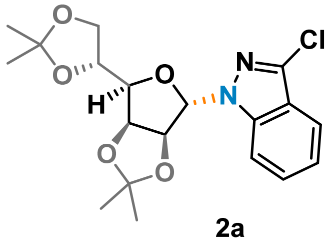

2a

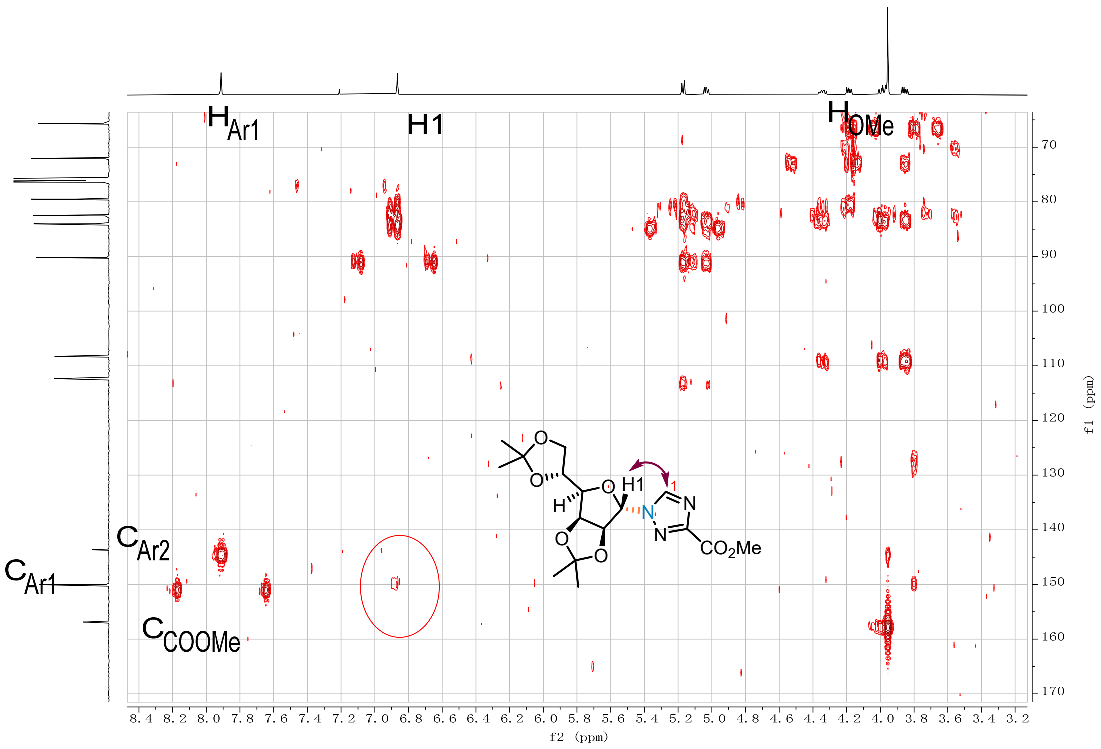

Supplementary Figure 27 | HMBC of **3h**

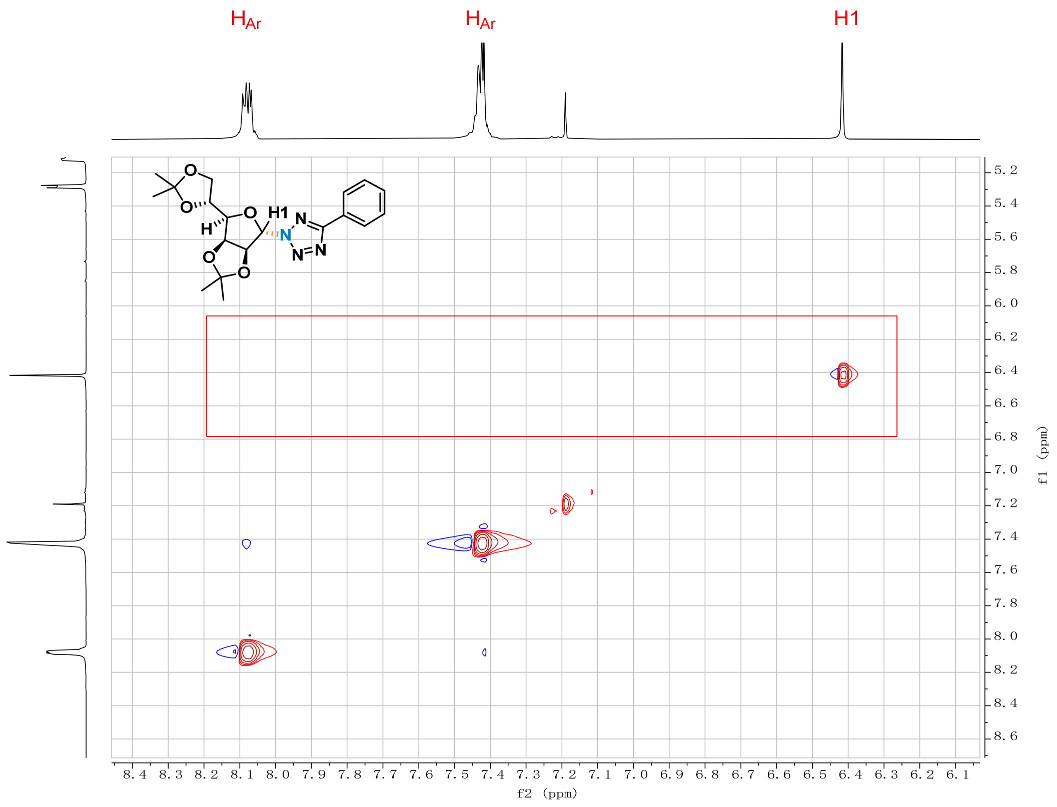

Supplementary Figure 28 | NOESY of **3i**

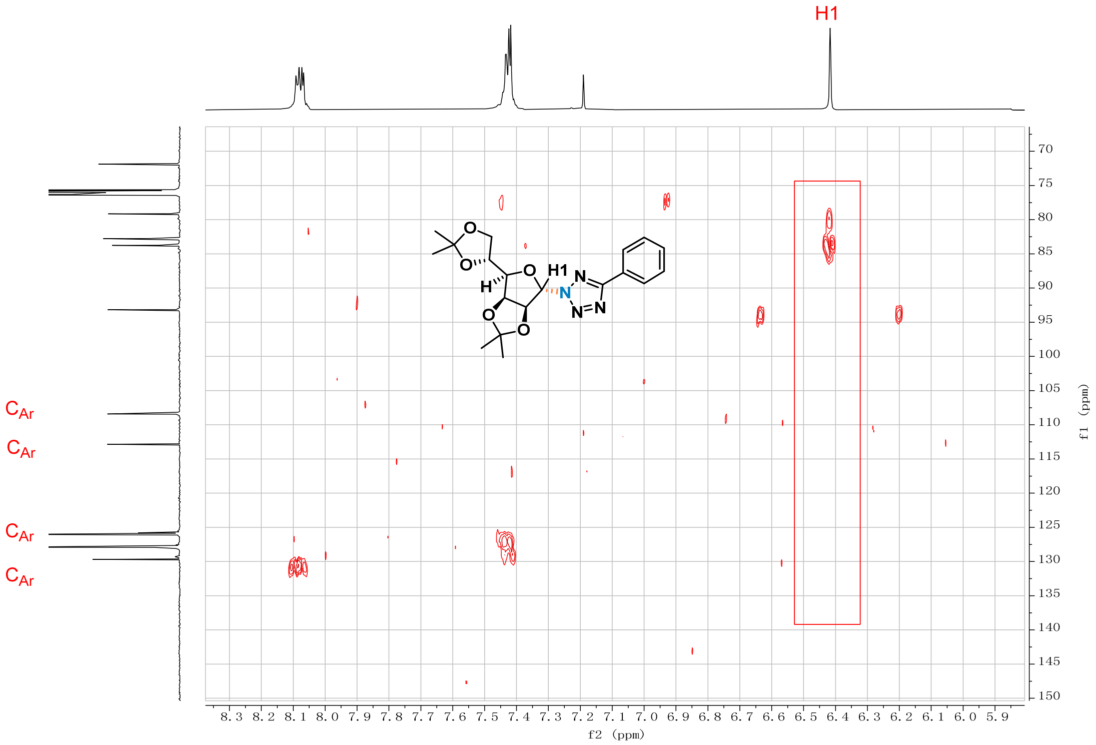

Supplementary Figure 29 | HMBC of **3i**

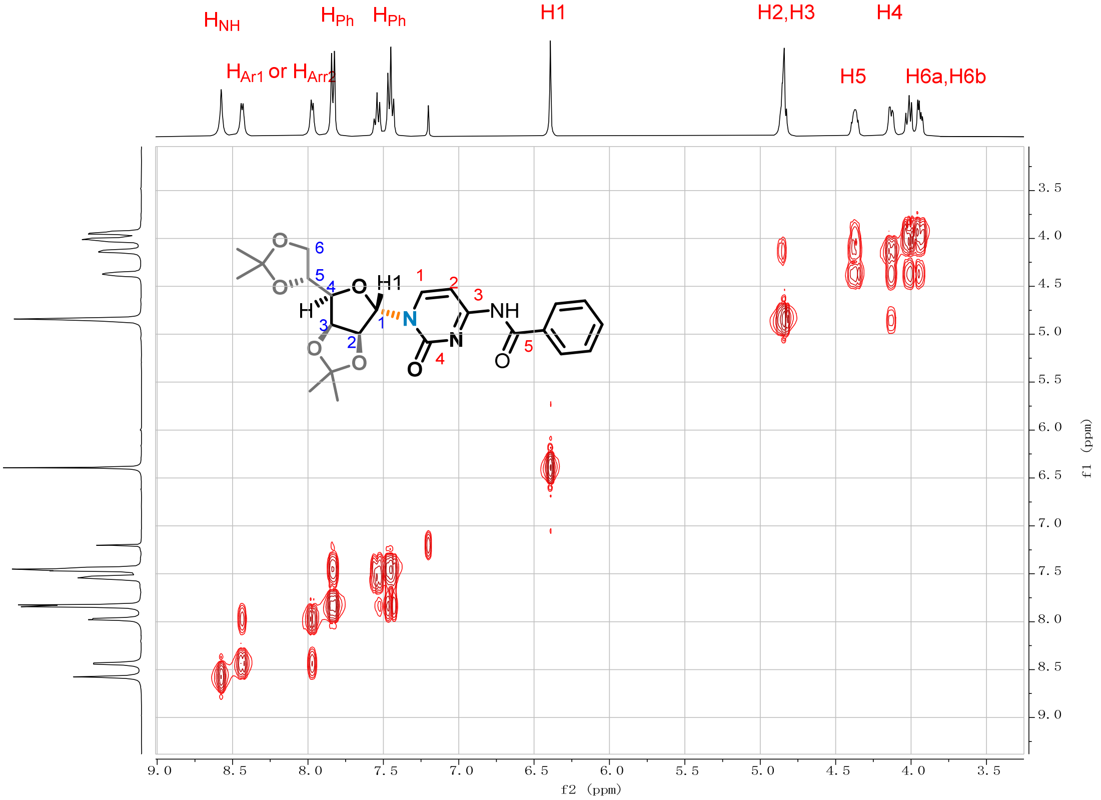

Supplementary Figure 30 | ^{1}H-^{1}H-COSY of **6a**

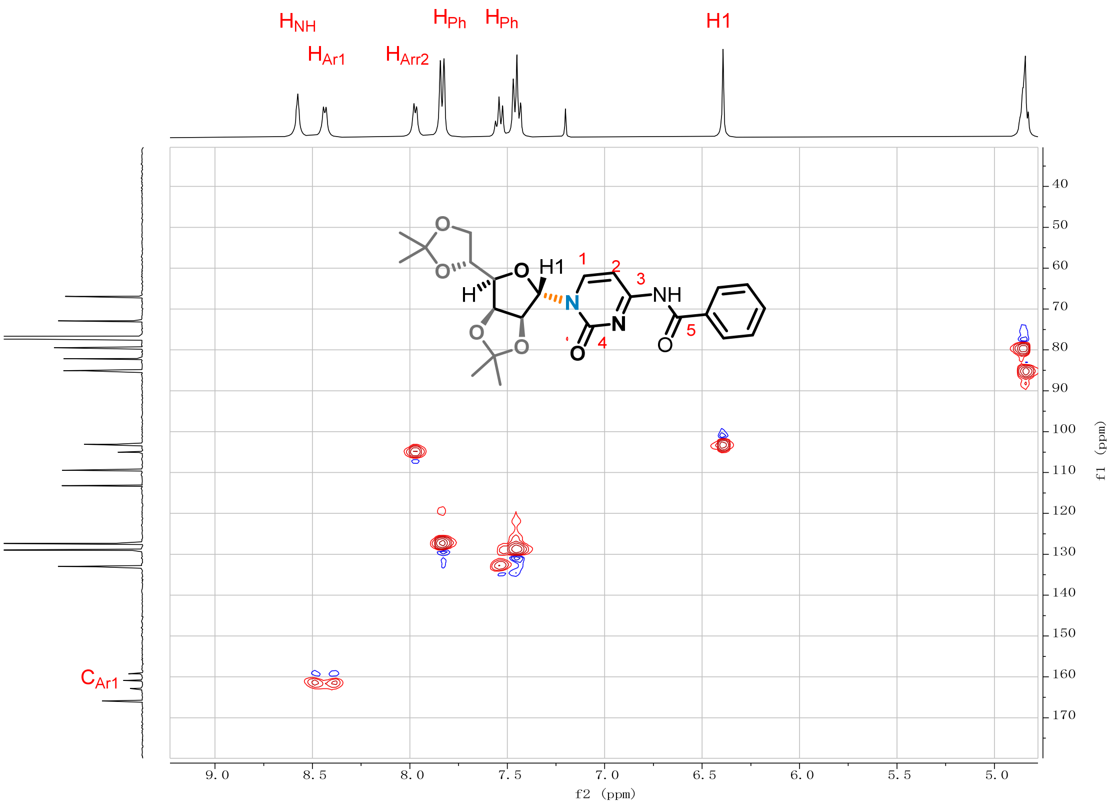

Supplementary Figure 31 | HSQC of **6a**

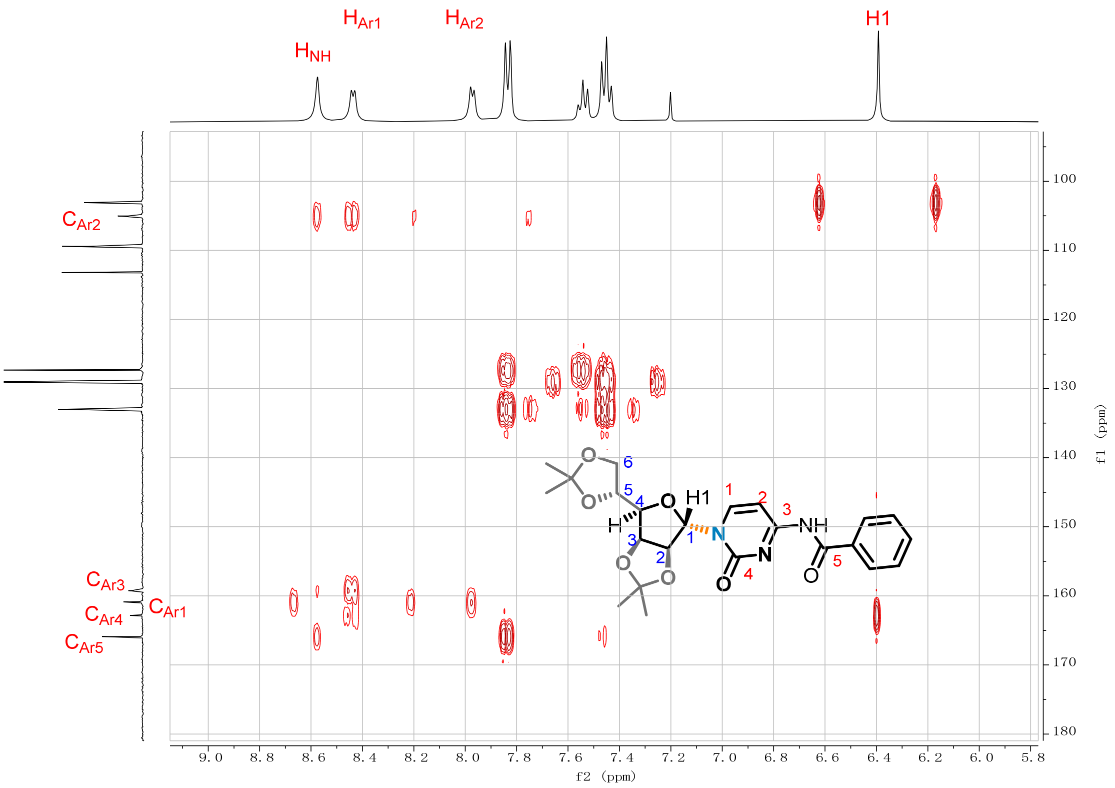

Supplementary Figure 32 | HMBC of **6a**

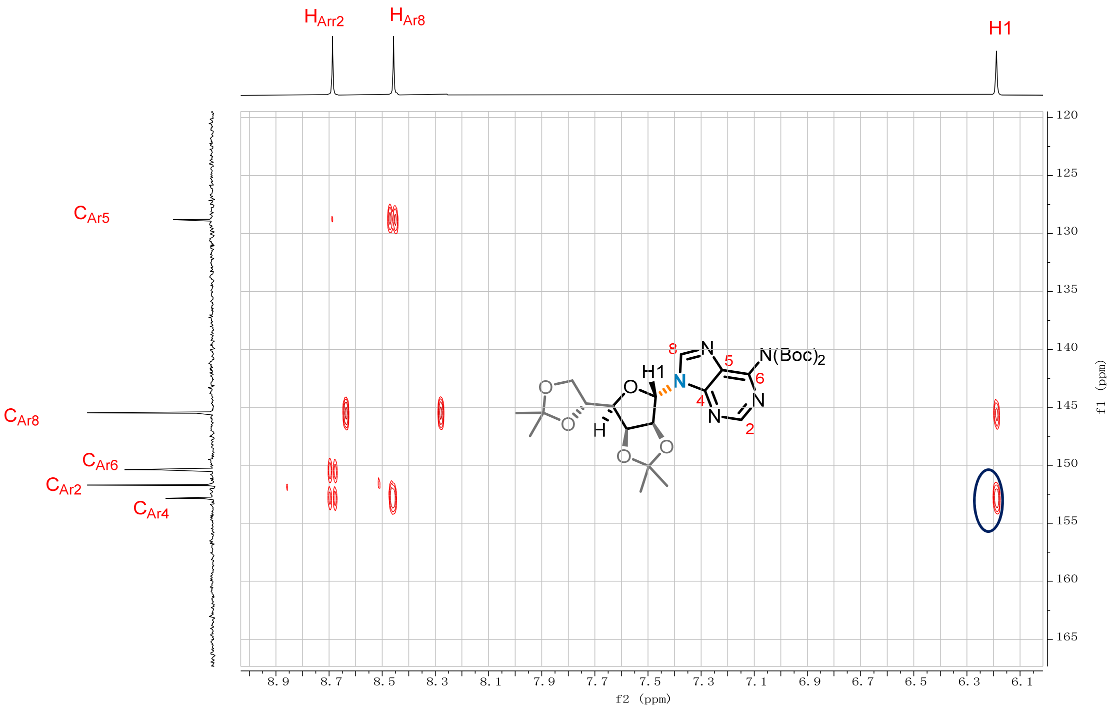

Supplementary Figure 33 | HMBC of **6e**

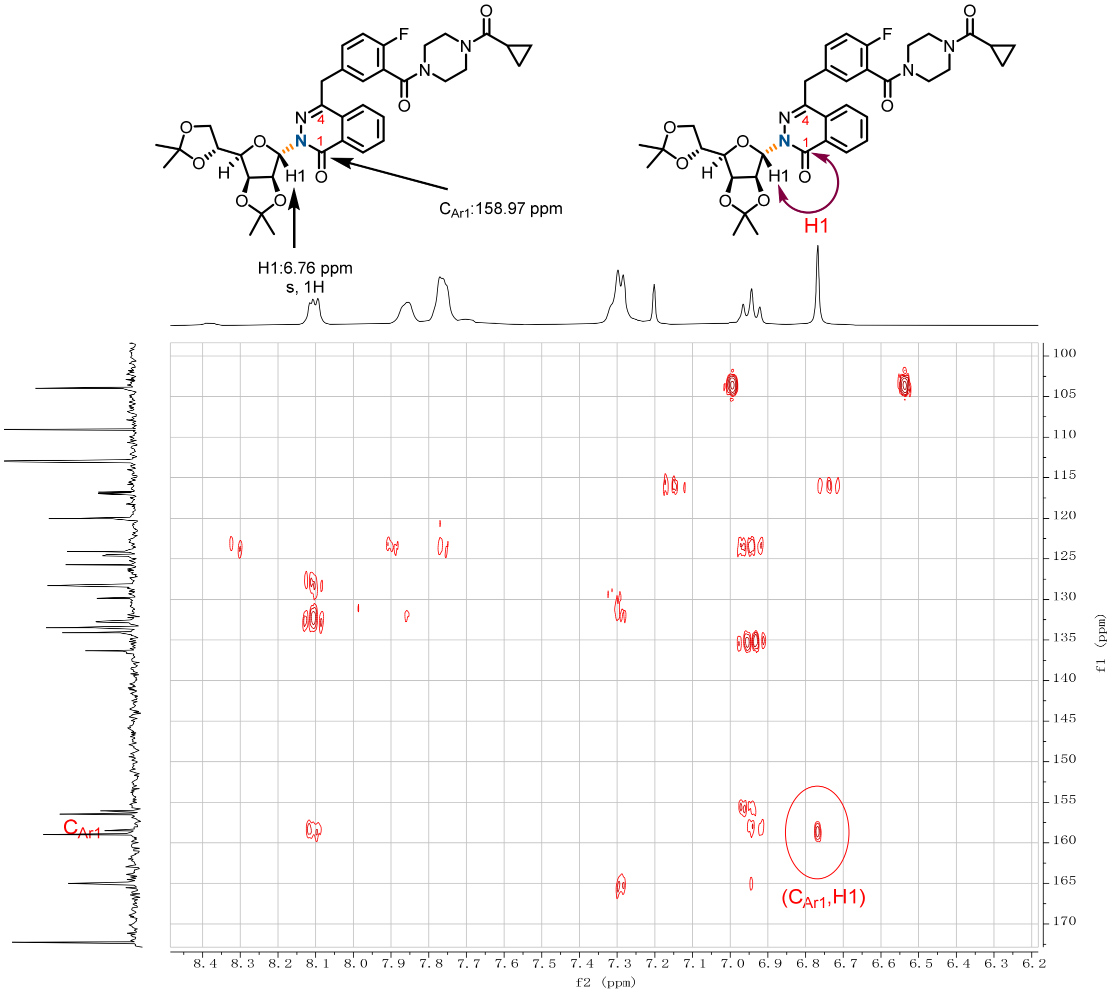

Supplementary Figure 34 | HMBC of **9a**

5. Copies of NMR spectra

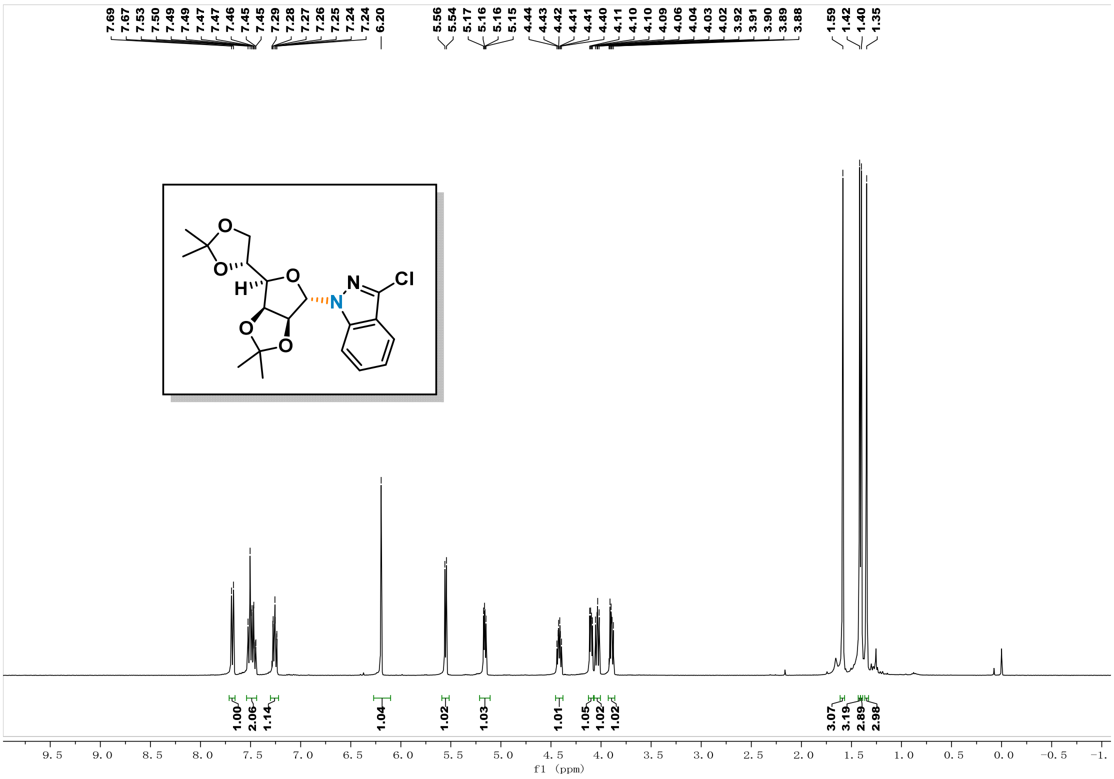

Supplementary Figure 35 | ^{1}H NMR (400 MHz, $CDCl_3$) **(2a)**

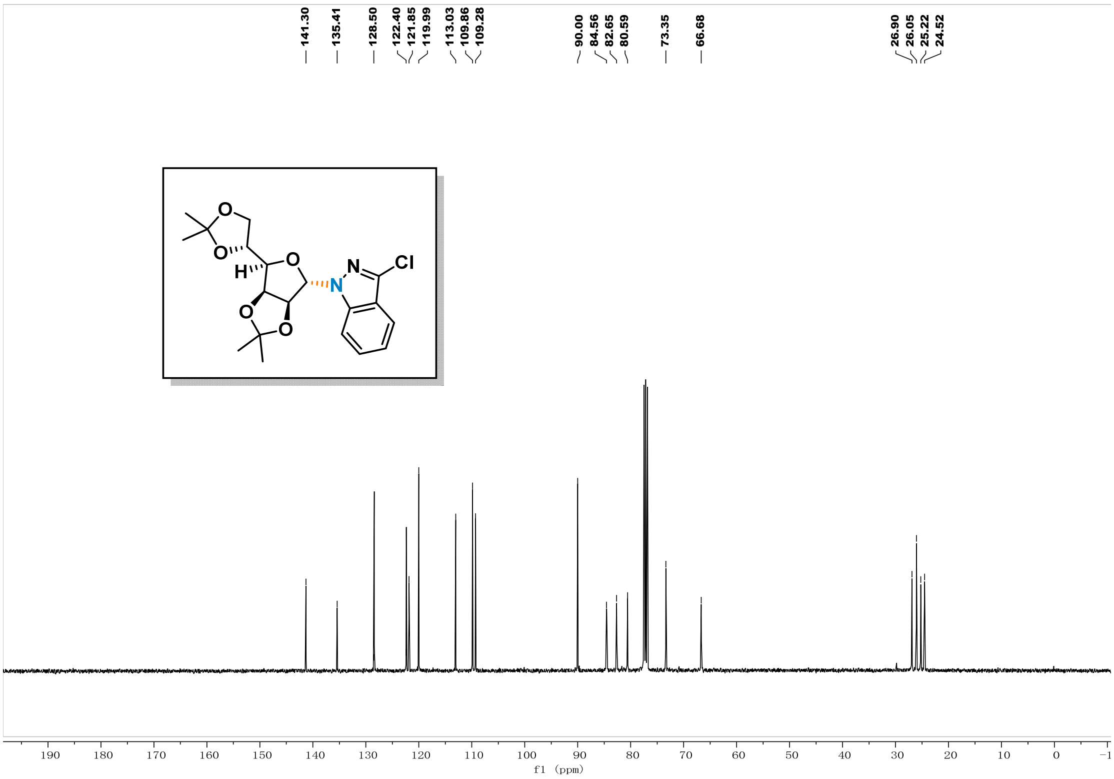

Supplementary Figure 36 | ^{13}C NMR (101 MHz, $CDCl_3$) **(2a)**

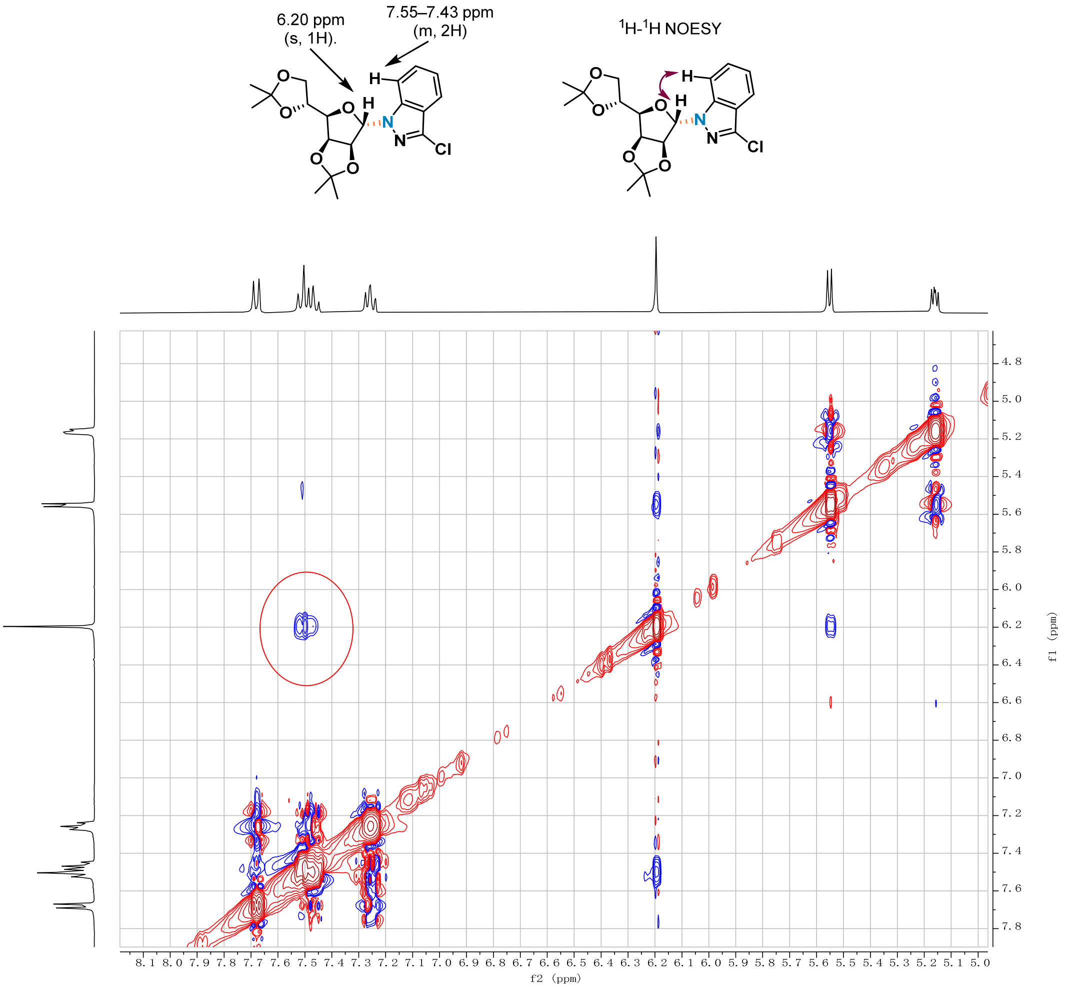

Supplementary Figure 37 | NOESY of **2a**

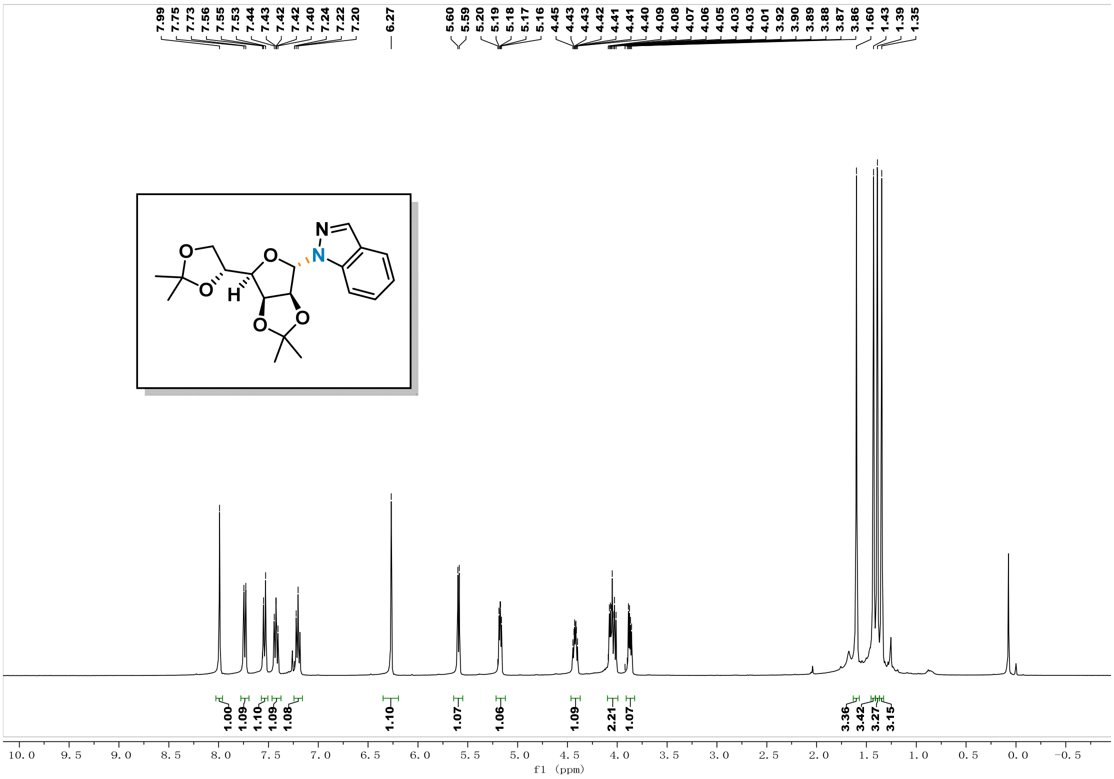

Supplementary Figure 38 | ^{1}H NMR (400 MHz, $CDCl_3$) **(2b)**

^{13}C NMR (101 MHz, $CDCl_3$) **(2b)**

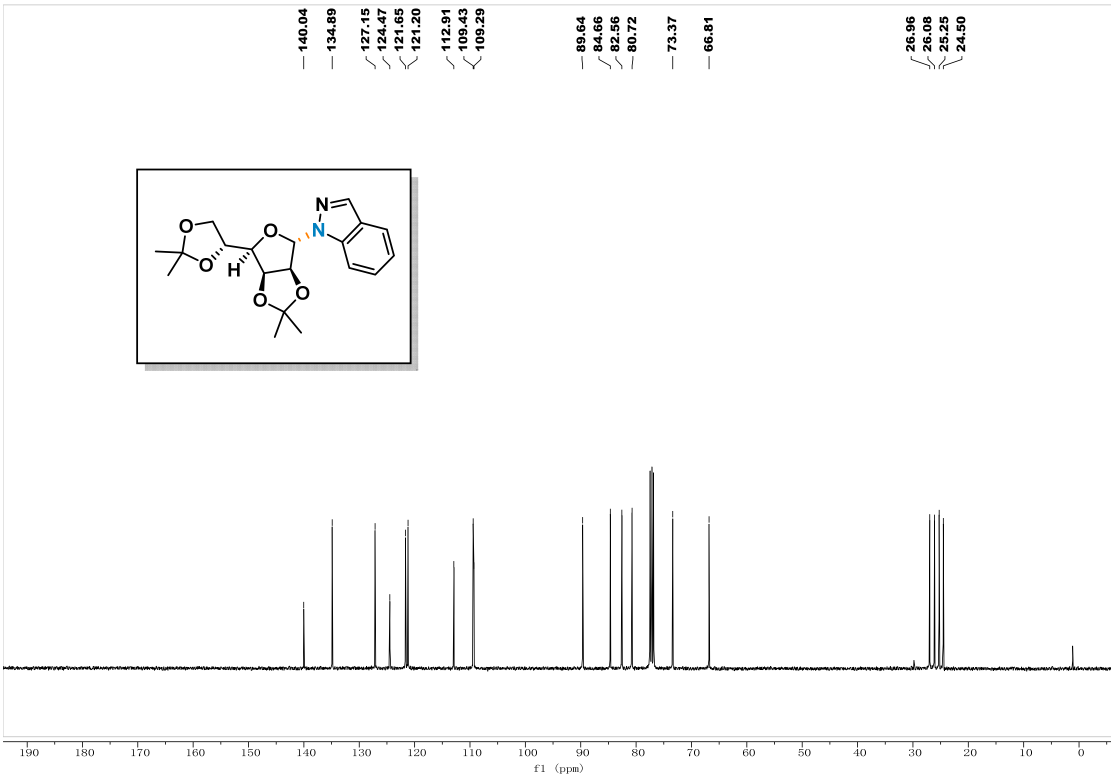

Supplementary Figure 39 | ^{13}C NMR (101 MHz, $CDCl_3$) **(2b)**

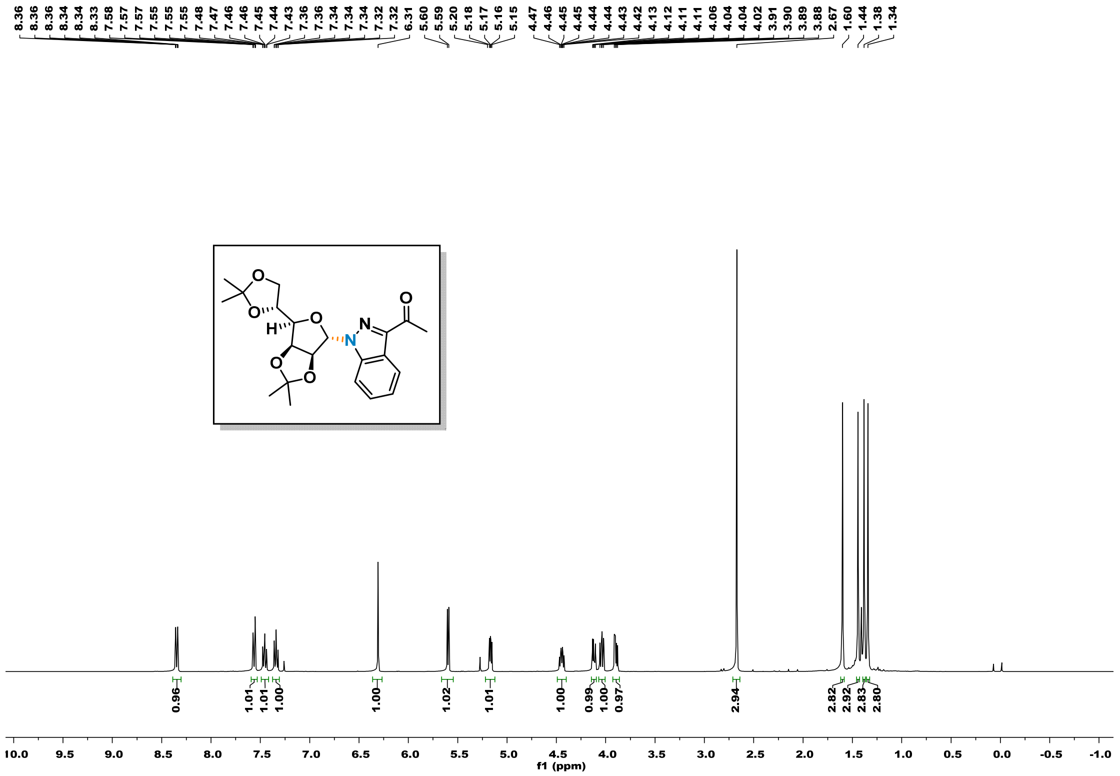

Supplementary Figure 40 | ^{1}H NMR (400 MHz, $CDCl_3$) **(2c)**

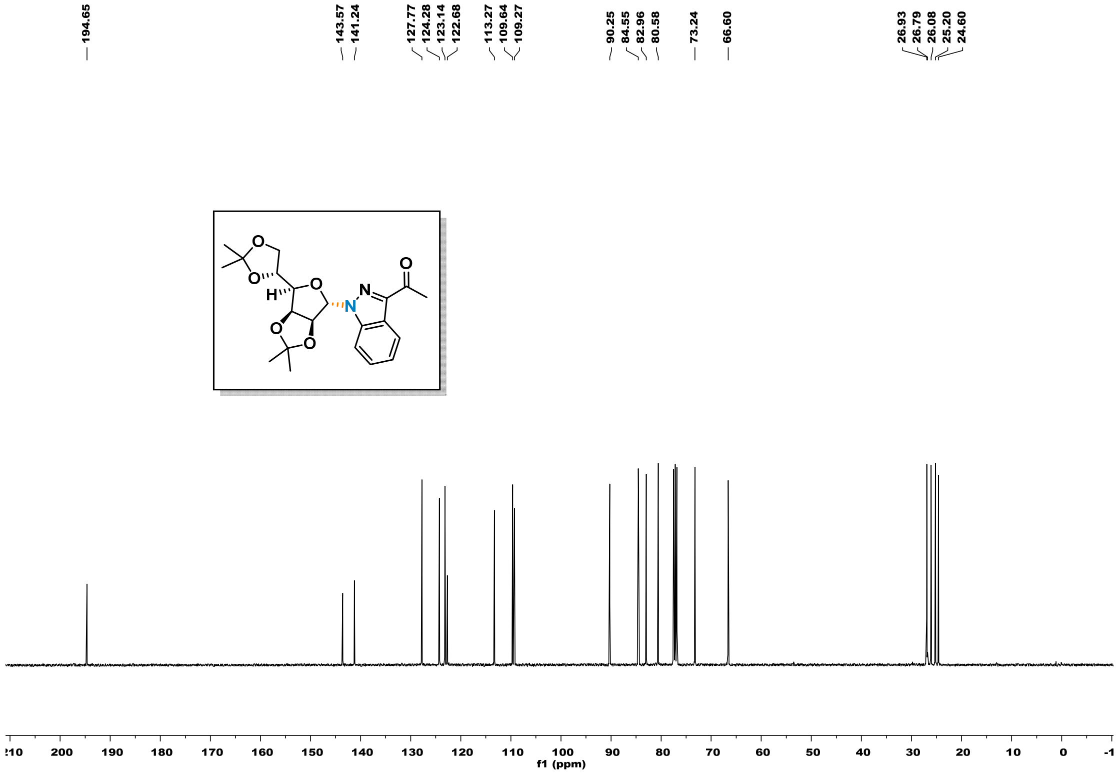

Supplementary Figure 41 | ^{13}C NMR (101 MHz, $CDCl_3$) **(2c)**

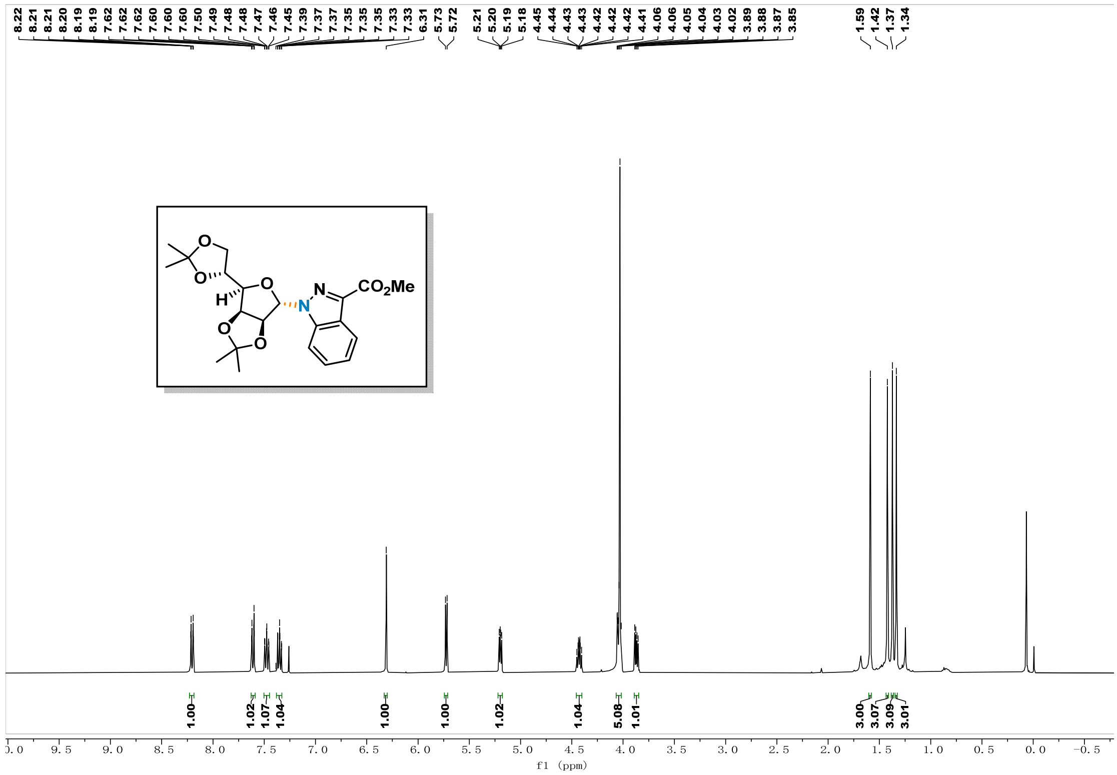

Supplementary Figure 42 | ^{1}H NMR (400 MHz, $CDCl_3$) **(2d)**

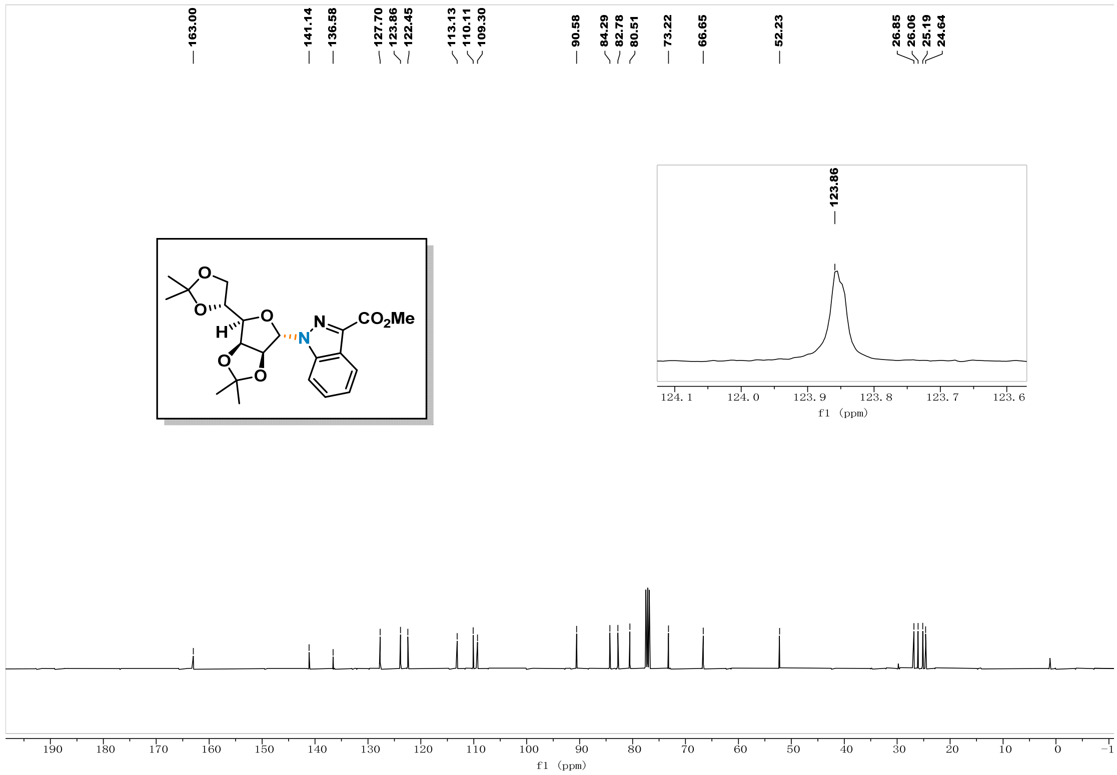

Supplementary Figure 43 | ^{13}C NMR (101 MHz, $CDCl_3$) **(2d)**

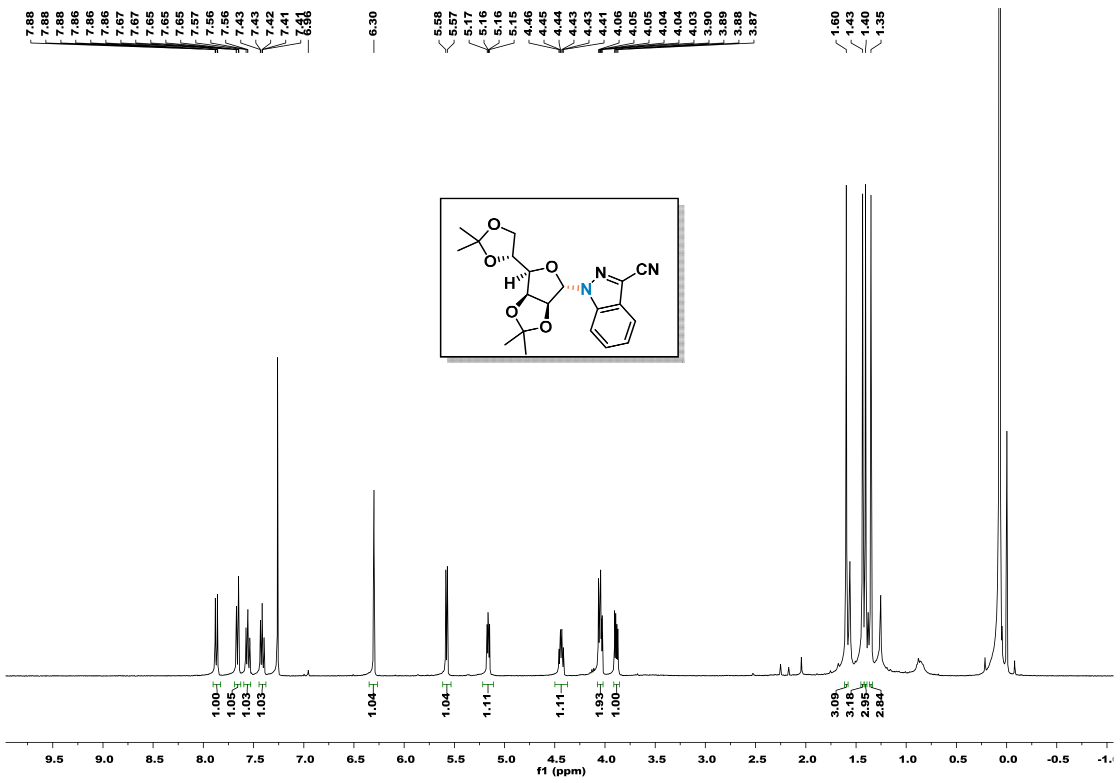

Supplementary Figure 44 | ^{1}H NMR (400 MHz, $CDCl_3$) **(2e)**

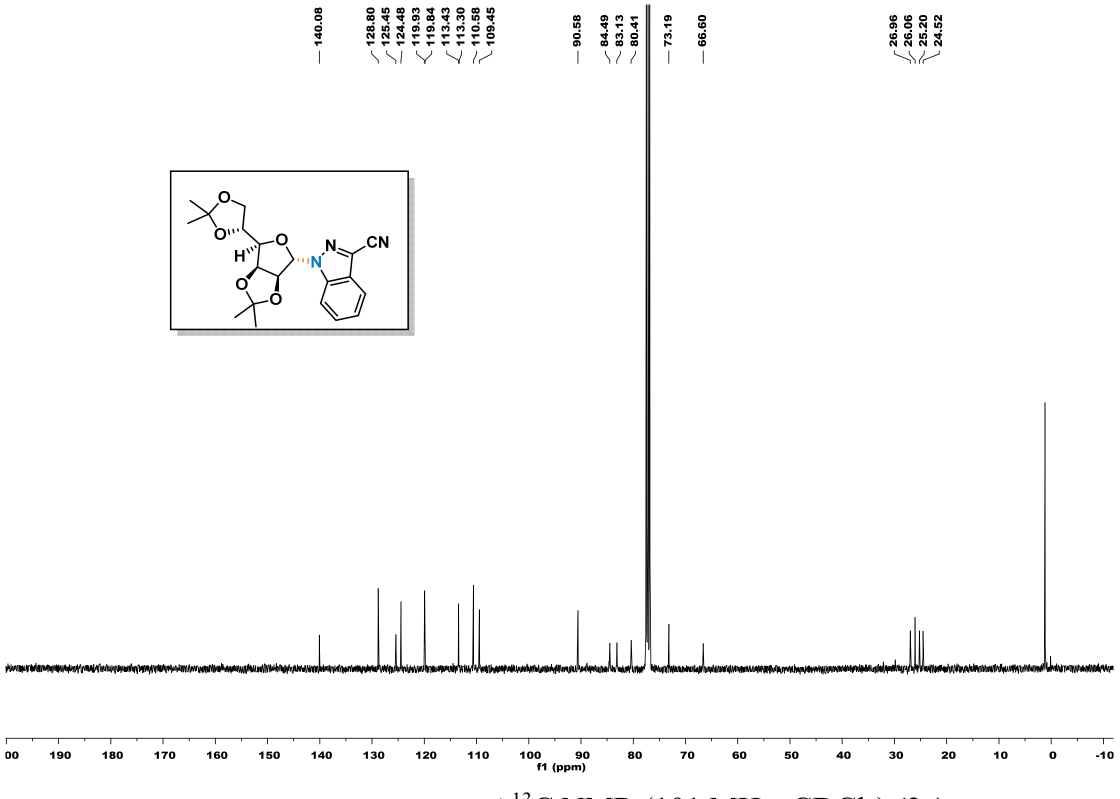

Supplementary Figure 45 | ^{13}C NMR (101 MHz, $CDCl_3$) **(2e)**

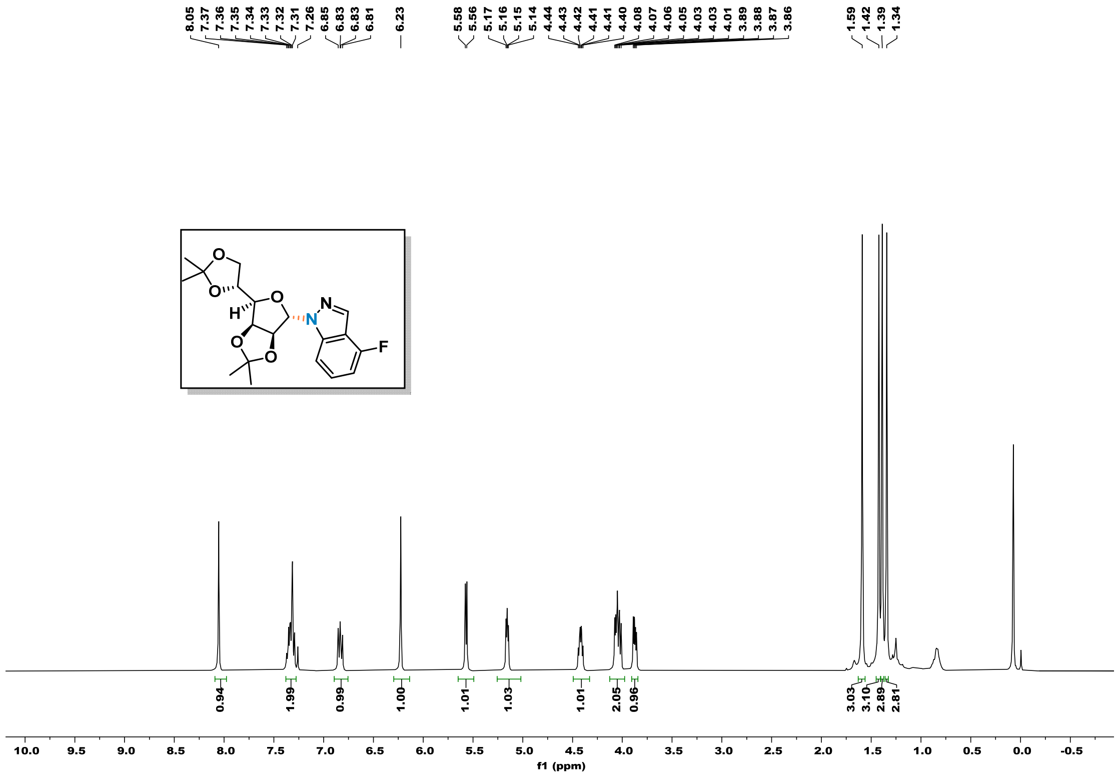

Supplementary Figure 46 | ^{1}H NMR (400 MHz, CDCl$_3$) **(2f)**

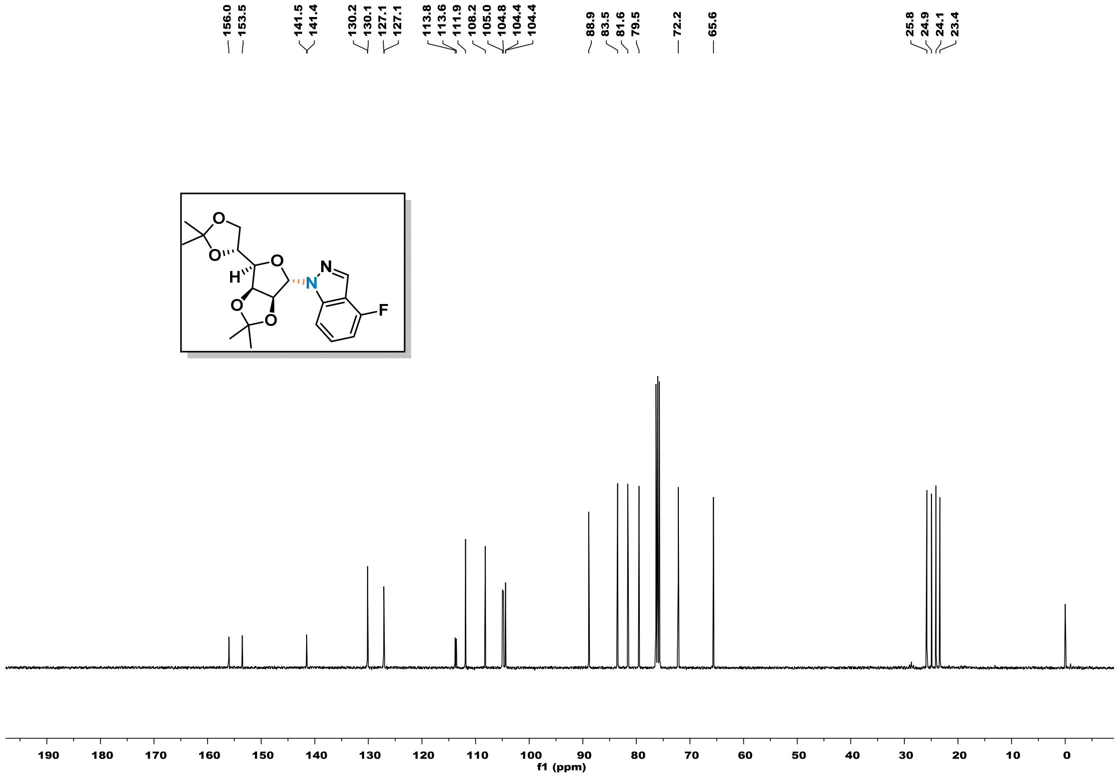

Supplementary Figure 47 | ^{13}C NMR (101 MHz, CDCl$_3$) **(2f)**

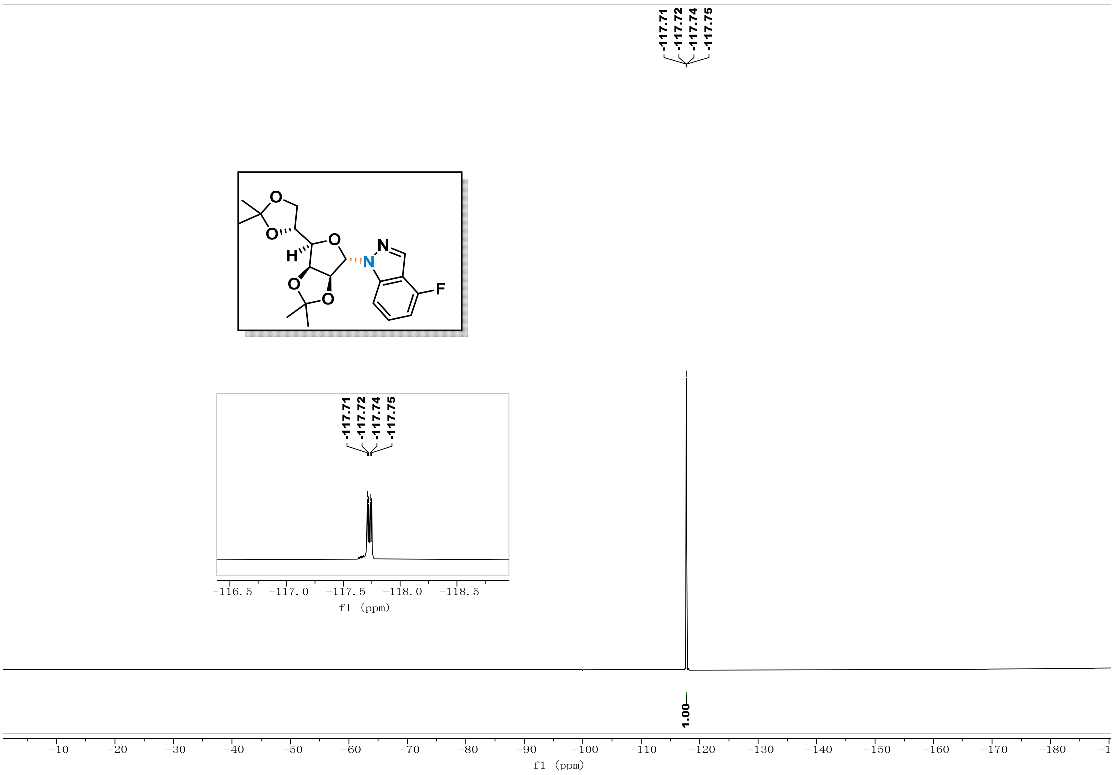

Supplementary Figure 48 | ^{19}F NMR (376 MHz, $CDCl_3$) (**2f**)

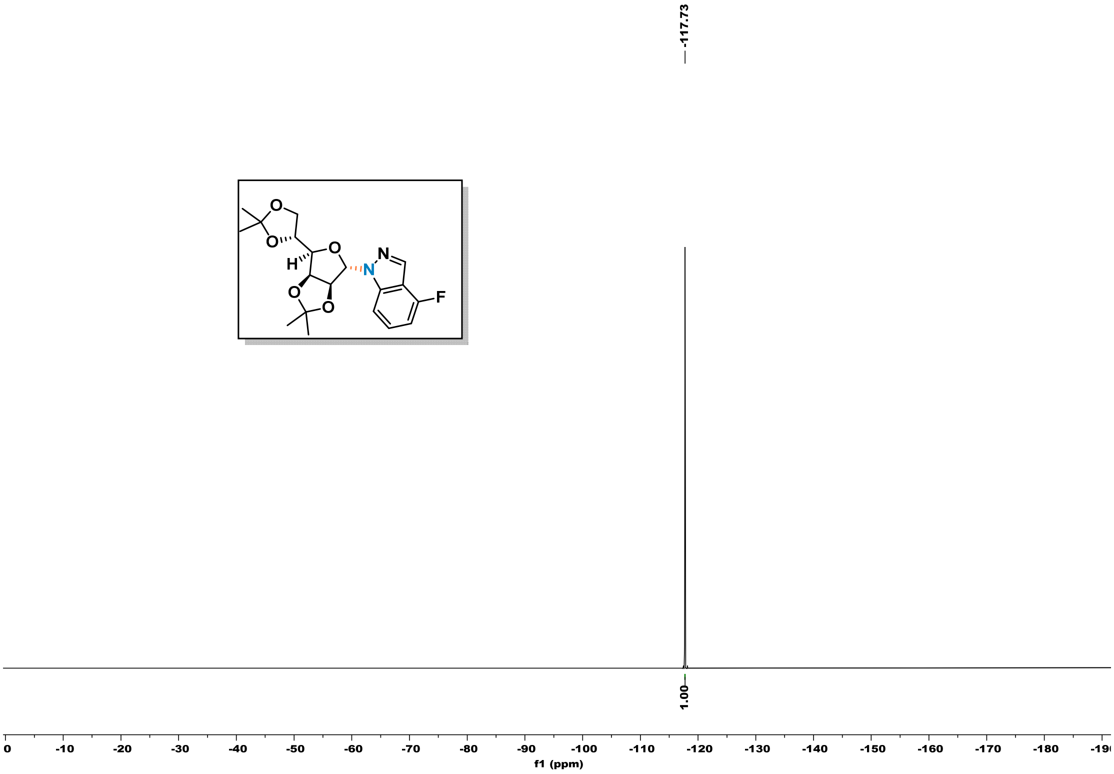

Supplementary Figure 49 | ^{19}F{H}NMR (376 MHz, $CDCl_3$) (**2f**)

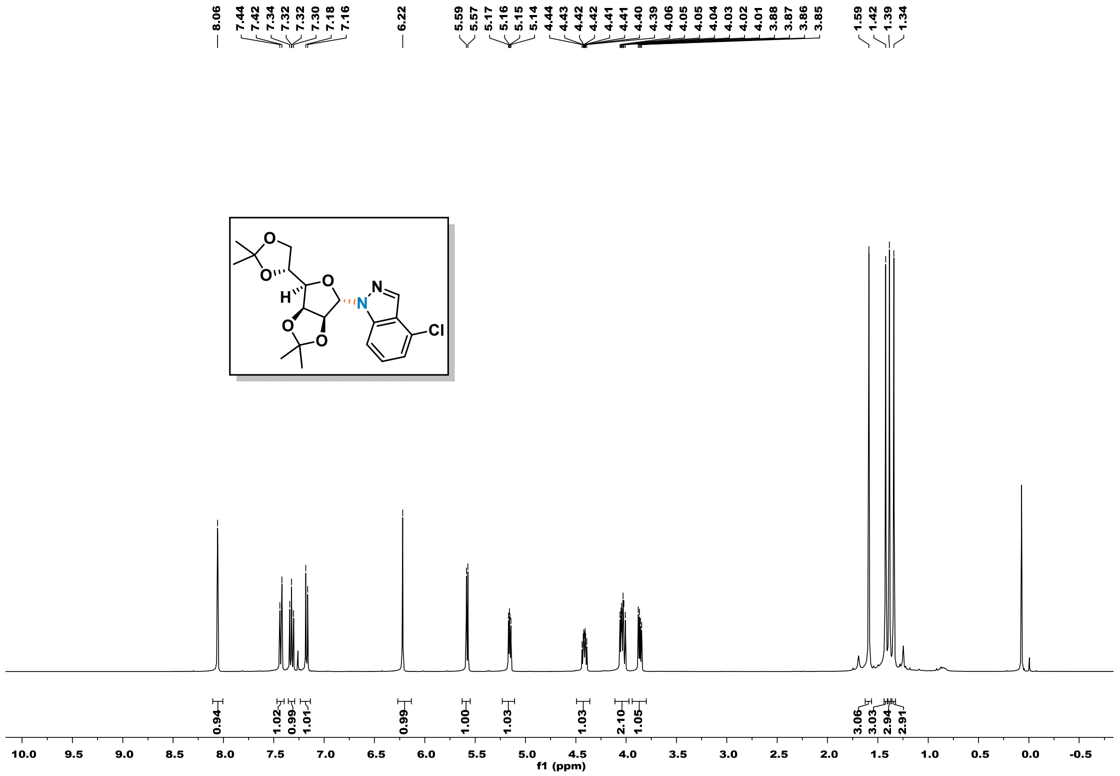

Supplementary Figure 50 | ^{1}H NMR (400 MHz, $CDCl_3$) **(2g)**

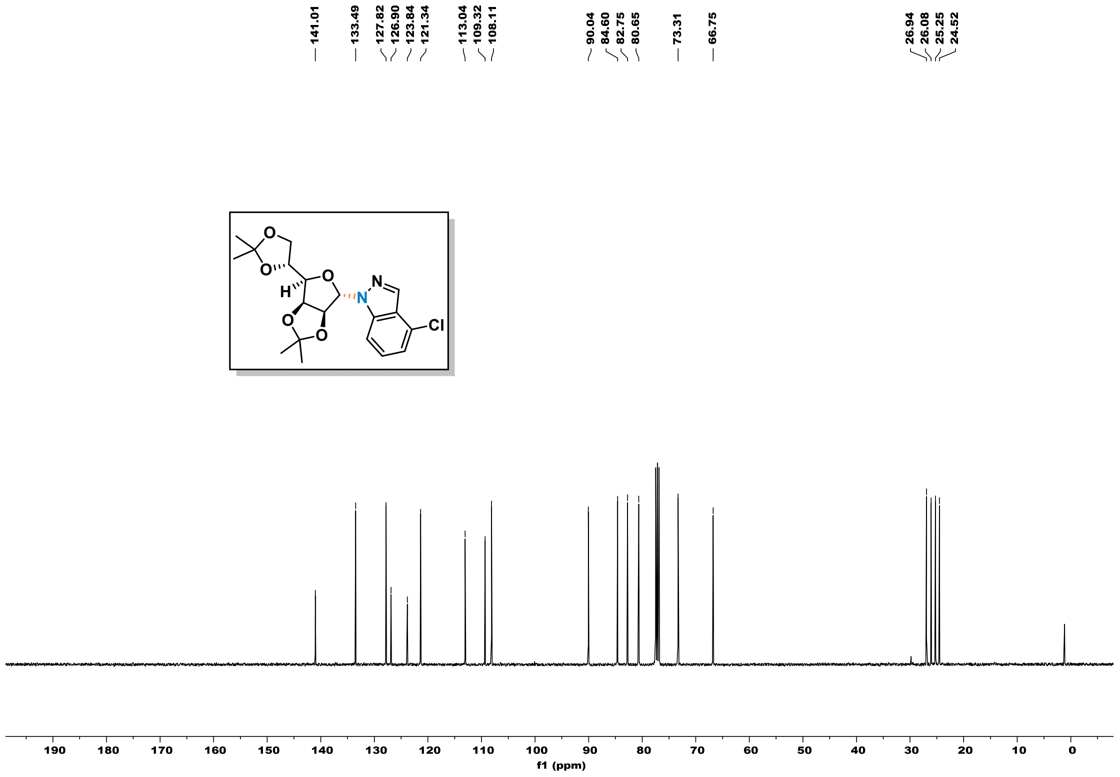

Supplementary Figure 51 | ^{13}C NMR (101 MHz, $CDCl_3$) **(2g)**

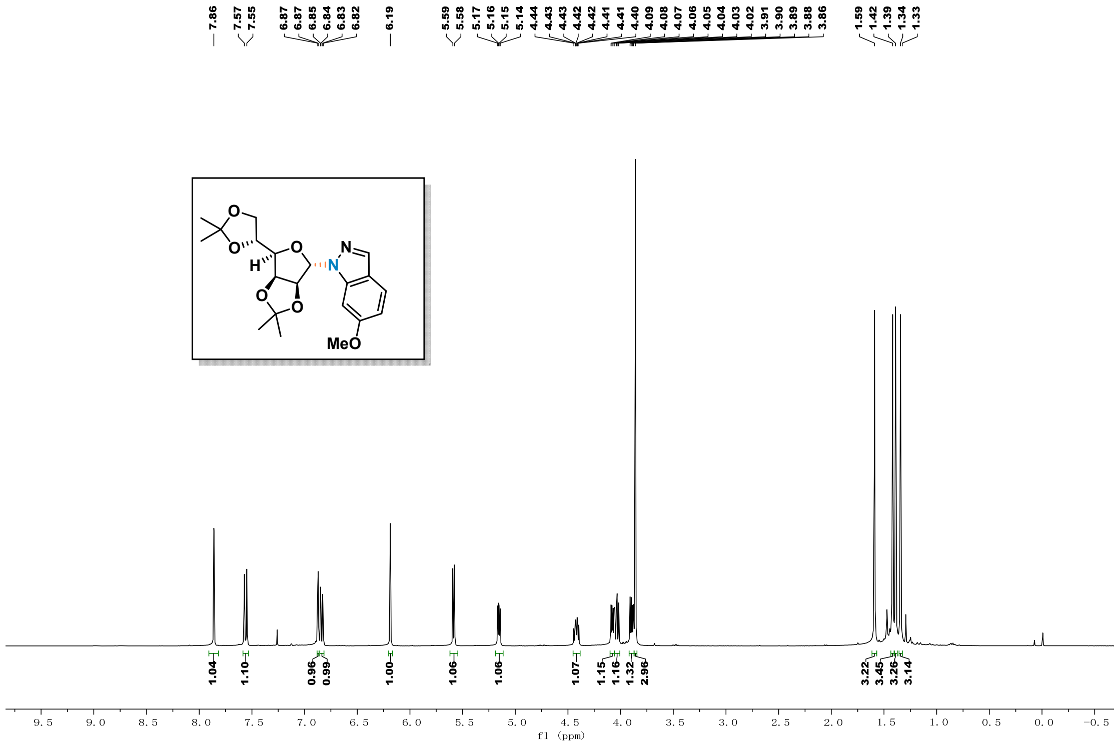

Supplementary Figure 52 | ^{1}H NMR (400 MHz, $CDCl_3$) **(2h)**

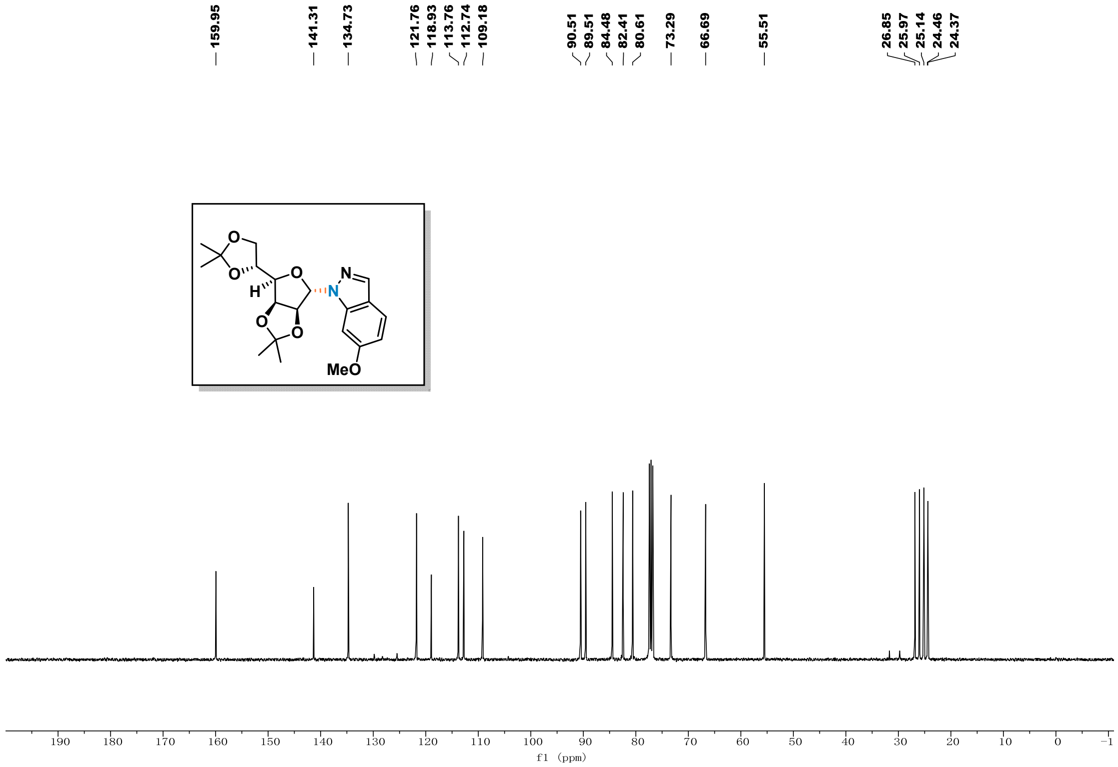

Supplementary Figure 53 | ^{13}C NMR (101 MHz, $CDCl_3$) **(2h)**

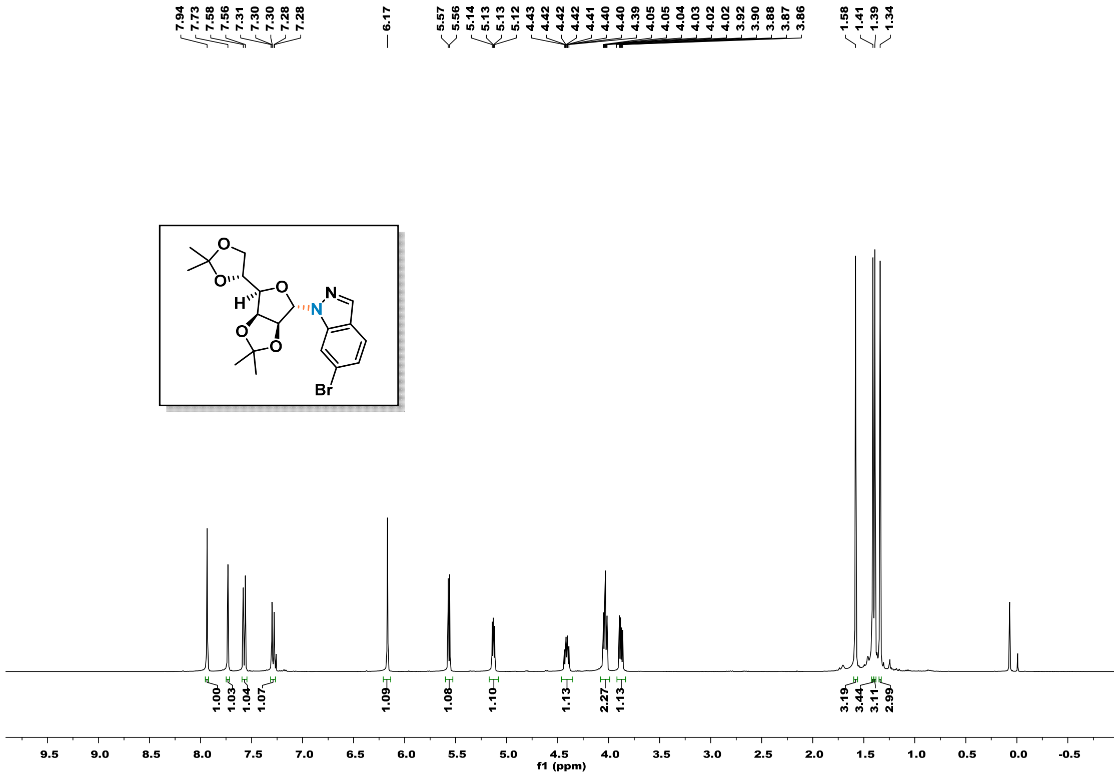

Supplementary Figure 54 | ^{1}H NMR (400 MHz, $CDCl_3$) **(2i)**

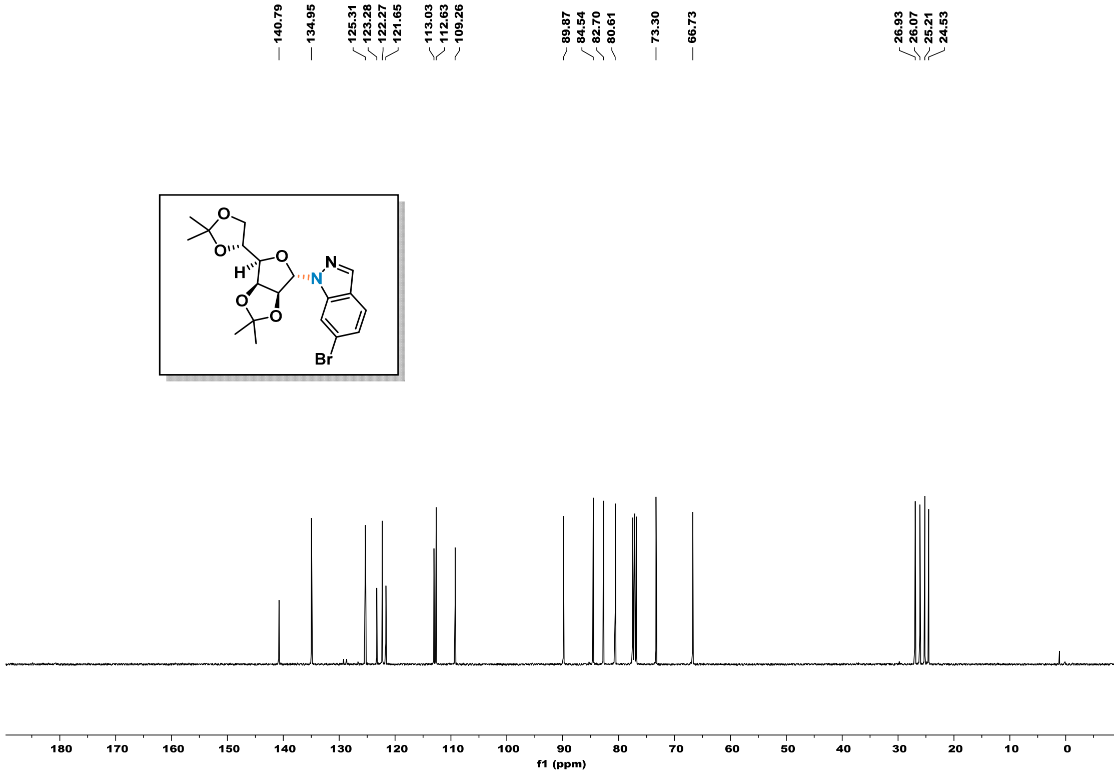

Supplementary Figure 55 | ^{13}C NMR (101 MHz, $CDCl_3$) **(2i)**

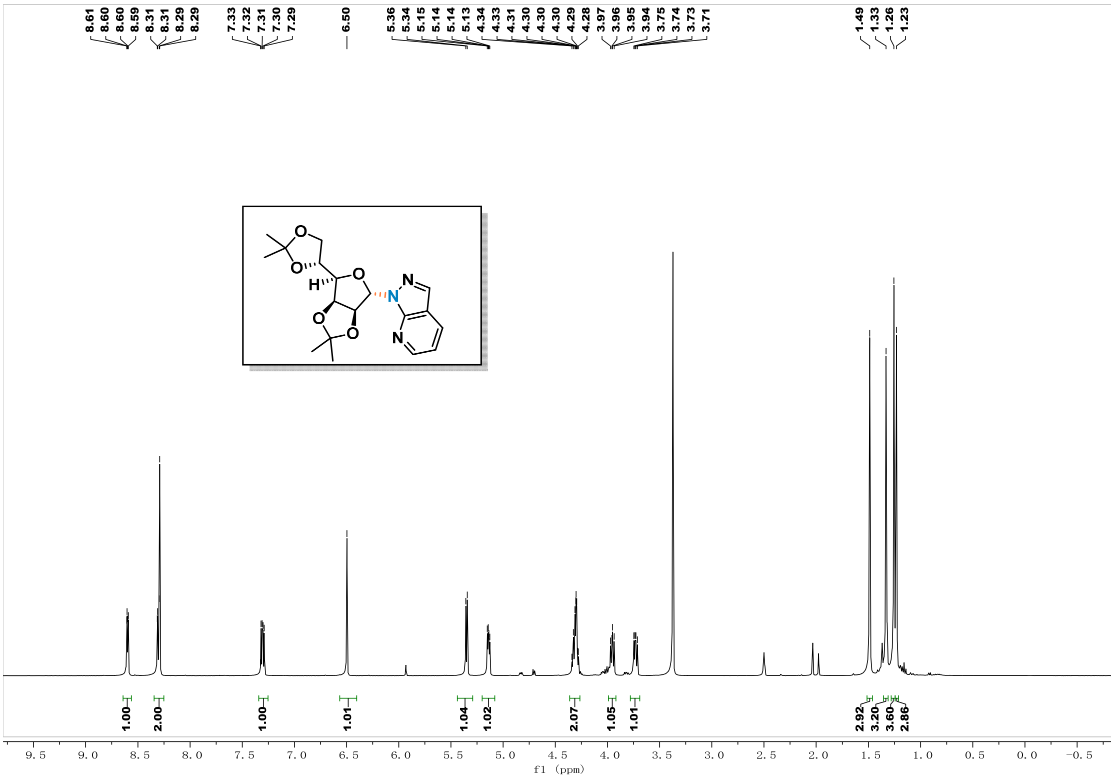

Supplementary Figure 56 | ^{1}H NMR (400 MHz, DMSO-d_6) **(2j)**

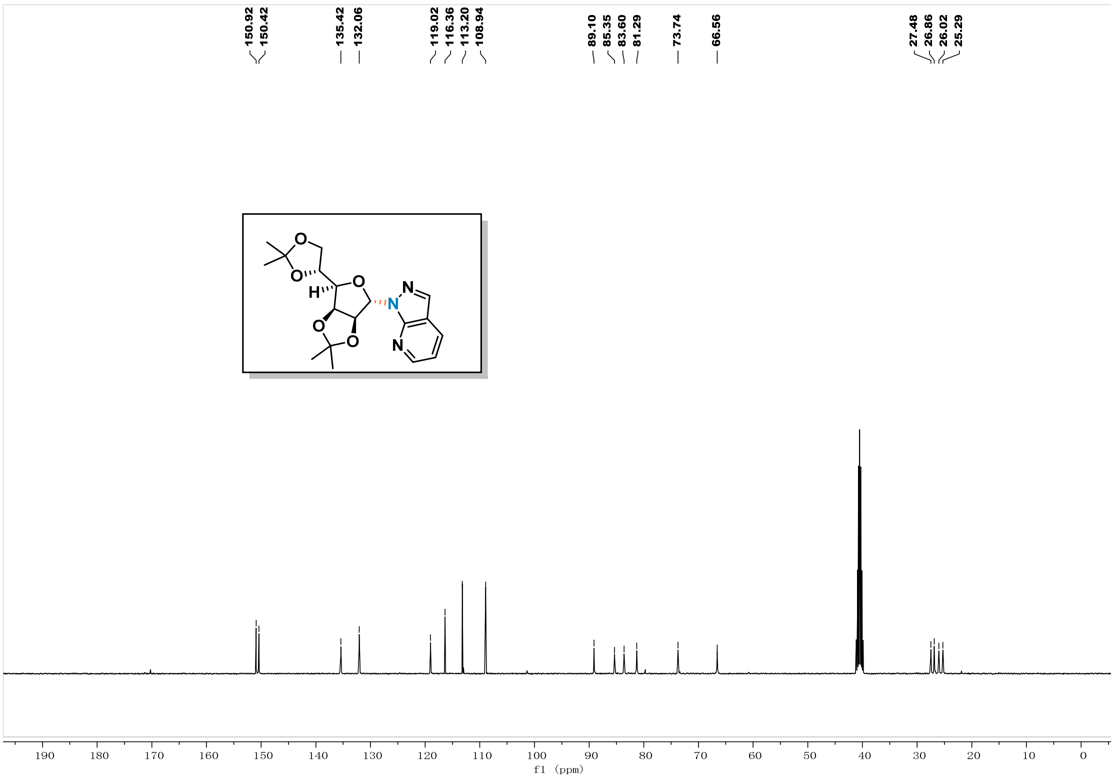

Supplementary Figure 57 | ^{13}C NMR (101 MHz, DMSO-d_6)) **(2j)**

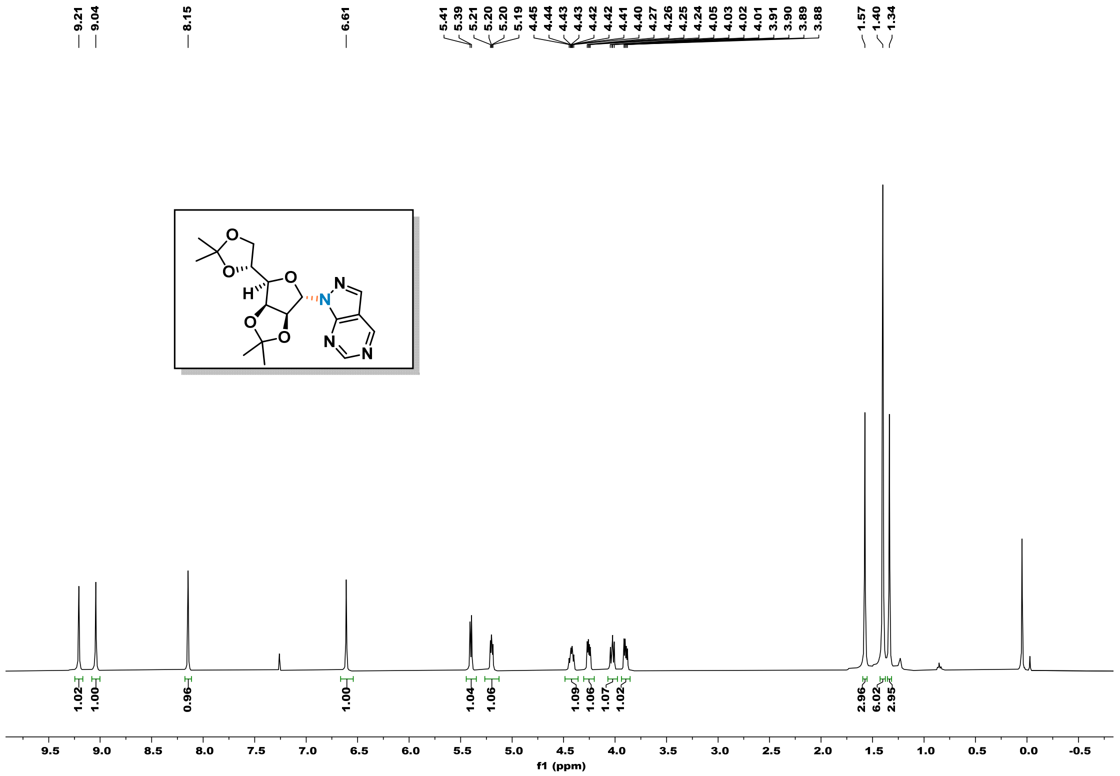

Supplementary Figure 58 | ^{1}H NMR (400 MHz, $CDCl_3$) **(2k)**

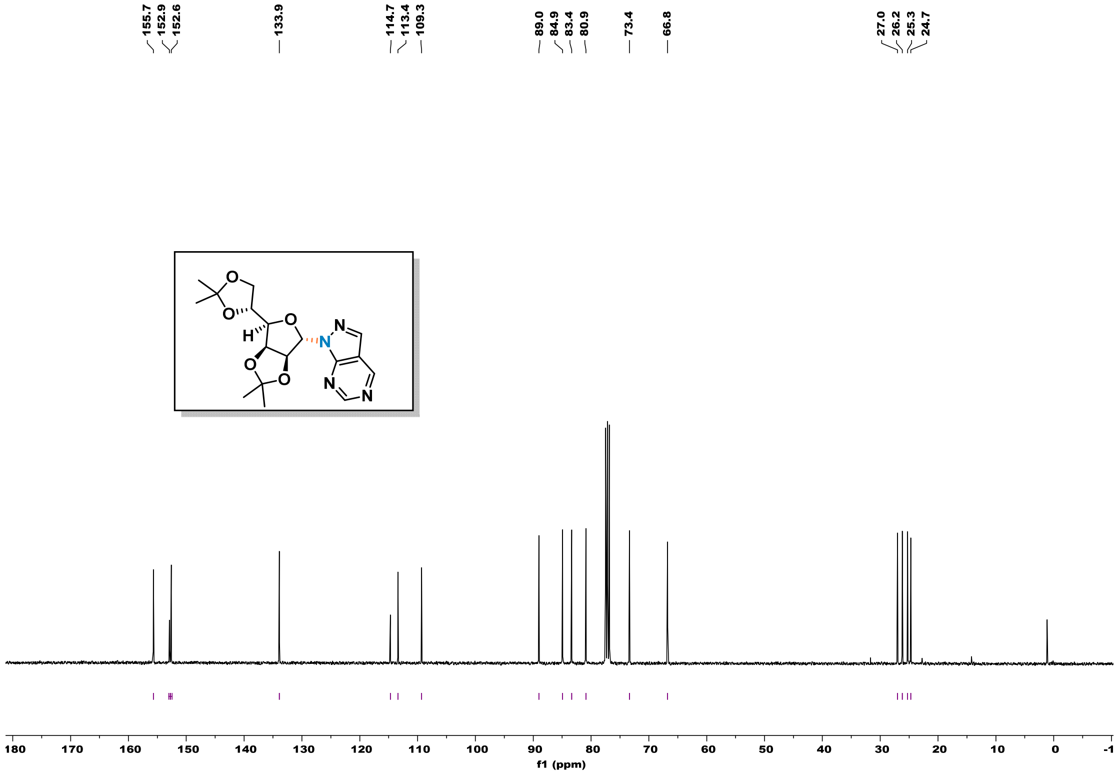

Supplementary Figure 59 | ^{13}C NMR (101 MHz, $CDCl_3$) **(2k)**

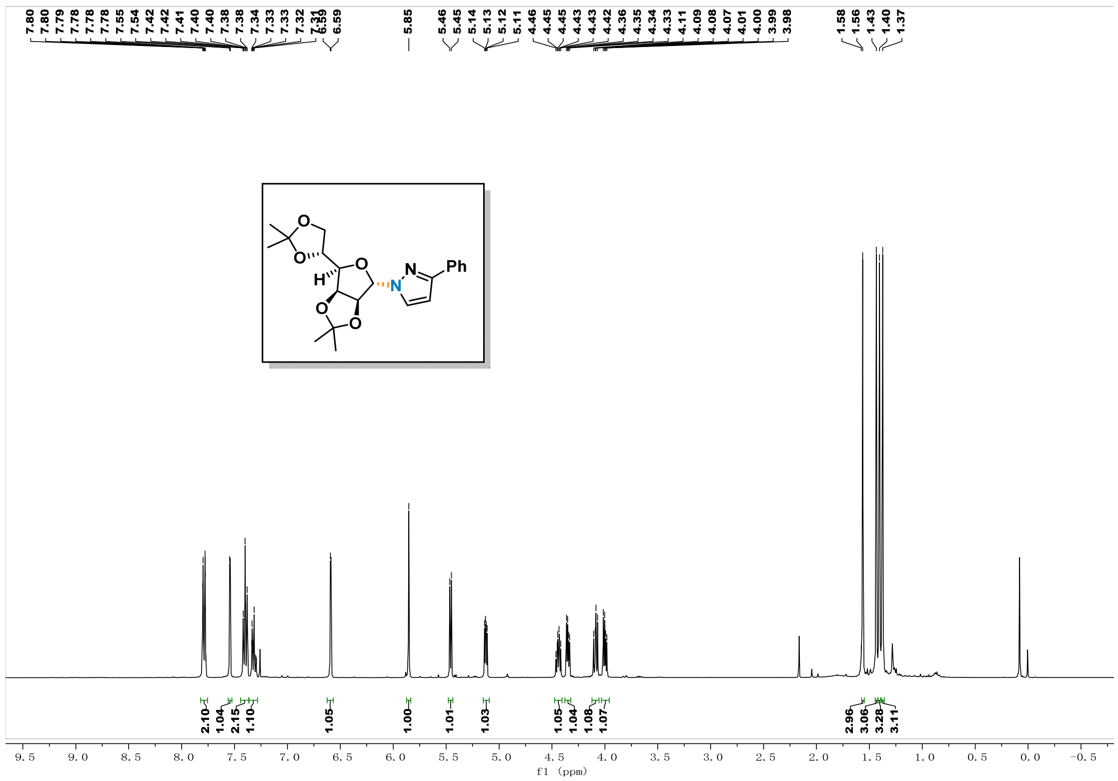

Supplementary Figure 60 | ^{1}H NMR (400 MHz, $CDCl_3$) **(3a)**

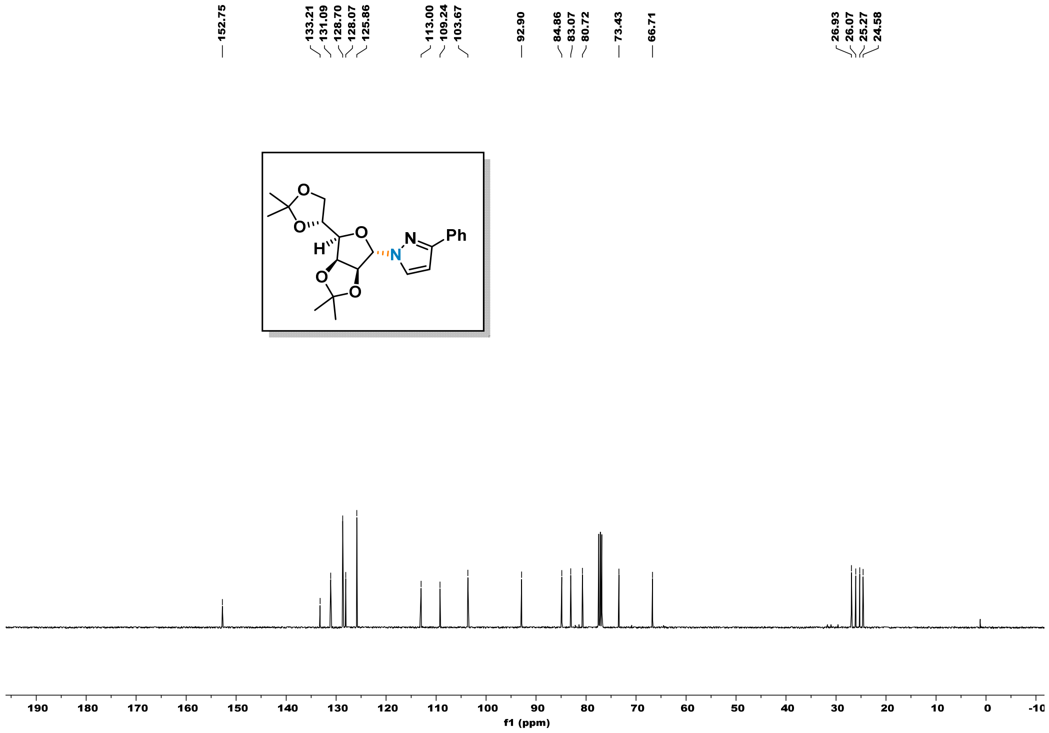

Supplementary Figure 61 | ^{13}C NMR (101 MHz, $CDCl_3$) **(3a)**

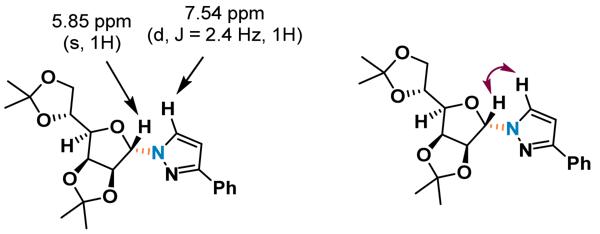

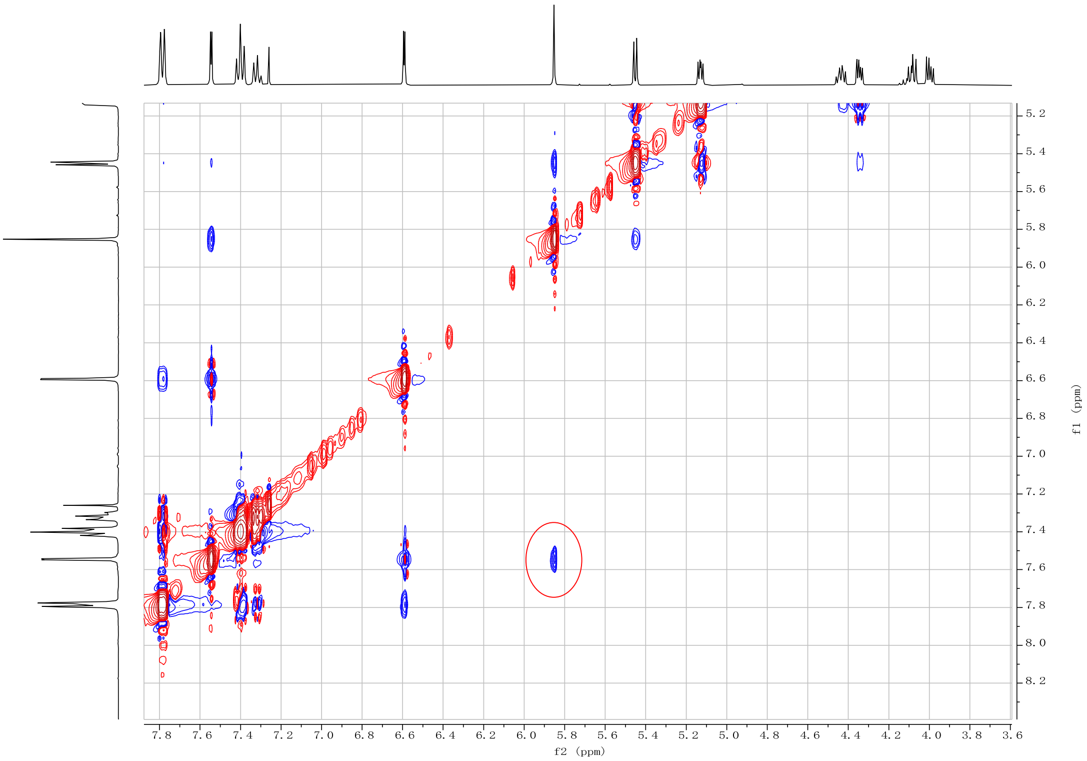

Supplementary Figure 62 | NOESY of **3a**

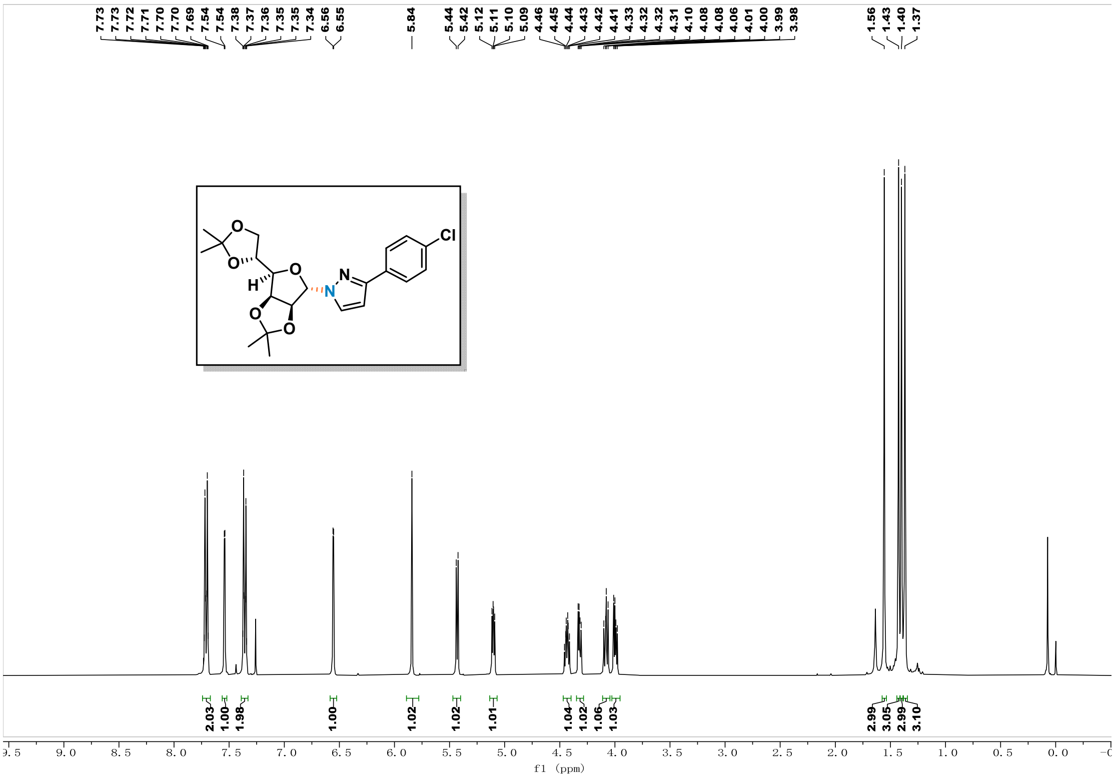

Supplementary Figure 63 | ^{1}H NMR (400 MHz, $CDCl_3$) **(3b)**

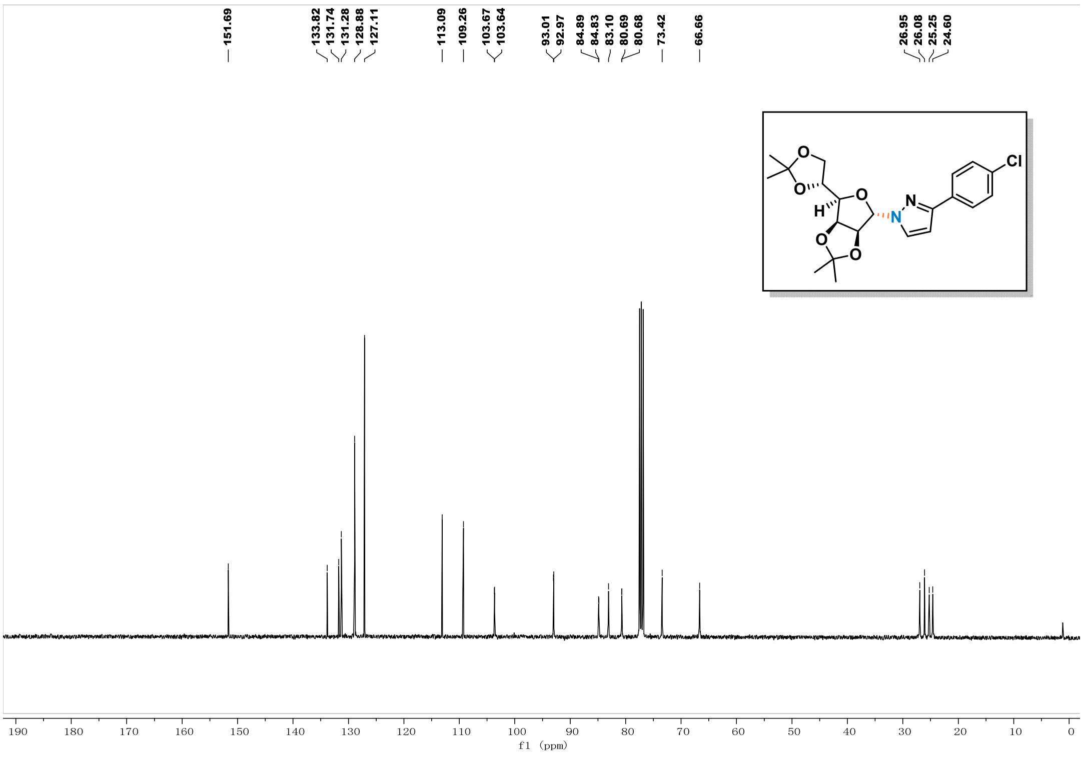

Supplementary Figure 64 | ^{13}C NMR (101 MHz, $CDCl_3$) **(3b)**

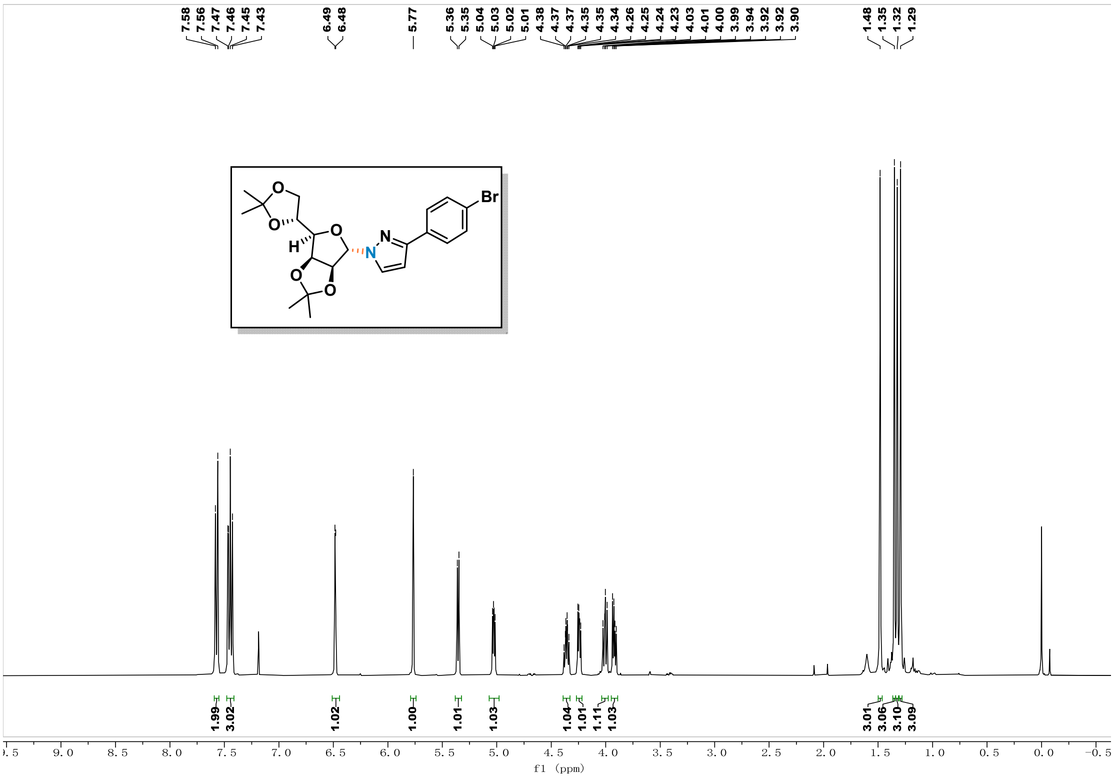

Supplementary Figure 65 | ^{1}H NMR (400 MHz, CDCl$_3$) **(3c)**

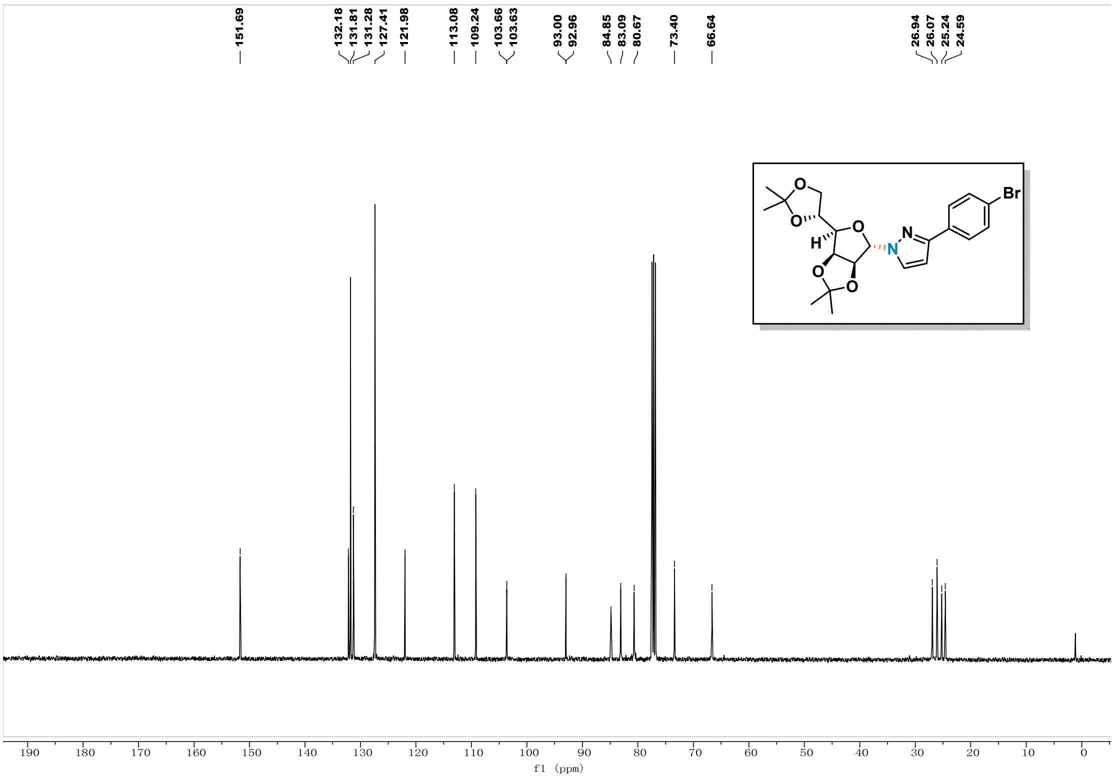

Supplementary Figure 66 | ^{13}C NMR (101 MHz, CDCl$_3$) **(3c)**

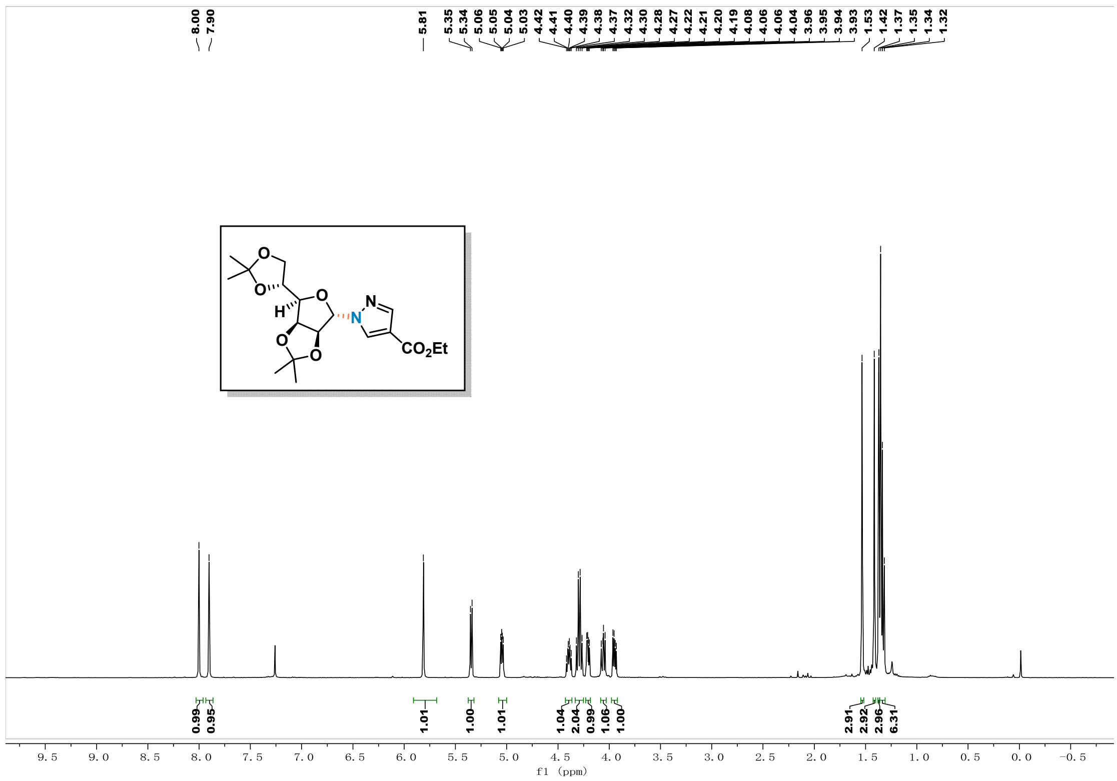

Supplementary Figure 67 | ^{1}H NMR (400 MHz, CDCl3) **(3d)**

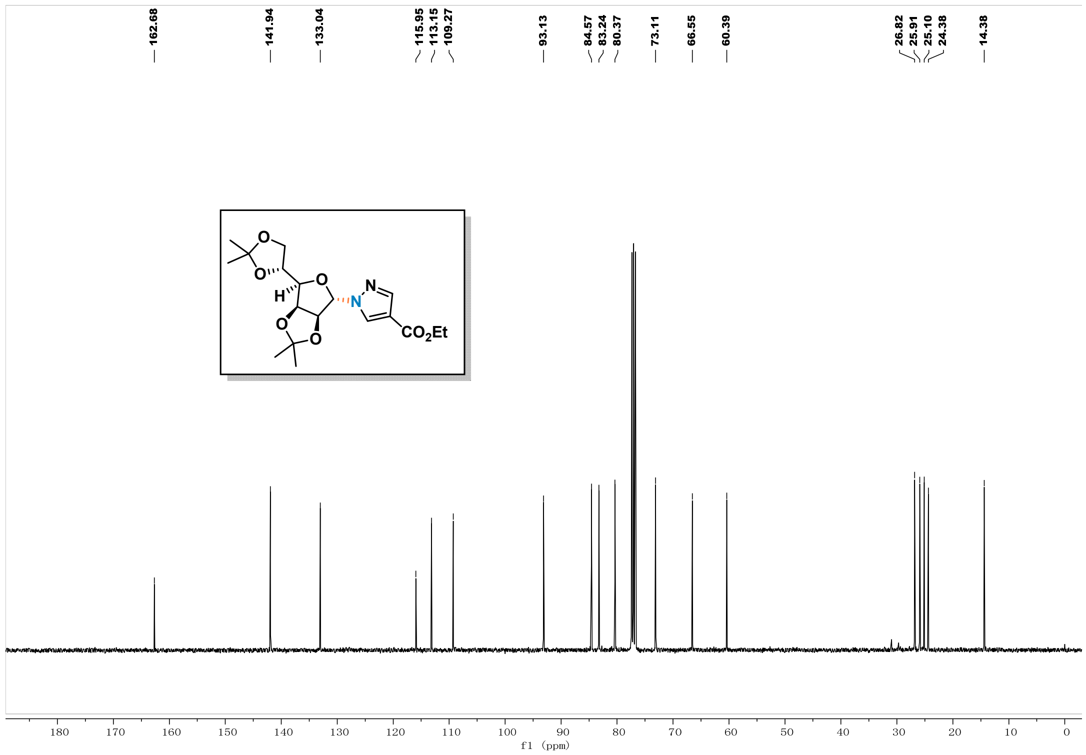

Supplementary Figure 68 | ^{13}C NMR (101 MHz, CDCl3) **(3d)**

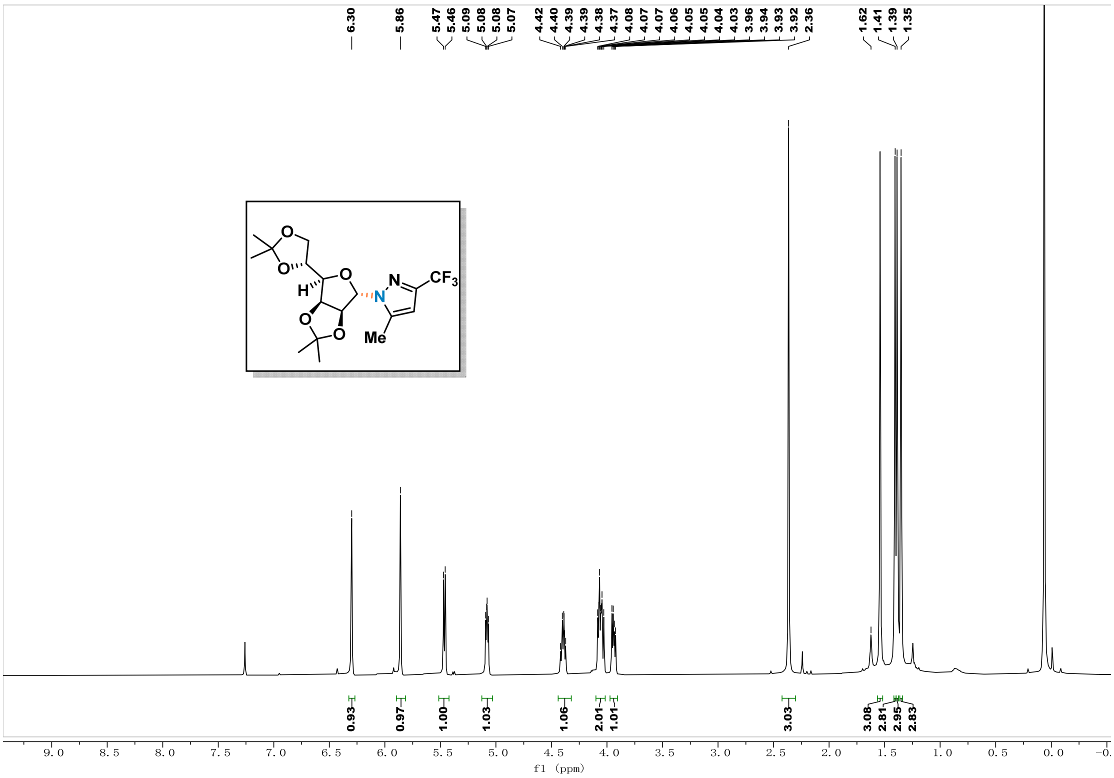

Supplementary Figure 69 | ^{1}H NMR (400 MHz, $CDCl_3$) **(3e)**

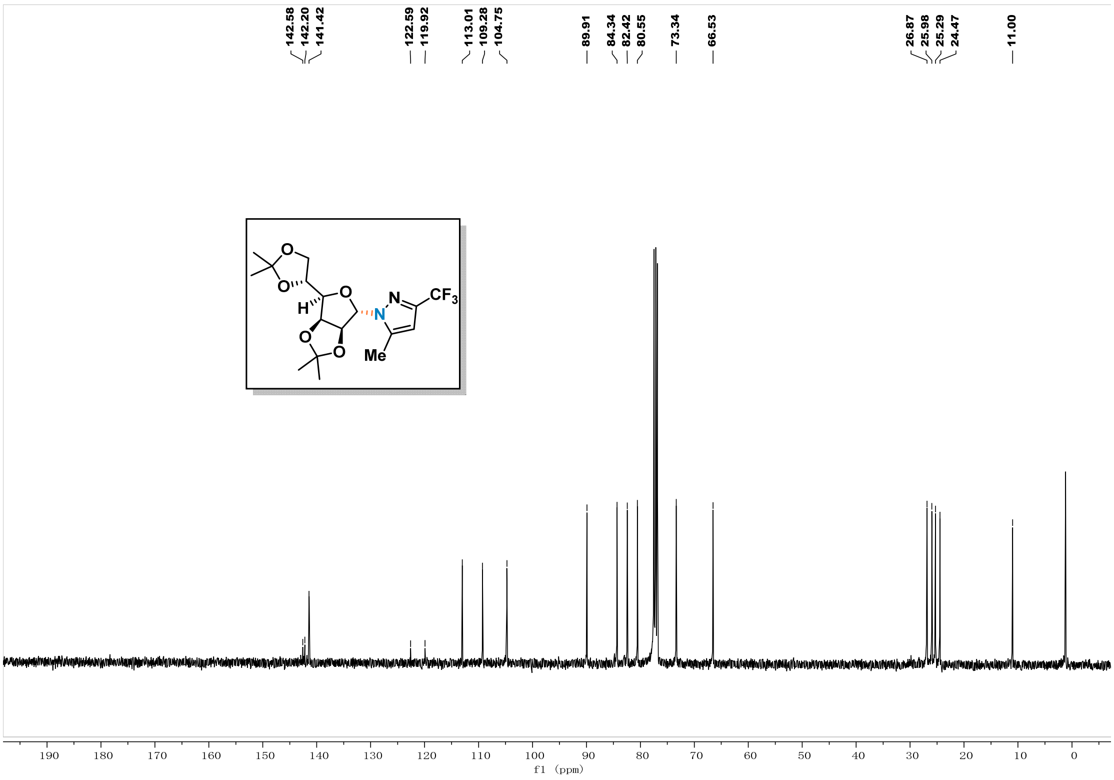

Supplementary Figure 70 | ^{13}C NMR (101 MHz, $CDCl_3$) **(3e)**

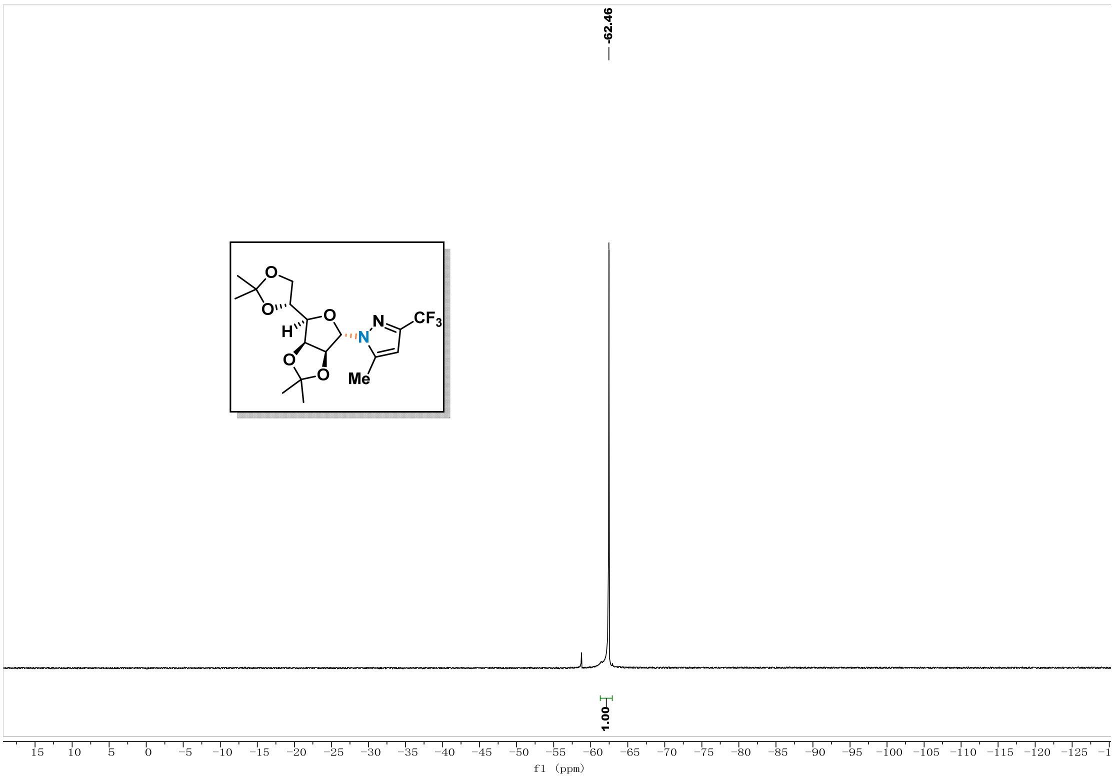

Supplementary Figure 71 | ^{19}F NMR (376 MHz, $CDCl_3$) **(3e)**

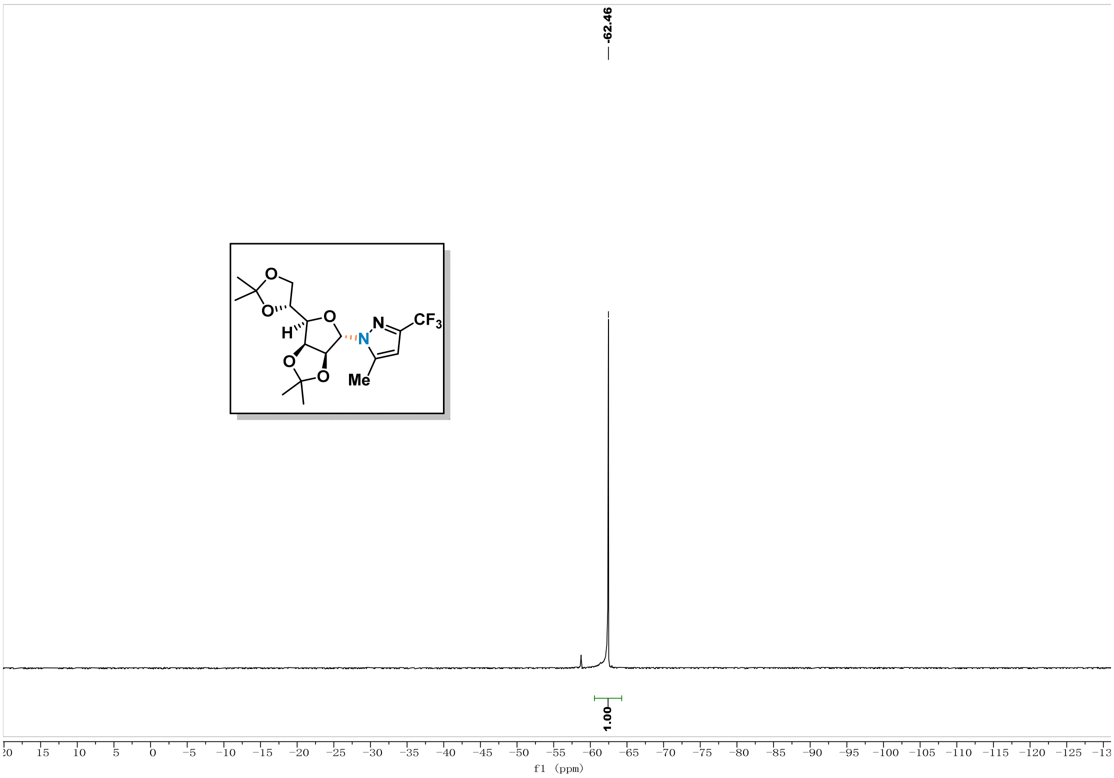

Supplementary Figure 72 | ^{19}F {^{1}H} NMR (376 MHz, $CDCl_3$) **(3e)**

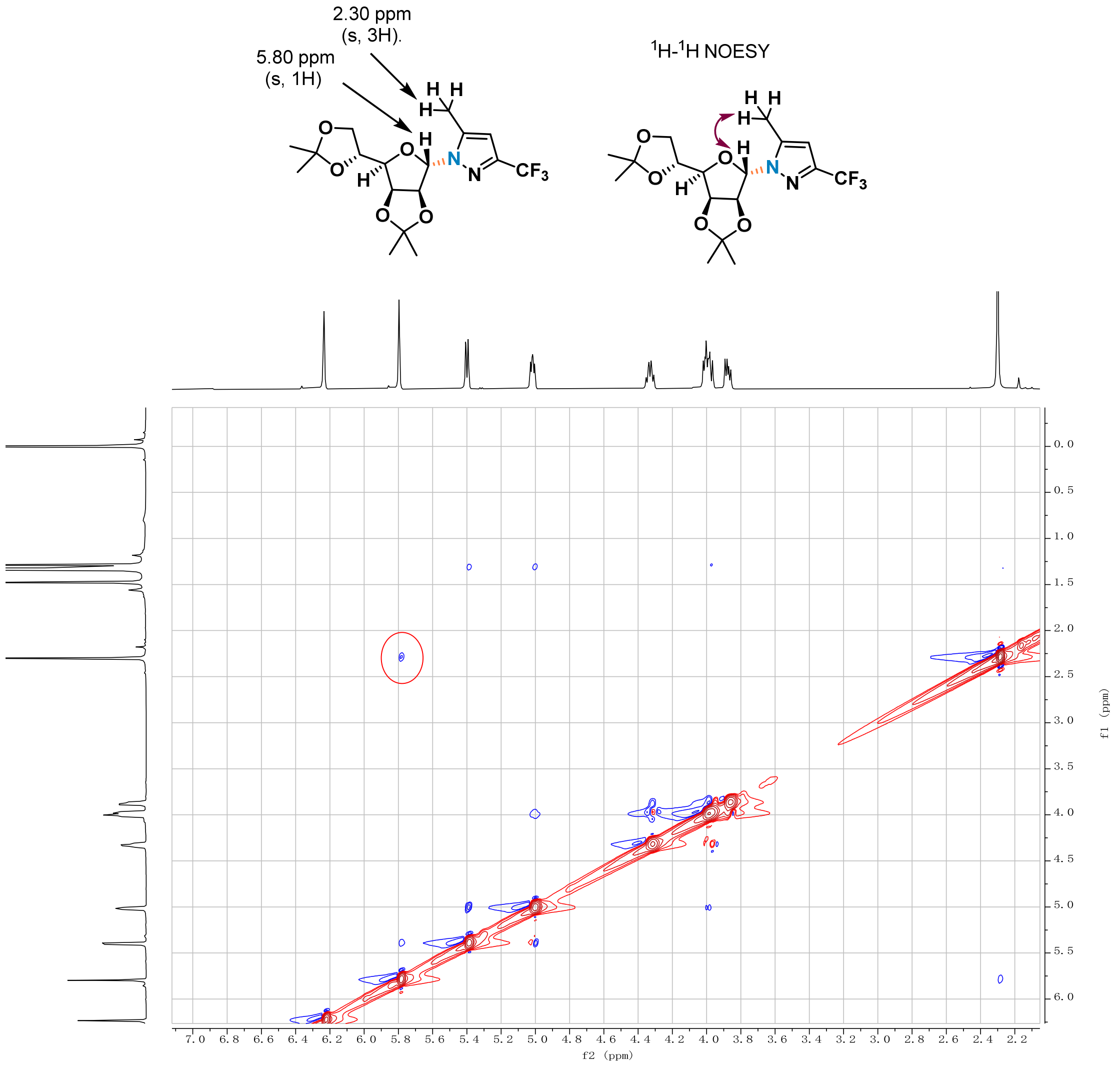

Supplementary Figure 73 | NOESY of **3e**

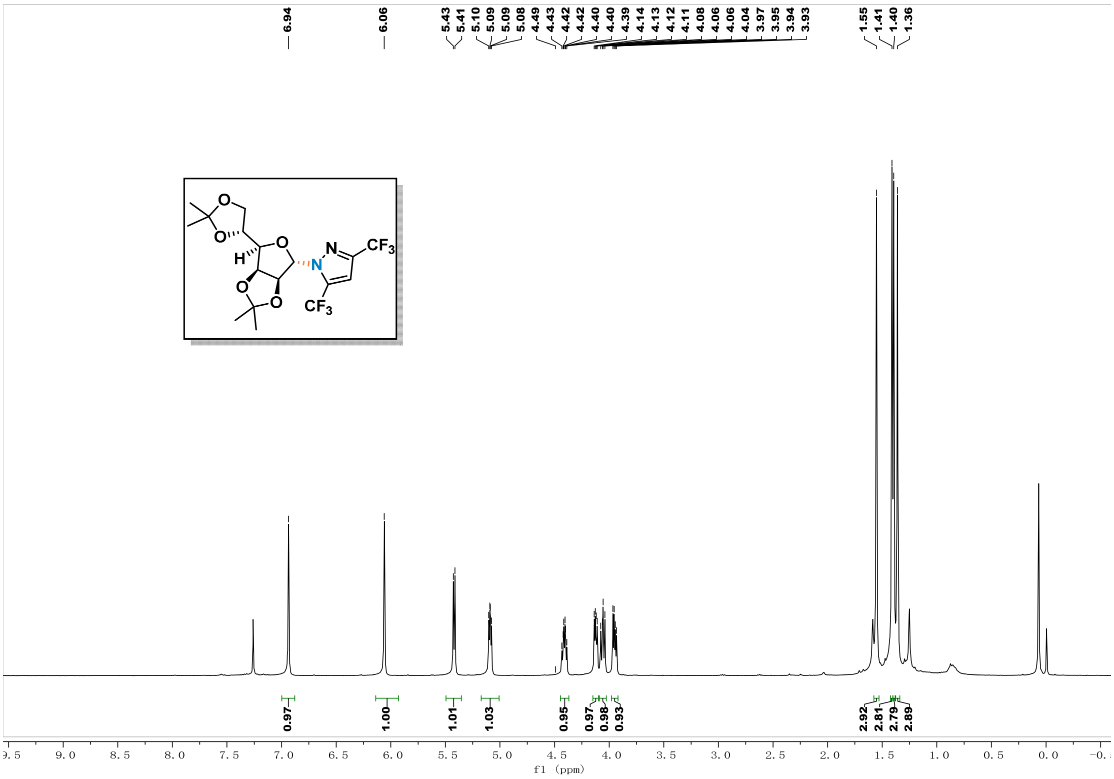

Supplementary Figure 74 | ^{1}H NMR (400 MHz, $CDCl_3$) **(3f)**

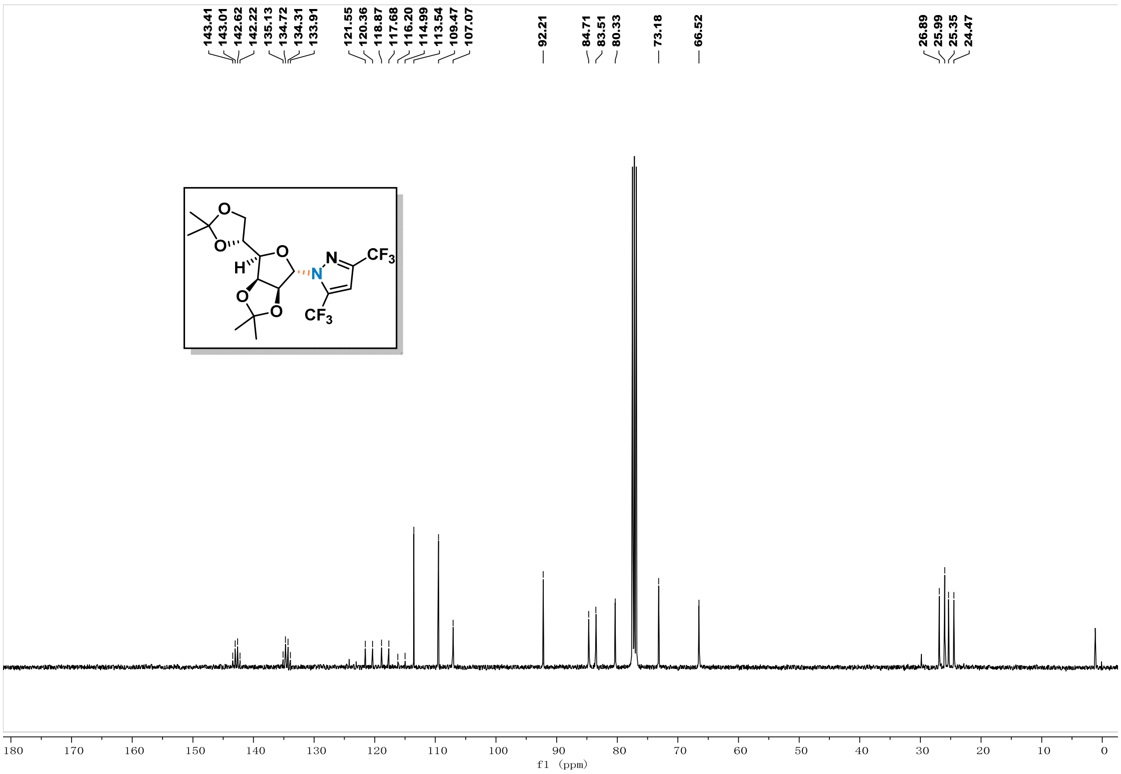

Supplementary Figure 75 | ^{13}C NMR (101 MHz, $CDCl_3$) **(3f)**

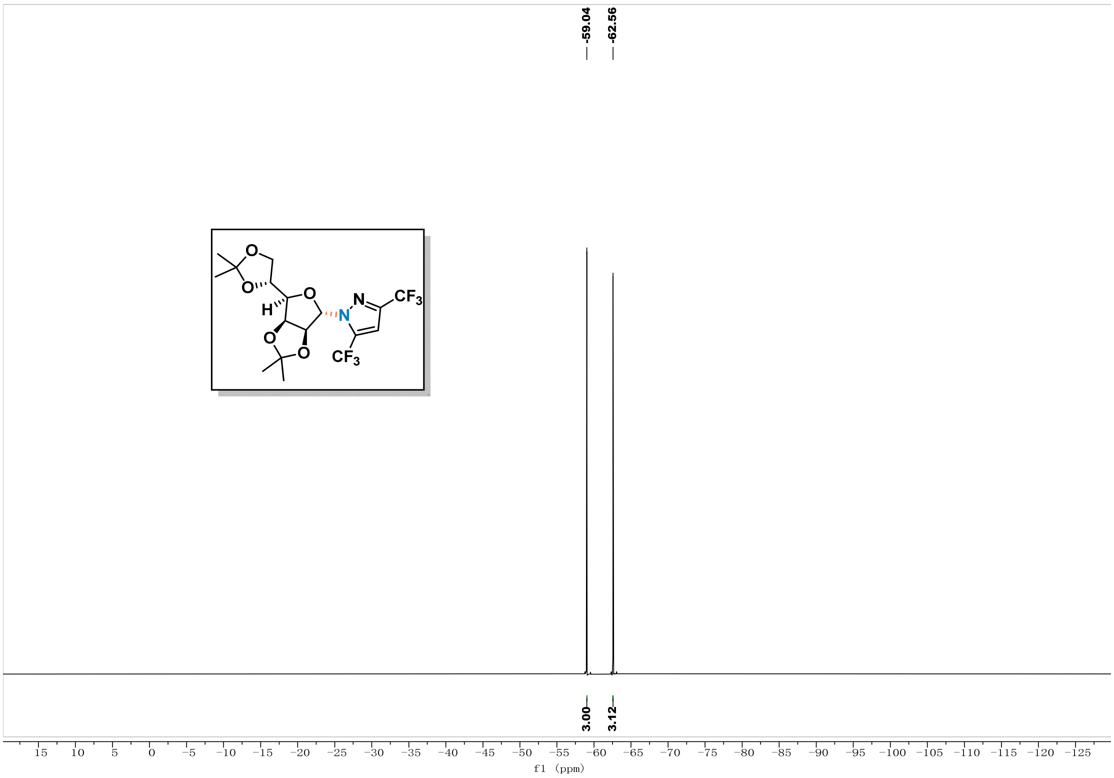

Supplementary Figure 76 | ^{19}F NMR (376 MHz, $CDCl_3$) **(3f)**

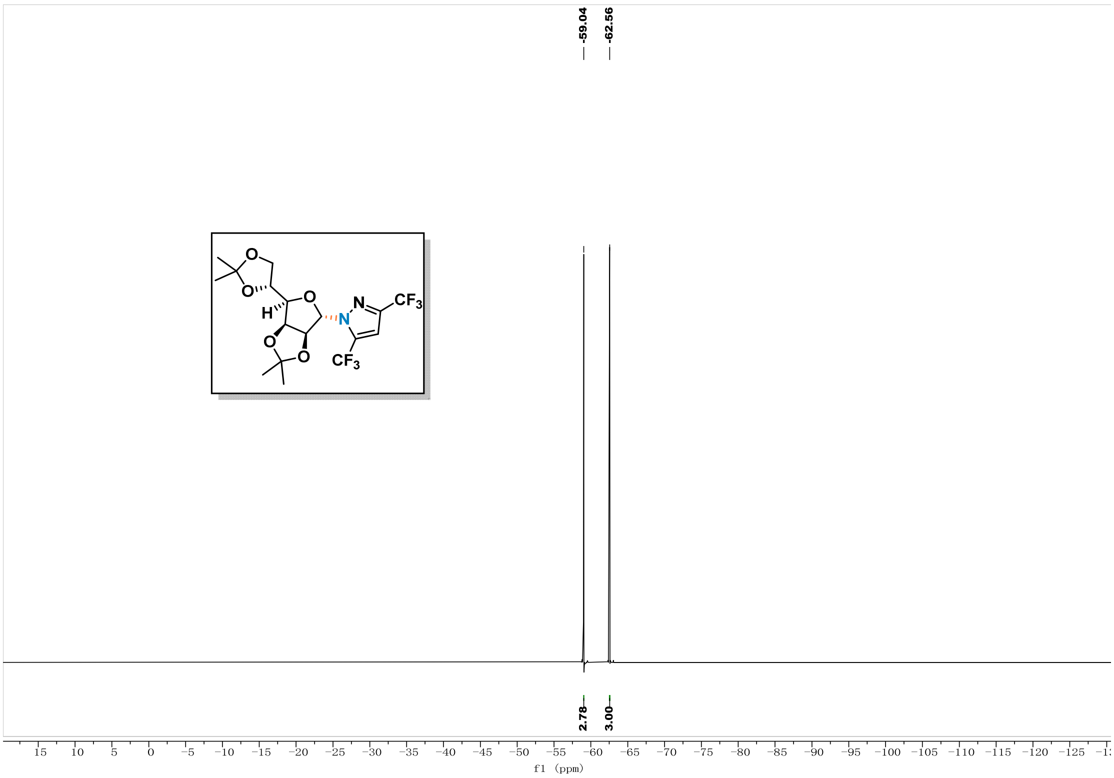

Supplementary Figure 77 | ^{19}F {^{1}H} NMR (376 MHz, $CDCl_3$) **(3f)**

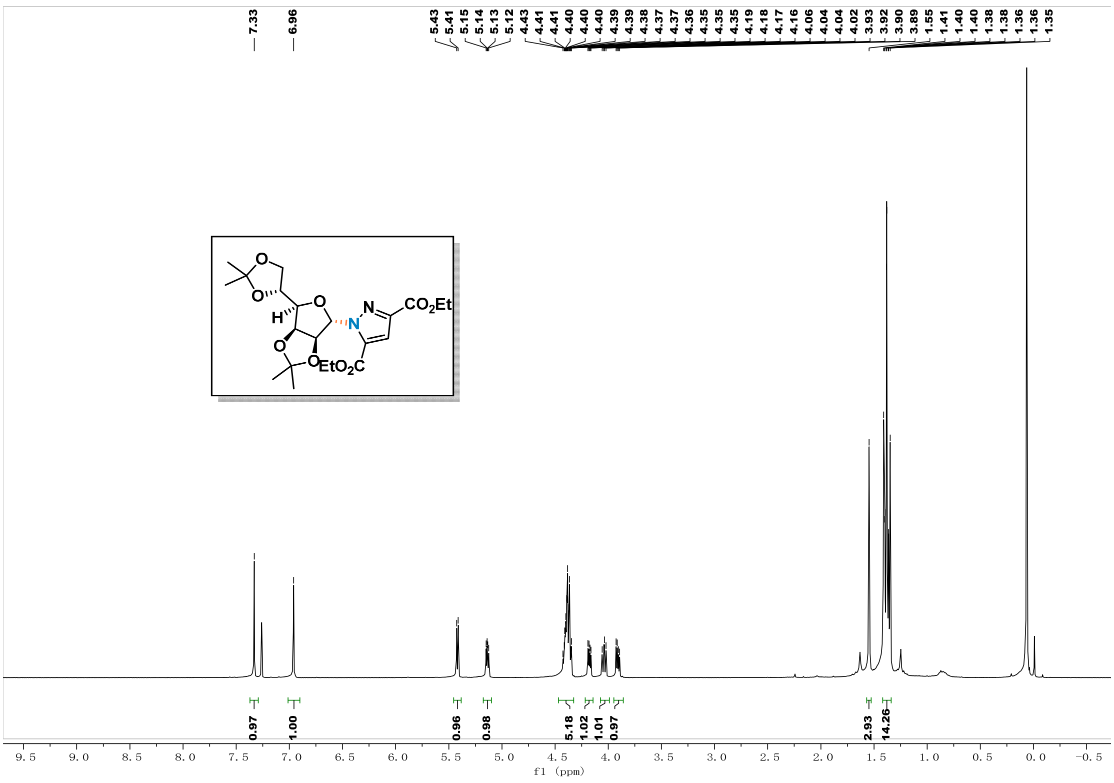

Supplementary Figure 78 | ^{1}H NMR (400 MHz, $CDCl_3$) **(3g)**

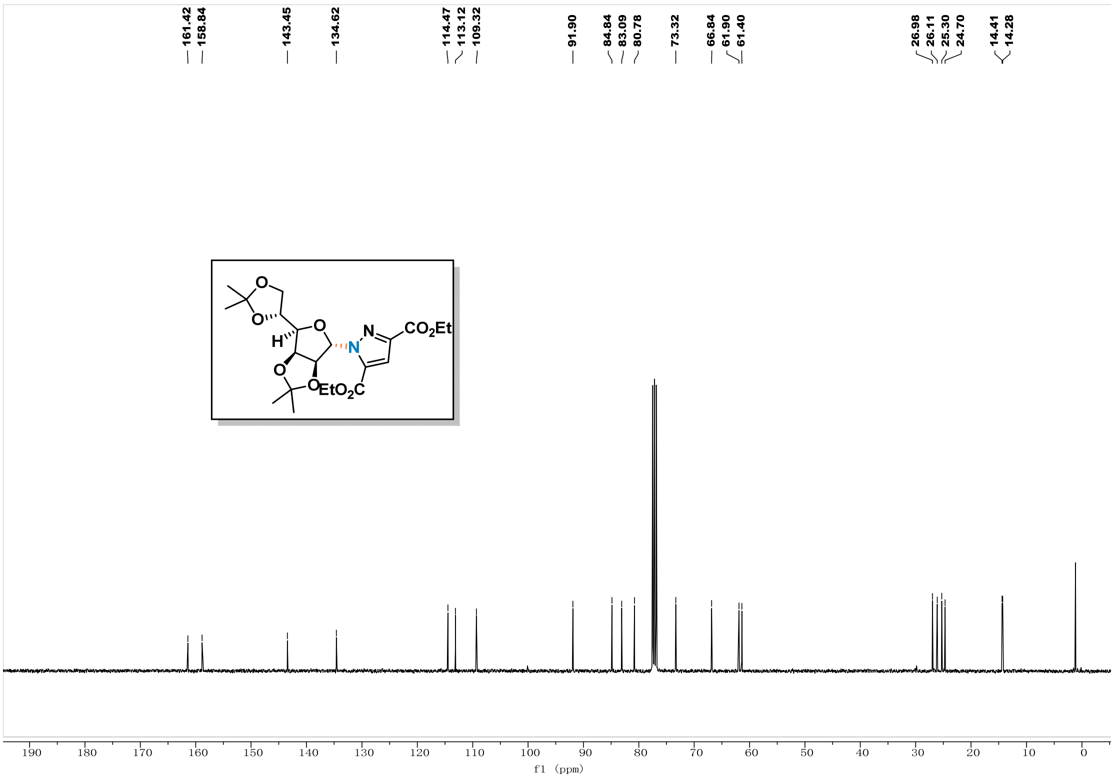

Supplementary Figure 79 | ^{13}C NMR (101 MHz, $CDCl_3$) **(3g)**

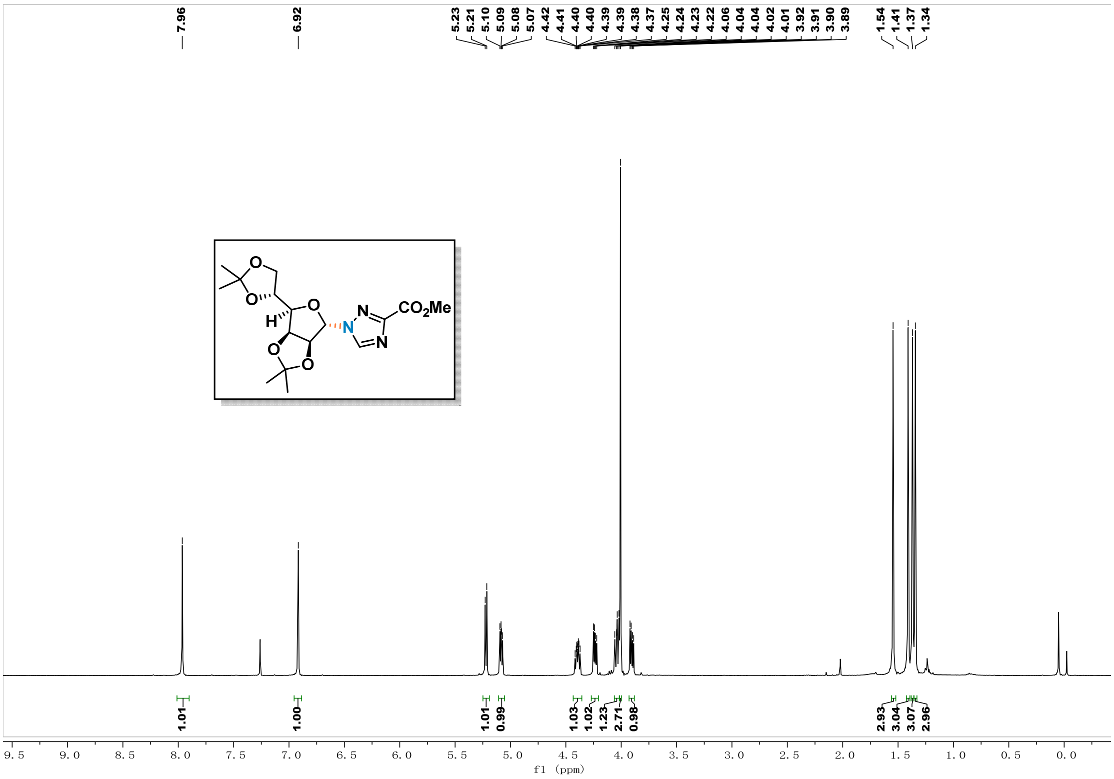

Supplementary Figure 80 | ^{1}H NMR (400 MHz, $CDCl_3$) **(3h)**

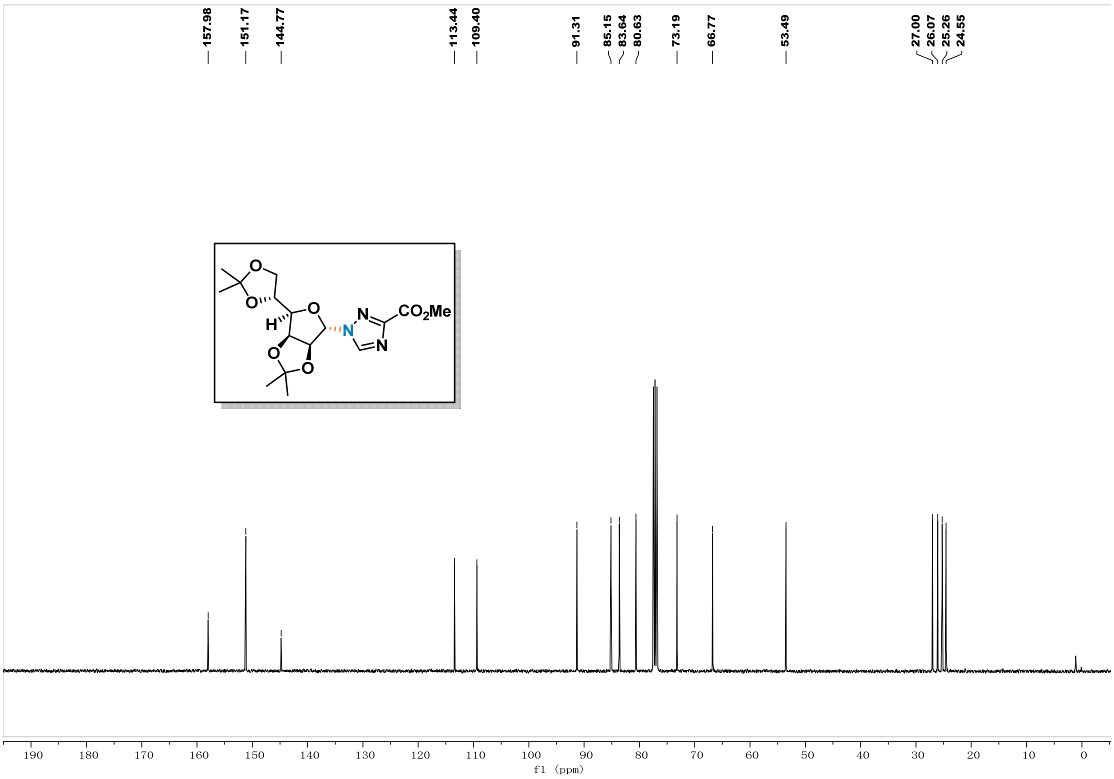

Supplementary Figure 81 | ^{13}C NMR (101 MHz, $CDCl_3$) **(3h)**

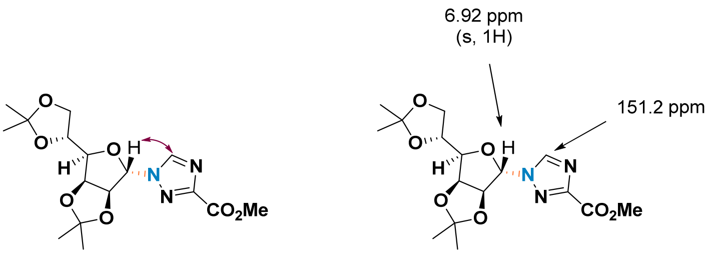

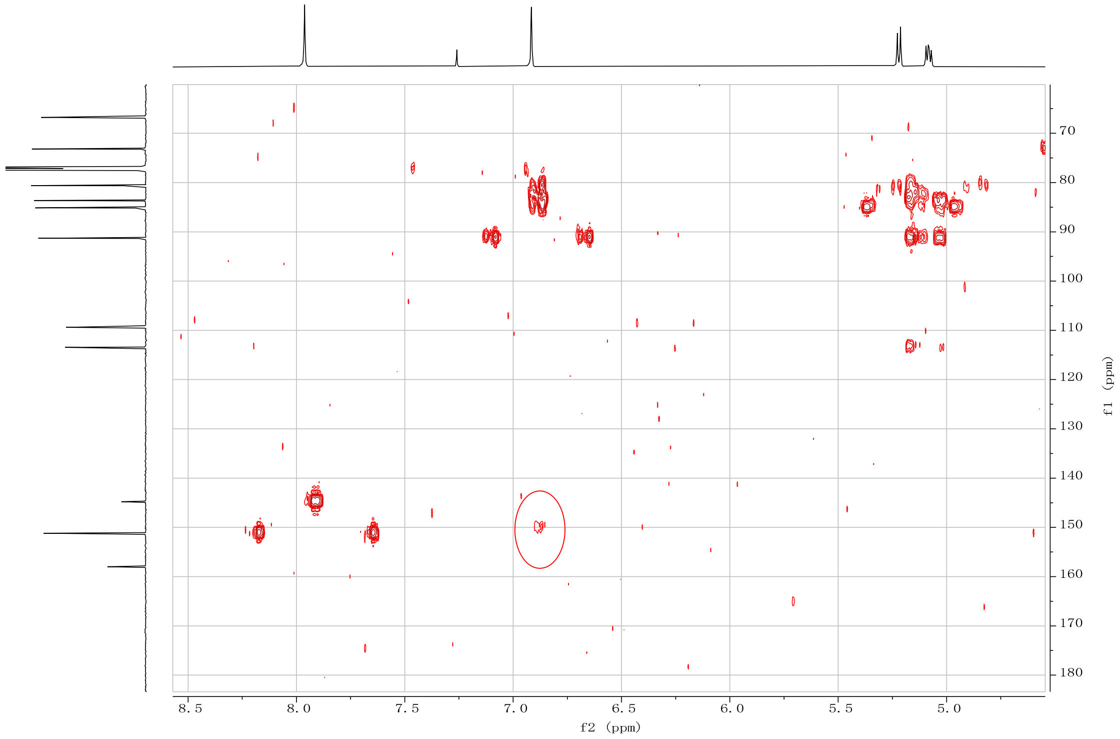

Supplementary Figure 82 | HMBC of **3h**

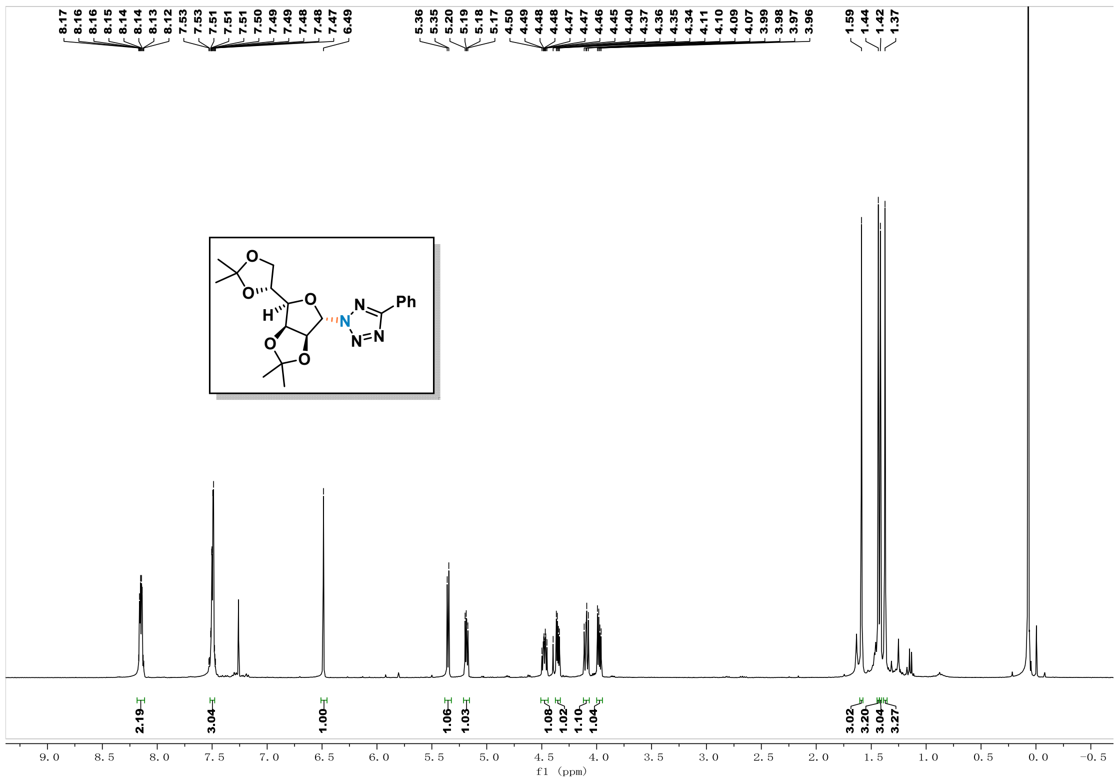

Supplementary Figure 83 | ^{1}H NMR (400 MHz, $CDCl_3$) **(3i)**

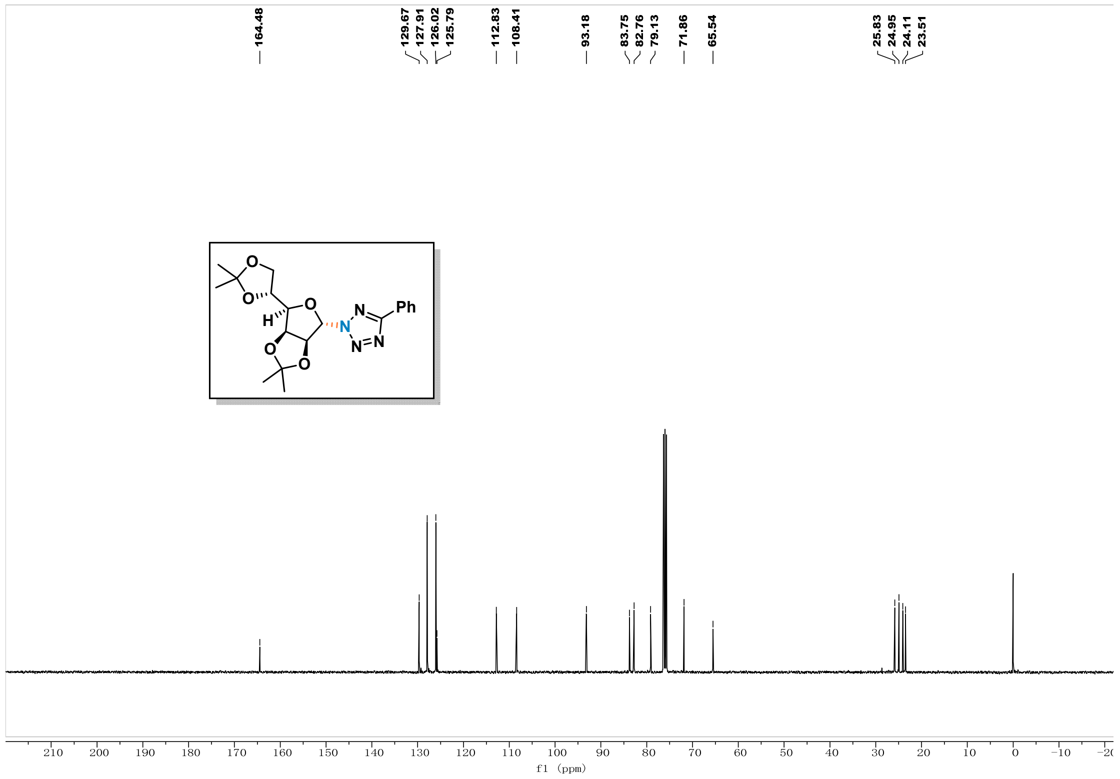

Supplementary Figure 84 | ^{13}C NMR (101 MHz, $CDCl_3$) **(3i)**

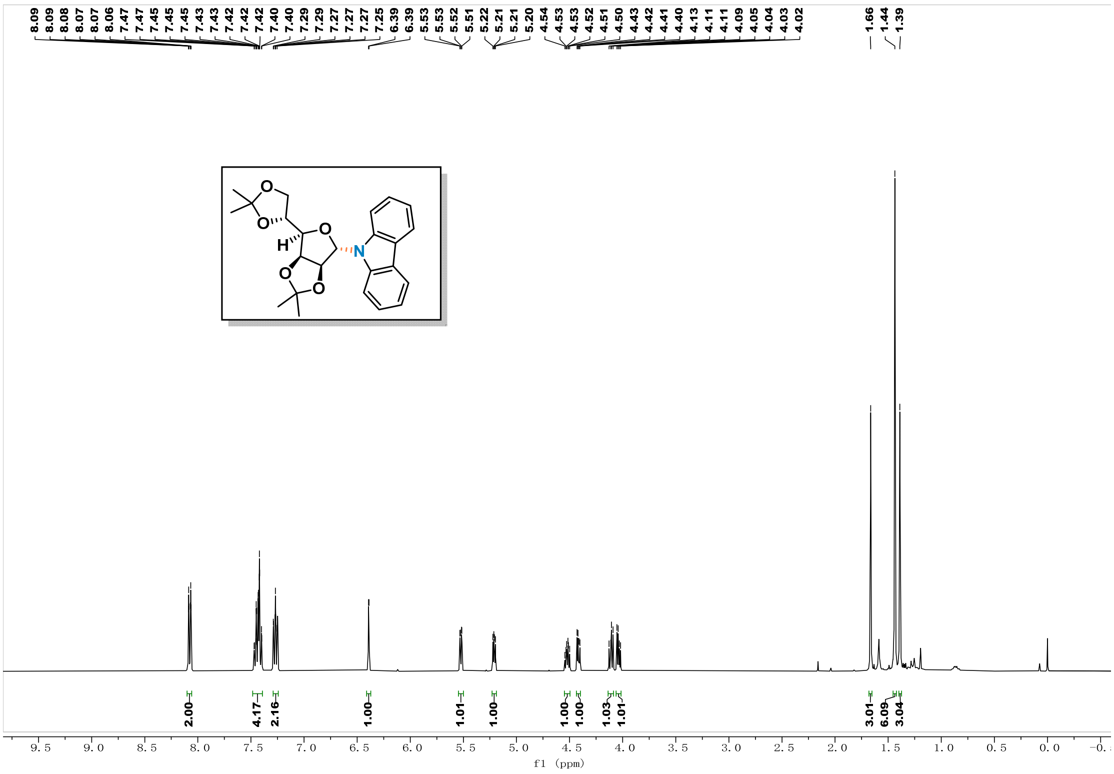

Supplementary Figure 85 | ^{1}H NMR (400 MHz, $CDCl_3$) **(4a)**

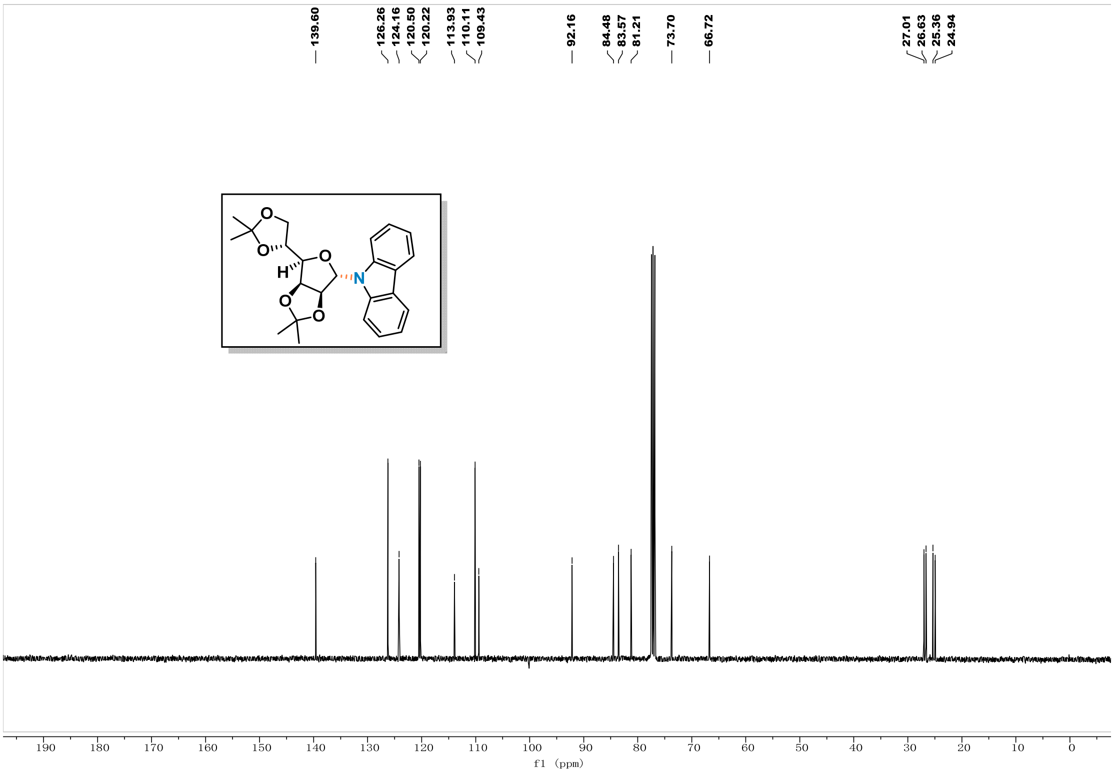

Supplementary Figure 86 | ^{13}C NMR (101 MHz, $CDCl_3$) **(4a)**

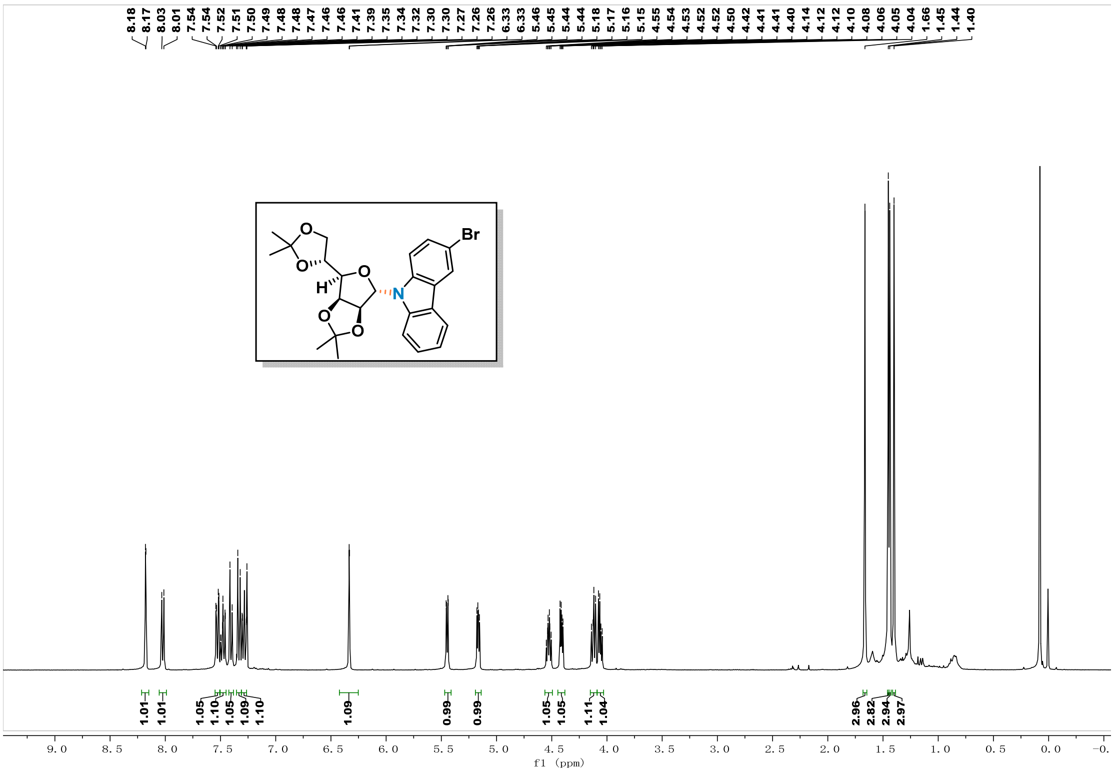

Supplementary Figure 87 | ^{1}H NMR (400 MHz, $CDCl_3$) **(4b)**

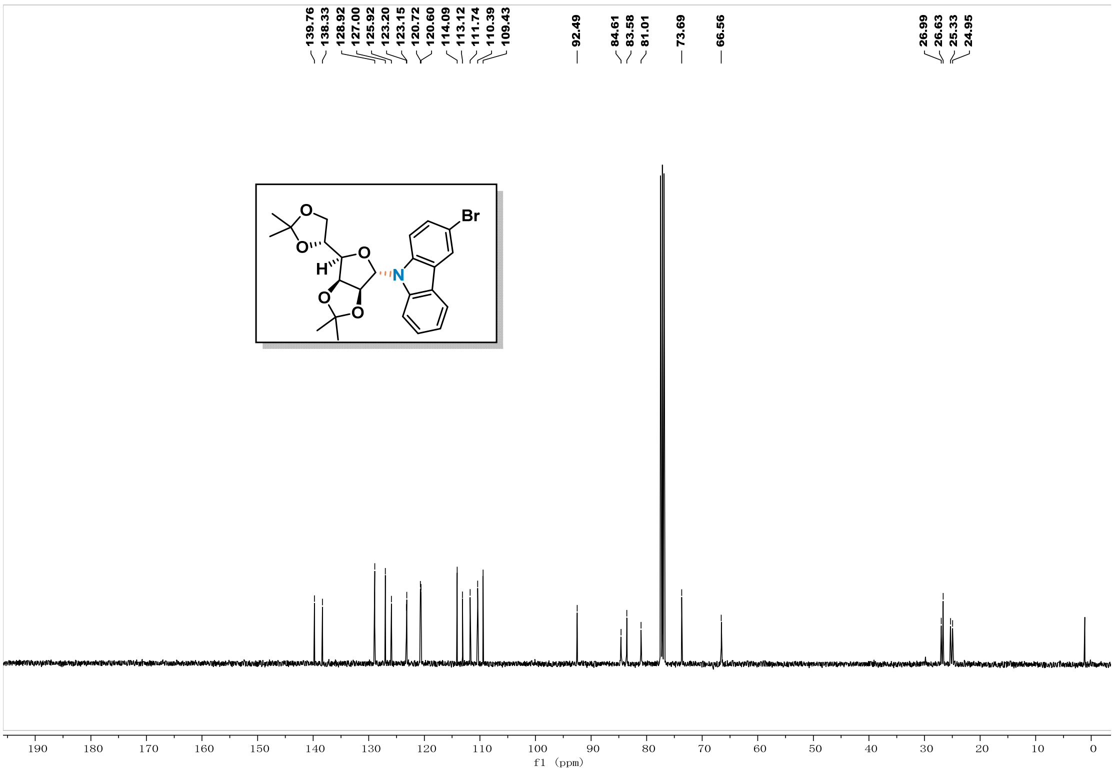

Supplementary Figure 88 | ^{13}C NMR (101 MHz, $CDCl_3$) **(4b)**

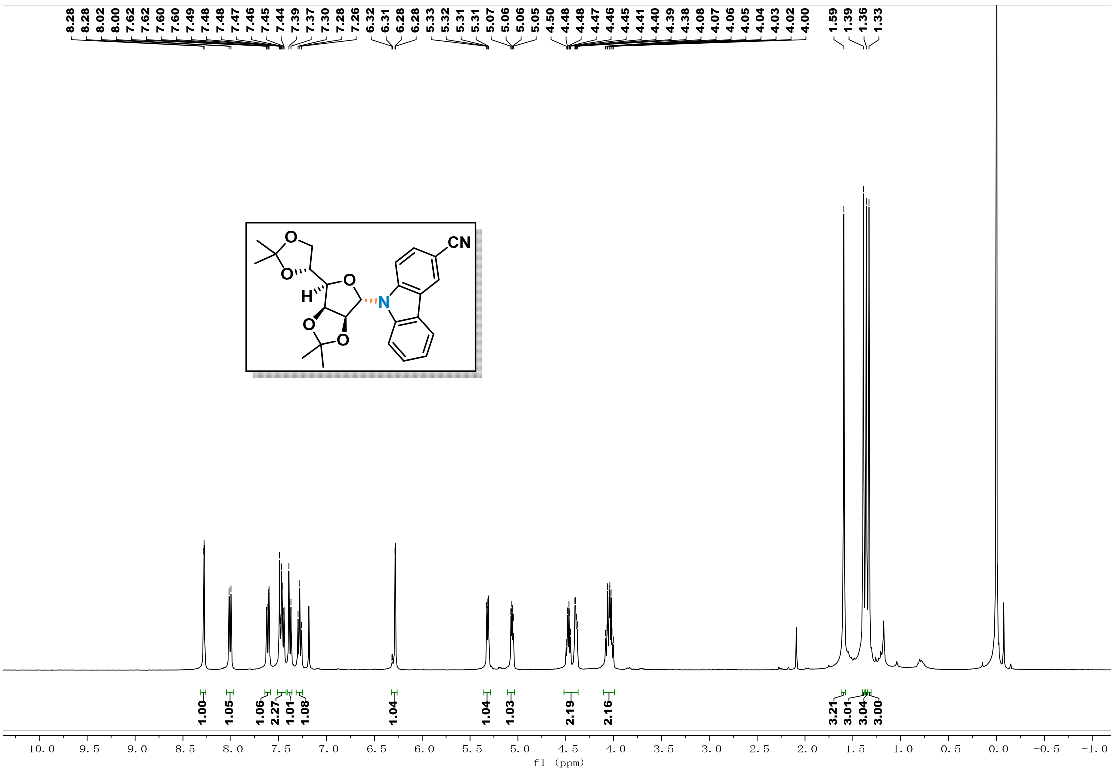

Supplementary Figure 89 | ^{1}H NMR (400 MHz, $CDCl_3$) **(4c)**

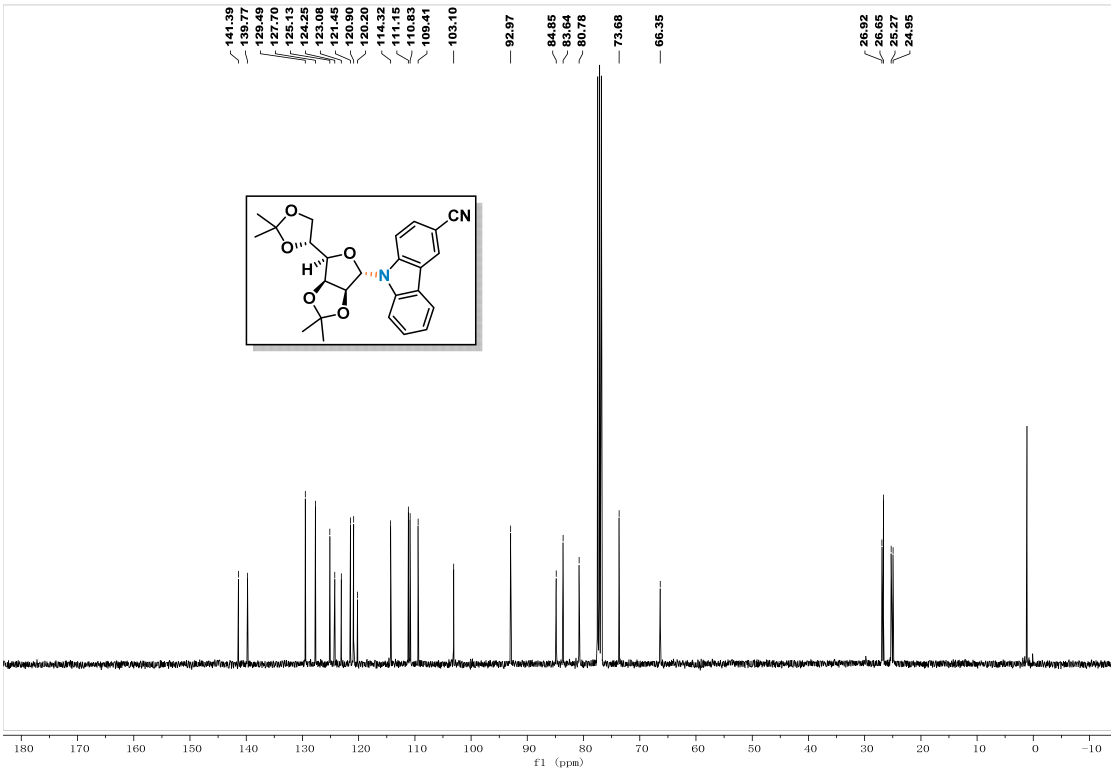

Supplementary Figure 90 | ^{13}C NMR (101 MHz, $CDCl_3$) **(4c)**

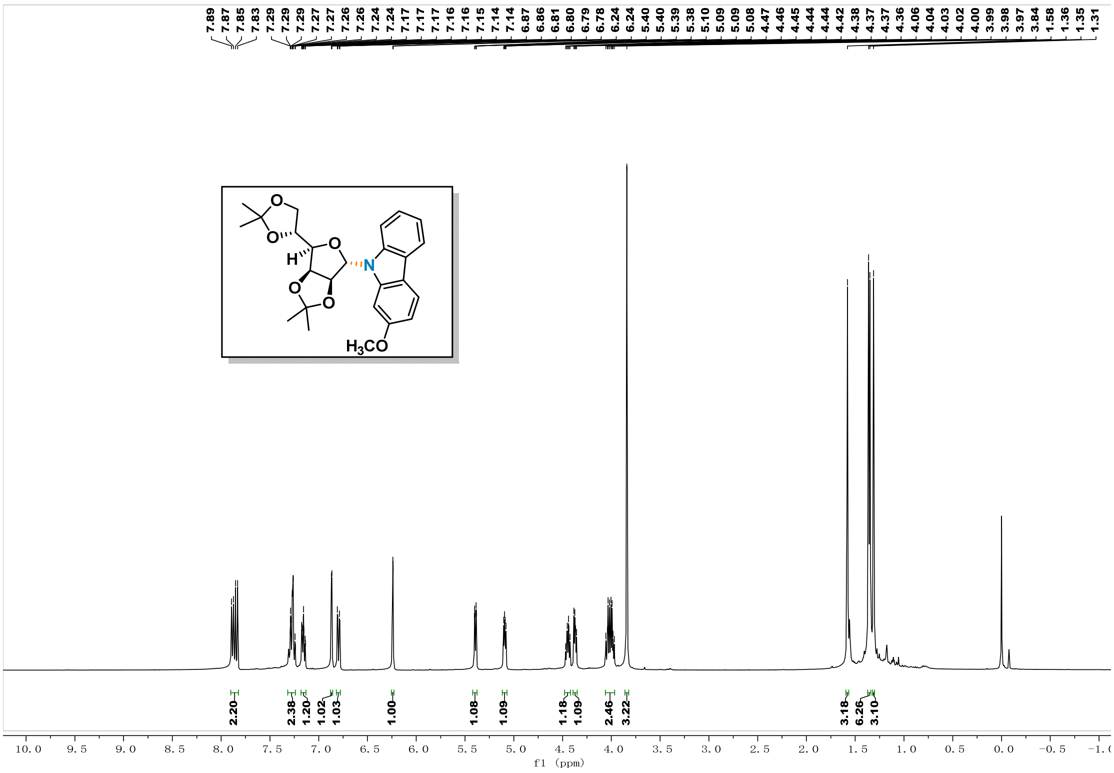

Supplementary Figure 91 | ^{1}H NMR (400 MHz, $CDCl_3$) **(4d)**

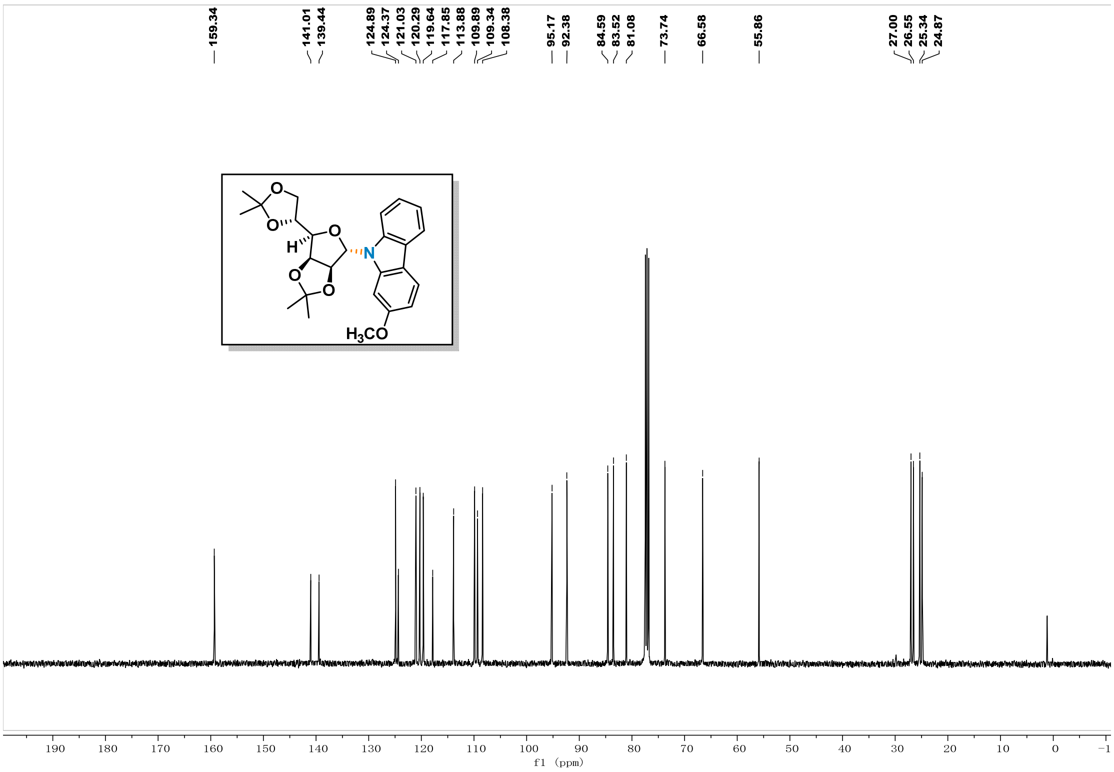

Supplementary Figure 92 | ^{13}C NMR (101 MHz, $CDCl_3$) **(4d)**

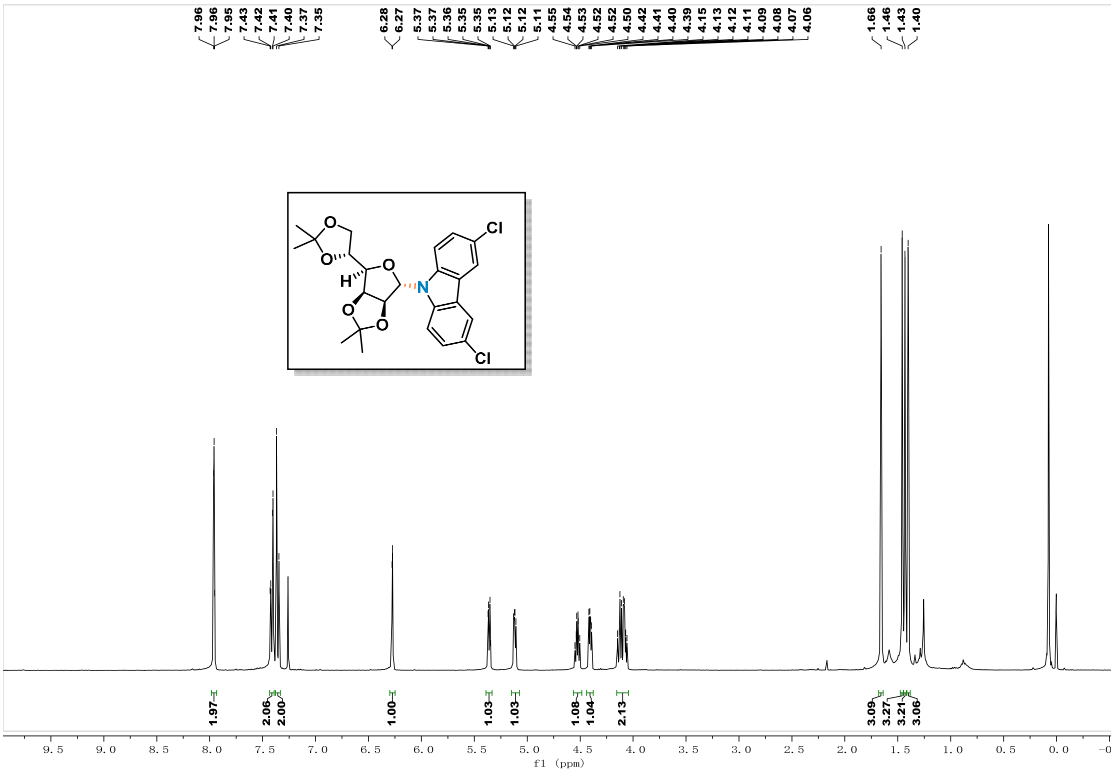

Supplementary Figure 93 | ^{1}H NMR (400 MHz, $CDCl_3$) **(4e)**

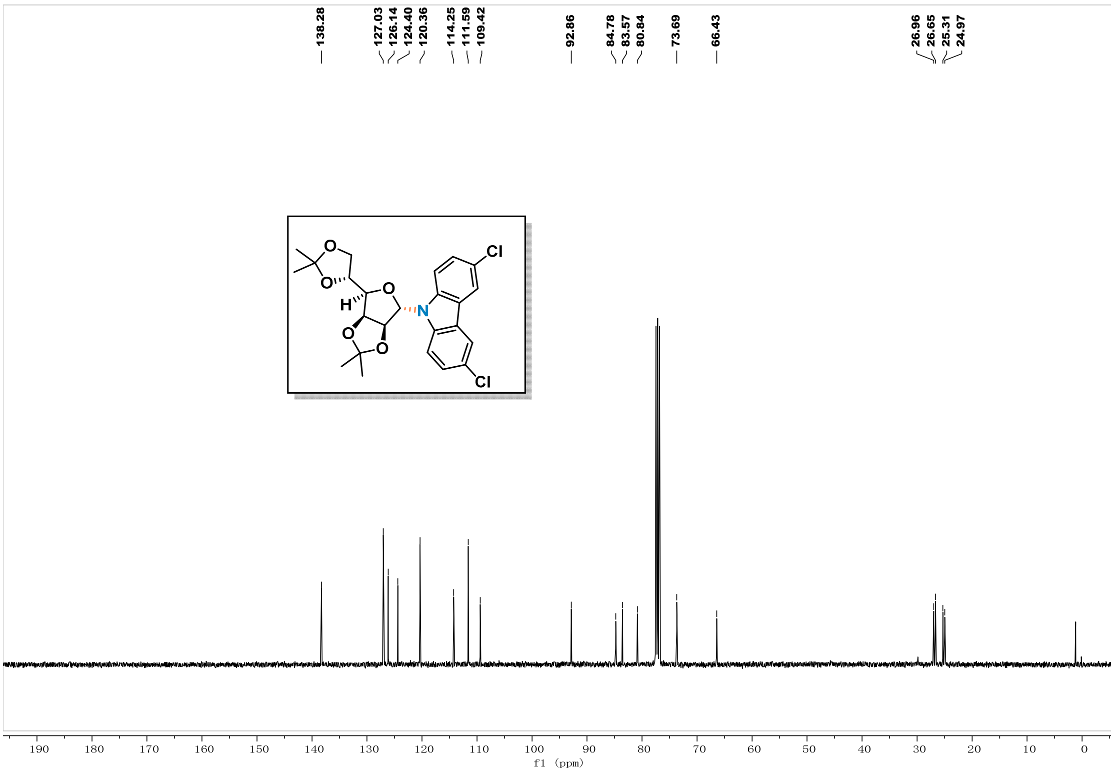

Supplementary Figure 94 | ^{13}C NMR (101 MHz, $CDCl_3$) **(4e)**

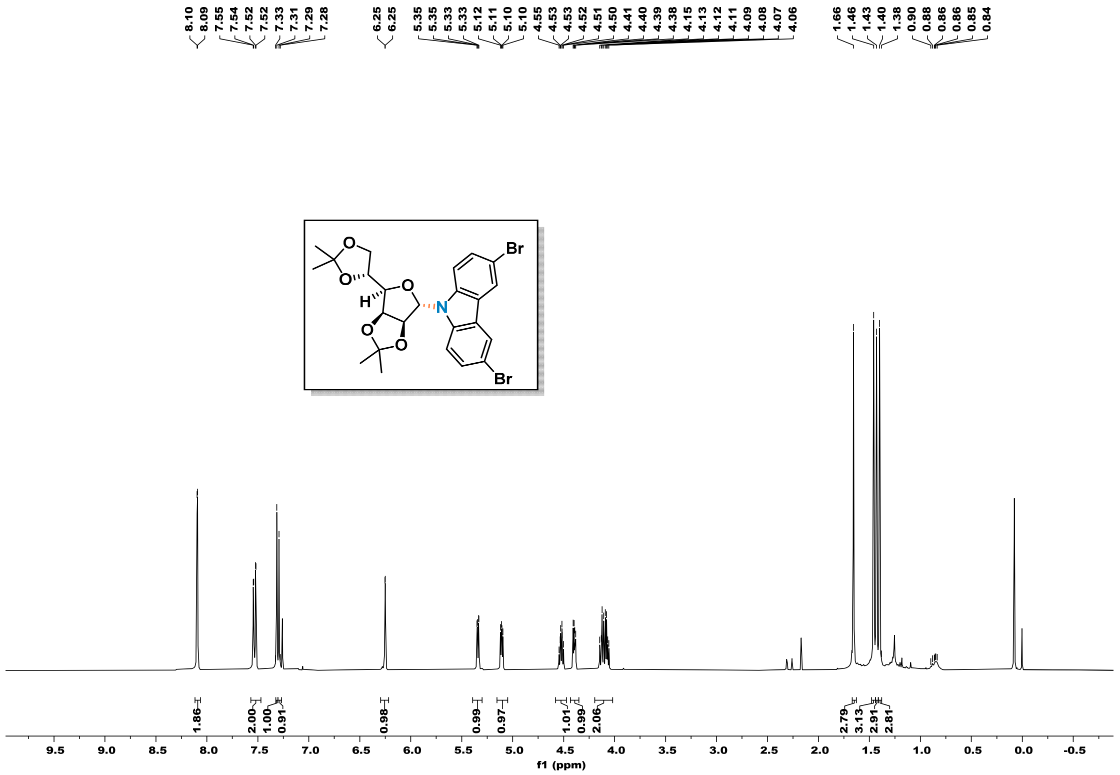

Supplementary Figure 95 | ^{1}H NMR (400 MHz, $CDCl_3$) **(4f)**

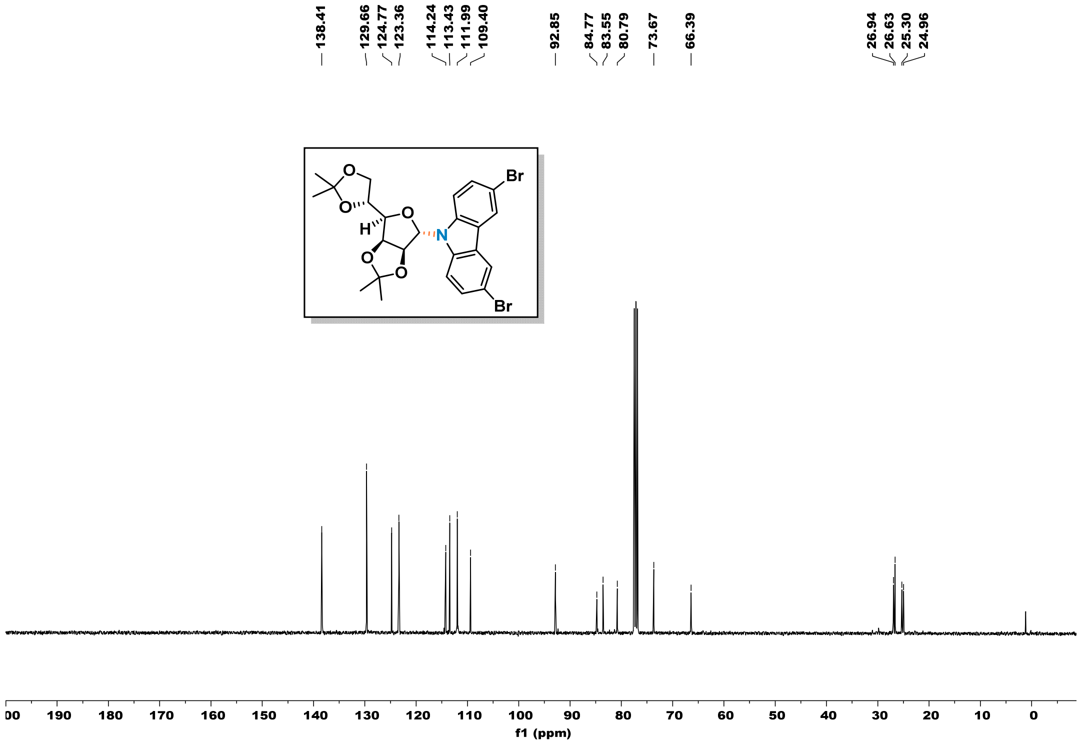

Supplementary Figure 96 | ^{13}C NMR (101 MHz, $CDCl_3$) **(4f)**

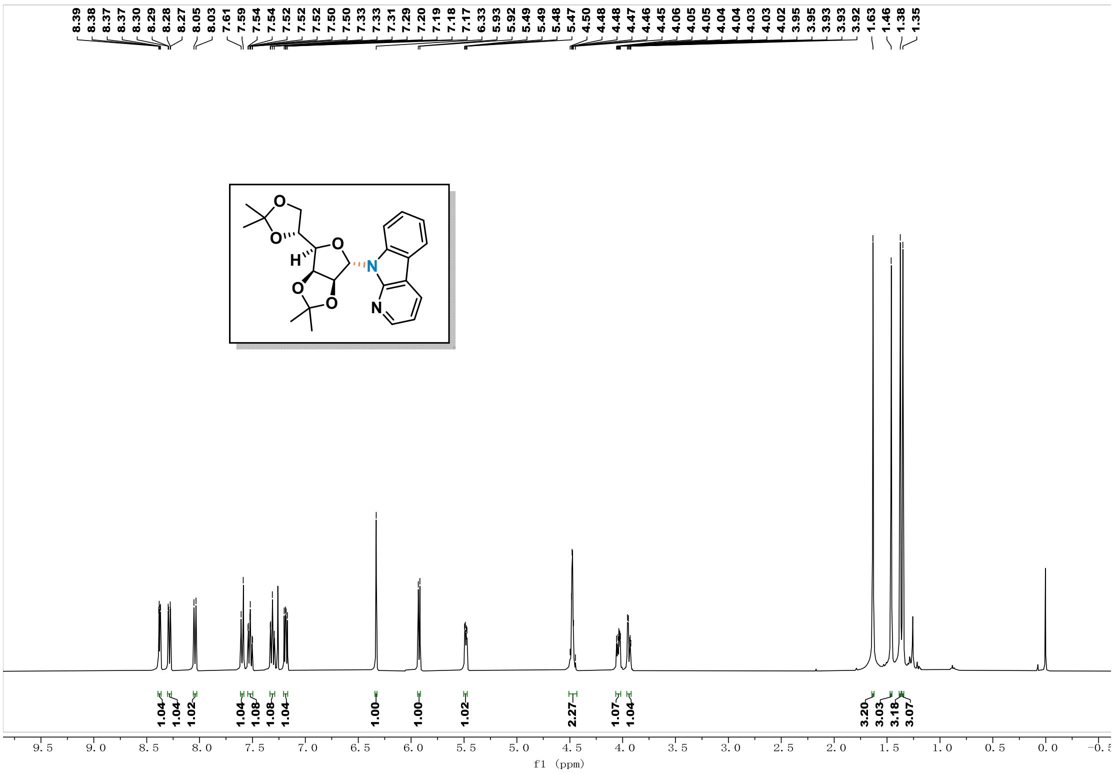

Supplementary Figure 97 | ^{1}H NMR (400 MHz, $CDCl_3$) **(4g)**

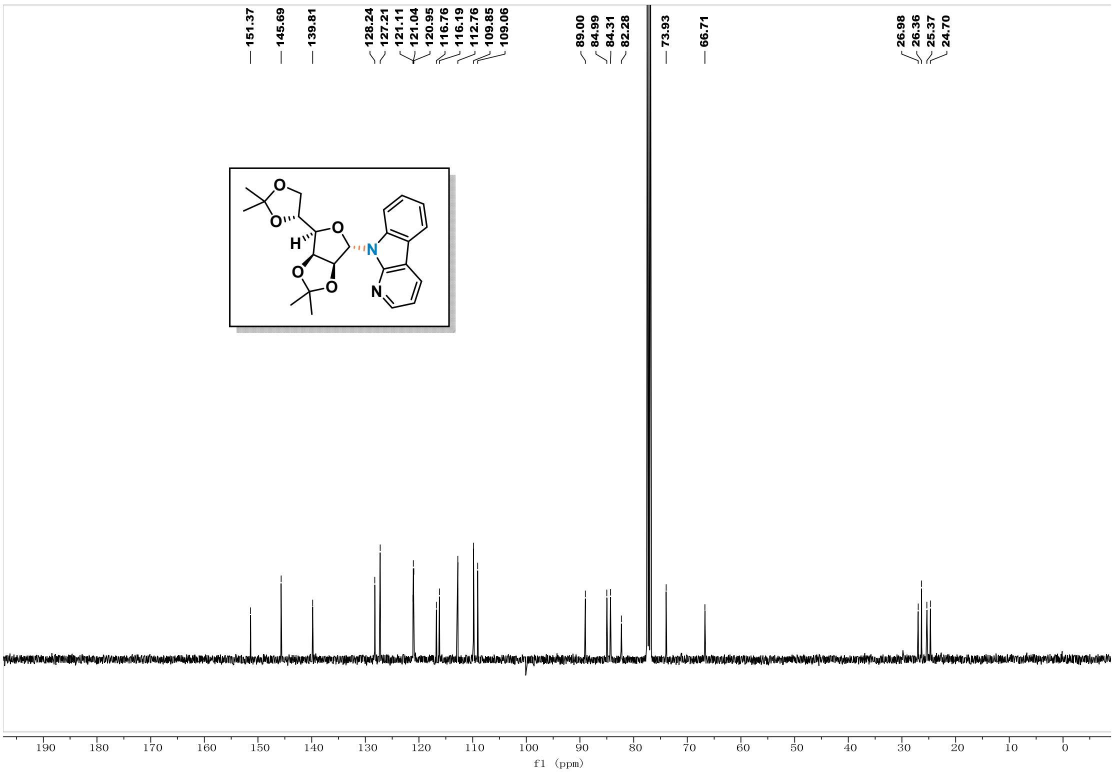

Supplementary Figure 98 | ^{13}C NMR (101 MHz, $CDCl_3$) **(4g)**

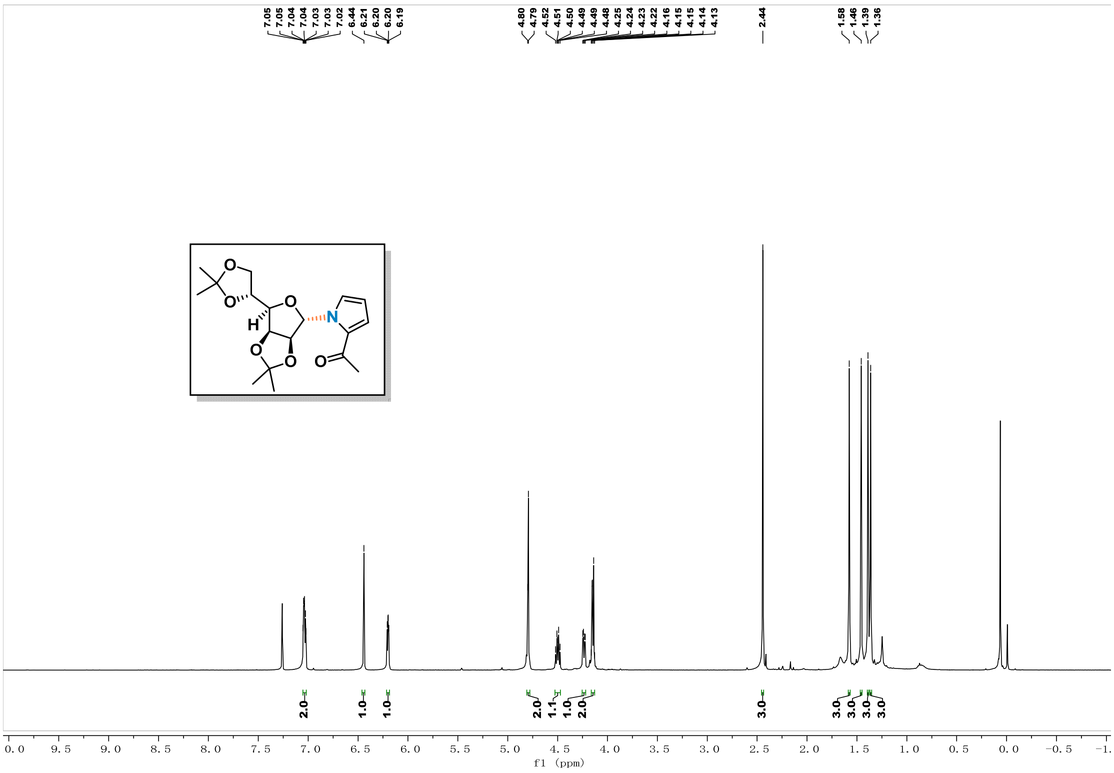

Supplementary Figure 99 | ^{1}H NMR (400 MHz, $CDCl_3$) **(4h)**

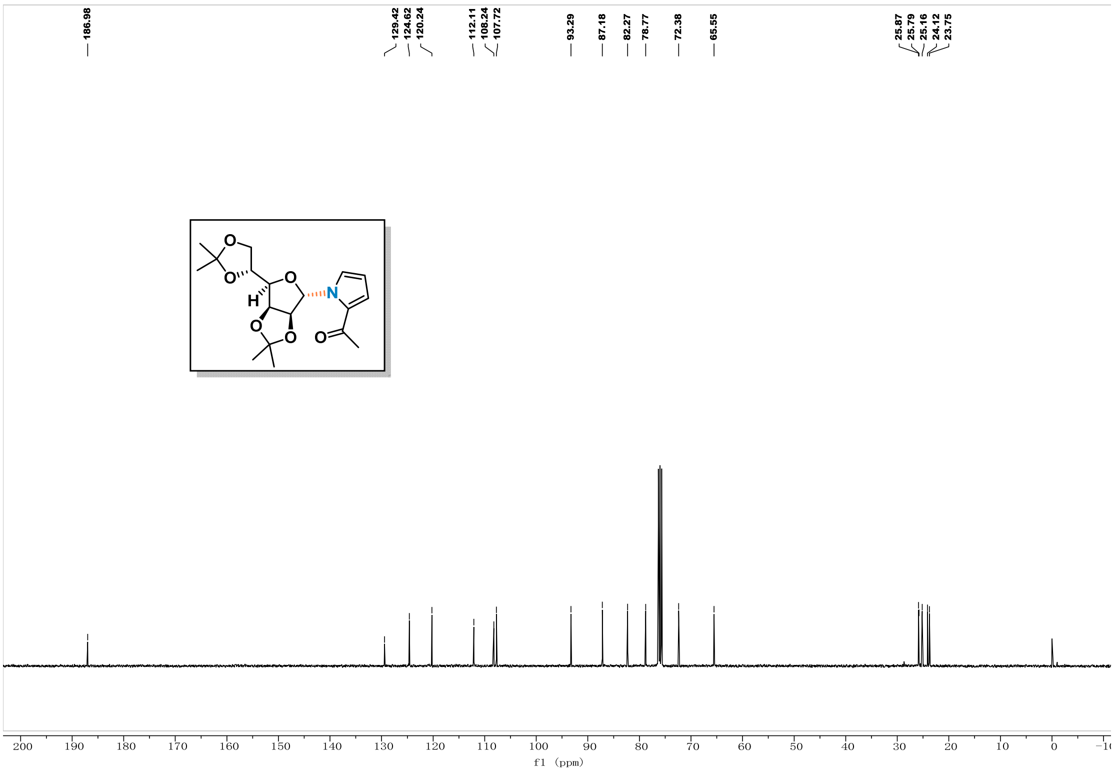

Supplementary Figure 100 | ^{13}C NMR (101 MHz, $CDCl_3$) **(4h)**

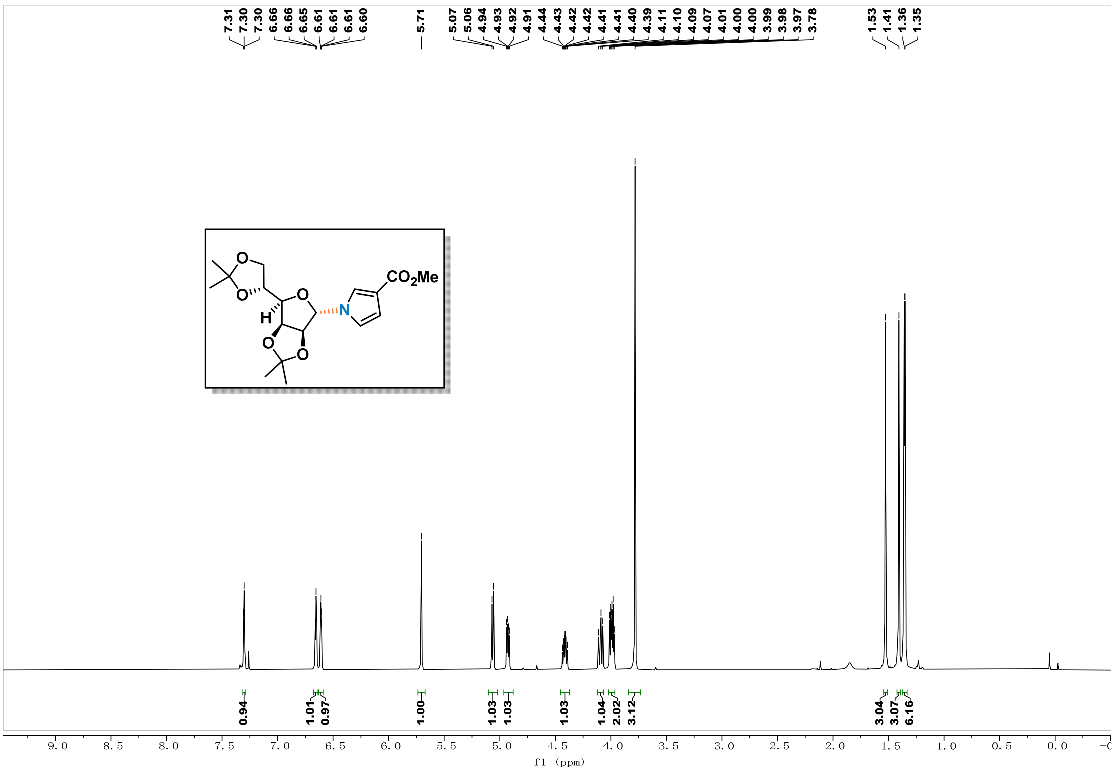

Supplementary Figure 101 | ^{1}H NMR (400 MHz, CDCl$_3$) **(4i)**

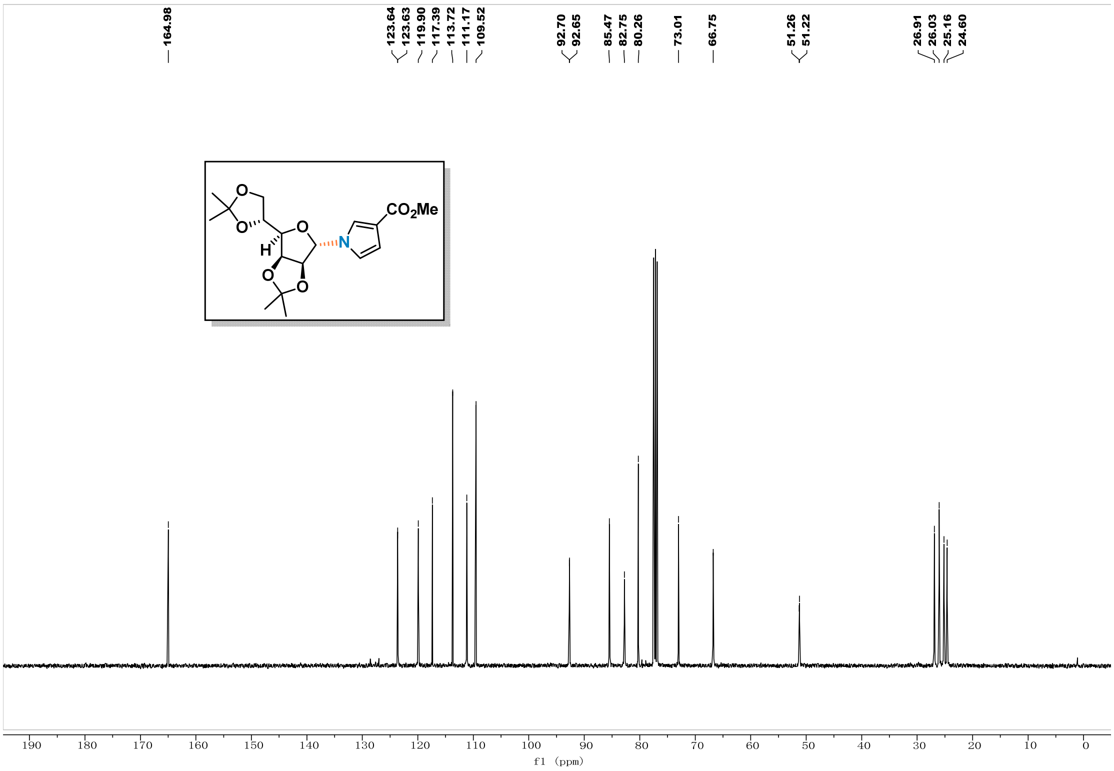

Supplementary Figure 102 | ^{13}C NMR (101 MHz, CDCl$_3$) **(4i)**

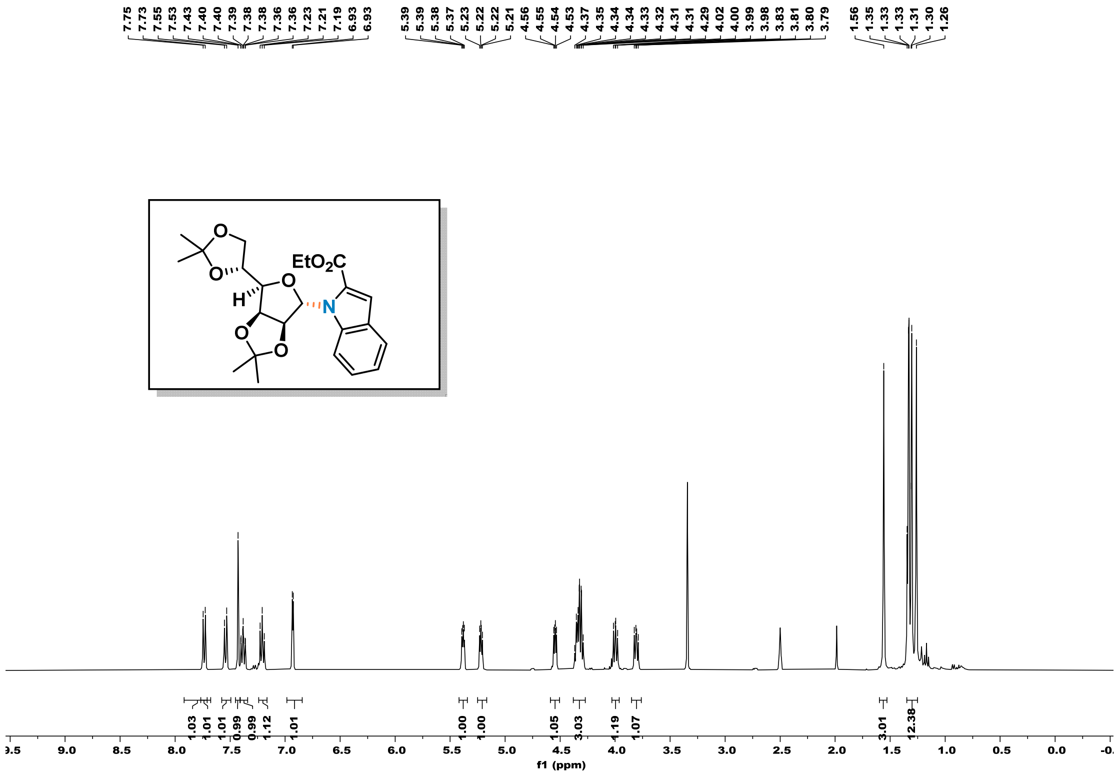

Supplementary Figure 103 | ^{1}H NMR (400 MHz, DMSO-*d6*) **(5a)**

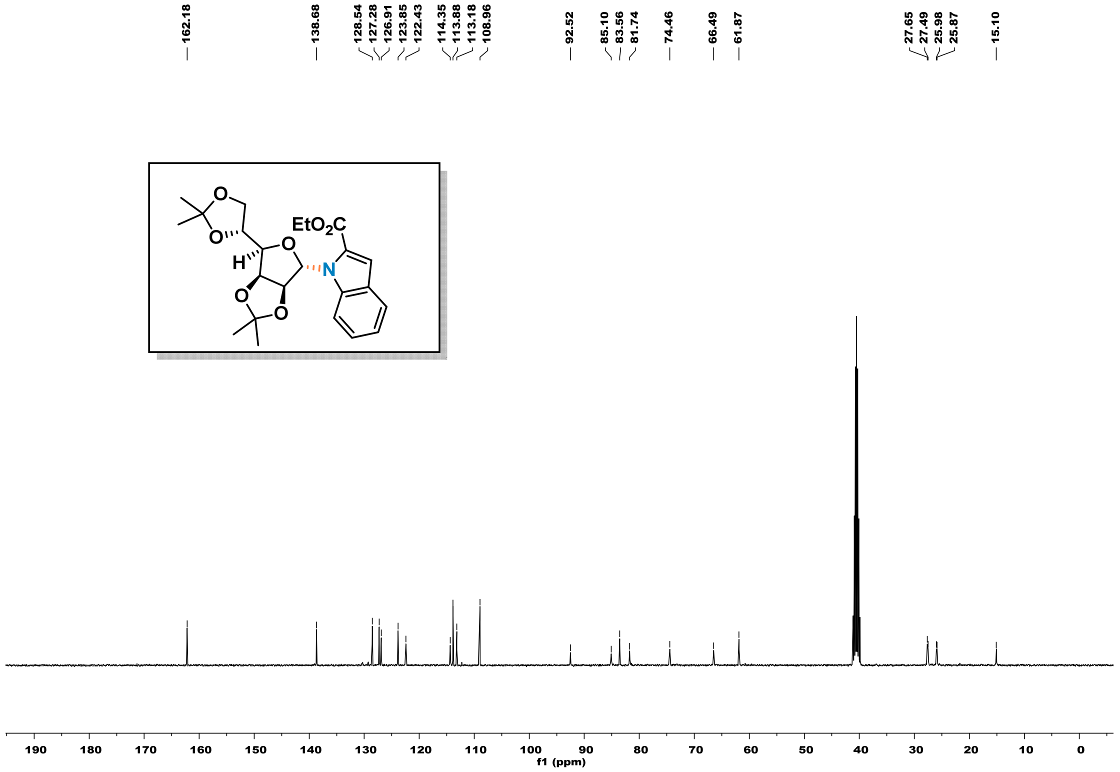

Supplementary Figure 104 | ^{13}C NMR (101 MHz, DMSO-*d6*) **(5a)**

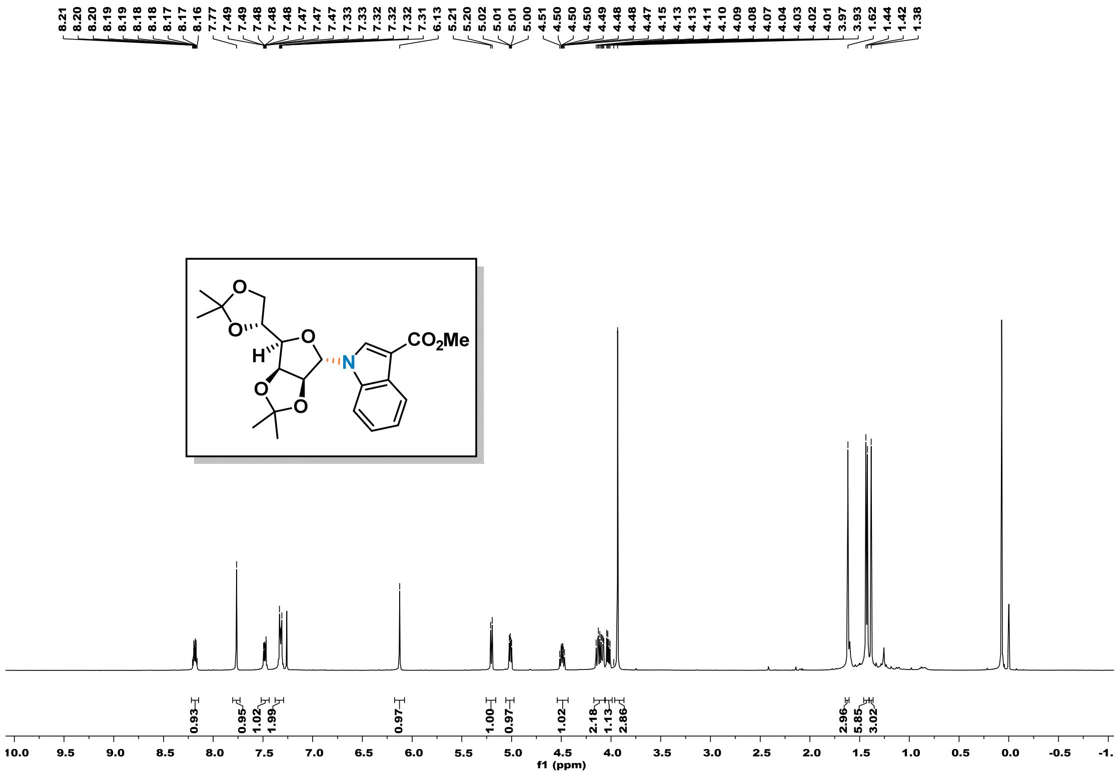

Supplementary Figure 105 | ^{1}H NMR (400 MHz, $CDCl_3$) **(5b)**

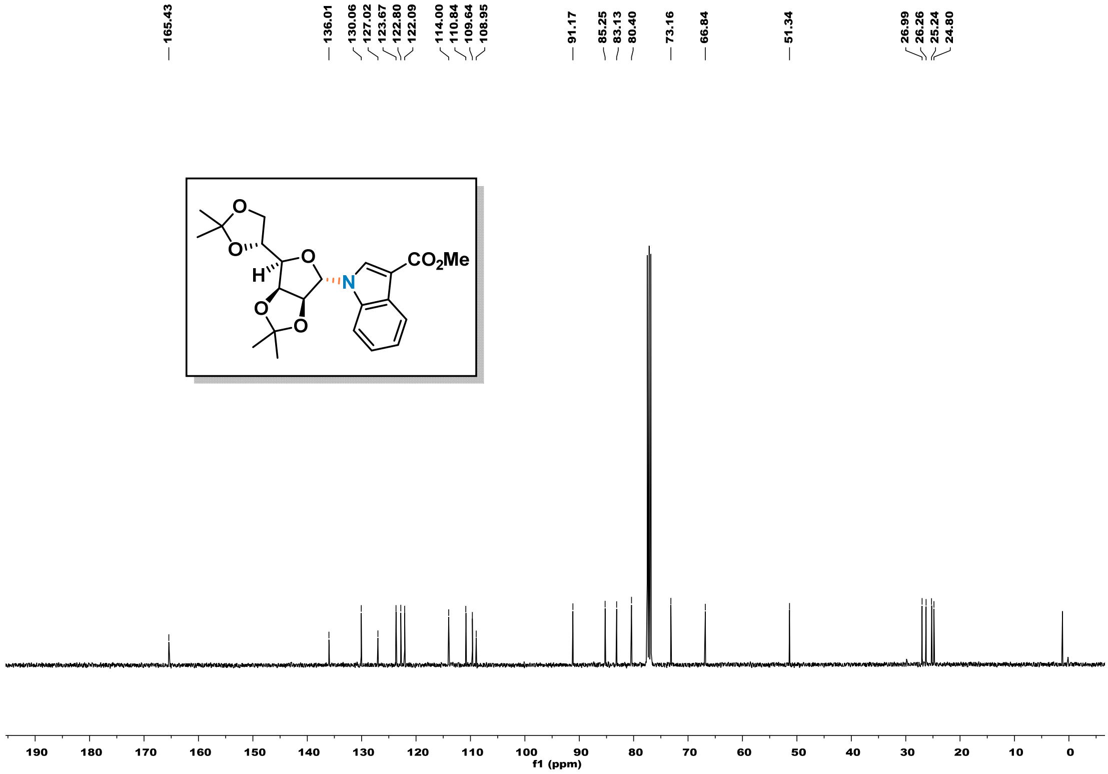

Supplementary Figure 106 | ^{13}C NMR (101 MHz, $CDCl_3$) **(5b)**

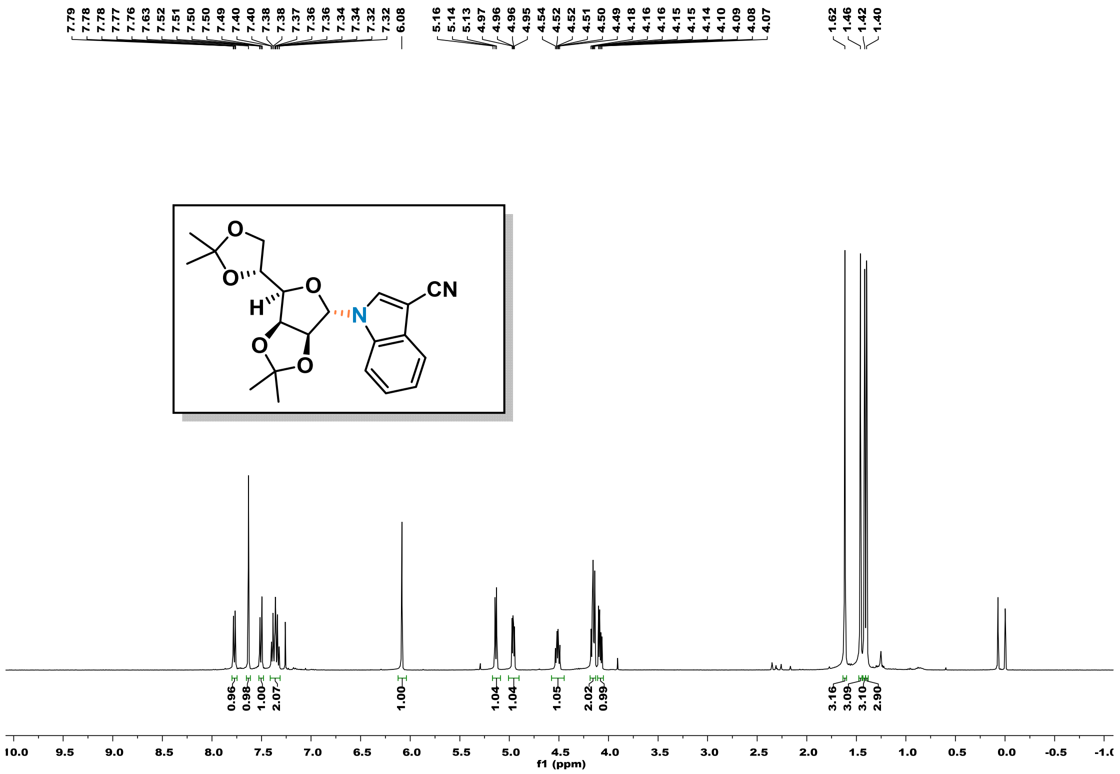

Supplementary Figure 107 | ^{1}H NMR (400 MHz, $CDCl_3$) **(5c)**

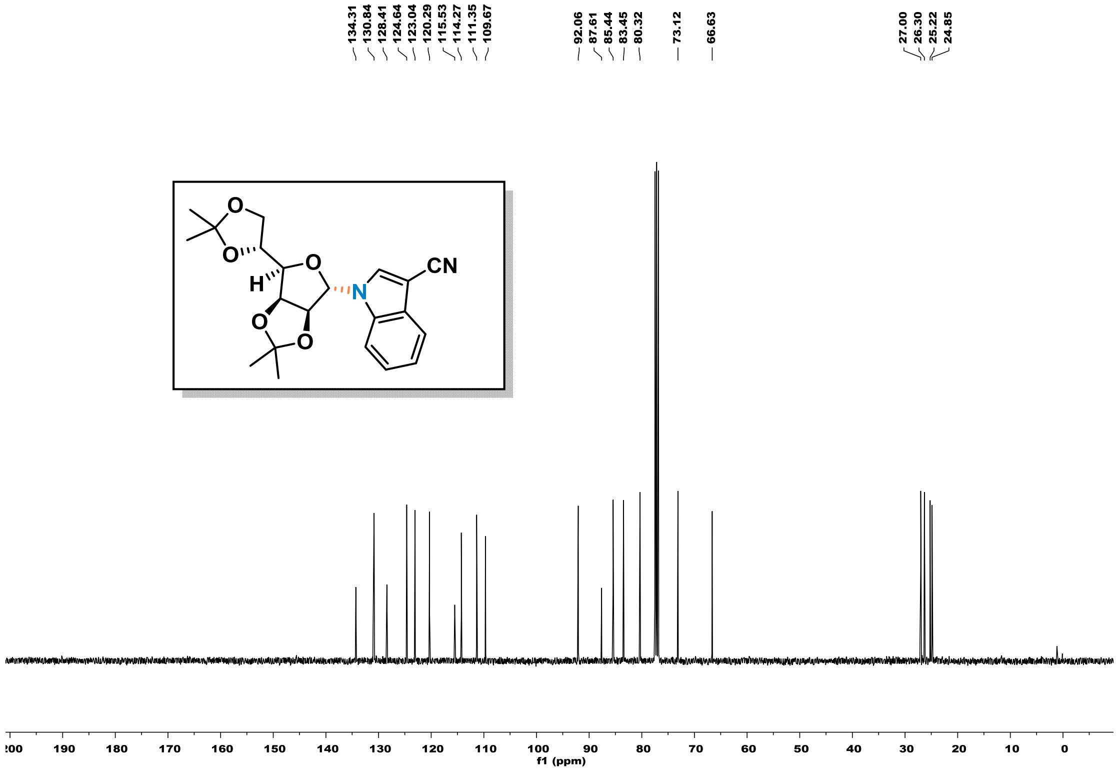

Supplementary Figure 108 | ^{13}C NMR (101 MHz, $CDCl_3$) **(5c)**

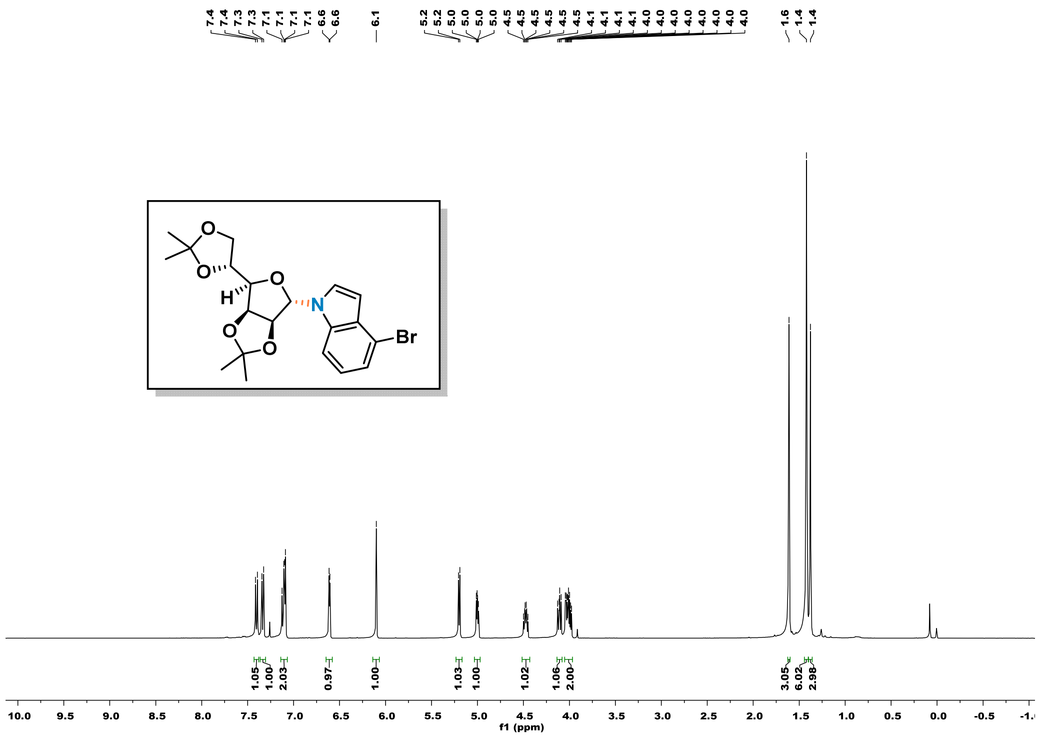

Supplementary Figure 109 | ^{1}H NMR (400 MHz, $CDCl_3$) **(5d)**

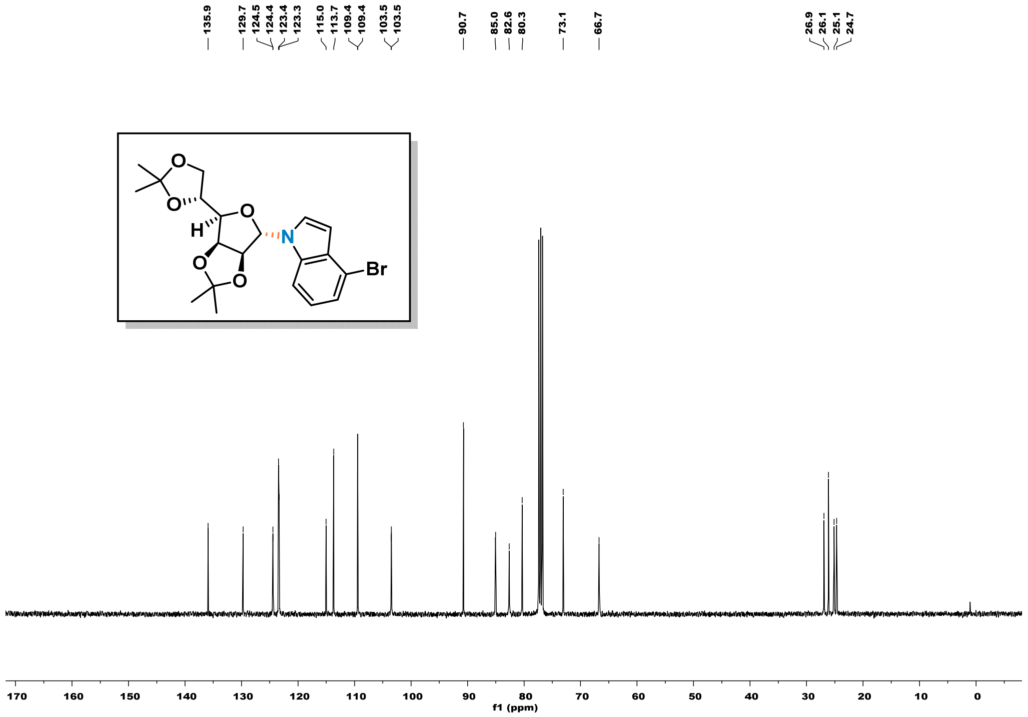

Supplementary Figure 110 | ^{13}C NMR (101 MHz, $CDCl_3$) **(5d)**

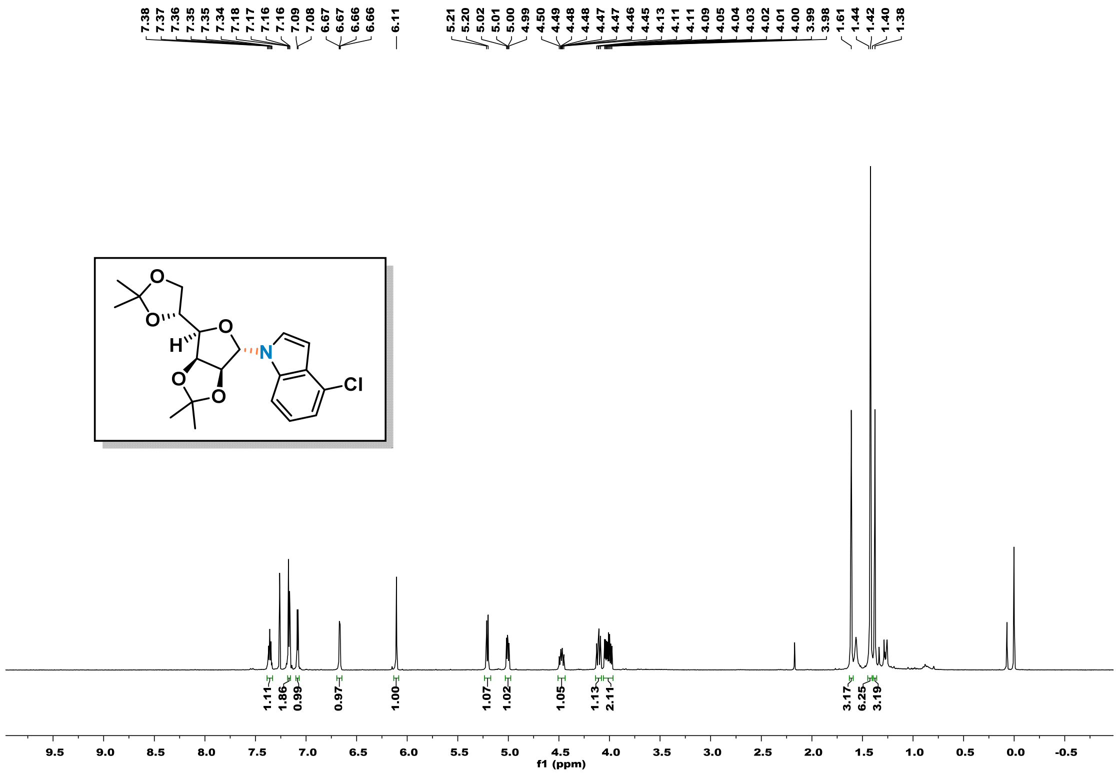

Supplementary Figure 112 | ^{1}H NMR (400 MHz, $CDCl_3$) **(5e)**

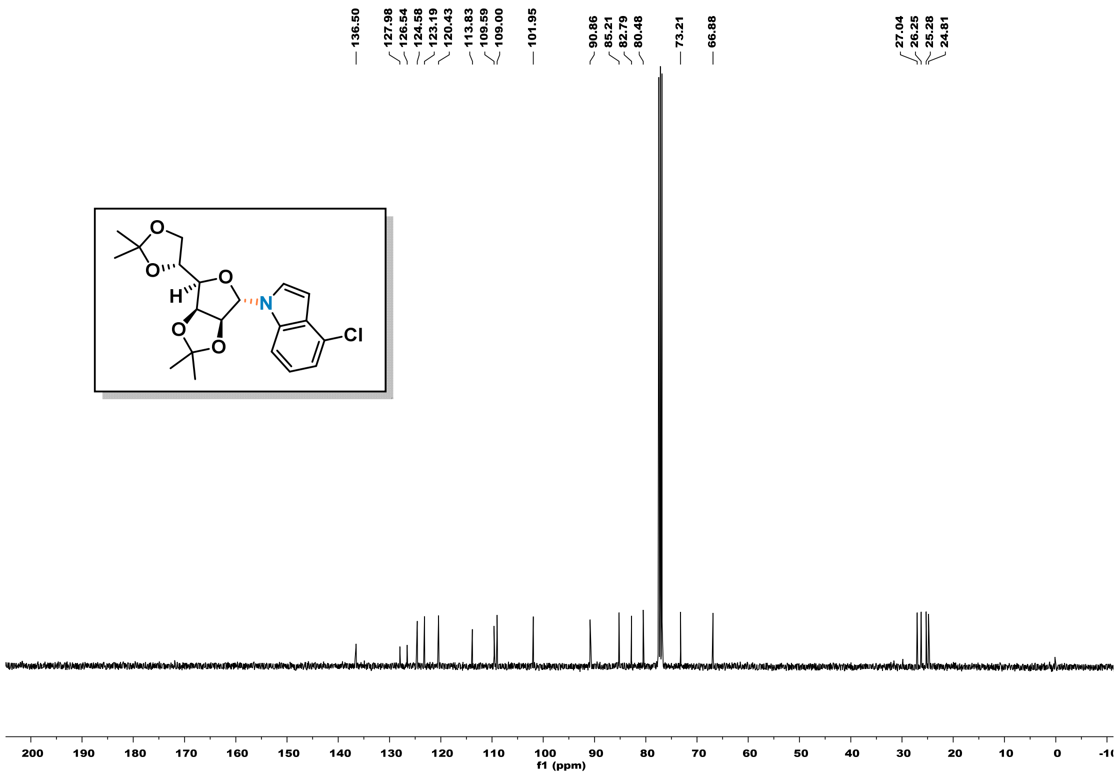

Supplementary Figure 113 | ^{13}C NMR (101 MHz, $CDCl_3$) **(5e)**

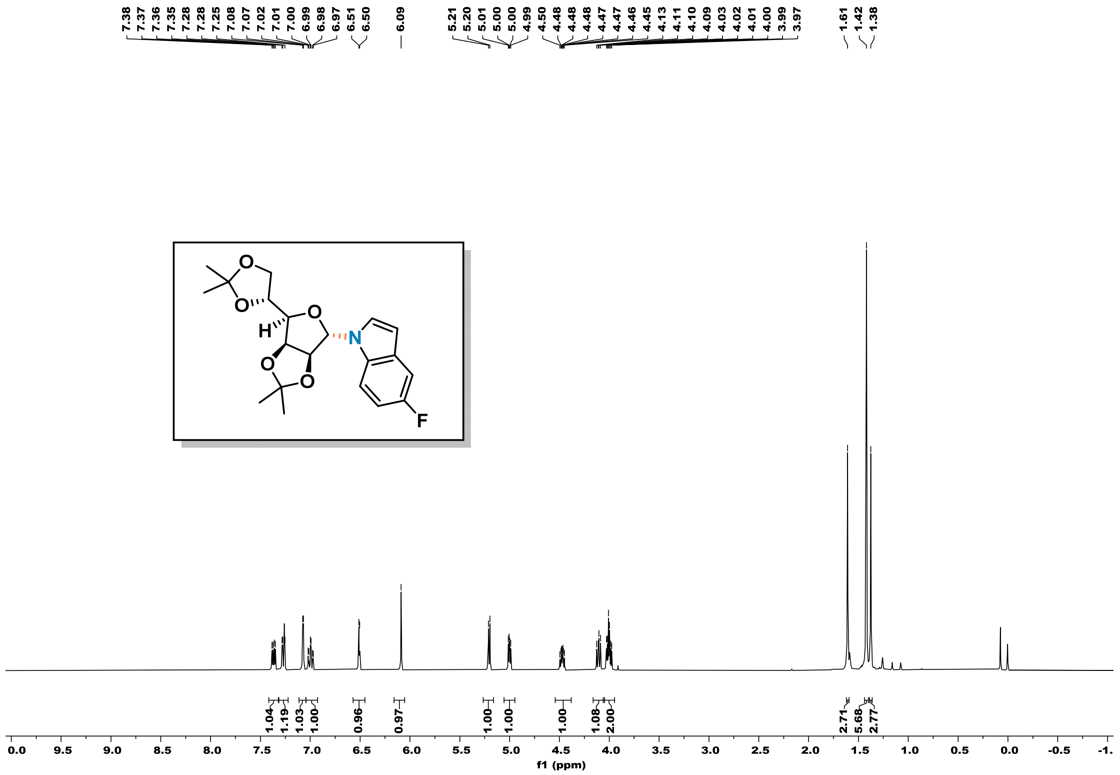

Supplementary Figure 114 | ^{1}H NMR (400 MHz, $CDCl_3$) **(5f)**

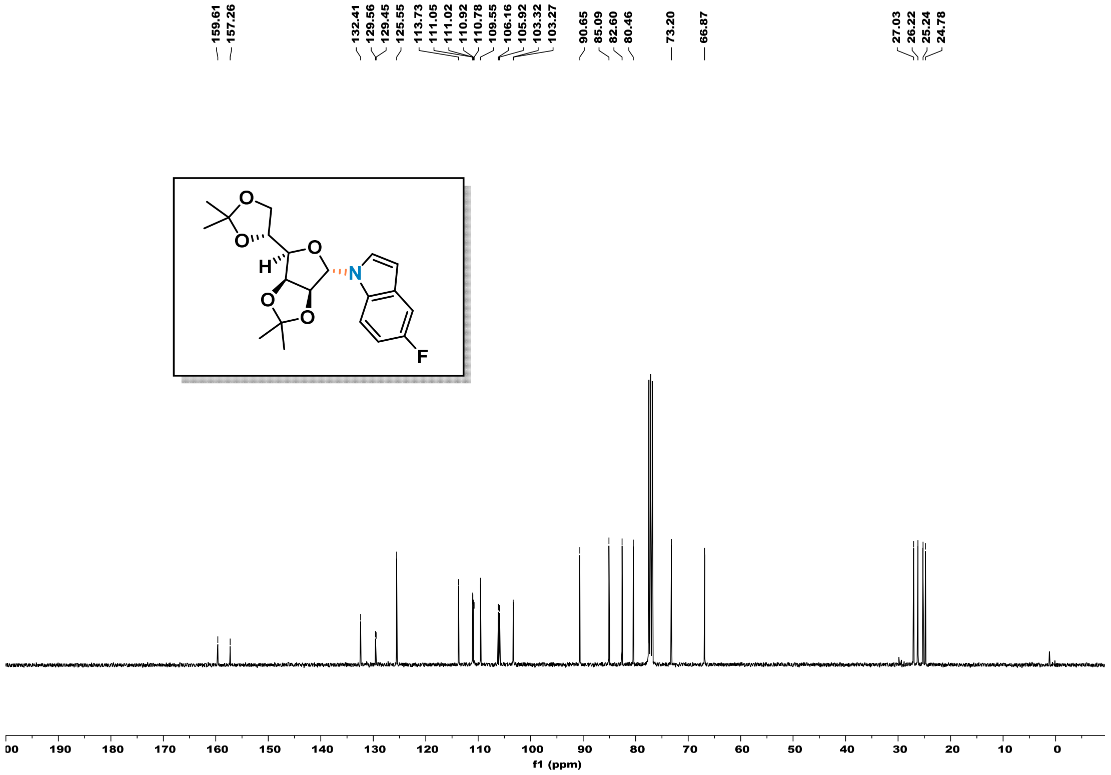

Supplementary Figure 115 | ^{13}C NMR (101 MHz, $CDCl_3$) **(5f)**

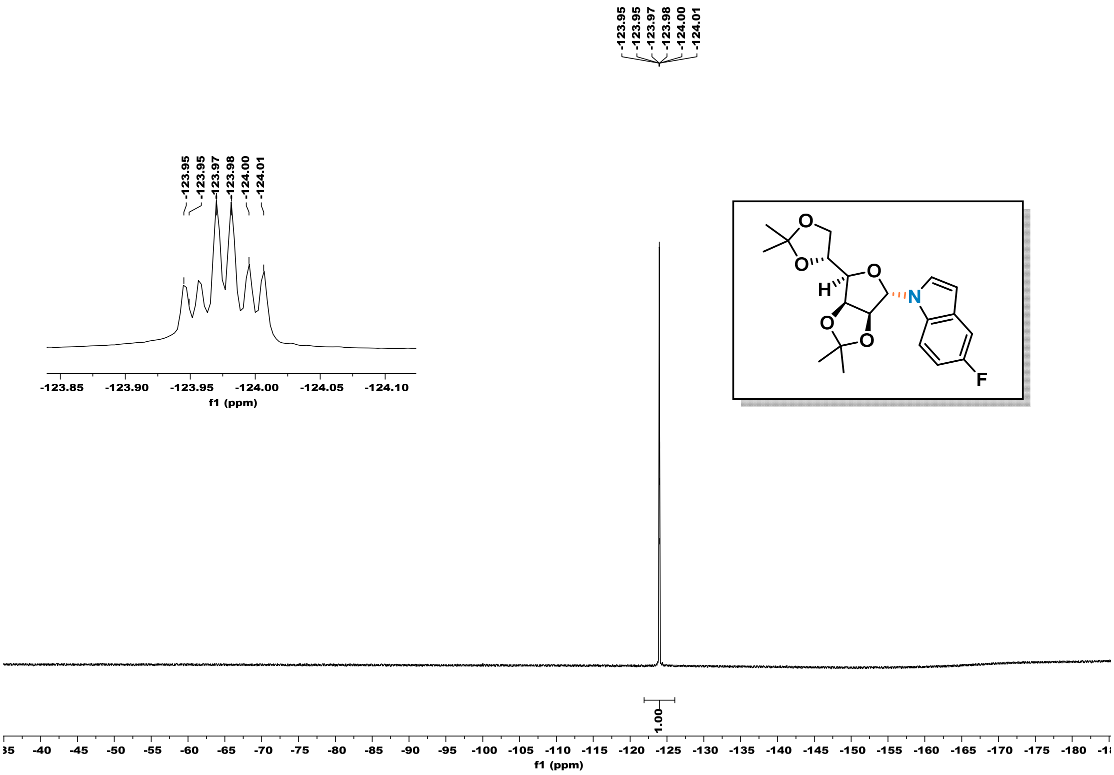

Supplementary Figure 116 | ^{19}F NMR (376 MHz, $CDCl_3$) **(5f)**

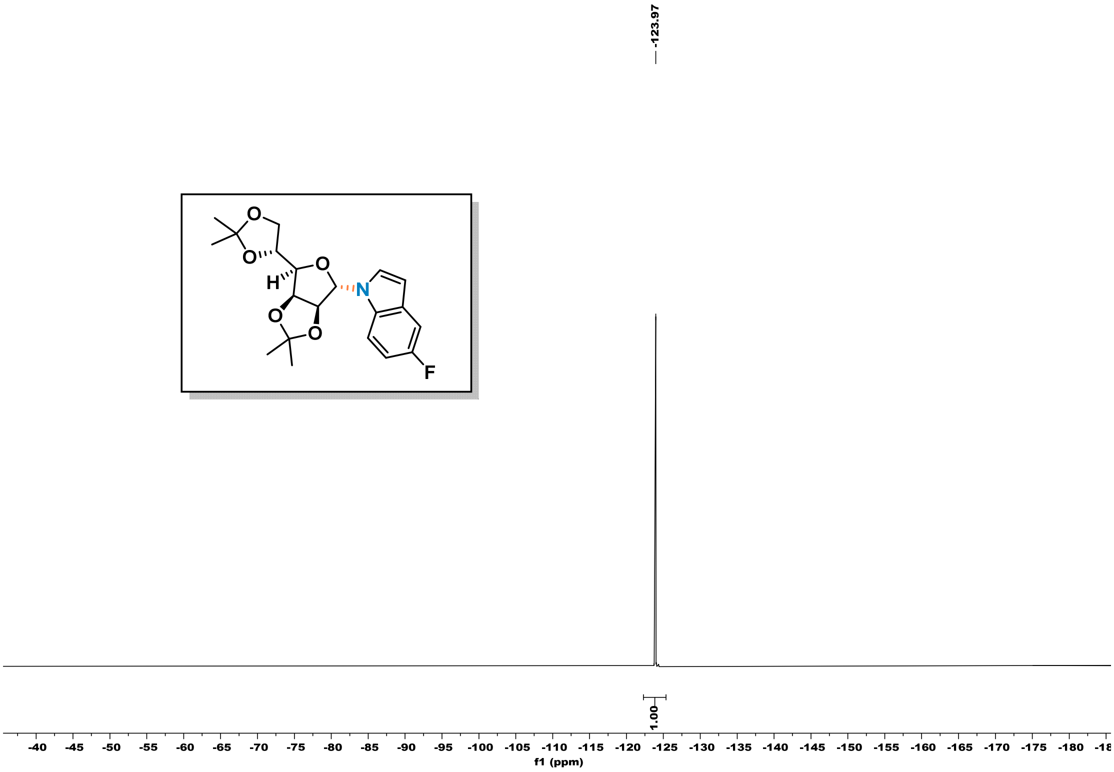

Supplementary Figure 117 | ^{19}F {^{1}H} NMR (376 MHz, $CDCl_3$) **(5f)**

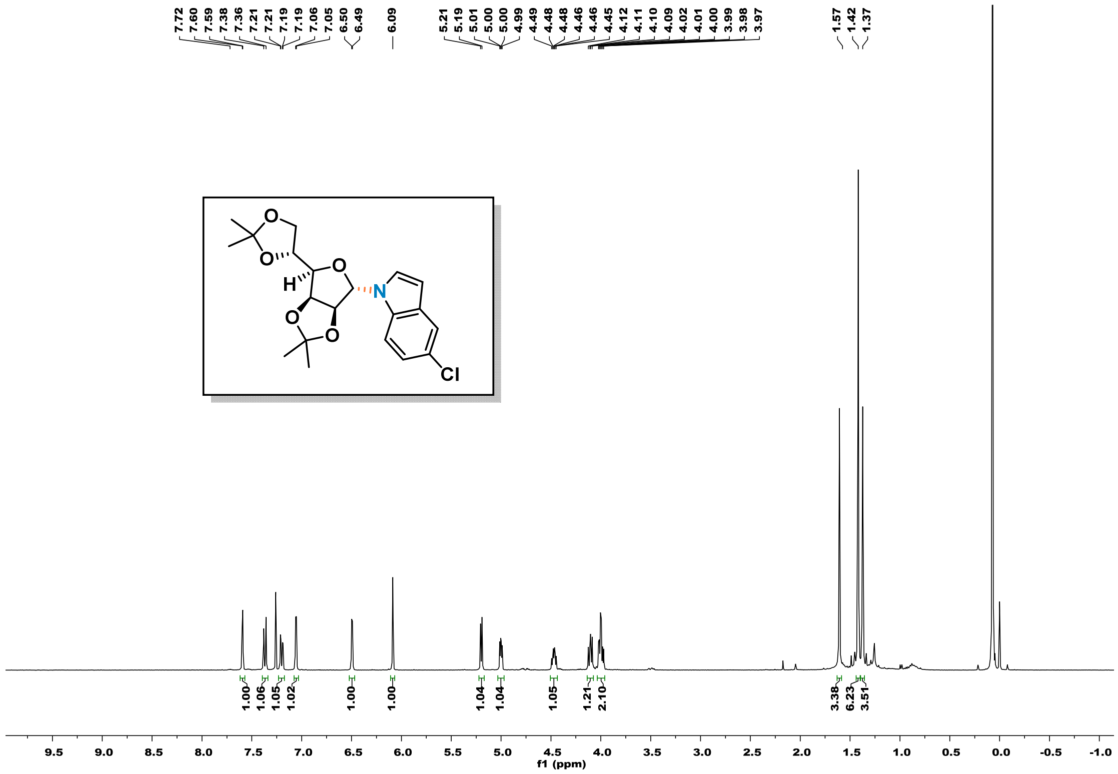

Supplementary Figure 118 | ^{1}H NMR (400 MHz, $CDCl_3$) **(5g)**

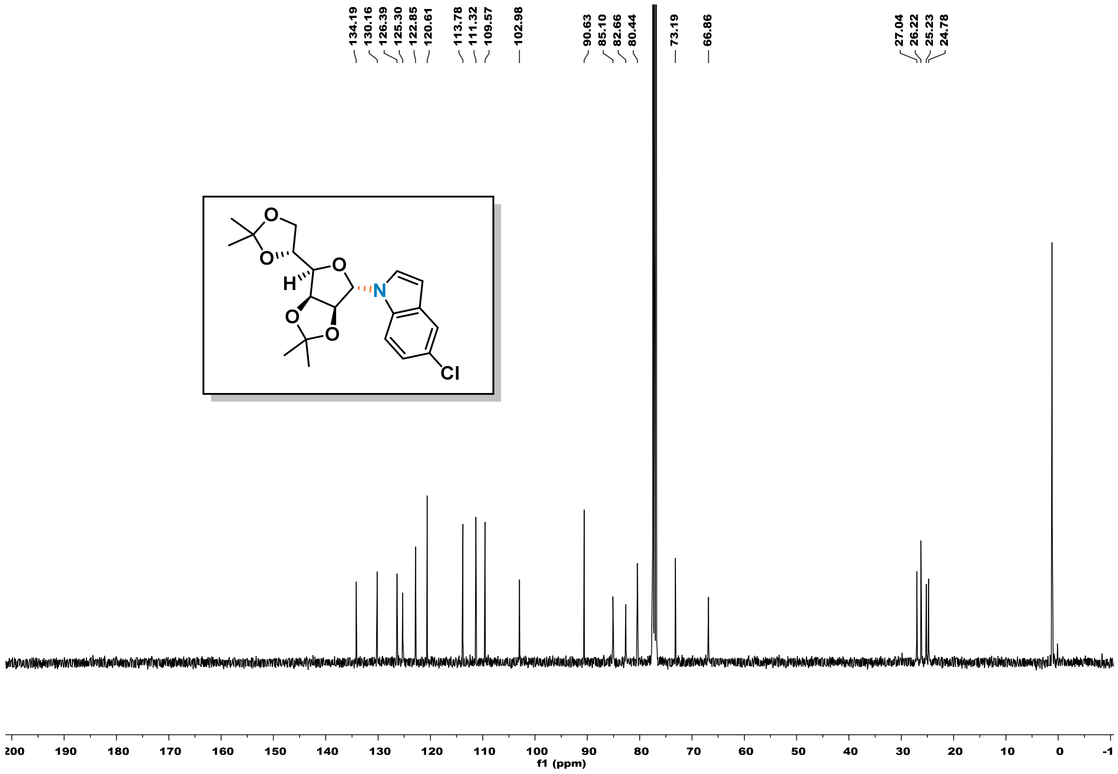

Supplementary Figure 119 | ^{13}C NMR (101 MHz, $CDCl_3$) **(5g)**

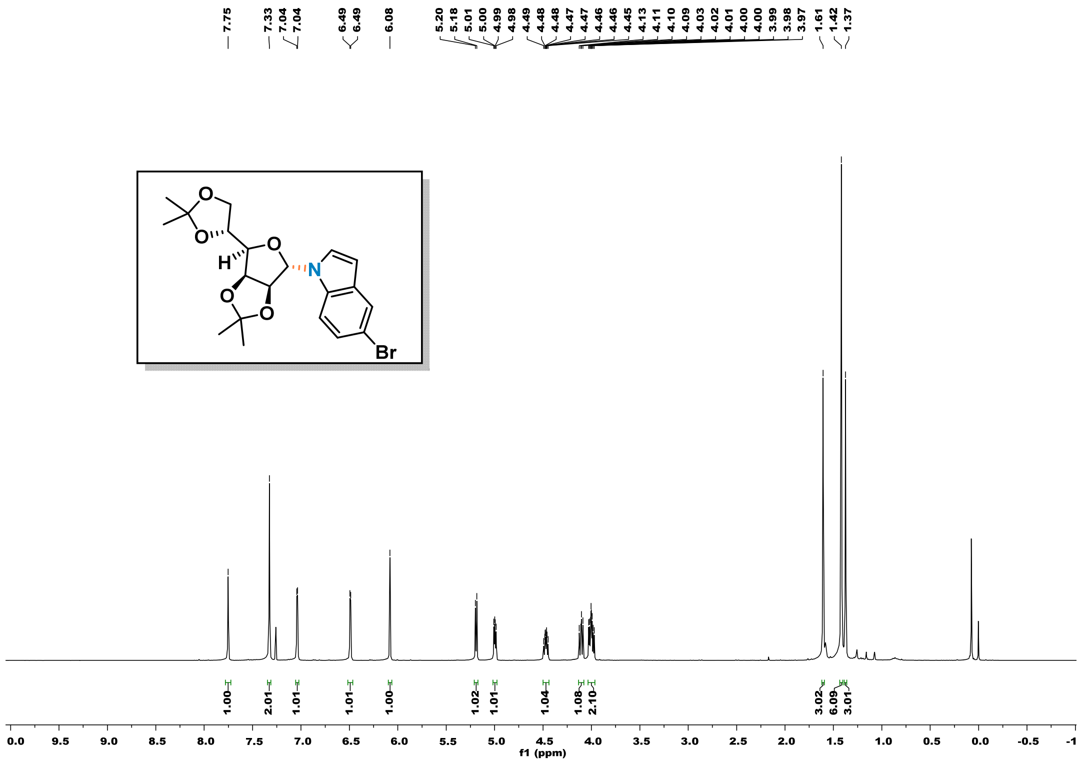

Supplementary Figure 120 | ^{1}H NMR (400 MHz, $CDCl_3$) **(5h)**

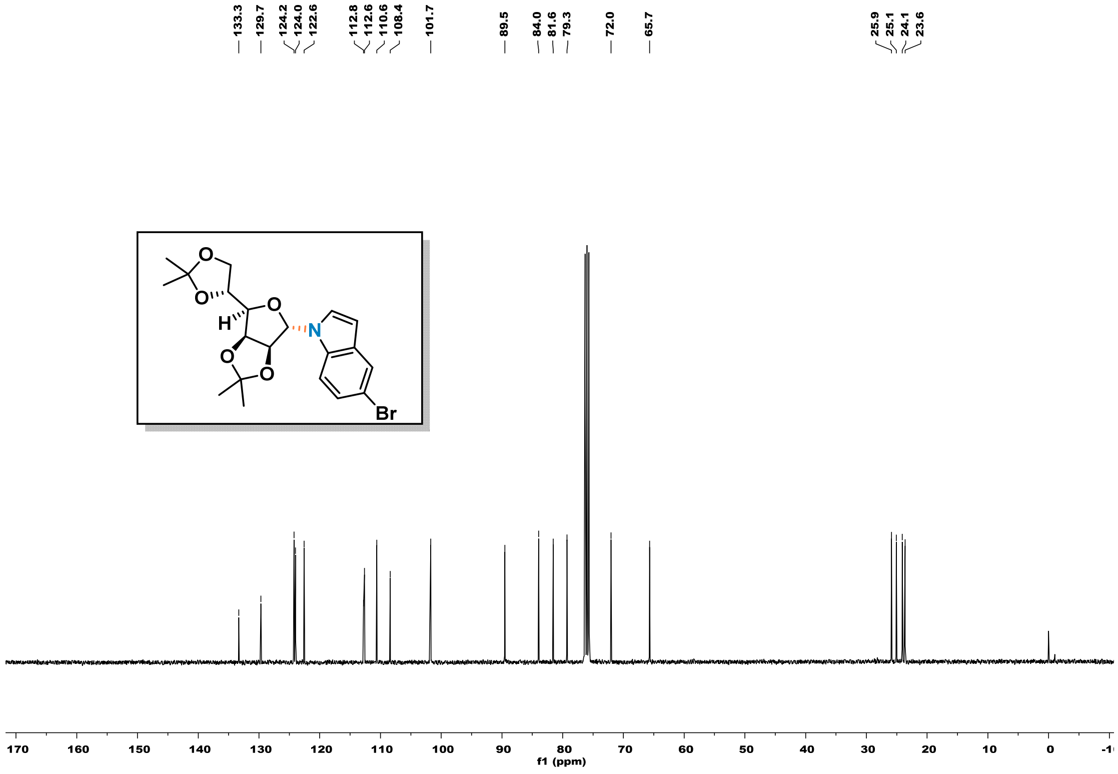

Supplementary Figure 121 | ^{13}C NMR (101 MHz, $CDCl_3$) **(5h)**

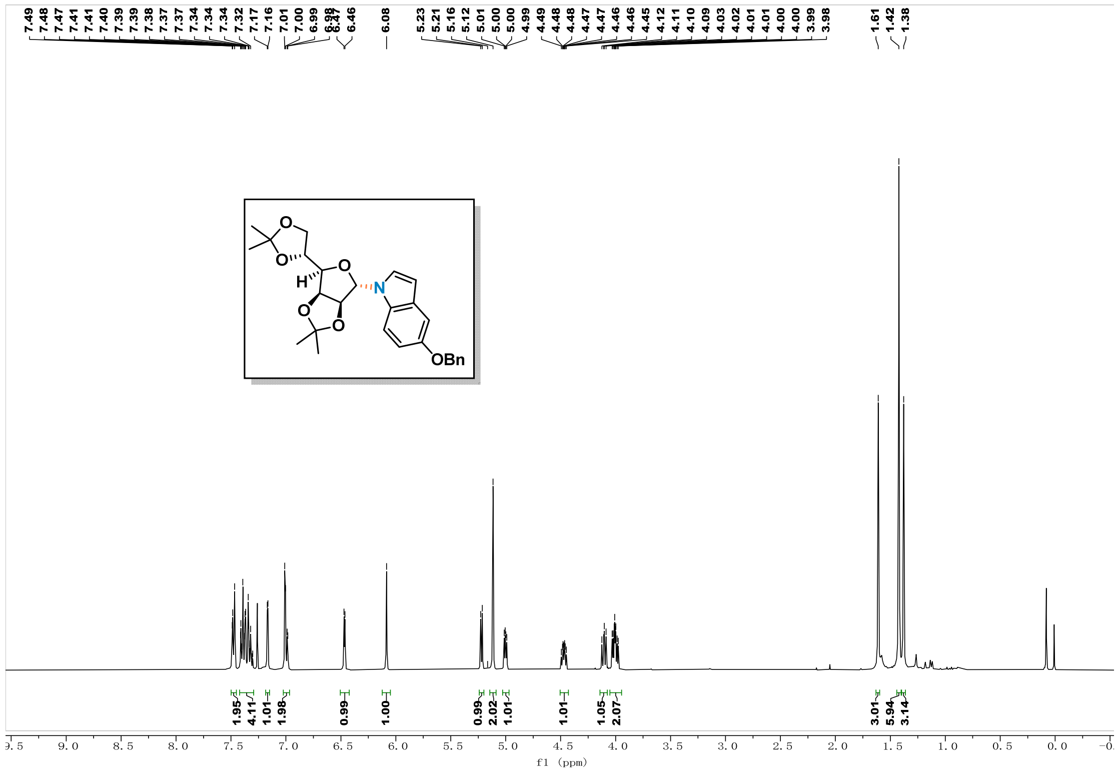

Supplementary Figure 122 | ^{1}H NMR (400 MHz, $CDCl_3$) **(5i)**

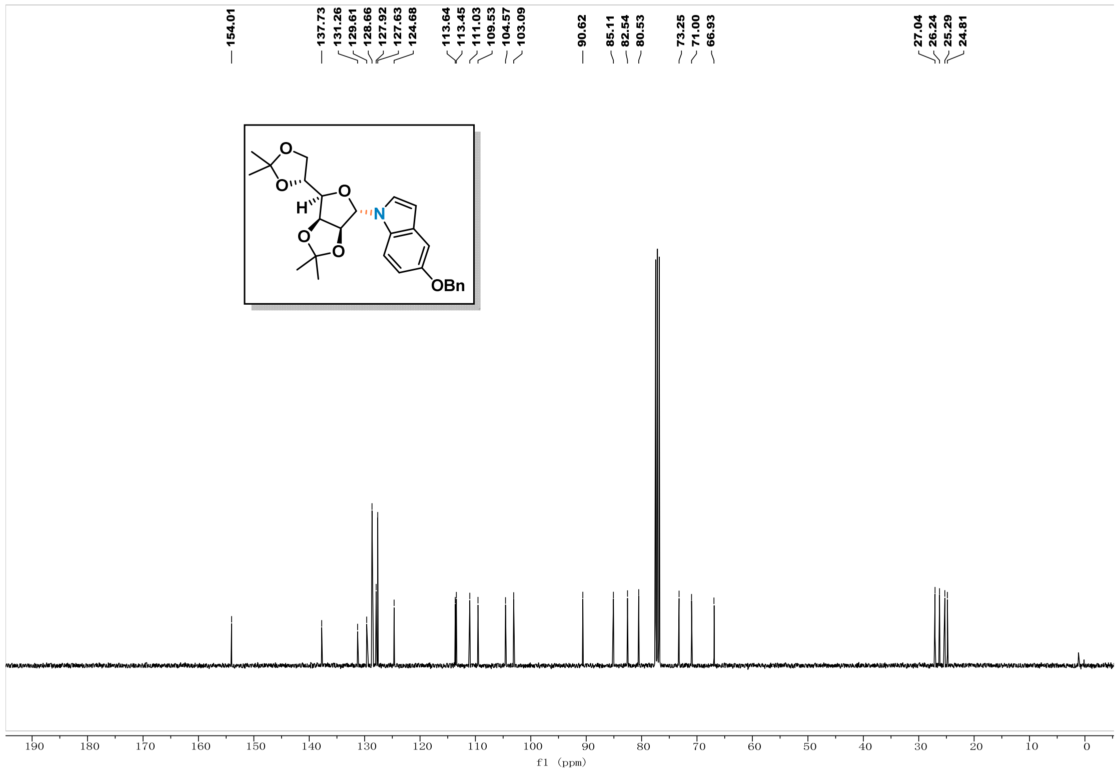

Supplementary Figure 123 | ^{13}C NMR (101 MHz, $CDCl_3$) **(5i)**

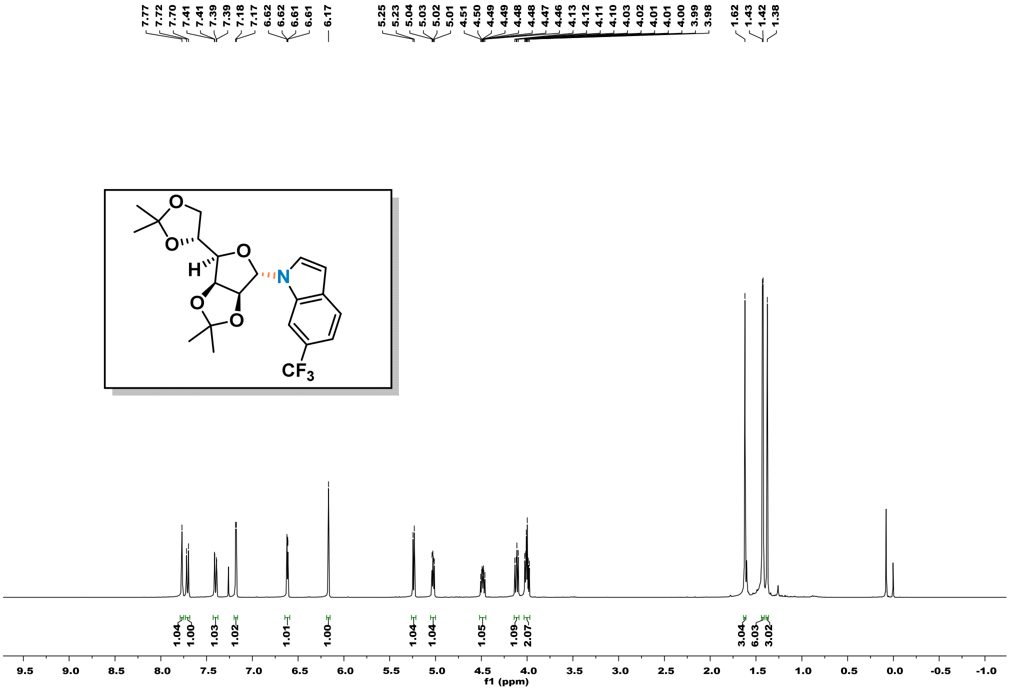

Supplementary Figure 124 | ^{1}H NMR (400 MHz, $CDCl_3$) **(5j)**

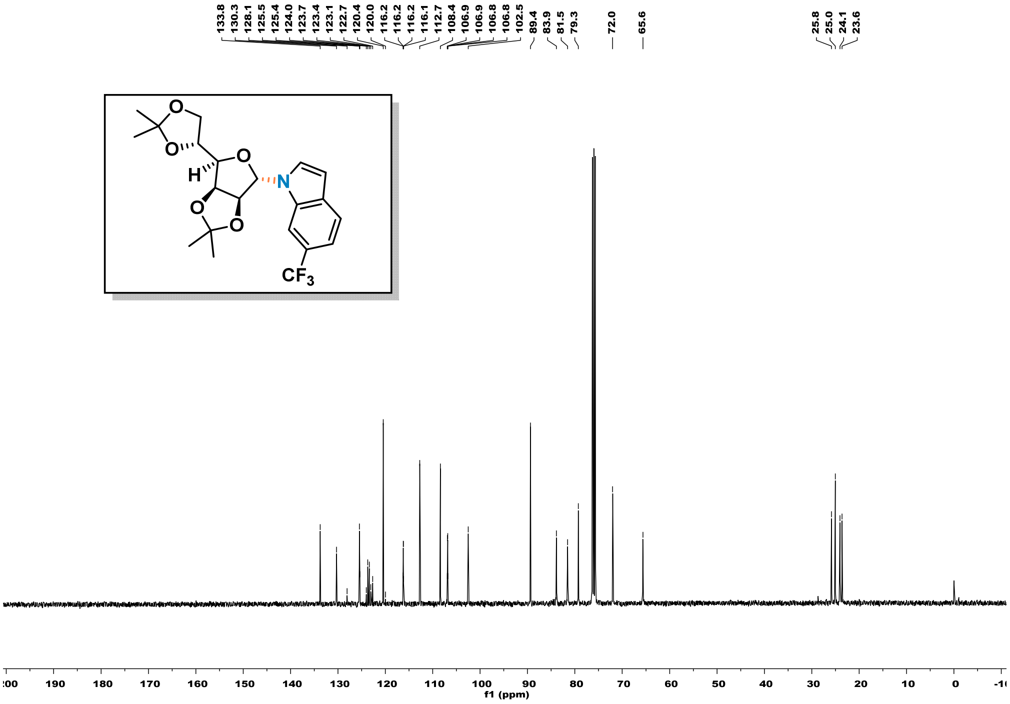

Supplementary Figure 125 | ^{13}C NMR (101 MHz, $CDCl_3$) **(5j)**

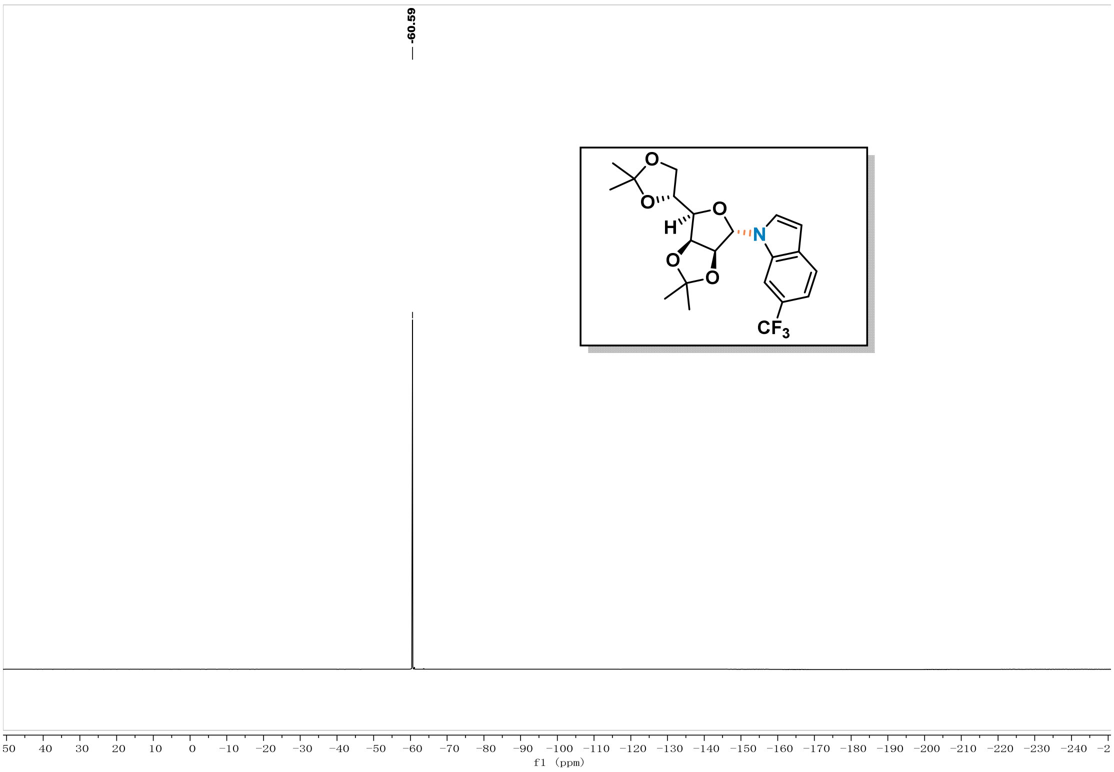

Supplementary Figure 126 | ^{19}F NMR (376 MHz , $CDCl_3$) **(5j)**

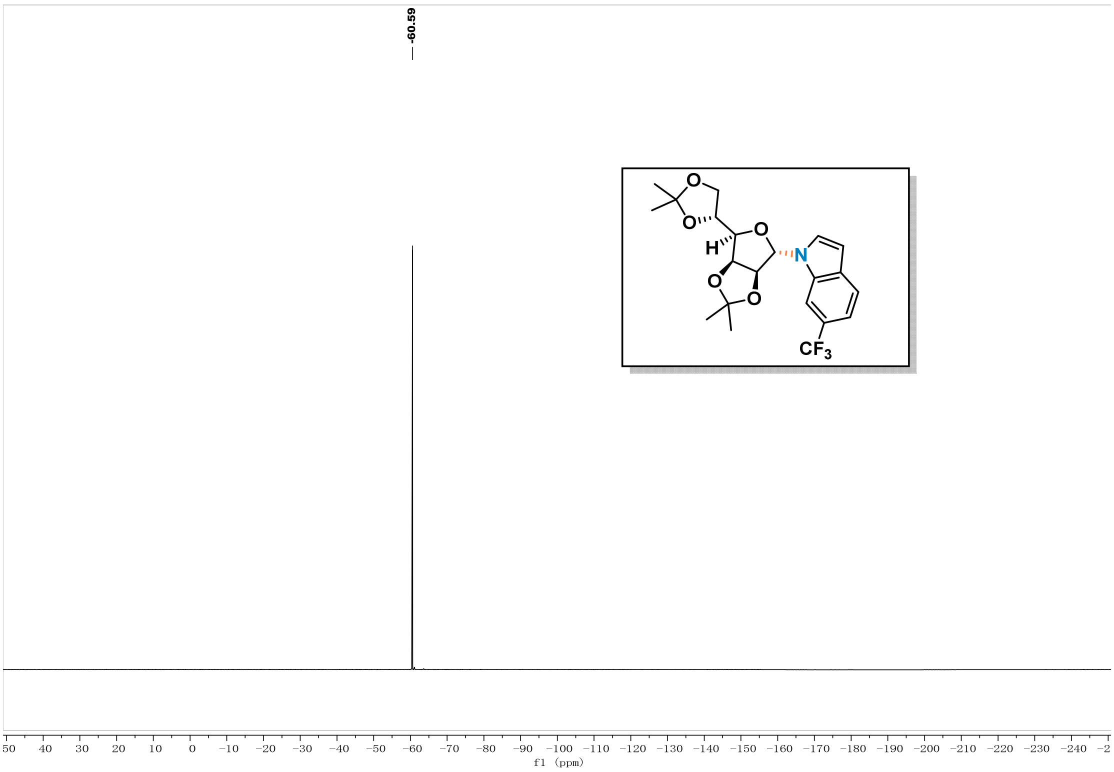

Supplementary Figure 127 | ^{19}F {^{1}H} NMR (376 MHz , $CDCl_3$) **(5j)**

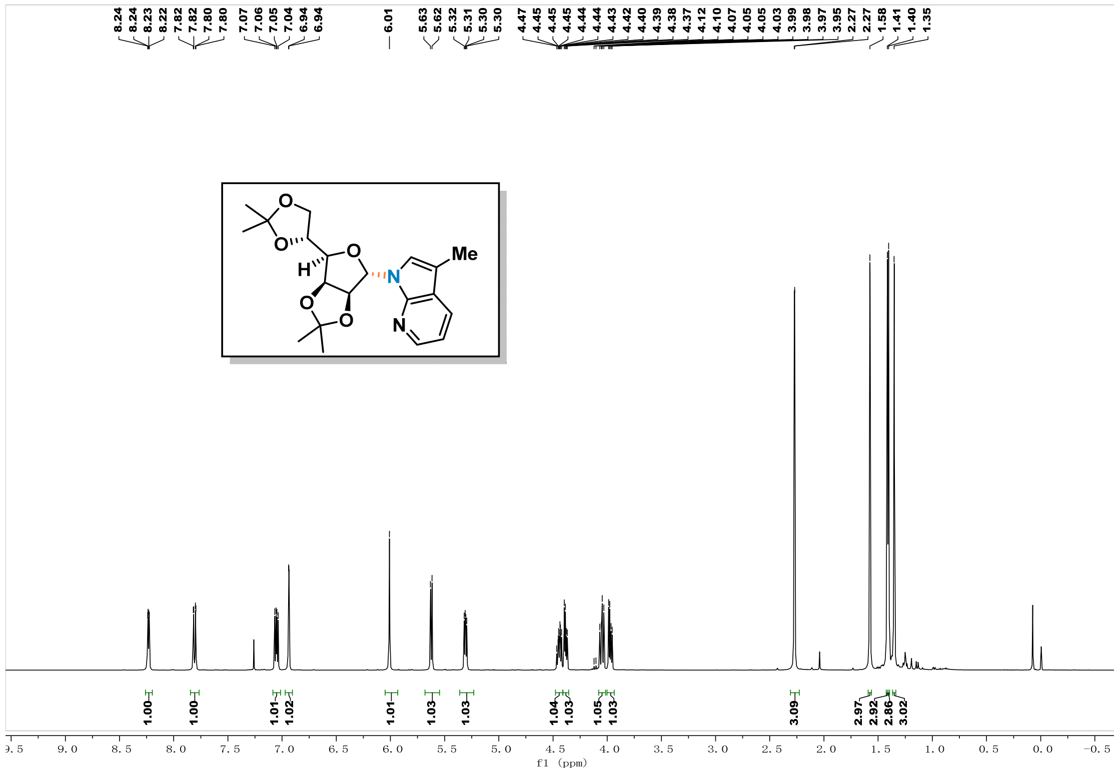

Supplementary Figure 128 | ^{1}H NMR (400 MHz, $CDCl_3$) **(5k)**

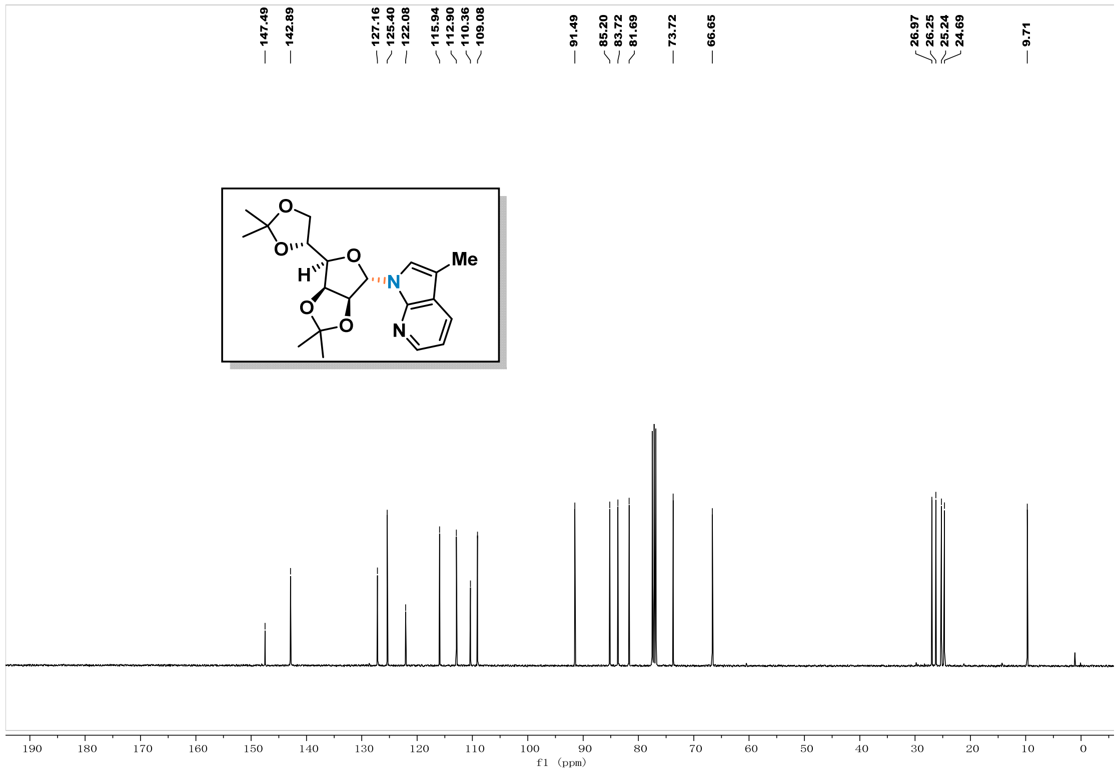

Supplementary Figure 129 | ^{13}C NMR (101 MHz, $CDCl_3$) **(5k)**

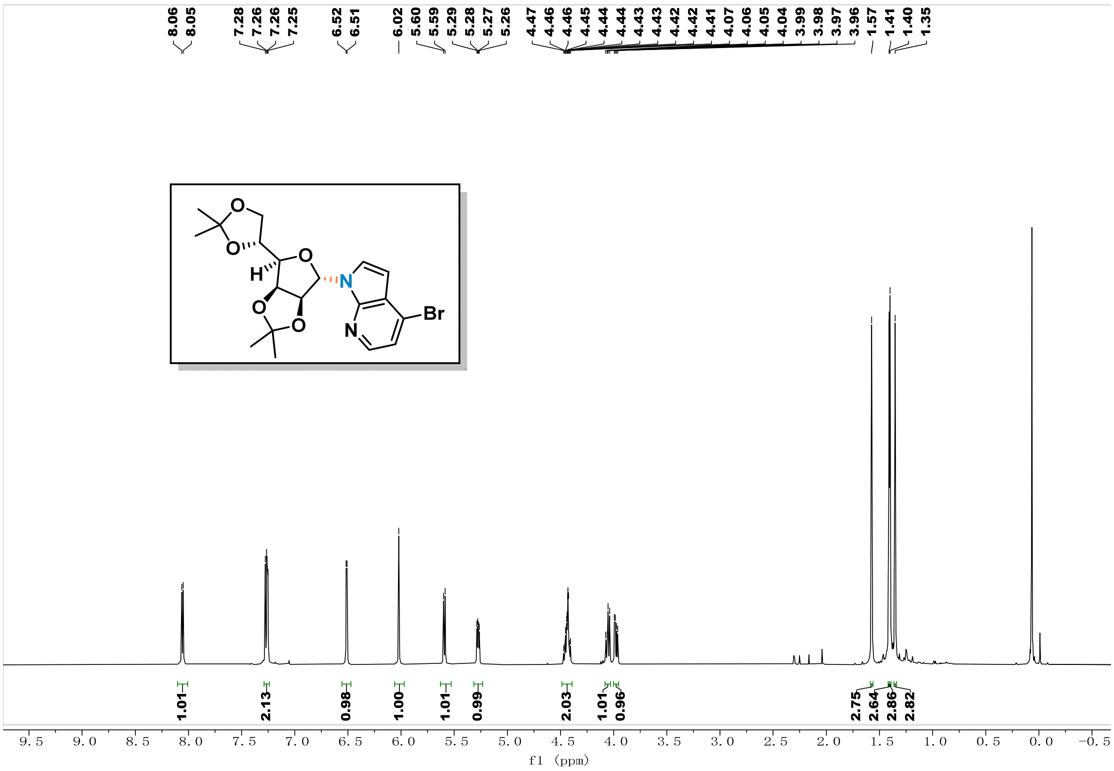

Supplementary Figure 130 | ^{1}H NMR (400 MHz, $CDCl_3$) **(5l)**

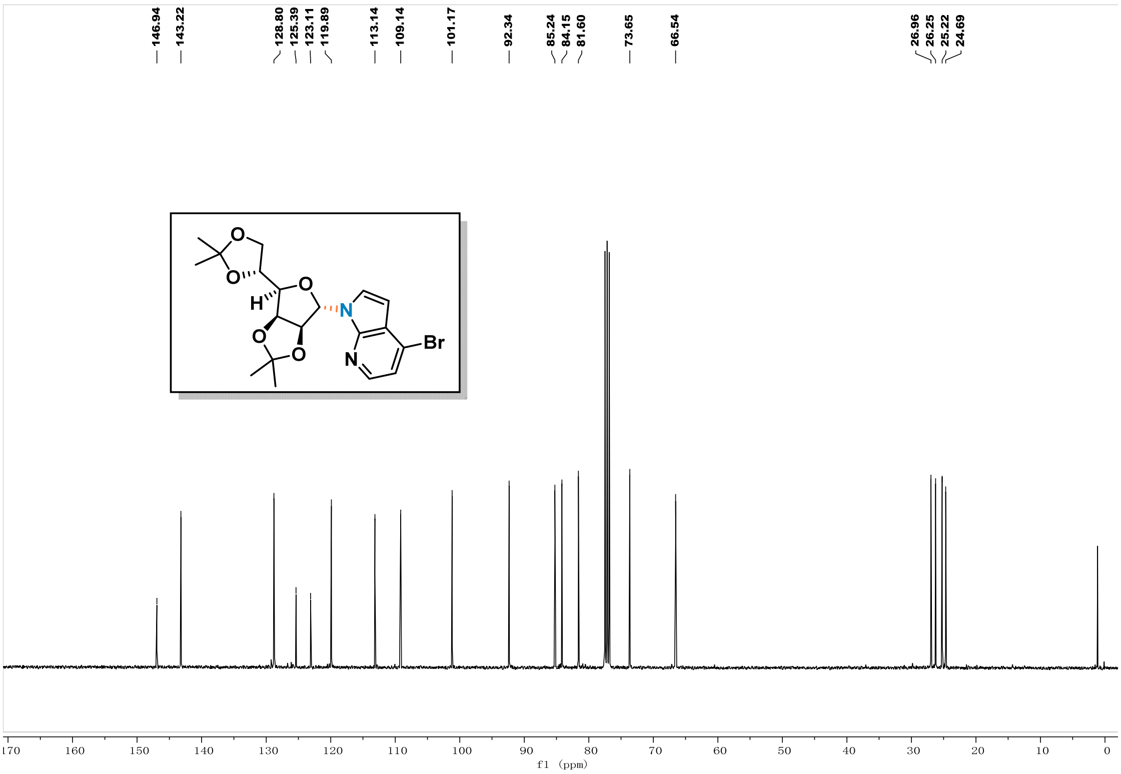

Supplementary Figure 131 | ^{13}C NMR (101 MHz, $CDCl_3$) **(5l)**

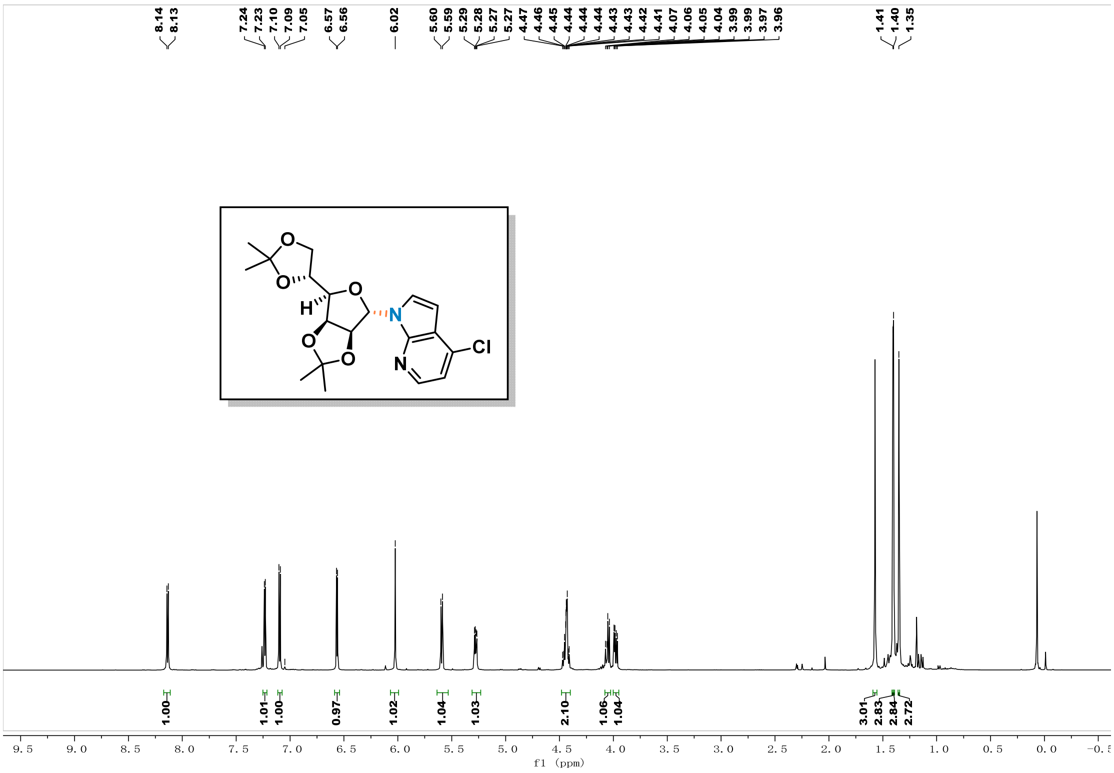

Supplementary Figure 132 | ^{1}H NMR (400 MHz, $CDCl_3$) **(5m)**

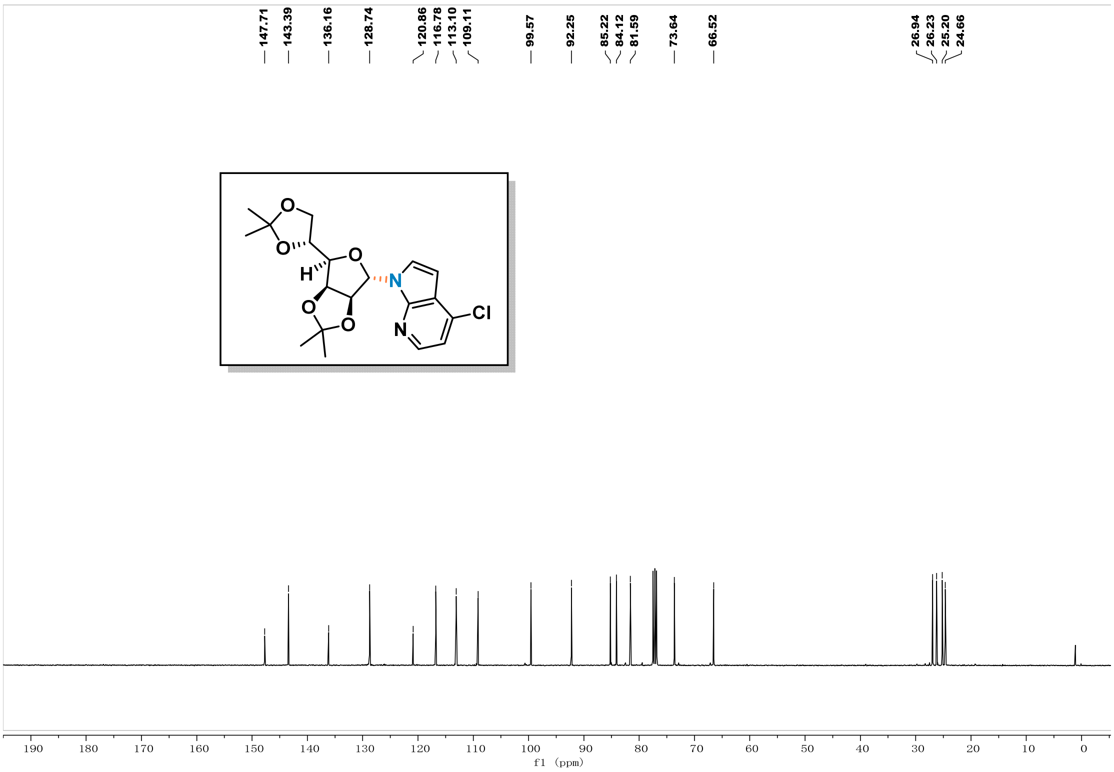

Supplementary Figure 133 | ^{13}C NMR (101 MHz, $CDCl_3$) **(5m)**

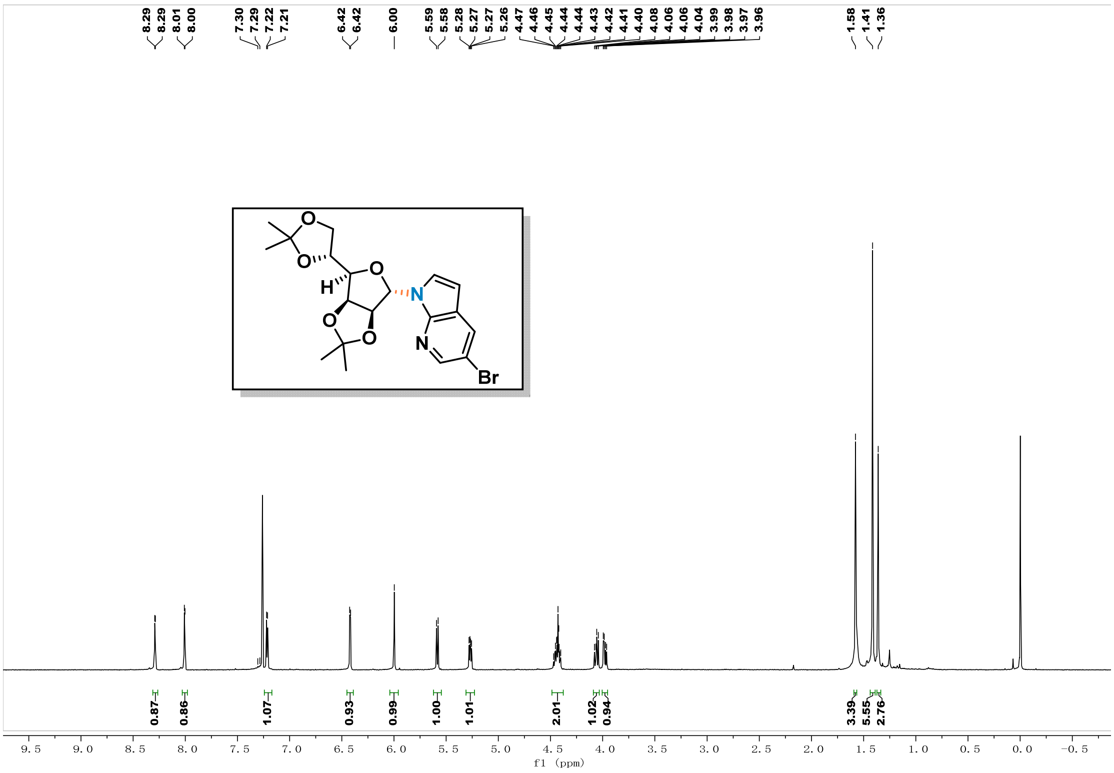

Supplementary Figure 134 | ^{1}H NMR (400 MHz, CDCl$_3$) **(5n)**

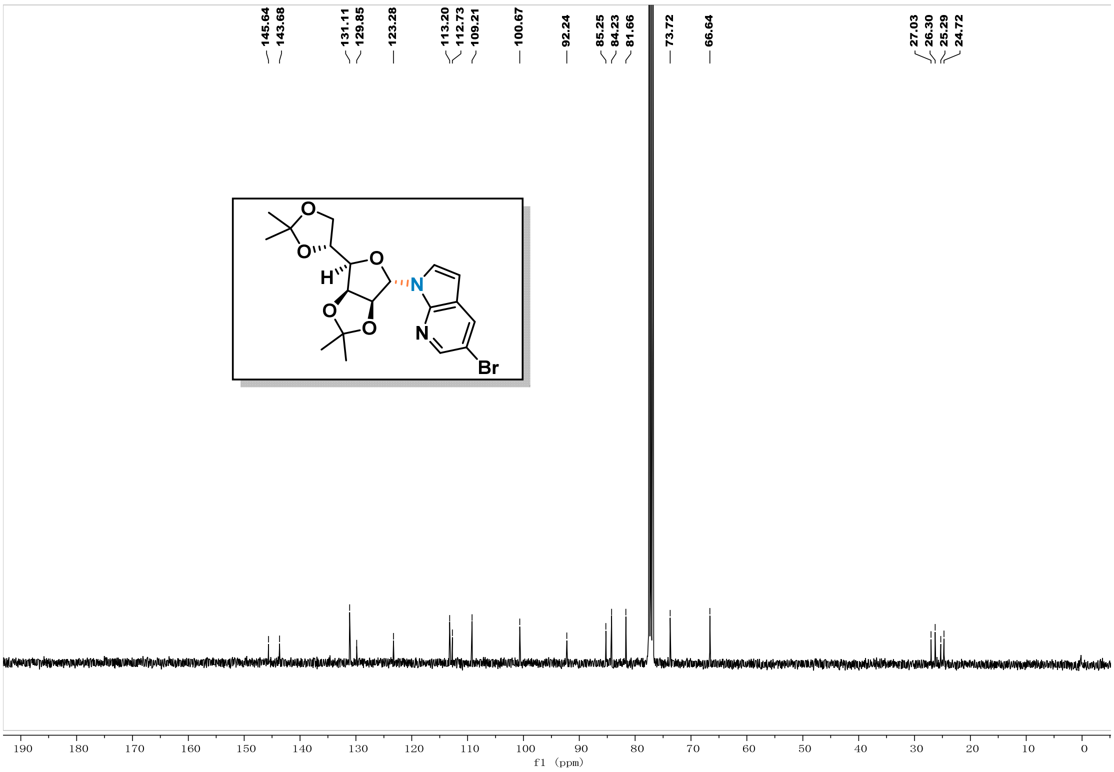

Supplementary Figure 135 | ^{13}C NMR (101 MHz, CDCl$_3$) **(5n)**

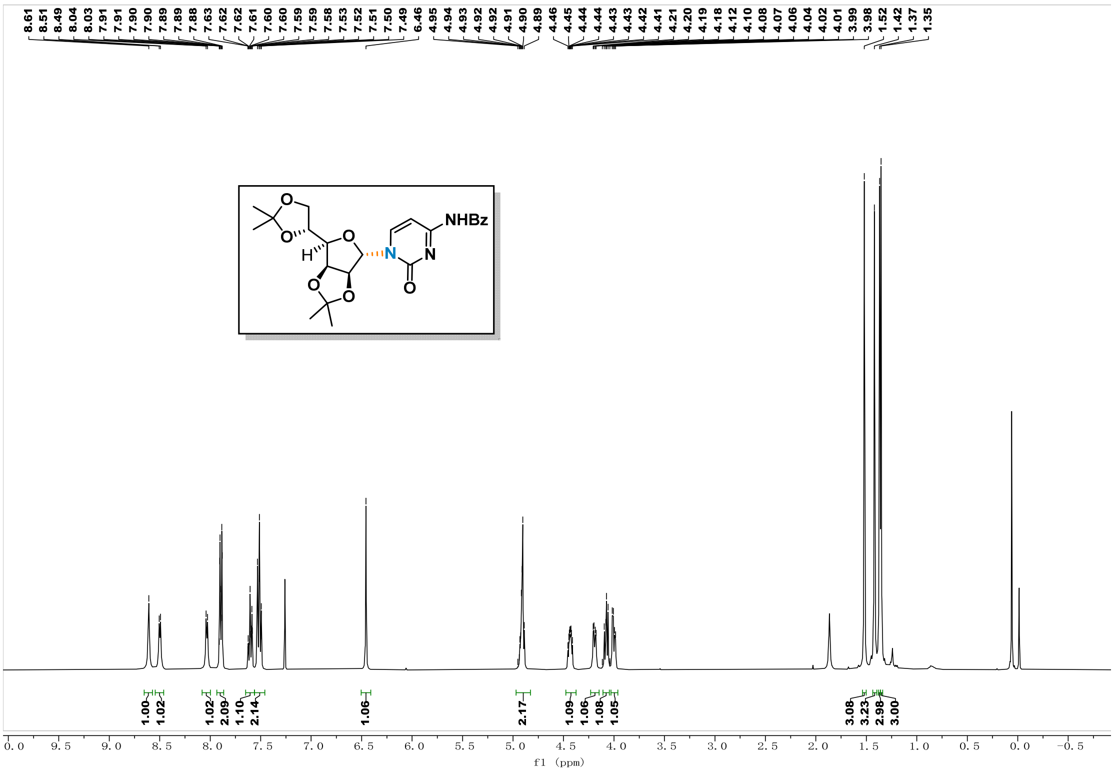

Supplementary Figure 136 | ^{1}H NMR (400 MHz, $CDCl_3$) **(6a)**

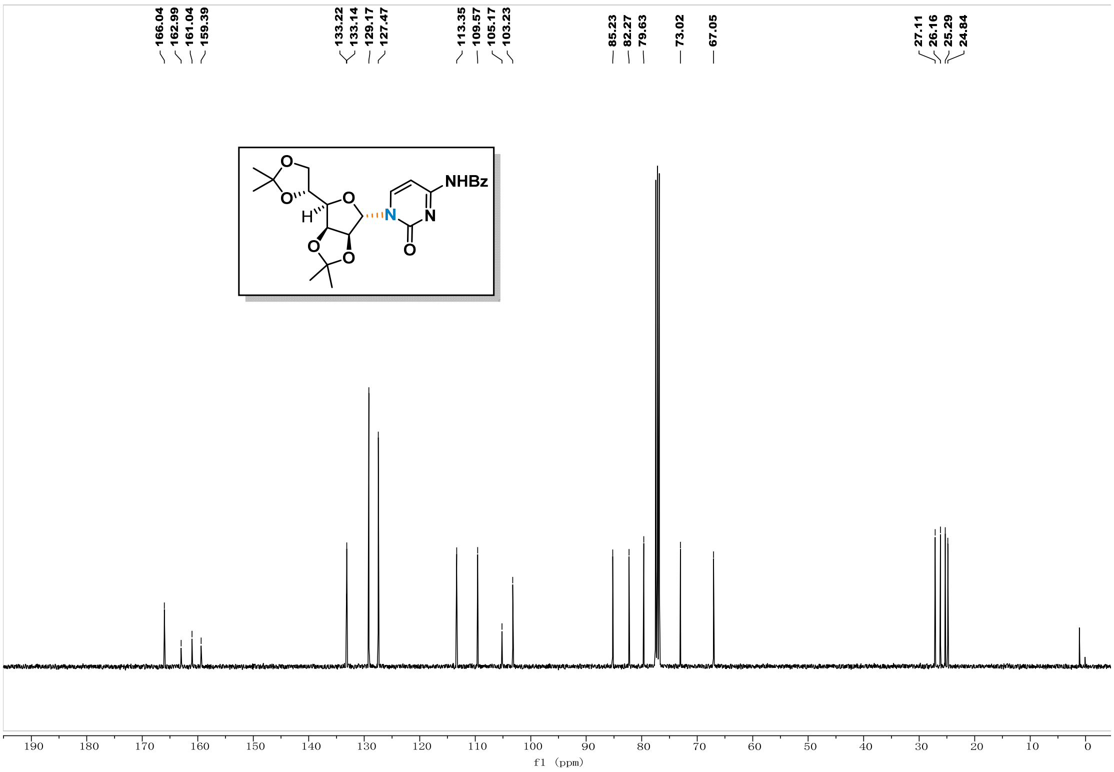

Supplementary Figure 137 | ^{13}C NMR (101 MHz, $CDCl_3$) **(6a)**

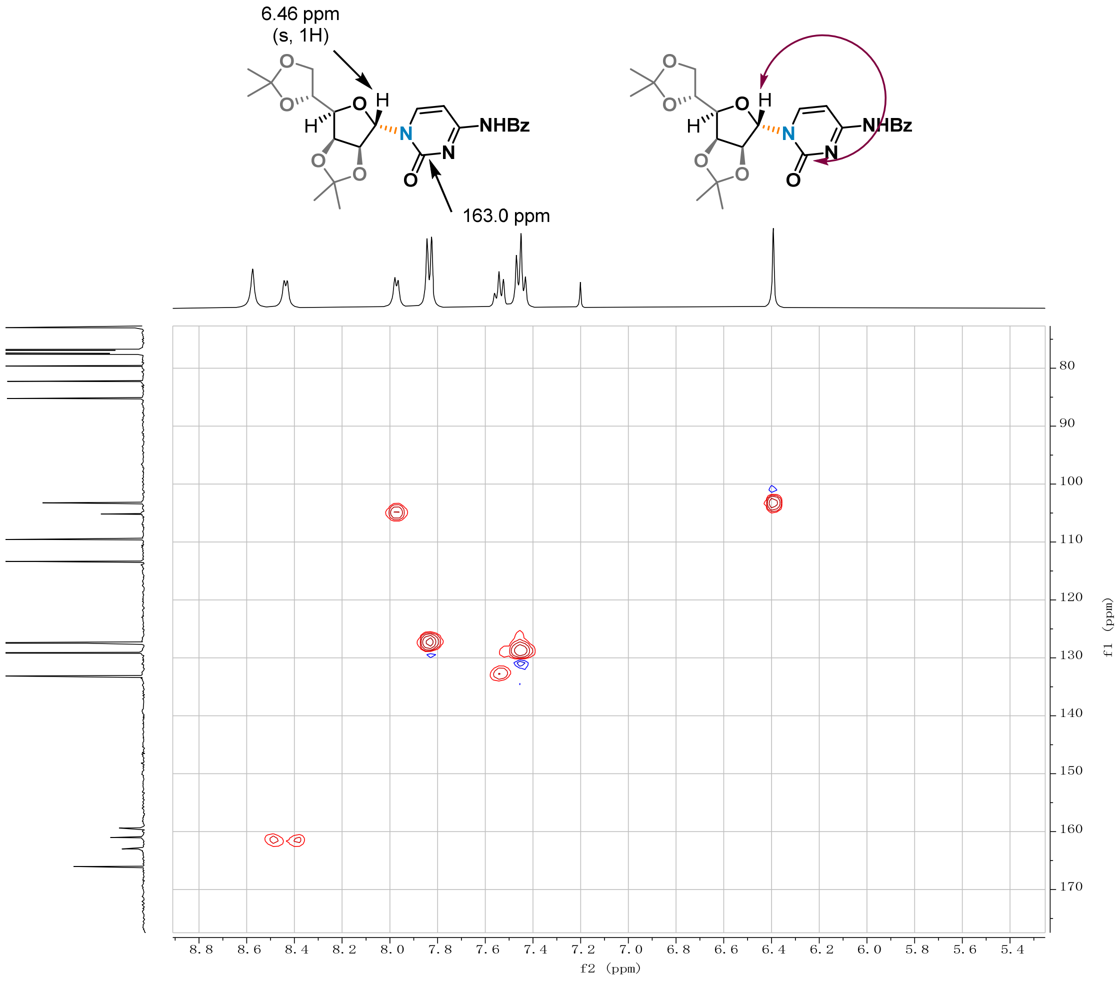

Supplementary Figure 138 | HMBC of **6a**

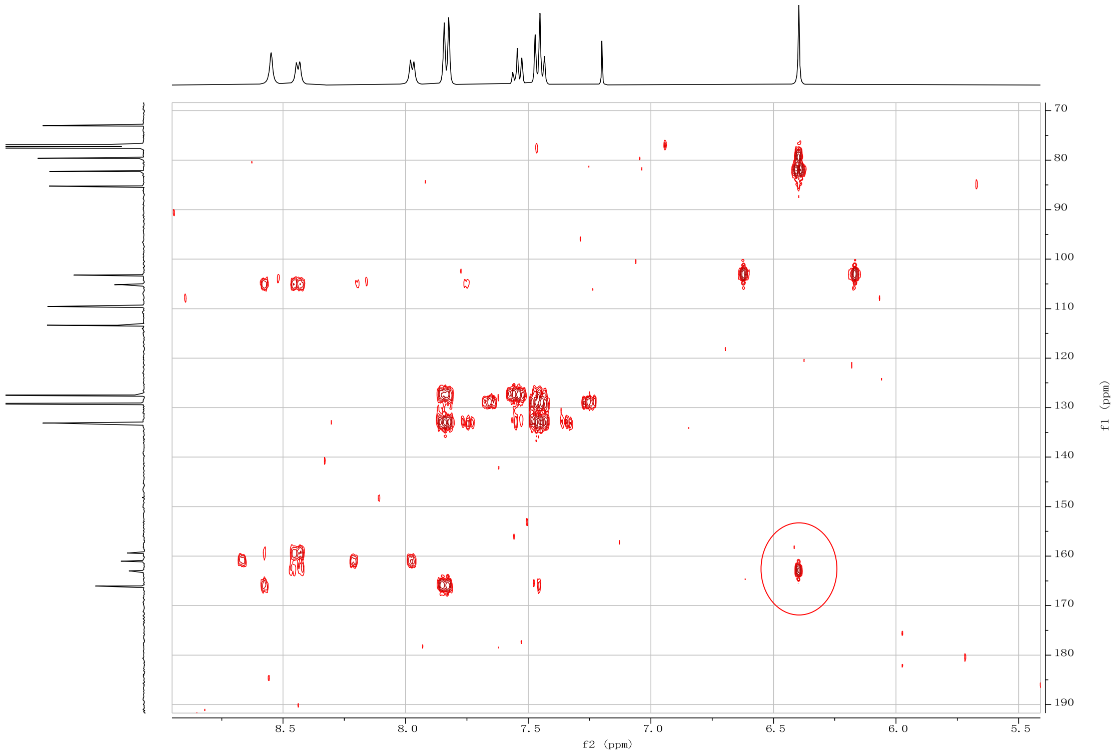

Supplementary Figure 139 | HMBC of **6a**

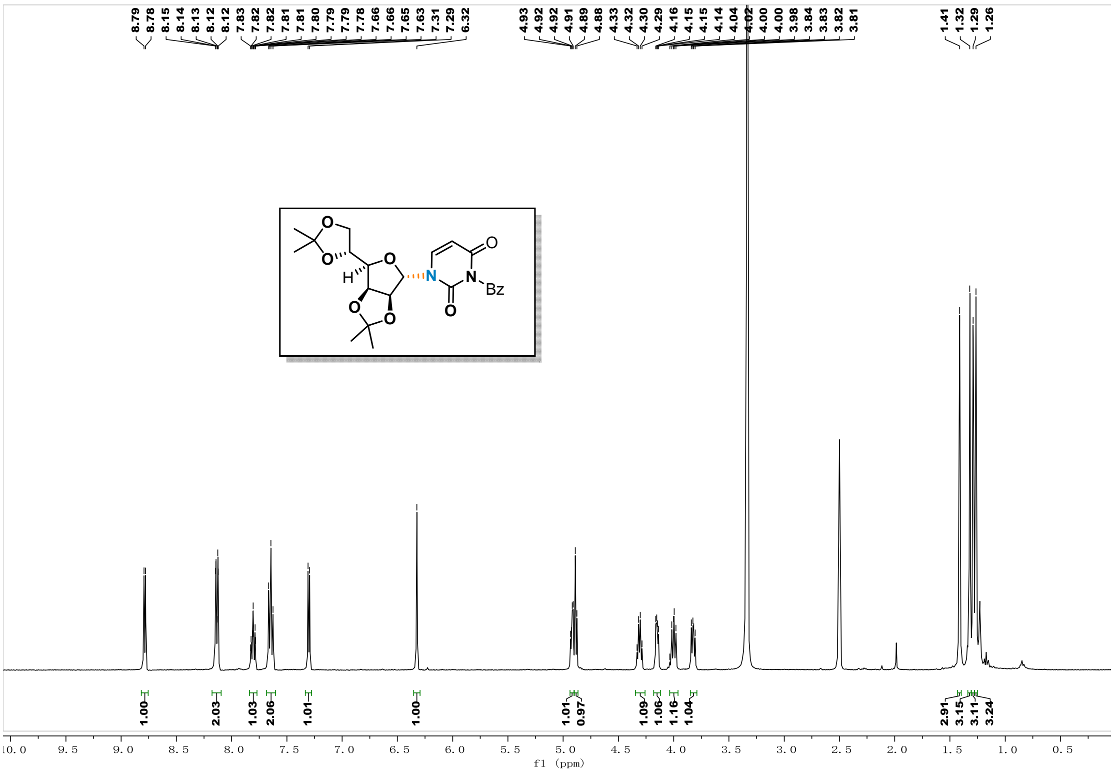

Supplementary Figure 140 | ^{1}H NMR (400 MHz, DMSO-*d*6) **(6b)**

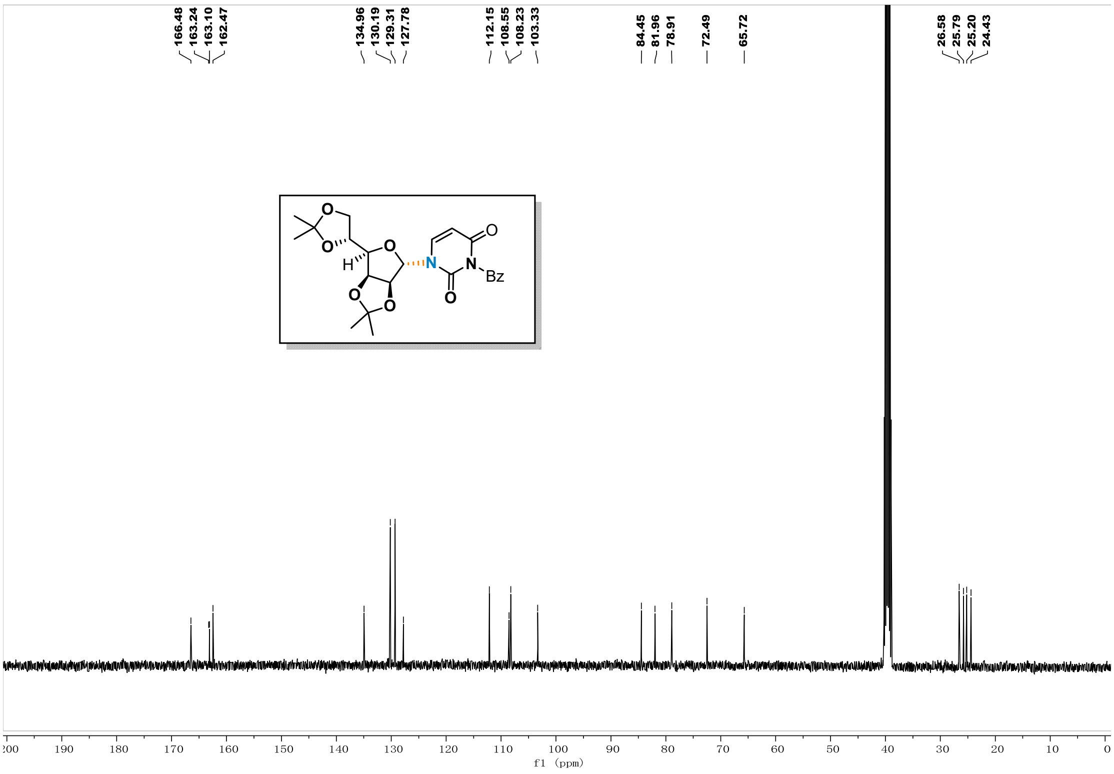

Supplementary Figure 141 | ^{13}C NMR (101 MHz, DMSO-*d*6) **(6b)**

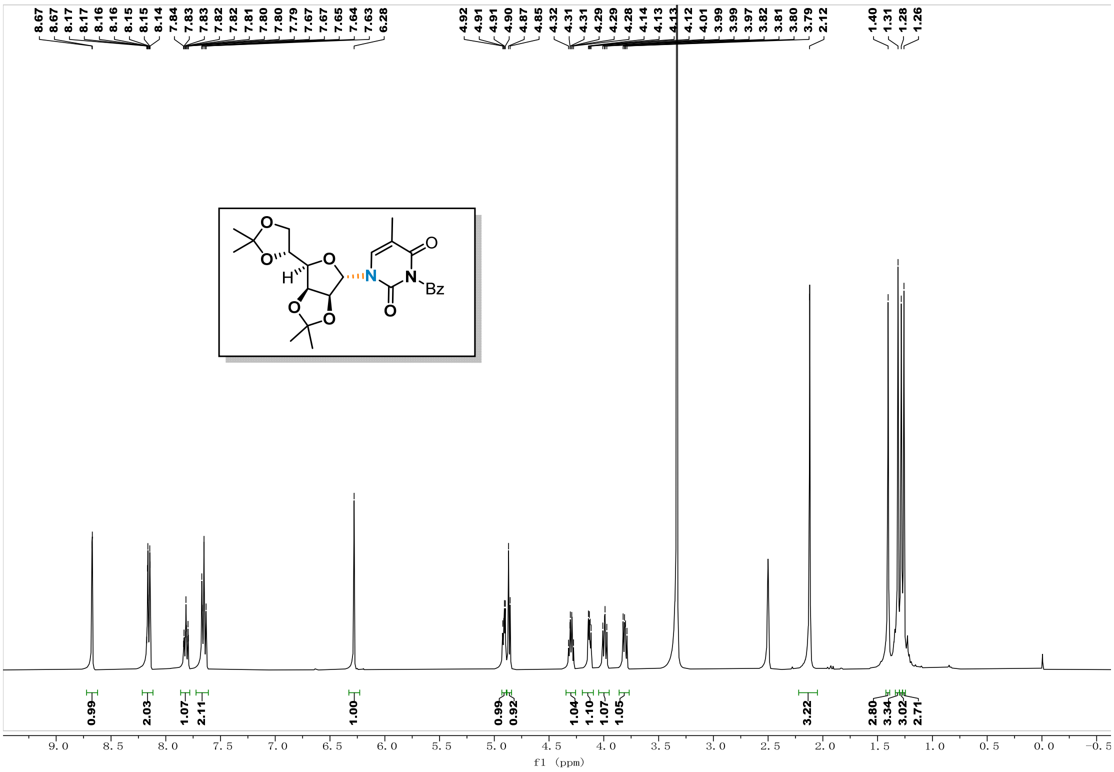

Supplementary Figure 142 | ^{1}H NMR (400 MHz, DMSO-d_6) **(6c)**

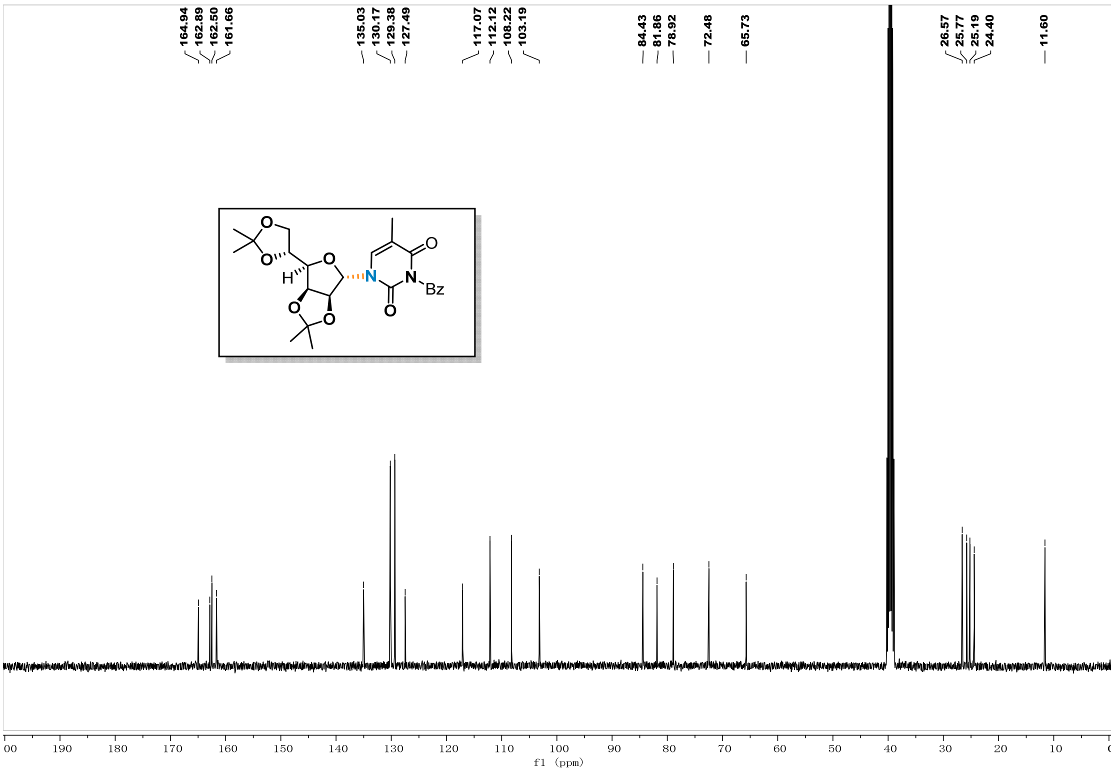

Supplementary Figure 143 | ^{13}C NMR (101 MHz, DMSO-d_6) **(6c)**

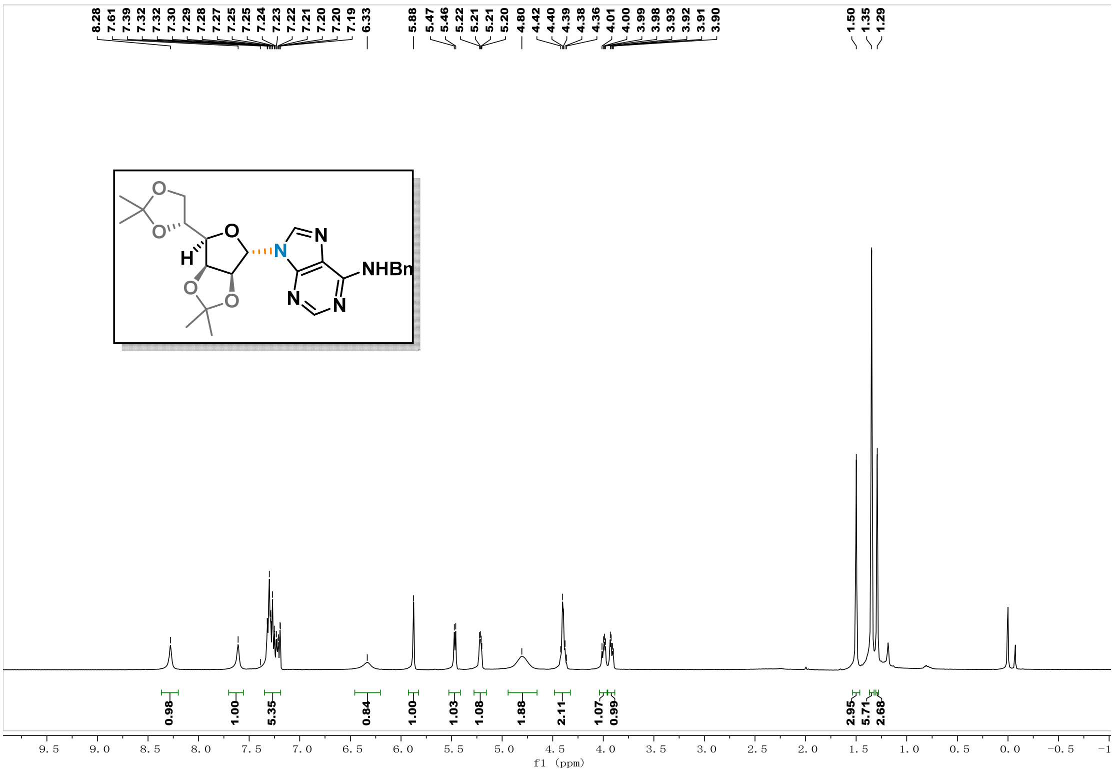

Supplementary Figure 144 | ^{1}H NMR (400 MHz, $CDCl_3$) **(6d)**

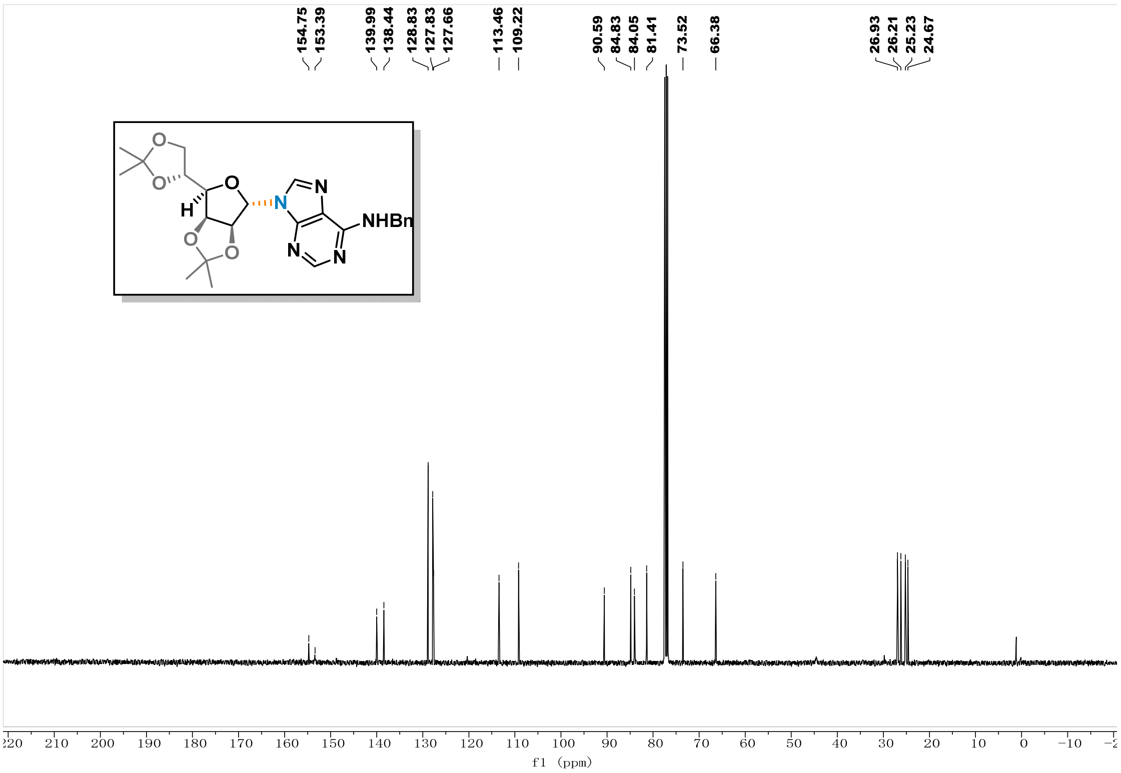

Supplementary Figure 145 | ^{13}C NMR (101 MHz, $CDCl_3$) **(6d)**

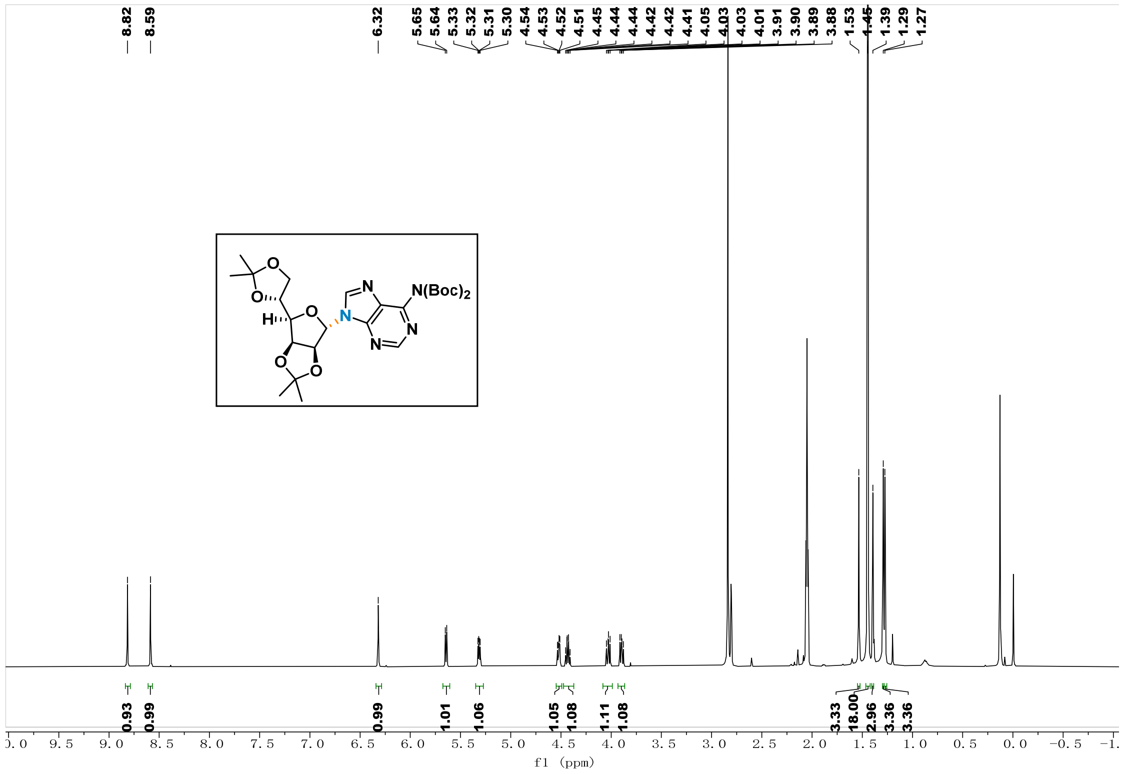

Supplementary Figure 146 | ^{1}H NMR (400 MHz, Acetone-d_6) **(6e)**

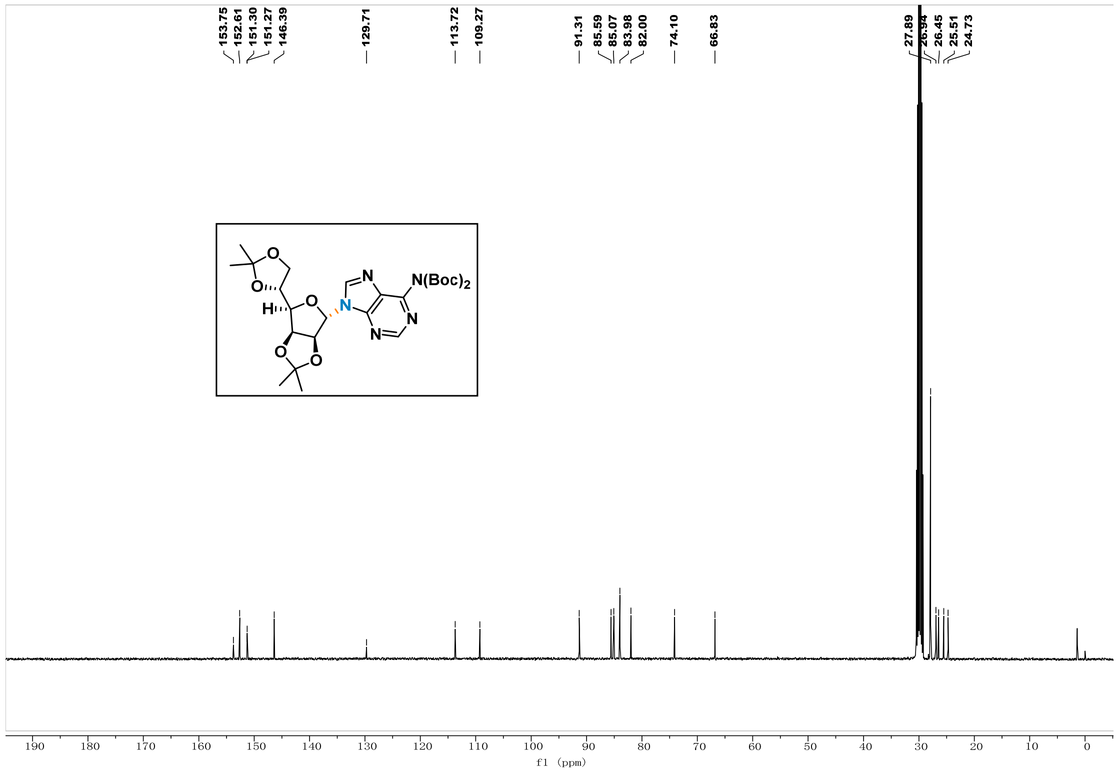

Supplementary Figure 147 | ^{13}C NMR (101 MHz, Acetone-d_6) **(6e)**

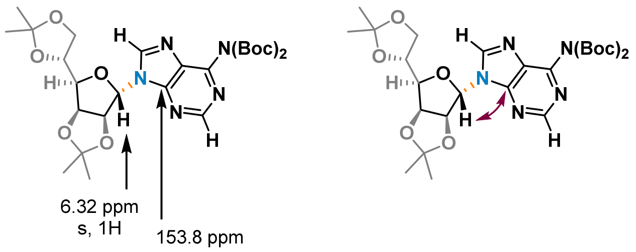

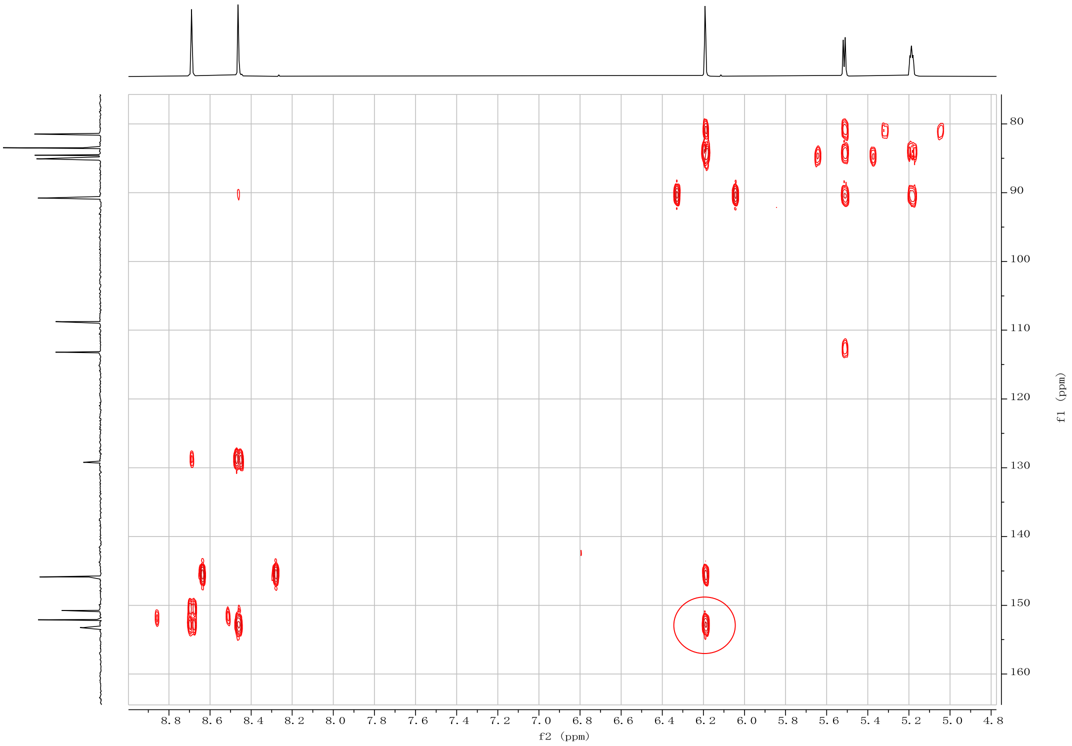

Supplementary Figure 148 | HMBC of **6e**

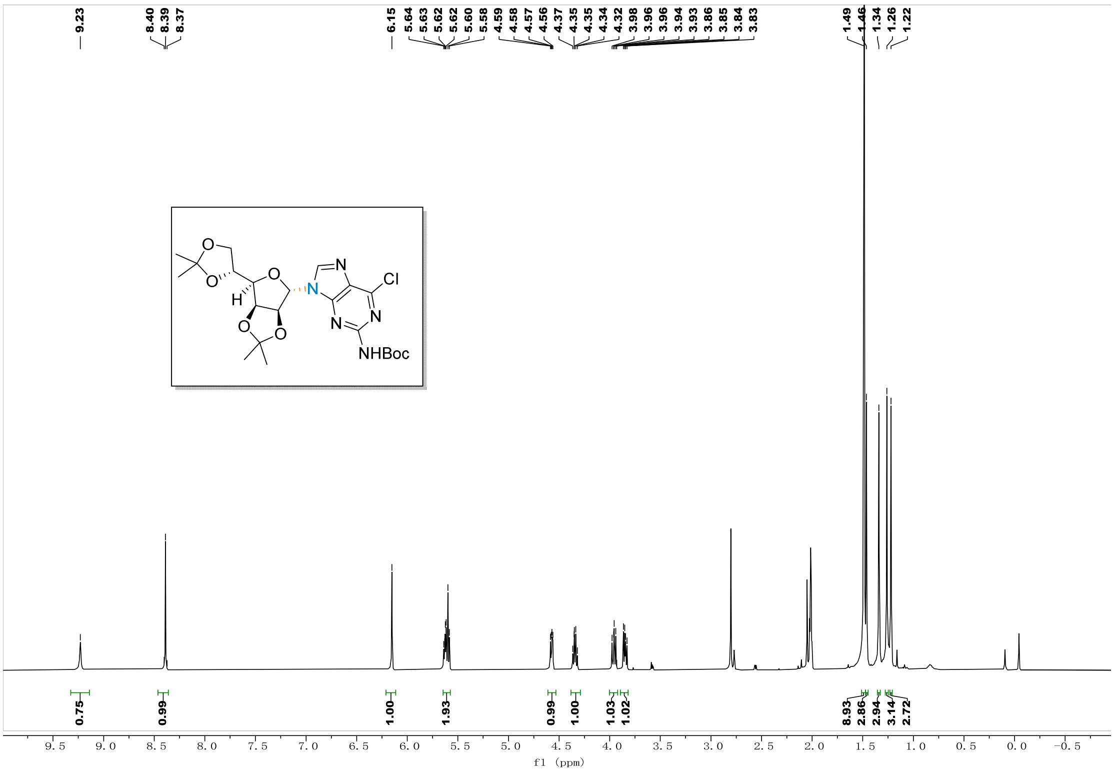

Supplementary Figure 149 | ^{1}H NMR (400 MHz, Acetone-d_6) **(6f)**

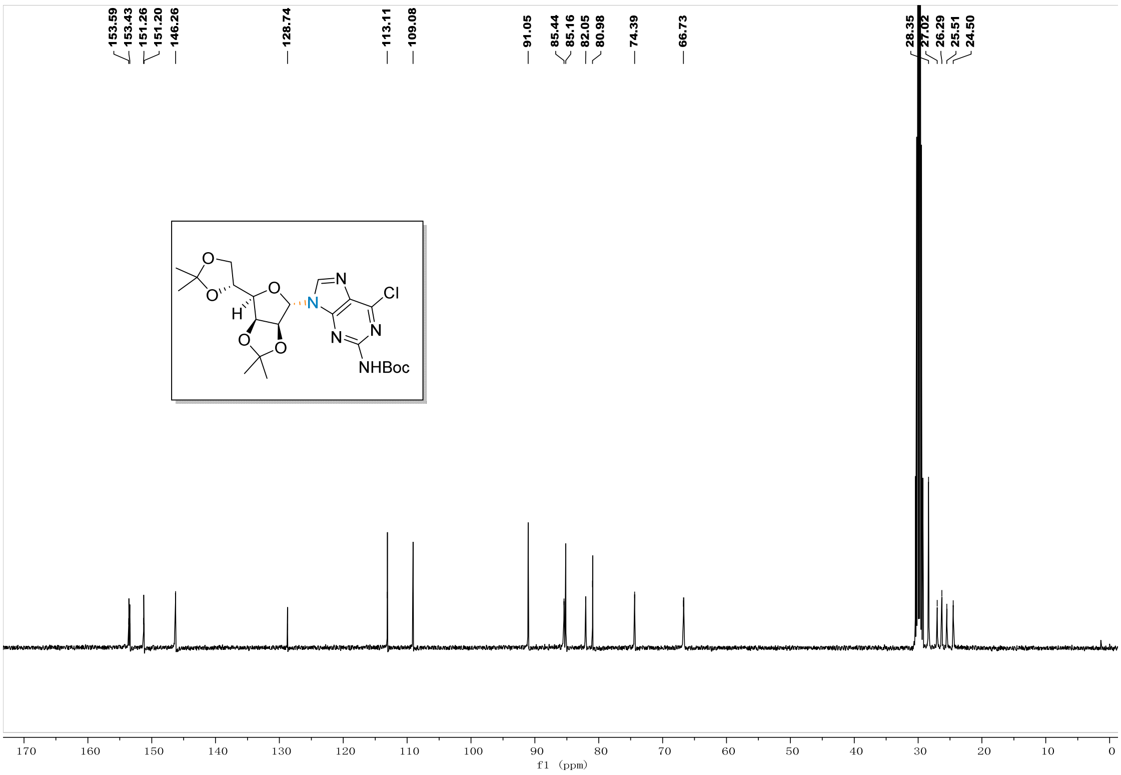

Supplementary Figure 150 | ^{13}C NMR (101 MHz, Acetone-d_6) **(6f)**

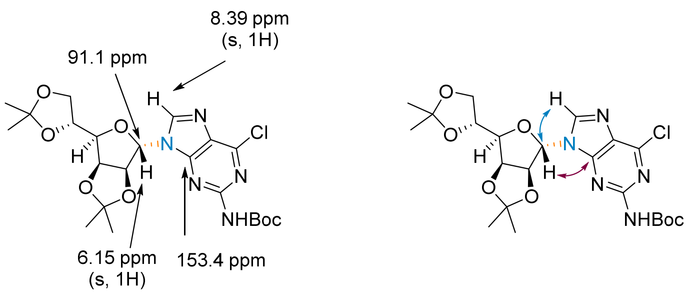

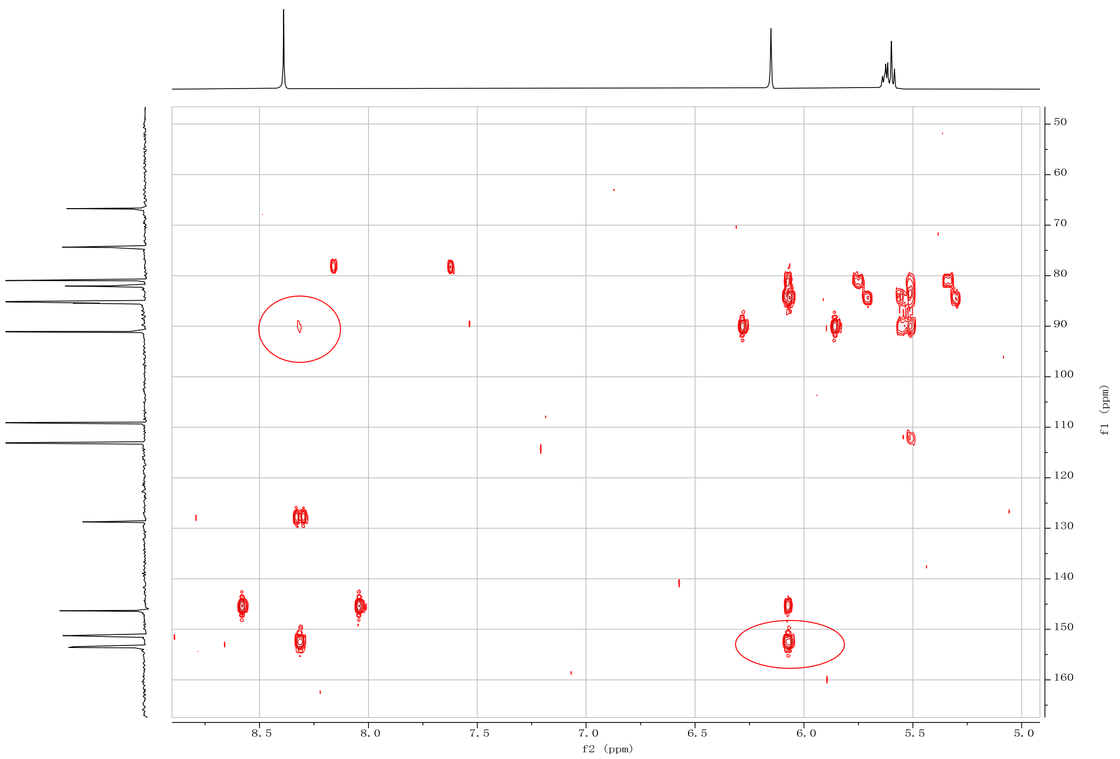

Supplementary Figure 151 | HMBC of **6f**

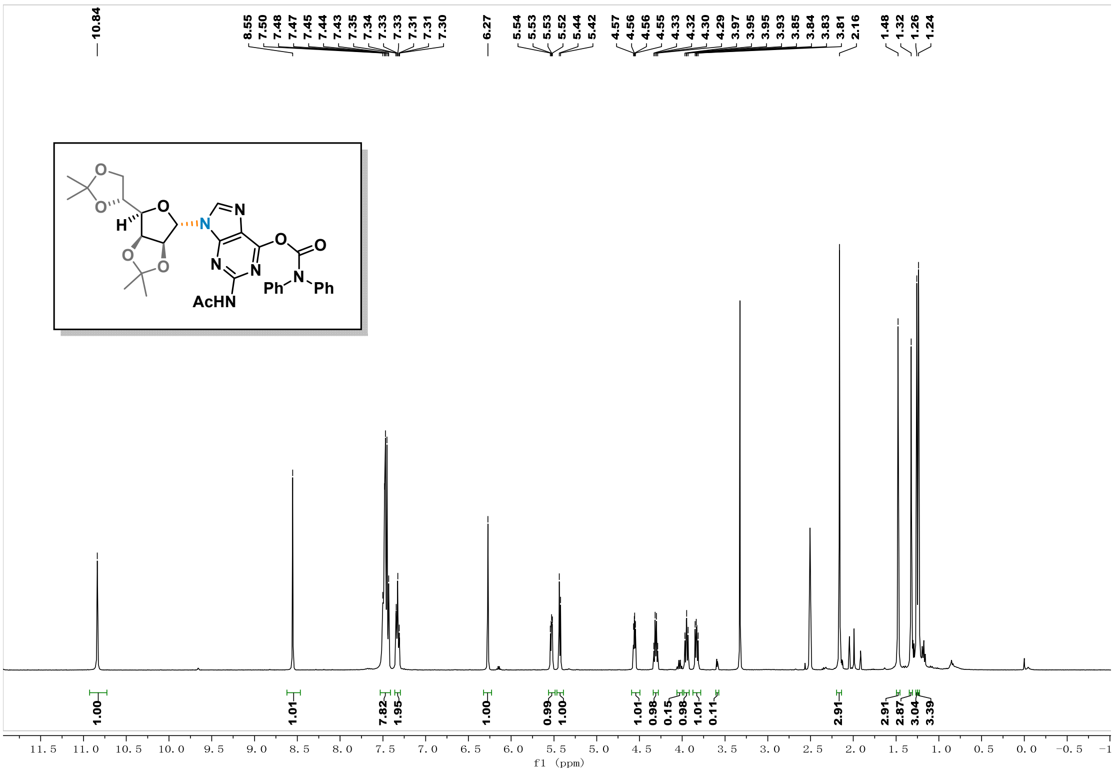

Supplementary Figure 152 | ^{1}H NMR (400 MHz, DMSO-d_6) **(6g)**

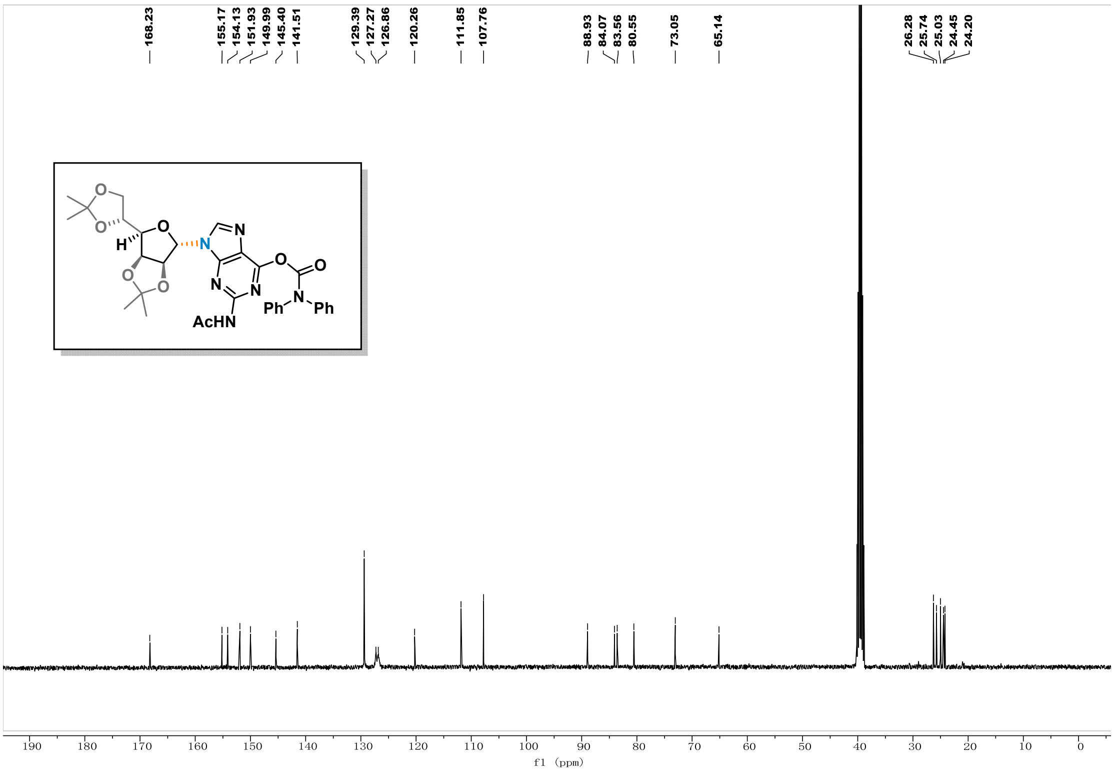

Supplementary Figure 153 | ^{13}C NMR (101 MHz, DMSO-d_6) **(6g)**

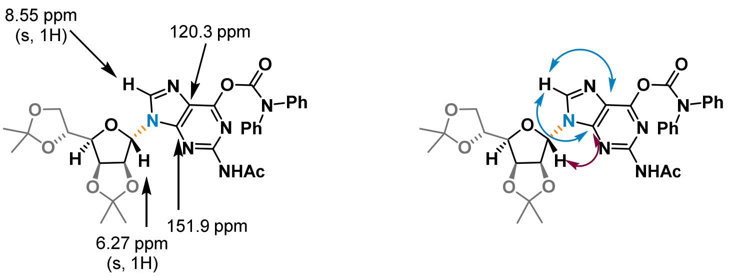

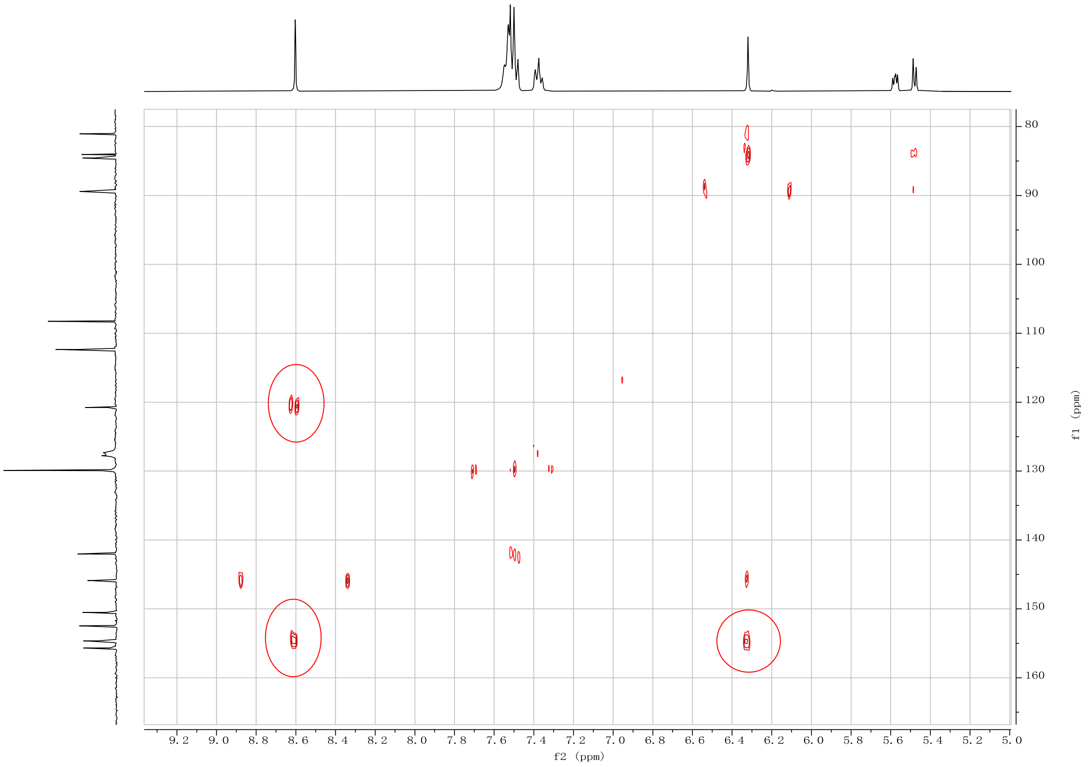

Supplementary Figure 154 | HMBC of **6g**

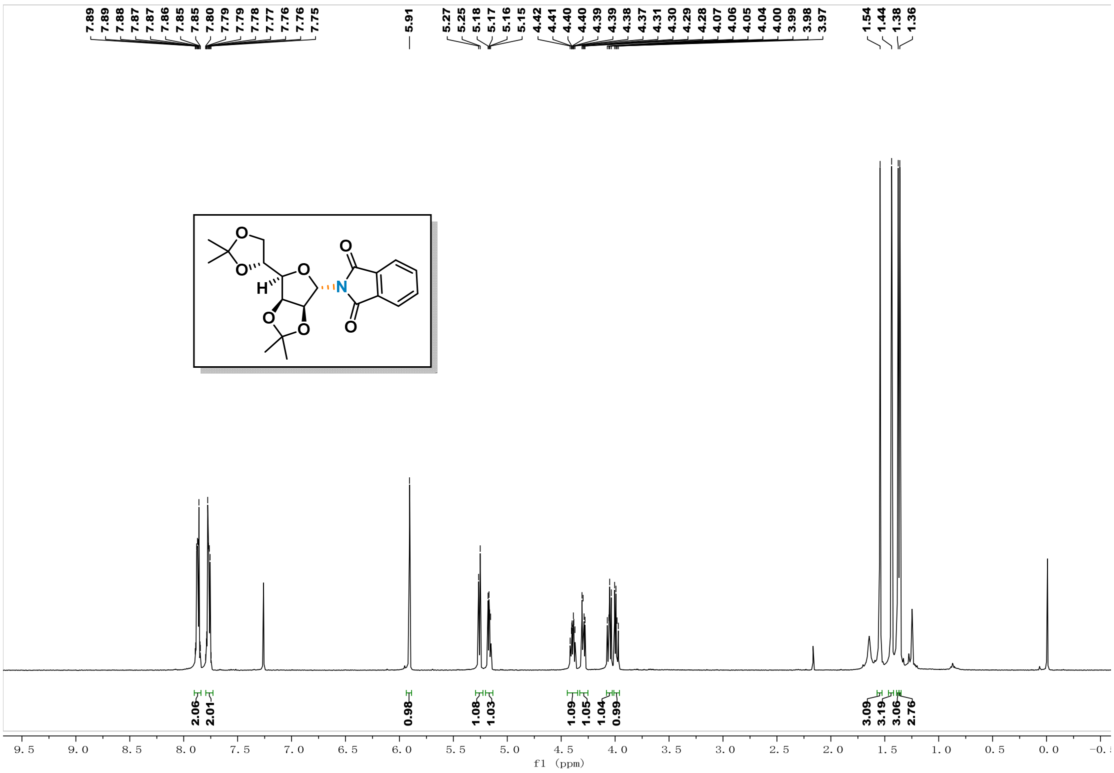

Supplementary Figure 155 | ^{1}H NMR (400 MHz, CDCl$_3$) **(7a)**

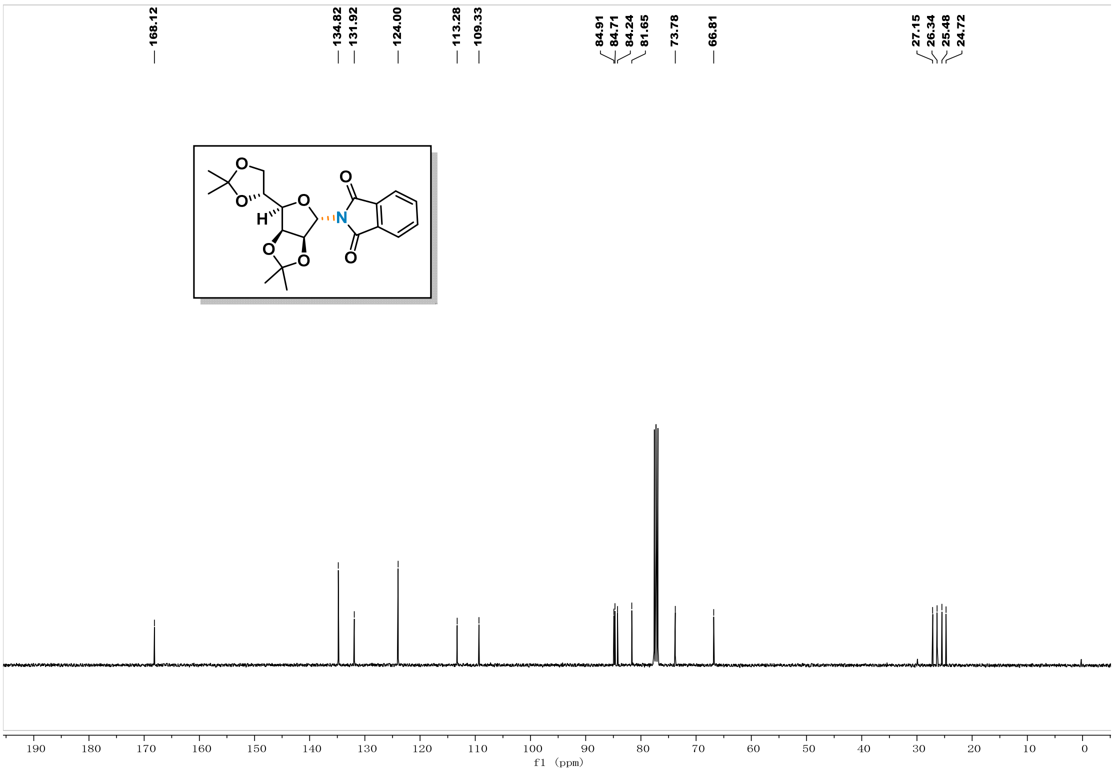

Supplementary Figure 156 | ^{13}C NMR (101 MHz, CDCl$_3$) **(7a)**

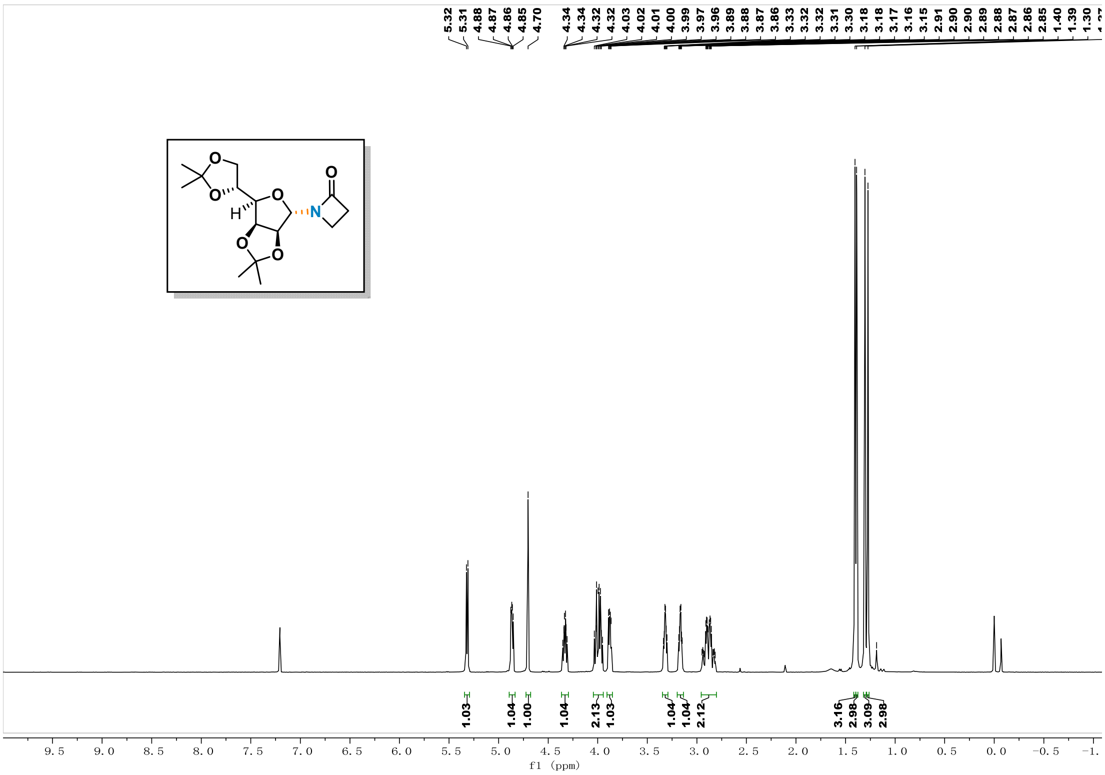

Supplementary Figure 157 | ^{1}H NMR (400 MHz, $CDCl_3$) **(7b)**

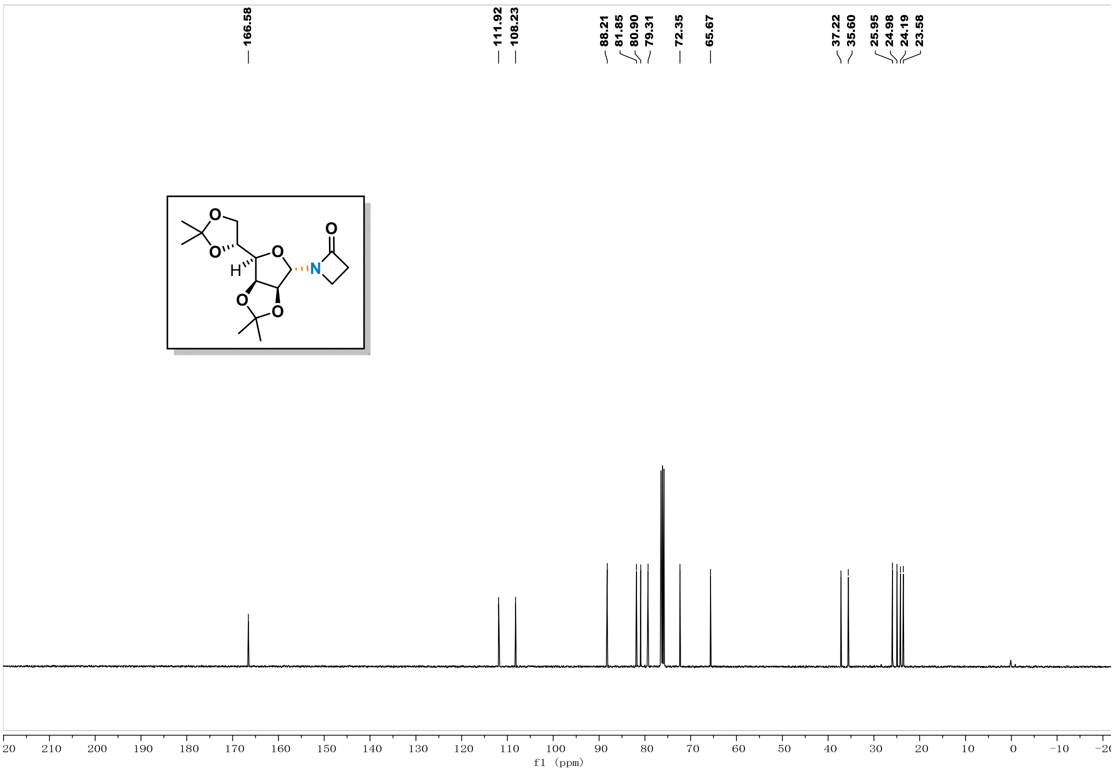

Supplementary Figure 158 | ^{13}C NMR (101 MHz, $CDCl_3$) **(7b)**

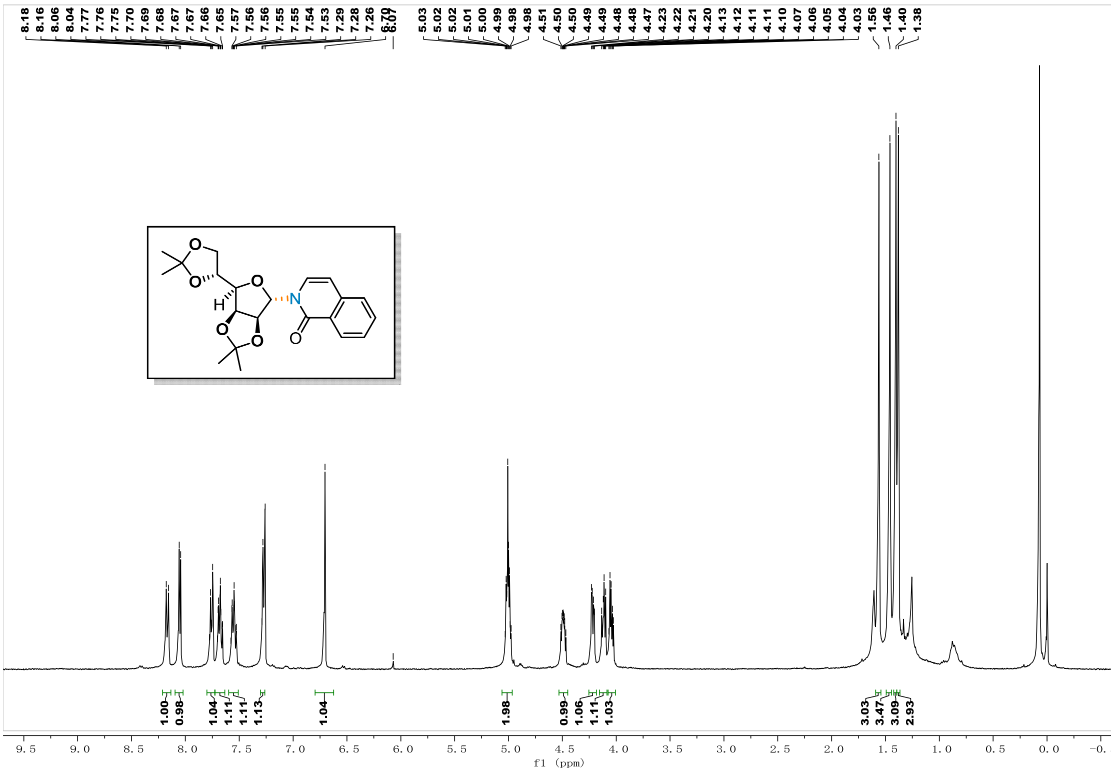

Supplementary Figure 159 | ^{1}H NMR (400 MHz, $CDCl_3$) **(7c)**

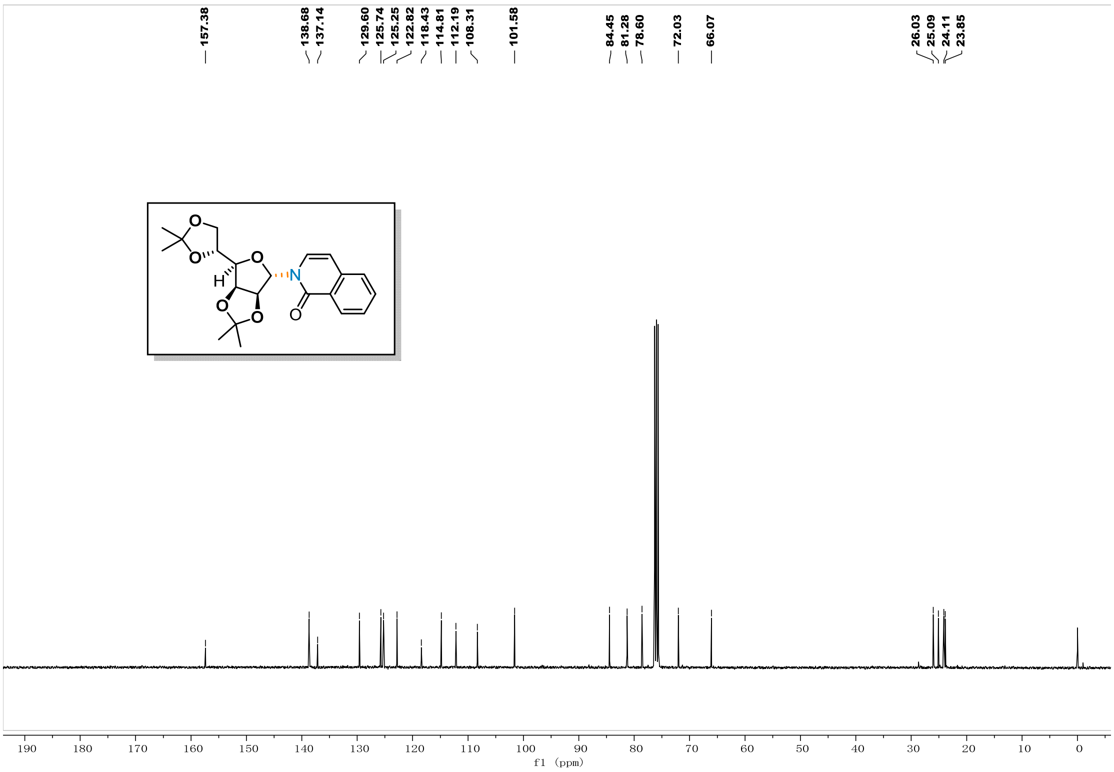

Supplementary Figure 160 | ^{13}C NMR (101 MHz, $CDCl_3$) **(7c)**

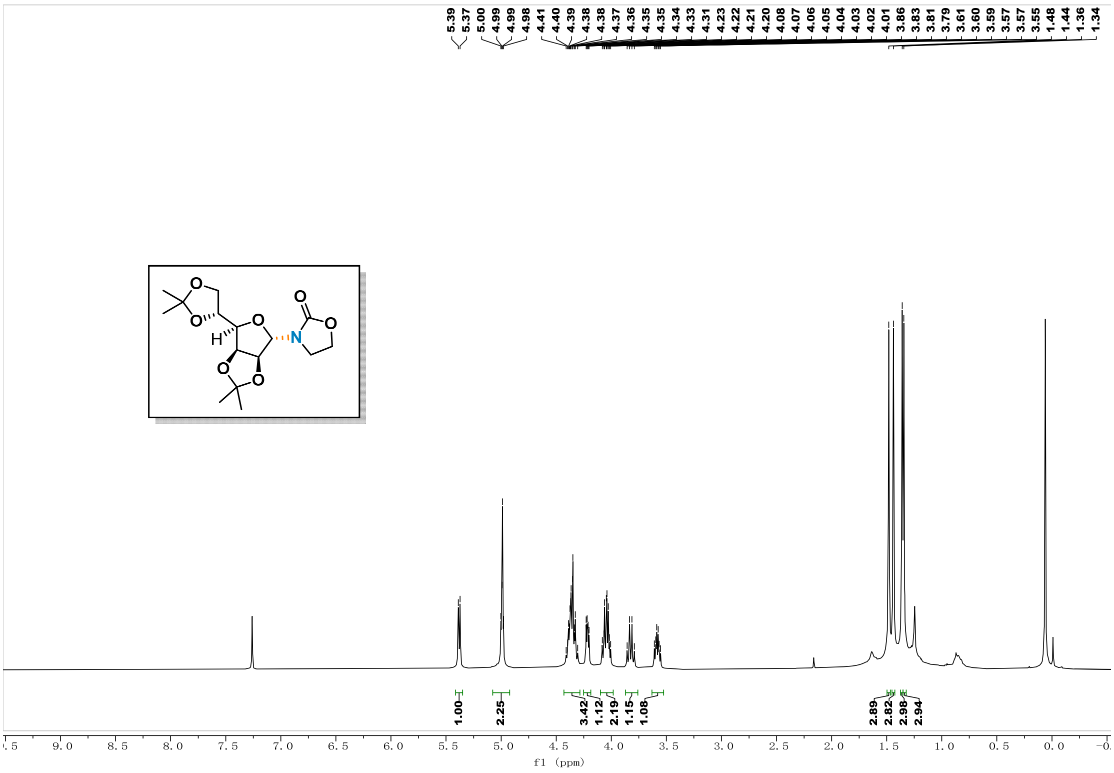

Supplementary Figure 161 | ^{1}H NMR (400 MHz, $CDCl_3$) **(7d)**

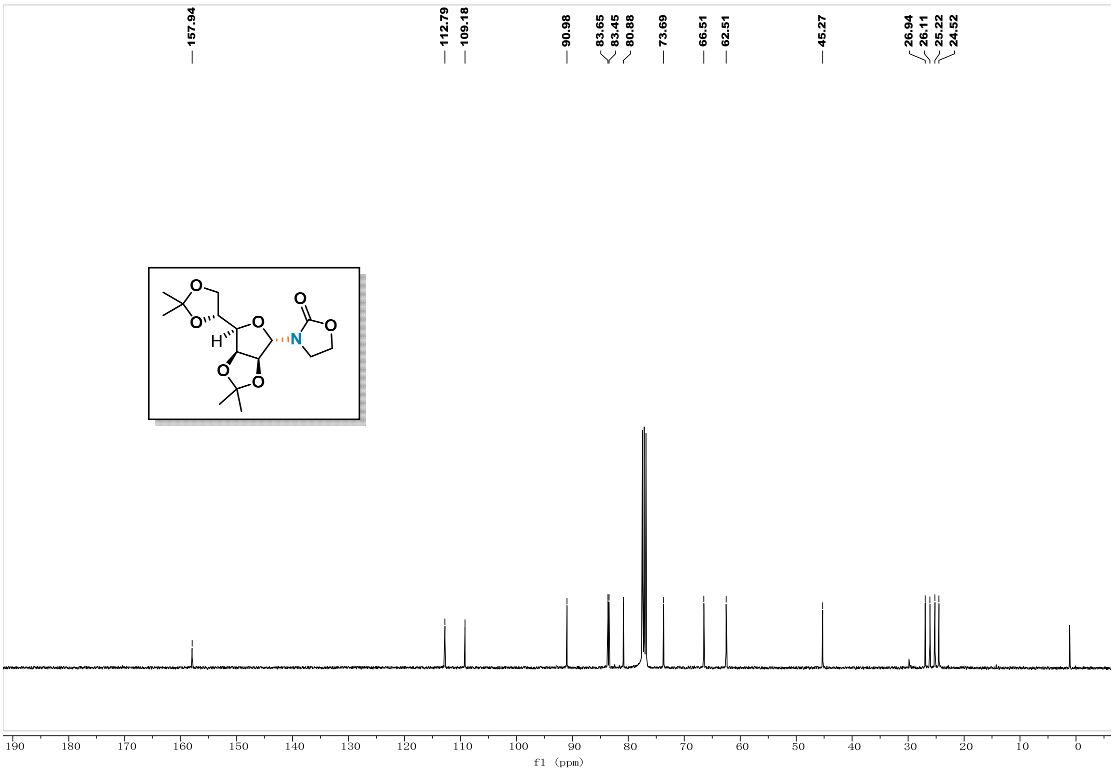

Supplementary Figure 162 | ^{13}C NMR (101 MHz, $CDCl_3$) **(7d)**

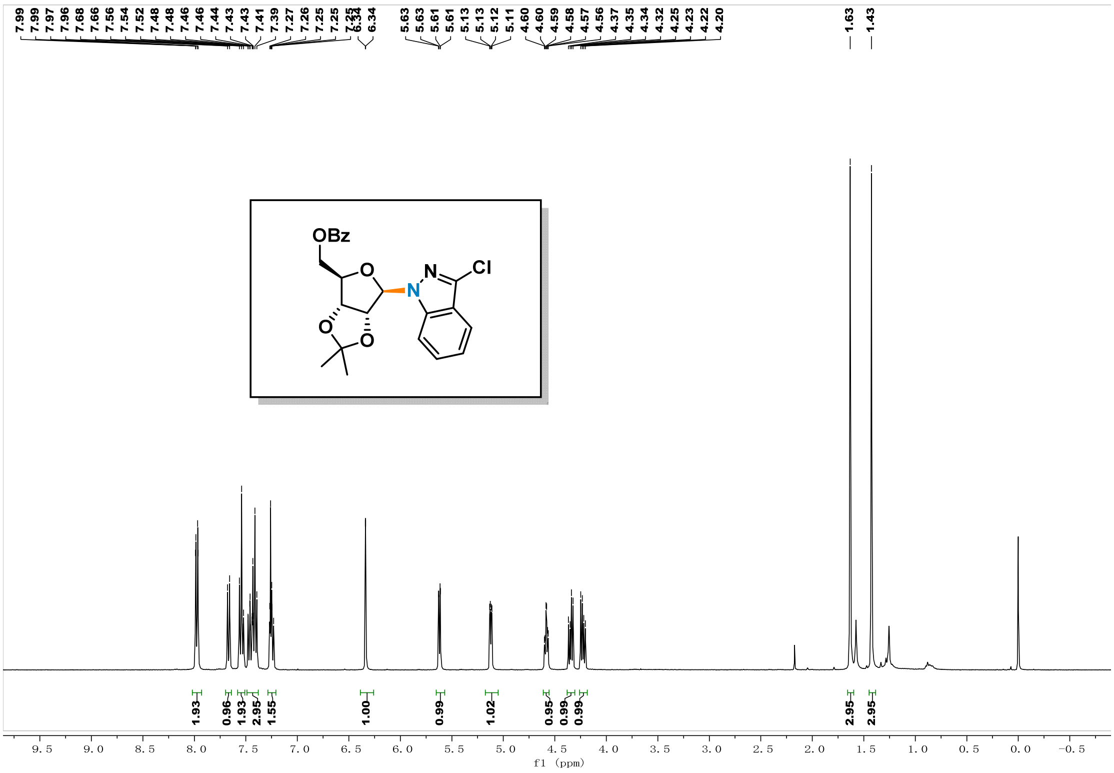

Supplementary Figure 163 | ^{1}H NMR (400 MHz, $CDCl_3$) **(8a)**

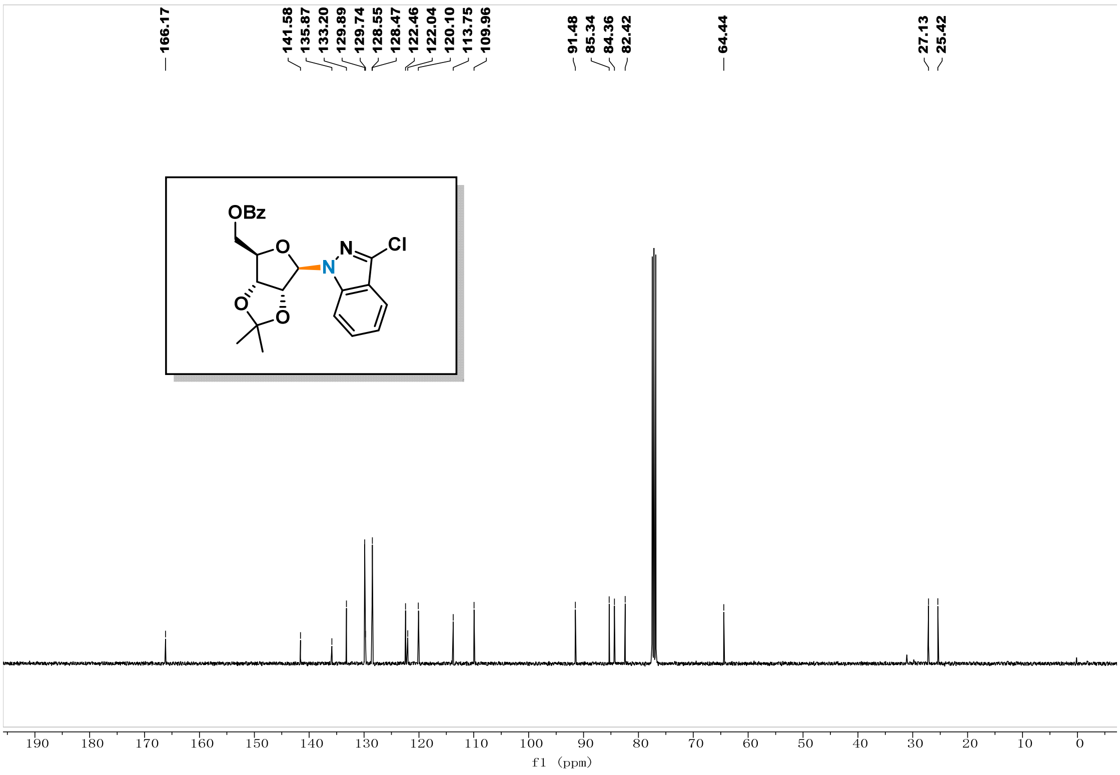

Supplementary Figure 164 | ^{13}C NMR (101 MHz, $CDCl_3$) **(8a)**

NOESY of **8a**

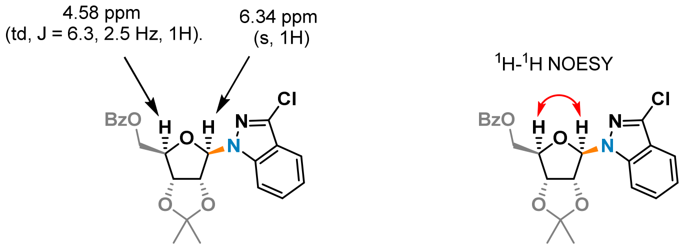

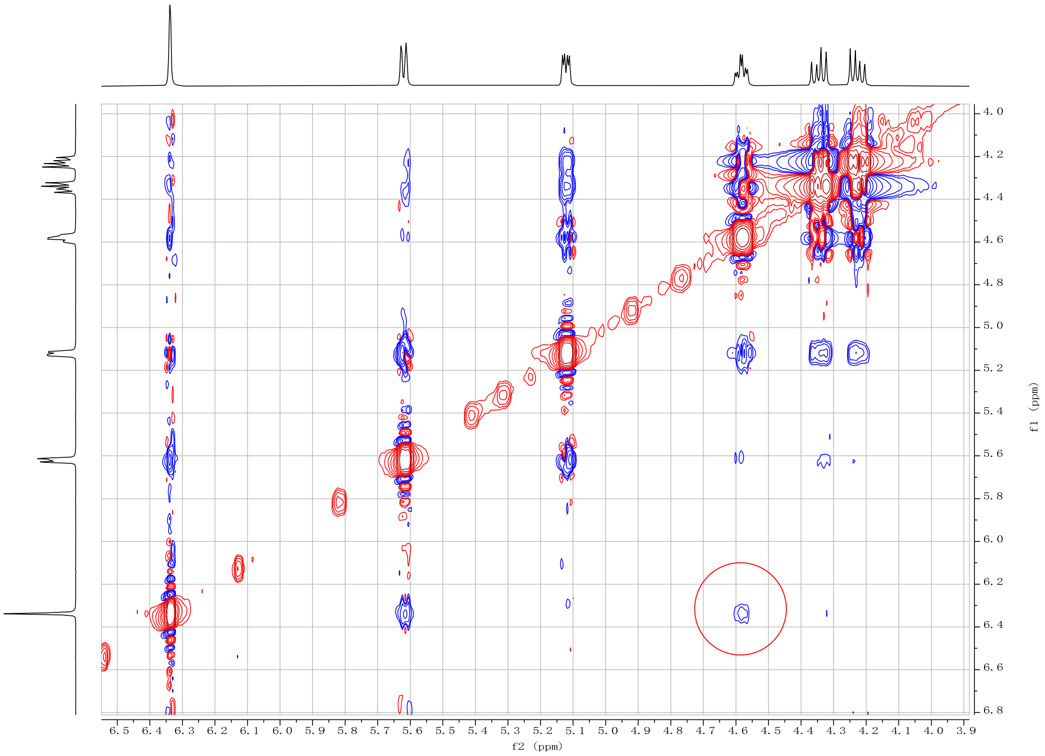

Supplementary Figure 165 | NOESY of **8a**

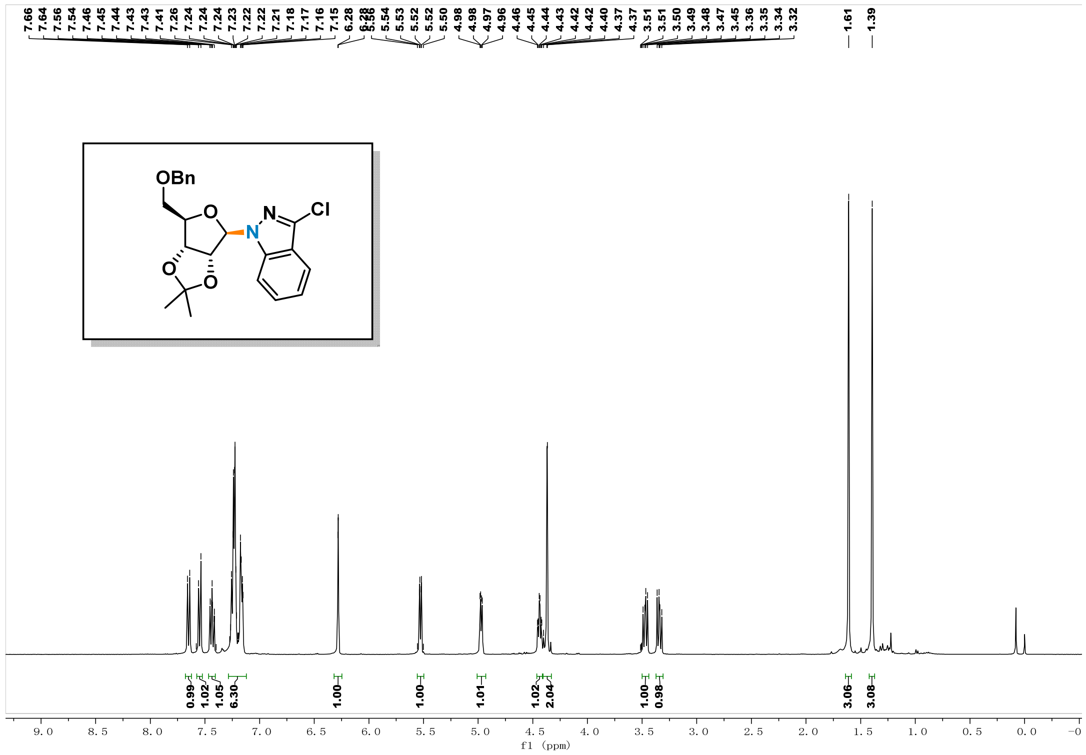

Supplementary Figure 166 | ^{1}H NMR (400 MHz, $CDCl_3$) **(8b)**

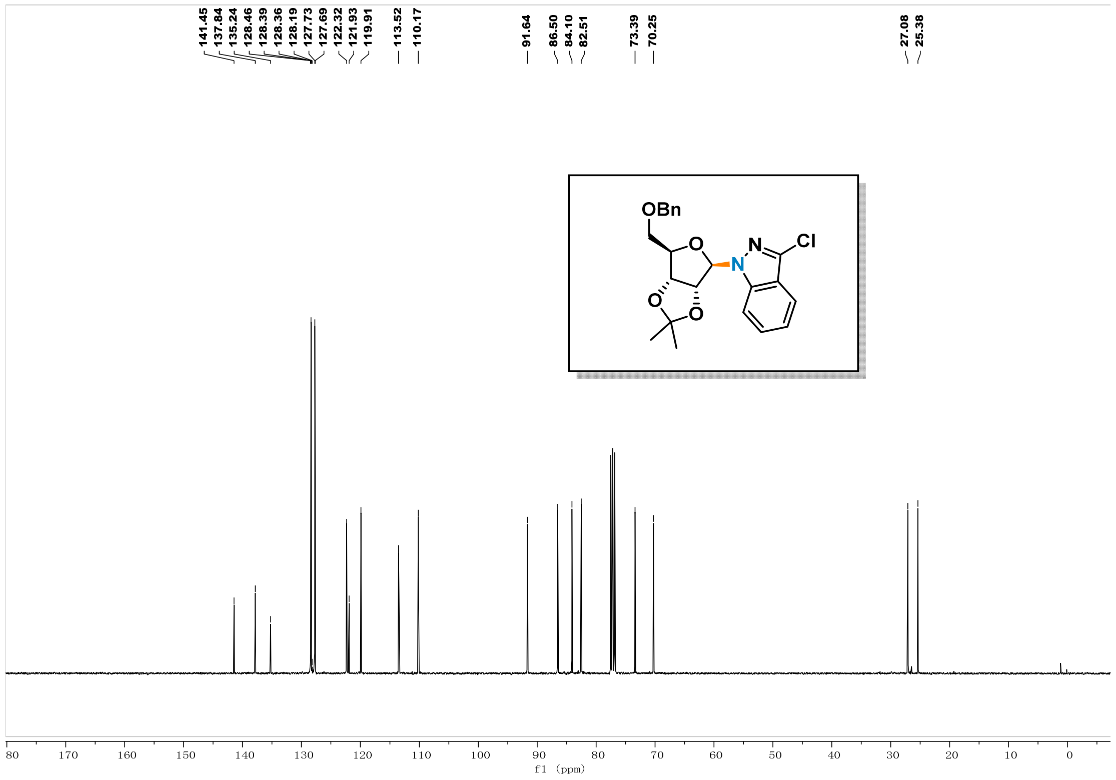

Supplementary Figure 167 | ^{13}C NMR (101 MHz, $CDCl_3$) **(8b)**

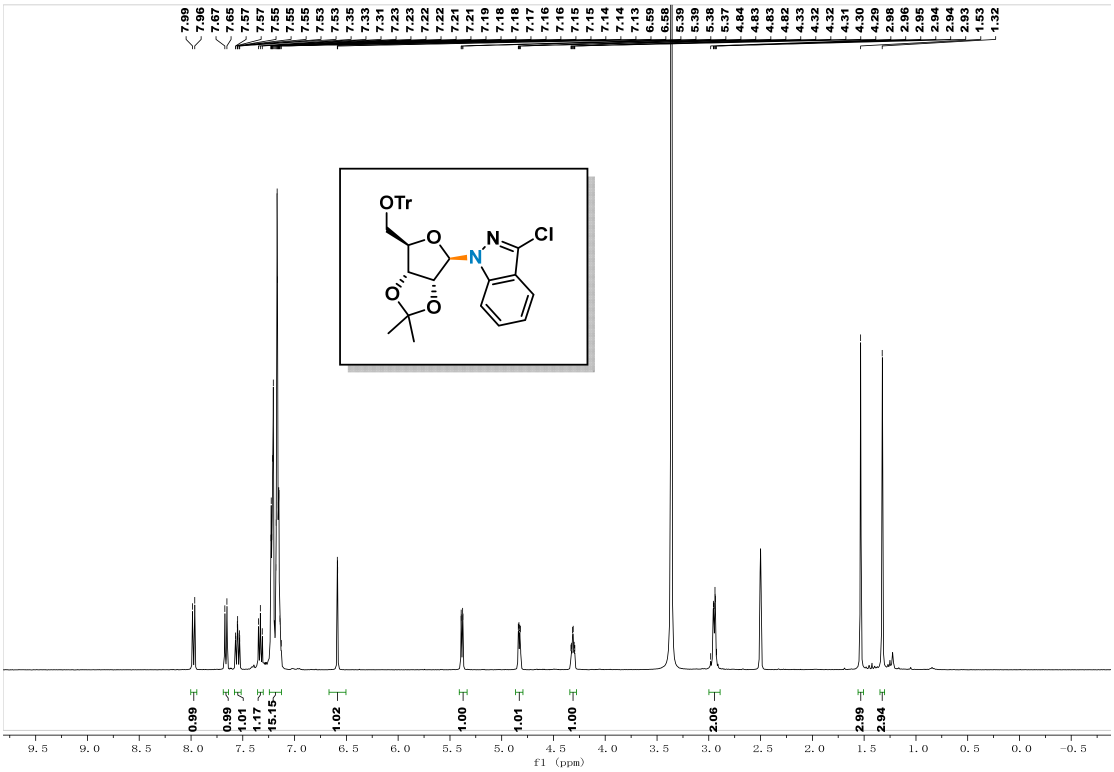

Supplementary Figure 168 | ^{1}H NMR (400 MHz, $CDCl_3$) **(8c)**

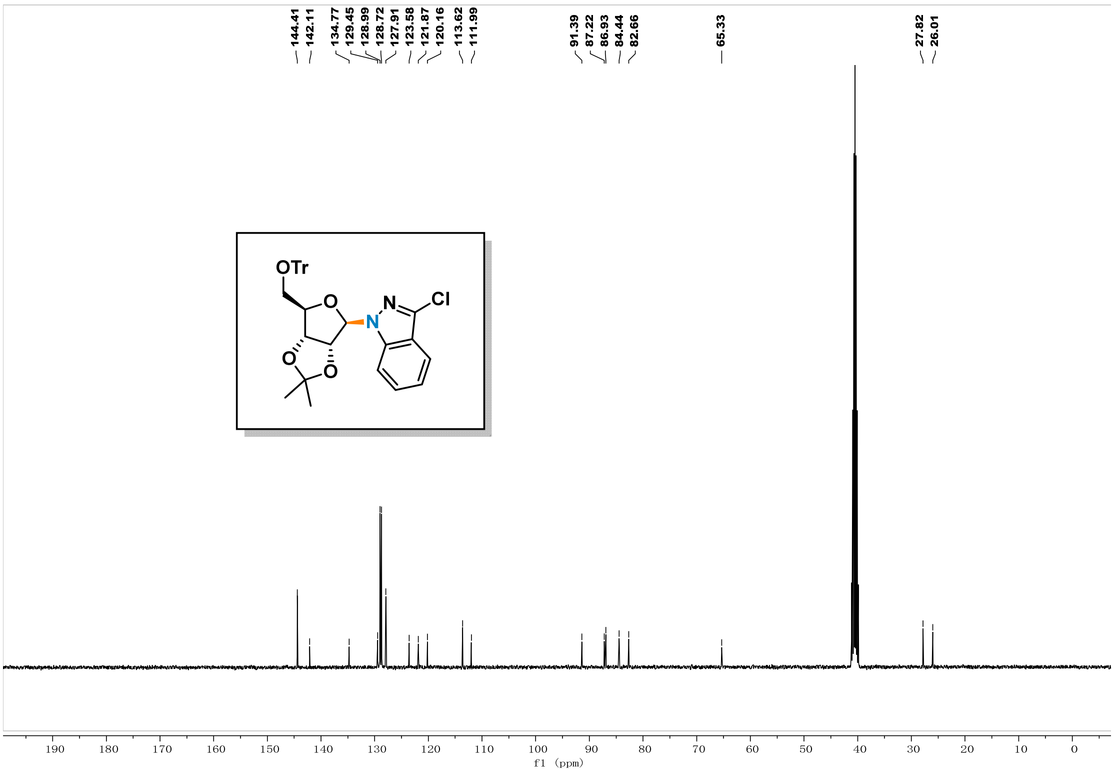

Supplementary Figure 169 | ^{13}C NMR (101 MHz, $CDCl_3$) **(8c)**

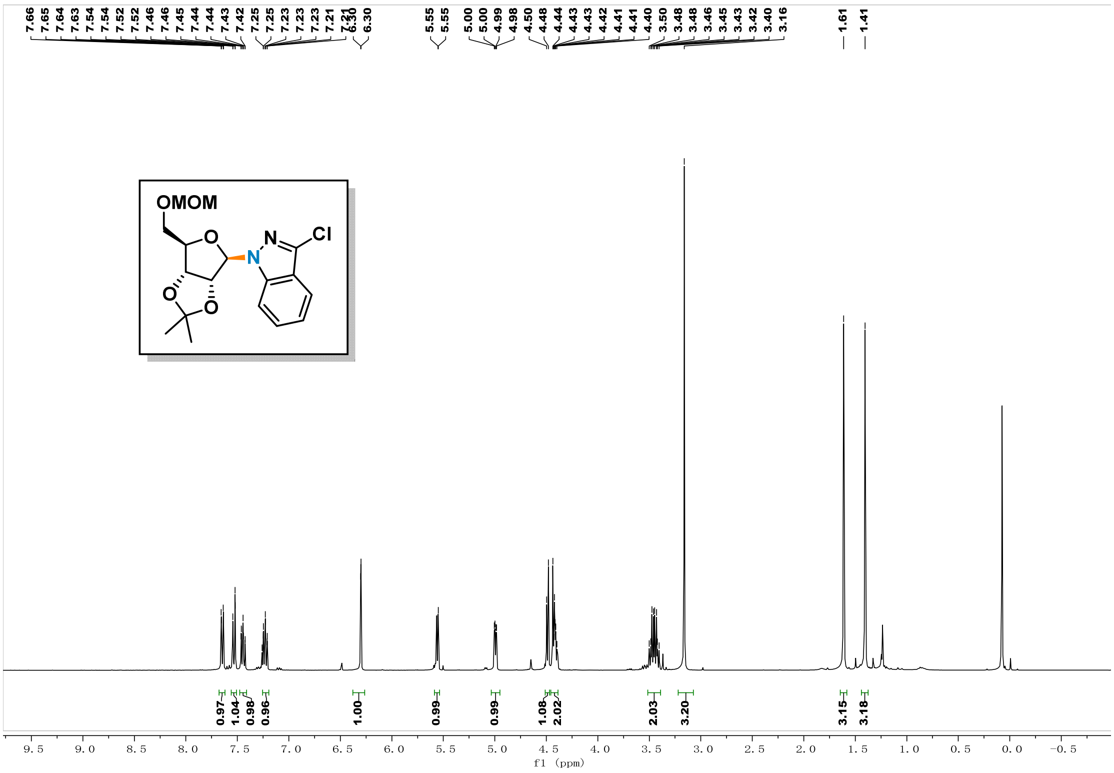

Supplementary Figure 170 | ^{1}H NMR (400 MHz, $CDCl_3$) **(8d)**

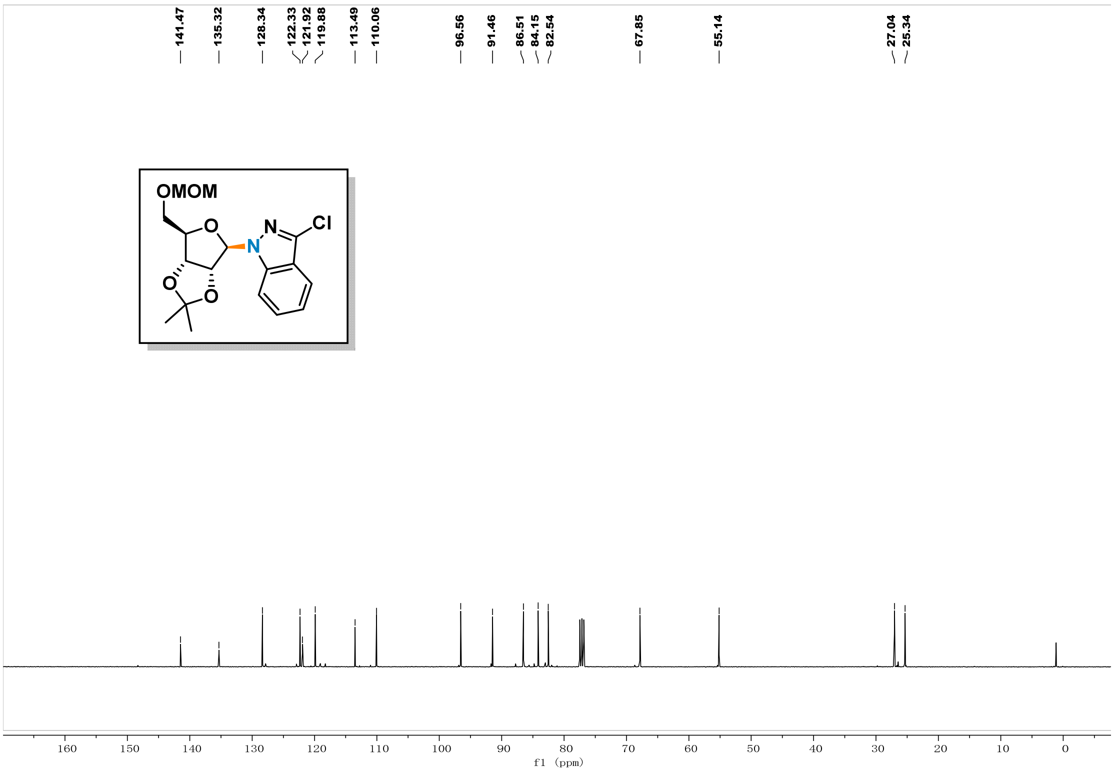

Supplementary Figure 171 | ^{13}C NMR (101 MHz, $CDCl_3$) **(8d)**

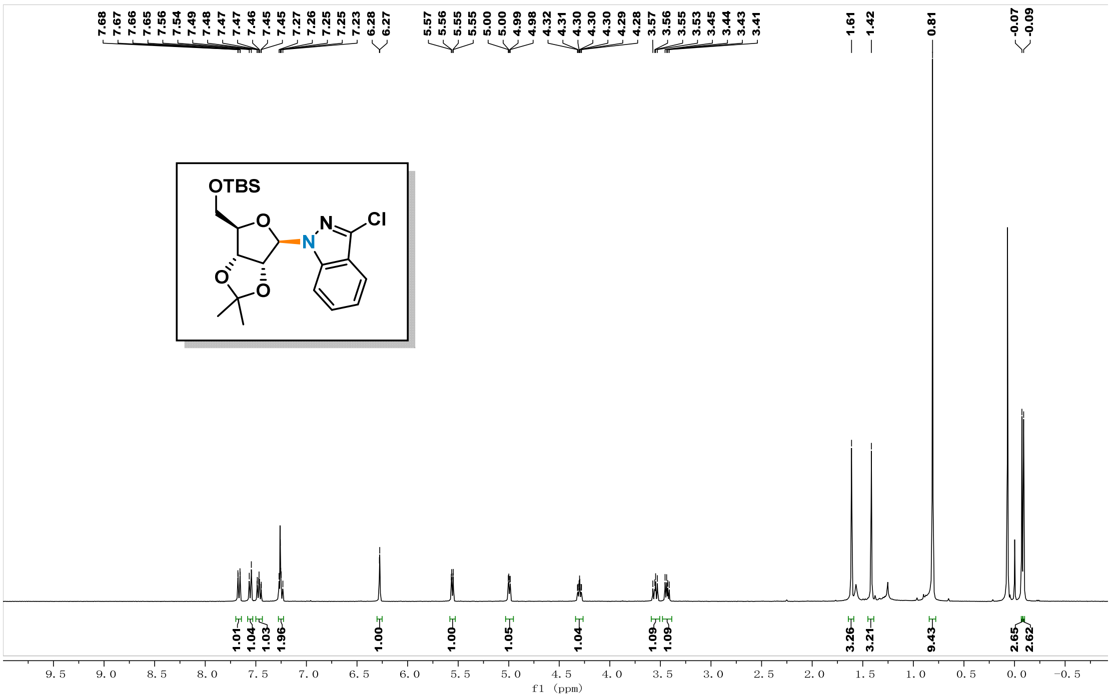

Supplementary Figure 172 | ^{1}H NMR (400 MHz, $CDCl_3$) **(8e)**

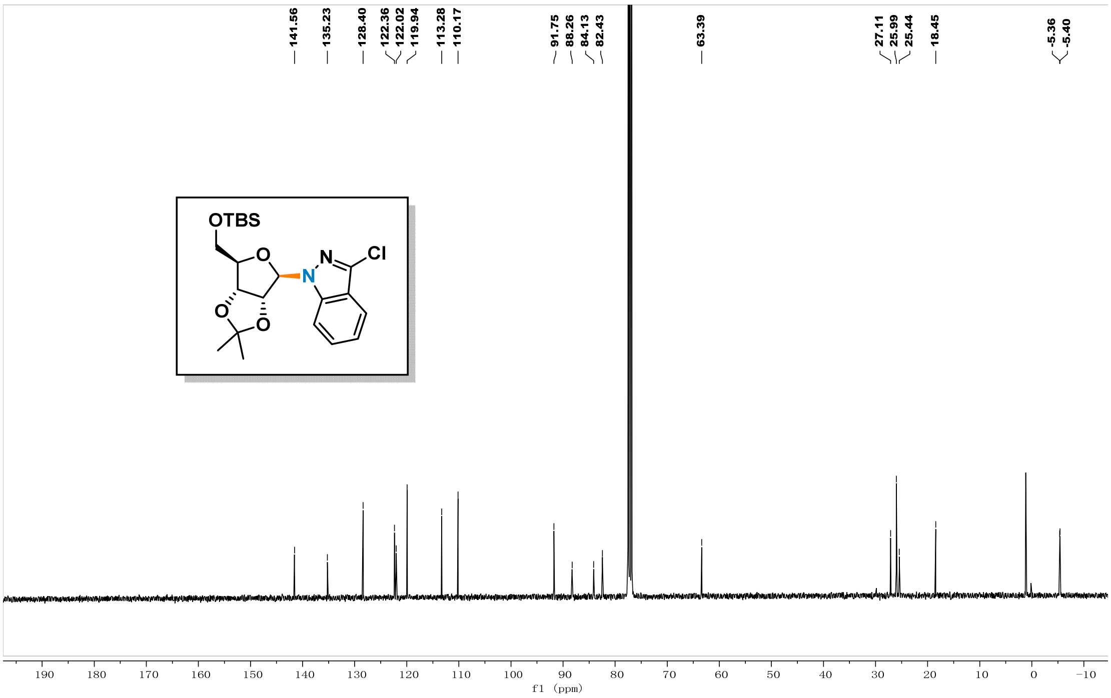

Supplementary Figure 173 | ^{13}C NMR (101 MHz, $CDCl_3$) **(8e)**

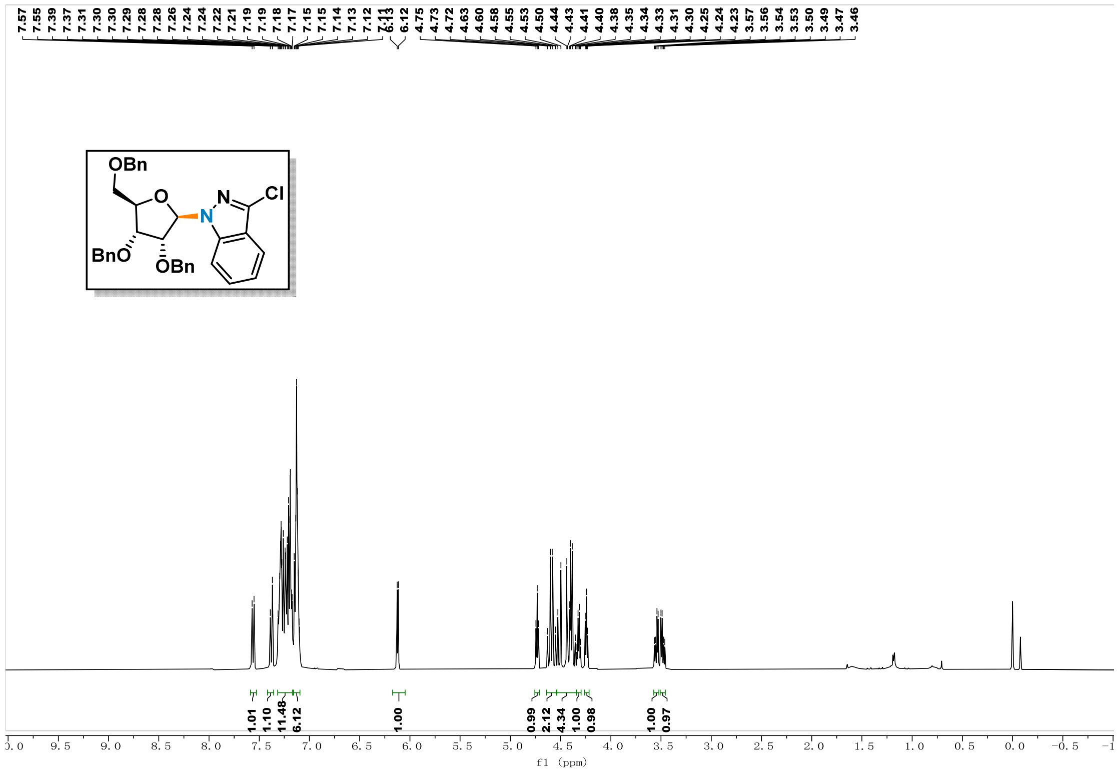

Supplementary Figure 174 | ^{1}H NMR (400 MHz, $CDCl_3$) **(8f)**

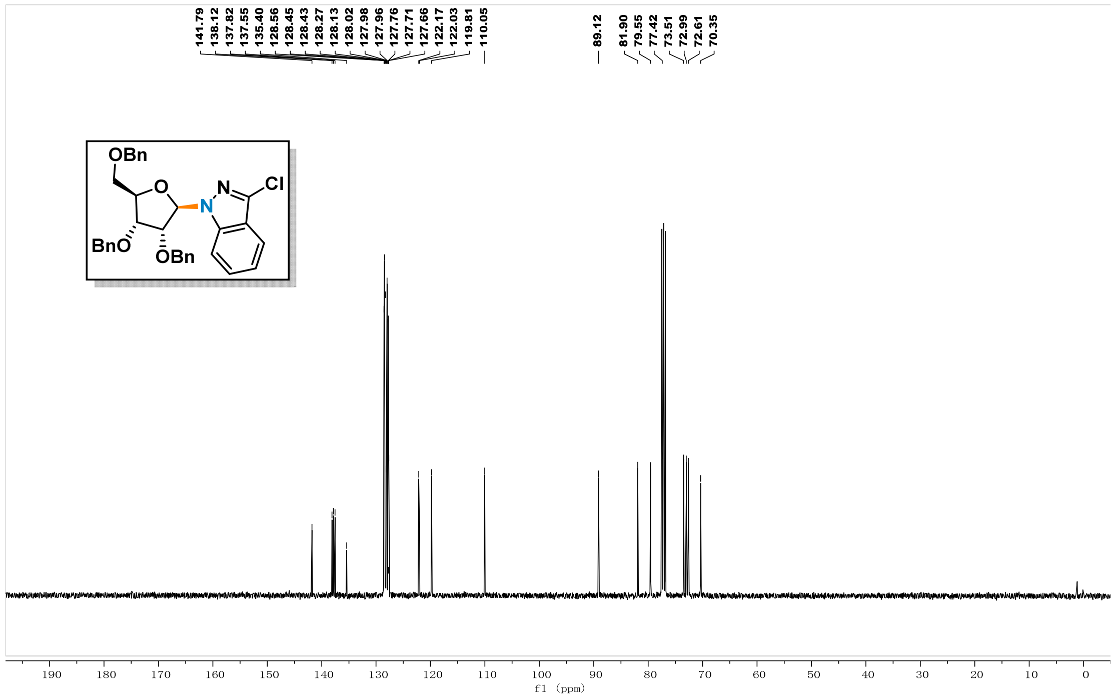

Supplementary Figure 175 | ^{13}C NMR (101 MHz, $CDCl_3$) **(8f)**

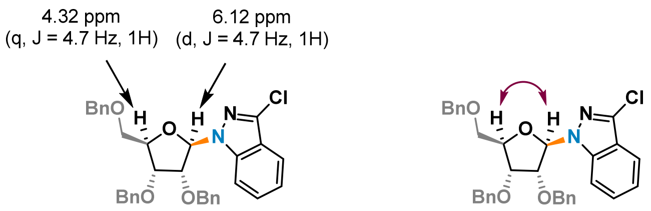

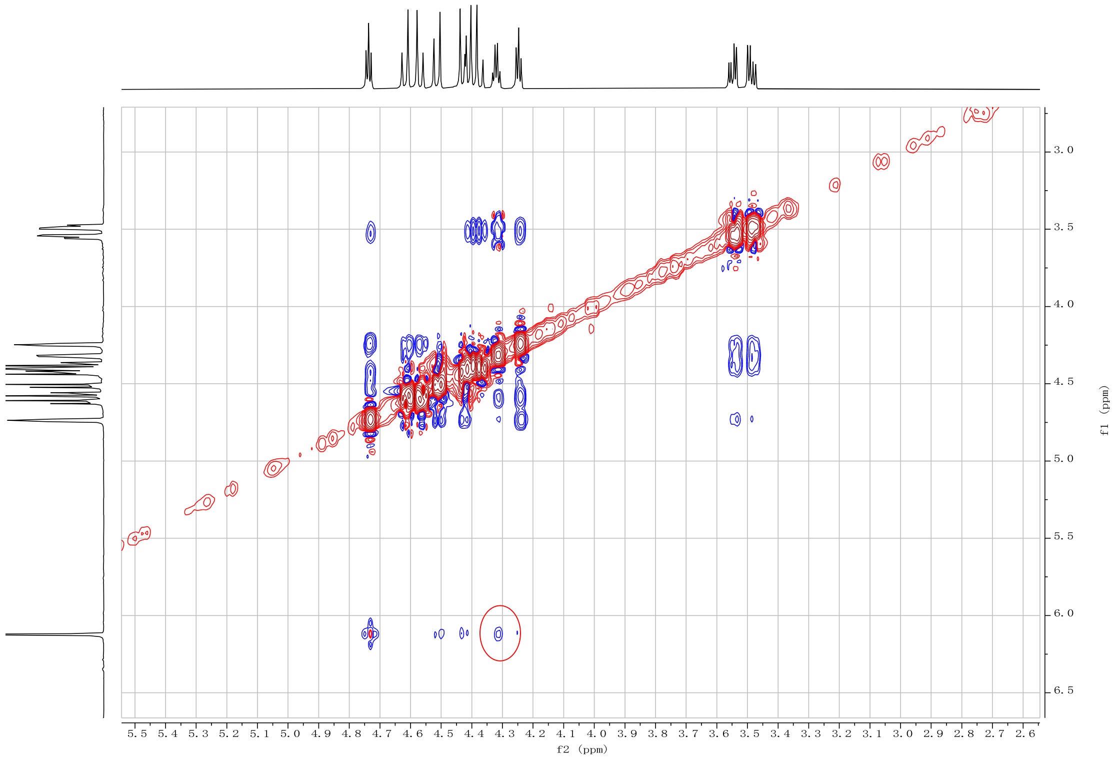

Supplementary Figure 176 | NOESY of **8f**

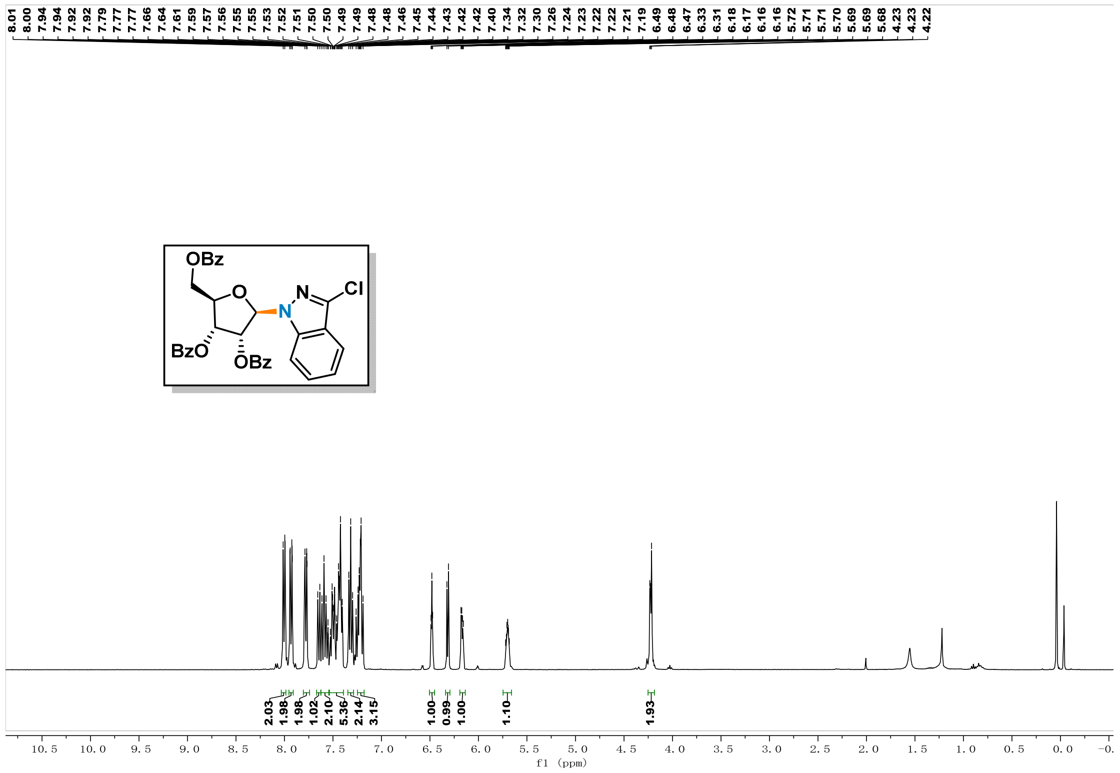

Supplementary Figure 177 | ^{1}H NMR (400 MHz, CDCl3) **(8g)**

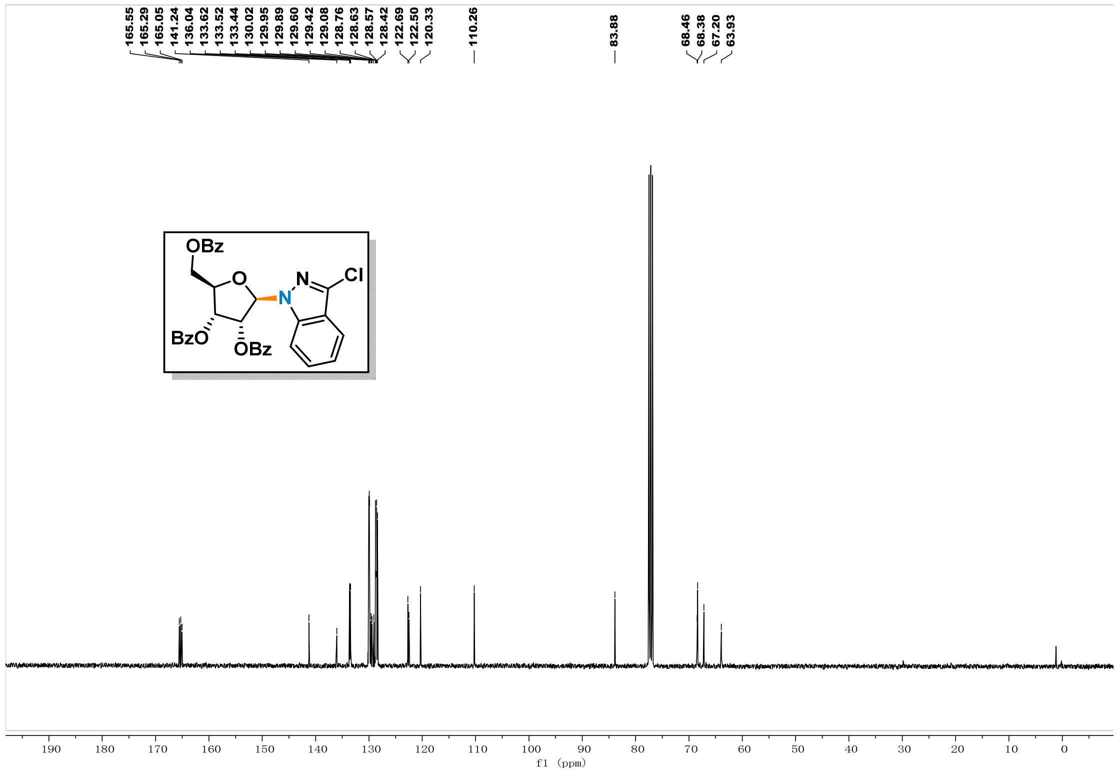

Supplementary Figure 178 | ^{13}C NMR (101 MHz, CDCl3) **(8g)**

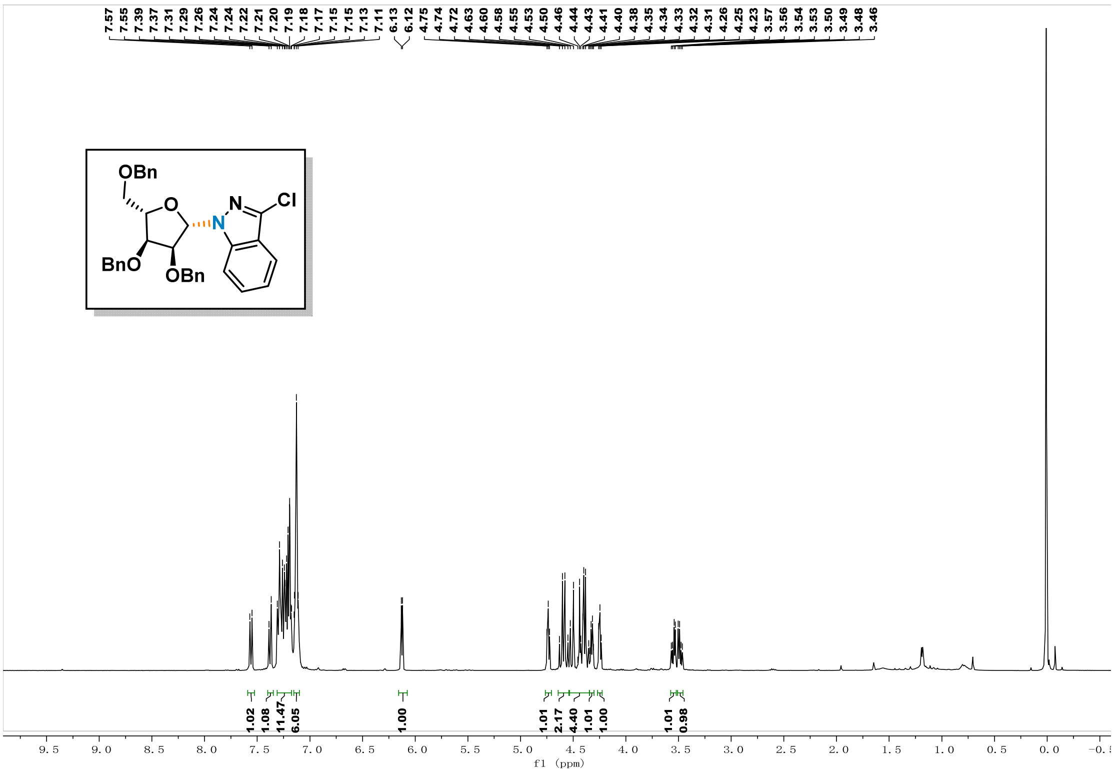

Supplementary Figure 179 | ^{1}H NMR (400 MHz, $CDCl_3$) **(8h)**

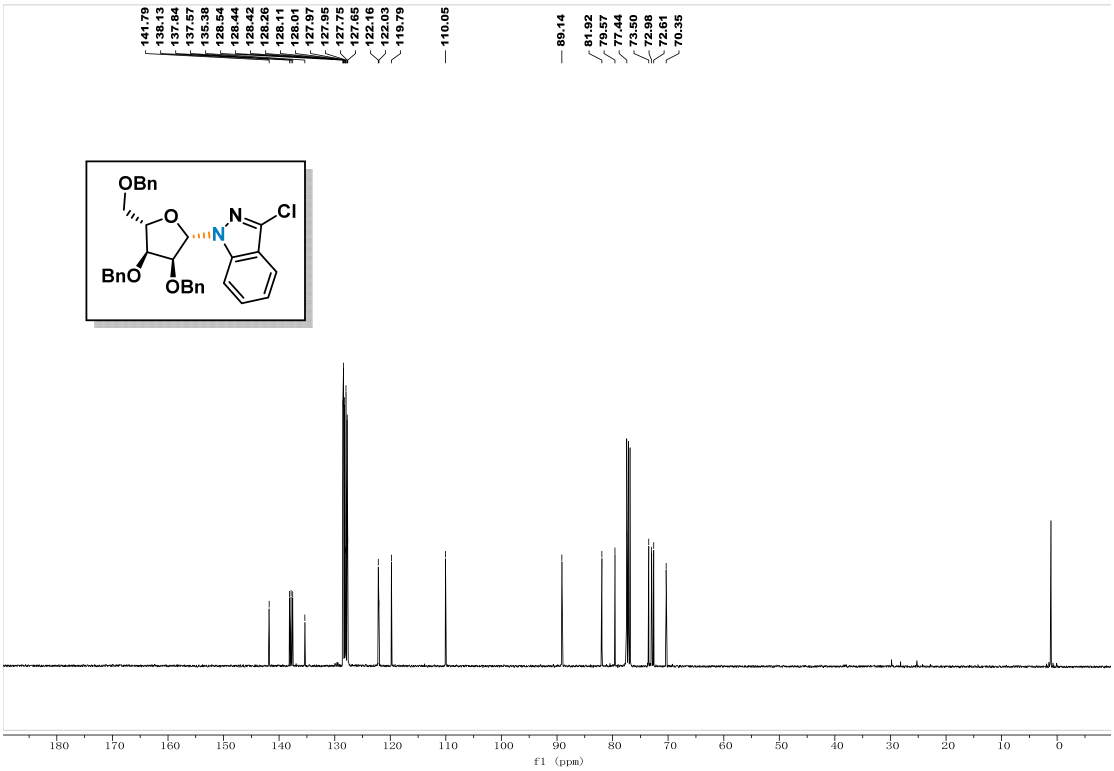

Supplementary Figure 180 | ^{13}C NMR (101 MHz, $CDCl_3$) **(8h)**

NOESY of **8h**

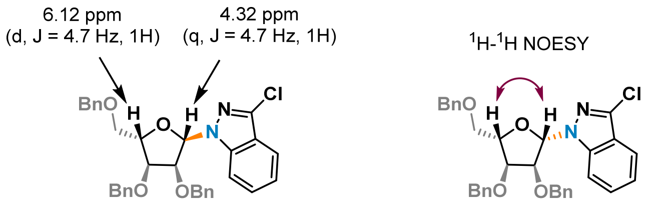

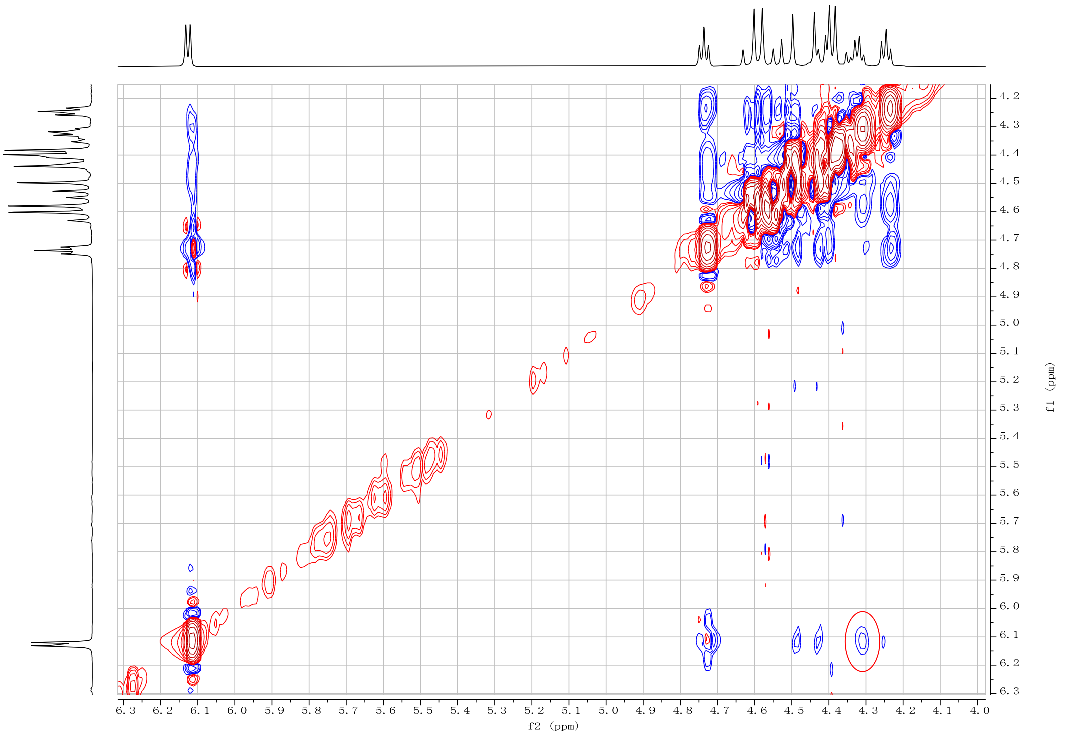

Supplementary Figure 181 | NOESY of **8h**

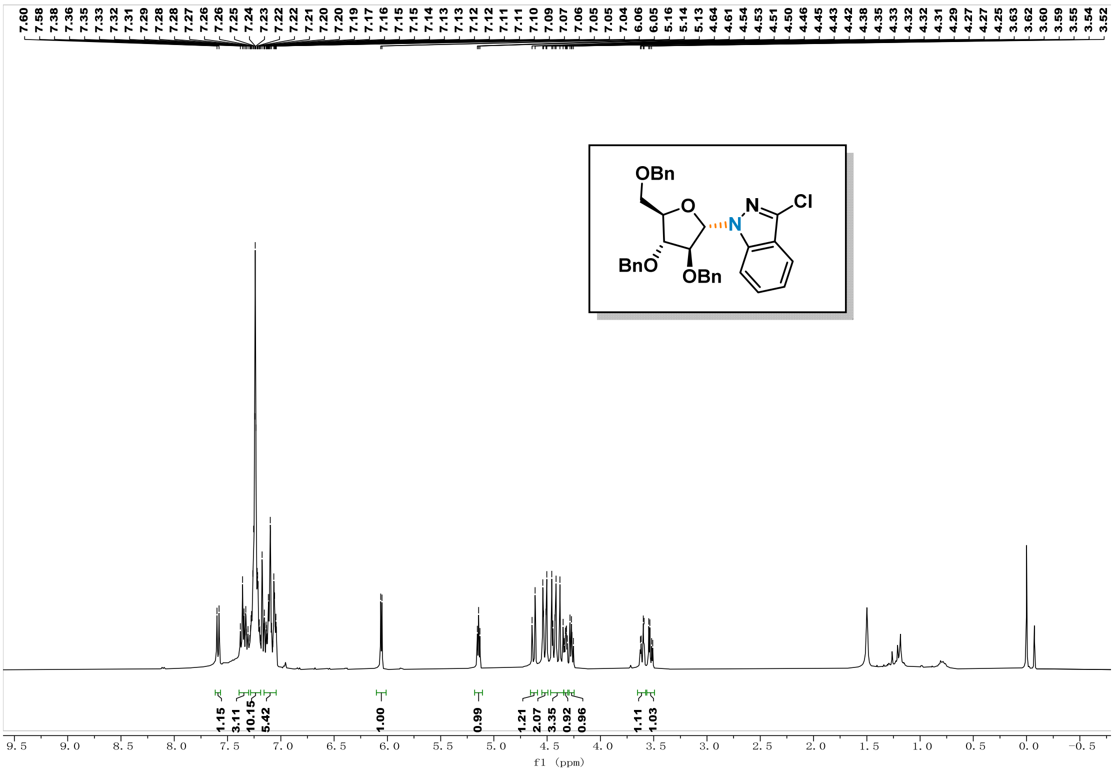

Supplementary Figure 182 | ^{1}H NMR (400 MHz, $CDCl_3$) **(8i)**

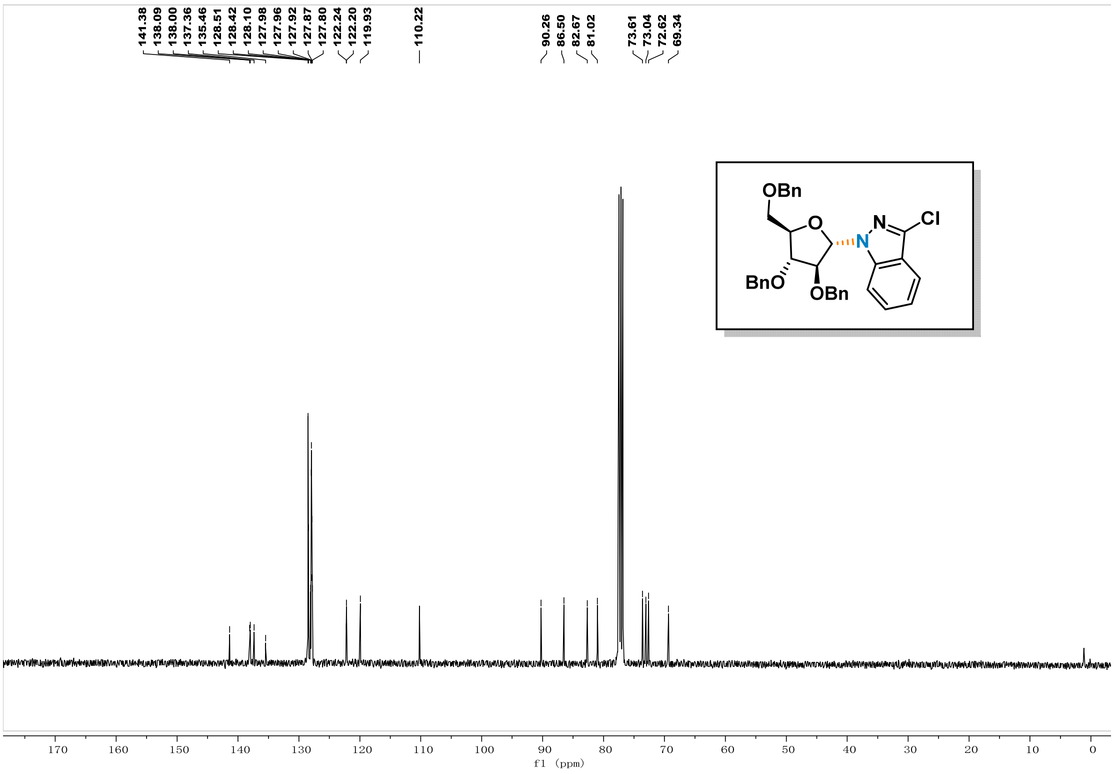

Supplementary Figure 183 | ^{13}C NMR (101 MHz, $CDCl_3$) **(8i)**

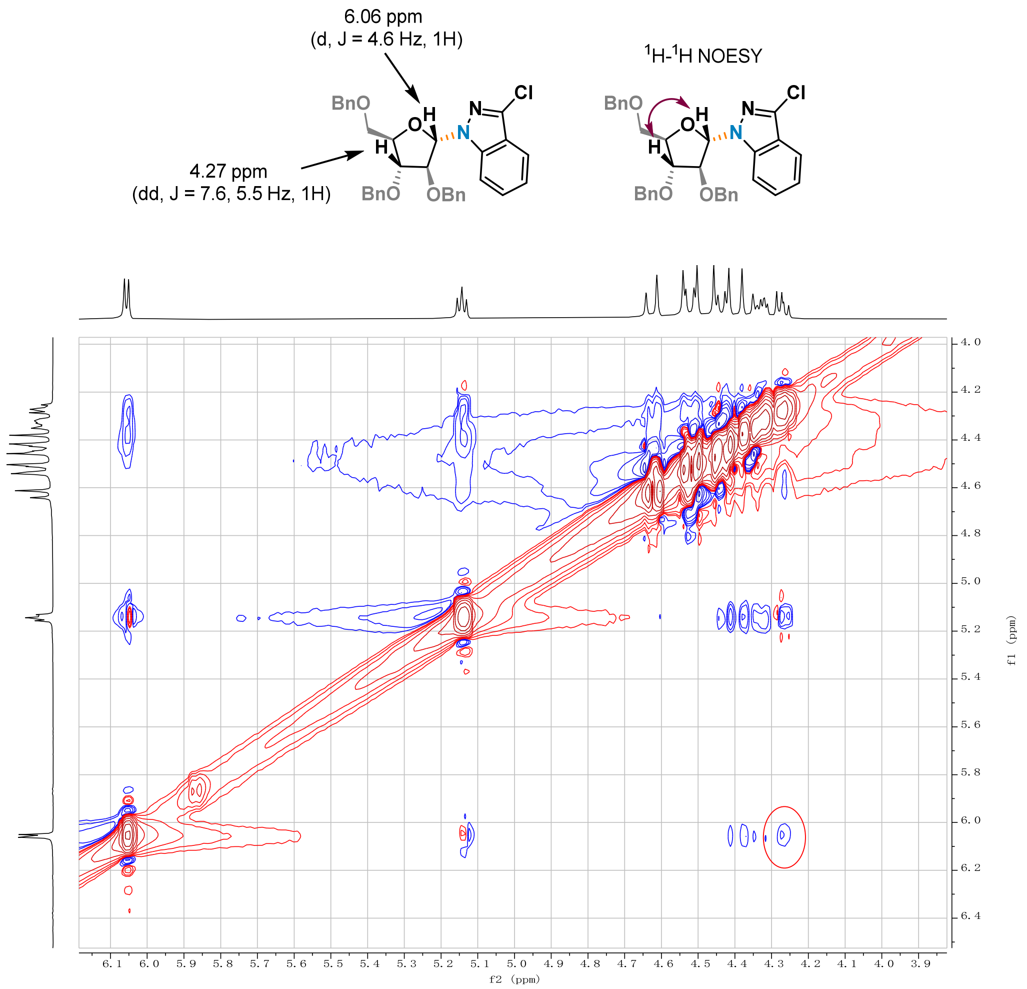

Supplementary Figure 184 | NOESY of **8i**

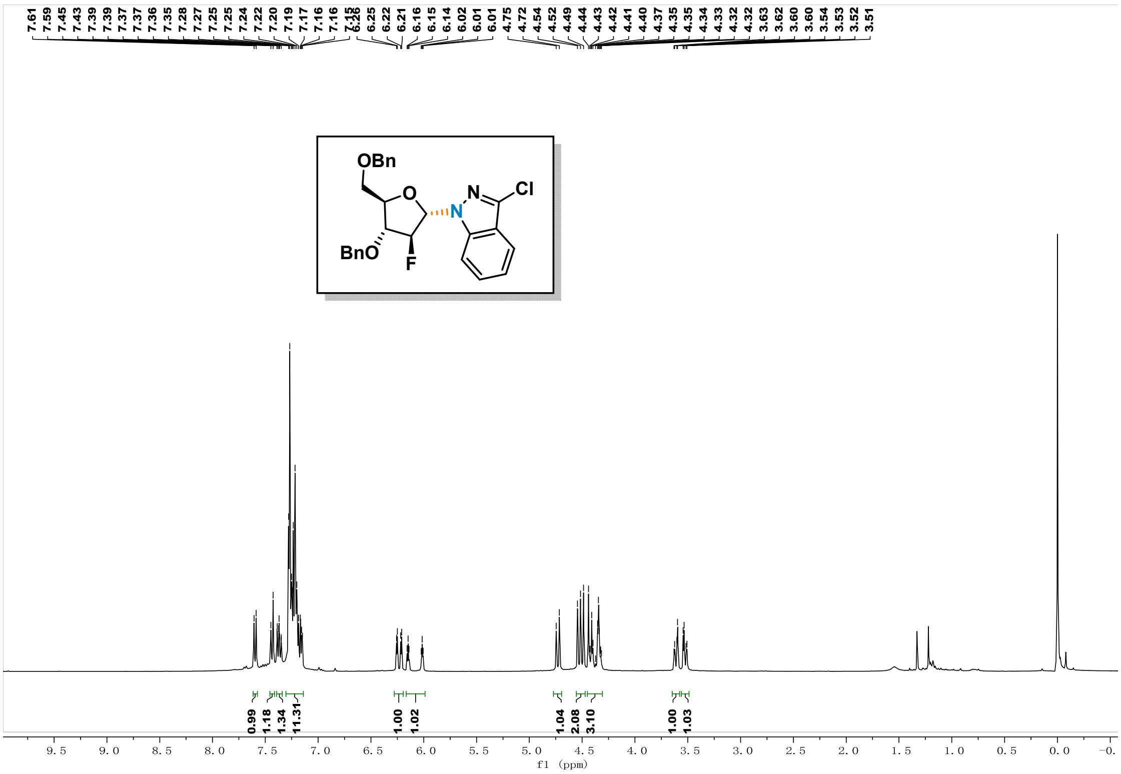

Supplementary Figure 185 | ^{1}H NMR (400 MHz, $CDCl_3$) **(8j)**

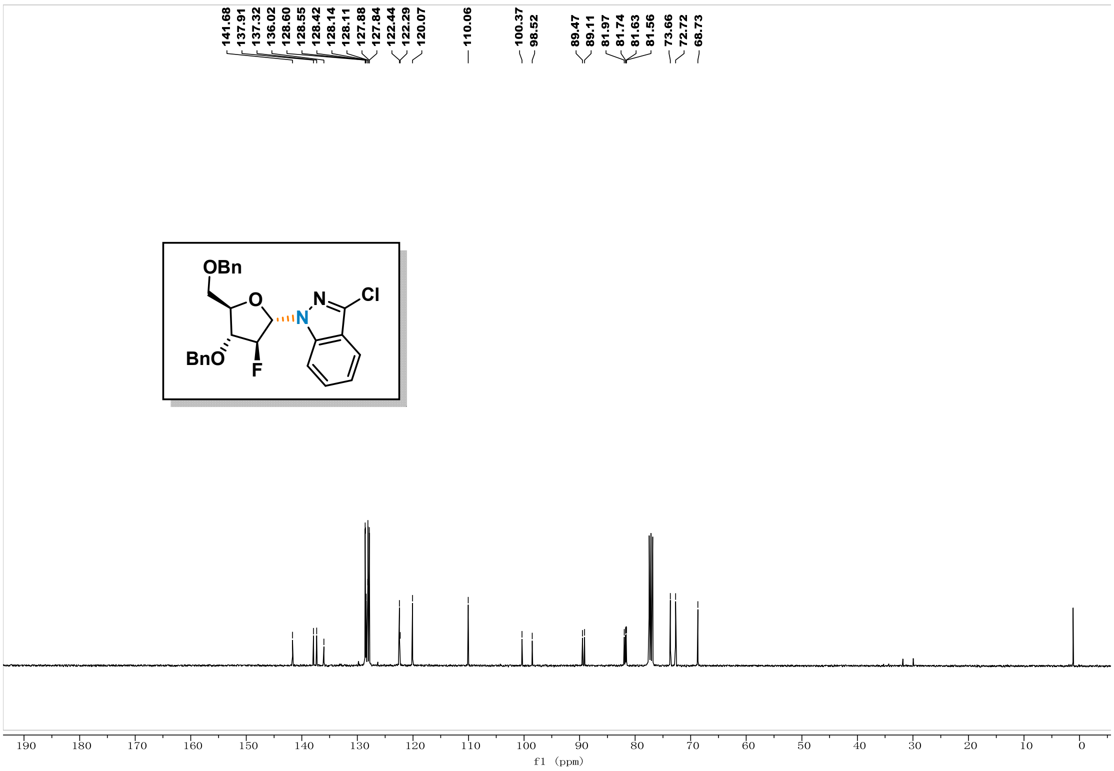

Supplementary Figure 186 | ^{13}C NMR (101 MHz, $CDCl_3$) **(8j)**

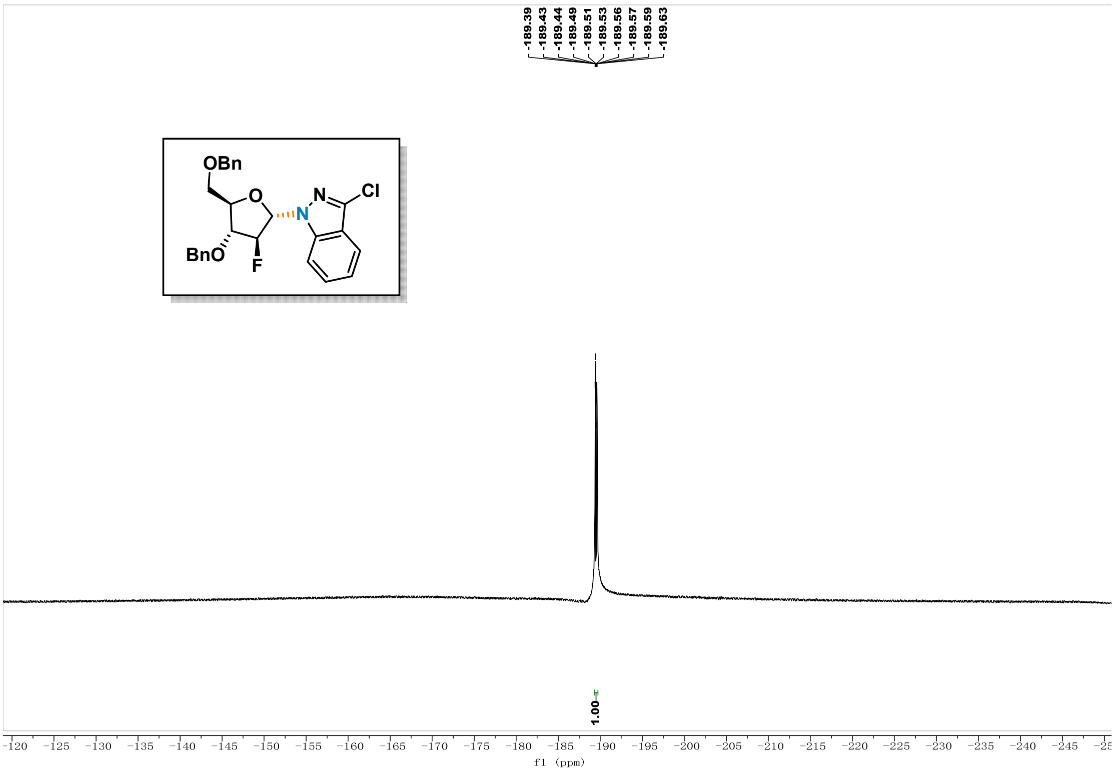

Supplementary Figure 187 | ^{19}F NMR (376 MHz, $CDCl_3$) **(8j)**

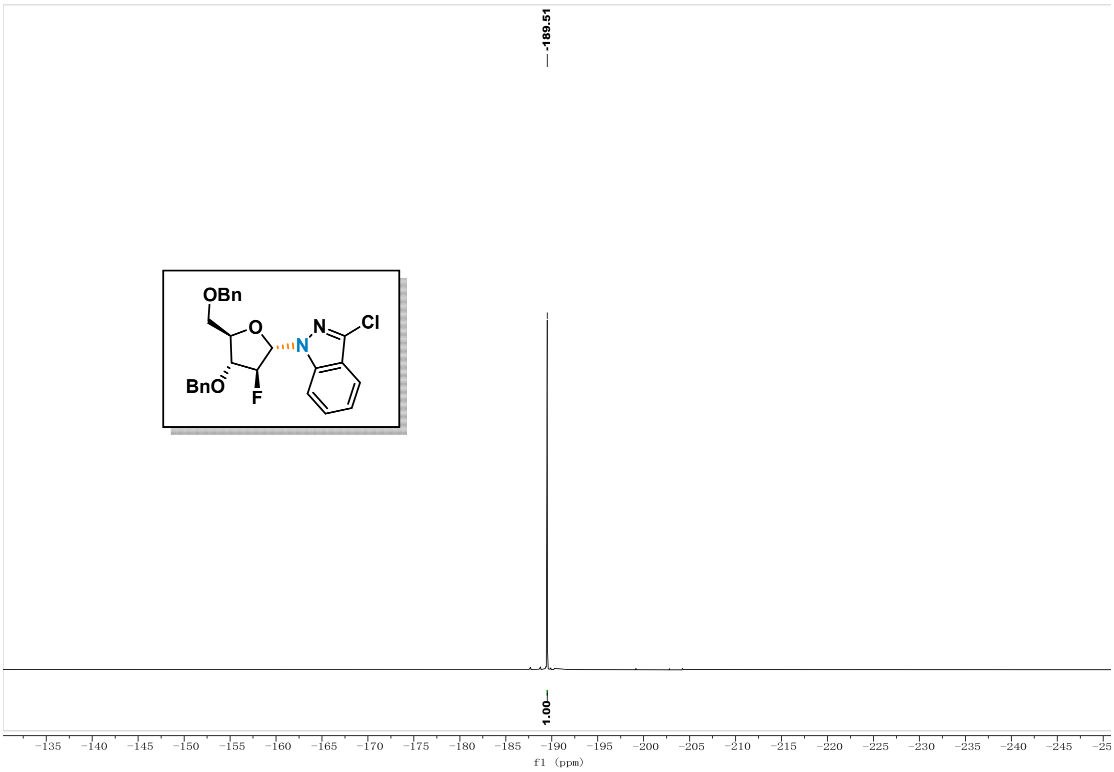

Supplementary Figure 188 | ^{19}F **{H}** NMR (376 MHz, $CDCl_3$) **(8j)**

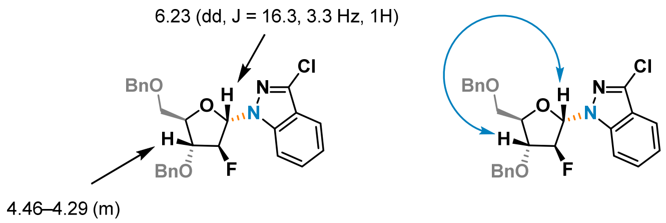

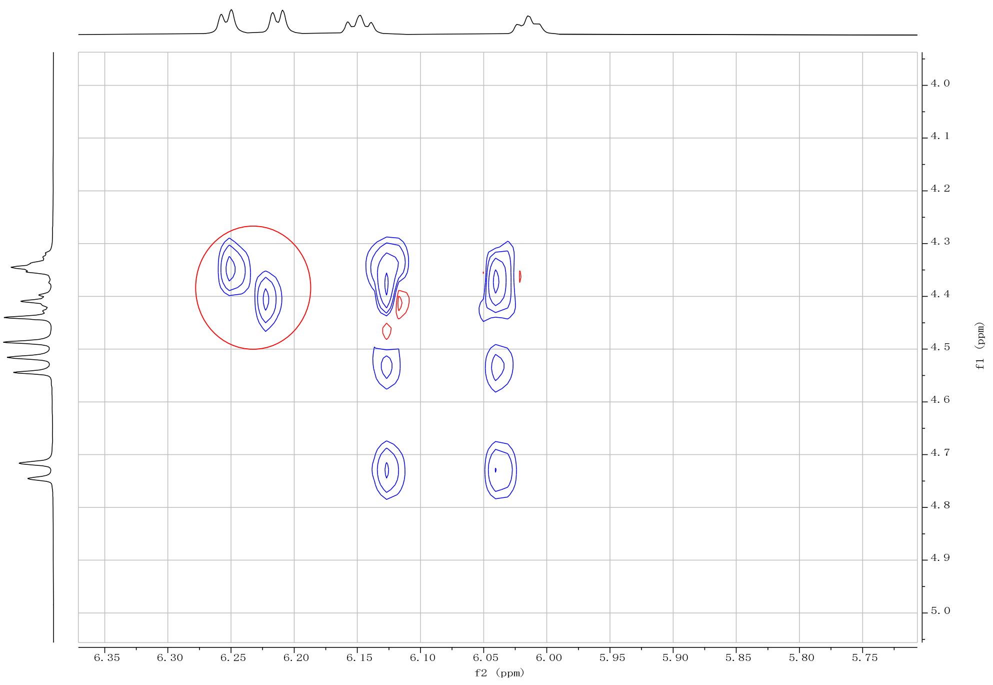

Supplementary Figure 189 | NOESY of **8j**

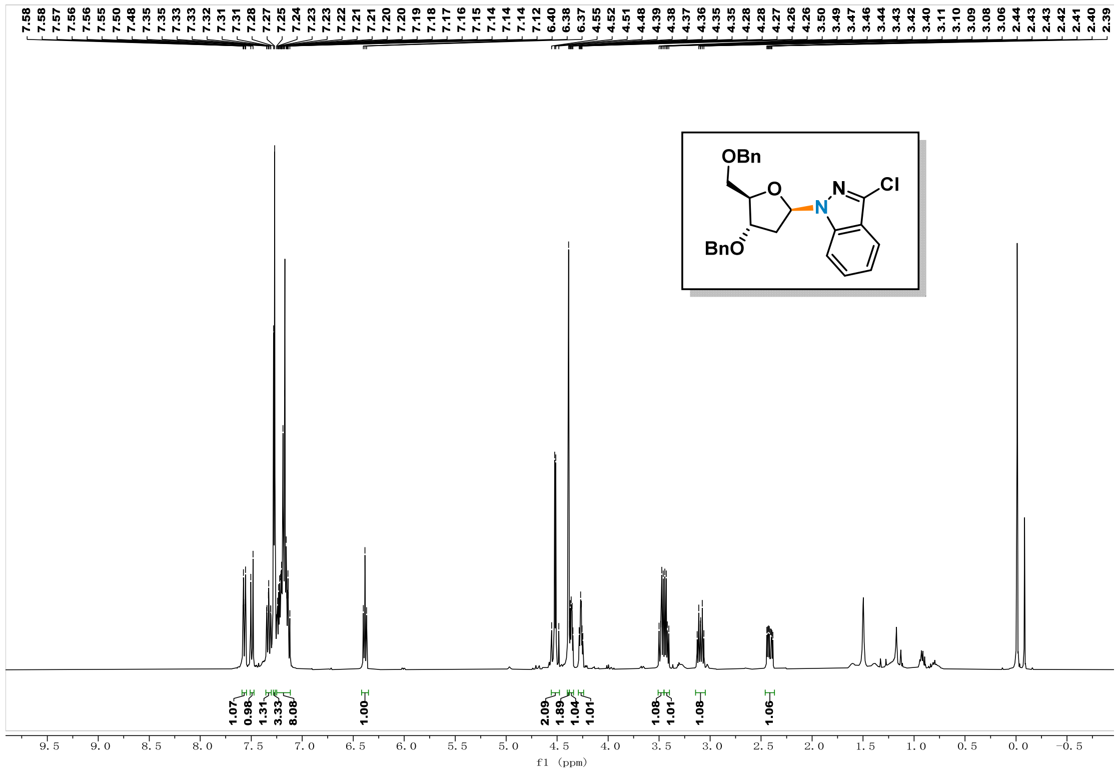

Supplementary Figure 190 | ^{1}H NMR (400 MHz, $CDCl_3$) **(8ka)**

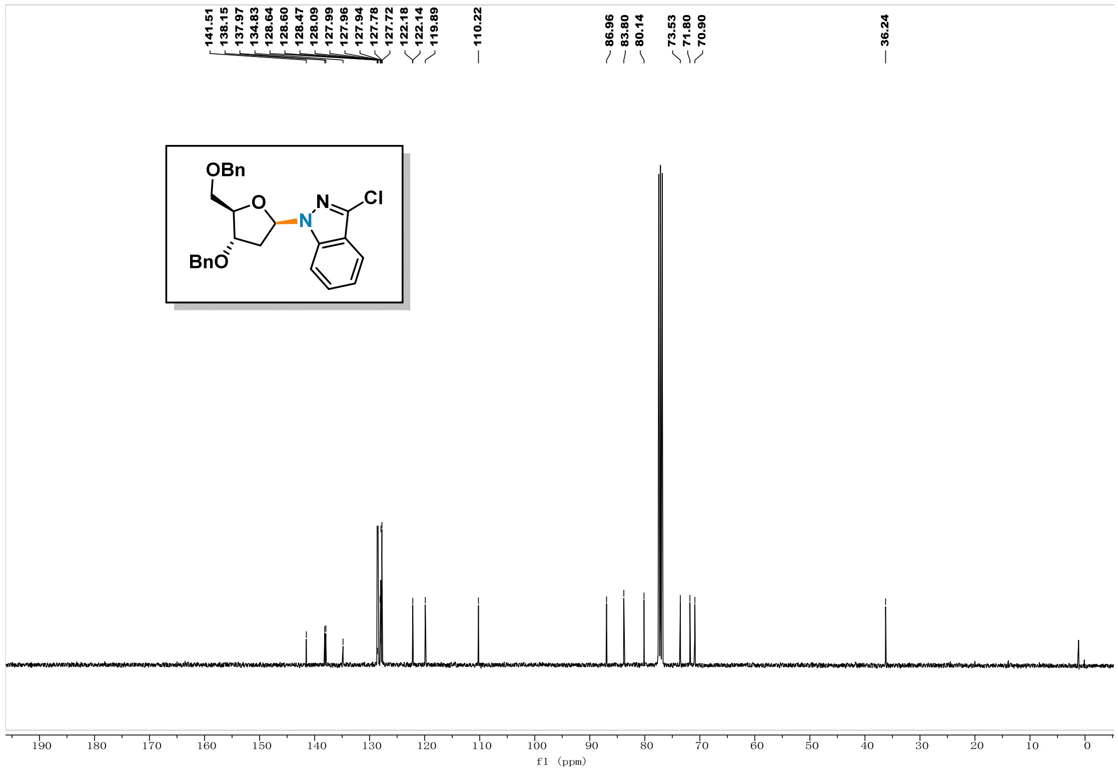

Supplementary Figure 191 | ^{13}C NMR (101 MHz, $CDCl_3$) **(8ka)**

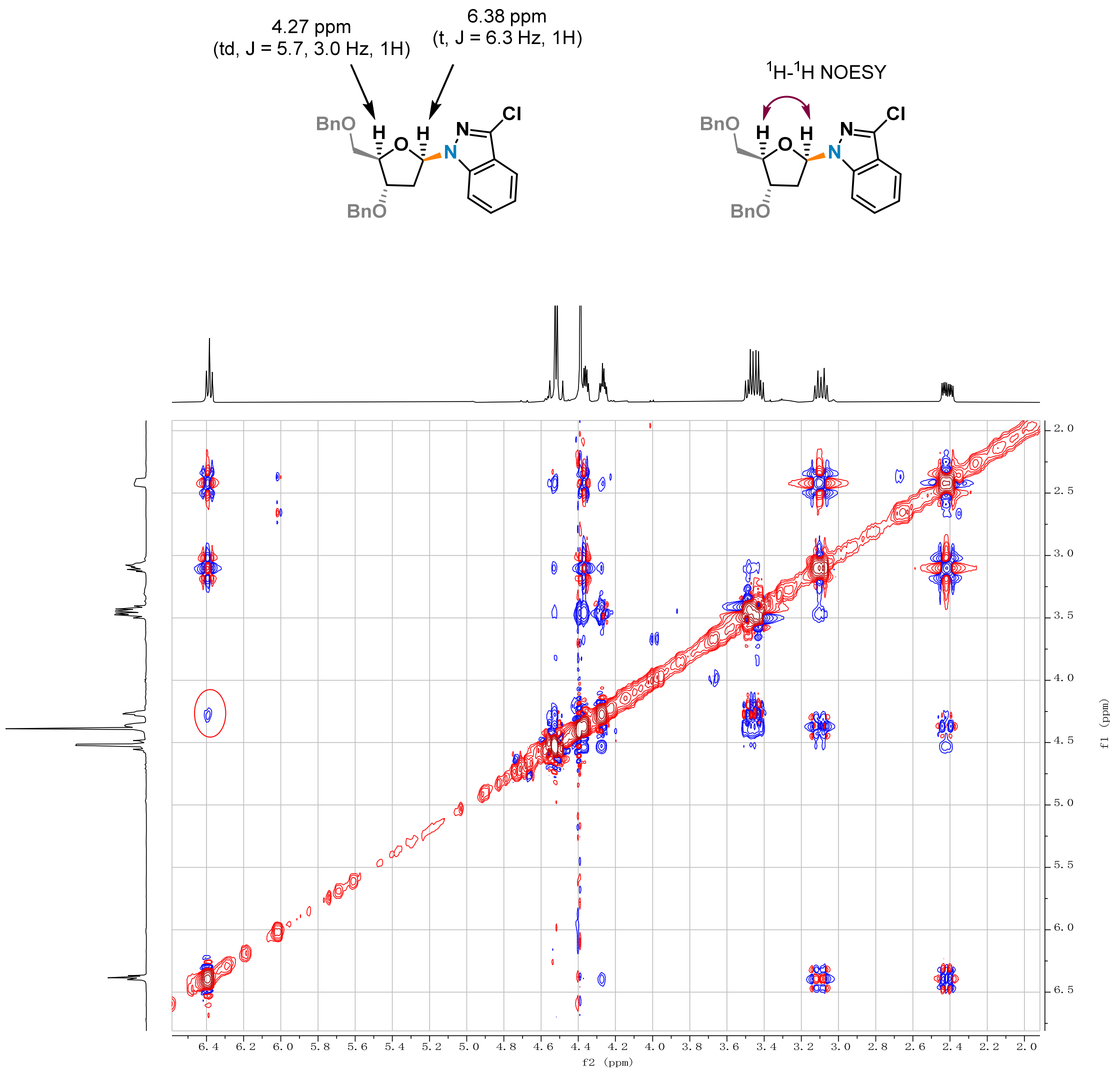

Supplementary Figure 192 | NOESY of **8ka**

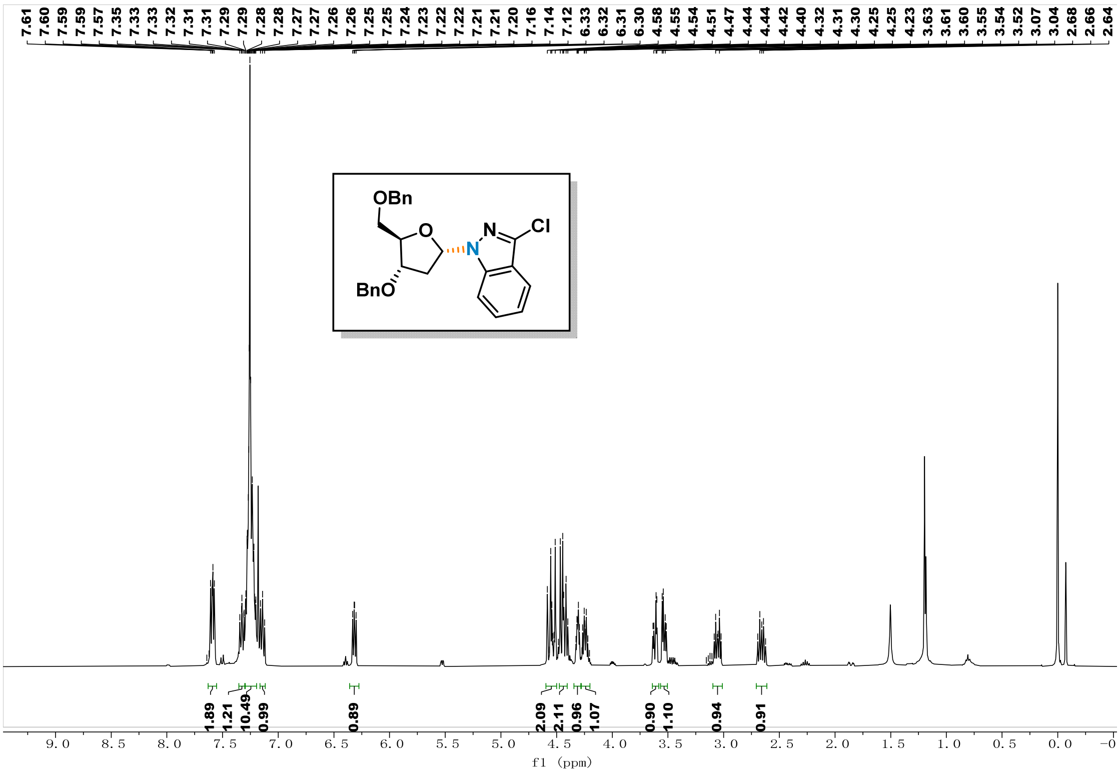

Supplementary Figure 193 | ^{1}H NMR (400 MHz, CDCl$_3$) **(8kb)**

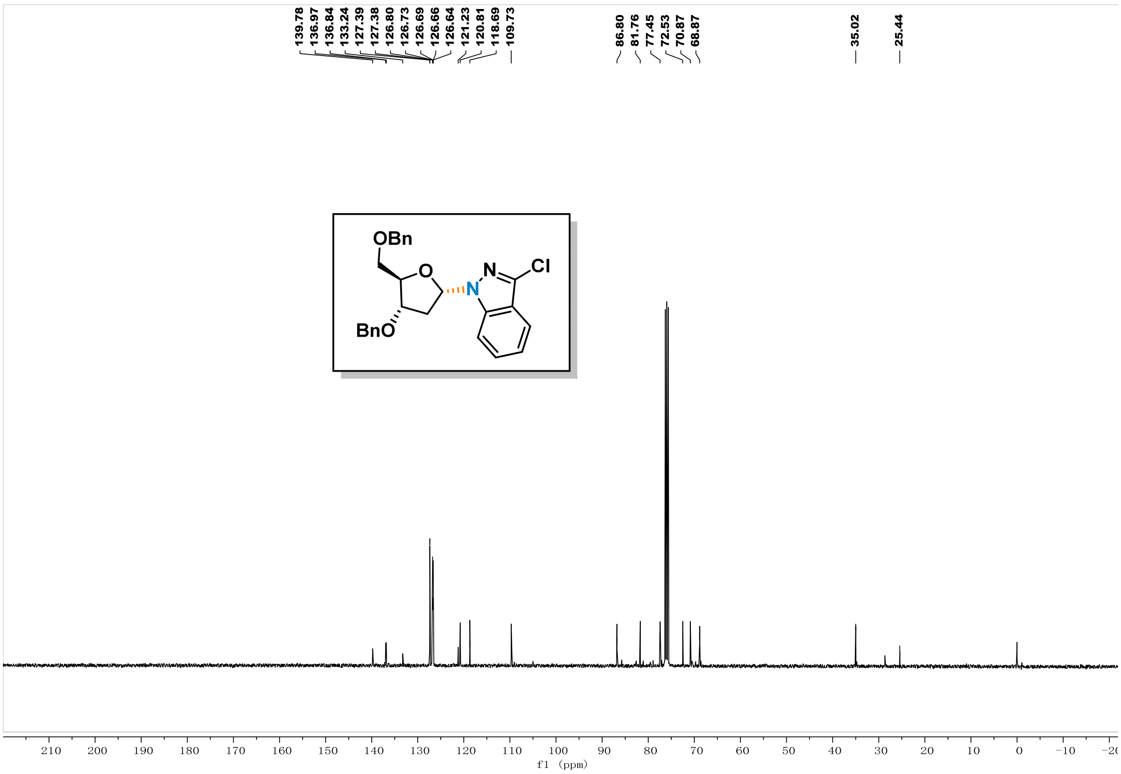

Supplementary Figure 194 | ^{13}C NMR (101 MHz, CDCl$_3$) **(8kb)**

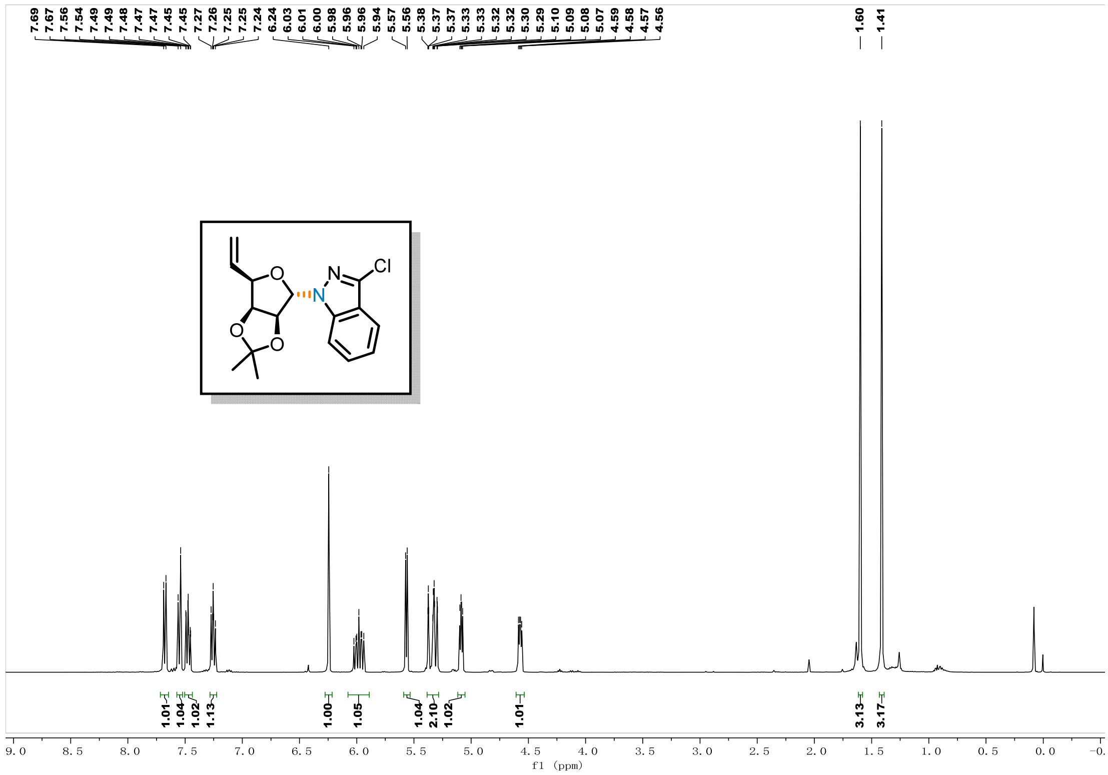

Supplementary Figure 195 | ^{1}H NMR (400 MHz, $CDCl_3$) **(8l)**

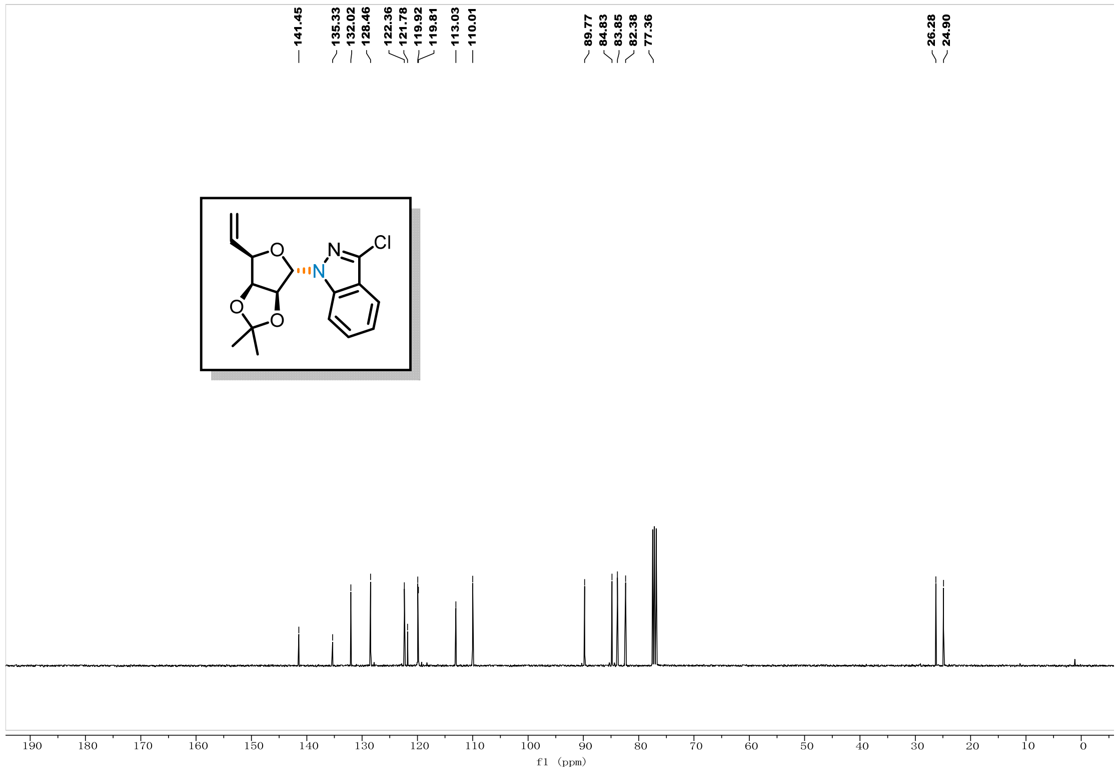

Supplementary Figure 196 | ^{13}C NMR (101 MHz, $CDCl_3$) **(8l)**

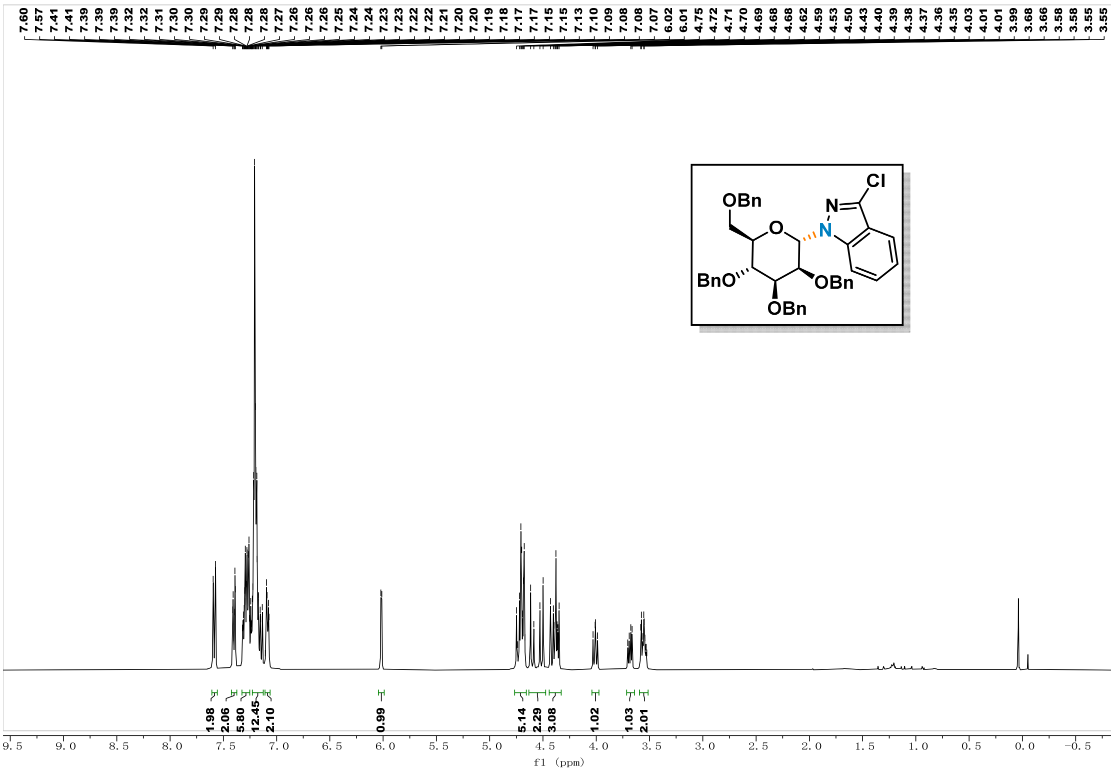

Supplementary Figure 197 | ^{1}H NMR (400 MHz, $CDCl_3$) **(8m)**

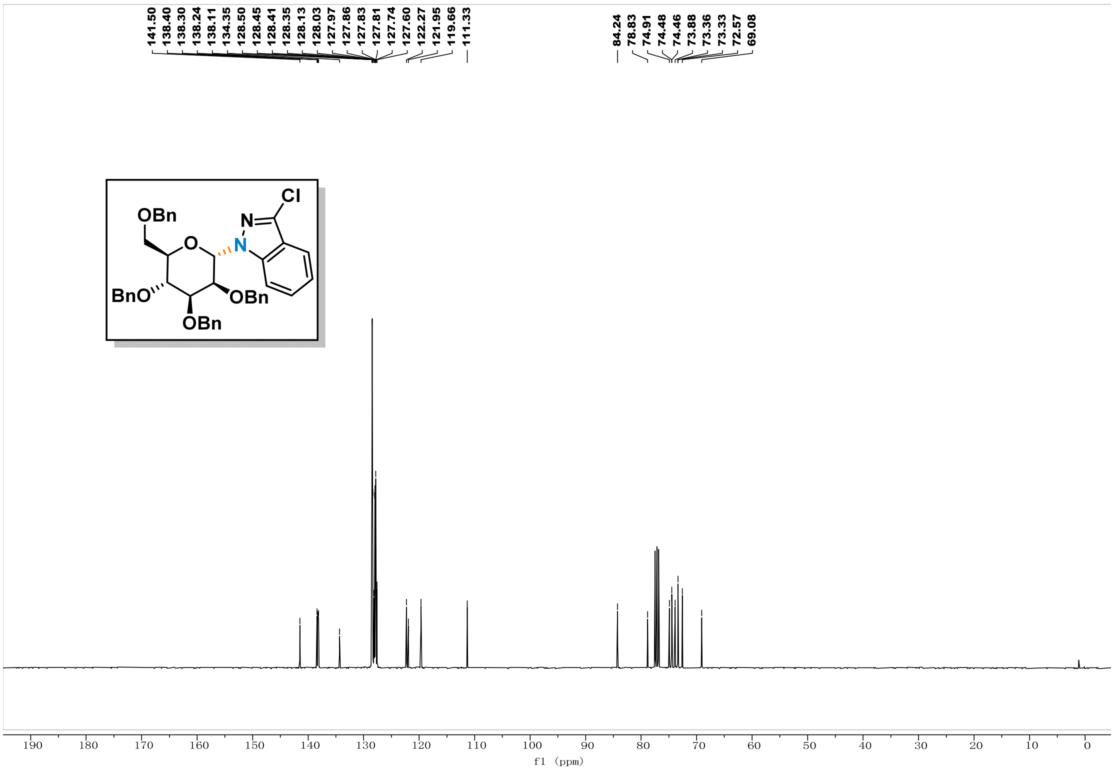

Supplementary Figure 198 | ^{13}C NMR (101 MHz, $CDCl_3$) **(8m)**

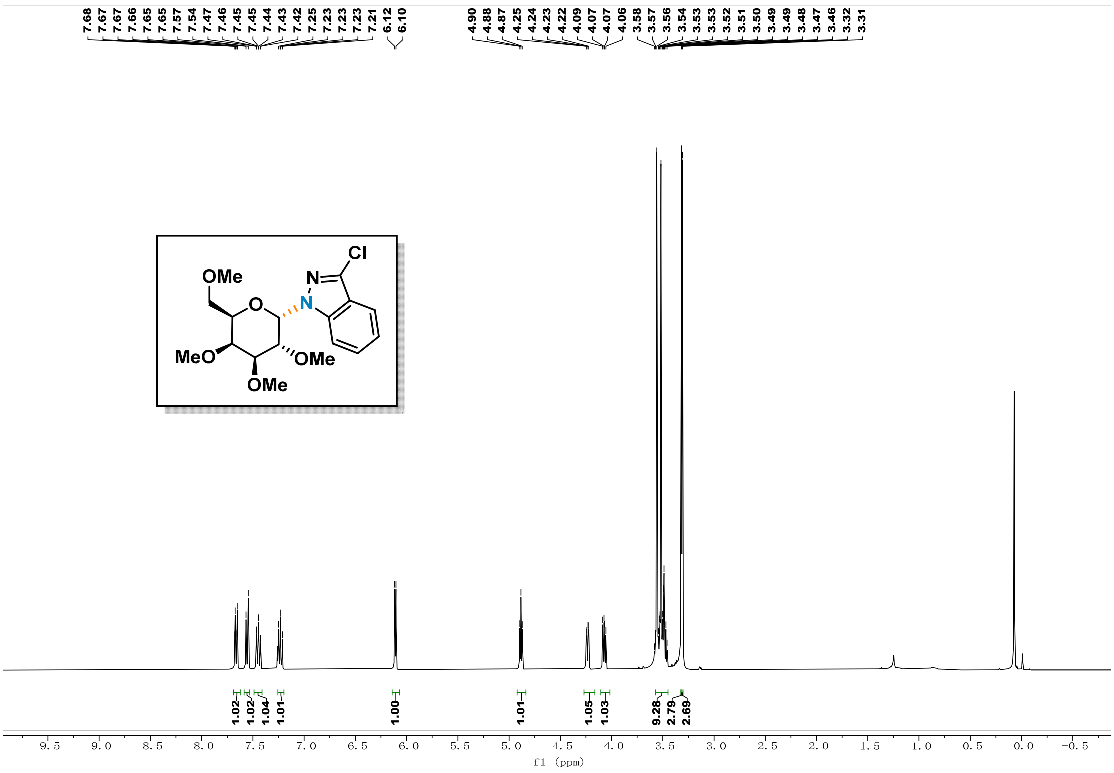

Supplementary Figure 199 | ^{1}H NMR (400 MHz, $CDCl_3$) **(8n)**

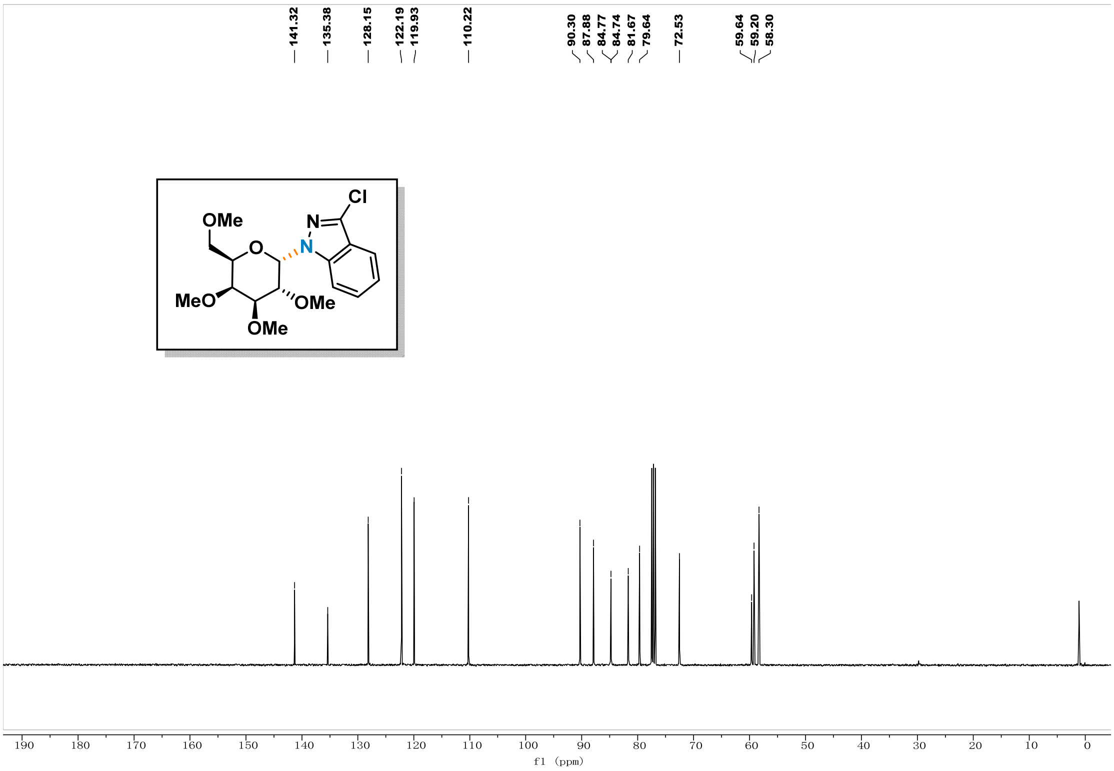

Supplementary Figure 200 | ^{13}C NMR (101 MHz, $CDCl_3$) **(8n)**

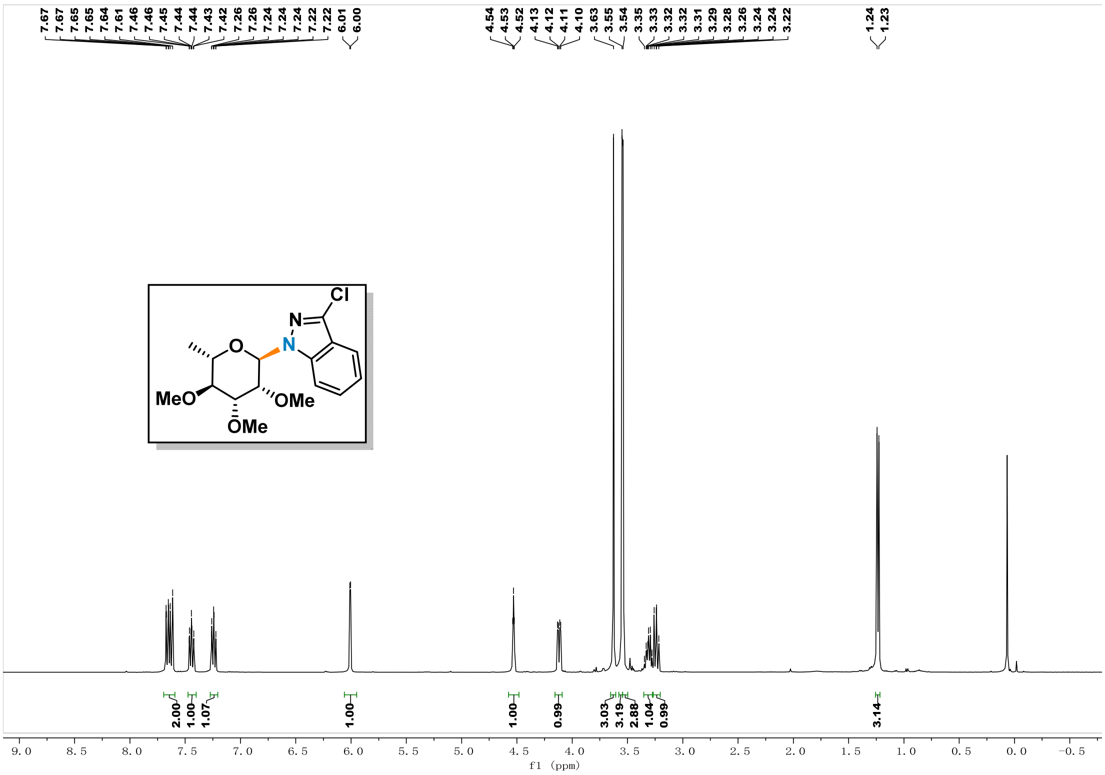

Supplementary Figure 201 | ^{1}H NMR (400 MHz, $CDCl_3$) **(8o)**

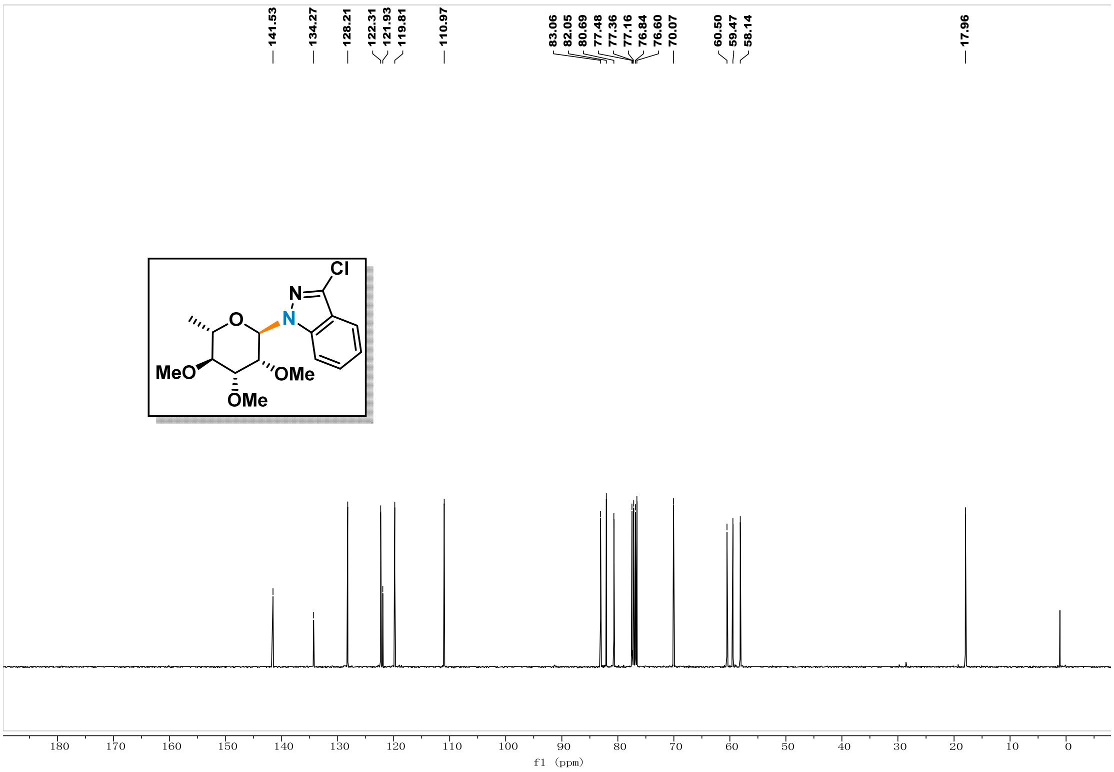

Supplementary Figure 202 | ^{13}C NMR (101 MHz, $CDCl_3$) **(8o)**

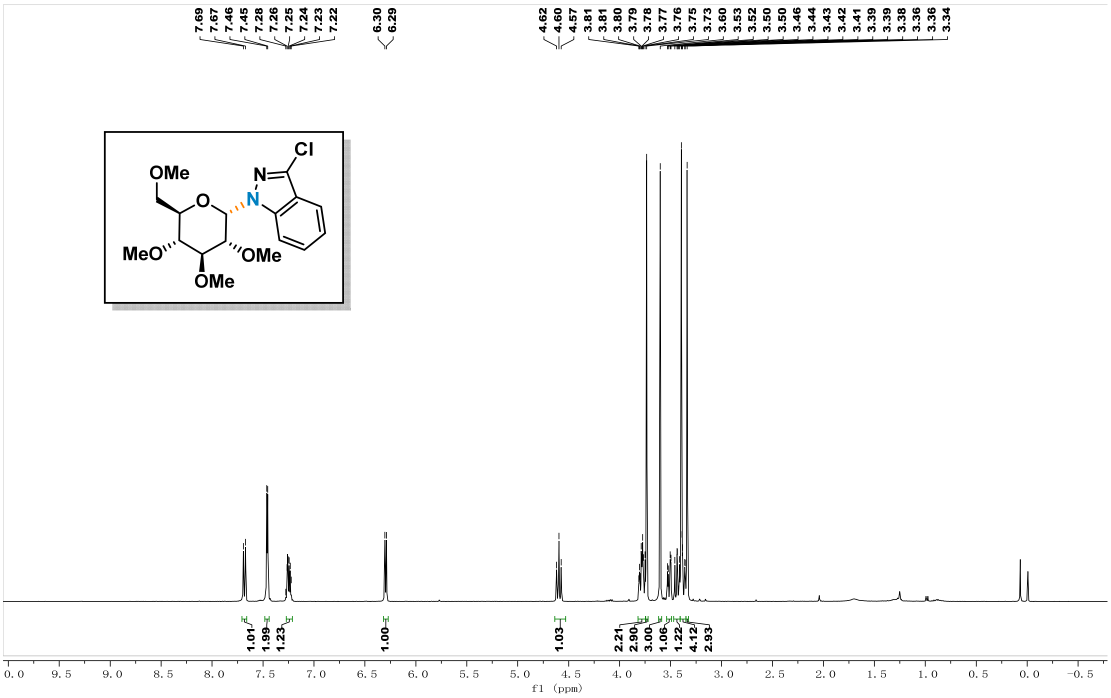

Supplementary Figure 203 | ^{1}H NMR (400 MHz, $CDCl_3$) **(8pa)**

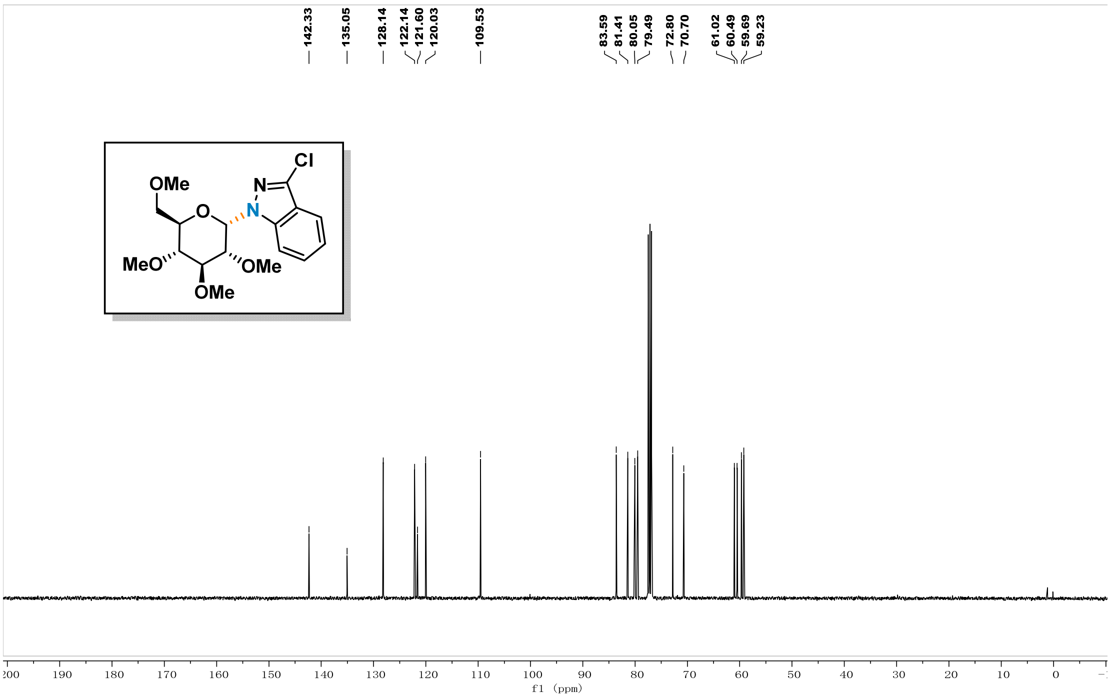

Supplementary Figure 204 | ^{13}C NMR (101 MHz, $CDCl_3$) **(8pa)**

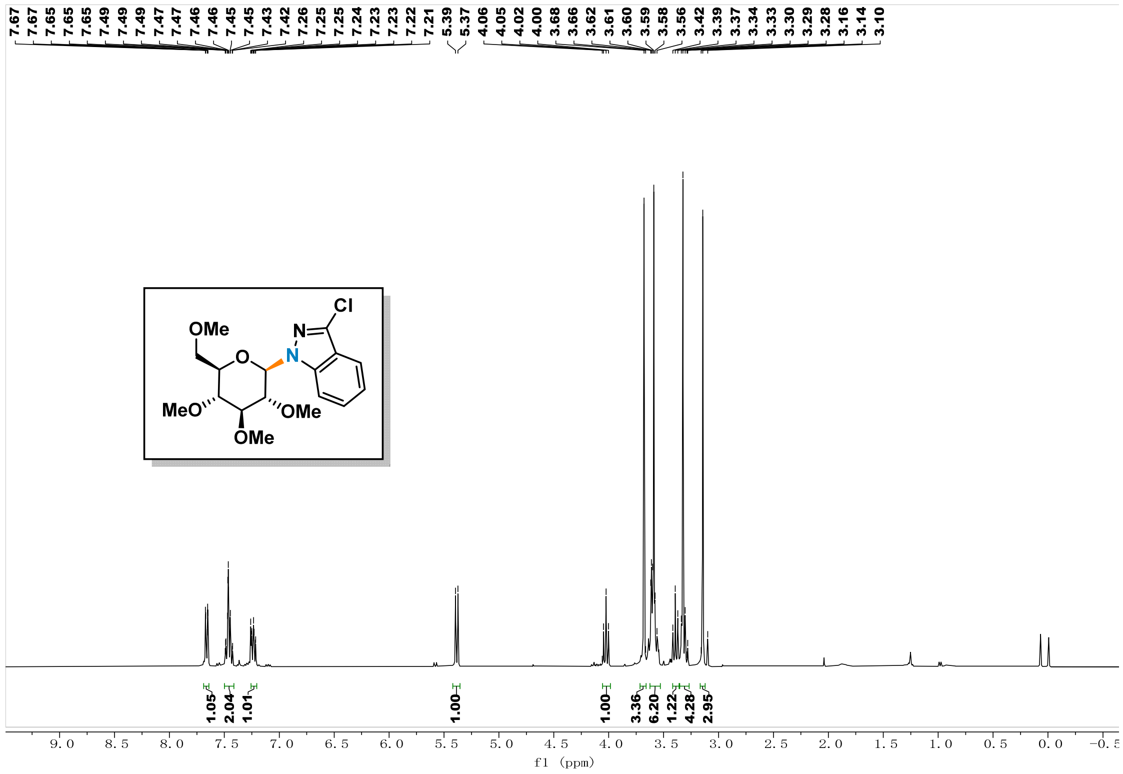

Supplementary Figure 205 | ^{1}H NMR (400 MHz, $CDCl_3$) **(8pb)**

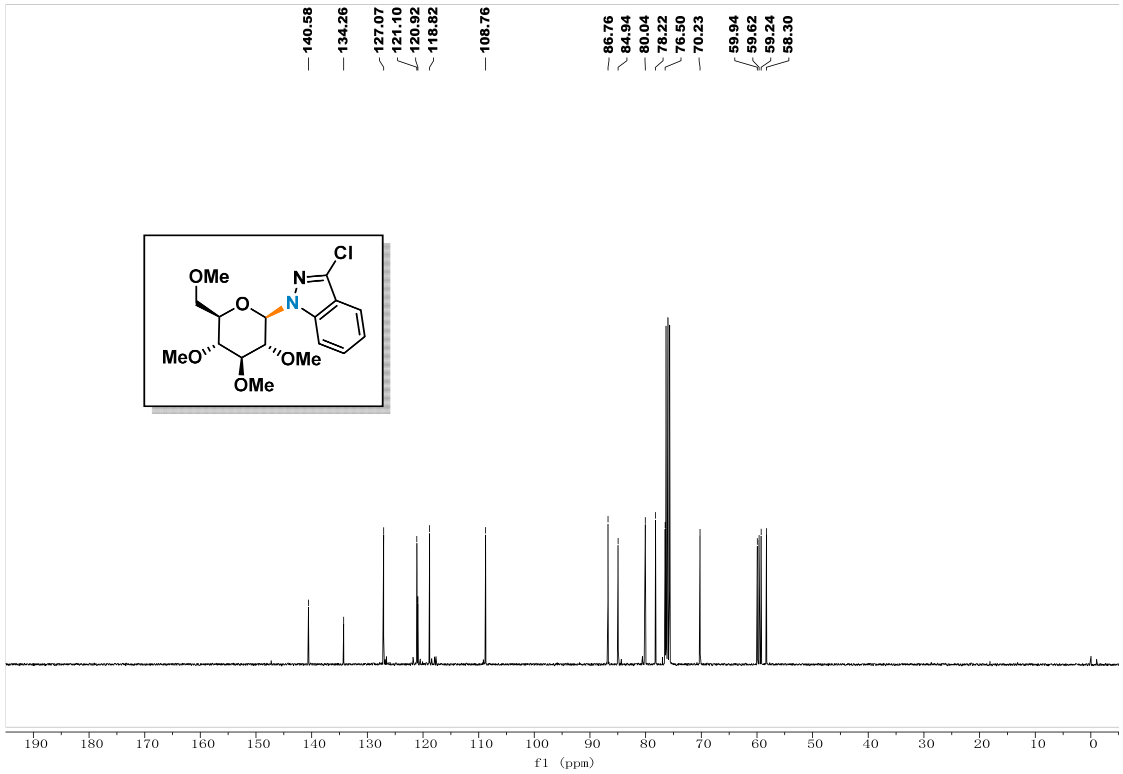

Supplementary Figure 206 | ^{13}C NMR (101 MHz, $CDCl_3$) **(8pb)**

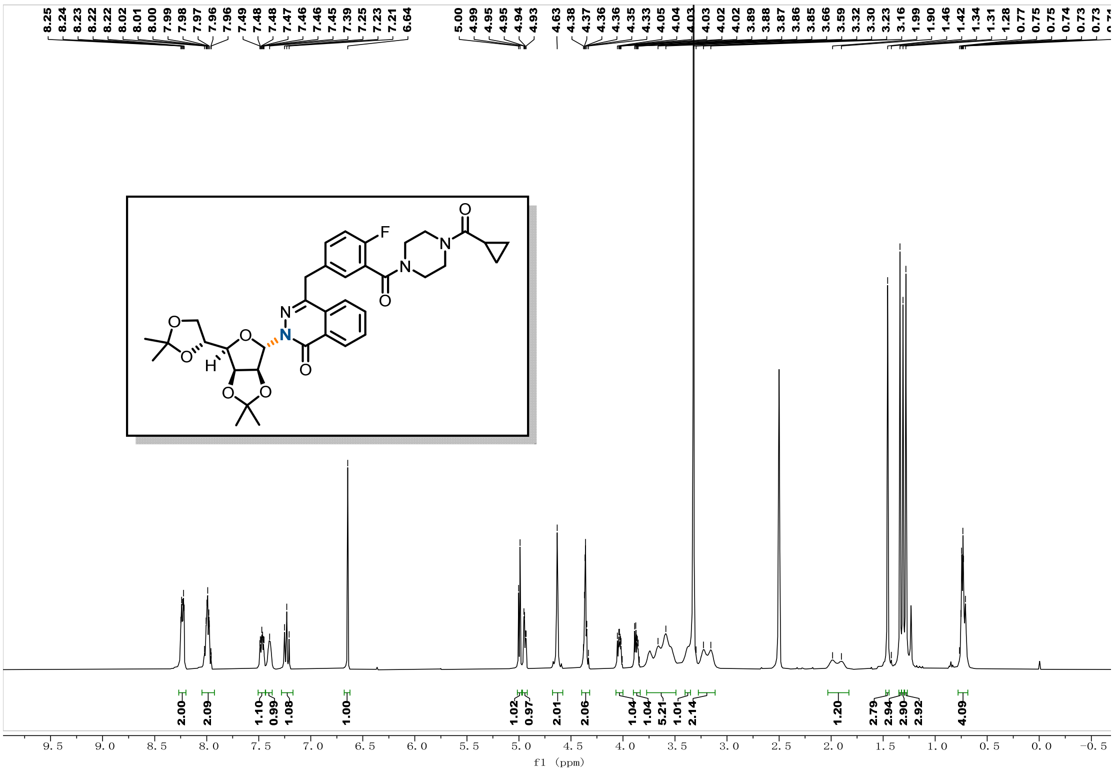

Supplementary Figure 207 | ^{1}H NMR (400 MHz, DMSO-d_6) **(9a)**

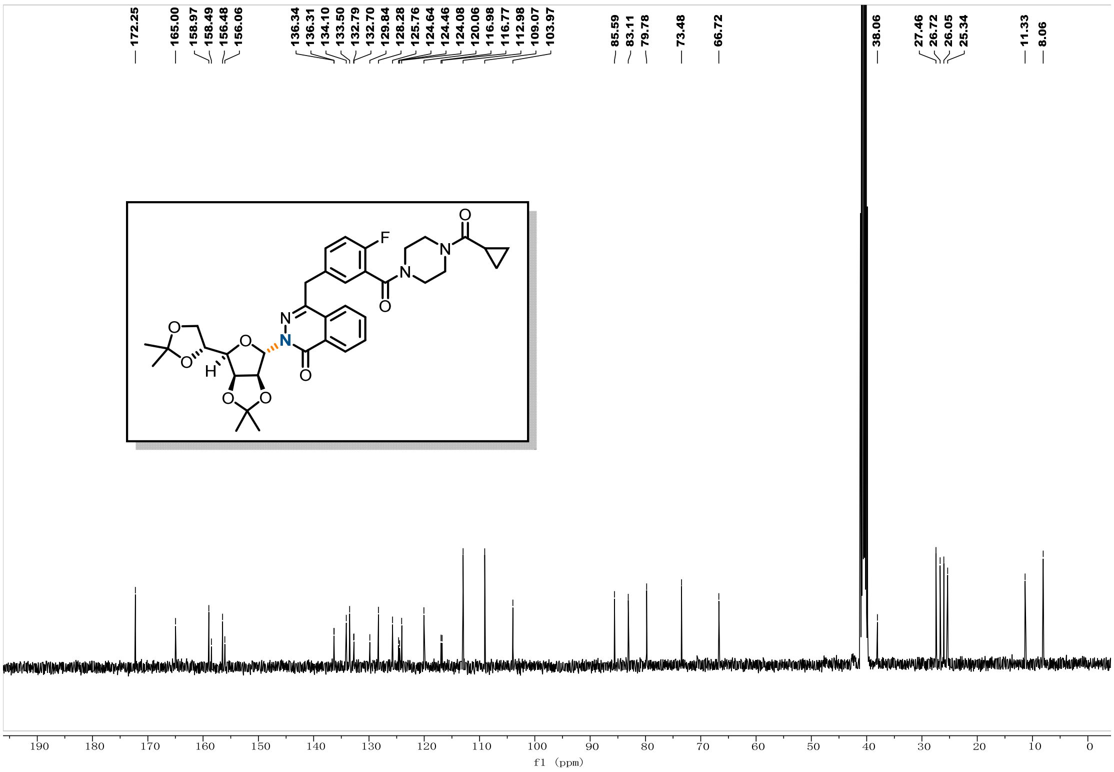

Supplementary Figure 208 | ^{13}C NMR (101 MHz, DMSO-d_6) **(9a)**

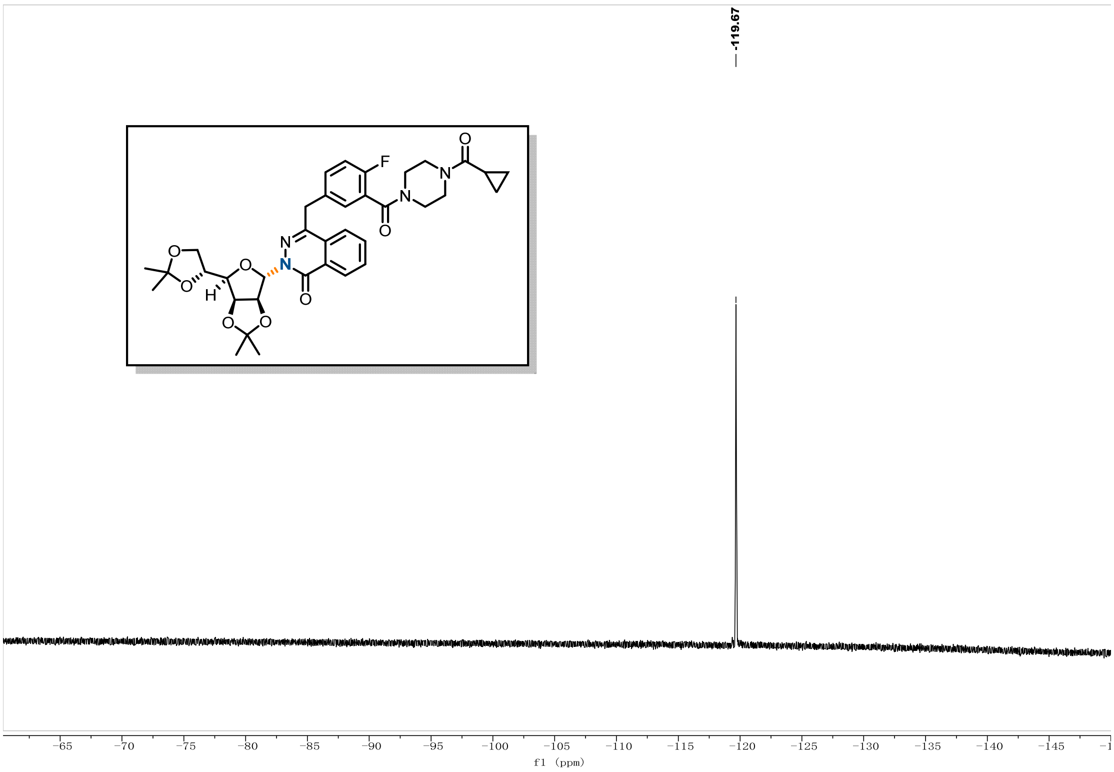

Supplementary Figure 209 | ^{19}F NMR (376 MHz, DMSO-d_6) **(9a)**

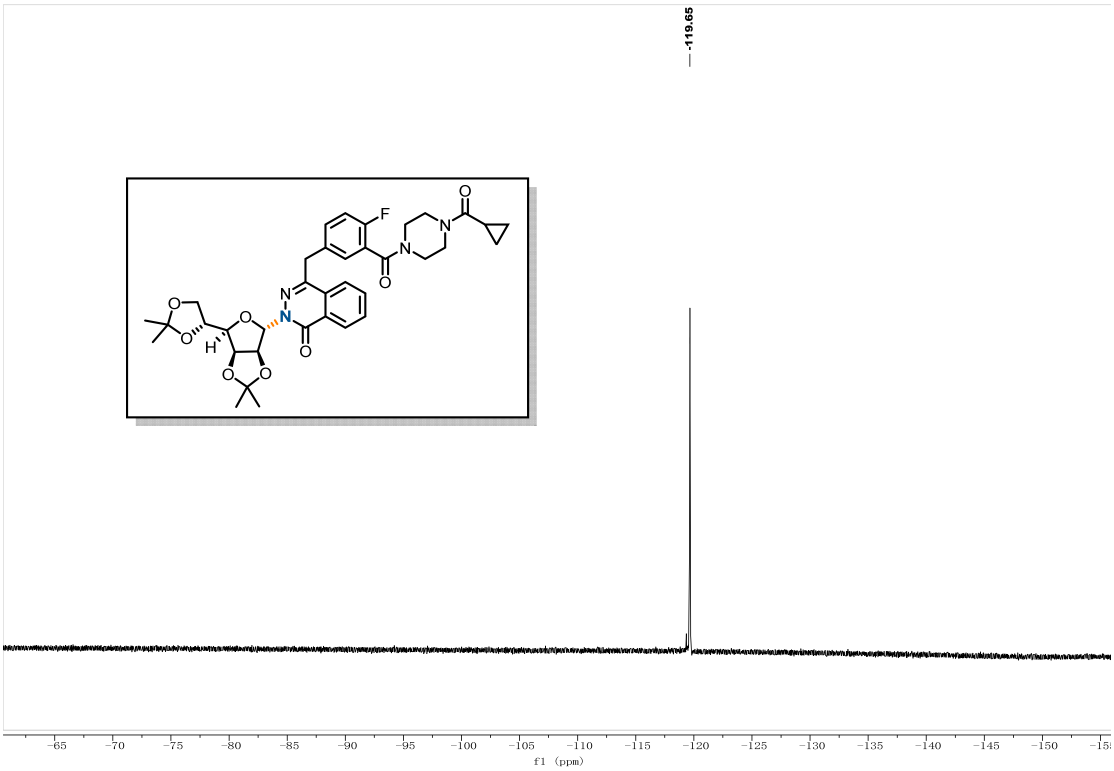

Supplementary Figure 210 | ^{19}F {^{1}H} NMR (376 MHz, DMSO-d_6) **(9a)**

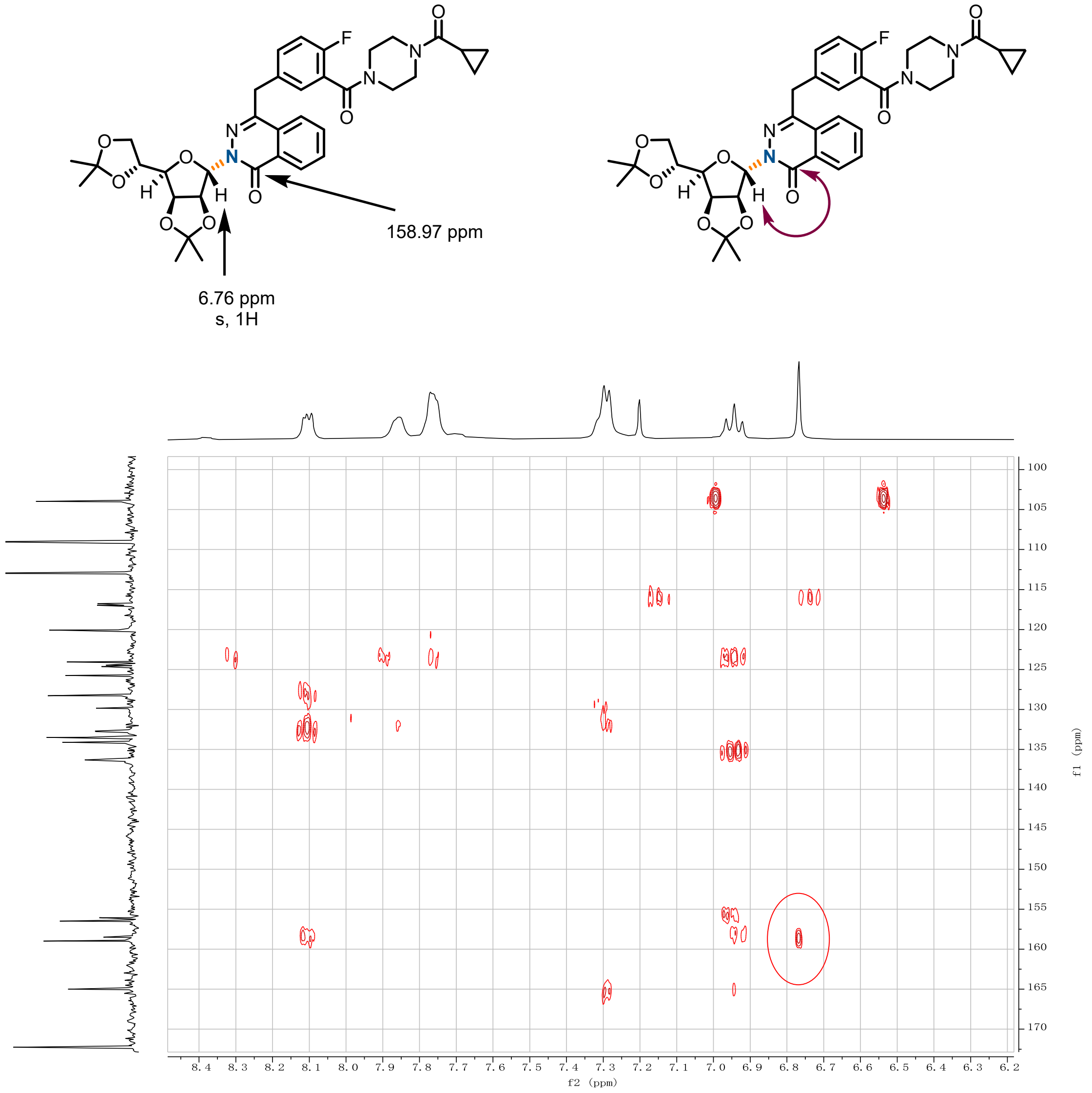

Supplementary Figure 211 | HMBC of **9a**

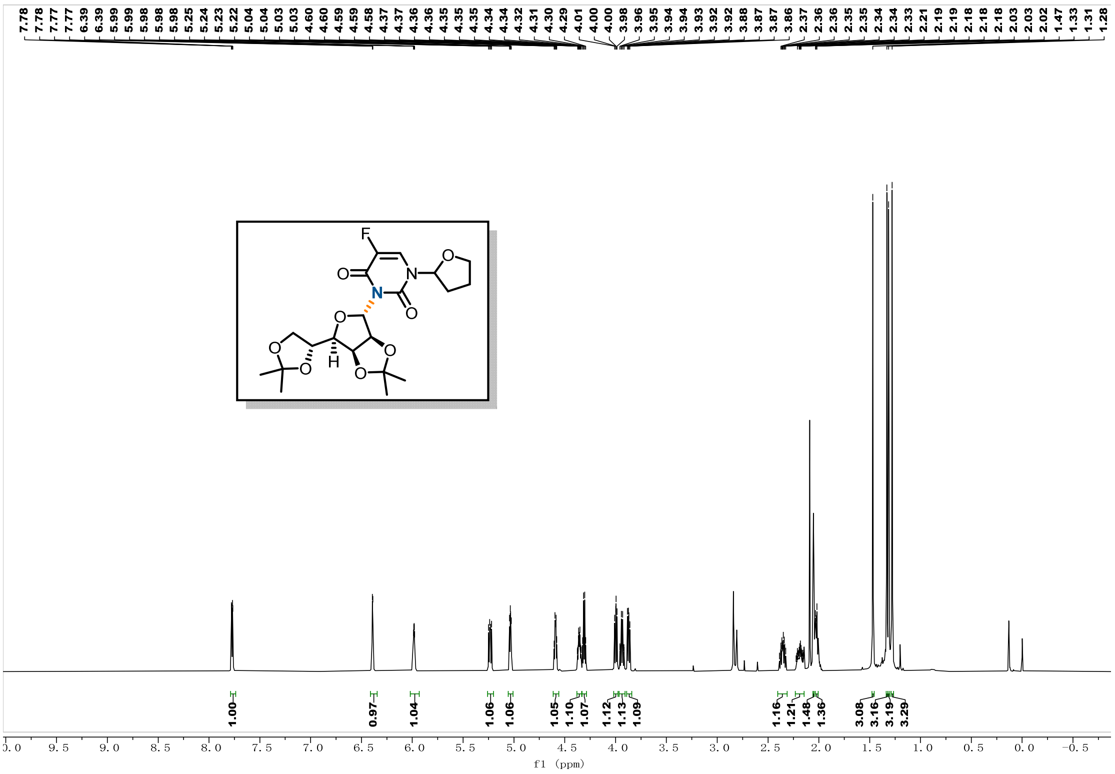

Supplementary Figure 212 | ^{1}H NMR (600 MHz, Acetone-d_6) **(9b)**

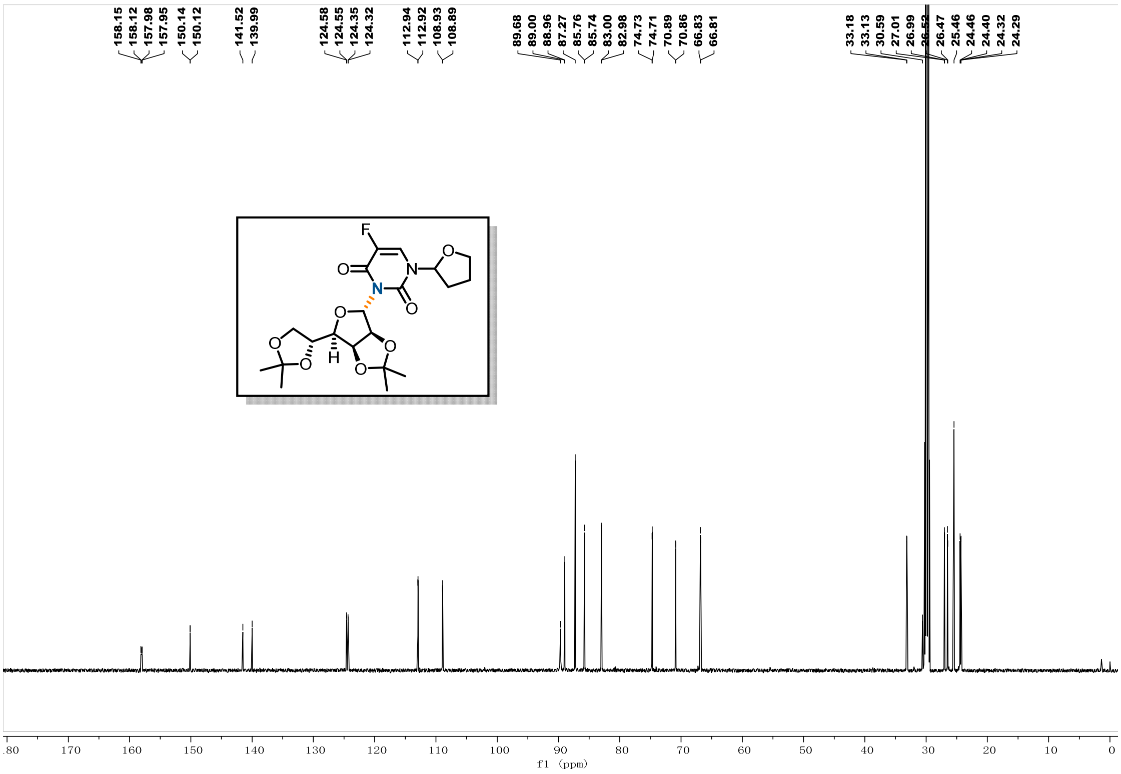

Supplementary Figure 213 | ^{13}C NMR (151 MHz, Acetone-d_6) **(9b)**

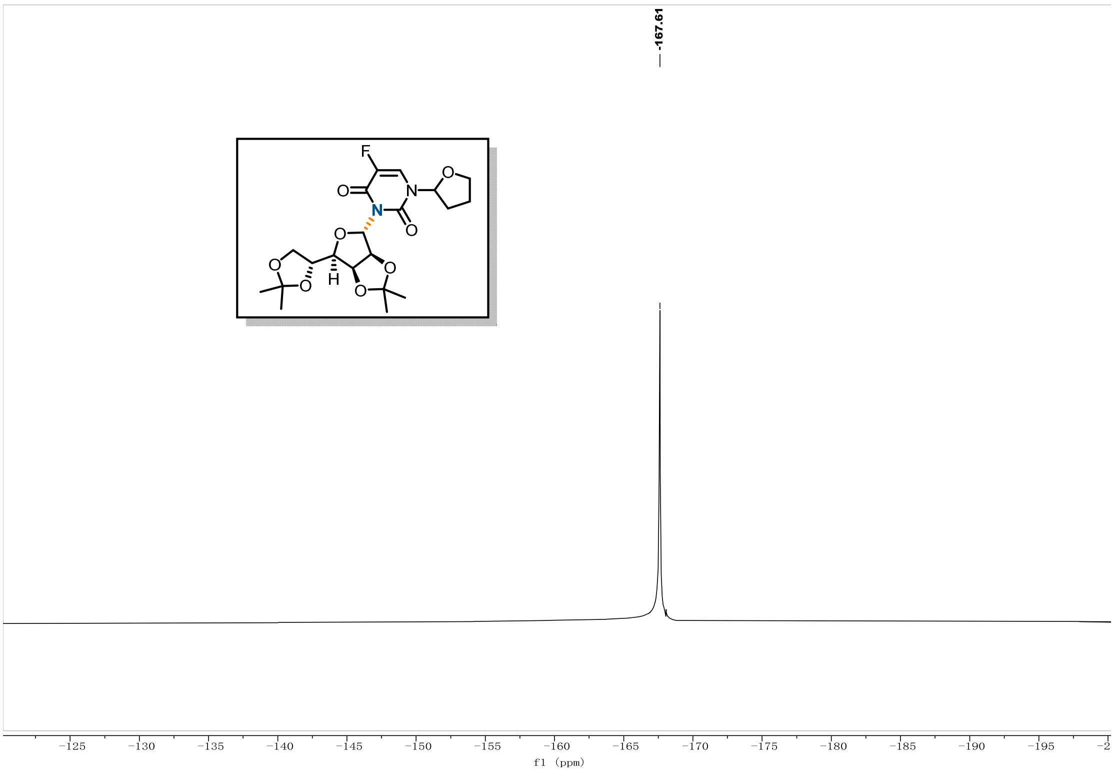

Supplementary Figure 214 | ^{19}F NMR (565 MHz, Acetone-d_6) **(9b)**

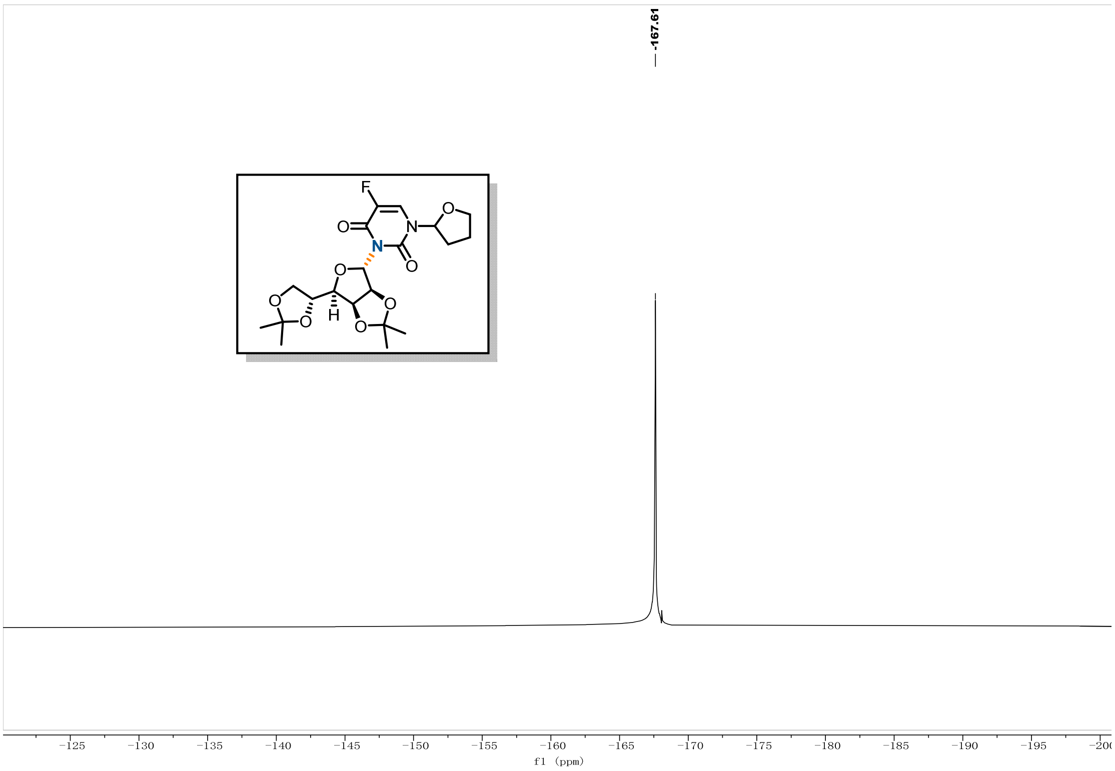

Supplementary Figure 215 | $^{19}F\{^1H\}$ NMR (565 MHz, Acetone-d_6) **(9b)**

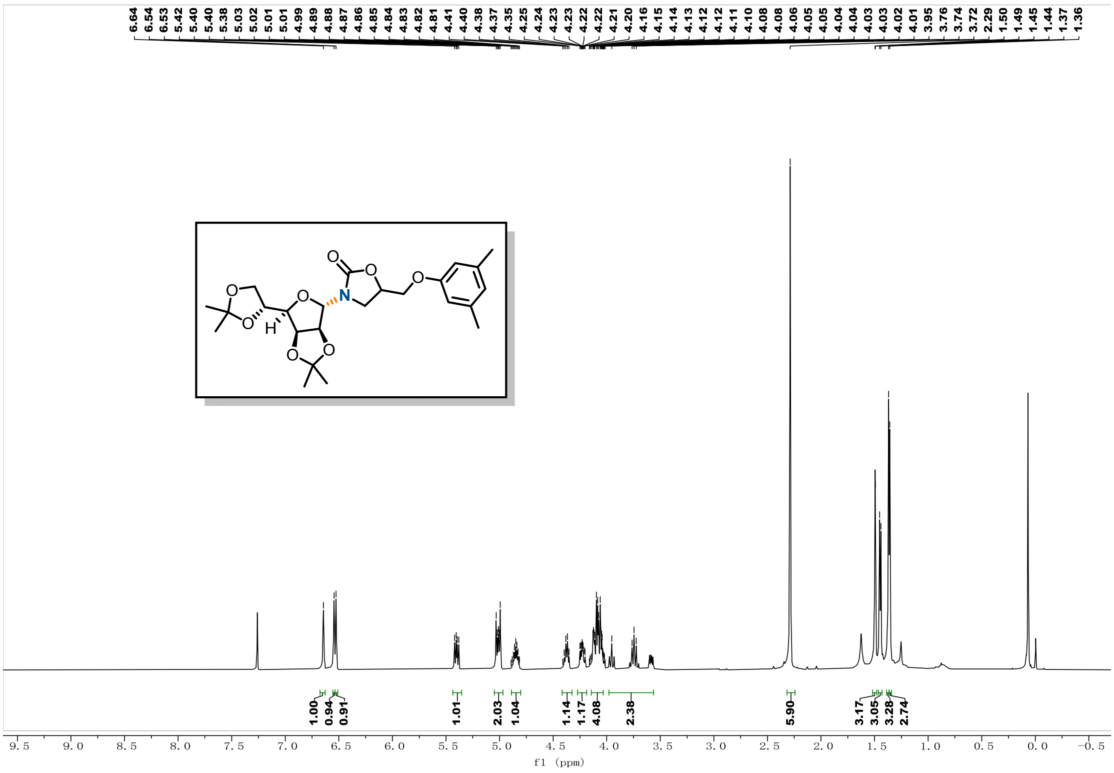

Supplementary Figure 216 | ^{1}H NMR (400 MHz, CDCl$_3$) **(9c)**

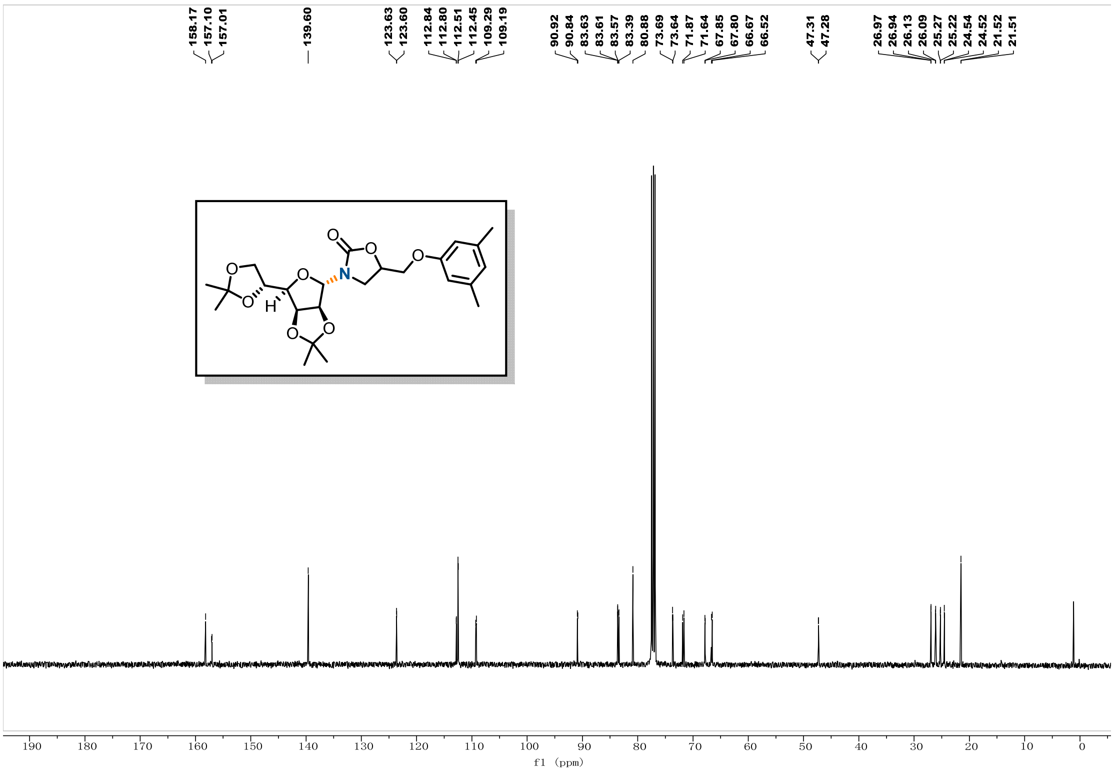

Supplementary Figure 217 | ^{13}C NMR (101 MHz, CDCl$_3$) **(9c)**

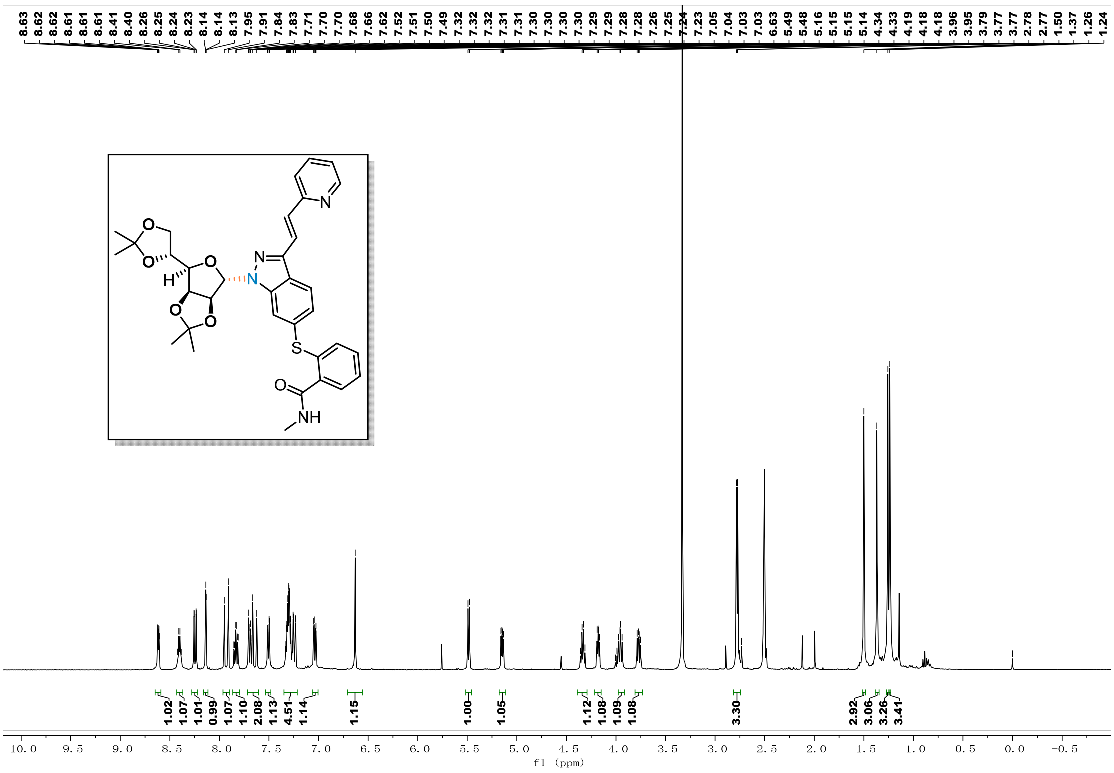

Supplementary Figure 218 | ^{1}H NMR (400 MHz, DMSO-d_6) **(9d)**

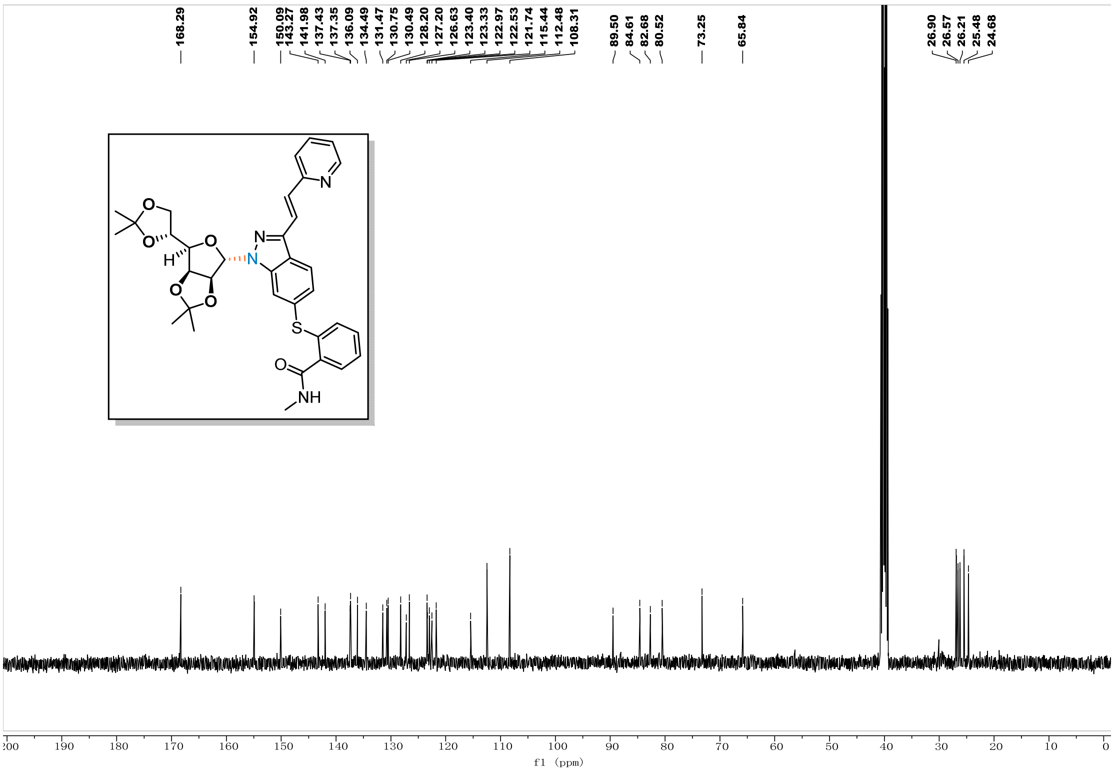

Supplementary Figure 219 | ^{13}C NMR (101 MHz, DMSO-d_6) **(9d)**

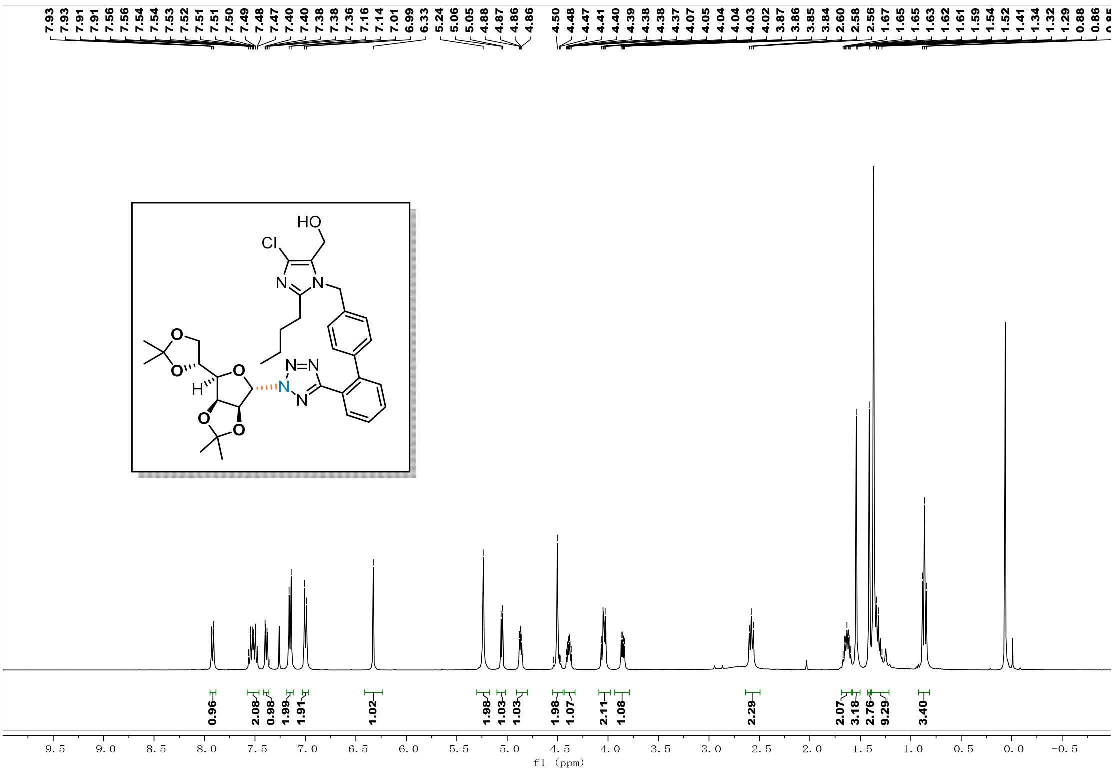

Supplementary Figure 220 | ^{1}H NMR (400 MHz, $CDCl_3$) **(9e)**

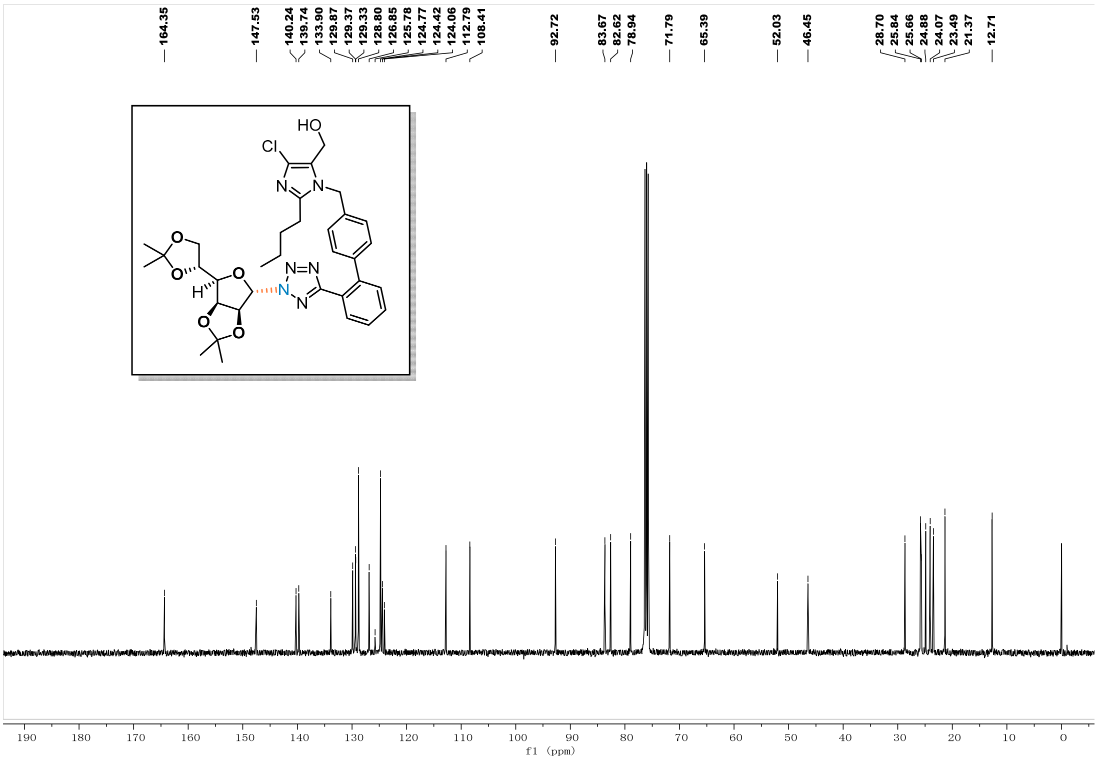

Supplementary Figure 221 | ^{13}C NMR (101 MHz, $CDCl_3$) **(9e)**

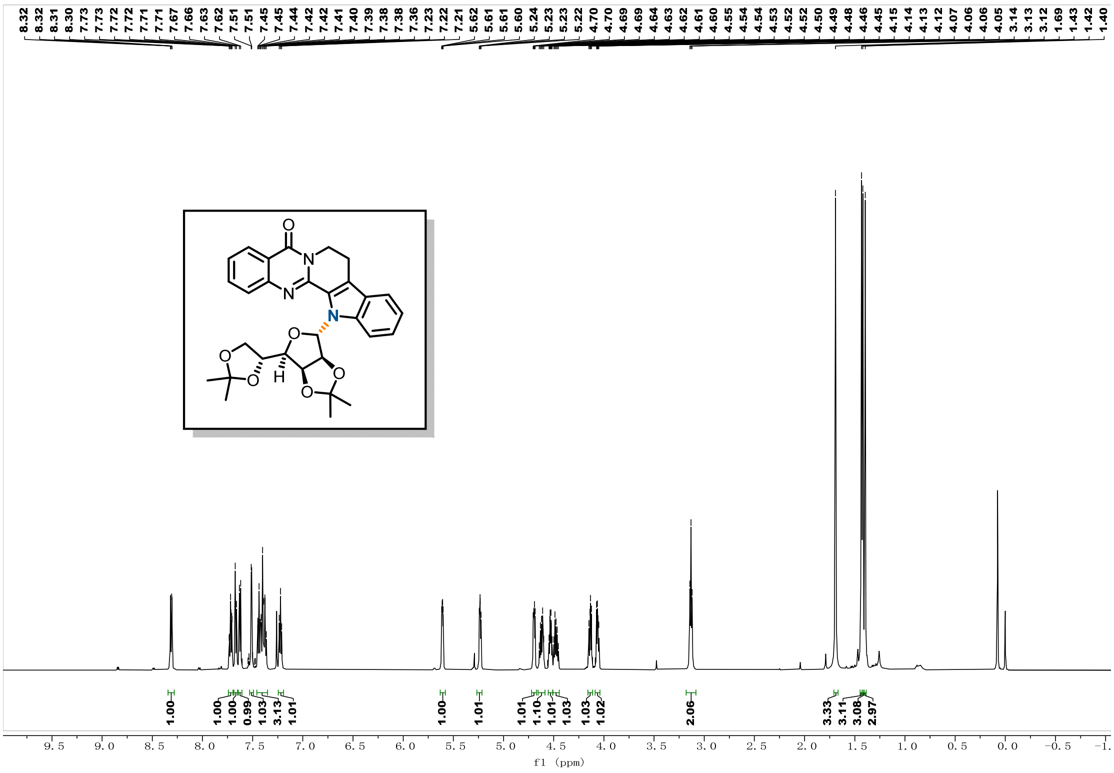

Supplementary Figure 222 | ^{1}H NMR (600 MHz, $CDCl_3$) **(9f)**

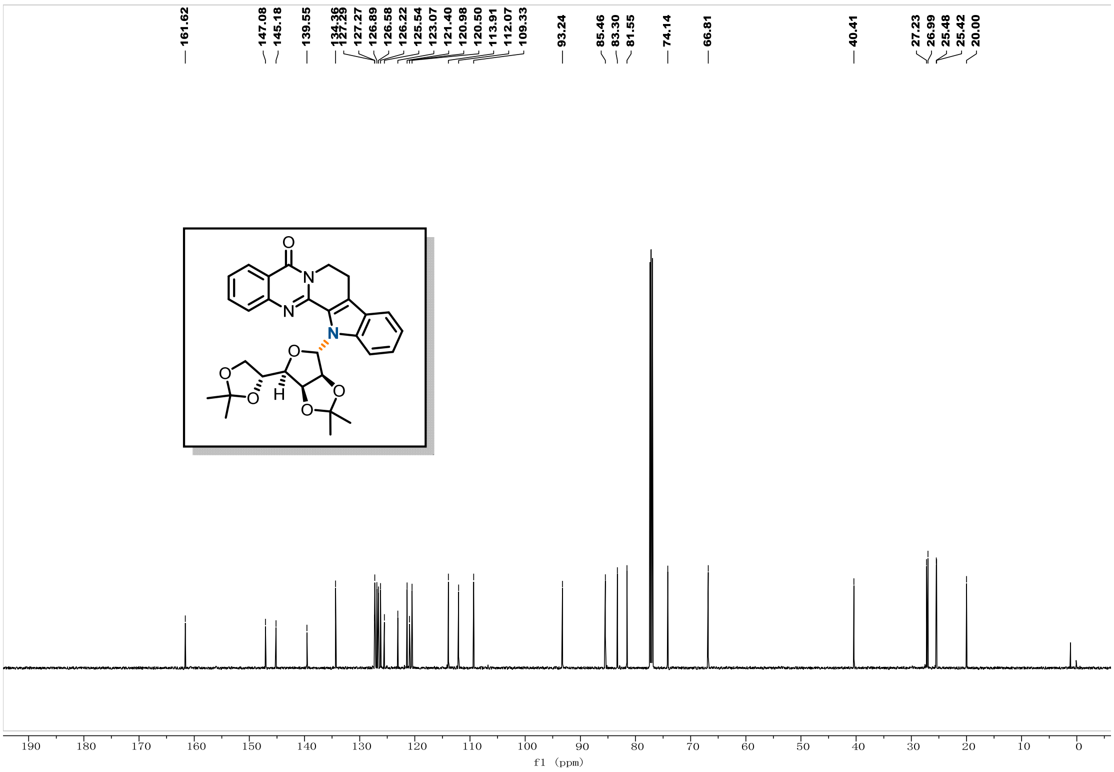

Supplementary Figure 223 | ^{13}C NMR (151 MHz, $CDCl_3$) **(9f)**

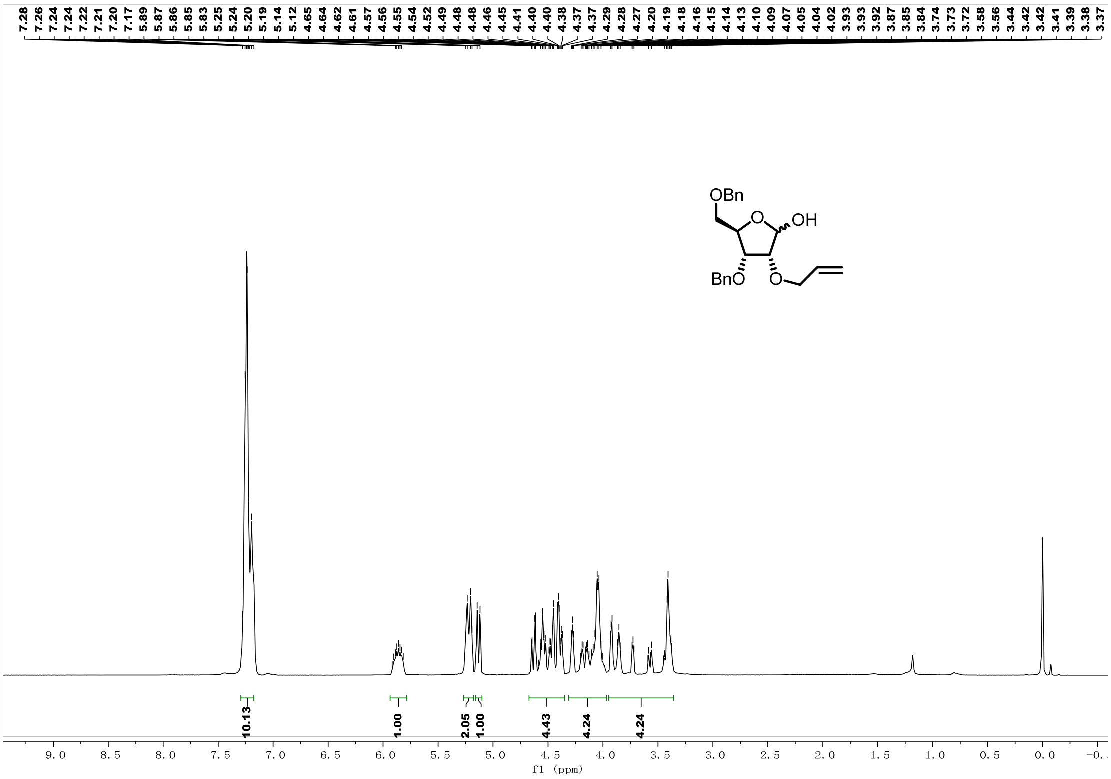

Supplementary Figure 224 | ^{1}H NMR (400 MHz, CDCl$_3$) **(10)**

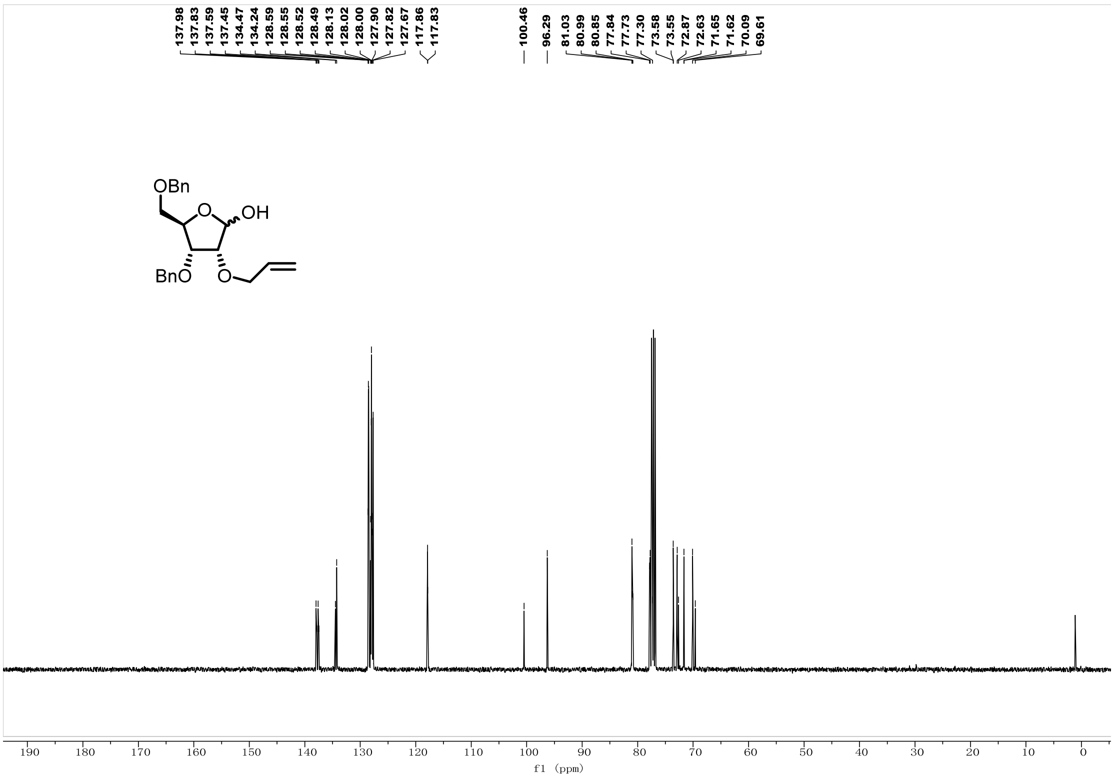

Supplementary Figure 225 | ^{13}C NMR (101 MHz, CDCl$_3$) **(10)**

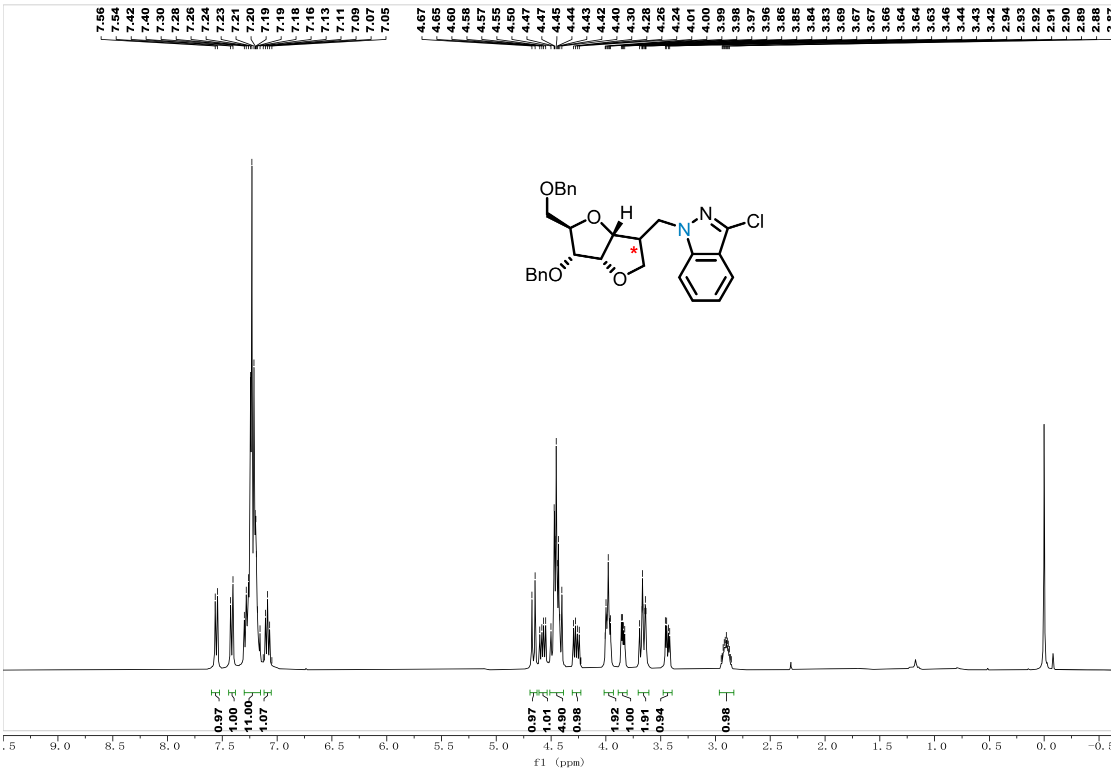

Supplementary Figure 226 | ^{1}H NMR (400 MHz, $CDCl_3$) **(11a)**

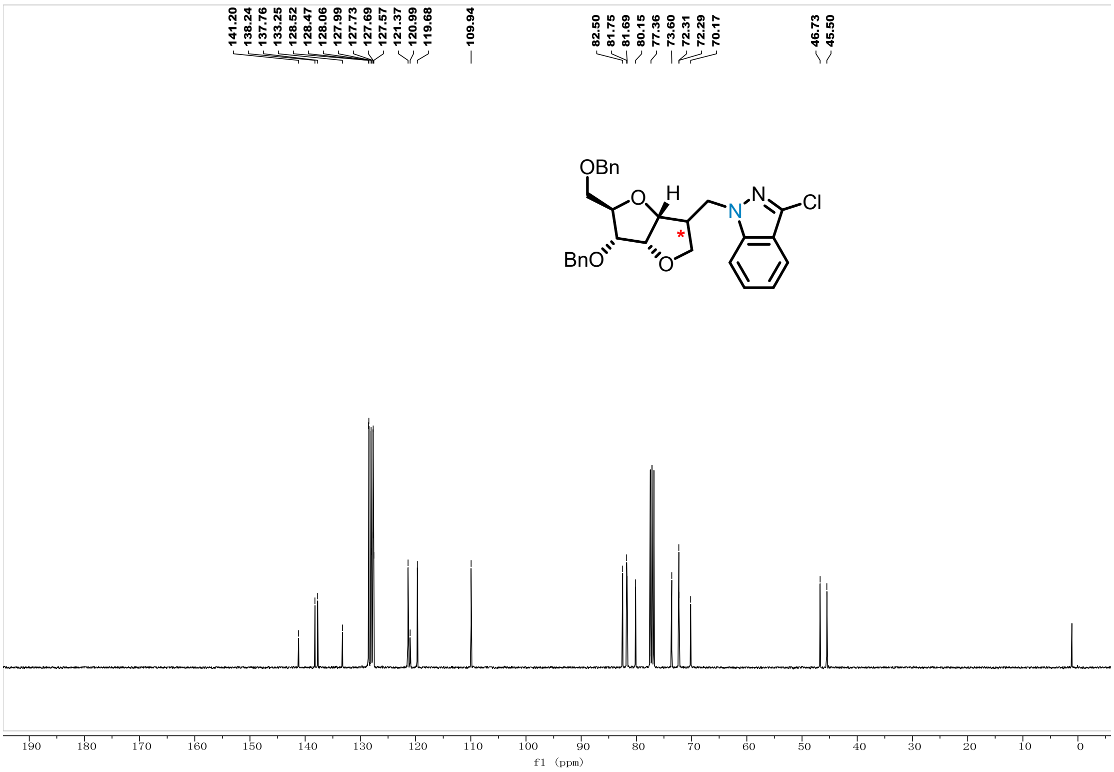

Supplementary Figure 227 | ^{13}C NMR (101 MHz, $CDCl_3$) **(11a)**

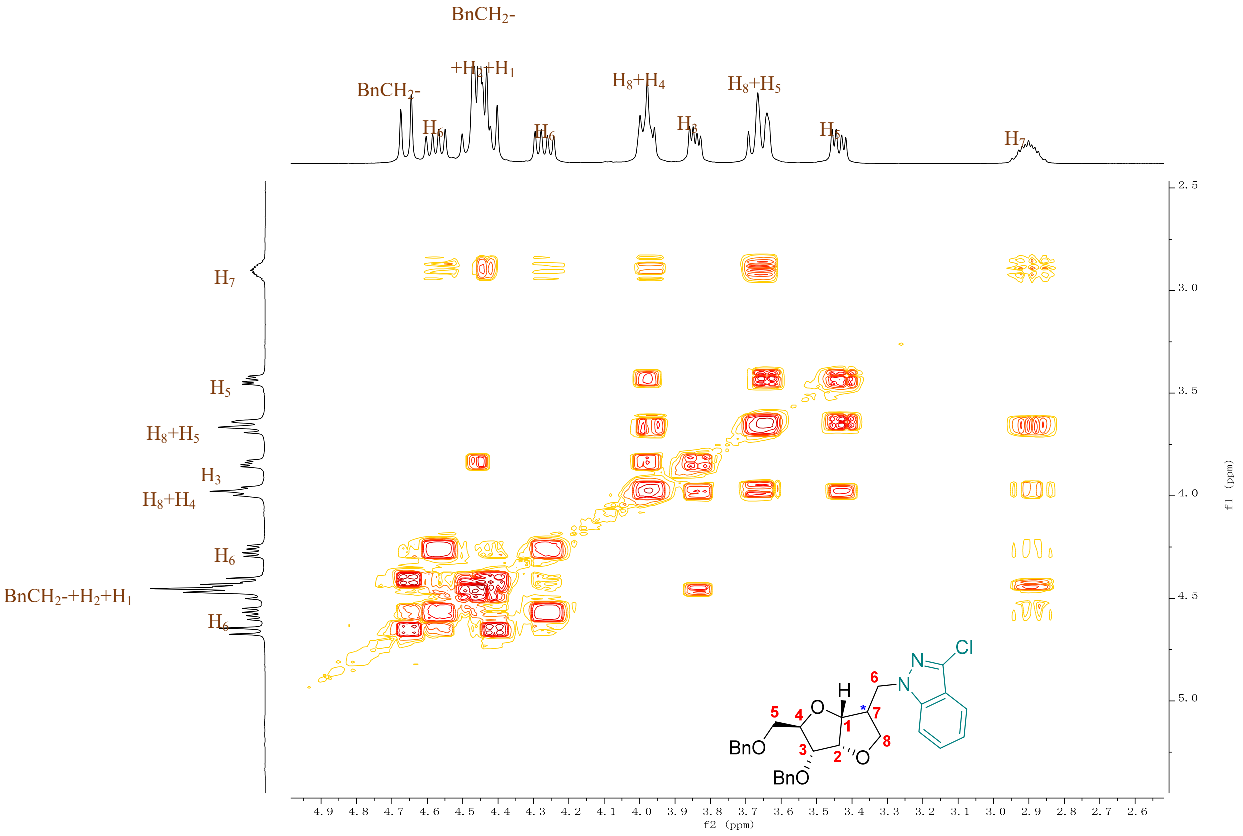

Supplementary Figure 228 | ^{1}H-^{1}H COSY of **11a**

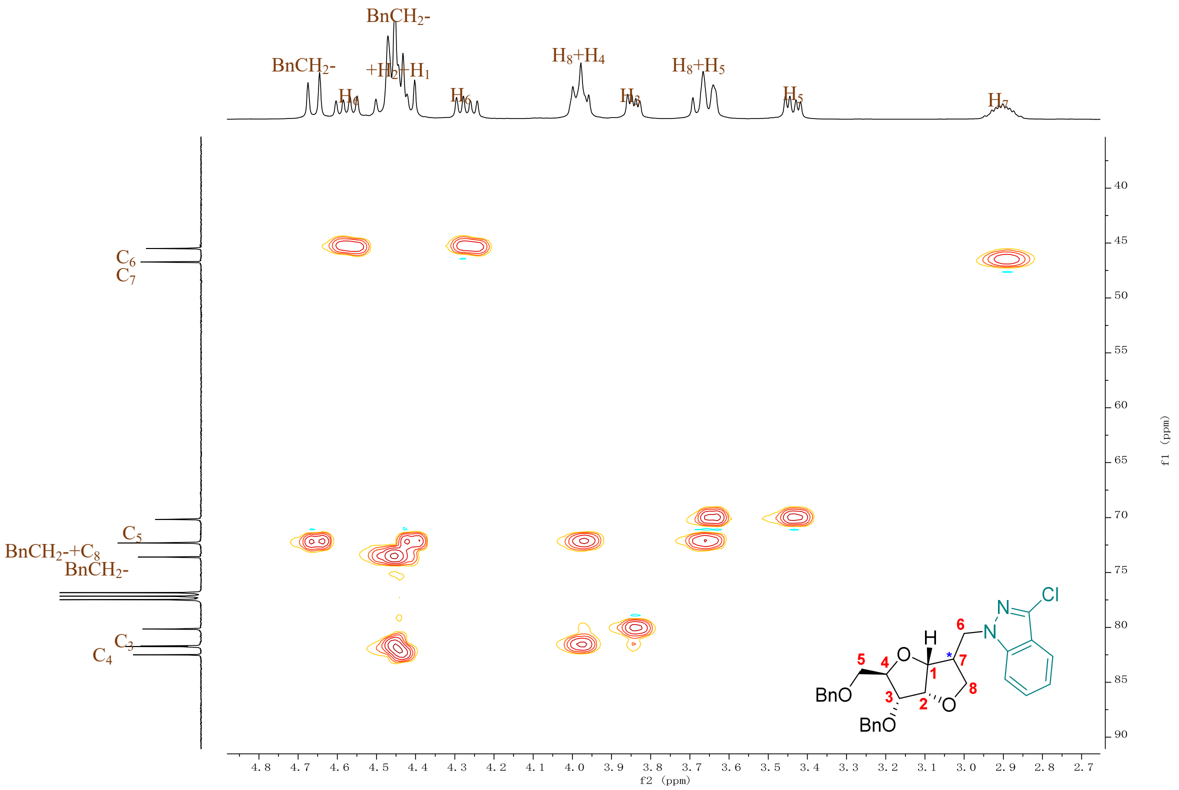

Supplementary Figure 229 | HSQC of **11a**

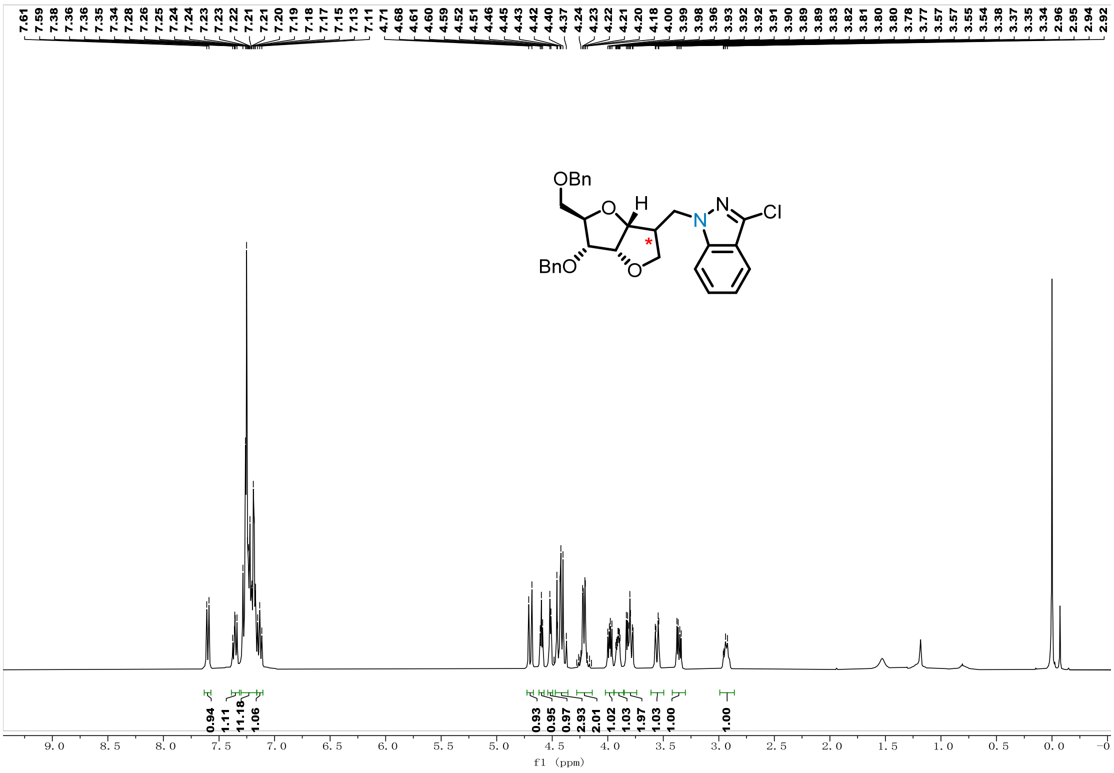

Supplementary Figure 230 | ^{1}H NMR (400 MHz, $CDCl_3$) **(11b)**

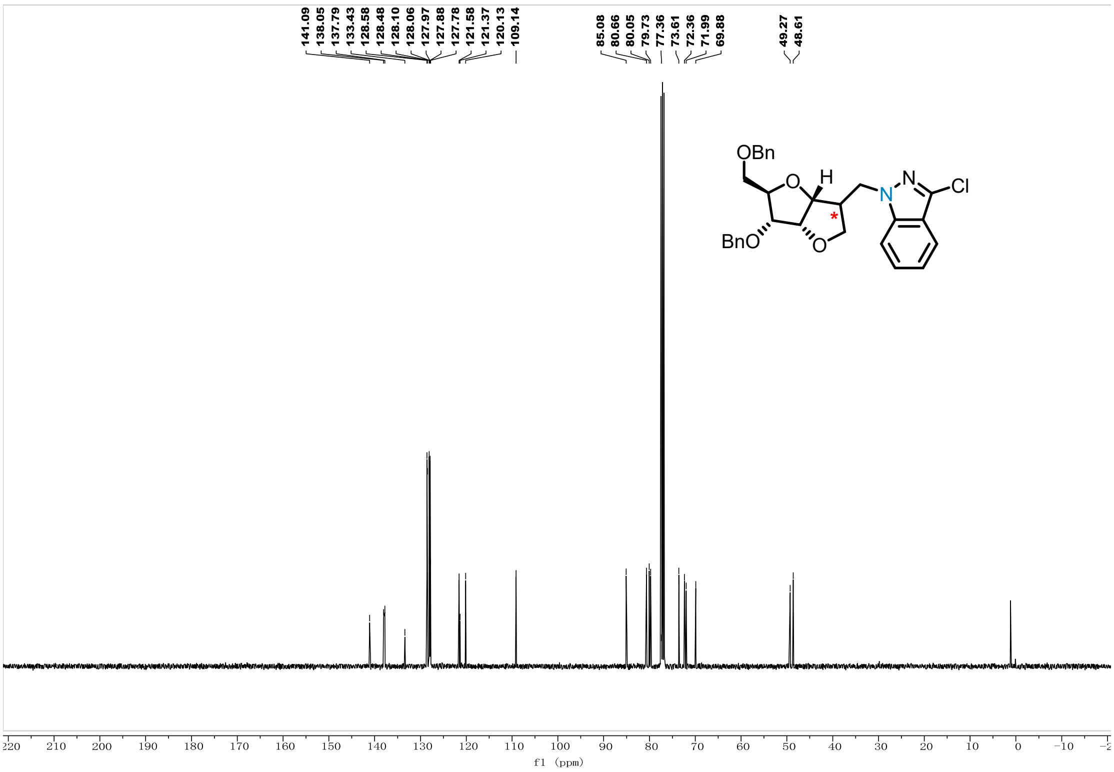

Supplementary Figure 231 | ^{13}C NMR (101 MHz, $CDCl_3$) **(11b)**

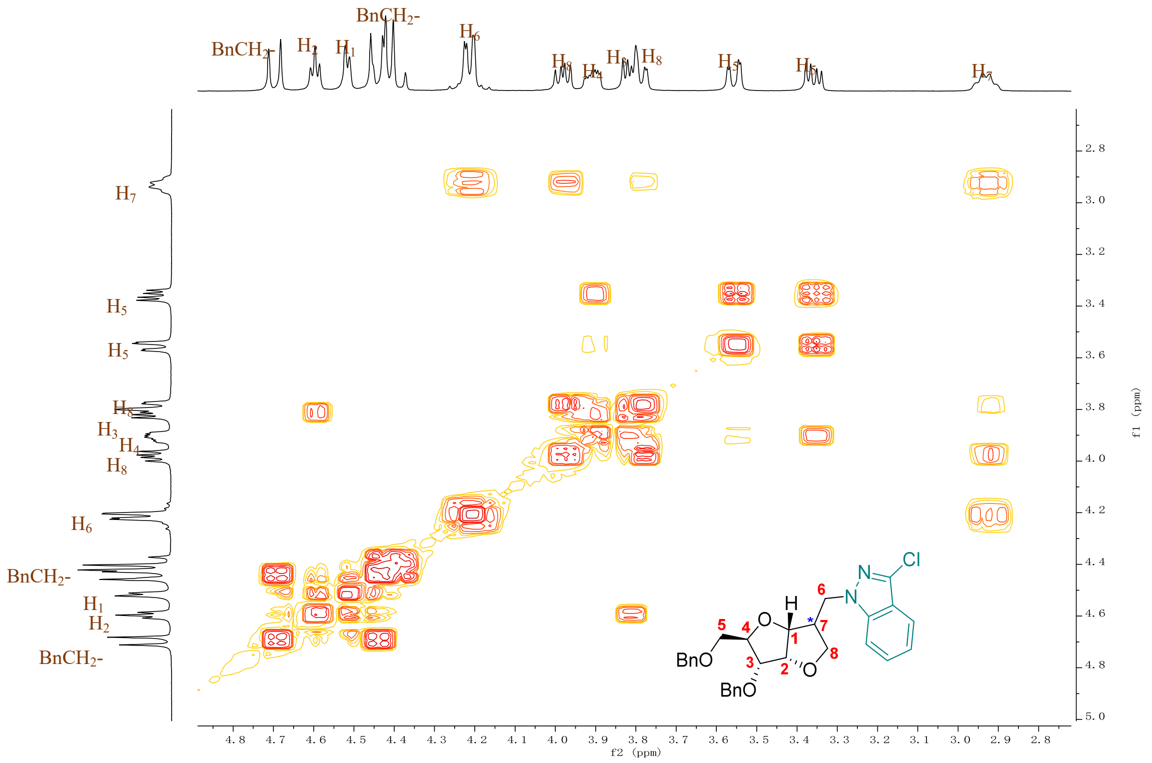

Supplementary Figure 232 | ^{1}H-^{1}H COSY of **11b**

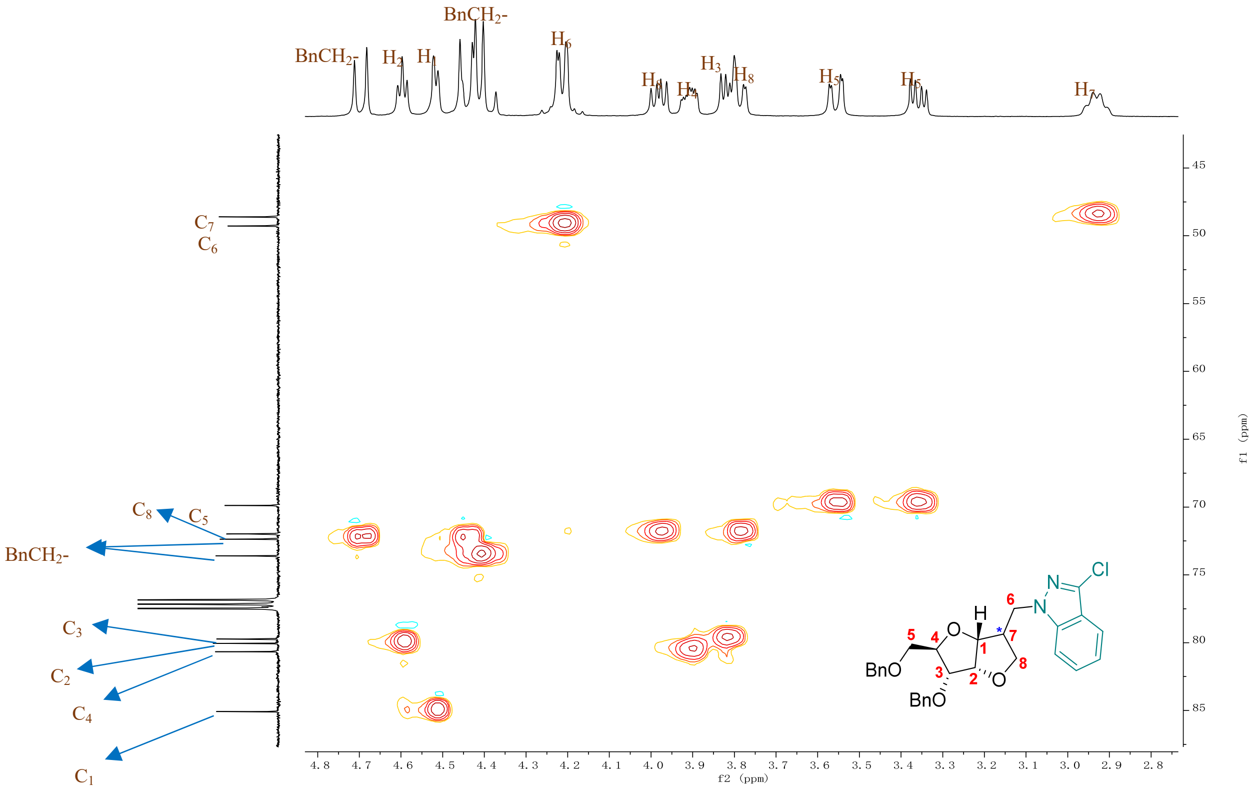

Supplementary Figure 233 | HSQC of **11b**

6. References

[1] Dong, Z. & MacMillan, D. W. C. Metallaphotoredox-enabled deoxygenative arylation of alcohols. *Nature*, 451-456 (2021).
[2] Intermaggio, N. E., Millet, A., Davis, D. L. & MacMillan, D. W. C. Deoxytrifluoromethylation of Alcohols. *J. Am. Chem. Soc.* **144**, 11961-11968 (2022).
[3] Lyon, W. L. & MacMillan, D. W. C. Expedient Access to Underexplored Chemical Space: Deoxygenative C(sp(3))-C(sp(3)) Cross-Coupling. *J. Am. Chem. Soc.* **145**, 7736-7742 (2023).
[4] Li, Y., Wang, Z., Li, L., Tian, X., Shao, F. & Li, C., Chemoselective and Diastereoselective Synthesis of C-Aryl Nucleoside Analogues by Nickel-Catalyzed Cross-Coupling of Furanosyl Acetates with Aryl Iodides. *Angew. Chem. Int. Ed.* **61**, e202110391 (2022).
[5] Nawrat, C. C., Jamison, C. R., Slutskyy, Y., MacMillan, D. W. C. & Overman, L. E., Oxalates as Activating Groups for Alcohols in Visible Light Photoredox Catalysis: Formation of Quaternary Centers by Redox-Neutral Fragment Coupling. *J. Am. Chem. Soc.* **137**, 11270-11273 (2015).
[6] Guo, H. M. & Wu, X., Selective deoxygenative alkylation of alcohols via photocatalytic domino radical fragmentations. *Nat. Commun.* **12**, 5365 (2021).
[7] Wei, Y., Ben-Zvi, B. & Diao, T., Diastereoselective Synthesis of Aryl C-Glycosides from Glycosyl Esters via C-O Bond Homolysis. *Angew. Chem. Int. Ed.* **60**, 9433-9438 (2021).
